# Supplementary material for: Coupling metabolomics and exome sequencing reveals graded effects of rare damaging heterozygous variants on gene function and human traits
Source: Nat Genet. 2025 Jan 2;57(1):193–205. doi: 10.1038/s41588-024-01965-7 (PMC11735408; doi:10.1038/s41588-024-01965-7)
Supplement: Supplementary file 1 — Supplementary Methods, Results, Discussion, Note and Figs. 1–3 [file 41588_2024_1965_MOESM1_ESM.pdf]

nature genetics

Supplementary information https://doi.org/10.1038/s41588-024-01965-7

Coupling metabolomics and exome sequencing reveals graded effects of rare damaging heterozygous variants on gene function and human traits

In the format provided by the authors and unedited

Table of Contents:

Supplementary Methods 2

Additional gene-based test 2
Stratified analyses 2
Comparison to previous association studies 3
Whole-body modeling 5
Absolute metabolite quantification for members of a family with the KYNU-attributed IEM .. 5
Analysis of phenylalanine in serum for members of a family with the PAH-attributed IEM 6
Relation of genes and variants to clinical traits, diseases and protein levels 7

Supplementary Results 8

Sensitivity analyses for gene-based testing: LoF only, SKAT, SKAT-O tests 8
Stratified analyses to investigate potential subgroup-specific effects 9
Comparison to previous rare variant studies 10
Variant characterization of gene-metabolite associations 10
Curation of whole body modeling based on the GCKD data 11
Modeling of 8-methoxykynurenate in the virtual IEM for kynureninase deficiency (KYNU) ... 12
Modeling of N-formylanthranilic acid in the virtual IEM for AFMID 13
Modeling of the virtual IEM for TMLHE 13
Modeling of dimethylglycine in the virtual IEM for dimethylglycine dehydrogenase deficiency (DMGDH) 14
Modeling of the virtual IEM for KYAT1 15
Modeling of hexanoylglycine in the virtual IEM for medium-chain acyl-CoA dehydrogenase deficiency (ACADM) 15
Modeling of the virtual IEM for ACY1 and N-acetylisoleucine 15
Personalized WBMs capture observed metabolic changes - PAH 16
Association of metabolite-associated variants and genes with human traits 16

Supplementary Discussion 18

Potential limitations of the study 18

Supplementary Acknowledgements 21

List of GCKD Study Investigators 21

Supplementary References 22

Supplementary Figures 26

Supplementary Figure 1: Number of significantly associated metabolites by matrix/matrices and biochemical super-pathway. 27
Supplementary Figure 2: Predicted changes upon *in silico* knockout modeling and observed effect sizes based on aggregate variant testing in the GCKD study. 28
Supplementary Figure 3: Contribution of individual QVs in *SLC13A1* and *SLC26A1* to their gene-based association signal with height measured in the GCKD study. 30
Supplementary Data 1: Plasma and urine metabolite levels among carriers and non-carriers of QVs in significantly associated genes. 31
Supplementary Data 2: Contribution of individual QVs to their gene-based association signal with plasma and urine metabolite levels. 31

Supplementary Methods

Additional gene-based test

As sensitivity analyses, we performed burden tests using a LoF mask, containing only high-confidence loss-of-function variants, for all significant gene-metabolite associations detected across matrices and masks in the main analyses. Moreover, SKAT and SKAT-O tests as implemented in the seqMeta R-package version 1.6.7[1] were carried out. Results from these sensitivity analyses are included in **Supplementary Table 4**.

Stratified analyses

Further sensitivity analyses evaluated the presence of differences for the significant associations identified in all main analyses across strata of sex and eGFR (≤/>45 ml/min/1.73m^2). Burden tests as implemented in the seqMeta R-package version 1.6.7[1] were calculated for both masks within each stratum, including the same covariates as in the main analyses with the exception of the variable that was used for stratification. Differences between effect sizes across strata were assessed based on the test statistic $Z = (beta_1 - beta_2)/(SE(beta_1)^2 + SE(beta_2)^2)^{1/2}$, with $beta_1$ and $beta_2$ indicating the effect sizes in each stratum and $SE(beta_1)$ and $SE(beta_2)$ their standard errors[2]. The test statistic Z follows approximately a standard normal distribution for large samples, based on which two-sided P-values were computed. Significant differences between effect sizes were defined as P-value <0.05/128 for plasma and P-value <0.05/107 for urine, correcting for the number of tested associations.

Comparison to previous association studies

We compared our significant findings to the findings from eight published genetic studies of the plasma/serum or urine metabolome that focused on rare exonic variant aggregation testing and used sequencing and high-throughput metabolomics data[3–10]. More information on the metabolomics platform, cohort, statistical tests, masks, aggregation units, transformation, covariates, and significance thresholds used in these previous studies can be found in the footnote of **Supplementary Table 6**.

We first assessed whether the genes identified in our study were reported as associated with any metabolite in any of the eight studies at their respective multiple-testing corrected significance threshold, after having mapped all gene names to their current version in Ensembl version 109 using https://www.ensembl.org/biomart/martview. We then ascertained for all matching i.e., previously reported genes whether they were associated with the same metabolite(s) as in our study. Metabolites were matched by biochemical name, with manual curation in case of similar names, and by HMDB ID and Compound ID for metabolites quantified at Metabolon, if available. To quantify the proportion of novel gene-metabolite associations that involve metabolites analyzed in a previous study, we focused on studies with MS-based quantification of plasma/serum metabolites with the same technology[3,5,6,10] to enable exact metabolite matching by biochemical name, HMDB ID, and Compound ID. We thereby compared all previously reported results from the analysis of rare exonic variants, both at the aggregation level as well as at the single-variant level. There were no previous independent studies of sequence-based rare variants and their associations with urine metabolites. Among the selected plasma/serum studies, only Bomba and colleagues[3] made summary statistics for aggregated variants available above their chosen significance threshold, i.e., at less significant levels. For all QVs and genes involved in significant

associations in our study, we extracted the summary statistics for the corresponding metabolite and variant or window and compared effect sizes. We focused on summary statistics from the burden test and the variable threshold (VT) test because of the closest similarity to our approach. For each significant gene-metabolite pair, we compared the GCKD effect size to the one for the window, the test, and the mask (LOF, MLOF, CODING) with the lowest P-value in Bomba *et al*[3].

The presence of common variants associated with the corresponding metabolite(s) in or near the identified genes was assessed by searching for common variants (MAF >1%) within a window of ±500 kb around the gene that were significantly (P-value <5e-8) associated with the implicated metabolite. Common variant associations were based on GWAS of inverse normal transformed metabolite levels in the GCKD study (N = 4,991 for plasma, N = 4,911 for urine) performed with REGENIE v2.2.4[11] under additive modeling, using array-based and TOPmed imputed genotypes with high imputation quality and adjusting for age, sex, and the first three genetic principal components[12]. Gene positions were based on Ensembl version 101. Conditional analyses were performed to assess the influence of common variants on gene-based rare variant association signals, for all 157 gene-metabolite associations across plasma and urine that contained an associated common variant within the gene region. We repeated the burden tests using the same masks, additionally adjusting for genotypes at the common variant. Differences between effect sizes without and with conditioning on common variants were assessed based on the test statistic Z (see paragraph above). Significant differences between effect sizes were defined as P-value <0.05/157.

Whole-body modeling

The implicated genes' loss-of-function were investigated in virtual IEMs generated through organ-resolved sex-specific whole-body models (WBMs) based on the Virtual Metabolic Human database (VMH)[13] using a constraint-based modeling and reconstruction analysis (COBRA) approach[9]. Mapping the gene-metabolite pairs significant in the exome-wide screening onto the VMH database[14], virtual IEMs were created to explore all represented gene-metabolite pairs via *in silico* knockout modeling of the gene's function. For male and female human modeling, the WBM model versions "Harvey_1_04b" and "Harvetta_1_04c" were employed, respectively.

Absolute metabolite quantification for members of a family with the KYNU-attributed IEM

8-methoxykynurenate, xanthurenate, and 3-hydroxykynurenine were quantified in urine samples using high performance liquid chromatography coupled to tandem mass spectrometry (HPLC/MS/MS; Exion LC and 5500+ triple quadrupole MS, AB Sciex, Framingham, MA, USA). Urine samples were diluted 1:10 with water and 10 µL of the diluted samples were injected. HPLC separation was performed at 40 °C on a Force C18 column (100 x 3.0 mm, 3 µm particles, Restek Corporation, Bellefonte, PA, USA) equipped with guard column using water (solvent A) and methanol (solvent B), both containing 0.01 vol% formic acid and 1 mM ammonium formate. The flow rate was 300 µL/min and the linear gradient profile of solvent B was as follows: 0 min 1%, 1 min 1%, 10 min 40%, 12 min 90%, then isocratic at 90% until re-equilibration. The analytes were detected using positive ion electrospray ionization (5500 V and 350 °C, nitrogen curtain and ion source gas, declustering potential 1.0 V, entrance potential 10 V) and the multiple reaction monitoring mode (nitrogen collision gas). Compound-specific MS parameters are given in **Supplementary Note Table 1**.

Supplementary Note Table 1. Mass spectrometric parameters for detection and quantification of the analytes

		Precursor ion [*m/z*]	Product ion [*m/z*]	Collision energy [V]	Collision cell exit potential [V]
8-methoxy-kynurenate	Quantifier	220.0	174.1	27	12
	Qualifier	220.0	118.1	39	14
Xanthurenate	Quantifier	206.0	160.1	27	12
	Qualifier	206.0	132.1	39	10
3-hydroxy kynurenine	Quantifier	225.0	162.1	29	10
	Qualifier	225.0	110.1	19	10

Quantification was based on external 4-point calibration curves covering the ranges of detected signal abundances in the samples. Quantitative results were normalized to urine creatinine concentrations (expressed as mmol/mol creatinine) before comparison between samples. Legal guardians of the proband consented for genetic and urine analyses.

Analysis of phenylalanine in serum for members of a family with the PAH-attributed IEM

Phenylalanine was quantified in serum samples using ion chromatography with post-column addition of ninhydrin and subsequent photometric detection (Biochrome 30+ amino acid analyzer, Biochrom Ltd., Cambridge, UK). A volume of 200 µL serum was mixed with 50 µL 10% sulfosalicylic acid (for denaturation of proteins) and 25 µL internal standard (S-2-aminoethyl-L-cysteine) solution. After centrifugation for 1 min at 2655 x g, 100 µL of the supernatant were mixed with 200 µL dilution buffer and 40 µL of the resulting solution were injected. Cation exchange ion chromatography was performed on a lithium column (prod. No. 40016551, Biochrom Ltd.) equipped with a guard column at a flow rate of 20 mL/h with a temperature and pH gradient using five different lithium citrate buffers and a lithium hydroxide solution. Post-column addition of the ninhydrin solution was also performed at a

flow rate of 20 mL/h. Primary amino acids (e. g. phenylalanine) were detected at 570 nm. Quantification was based on internal standard methods using an external 1-point calibration.

Relation of genes and variants to clinical traits, diseases and protein levels

We used different data sources to link the associated genes and QVs identified in our study to clinical outcomes and diseases. Implicated genes were queried for related monogenic disorders and traits using the OMIM catalog (https://www.omim.org/; accessed on 1/6/2022), and for the presence of known IEMs using https://panelapp.genomicsengland.co.uk/panels/467/ version v4.0. Drug target status and the corresponding indication were annotated for all identified genes by querying https://platform.opentargets.org/ on 7/12/2022. Clinical significance and the corresponding trait or disease were annotated for all qualifying variants based on ClinVar https://www.ncbi.nlm.nih.gov/clinvar/ accessed on 3/30/2022. Fisher's exact test was used to test whether metabolite-associated genes were overrepresented among genes known to be causative for IEMs, defined as those with high ("green") and moderate ("amber") evidence in the Genomics England panel https://panelapp.genomicsengland.co.uk/panels/467/ version v4.0 and present among the 16,525 genes analyzed in our study.

We additionally searched for gene-level and variant-level associations of the genes and QVs identified in our study with about 15,500 binary and 1,500 continuous phenotypes contained in the AstraZeneca PheWAS Portal (https://azphewas.com/; downloaded on 26/08/2022, v4 450k). This portal contains genetic associations identified based on whole-exome sequencing data from ~450,000 UK Biobank (UKB) participants.[15] Binary phenotypes with <30 cases were excluded from both gene- and variant-level analysis. At the variant level, associations were restricted to those identified in at least 30 samples. For gene-level and

variant-level associations, we only extracted the most significant collapsing model and genotype model per trait, respectively. Statistical significance was defined as P-value <2e-09[15], and suggestive significance as P-value <1e-05. Fisher's exact test was used to test whether metabolite-associated genes were overrepresented among genes associated with binary traits in the UKB at suggestive significance (P-value <1e-05).

Moreover, we searched for *cis*-associations of the metabolite-associated genes with plasma protein levels in the UKB to investigate whether damaging variants influencing metabolite levels also result in altered plasma protein levels. We used gene-level summary statistics of protein levels measured by Olink Explore 3072 platform[16] resulting from masks similar to the ones we used (aggregating protein truncating and/or rare damaging variants; ptv, raredmg, ptvraredmg)[17] available at the AstraZeneca PheWAS Portal (https://azphewas.com/). For 17 of 73 significant genes detected in our study, plasma levels of the encoded proteins were present and *cis*-associations could be assessed.

Supplementary Results

Sensitivity analyses for gene-based testing: LoF only, SKAT, SKAT-O tests

We performed sensitivity burden test analyses based on high confidence LoF variants only to investigate how the choice of QV selection affected the significant gene-metabolite pairs identified in the main analysis. Whereas effect sizes in the LoF only mask tended to be greater than in the two main masks, association P-values were much less significant (**Extended Data Fig. 1**, **Supplementary Table 4**). Moreover, almost a quarter of associations detected with the two main "HI_mis" and "LoF_mis" masks could not be assessed with the LoF only mask due to missing high confidence LoF variants in the corresponding genes.

In addition, we evaluated the identified gene-metabolite associations using the SKAT and SKAT-O tests (**Supplementary Methods**) to compare power between burden test and alternative approaches in our setting. The P-value provided by the burden test outperformed the one provided by the SKAT test for 369 of 382 associations (**Extended Data Fig. 2**, **Supplementary Table 4**), supporting that burden tests perform better in a setting of assumed loss-of-function as the mechanism underlying metabolic changes. As expected, the SKAT-O performed better than the SKAT test, but nevertheless, burden tests provided lower P-values for 338 of 382 SKAT-O associations (**Supplementary Table 4**).

Stratified analyses to investigate potential subgroup-specific effects

Several stratified analyses were conducted for all significant gene-metabolite associations. Effect sizes across individuals with lower (≤45 ml/min/1.73m^2) and higher (>45 ml/min/1.73m^2) eGFR were strongly correlated (Pearson correlation coefficient 0.97), and none of the gene-metabolite pairs showed significantly different effect sizes across groups (**Extended Data Fig. 3a, Supplementary Table 5**). This supports that the identified gene-metabolite associations were not affected by differences in eGFR.

With regard to sex, effect sizes between men and women were highly correlated as well (Pearson correlation coefficient 0.96), and significant differences in effect sizes were exclusively observed for associations at the X-chromosomal *TMLHE* gene, where men showed more extreme effects on metabolite levels compared to women (**Extended Data Fig. 3b**). These findings are consistent with hemizygosity (and therefore effectively homozygosity) in men compared to heterozygosity in women.

Comparison to previous rare variant studies

We compared our identified gene-metabolite associations to significant findings from previous genetic studies on metabolite levels that focused on the aggregation of rare exonic variants using sequencing and high throughput metabolomics[3–10] (**Supplementary Methods**). Of the 73 identified unique genes, 31 (42.5%) have not been reported as significant in any of these studies. Moreover, 110 of all 192 detected gene-metabolite associations (57.3%) were novel (**Supplementary Table 6**). Seven of these eight previous studies were independent and focused on plasma/serum[3–8,10]. Comparison of the 128 identified gene-plasma metabolite associations to those detected in these independent studies showed that 83 of them (65%) were novel (**Supplementary Methods**, **Supplementary Table 6**). When focusing on studies that employed comparable metabolite quantification technology[3,5,6,10], 69% (88/128) of the associations with plasma metabolites have not been reported, although the underlying metabolite had been analyzed for 95% (122/128) of the identified associations in at least one of the four studies. Hence, 93% (82/88) of these novel plasma associations involved metabolites analyzed before. Among the newly reported genes, two are targets of drugs that are already approved or in development (**Supplementary Table 6**).

Variant characterization of gene-metabolite associations

To characterize the genetic architecture underlying the identified gene-metabolite associations, we initially evaluated the contribution of individual QVs to their gene association signal by performing a forward selection procedure[3] (Methods). The visualization of the association P-value based on the successive aggregation of the most influential QVs (**Supplementary Data 2**) showed notable differences: first, each of the two masks detected

some unique genetic associations, highlighting differences in statistical power to detect associations as well as in genetic architecture. Second, some genes showed different association patterns for the same metabolite in plasma and in urine (e.g., *TMLHE* and hydroxy-N6,N6,N6-trimethyllysine). Third, histidine exemplifies a metabolite with different associated genes in plasma (*HAL*) and urine (*SLC6A19*), implicating an enzyme involved in its hepatic and blood-based breakdown and a transporter responsible for its tubular reabsorption. Fourth, genes associated with the same metabolite in the same matrix can differ in terms of genetic architecture (e.g., urine diacetylspermidine with *PAOX* and *HDAC10*).

Furthermore, we evaluated the convergence of rare and common variant association signals by assessing any common variant in the identified metabolite-associated gene regions (**Supplementary Methods**). We detected significant common variant associations in the regions of 157 of the 235 (192+43) unique gene-metabolite pairs (**Supplementary Table 9**). There was no relation between the absolute aggregated effect size of rare variants with the presence of a GWAS signal in the region (**Extended Data Fig. 5a**). Sensitivity analyses that additionally conditioned the gene-based tests on the associated common variant within a region showed no significant differences in effect sizes compared to the unconditional analysis (Spearman correlation 1.0; **Extended Data Fig. 5b**, **Supplementary Table 9**).

Curation of whole body modeling based on the GCKD data

We performed a range of model curation steps in order to leverage the biological information generated by the WES-metabolite association data from the GCKD study for improving the knowledge base underlying the WBM. These curation steps ranged from adding pathways over improved mapping and checking failing simulations to altering model constraints. The

following paragraphs detail all performed model curations. We performed curations for six virtual IEMs, for which we could identify reasons for model failure (e.g., in the case of *DMGDH*) or for which the GCKD data was instrumental in improving the knowledge base (e.g., in the case of *KYNU* and 8-methoxykynurenate).

Modeling of 8-methoxykynurenate in the virtual IEM for kynureninase deficiency (KYNU)

Although a known human metabolite, the metabolite 8-methoxykynurenate was not included in the initial WBMs due to limited evidence on the enzymes involved in its production. However, in the association results from the GCKD study, urine 8-methoxykynurenate was positively associated with rare, putatively damaging variants in *KYNU.* This indicates that this metabolite originates upstream of a reaction catalyzed by kynureninase. As 8-methoxykynurenate is a methylated derivative of xanthurenate, it is plausibly generated by a corresponding methylation reaction as noted in KEGG (KEGG reaction R03955; Xanthurenic acid + S-adenosyl-L-methionine <=> 8-methoxykynurenate + S-adenosyl-L-homocysteine). Interestingly, we found *ASMTL*, a gene encoding for a protein with presence of a probable catalytic S-adenosyl-L-methionine binding domain in the C-terminal region and thus a probable methyltransferase, to be negatively associated with urine 8-methoxykynurenate (P-value=5.1e-09), which barely missed the study-wide multiple-testing corrected significance threshold. On these grounds, we added 8-methoxykynurenate (C05830) along with the (hypothesized) associated methylation reaction (Xanthurenic acid + S-adenosyl-L-methionine <=> 8-Methoxykynurenate + S-adenosyl-L-homocysteine) and corresponding transport reactions to the ten organs of the male WBM and twelve organs of the female WBM (**Supplementary Table 12**), where the participating metabolites of the methylation reaction were all present. We then repeated the *in silico* knockout of *KYNU*, and successfully replicated

the association of *KYNU* with higher flux of 8-methoxykynurenate into urine compared to the wild-type.

Modeling of N-formylanthranilic acid in the virtual IEM for AFMID

Both N-formylanthranilic acid and the *AFMID* gene were represented in the initial WBM. However, the urinary secretion of N-formylanthranilic acid could not carry flux in the initial simulations. Investigating the model setup for N-formylanthranilic acid, we found that for the transport reaction from the blood compartment to the kidney (WBM reaction name: Kidney_EX_nformanth(e)_[bc]) under the current default constraint setting (lower bound=-3.7368, upper bound=0) any flux of N-formylanthranilic into the kidney compartment was blocked. Consequently, no excretion process into urine could occur. As N-formylanthranilic acid is, however, detected in human urine, we made corresponding adjustments to the constraint setting, allowing N-formylanthranilic acid to be secreted into urine. After this adjustment, the model correctly predicted the observed association between rare, damaging variants in *AFMID* and urine N-formylanthranilic acid levels in the GCKD study. Both the initial and the curated virtual IEM correctly predicted the observed association between rare, damaging *AFMID* variants and plasma N-formylanthranilic acid levels.

Modeling of the virtual IEM for TMLHE

TMLHE encodes the enzyme trimethyllysine dioxygenase, which uses N6,N6,N6-trimethyl-L-lysine as one of its substrates. While *TMHLE* had been included in the initial version of the WBM, none of the metabolites associated with it in the GCKD study could be modeled. We found that in the initial WBM, N6,N6,N6-trimethyl-L-lysine was neither produced from methylated protein-bound lysine residuals, nor was it covered by dietary constraints,

meaning that trimethyllysine dioxygenase reactions could not carry flux. To enable modeling, we unbound the diet constraint for N6,N6,N6-trimethyl-L-lysine[18], making N6,N6,N6-trimethyl-L-lysine available to the WBM. After this step, the virtual IEM for *TMLHE* was perfectly predicting the signs of the observed *TMLHE*-metabolite associations in the GCKD study.

Modeling of dimethylglycine in the virtual IEM for dimethylglycine dehydrogenase deficiency (DMGDH)

Both dimethylglycine and the gene *DMGDH* could be mapped in the initial WBM. However, knockout of *DMGDH* had no effect on dimethylglycine blood and urine secretion fluxes in the female model, and no effect on urine secretion in the male model. Exploring the gene-protein-reaction relations in the initial WBM, we found three reactions assigned to *DMGDH* (mitochondrial dimethylglycine dehydrogenase (VMH ID: DMGDHm), N,N-dimethylglycine:electron-transfer flavoprotein oxidoreductase (VMH ID: HMR_4700), and S-adenosyl-L-methionine:sarcosine N-methyltransferase (VMH ID: HMR_4701)). To the latter two reactions, the gene *PDPR*, encoding for a regulatory subunit of the pyruvate dehydrogenase phosphatase, was assigned as well. We removed the assignment to *PDPR,* as we could not find additional evidence for *PDPR* playing a role in dimethylglycine metabolism besides a distant relation in terms of sequence similarity to *DMGDH*[19]. After removing *PDPR* as a hypothetical isozyme for the reactions HMR_4700 and HMR_4701, the virtual IEM for *DMDGH* correctly predicted the observed effect direction for dimethylglycine both in blood and urine and in both sexes.

Modeling of the virtual IEM for KYAT1

In the initial WBM, we were unable to map the *KYAT1* gene, although it was actually included in the model, due to an identifier discrepancy. We rectified this by adding the corresponding identifier for *KYAT1* in the VMH database (VMH gene identifier: 883), which increased the number of mapped and modeled genes to 26. Three of the metabolites associated with *KYAT1* in the GCKD study, 3-(4-hydroxyphenyl)lactate, indolelactate, and phenylpyruvate, could be mapped in the WBMs and two, 3-(4-hydroxyphenyl)lactate and phenylpyruvate, could be modeled. However, *KYAT1* knockout did not replicate the observed effects from the GCKD study, indicating that further curation of the WBMs is needed in the case of *KYAT1.*

Modeling of hexanoylglycine in the virtual IEM for medium-chain acyl-CoA dehydrogenase deficiency (ACADM)

In the wild-type and knockout *ACADM* models, we initially calculated maximal secretion fluxes for hexanoylglycine into urine. However, the result was consistently a maximum secretion flux of zero for all simulations. Upon exploration, we found that none of the hexanoylglycine-related reactions carried flux in the current WBM. Thus, the metabolite fails the criteria of being transported to blood and urine, and the current WBM is unable to model the *ACADM*-hexanoylglycine gene-metabolite pair. The initial flux calculations of zero were therefore without biological meaning.

Modeling of the virtual IEM for ACY1 and N-acetylisoleucine

We observed the same scenario as in the curation of the virtual IEM of *ACADM* and hexanoylglycine. The initial computation of maximal secretion flux of N-acetylisoleucine into urine consistently yielded zero for all simulations, and none of the related reactions did carry

flux. We were unable to detect the presence of N-acetylisoleucine in human reactions; instead it was exclusively involved in transport reactions across compartments. As a result, the *ACY1*-N-acetylisoleucine pair could not be modeled within the current WBMs.

Personalized WBMs capture observed metabolic changes - PAH

Analogously to the showcase with regard to *KYNU*, where *in silico* WBMs captured changes in urine levels of metabolites in the kynurenine-pathway observed for both heterozygous and homozygous loss of *KYNU* function, we focused on the gene *PAH*. 567 microbiome-personalized[20] WBMs could be successfully generated (Methods) and effect sizes of *in silico* *PAH* knockout on metabolite excretion into urine against the natural variation induced by the personalized microbiomes were calculated (**Supplementary Table 13**). Nine of 272 available metabolites had a modeling P-value <0.05/272, where five of them belong to the phenylalanine metabolism, highlighting their potential role for the corresponding IEM phenylketonuria (**Supplementary Table 14**). Moreover, effect sizes of these 9 metabolites based on *in silico* knockout of *PAH* were significantly correlated with those for *PAH* in the GCKD study, with phenylalanine showing the largest effect in both (**Supplementary Fig. 2b**). As a known biomarker of phenylketonuria, absolute levels of phenylalanine measured in serum samples of a patient with phenylketonuria and her parents (**Supplementary Methods**) were highly elevated in the homozygous patient and in the compound heterozygous father (**Fig. 6b**). This additional showcase serves to corroborate the findings with regard to *KYNU*.

Association of metabolite-associated variants and genes with human traits

Data from ~450,000 UKB participants with WES was queried for associations of the identified 2,077 QVs and 73 genes with thousands of quantitative and binary health outcomes to assess

whether they may be plausibly related to disturbances of the implicated metabolites. The prefiltered UKB dataset contained 696 QVs and 72 genes. At the gene-level, significant associations (P-value<2e-09) were identified between *APOC3* and the binary health outcome "disorders of lipoprotein metabolism and other lipidaemias" (**Supplementary Table 15**), consistent with its association with 19 plasma phosphatidylethanolamine and diacylglycerol metabolites in our study. Moreover, 13 genes showed 282 significant associations with quantitative health outcomes. These mostly arose from clinical chemistry parameters and contained many plausible and well supported examples (**Supplementary Table 15**). At the variant-level, there were 555 significant associations between a QV and a quantitative as well as two additional associations with a binary health outcome (**Supplementary Table 18**). These included well-established examples, but also less studied candidates such as an *SLC6A19* variant encoding the p.Asp173Asn substitution in the sodium-dependent neutral amino acid transporter SLC6A19 (B0AT1), which was associated with lower serum creatinine and cystatin C levels and erythrocyte distribution width.

We have previously shown that the comparison of the effect of common genetic variants (minor allele frequency >0.01) on plasma and urine metabolite levels can deliver specific insights into functions of the kidney[12]. In this study of rare variants of minor allele frequency <0.01, all identified genes that were associated with one or more measures of kidney function (i.e., serum creatinine or cystatin C) in the UKB encode for transport proteins that are highly expressed in the kidney[21–23]: *SLC47A1*, *SLC6A19*, *SLC7A9*, and *SLC22A7* (**Supplementary Table 15**). The gene products of *SLC47A1*, *SLC6A19*, and *SLC7A9* are localized in the apical membrane of tubular cells[21–23]. Their metabolic fingerprints were almost exclusively detected in urine (**Supplementary Table 3**) and reflected the encoded proteins' functions. Conversely, *SLC22A7* encodes for an organic anion transporter in the basolateral

membrane of tubular cells[24], leaving a metabolic signature in plasma. QVs in *SLC47A1* and *SLC22A7* were only associated with creatinine levels but not with cystatin C, in agreement with their known role as creatinine transporters[25]. In contrast, QVs in *SLC7A9* and *SLC6A19* showed association with lower levels of both creatinine and cystatin C[26], suggesting that their loss-of-function is associated with better kidney function through yet unidentified mechanisms. These observations illustrate how rare damaging variants leave a specific signature in plasma and urine metabolomes that mirror exchange processes at the plasma membrane domains of renal epithelial cells and are associated with clinical measures of kidney function, but not with binary kidney disease outcomes after correction for multiple testing (**Supplementary Table 15**). With regard to all metabolite-associated genes and diseases studied in the UKB, similar observations were made. Metabolite-associated genes were not overrepresented among genes associated with binary traits in the UKB, of which many are not expected to be related to altered metabolite levels (odds ratio=1.48, P-value=0.085; **Supplementary Methods**), suggesting that strong genetic effects on metabolite levels do not necessarily translate into genetic effects on diseases.

Supplementary Discussion

Potential limitations of the study

First, discovered gene-metabolite relationships were based on study participants of European ancestry with moderately reduced kidney function, and might therefore not be generalizable. However, rare genetic variants that are predicted or experimentally shown to result in loss-of-function should show effects on associated metabolites regardless of genetic background. Moreover, although metabolite levels may differ between persons with and without reduced

kidney function, our previous work[12,27] and the kidney function-stratified analyses in this study showed comparable genetic effect sizes across different levels of kidney function, including persons with normal kidney function from several population-based cohorts as the UKB. Second, burden tests assume that all aggregated QVs result in direction-consistent effects of similar size, which, if violated, results in a loss of power[28]. Because our study assumed loss-of-function as the mechanism underlying metabolic changes, we did not evaluate alternative aggregate variant tests such as SKAT[29] on an exome-wide basis. SKAT is less powerful in a setting with direction-consistent effects[30], does not provide effect sizes, and is difficult to interpret and replicate[31,32]. Our comparisons to findings from previous studies of the plasma/serum metabolome need to be interpreted in light of such differences in statistical tests, as well as in study design and definition of QVs. Third, inclusion of effectively neutral variants as QVs in a burden test can lead to an underestimation of a gene's effect. Further methodological improvements are required in order to better predict a variant's functional consequence, as well as for optimizing the selection and weighting of QVs to better reflect specific genetic architectures. Fourth, we analyzed non-targeted, semi-quantitative population metabolomics data that do not allow for conclusions whether metabolite levels are outside the clinical reference range. However, non-targeted metabolomics provides much broader coverage than conventional targeted screening within and across biochemical pathways[33], thus enabling the discovery of genetic associations with previously unreported metabolites, as well as the detection of entirely new gene-metabolite relationships as observed here. Lastly, we utilized WBMs for *in silico* validation based on the steady state assumption, whereas it is conceivable that dynamic modeling may improve the predictive power of virtual IEMs. However, such modeling is computationally expensive, and adequate data for fitting dynamic models are often missing. A great advantage of the utilized

constraint-based modeling is its scalability, permitting easy integration with genome-wide genetic screens.

Supplementary Acknowledgements

List of GCKD Study Investigators

A list of nephrologists currently collaborating with the GCKD study is available at http://www.gckd.org.

University of Erlangen-Nürnberg	Kai-Uwe Eckardt, Heike Meiselbach, Markus Schneider, Mario Schiffer, Hans-Ulrich Prokosch, Barbara Bärthlein, Andreas Beck, André Reis, Arif B. Ekici, Susanne Becker, Ulrike Alberth-Schmidt, Anke Weigel, Sabine Marschall, Eugenia Schefler
University of Freiburg	Gerd Walz, Anna Köttgen, Ulla Schultheiß, Fruzsina Kotsis, Simone Meder, Erna Mitsch, Ursula Reinhard
RWTH Aachen University	Jürgen Floege, Turgay Saritas, Alice Groß
Charité, University Medicine Berlin	Elke Schaeffner, Seema Baid-Agrawal, Kerstin Theisen
Hannover Medical School	Hermann Haller
University of Heidelberg	Martin Zeier, Claudia Sommerer Mehtap Aykac
University of Jena	Gunter Wolf, Martin Busch, Rainer Paul
Ludwig-Maximilians University of München	Thomas Sitter
University of Würzburg	Christoph Wanner, Vera Krane, Antje Börner-Klein, Britta Bauer
Medical University of Innsbruck, Division of Genetic Epidemiology	Florian Kronenberg, Julia Raschenberger, Barbara Kollerits, Lukas Forer, Sebastian Schönherr, Hansi Weissensteiner
University of Regensburg, Institute of Functional Genomics	Peter Oefner, Wolfram Gronwald
Department of Medical Biometry, Informatics and Epidemiology (IMBIE), University of Bonn	Matthias Schmid, Jennifer Nadal

Supplementary References

1. Voorman, A., Brody, J., Chen, H., Lumley, T. & Davis, B. seqMeta: Meta-Analysis of Region-Based Tests of Rare DNA Variants. (2017).
2. COHEN, A. Comparing Regression Coefficients Across Subsamples: A Study of the Statistical Test. *Sociol. Methods Res.* **12**, 77–94 (1983).
3. Bomba, L. *et al.* Whole-exome sequencing identifies rare genetic variants associated with human plasma metabolites. *Am. J. Hum. Genet.* **109**, 1038–1054 (2022).
4. König, E. *et al.* Whole Exome Sequencing Enhanced Imputation Identifies 85 Metabolite Associations in the Alpine CHRIS Cohort. *Metabolites* **12**, 604 (2022).
5. Yousri, N. A. *et al.* Whole-exome sequencing identifies common and rare variant metabolic QTLs in a Middle Eastern population. *Nat. Commun.* **9**, 333 (2018).
6. Yu, B. *et al.* Loss-of-function variants influence the human serum metabolome. *Sci. Adv.* **2**, e1600800 (2016).
7. Nag, A. *et al.* Effects of protein-coding variants on blood metabolite measurements and clinical biomarkers in the UK Biobank. *Am. J. Hum. Genet.* **110**, 487–498 (2023).
8. Riveros-Mckay, F. *et al.* The influence of rare variants in circulating metabolic biomarkers. *PLoS Genet.* **16**, e1008605 (2020).
9. Cheng, Y. *et al.* Rare genetic variants affecting urine metabolite levels link population variation to inborn errors of metabolism. *Nat. Commun.* **12**, 964 (2021).
10. Feofanova, E. V. *et al.* Whole-Genome Sequencing Analysis of Human Metabolome in Multi-Ethnic Populations. *Nat. Commun.* **14**, 3111 (2023).
11. Mbatchou, J. *et al.* Computationally efficient whole-genome regression for quantitative and binary traits. *Nat. Genet.* **53**, 1097–1103 (2021).

12. Schlosser, P. *et al.* Genetic studies of paired metabolomes reveal enzymatic and transport processes at the interface of plasma and urine. *Nat. Genet.* **55**, 995–1008 (2023).

13. Noronha, A. *et al.* The Virtual Metabolic Human database: integrating human and gut microbiome metabolism with nutrition and disease. *Nucleic Acids Res.* **47**, D614–D624 (2019).

14. Thiele, I. *et al.* Personalized whole-body models integrate metabolism, physiology, and the gut microbiome. *Mol. Syst. Biol.* **16**, e8982 (2020).

15. Wang, Q. *et al.* Rare variant contribution to human disease in 281,104 UK Biobank exomes. *Nature* **597**, 527–532 (2021).

16. Sun, B. B. *et al.* Plasma proteomic associations with genetics and health in the UK Biobank. *Nature* **622**, 329–338 (2023).

17. Dhindsa, R. S. *et al.* Rare variant associations with plasma protein levels in the UK Biobank. *Nature* **622**, 339–347 (2023).

18. Servillo, L., Giovane, A., Cautela, D., Castaldo, D. & Balestrieri, M. L. Where Does Nε-Trimethyllysine for the Carnitine Biosynthesis in Mammals Come from? *PLoS ONE* **9**, e84589 (2014).

19. Lawson, J. E., Park, S. H., Mattison, A. R., Yan, J. & Reed, L. J. Cloning, expression, and properties of the regulatory subunit of bovine pyruvate dehydrogenase phosphatase. *J. Biol. Chem.* **272**, 31625–31629 (1997).

20. Yachida, S. *et al.* Metagenomic and metabolomic analyses reveal distinct stage-specific phenotypes of the gut microbiota in colorectal cancer. *Nat. Med.* **25**, 968–976 (2019).

21. Otsuka, M. *et al.* A human transporter protein that mediates the final excretion step for toxic organic cations. *Proc. Natl. Acad. Sci. U. S. A.* **102**, 17923–17928 (2005).

22. Kleta, R. *et al.* Mutations in SLC6A19, encoding B0AT1, cause Hartnup disorder. *Nat. Genet.* **36**, 999–1002 (2004).

23. Furriols, M. *et al.* rBAT, related to L-cysteine transport, is localized to the microvilli of proximal straight tubules, and its expression is regulated in kidney by development. *J. Biol. Chem.* **268**, 27060–27068 (1993).

24. Enomoto, A. *et al.* Interaction of human organic anion transporters 2 and 4 with organic anion transport inhibitors. *J. Pharmacol. Exp. Ther.* **301**, 797–802 (2002).

25. Lepist, E.-I. *et al.* Contribution of the organic anion transporter OAT2 to the renal active tubular secretion of creatinine and mechanism for serum creatinine elevations caused by cobicistat. *Kidney Int.* **86**, 350–357 (2014).

26. Sveinbjornsson, G. *et al.* Rare mutations associating with serum creatinine and chronic kidney disease. *Hum. Mol. Genet.* **23**, 6935–6943 (2014).

27. Schlosser, P. *et al.* Genetic studies of urinary metabolites illuminate mechanisms of detoxification and excretion in humans. *Nat. Genet.* **52**, 167–176 (2020).

28. Lee, S., Abecasis, G. R., Boehnke, M. & Lin, X. Rare-variant association analysis: study designs and statistical tests. *Am. J. Hum. Genet.* **95**, 5–23 (2014).

29. Wu, M. C. *et al.* Rare-variant association testing for sequencing data with the sequence kernel association test. *Am. J. Hum. Genet.* **89**, 82–93 (2011).

30. Lee, S. *et al.* Optimal unified approach for rare-variant association testing with application to small-sample case-control whole-exome sequencing studies. *Am. J. Hum. Genet.* **91**, 224–237 (2012).

31. Cirulli, E. T. *et al.* Genome-wide rare variant analysis for thousands of phenotypes in over 70,000 exomes from two cohorts. *Nat. Commun.* **11**, 542 (2020).

32. Long, T. *et al.* Whole-genome sequencing identifies common-to-rare variants associated with human blood metabolites. *Nat. Genet.* **49**, 568–578 (2017).

33. Liu, N. *et al.* Comparison of Untargeted Metabolomic Profiling vs Traditional Metabolic Screening to Identify Inborn Errors of Metabolism. *JAMA Netw. Open* **4**, e2114155 (2021).

Supplementary Figures

Supplementary Figure 1: **Number of significantly associated metabolites by matrix/matrices and biochemical super-pathway.**

Bar plots display the number of metabolites (y-axis) that were determined in plasma or urine only, or in both (x-axis) for each super-pathway. The coloring of each bar indicates which proportion of the measured metabolites was significantly associated in plasma (red) or urine (blue) only, in both (purple), or not significantly associated (light gray).

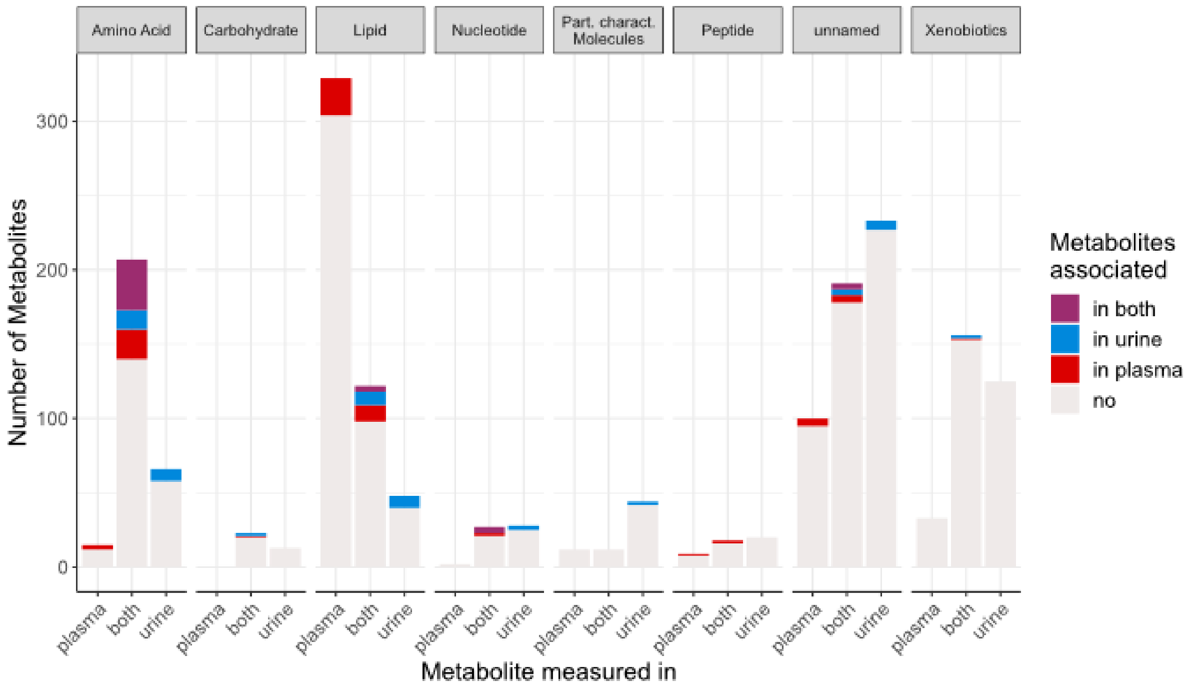

Supplementary Figure 2: Predicted changes upon *in silico* knockout modeling and observed effect sizes based on aggregate variant testing in the GCKD study.

Relation between effect sizes (regression coefficients) upon *in silico* knockout of *KYNU* **(a)** and *PAH* **(b)** (x-axis) and observed effect sizes in the GCKD study (y-axis) for 18 and 9 metabolites that showed significant changes upon *in silico* knockout of *KYNU* (modeling P-value <0.05/257 adjusted for the number of available metabolites) and *PAH* (modeling P-value <0.05/272), respectively. WBM estimates are based on QP-modeling (Methods), and GCKD estimates on aggregating rare, damaging variants in *KYNU and PAH*, respectively. Symbol color represents the sub-pathway of the corresponding metabolite. The gray line is the linear regression line through the data points, the shaded gray area represents its 95% confidence interval. Simulated *in silico* effects of *KYNU* and *PAH* knockouts are clearly correlated with the observed effects in humans given the Pearson correlation coefficients and the corresponding two-sided P-values.

a.

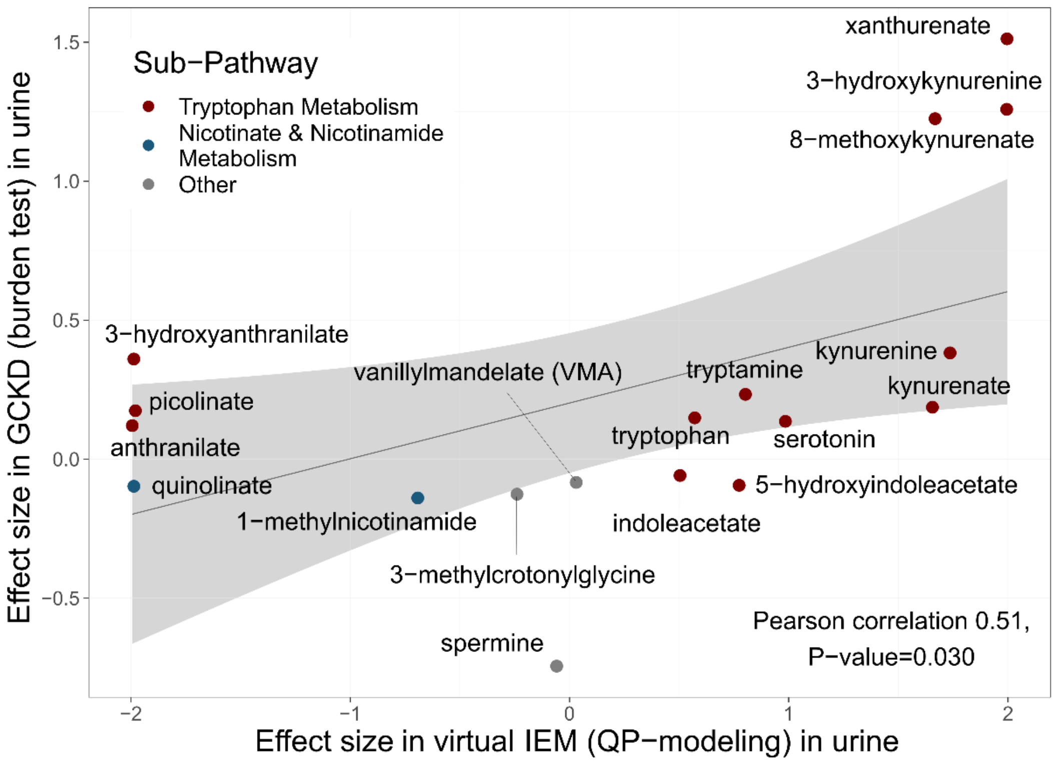

Sub−Pathway
Tryptophan Metabolism
Nicotinate & Nicotinamide Metabolism
Other
xanthurenate
3−hydroxykynurenine
8−methoxykynurenate
3−hydroxyanthranilate
picolinate
anthranilate
quinolinate
vanillylmandelate (VMA)
1−methylnicotinamide
3−methylcrotonylglycine
spermine
tryptamine
kynurenine
kynurenate
tryptophan
serotonin
5−hydroxyindoleacetate
indoleacetate
Pearson correlation 0.51,
P−value=0.030
Effect size in GCKD (burden test) in urine
Effect size in virtual IEM (QP−modeling) in urine
1.5
1.0
0.5
0.0
−0.5
−2
−1
0
1
2

b.

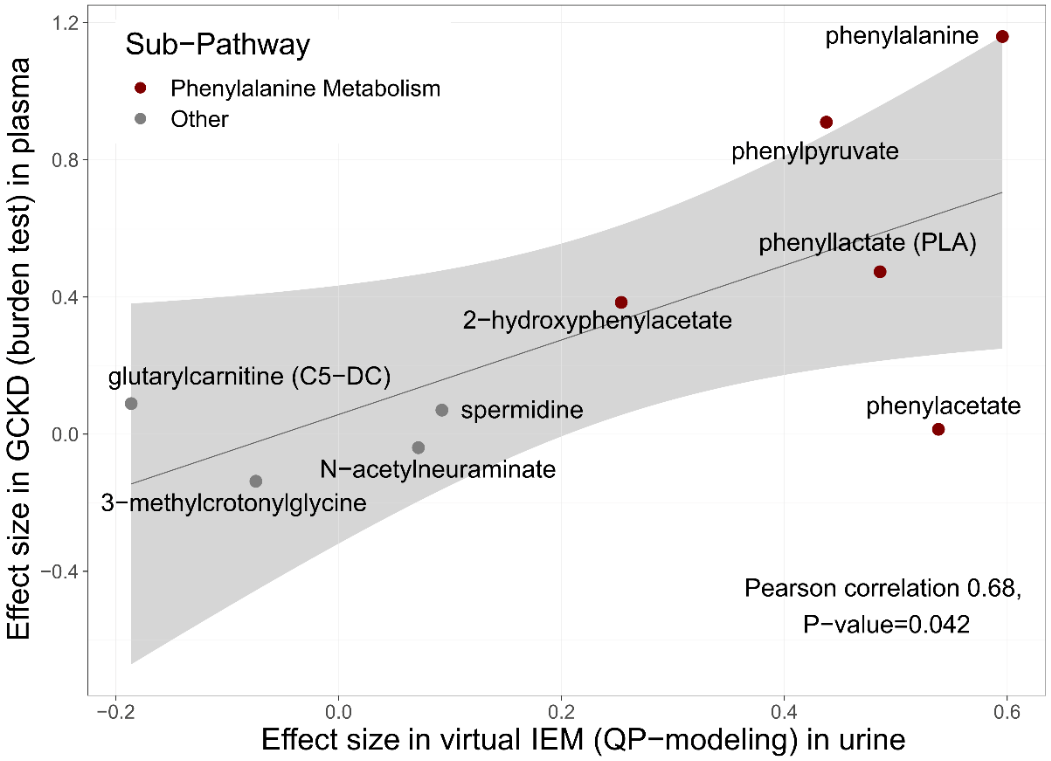

Sub−Pathway
Phenylalanine Metabolism
Other
phenylalanine
phenylpyruvate
phenyllactate (PLA)
2−hydroxyphenylacetate
glutarylcarnitine (C5−DC)
spermidine
N−acetylneuraminate
3−methylcrotonylglycine
phenylacetate
Pearson correlation 0.68,
P−value=0.042
Effect size in GCKD (burden test) in plasma
Effect size in virtual IEM (QP−modeling) in urine
1.2
0.8
0.4
0.0
−0.4
−0.2
0.0
0.2
0.4
0.6

Supplementary Figure 3: Contribution of individual QVs in *SLC13A1* and *SLC26A1* to their gene-based association signal with height measured in the GCKD study.

The symbols visualize the -$\log_{10}$(P-value) (y-axis) with regard to height measured in 3,239 participants of the GCKD study for the successive aggregation of the most influential QVs in *SLC13A1* (mask HI_mis) **(a)** and *SLC26A1* (mask LoF_mis) **(b)** with respect to the forward selection procedure (Bomba *et al.*, PMID: 35568032, Methods) based on burden tests. The number of QVs aggregated for burden testing is shown on the x-axis. Symbol shape indicates the variant's consequence. The symbol color and size reflect the effect size and the P-value of the variant based on its single-variant association test. The gray dashed lines represent the significance threshold (-$\log_{10}$(0.05)), the total -$\log_{10}$(P-value) of the aggregate variant test including all QVs in *SLC13A1* and *SLC26A1* for the respective mask, and the -$\log_{10}$(lowest P-value) that can be reached by aggregating only the driver variants from the forward selection procedure. For both genes, a clear association with height in the GCKD study is observed when aggregating driver variants.

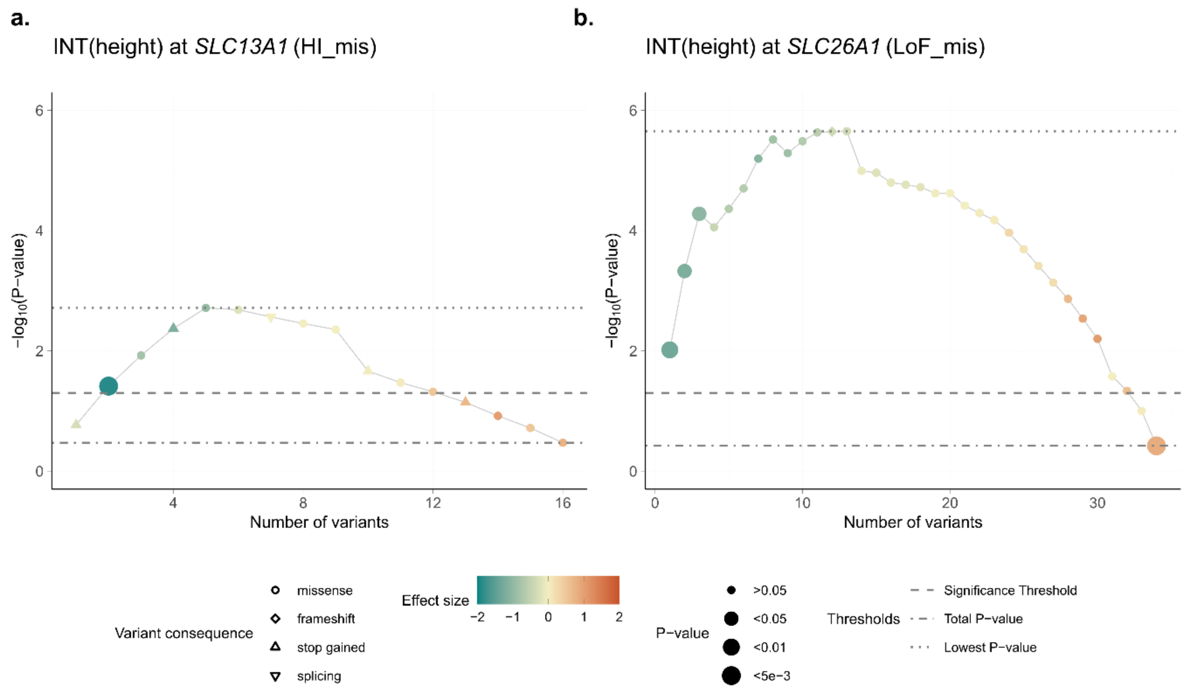

Supplementary Data 1: Plasma and urine metabolite levels among carriers and non-carriers of QVs in significantly associated genes.
Metabolite levels after inverse normal transformation and covariate-adjustment are shown on the y-axis, among non-carriers and carriers of QVs in both masks (LoF_mis and HI_mis) on the x-axis for all significant gene-metabolite associations based on up to 4,713 individuals (**Supplementary Table 3**). Associations in plasma are shown on the left, in urine on the right. Plots are sorted alphabetically by gene name. Symbol color and shape indicate a variant's driver status and consequence, respectively. Carriers of multiple heterozygous QVs are denoted by an asterisk. Orange filling of symbols denotes homozygosity for the respective QV for autosomal genes, and hemizygosity for X-chromosomal genes. The boxes range from the 25th to the 75th percentile of metabolite levels, the median is indicated by a line, and whiskers end at the last observed value within 1.5*(interquartile range) away from the box.

Due to size limits, these plots are included as a separate file.

Supplementary Data 2: Contribution of individual QVs to their gene-based association signal with plasma and urine metabolite levels.
For each significant gene-metabolite pair in plasma and/or in urine (sorted by gene and metabolite's biochemical name), the symbols visualize the $-\log_{10}$(P-value) (y-axis) for the successive aggregation of the most influential QVs with respect to the forward selection procedure (Bomba et al. 2022, PMID: 35568032, Methods) based on burden tests for both masks (LoF_mis on the left, HI_mis on the right). The number of QVs aggregated for burden testing is given on the x-axis. Symbol shape indicates the variant's consequence. The symbol color and size reflect the effect size and the P-value of the variant based on its single-variant association test. The gray dashed lines represent the significance threshold ($-\log_{10}$(5.04e-9) for plasma and $-\log_{10}$(4.46e-9) for urine), the total $-\log_{10}$(P-value) of the aggregate variant test including all QVs in the respective gene and mask, and the $-\log_{10}$(lowest P-value) that can be reached by aggregating only the driver variants from the forward selection procedure. Hence, the variants sorted on the left, which provide the lowest P-value when aggregated, represent the driver variants (for further variant annotation see **Supplementary Tables 7a**,**b**).

Due to size limits, these plots are included as a separate file.
