# Supplementary material for: Coupling metabolomics and exome sequencing reveals graded effects of rare damaging heterozygous variants on gene function and human traits
Source: Nat Genet. 2025 Jan 2;57(1):193–205. doi: 10.1038/s41588-024-01965-7 (PMC11735408; doi:10.1038/s41588-024-01965-7)
Supplement: Supplementary file 2 — Reporting Summary [file 41588_2024_1965_MOESM2_ESM.pdf]

nature portfolio

Corresponding author(s): Anna Köttgen, Johannes Hertel

Last updated by author(s): Sep 27, 2024

Reporting Summary

Nature Portfolio wishes to improve the reproducibility of the work that we publish. This form provides structure for consistency and transparency in reporting. For further information on Nature Portfolio policies, see our Editorial Policies and the Editorial Policy Checklist.

Statistics

For all statistical analyses, confirm that the following items are present in the figure legend, table legend, main text, or Methods section.

n/a	Confirmed	
☐	☒	The exact sample size (*n*) for each experimental group/condition, given as a discrete number and unit of measurement
☐	☒	A statement on whether measurements were taken from distinct samples or whether the same sample was measured repeatedly
☐	☒	The statistical test(s) used AND whether they are one- or two-sided<br>*Only common tests should be described solely by name; describe more complex techniques in the Methods section.*
☐	☒	A description of all covariates tested
☐	☒	A description of any assumptions or corrections, such as tests of normality and adjustment for multiple comparisons
☐	☒	A full description of the statistical parameters including central tendency (e.g. means) or other basic estimates (e.g. regression coefficient) AND variation (e.g. standard deviation) or associated estimates of uncertainty (e.g. confidence intervals)
☐	☒	For null hypothesis testing, the test statistic (e.g. *F*, *t*, *r*) with confidence intervals, effect sizes, degrees of freedom and *P* value noted<br>*Give P values as exact values whenever suitable.*
☒	☐	For Bayesian analysis, information on the choice of priors and Markov chain Monte Carlo settings
☒	☐	For hierarchical and complex designs, identification of the appropriate level for tests and full reporting of outcomes
☐	☒	Estimates of effect sizes (e.g. Cohen's *d*, Pearson's *r*), indicating how they were calculated

Our web collection on statistics for biologists contains articles on many of the points above.

Software and code

Policy information about availability of computer code

Data collection	The data for the GCKD study was collected using the software Askimed (https://www.askimed.com/).
Data analysis	- Software-tools for processing of whole-exome sequencing data: Illumina DRAGEN Bio-IT Platform Germline Pipeline v3.0.7 at Astra Zeneca's Centre for Genomics Research<br>Software-tools for QC of whole-exome sequencing data: KING --kinship v2.2.3<br>- Software-tools for variant and gene annotation: Variant Effect Predictor (VEP) v101 with plugins REVEL v2020-5, CADD v3.0, LoFtee v2020-8, dbNSFP v4.1a, gnomAD v 2.1<br>- Software-tools for rare variant aggregation testing in the GCKD study: seqMeta R-package v1.6.7<br>- Software-tools for GWAS of metabolites to compare with rare variant results: REGENIE v2.2.4<br>- Software-tools for Firth regression: R-package "brglm2"<br>- Software-tools for gene-based testing in the UKB: REGENIE v3.3<br>- Software-tools for in silico whole-body modeling: COBRA Toolbox v3.4 (https://opencobra.github.io/cobratoolbox/stable/index.html), Matlab 2019b and 2021a, Ilog Cplex v12.9 and v12.10, physiologically and stoichiometrically constrained modeling (PSCM) toolbox (https://github.com/opencobra/cobratoolbox/tree/master/src/analysis/wholeBody/PSCMToolbox), R-package "plm" for regression of panel data.<br>- Source codes for personalized whole-body modelling: https://github.com/SysPsyHertel/CodeBase/tree/main/Scripts_Scherer_WBM<br>- Data bases, publicly available: Ensembl VEP tool, GTEx Project, Ensembl Biomart, AstraZeneca PheWAS Portal, OMIM catalog, Genomics England PanelApp v4.0, Open Targets Platform, ClinVar archive, Virtual Metabolic Human database<br>- Miscellaneous: R v3.6.3 and v4.0.5<br>References or website addresses are provided in the manuscript.

For manuscripts utilizing custom algorithms or software that are central to the research but not yet described in published literature, software must be made available to editors and reviewers. We strongly encourage code deposition in a community repository (e.g. GitHub). See the Nature Portfolio guidelines for submitting code & software for further information.

Data

Policy information about availability of data

All manuscripts must include a data availability statement. This statement should provide the following information, where applicable:

- Accession codes, unique identifiers, or web links for publicly available datasets
- A description of any restrictions on data availability
- For clinical datasets or third party data, please ensure that the statement adheres to our policy

Data preparation, quality control, data modeling and statistical analyses of the data presented in this manuscript were performed at the Institute of Genetic Epidemiology, Medical Center - University of Freiburg, Freiburg (Germany) and at the Department of Psychiatry and Psychotherapy, University Medicine Greifswald, Greifswald (Germany), unless otherwise mentioned in the Methods.
The summary statistics of all significant gene-metabolite associations based on burden tests using two masks as well as all involved QVs with annotations are available in Supplementary Table 3 and Supplementary Tables 7a, b, respectively. Genotype, metabolite, protein and phenotype data were obtained from the UKB (https://www.ukbiobank.ac.uk/) and the GCKD study (https://www.gckd.org/). This research has been conducted using the UK Biobank Resource under Application Number 64806.
The following external data sources were used: GRCh38 reference genome (https://ftp.ncbi.nlm.nih.gov/genomes/all/GCA/000/001/405/GCA_000001405.15_GRCh38/): alignment of reads; GTEx Project (https://gtexportal.org/home/): investigation of gene expression and QTLs across tissues; AstraZeneca PheWAS Portal (https://azphewas.com/): search for gene- and variant-level associations of detected genes and QVs; OMIM catalog (https://www.omim.org/): query for monogenic disorders and traits related to identified genes; Genomics England PanelApp (https://panelapp.genomicsengland.co.uk/panels/467/ version v4.0): search for known IEM related to the detected genes; Open Targets Platform (https://platform.opentargets.org/): search for drug target status and corresponding indication for identified genes; ClinVar archive (https://www.ncbi.nlm.nih.gov/clinvar/): query for clinical significance and corresponding trait/disease of detected QVs. Microbiome abundance data (https://static-content.springer.com/esm/art%3A10.1038%2Fs41591-019-0458-7/MediaObjects/41591_2019_458_MOESM3_ESM.xlsx) and the AGORA resource of genome-scale microbial reconstructions (https://github.com/VirtualMetabolicHuman/AGORA/): Creating in silico microbiome models; Organ-resolved, sex-specific whole-body metabolic reconstructions, Harvey_1_04b and Harvetta_1_04c, which are updated versions of the current public models v1_03c (https://www.digitalmetabolictwin.org/copy-of-reconstructions): Creating (personalized) whole-body models; Virtual Metabolic Human database (https://vmh.life/): identifying reactions carried out by corresponding genes.

Human research participants

Policy information about studies involving human research participants and Sex and Gender in Research.

Reporting on sex and gender

Persons of both sexes were included in all analyses. In the context of this study, biological sex was used. X chromosomal genetic variants were included in all analyses.

Population characteristics

Please see Supplementary Table 1:
Characteristics of the GCKD study overall: N=4,737
Mean age (SD), years: 60.26 (11.88)
Female sex, % (n): 39.75% (1883)
Mean BMI (SD), kg/m²: 29.8 (5.97)
Mean systolic blood pressure (SD), mm Hg: 139.57 (20.38)
Mean Hemoglobin A1c (SD), mmol/mol: 45.82 (11.29)
Diabetes, % (n): 35.61% (1687)
Mean eGFR (SD), ml/min/1.73m²: 49.42 (18.21)
Median urinary albumin-to-creatinine ratio (IQR), mg/g: 49.28 (9.3-375.78)
Mean albumin (SD), g/l: 38.33 (4.25)

Recruitment

The GCKD study is an ongoing prospective observational cohort study of participants with CKD. Between 2010 and 2012, 5,217 adult persons with CKD under regular care by nephrologists provided written informed consent and were enrolled into the study at nine participating study centers across Germany (see below). Participants were included if they met inclusion criteria, but - as in all epidemiological studies - it cannot be excluded that eligible persons with many or severe comorbidities were less likely to participate than other eligible participants. For this project, all participants with available plasma or urine collected at the baseline visit and with available whole-exome sequencing data were selected (N=4,737).

Ethics oversight

The GCKD Study was registered in the national registry for clinical studies (DRKS 00003971) and approved by all local ethic committees of the nine participating centers (Universities or Medical Faculties of Aachen, Berlin, Erlangen, Freiburg, Hannover, Heidelberg, Jena, München, Würzburg).

Note that full information on the approval of the study protocol must also be provided in the manuscript.

Field-specific reporting

Please select the one below that is the best fit for your research. If you are not sure, read the appropriate sections before making your selection.

☒ Life sciences ☐ Behavioural & social sciences ☐ Ecological, evolutionary & environmental sciences

For a reference copy of the document with all sections, see nature.com/documents/nr-reporting-summary-flat.pdf

Life sciences study design

All studies must disclose on these points even when the disclosure is negative.

Sample size	We included all 4,737 GCKD participants with available plasma or urine metabolite quantification and with available whole-exome sequencing data. The entire sample was therefore utilized, and no selection was made.
Data exclusions	Metabolites were excluded for high proportions of missingness (>93%). Samples were excluded if no high-quality whole-exome sequencing data was available. This is clearly described in the methods.
Replication	Replication of gene-metabolite associations based on aggregating rare damaging variants is difficult because of the non-availability of the same rare damaging variant in an independent cohort.<br>Therefore, we evaluated reproducibility of our findings by different means :<br>- We compared our identified gene-metabolite associations to those from 8 published studies that focused on rare variant aggregation testing of metabolite levels. For one published study of the plasma metabolome (based on MS-quantification) that made summary statistics accessible we compared effect sizes on the variant- and gene-level.<br>- For overlapping metabolites, we conducted gene-based tests in the UKB using the same variants and tests as in our study.<br>- We investigated the role of our metabolite-associated genes in currently known inborn errors of metabolism (IEM).<br>- We used whole-body models where in silico gene knockouts were modeled to validate our identified gene-metabolite pairs.<br>- We performed a proof-of-concept experimental validation study for an implicated metabolite not yet shown to be involved in the encoded protein's function.<br>When information was available, each of these approaches supported the validity of findings.
Randomization	Not relevant to this study because this is an observational study.
Blinding	Not relevant to this study because this is an observational study.

Reporting for specific materials, systems and methods

We require information from authors about some types of materials, experimental systems and methods used in many studies. Here, indicate whether each material, system or method listed is relevant to your study. If you are not sure if a list item applies to your research, read the appropriate section before selecting a response.

Materials & experimental systems

n/a	Involved in the study	
☒	☐	Antibodies
☐	☒	Eukaryotic cell lines
☒	☐	Palaeontology and archaeology
☒	☐	Animals and other organisms
☐	☒	Clinical data
☒	☐	Dual use research of concern

Methods

n/a	Involved in the study	
☒	☐	ChIP-seq
☒	☐	Flow cytometry
☒	☐	MRI-based neuroimaging

Eukaryotic cell lines

Policy information about cell lines and Sex and Gender in Research

Cell line source(s)	Chinese Hamster Ovary (CHO) cells with Tetracycline-Regulated Expression (T-REx) system. Cell line obtained from and engineered at Axxam.
Authentication	Cells were not authenticated.
Mycoplasma contamination	Cell lines previously tested negative for mycoplasma. Cells in this experiment were not tested for mycoplasma.
Commonly misidentified lines<br>(See ICLAC register)	No commonly misidentified cell lines were used.

Clinical data

Policy information about clinical studies

All manuscripts should comply with the ICMJE guidelines for publication of clinical research and a completed CONSORT checklist must be included with all submissions.

Clinical trial registration	This study is an observational study (DRKS 00003971).
Study protocol	The study protocol and design has been published (PMID: 21862458).

Data collection	Between 2010 and 2012, 5,217 adult persons with CKD under regular care by nephrologists provided written informed consent and were enrolled into the study at nine participating study centers across Germany (Aachen, Berlin, Erlangen, Freiburg, Hannover, Heidelberg, Jena, München, Würzburg).<br>Data was collected during GCKD study visits by trained personnel in any of the nine study centers following a published pre-specified protocol and standard operating procedures, and captured with the software Askimed (https://www.askimed.com/).<br>For this project, all participants with available plasma or urine collected at the baseline visit and with available whole-exome sequencing data were selected (N=4,737).<br>The participants are currently followed for clinical outcomes for more than 10 years.
Outcomes	The predefined outcomes of this study were metabolite levels in plasma and urine, which was defined before study initiation by the authors. Non-targeted MS analysis was performed at Metabolon, Inc., from plasma and urine samples collected at the study's baseline visit, as described in detail in the publication.
