# Supplementary material for: Coupling metabolomics and exome sequencing reveals graded effects of rare damaging heterozygous variants on gene function and human traits
Source: Nat Genet. 2025 Jan 2;57(1):193–205. doi: 10.1038/s41588-024-01965-7 (PMC11735408; doi:10.1038/s41588-024-01965-7)
Supplement: Supplementary file 5 — Plasma and urine metabolite levels among carriers and noncarriers of QVs in significantly associated genes. [file 41588_2024_1965_MOESM5_ESM.pdf]

Supplementary Data 1: Plasma and urine metabolite levels among carriers and non-carriers of QVs in significantly associated genes.
Metabolite levels after inverse normal transformation and covariate-adjustment are shown on the y-axis, among non-carriers and carriers of QVs in both masks (LoF_mis and HI_mis) on the x-axis for all significant gene-metabolite associations based on up to 4,713 individuals (**Supplementary Table 3**). Associations in plasma are shown on the left, in urine on the right. Plots are sorted alphabetically by gene name. Symbol color and shape indicate a variant's driver status and consequence, respectively. Carriers of multiple heterozygous QVs are denoted by an asterisk. Orange filling of symbols denotes homozygosity for the respective QV for autosomal genes, and hemizygosity for X-chromosomal genes. The boxes range from the 25th to the 75th percentile of metabolite levels, the median is indicated by a line, and whiskers end at the last observed value within 1.5*(interquartile range) away from the box.

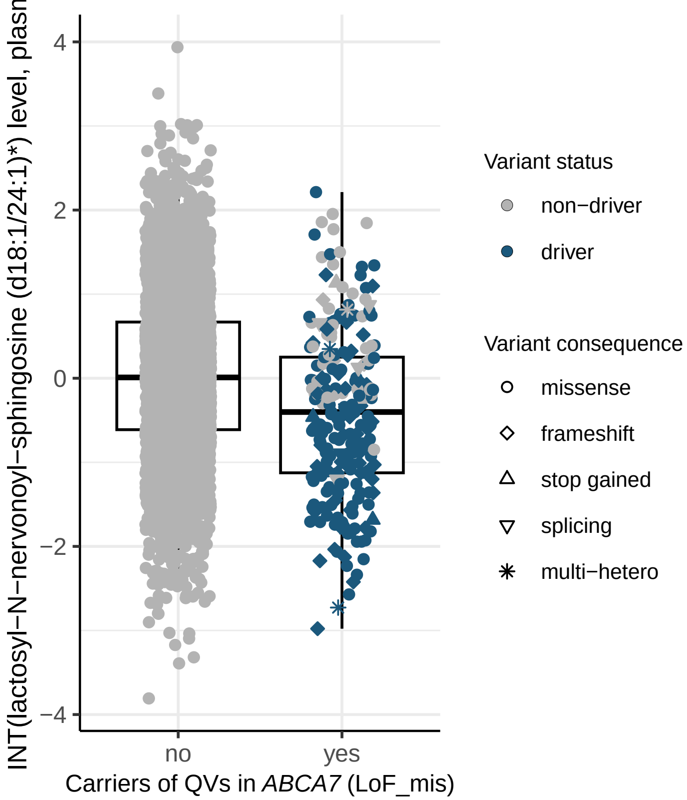

INT(lactosyl−N−nervonoyl−sphingosine (d18:1/24:1)*) level, plasm
4
2
0
−2
−4
no
yes
Carriers of QVs in *ABCA7* (LoF_mis)
Variant status
non−driver
driver
Variant consequence
missense
frameshift
stop gained
splicing
multi−hetero

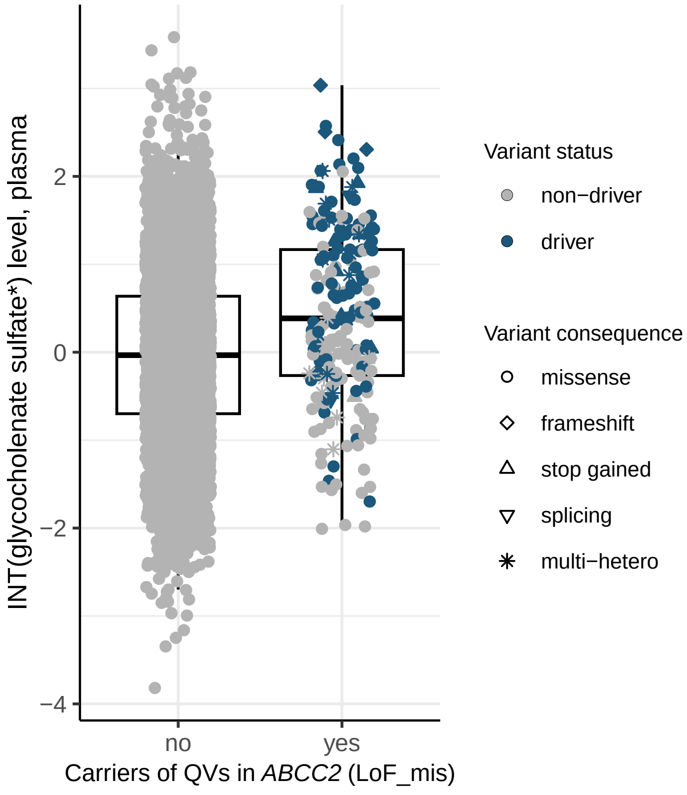

INT(glycocholenate sulfate*) level, plasma
2
0
−2
−4
no
yes
Carriers of QVs in *ABCC2* (LoF_mis)
Variant status
non−driver
driver
Variant consequence
missense
frameshift
stop gained
splicing
multi−hetero

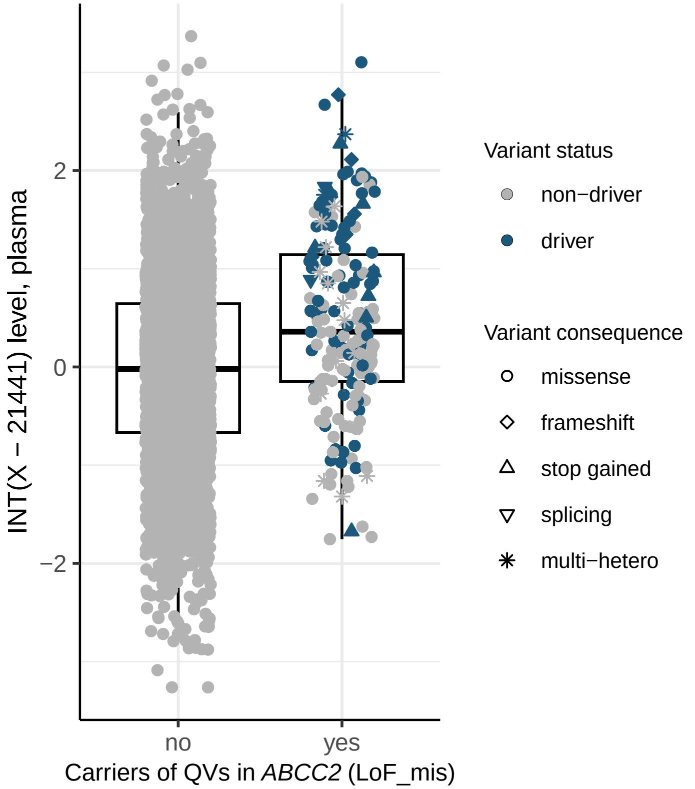
INT(X − 21441) level, plasma
2
0
−2
no
yes
Carriers of QVs in *ABCC2* (LoF_mis)
Variant status
non−driver
driver
Variant consequence
missense
frameshift
stop gained
splicing
multi−hetero

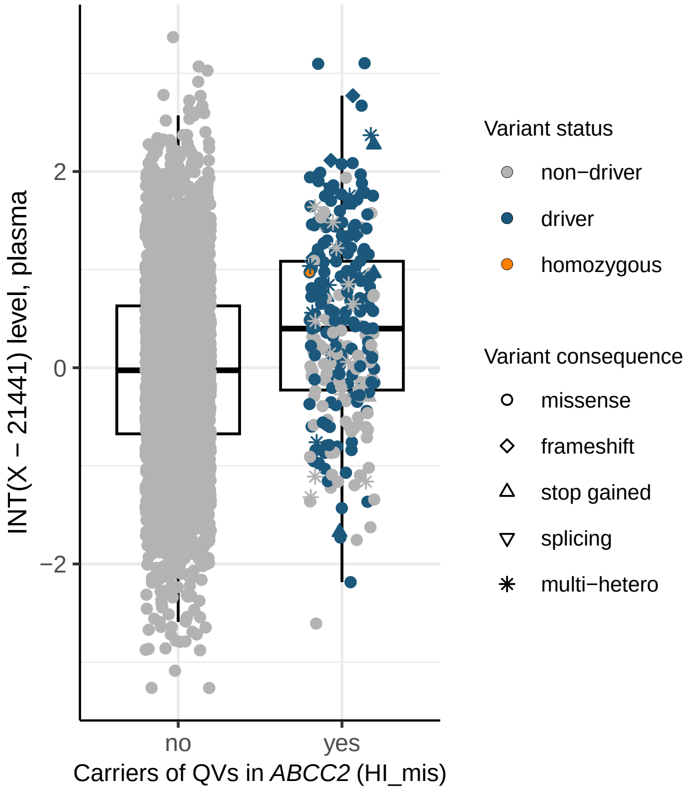
INT(X − 21441) level, plasma
2
0
−2
no
yes
Carriers of QVs in *ABCC2* (HI_mis)
Variant status
non−driver
driver
homozygous
Variant consequence
missense
frameshift
stop gained
splicing
multi−hetero

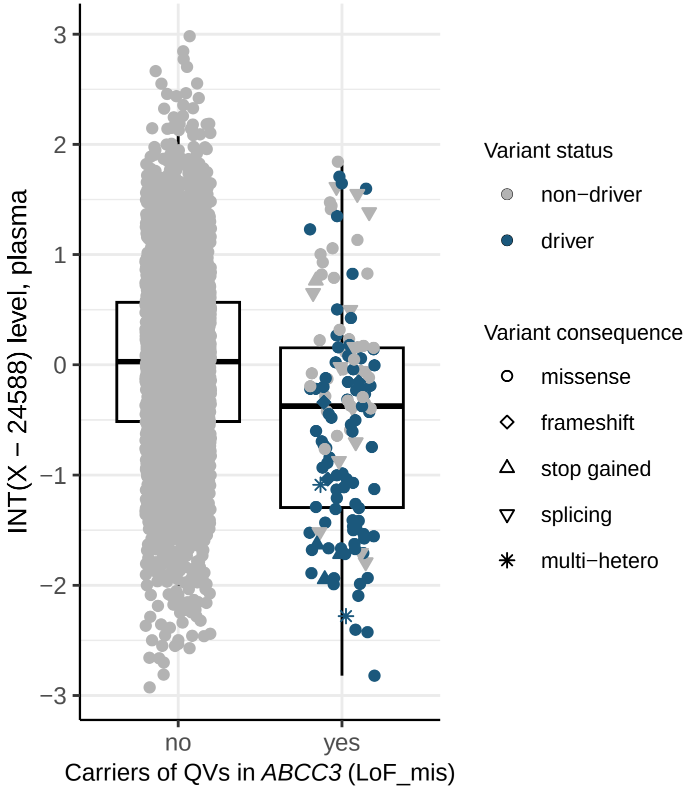

3
2
1
0
−1
−2
−3
INT(X − 24588) level, plasma
no
yes
Carriers of QVs in ABCC3 (LoF_mis)
Variant status
non−driver
driver
Variant consequence
missense
frameshift
stop gained
splicing
multi−hetero

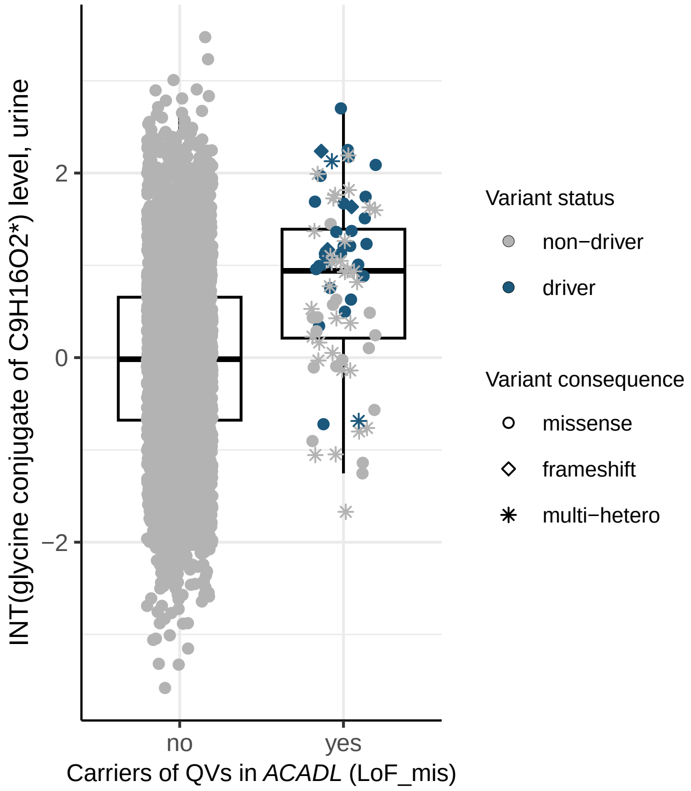

2
0
−2
INT(glycine conjugate of C9H16O2*) level, urine
no
yes
Carriers of QVs in ACADL (LoF_mis)
Variant status
non−driver
driver
Variant consequence
missense
frameshift
multi−hetero

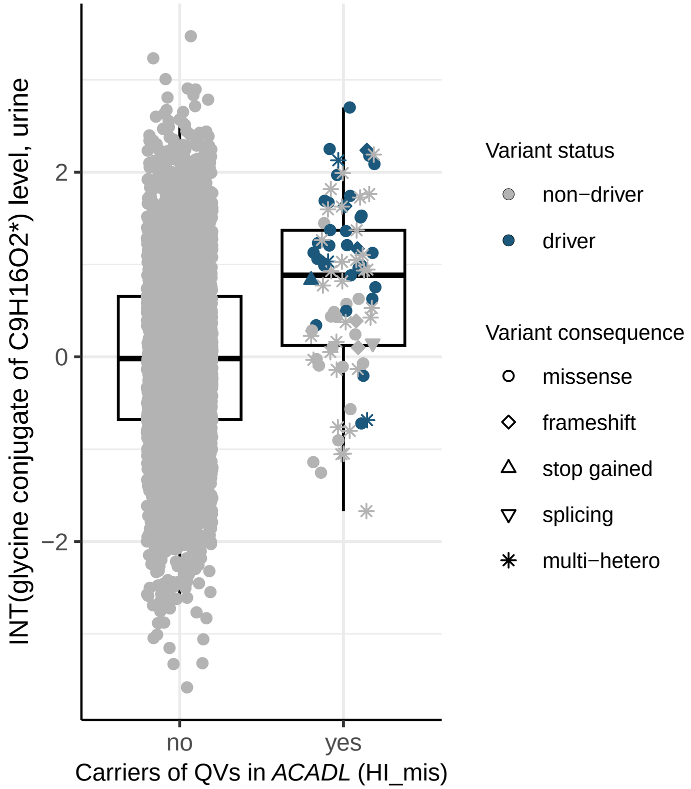

INT(glycine conjugate of C9H16O2*) level, urine
2
0
−2
no
yes
Carriers of QVs in *ACADL* (HI_mis)
Variant status
non−driver
driver
Variant consequence
missense
frameshift
stop gained
splicing
multi−hetero

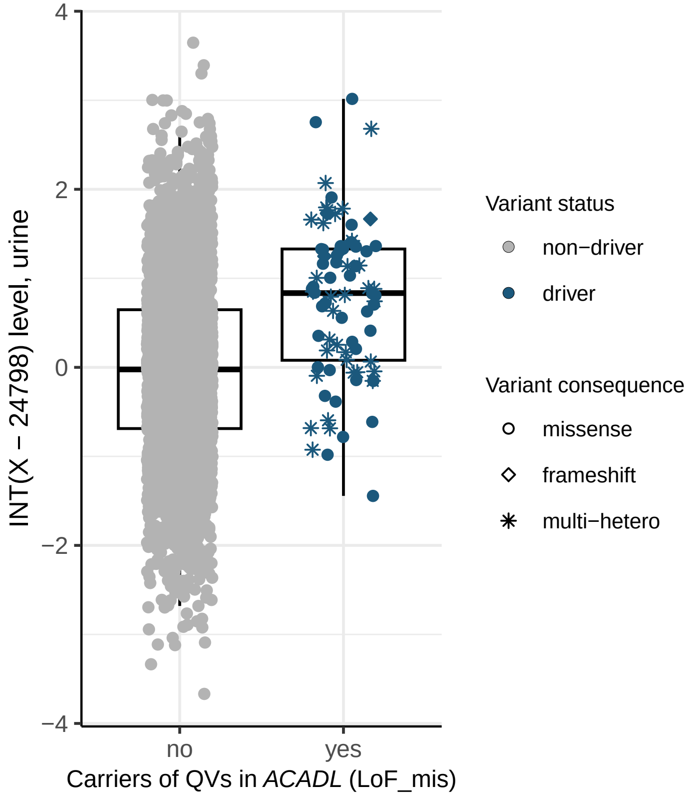

INT(X − 24798) level, urine
4
2
0
−2
−4
no
yes
Carriers of QVs in *ACADL* (LoF_mis)
Variant status
non−driver
driver
Variant consequence
missense
frameshift
multi−hetero

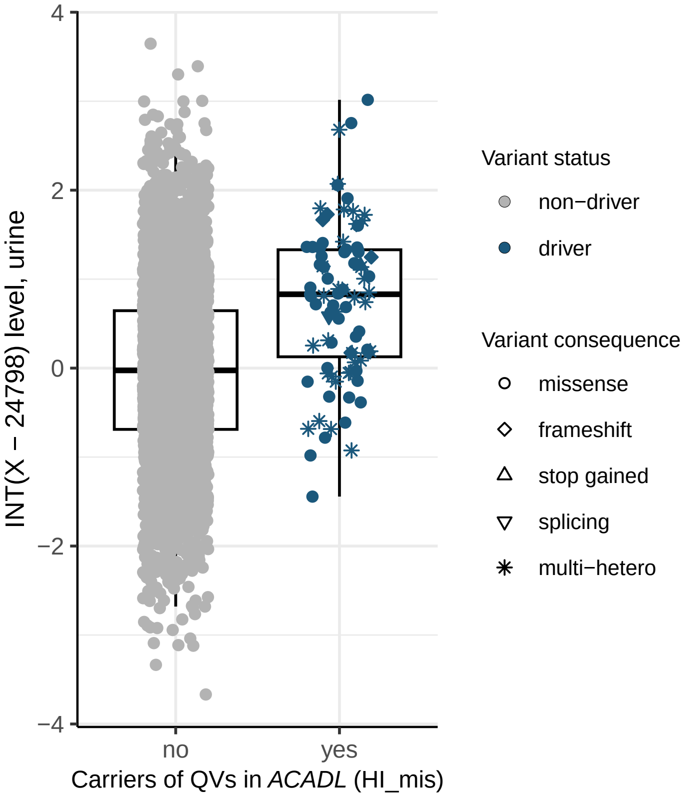
INT(X − 24798) level, urine
4
2
0
−2
−4
no
yes
Carriers of QVs in ACADL (HI_mis)
Variant status
non−driver
driver
Variant consequence
missense
frameshift
stop gained
splicing
multi−hetero

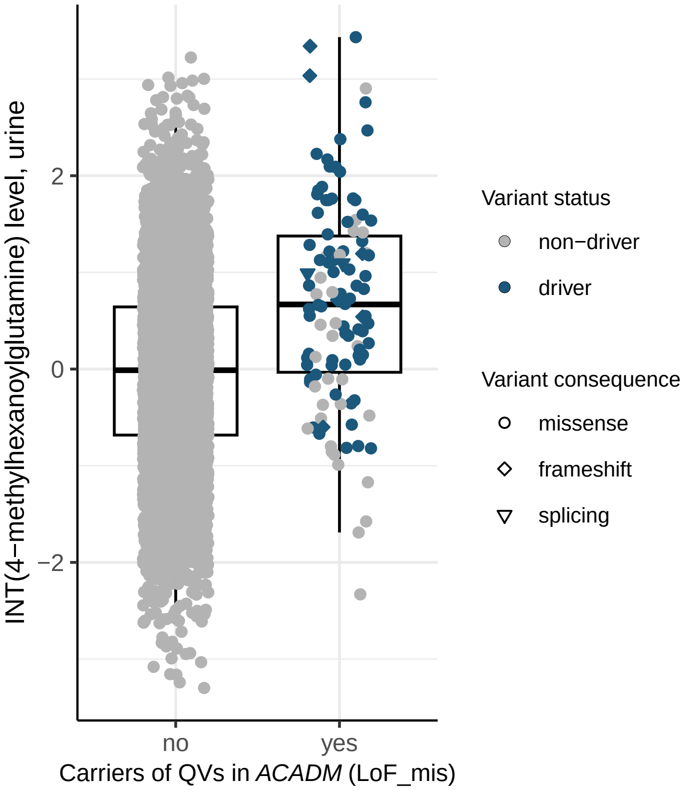
INT(4−methylhexanoylglutamine) level, urine
2
0
−2
no
yes
Carriers of QVs in ACADM (LoF_mis)
Variant status
non−driver
driver
Variant consequence
missense
frameshift
splicing

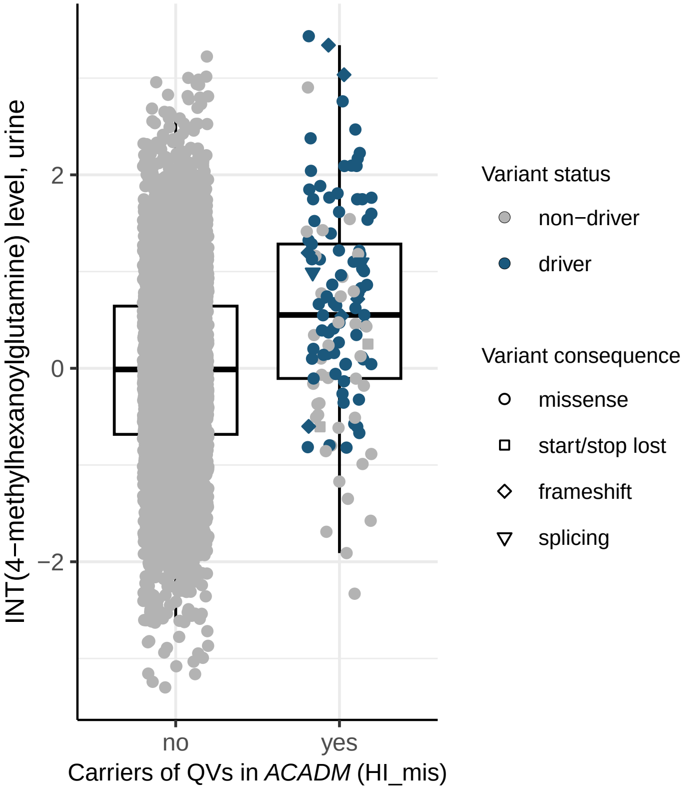
INT(4−methylhexanoylglutamine) level, urine
2
0
−2
no
yes
Carriers of QVs in *ACADM* (HI_mis)
Variant status
non−driver
driver
Variant consequence
missense
start/stop lost
frameshift
splicing

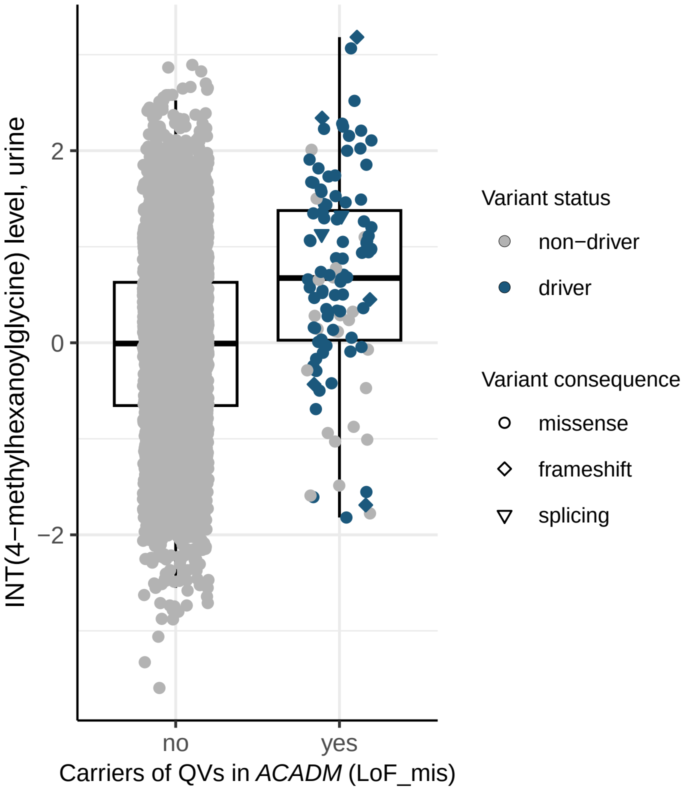
INT(4−methylhexanoylglycine) level, urine
2
0
−2
no
yes
Carriers of QVs in *ACADM* (LoF_mis)
Variant status
non−driver
driver
Variant consequence
missense
frameshift
splicing

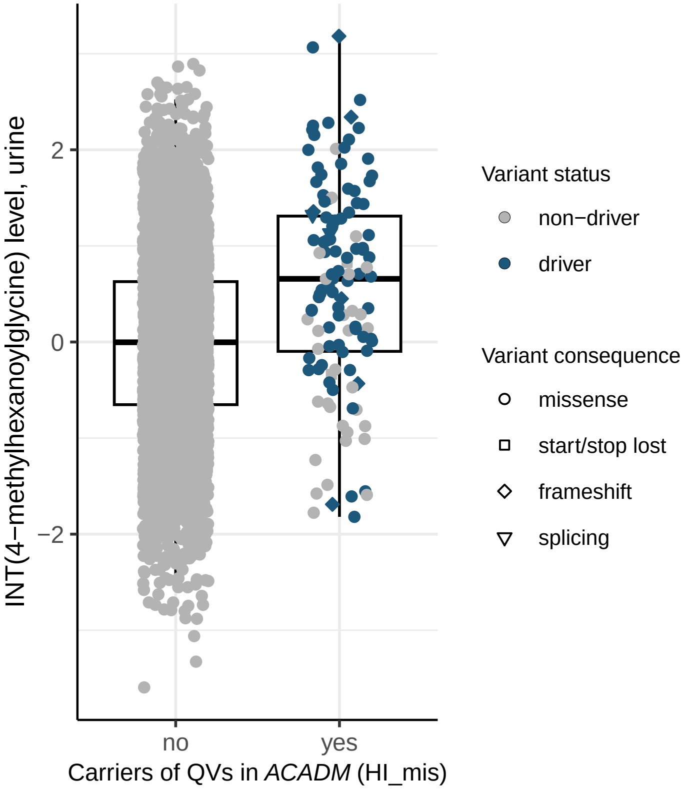
INT(4−methylhexanoylglycine) level, urine
2
0
−2
no
yes
Carriers of QVs in *ACADM* (HI_mis)
Variant status
non−driver
driver
Variant consequence
missense
start/stop lost
frameshift
splicing

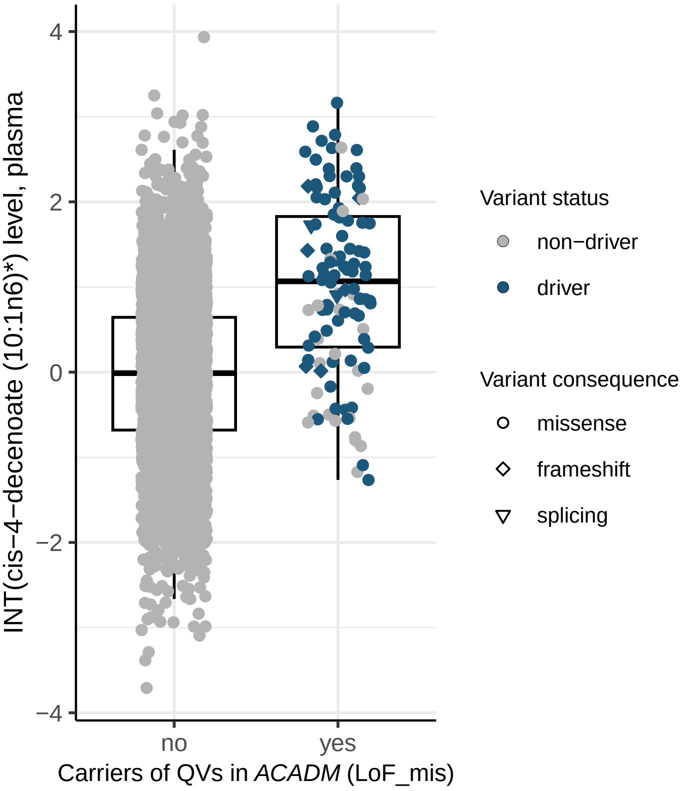
INT(cis−4−decenoate (10:1n6)*) level, plasma
4
2
0
−2
−4
no
yes
Carriers of QVs in *ACADM* (LoF_mis)
Variant status
non−driver
driver
Variant consequence
missense
frameshift
splicing

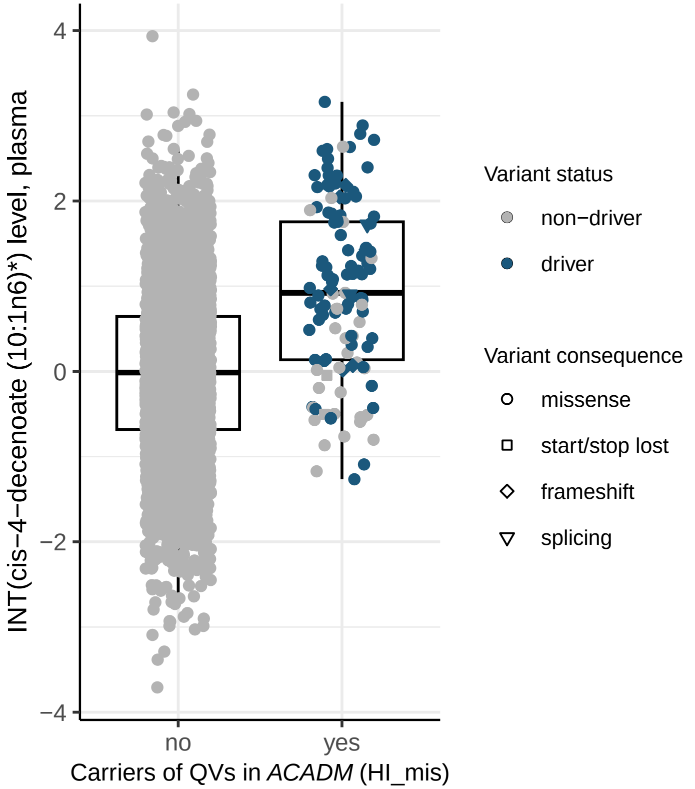

INT(cis−4−decenoate (10:1n6)*) level, plasma
4
2
0
−2
−4
no
yes
Carriers of QVs in ACADM (HI_mis)
Variant status
non−driver
driver
Variant consequence
missense
start/stop lost
frameshift
splicing

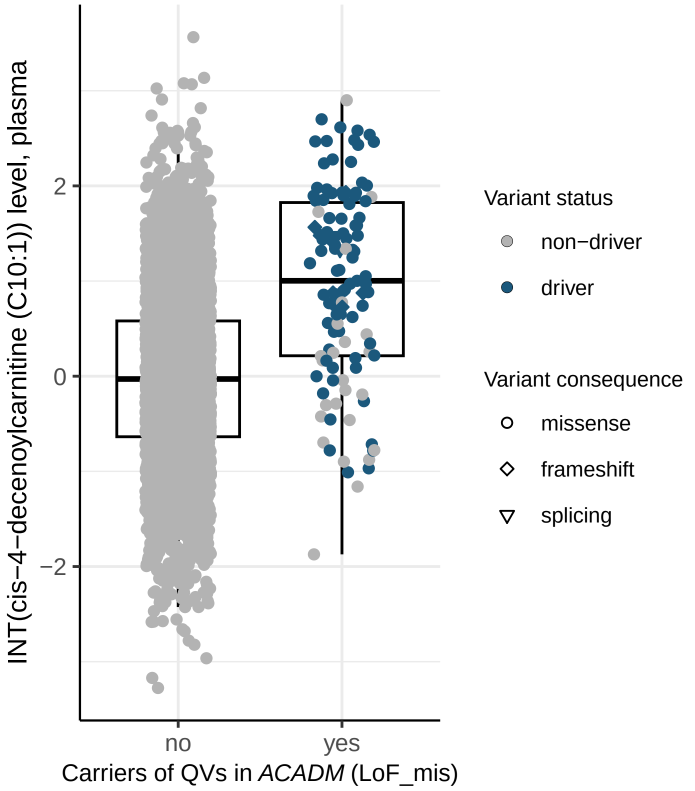

INT(cis−4−decenoylcarnitine (C10:1)) level, plasma
2
0
−2
no
yes
Carriers of QVs in ACADM (LoF_mis)
Variant status
non−driver
driver
Variant consequence
missense
frameshift
splicing

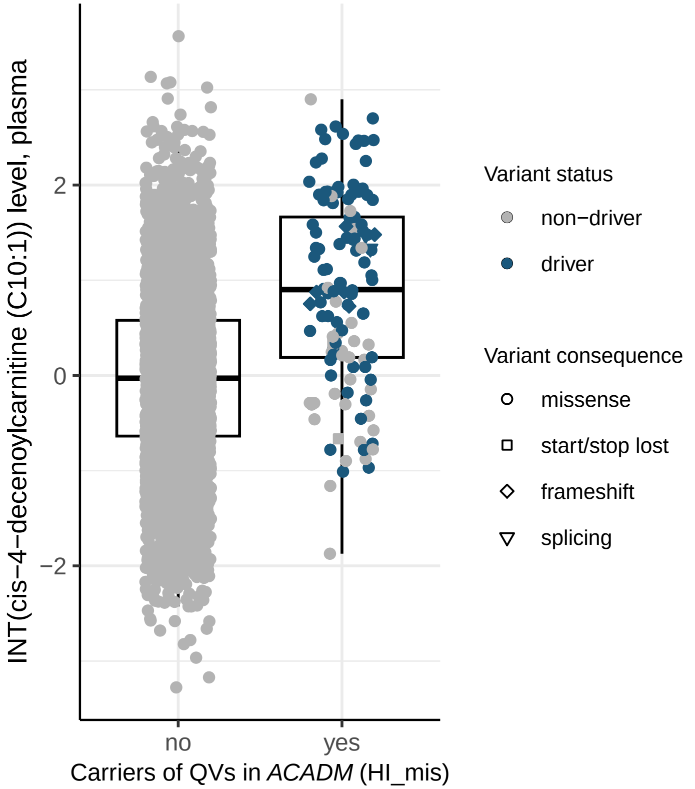
INT(cis−4−decenoylcarnitine (C10:1)) level, plasma
2
0
−2
no
yes
Carriers of QVs in *ACADM* (HI_mis)
Variant status
non−driver
driver
Variant consequence
missense
start/stop lost
frameshift
splicing

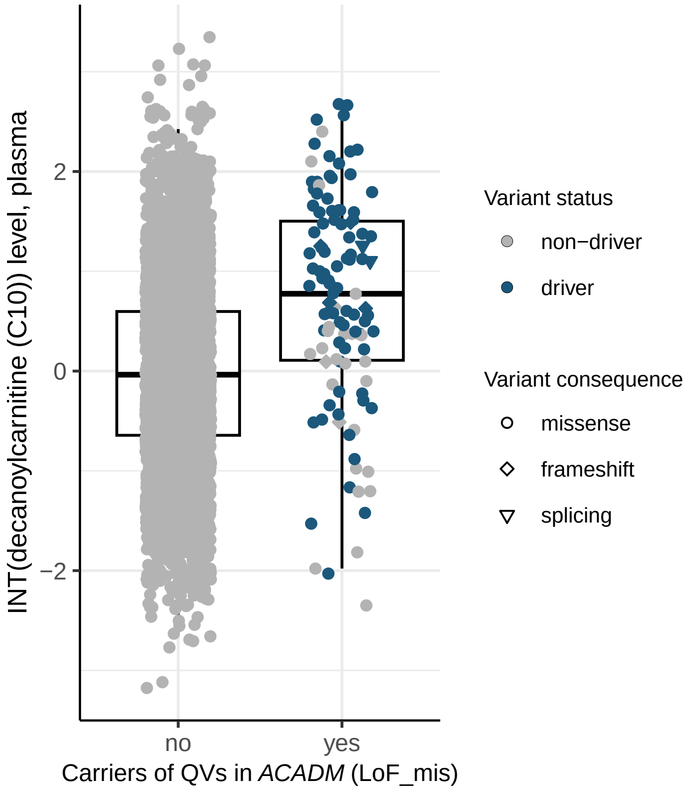
INT(decanoylcarnitine (C10)) level, plasma
2
0
−2
no
yes
Carriers of QVs in *ACADM* (LoF_mis)
Variant status
non−driver
driver
Variant consequence
missense
frameshift
splicing

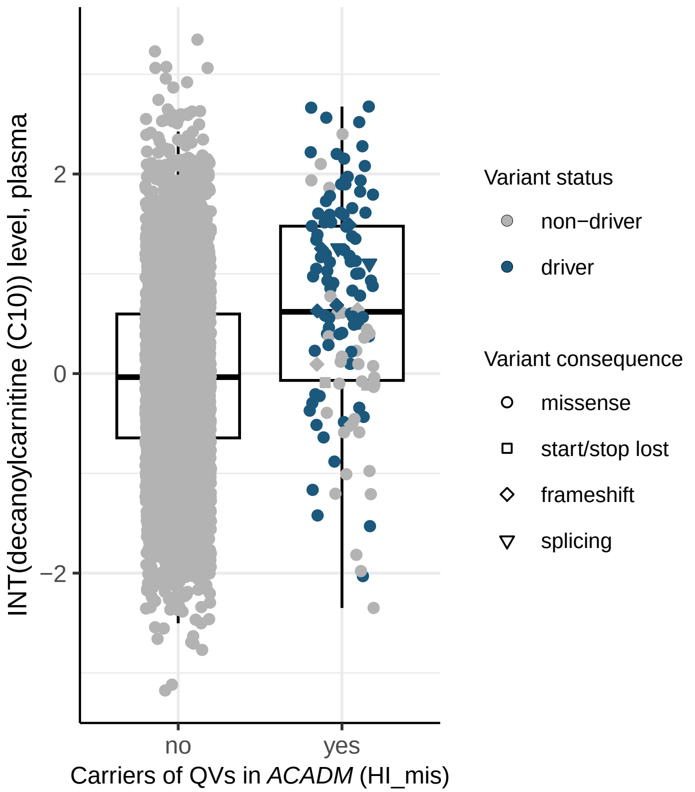

INT(decanoylcarnitine (C10)) level, plasma
2
0
−2
no
yes
Carriers of QVs in ACADM (HI_mis)
Variant status
non−driver
driver
Variant consequence
missense
start/stop lost
frameshift
splicing

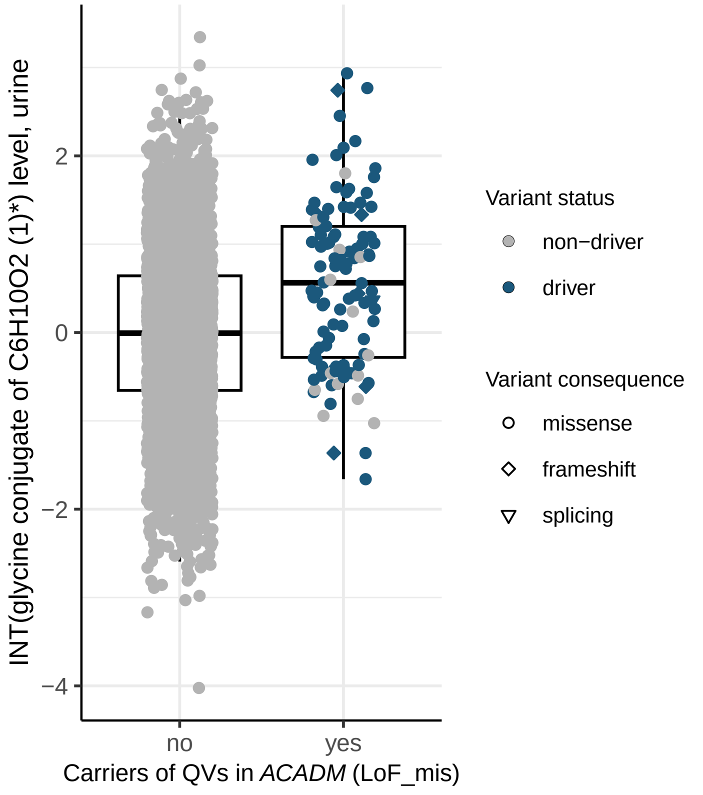

INT(glycine conjugate of C6H10O2 (1)*) level, urine
2
0
−2
−4
no
yes
Carriers of QVs in ACADM (LoF_mis)
Variant status
non−driver
driver
Variant consequence
missense
frameshift
splicing

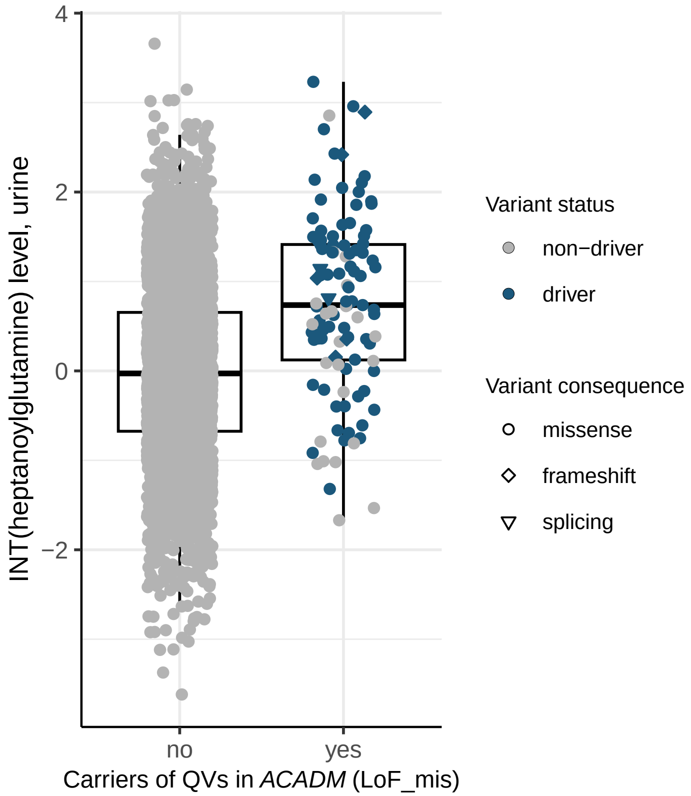

4
2
0
−2
INT(heptanoylglutamine) level, urine
no
yes
Carriers of QVs in ACADM (LoF_mis)
Variant status
non−driver
driver
Variant consequence
missense
frameshift
splicing

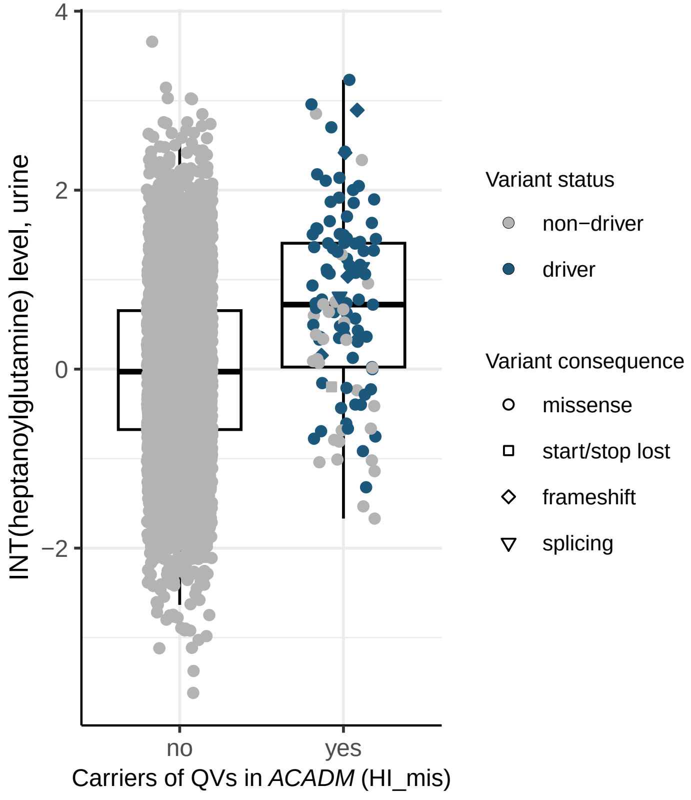

4
2
0
−2
INT(heptanoylglutamine) level, urine
no
yes
Carriers of QVs in ACADM (HI_mis)
Variant status
non−driver
driver
Variant consequence
missense
start/stop lost
frameshift
splicing

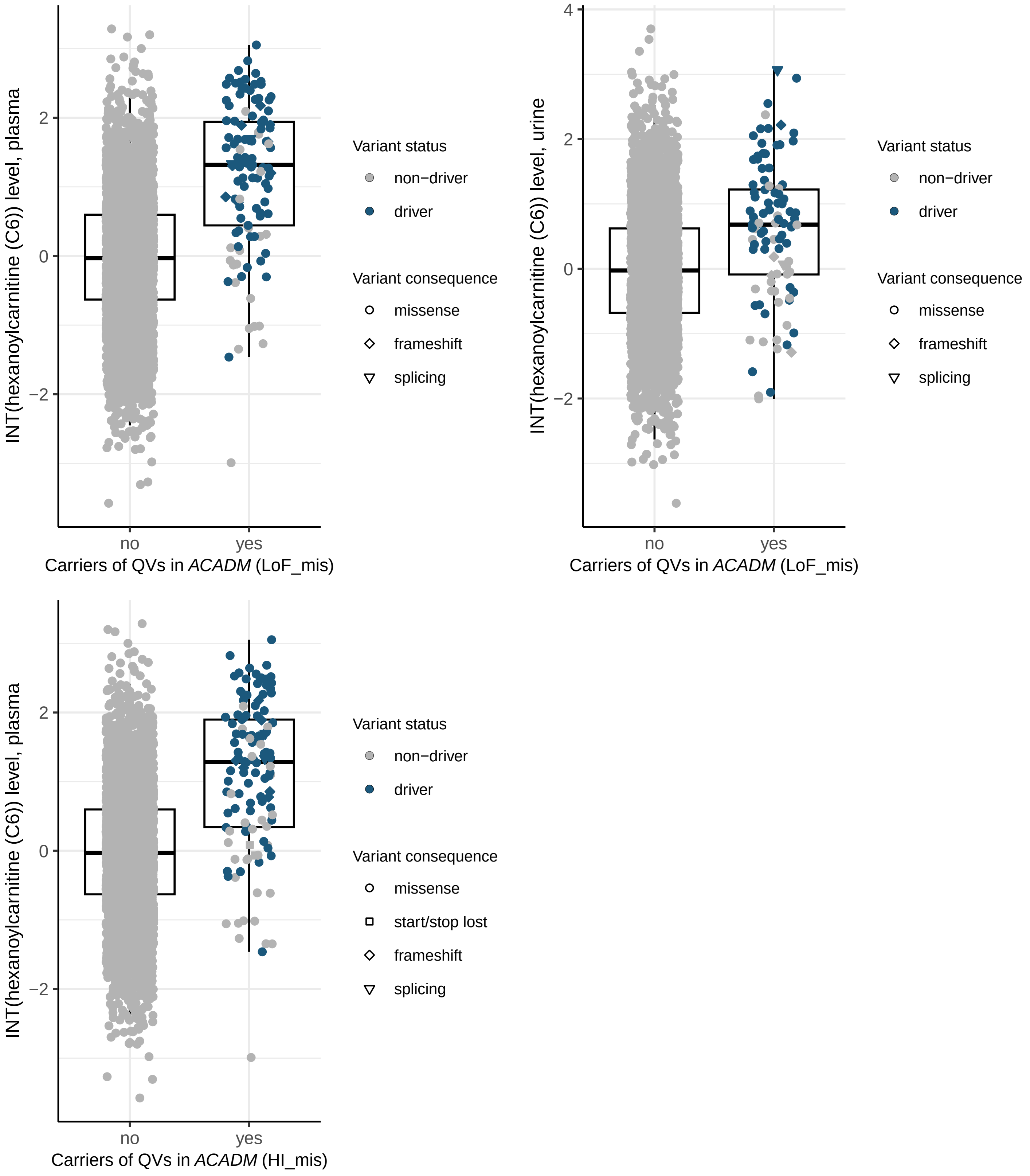
INT(hexanoylcarnitine (C6)) level, plasma
2
0
−2
no
yes
Carriers of QVs in ACADM (LoF_mis)
Variant status
non−driver
driver
Variant consequence
missense
frameshift
splicing
INT(hexanoylcarnitine (C6)) level, urine
4
2
0
−2
no
yes
Carriers of QVs in ACADM (LoF_mis)
Variant status
non−driver
driver
Variant consequence
missense
frameshift
splicing
INT(hexanoylcarnitine (C6)) level, plasma
2
0
−2
no
yes
Carriers of QVs in ACADM (HI_mis)
Variant status
non−driver
driver
Variant consequence
missense
start/stop lost
frameshift
splicing

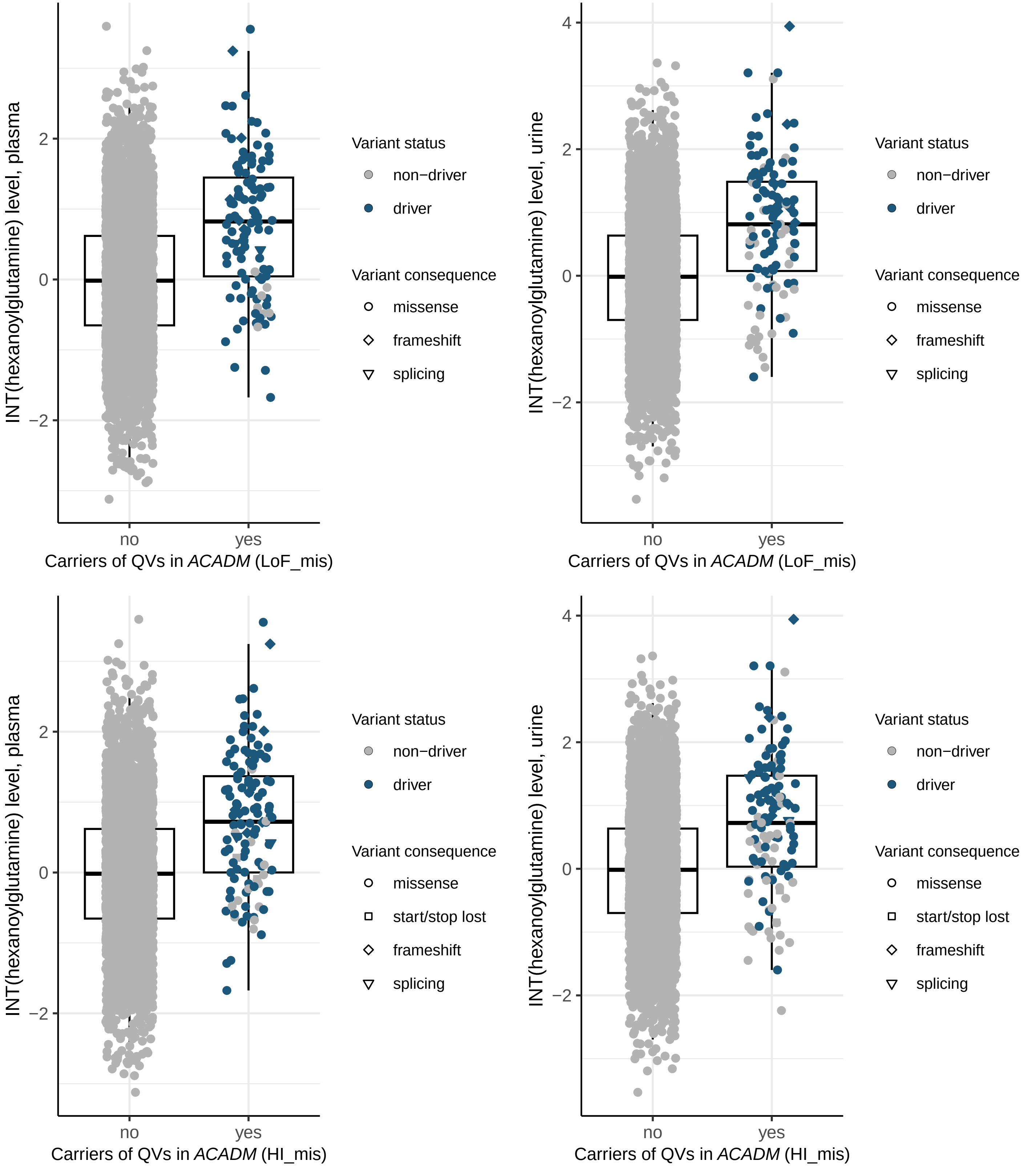
INT(hexanoylglutamine) level, plasma
Carriers of QVs in *ACADM* (LoF_mis)
no
yes
−2
0
2
Variant status
non−driver
driver
Variant consequence
missense
frameshift
splicing
INT(hexanoylglutamine) level, urine
Carriers of QVs in *ACADM* (LoF_mis)
−2
0
2
4
Variant status
non−driver
driver
Variant consequence
missense
frameshift
splicing
INT(hexanoylglutamine) level, plasma
Carriers of QVs in *ACADM* (HI_mis)
Variant status
non−driver
driver
Variant consequence
missense
start/stop lost
frameshift
splicing
INT(hexanoylglutamine) level, urine
Carriers of QVs in *ACADM* (HI_mis)
Variant status
non−driver
driver
Variant consequence
missense
start/stop lost
frameshift
splicing

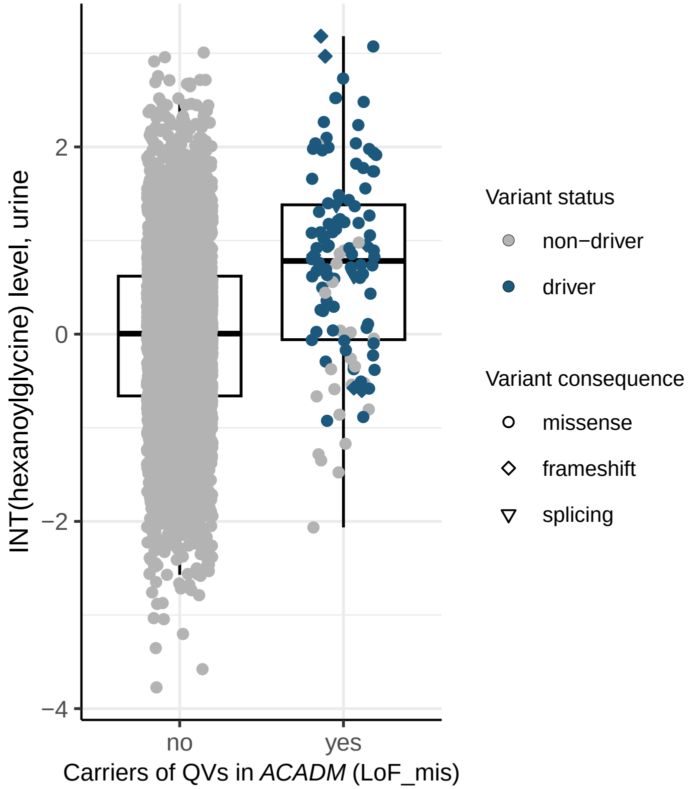
INT(hexanoylglycine) level, urine
2
0
−2
−4
no
yes
Carriers of QVs in ACADM (LoF_mis)
Variant status
non−driver
driver
Variant consequence
missense
frameshift
splicing

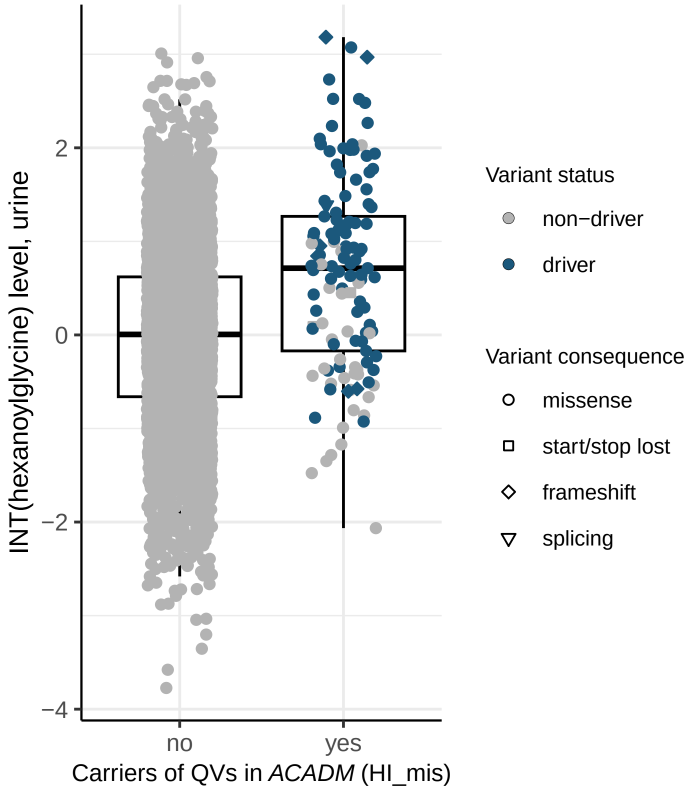
INT(hexanoylglycine) level, urine
2
0
−2
−4
no
yes
Carriers of QVs in ACADM (HI_mis)
Variant status
non−driver
driver
Variant consequence
missense
start/stop lost
frameshift
splicing

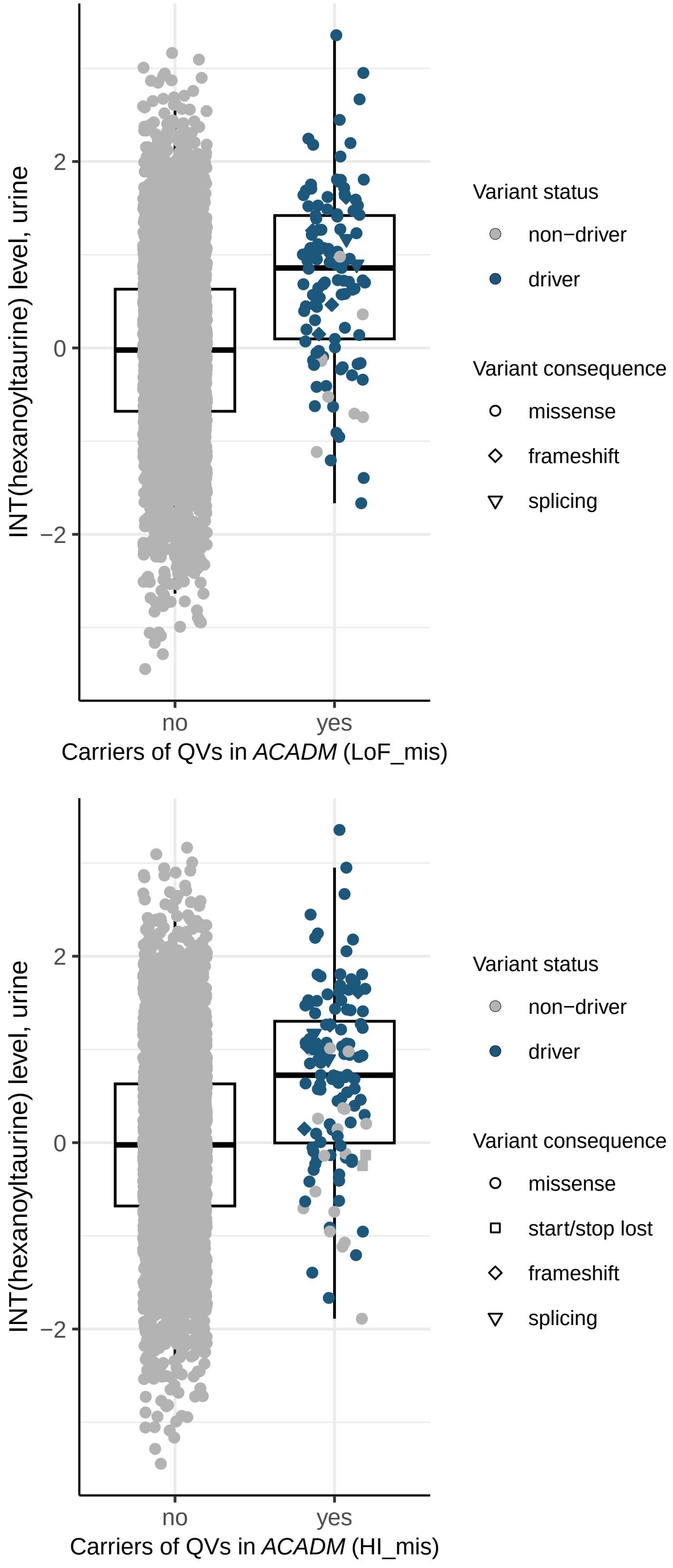
INT(hexanoyltaurine) level, urine
Carriers of QVs in *ACADM* (LoF_mis)
Variant status
non−driver
driver
Variant consequence
missense
frameshift
splicing
INT(hexanoyltaurine) level, urine
Carriers of QVs in *ACADM* (HI_mis)
Variant status
non−driver
driver
Variant consequence
missense
start/stop lost
frameshift
splicing

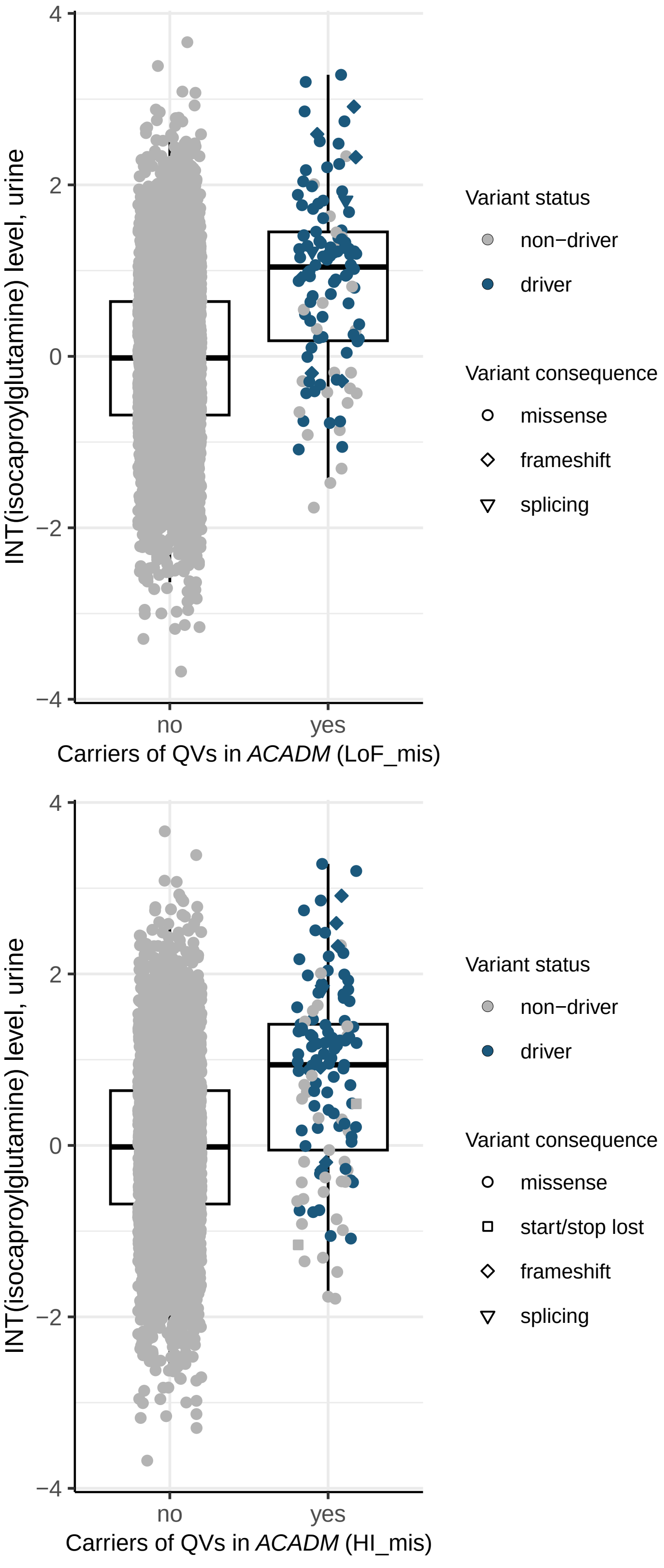
4
2
0
−2
−4
INT(isocaproylglutamine) level, urine
no
yes
Carriers of QVs in *ACADM* (LoF_mis)
Variant status
non−driver
driver
Variant consequence
missense
frameshift
splicing
4
2
0
−2
−4
INT(isocaproylglutamine) level, urine
no
yes
Carriers of QVs in *ACADM* (HI_mis)
Variant status
non−driver
driver
Variant consequence
missense
start/stop lost
frameshift
splicing

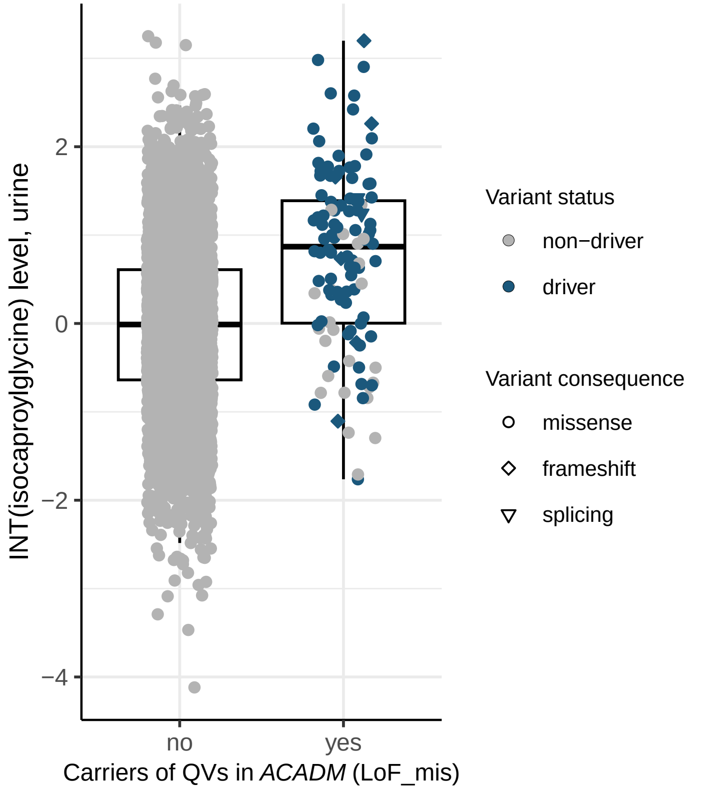
INT(isocaproylglycine) level, urine
2
0
−2
−4
no
yes
Carriers of QVs in ACADM (LoF_mis)
Variant status
non−driver
driver
Variant consequence
missense
frameshift
splicing

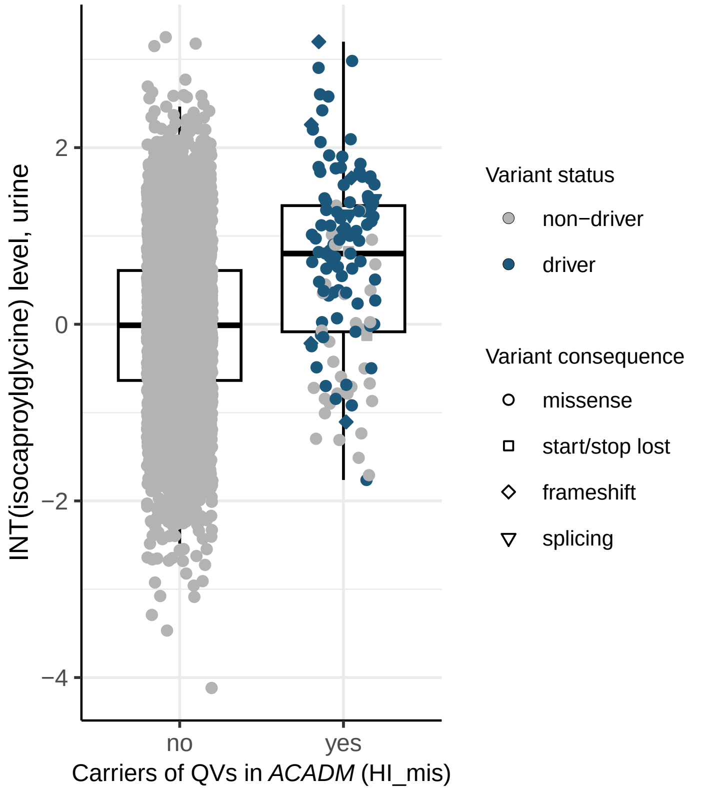
INT(isocaproylglycine) level, urine
2
0
−2
−4
no
yes
Carriers of QVs in ACADM (HI_mis)
Variant status
non−driver
driver
Variant consequence
missense
start/stop lost
frameshift
splicing

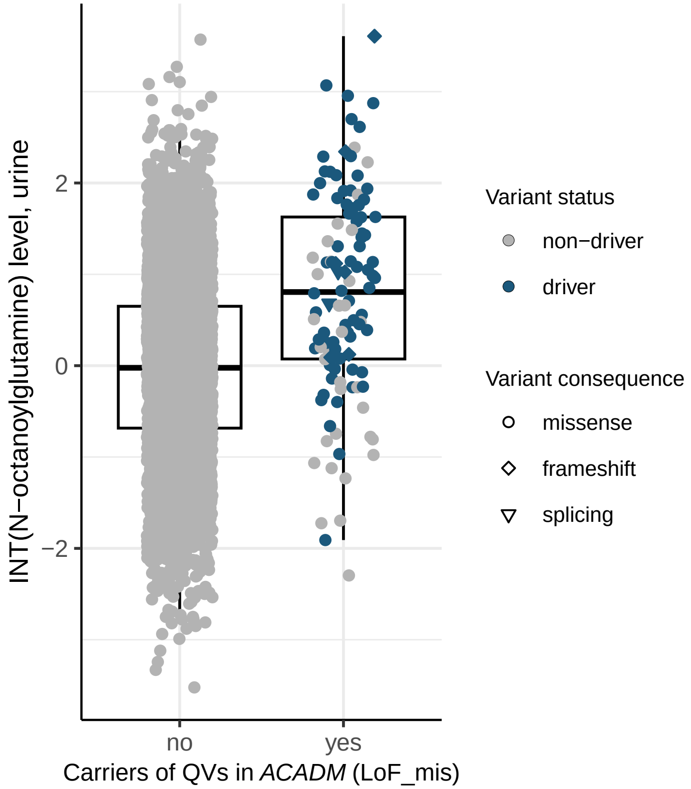
INT(N−octanoylglutamine) level, urine
2
0
−2
no
yes
Carriers of QVs in *ACADM* (LoF_mis)
Variant status
non−driver
driver
Variant consequence
missense
frameshift
splicing

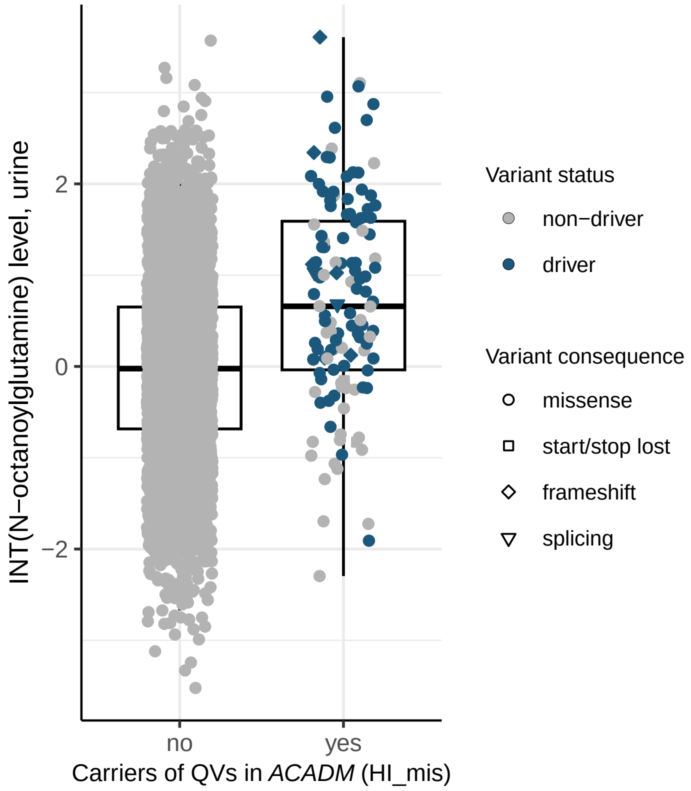
INT(N−octanoylglutamine) level, urine
2
0
−2
no
yes
Carriers of QVs in *ACADM* (HI_mis)
Variant status
non−driver
driver
Variant consequence
missense
start/stop lost
frameshift
splicing

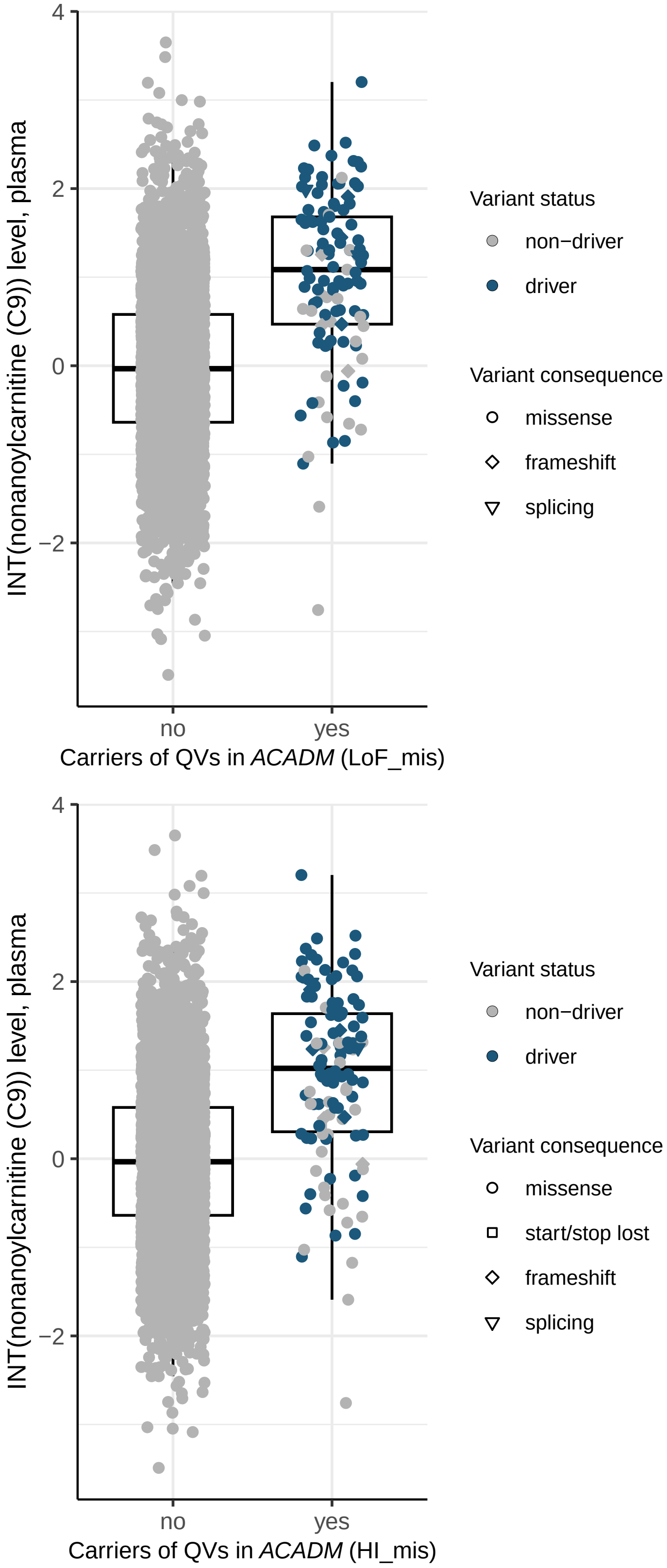

INT(nonanoylcarnitine (C9)) level, plasma
4
2
0
−2
no
yes
Carriers of QVs in ACADM (LoF_mis)
Variant status
non−driver
driver
Variant consequence
missense
frameshift
splicing
INT(nonanoylcarnitine (C9)) level, plasma
4
2
0
−2
no
yes
Carriers of QVs in ACADM (HI_mis)
Variant status
non−driver
driver
Variant consequence
missense
start/stop lost
frameshift
splicing

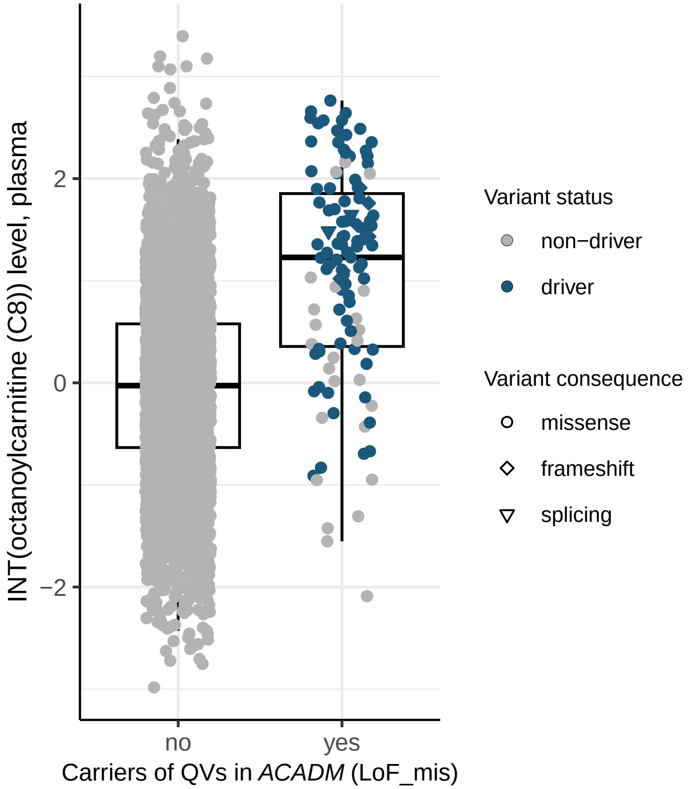
INT(octanoylcarnitine (C8)) level, plasma
2
0
−2
no
yes
Carriers of QVs in *ACADM* (LoF_mis)
Variant status
non−driver
driver
Variant consequence
missense
frameshift
splicing

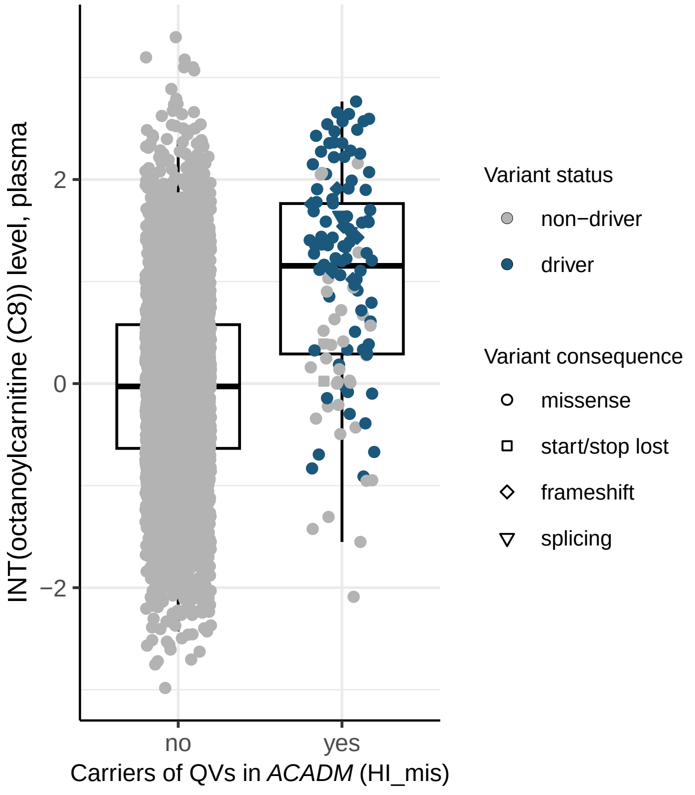
INT(octanoylcarnitine (C8)) level, plasma
2
0
−2
no
yes
Carriers of QVs in *ACADM* (HI_mis)
Variant status
non−driver
driver
Variant consequence
missense
start/stop lost
frameshift
splicing

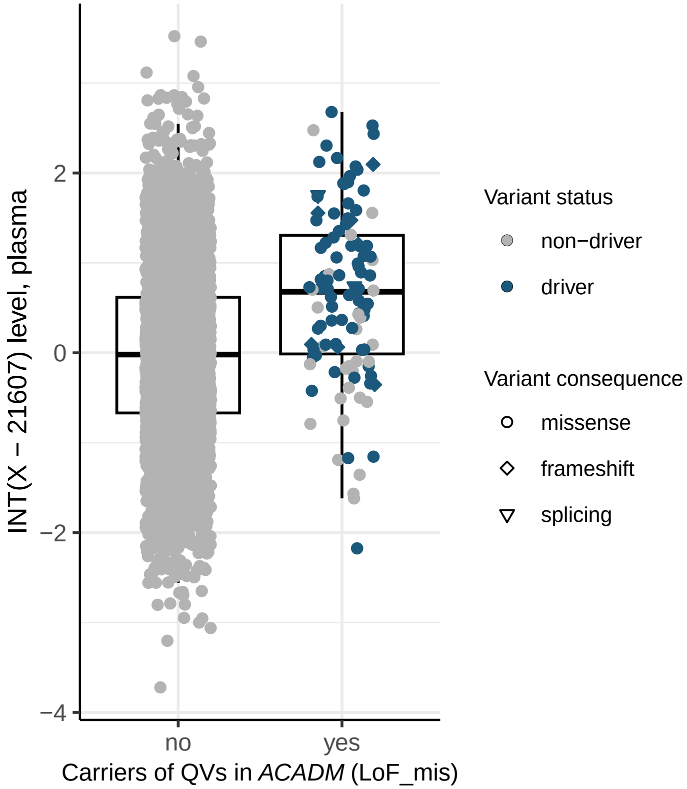
INT(X − 21607) level, plasma
2
0
−2
−4
no
yes
Carriers of QVs in ACADM (LoF_mis)
Variant status
non−driver
driver
Variant consequence
missense
frameshift
splicing

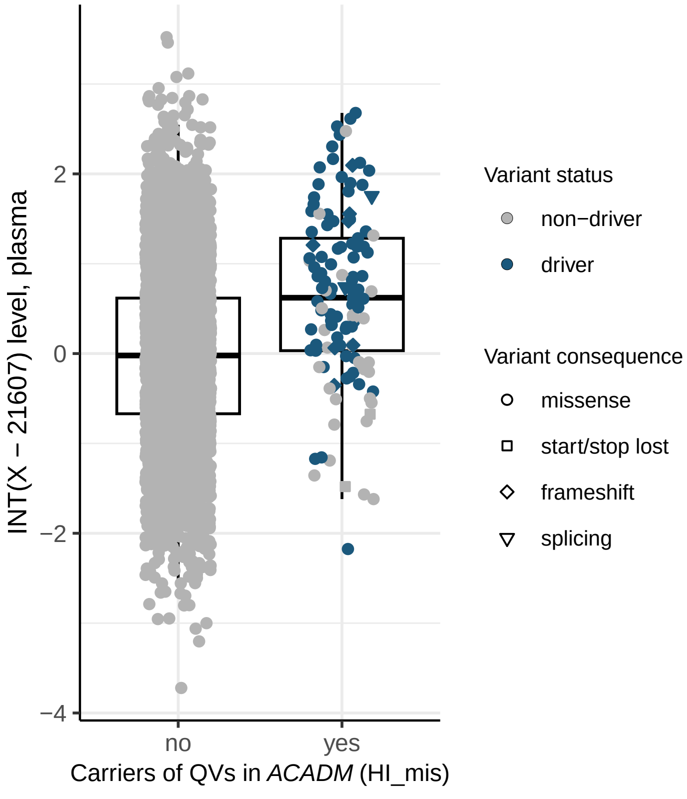
INT(X − 21607) level, plasma
2
0
−2
−4
no
yes
Carriers of QVs in ACADM (HI_mis)
Variant status
non−driver
driver
Variant consequence
missense
start/stop lost
frameshift
splicing

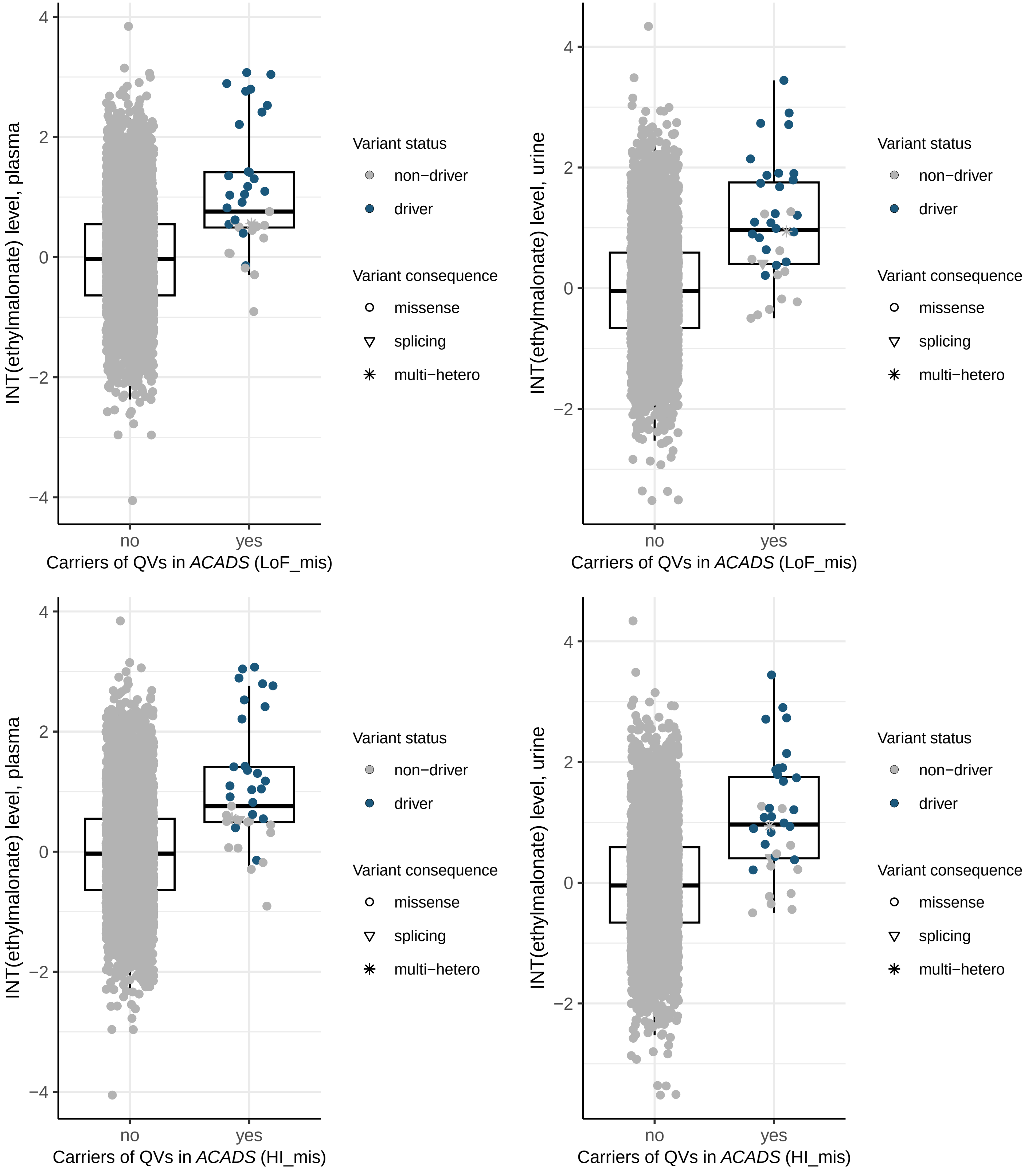
INT(ethylmalonate) level, plasma
Carriers of QVs in ACADS (LoF_mis)
INT(ethylmalonate) level, urine
Carriers of QVs in ACADS (LoF_mis)
INT(ethylmalonate) level, plasma
Carriers of QVs in ACADS (HI_mis)
INT(ethylmalonate) level, urine
Carriers of QVs in ACADS (HI_mis)
no
yes
4
2
0
−2
−4
Variant status
non−driver
driver
Variant consequence
missense
splicing
multi−hetero

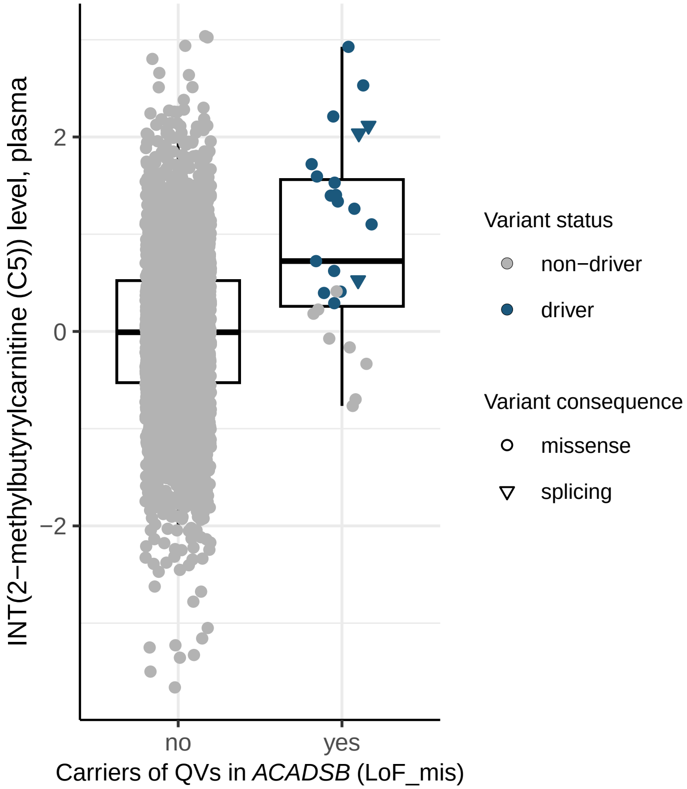
INT(2−methylbutyrylcarnitine (C5)) level, plasma
2
0
−2
no
yes
Carriers of QVs in ACADSB (LoF_mis)
Variant status
non−driver
driver
Variant consequence
missense
splicing

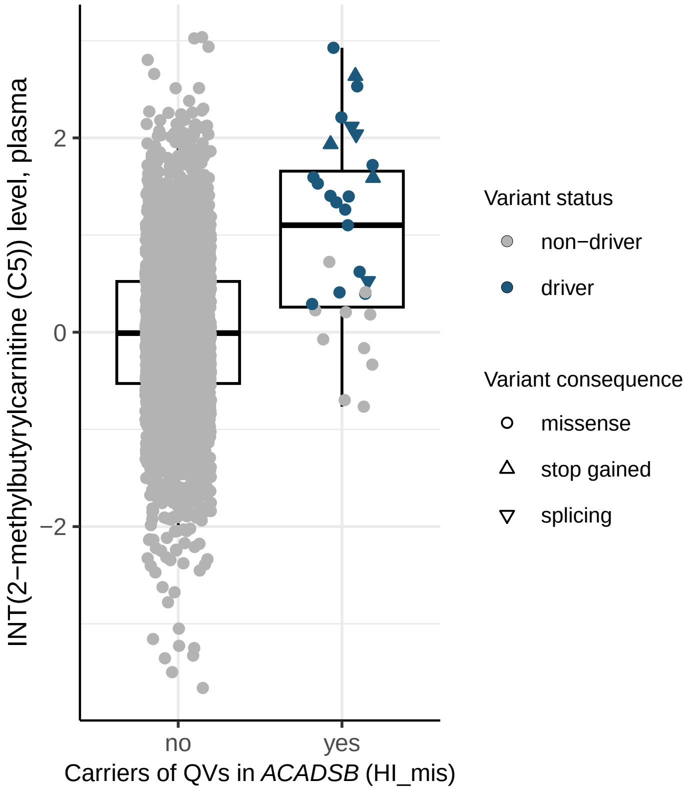
INT(2−methylbutyrylcarnitine (C5)) level, plasma
2
0
−2
no
yes
Carriers of QVs in ACADSB (HI_mis)
Variant status
non−driver
driver
Variant consequence
missense
stop gained
splicing

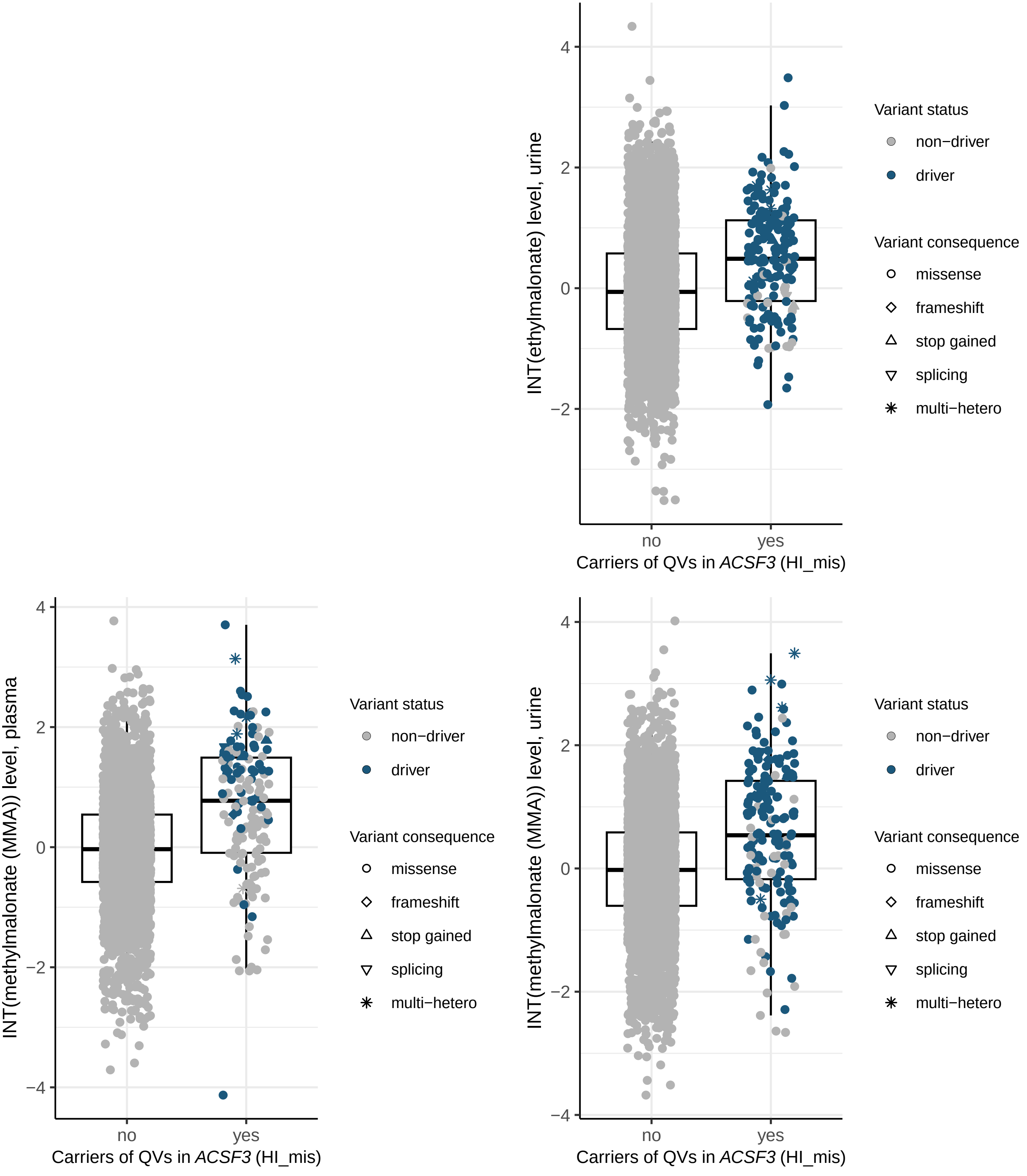

INT(ethylmalonate) level, urine
4
2
0
−2
no
yes
Carriers of QVs in *ACSF3* (HI_mis)
Variant status
non−driver
driver
Variant consequence
missense
frameshift
stop gained
splicing
multi−hetero
INT(methylmalonate (MMA)) level, plasma
4
2
0
−2
−4
no
yes
Carriers of QVs in *ACSF3* (HI_mis)
Variant status
non−driver
driver
Variant consequence
missense
frameshift
stop gained
splicing
multi−hetero
INT(methylmalonate (MMA)) level, urine
4
2
0
−2
−4
no
yes
Carriers of QVs in *ACSF3* (HI_mis)
Variant status
non−driver
driver
Variant consequence
missense
frameshift
stop gained
splicing
multi−hetero

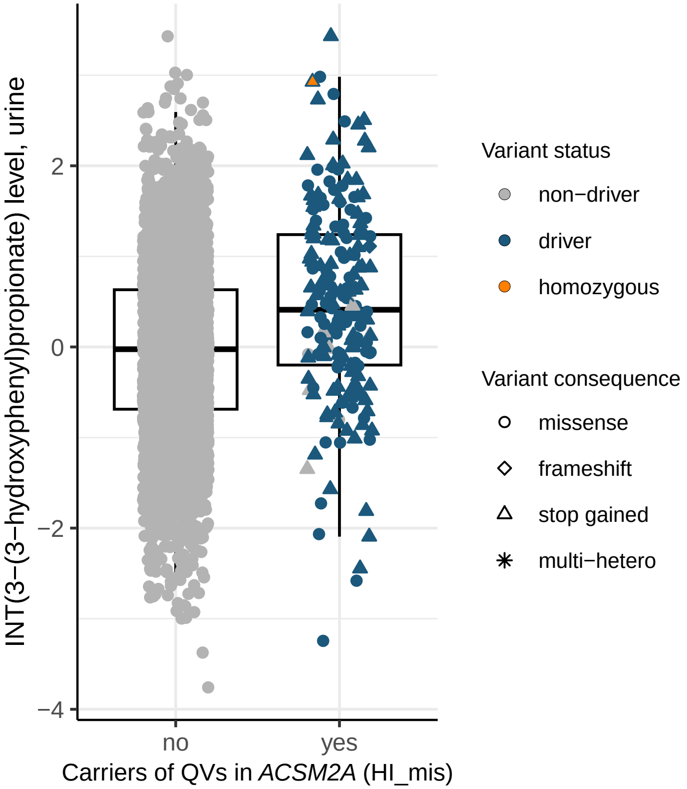

INT(3−(3−hydroxyphenyl)propionate) level, urine
2
0
−2
−4
no
yes
Carriers of QVs in *ACSM2A* (HI_mis)
Variant status
non−driver
driver
homozygous
Variant consequence
missense
frameshift
stop gained
multi−hetero

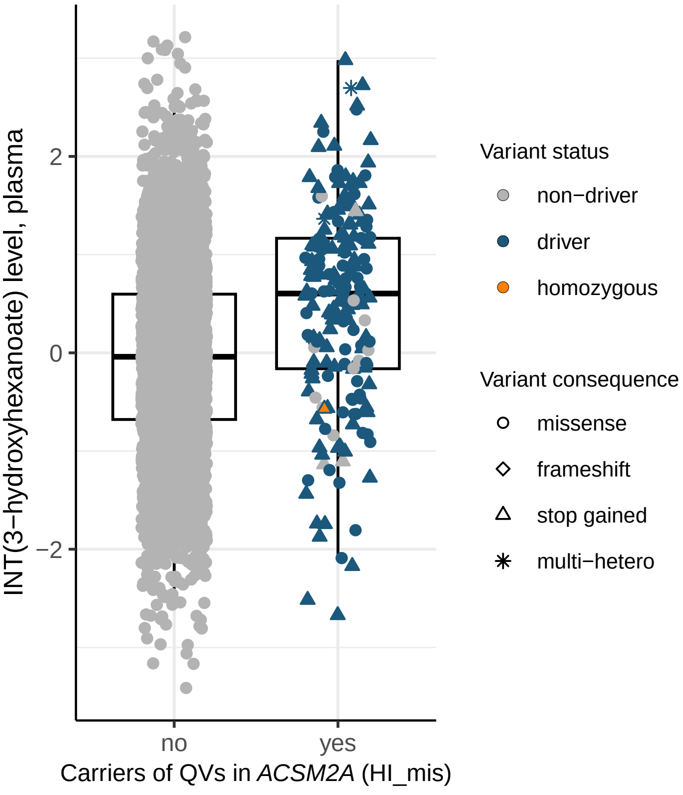

INT(3−hydroxyhexanoate) level, plasma
2
0
−2
no
yes
Carriers of QVs in *ACSM2A* (HI_mis)
Variant status
non−driver
driver
homozygous
Variant consequence
missense
frameshift
stop gained
multi−hetero

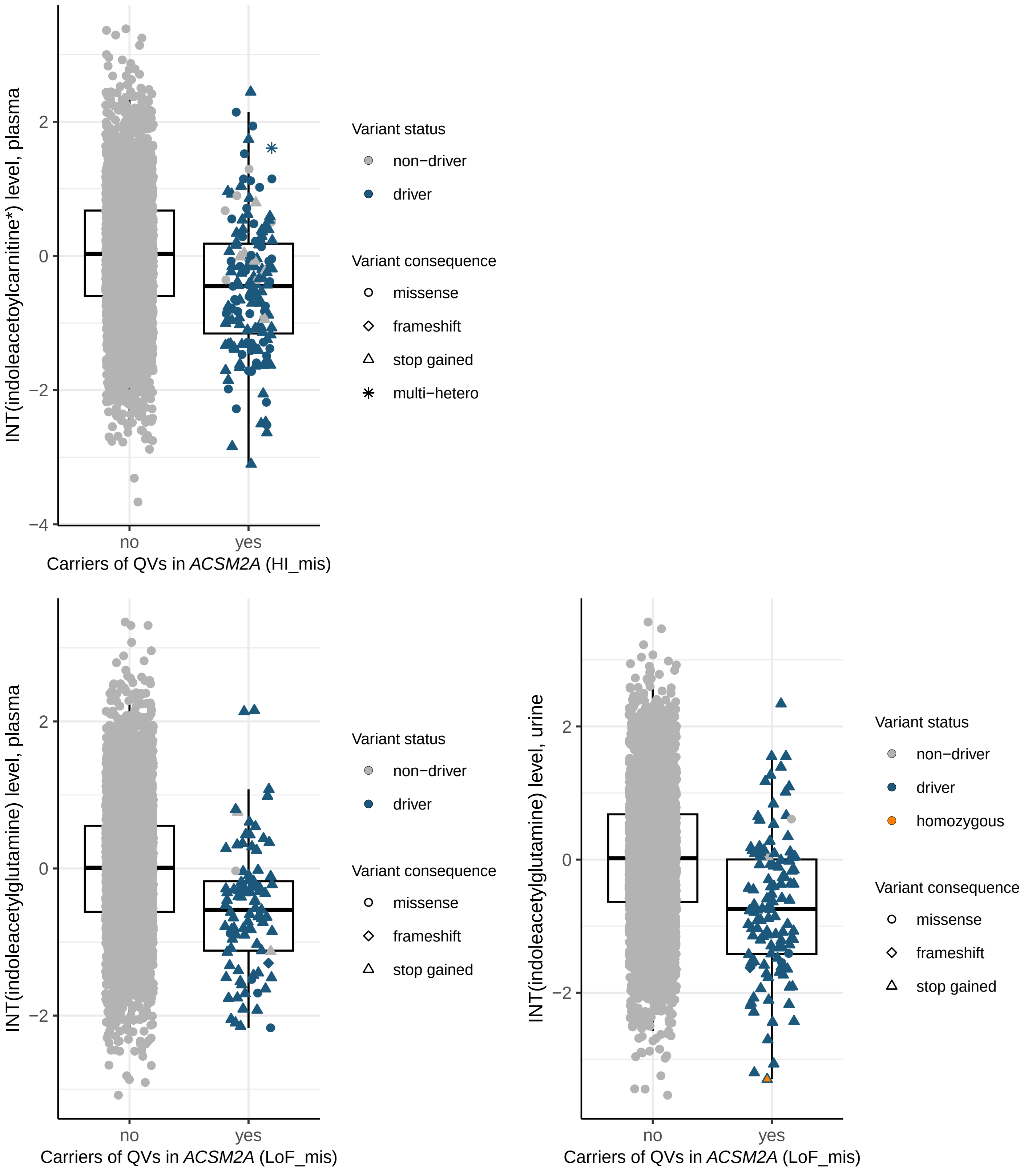

INT(indoleacetoylcarnitine*) level, plasma
2
0
−2
−4
no
yes
Carriers of QVs in ACSM2A (HI_mis)
Variant status
non−driver
driver
Variant consequence
missense
frameshift
stop gained
multi−hetero
INT(indoleacetylglutamine) level, plasma
2
0
−2
no
yes
Carriers of QVs in ACSM2A (LoF_mis)
Variant status
non−driver
driver
Variant consequence
missense
frameshift
stop gained
INT(indoleacetylglutamine) level, urine
2
0
−2
no
yes
Carriers of QVs in ACSM2A (LoF_mis)
Variant status
non−driver
driver
homozygous
Variant consequence
missense
frameshift
stop gained

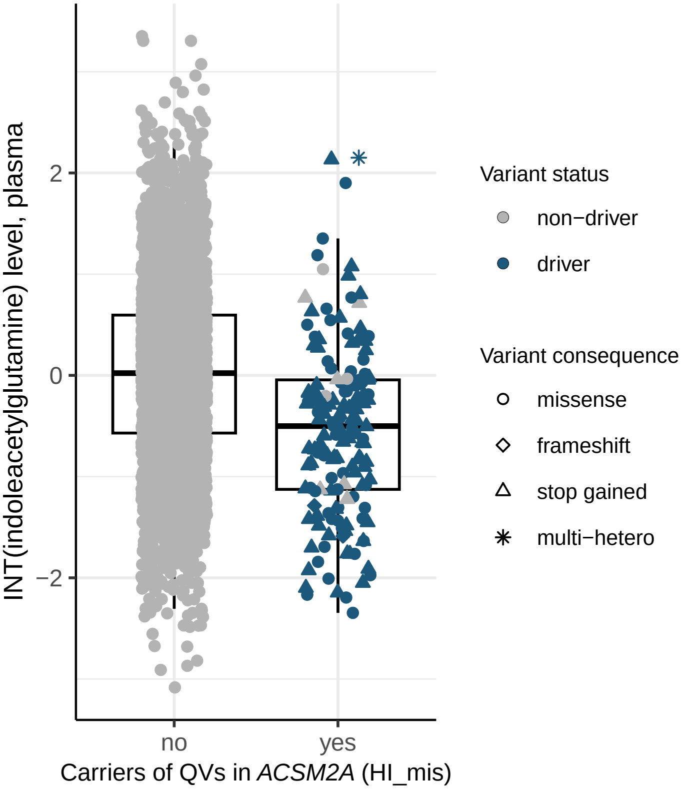
INT(indoleacetylglutamine) level, plasma
2
0
−2
no
yes
Carriers of QVs in *ACSM2A* (HI_mis)
Variant status
non−driver
driver
Variant consequence
missense
frameshift
stop gained
multi−hetero

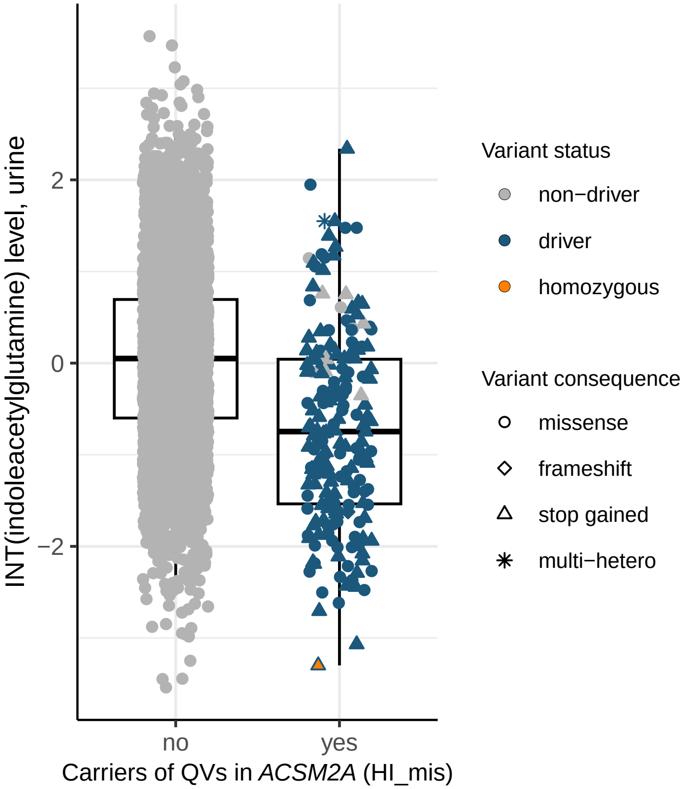
INT(indoleacetylglutamine) level, urine
2
0
−2
no
yes
Carriers of QVs in *ACSM2A* (HI_mis)
Variant status
non−driver
driver
homozygous
Variant consequence
missense
frameshift
stop gained
multi−hetero

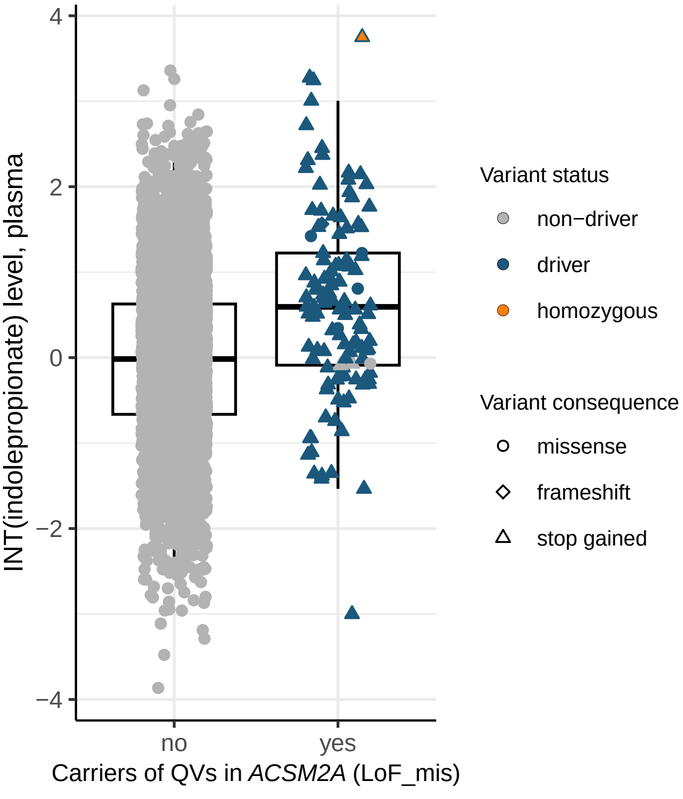
INT(indolepropionate) level, plasma
4
2
0
−2
−4
no
yes
Carriers of QVs in *ACSM2A* (LoF_mis)
Variant status
non−driver
driver
homozygous
Variant consequence
missense
frameshift
stop gained

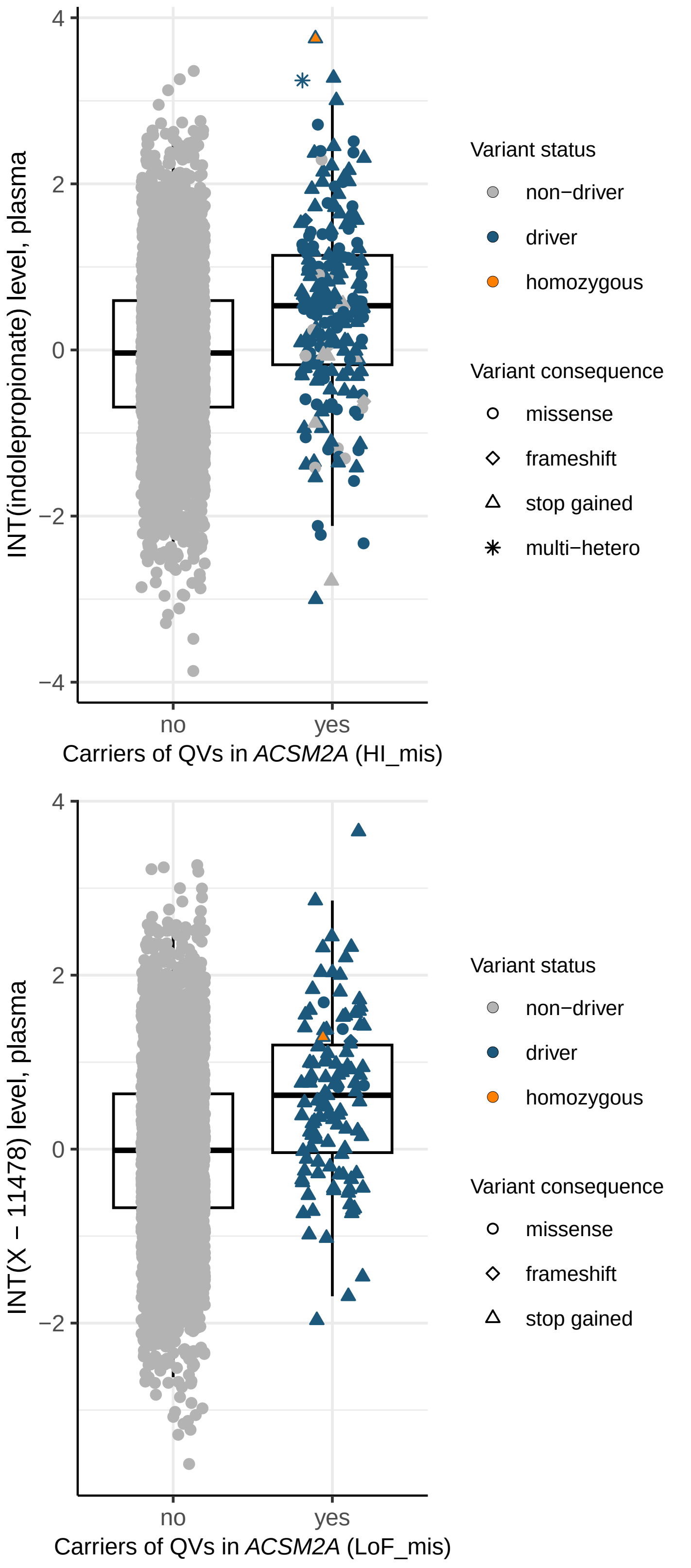

INT(indolepropionate) level, plasma
4
2
0
−2
−4
no
yes
Carriers of QVs in *ACSM2A* (HI_mis)
Variant status
non−driver
driver
homozygous
Variant consequence
missense
frameshift
stop gained
multi−hetero
INT(X − 11478) level, plasma
4
2
0
−2
no
yes
Carriers of QVs in *ACSM2A* (LoF_mis)
Variant status
non−driver
driver
homozygous
Variant consequence
missense
frameshift
stop gained

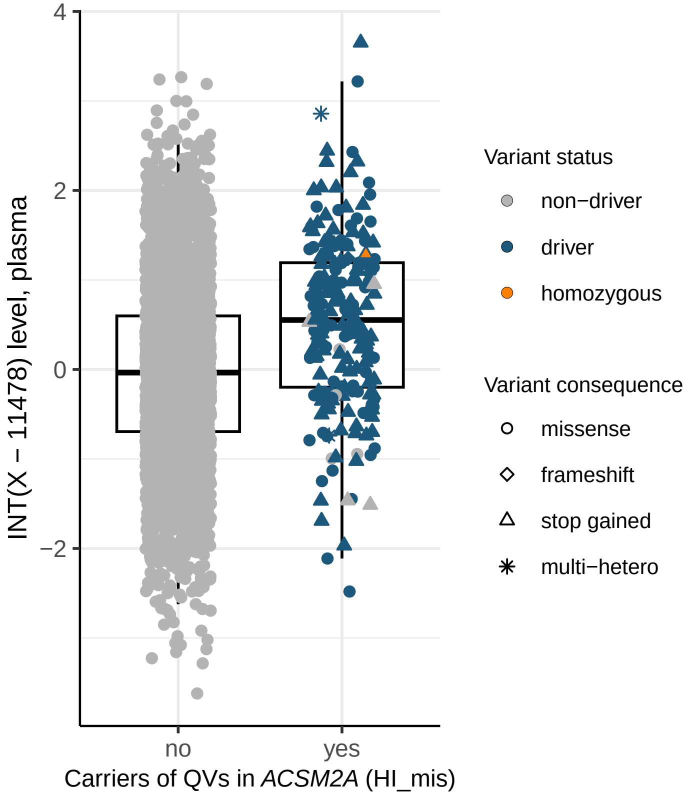

4
2
0
−2
INT(X − 11478) level, plasma
no
yes
Carriers of QVs in *ACSM2A* (HI_mis)
Variant status
non−driver
driver
homozygous
Variant consequence
missense
frameshift
stop gained
multi−hetero

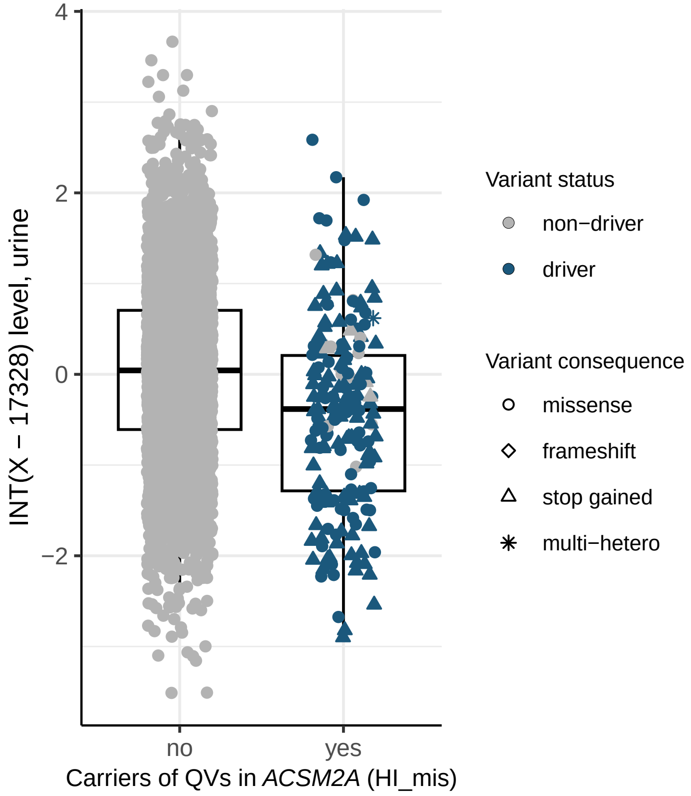

4
2
0
−2
INT(X − 17328) level, urine
no
yes
Carriers of QVs in *ACSM2A* (HI_mis)
Variant status
non−driver
driver
Variant consequence
missense
frameshift
stop gained
multi−hetero

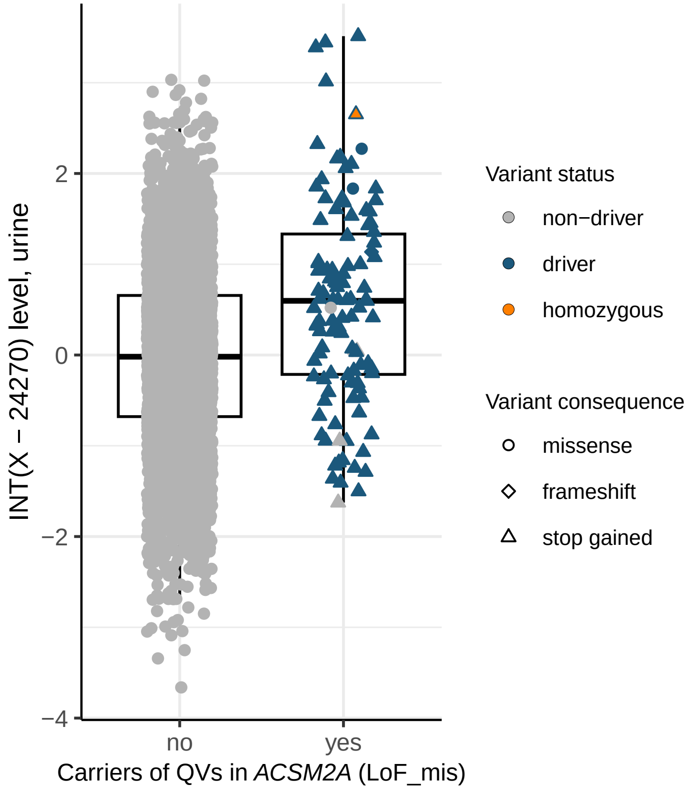
INT(X – 24270) level, urine
2
0
−2
−4
no
yes
Carriers of QVs in ACSM2A (LoF_mis)
Variant status
non−driver
driver
homozygous
Variant consequence
missense
frameshift
stop gained

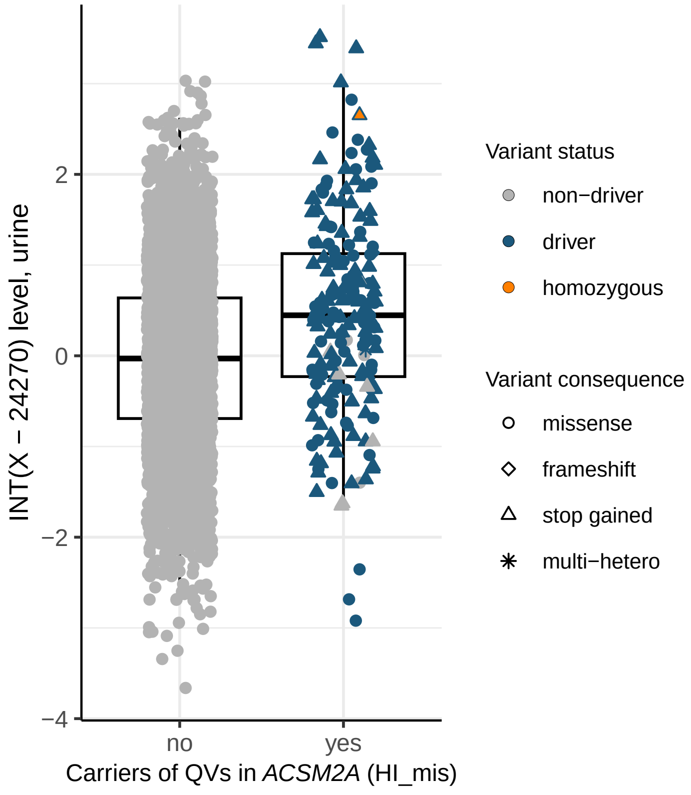
INT(X – 24270) level, urine
2
0
−2
−4
no
yes
Carriers of QVs in ACSM2A (HI_mis)
Variant status
non−driver
driver
homozygous
Variant consequence
missense
frameshift
stop gained
multi−hetero

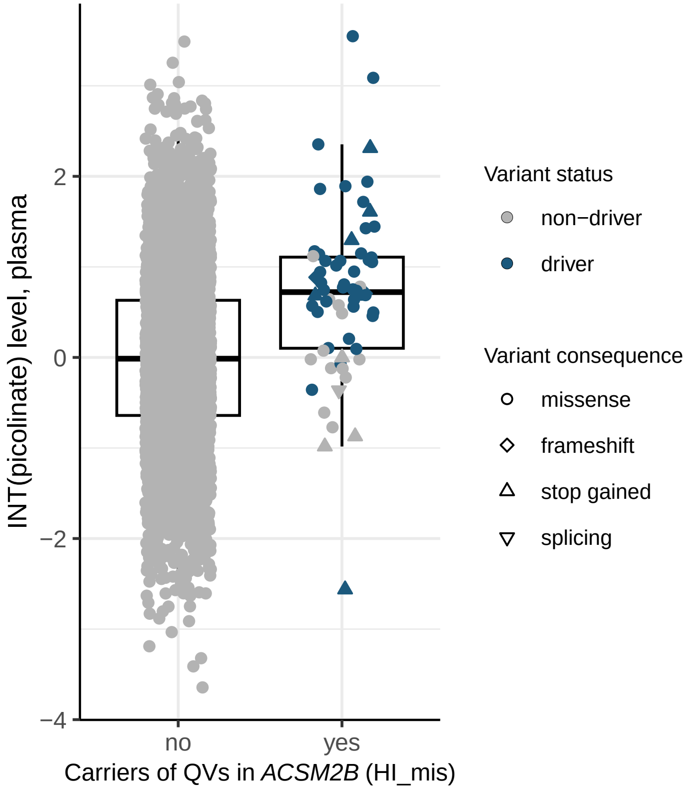
INT(picolinate) level, plasma
Carriers of QVs in *ACSM2B* (HI_mis)
no
yes
2
0
−2
−4
Variant status
non−driver
driver
Variant consequence
missense
frameshift
stop gained
splicing

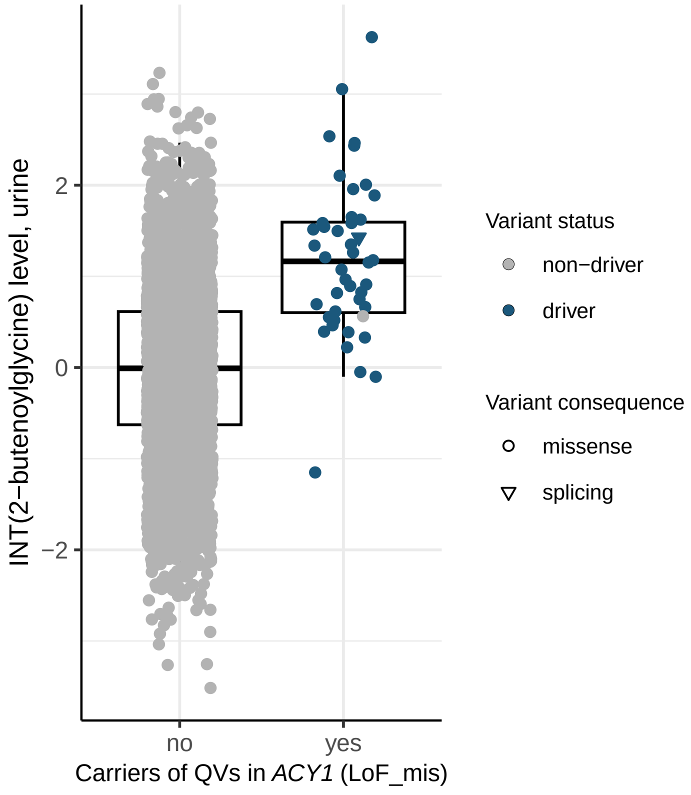
INT(2−butenoylglycine) level, urine
Carriers of QVs in *ACY1* (LoF_mis)
no
yes
2
0
−2
Variant status
non−driver
driver
Variant consequence
missense
splicing

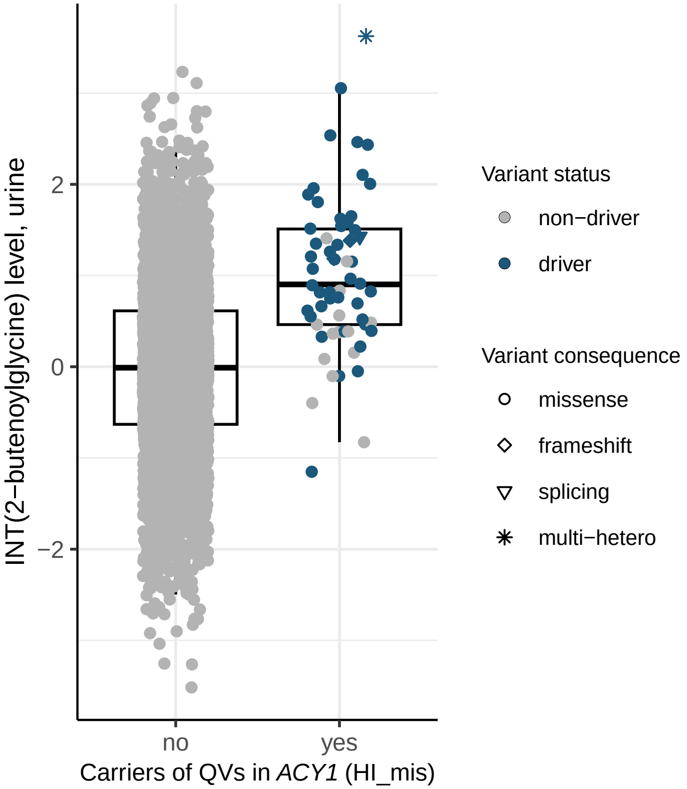

INT(2−butenoylglycine) level, urine
2
0
−2
no
yes
Carriers of QVs in ACY1 (HI_mis)
Variant status
non−driver
driver
Variant consequence
missense
frameshift
splicing
multi−hetero

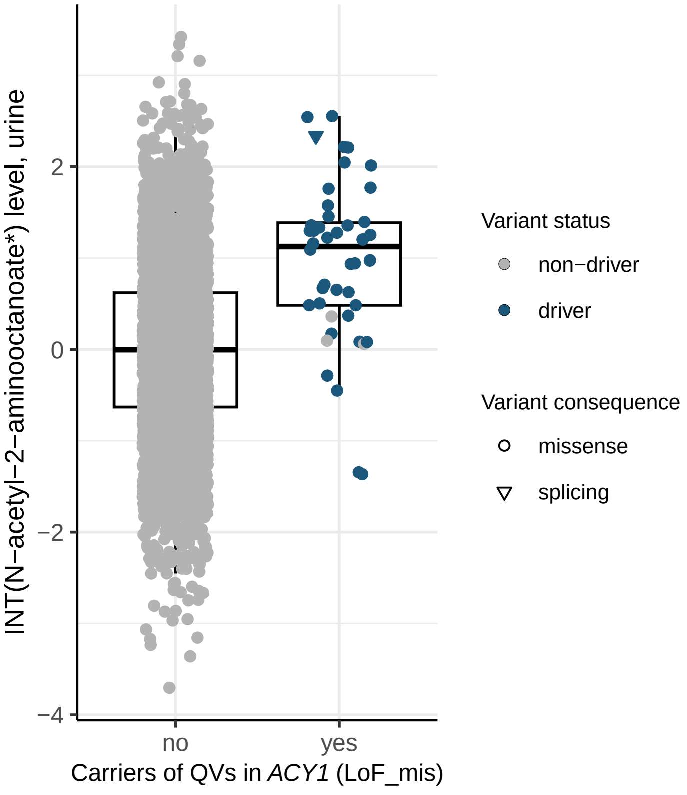

INT(N−acetyl−2−aminooctanoate*) level, urine
2
0
−2
−4
no
yes
Carriers of QVs in ACY1 (LoF_mis)
Variant status
non−driver
driver
Variant consequence
missense
splicing

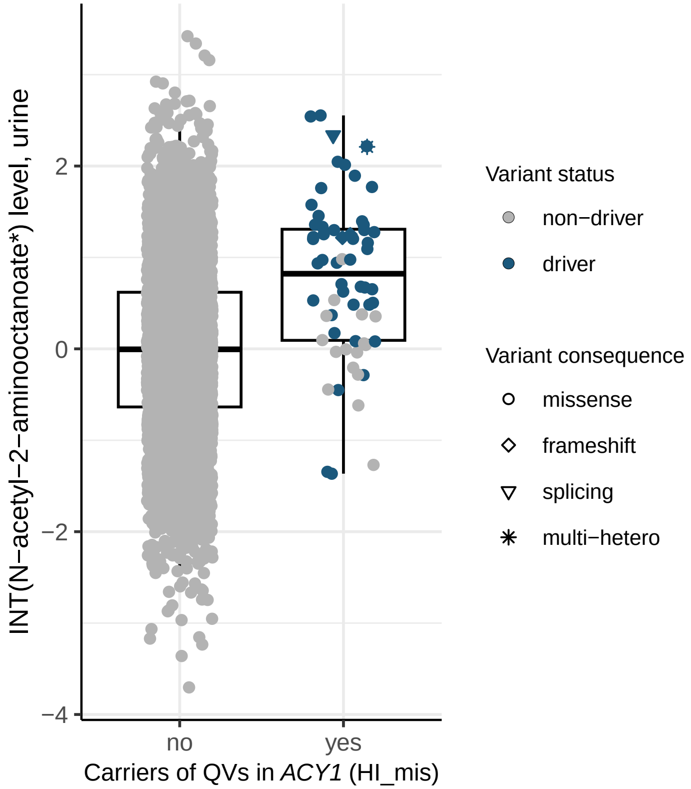

INT(N−acetyl−2−aminooctanoate*) level, urine
2
0
−2
−4
no
yes
Carriers of QVs in *ACY1* (HI_mis)
Variant status
non−driver
driver
Variant consequence
missense
frameshift
splicing
multi−hetero

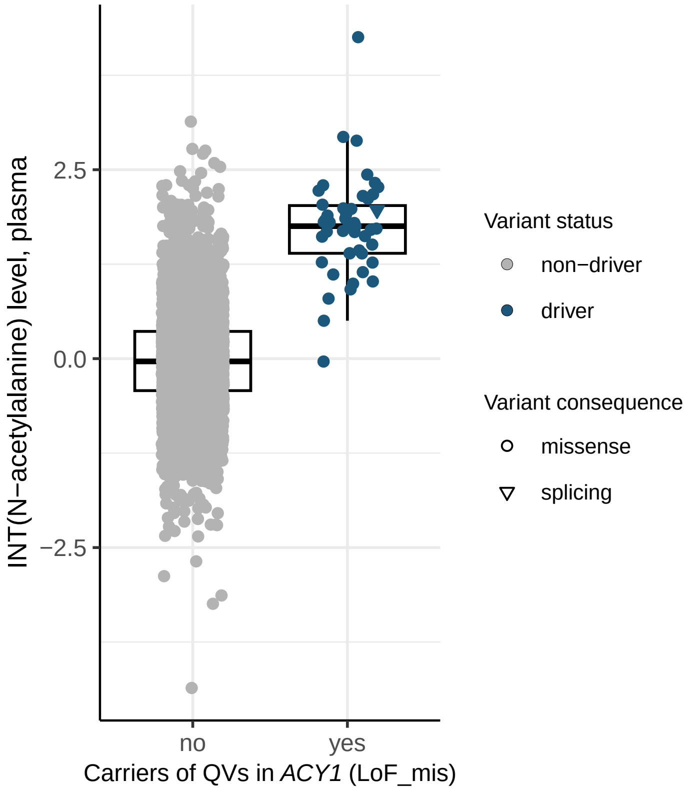

INT(N−acetylalanine) level, plasma
2.5
0.0
−2.5
no
yes
Carriers of QVs in *ACY1* (LoF_mis)
Variant status
non−driver
driver
Variant consequence
missense
splicing

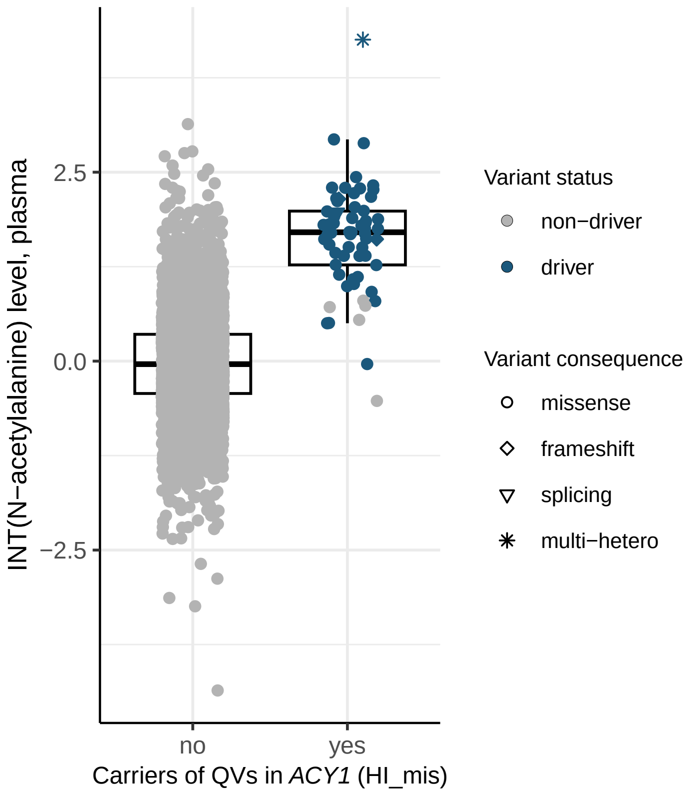
INT(N−acetylalanine) level, plasma
2.5
0.0
−2.5
no
yes
Carriers of QVs in *ACY1* (HI_mis)
Variant status
non−driver
driver
Variant consequence
missense
frameshift
splicing
multi−hetero

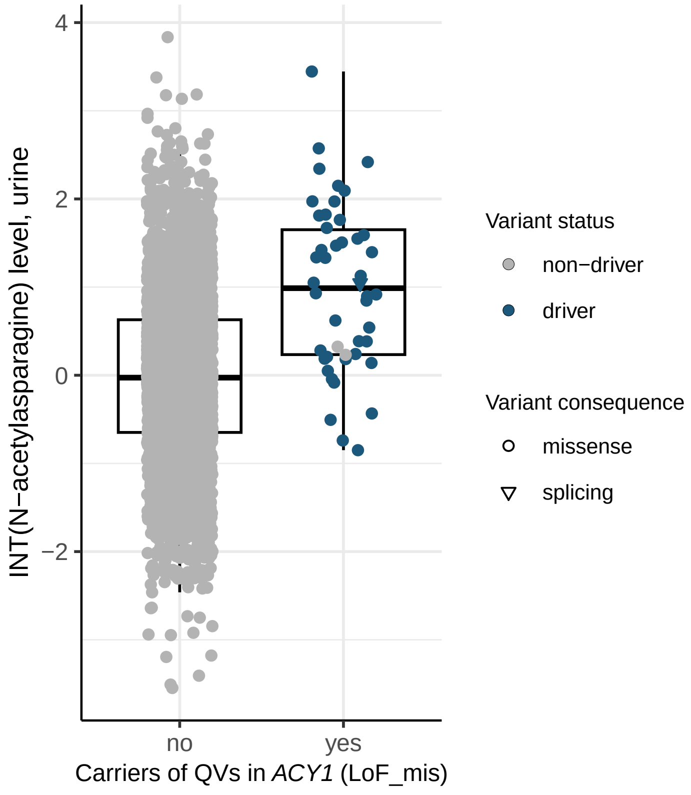
INT(N−acetylasparagine) level, urine
4
2
0
−2
no
yes
Carriers of QVs in *ACY1* (LoF_mis)
Variant status
non−driver
driver
Variant consequence
missense
splicing

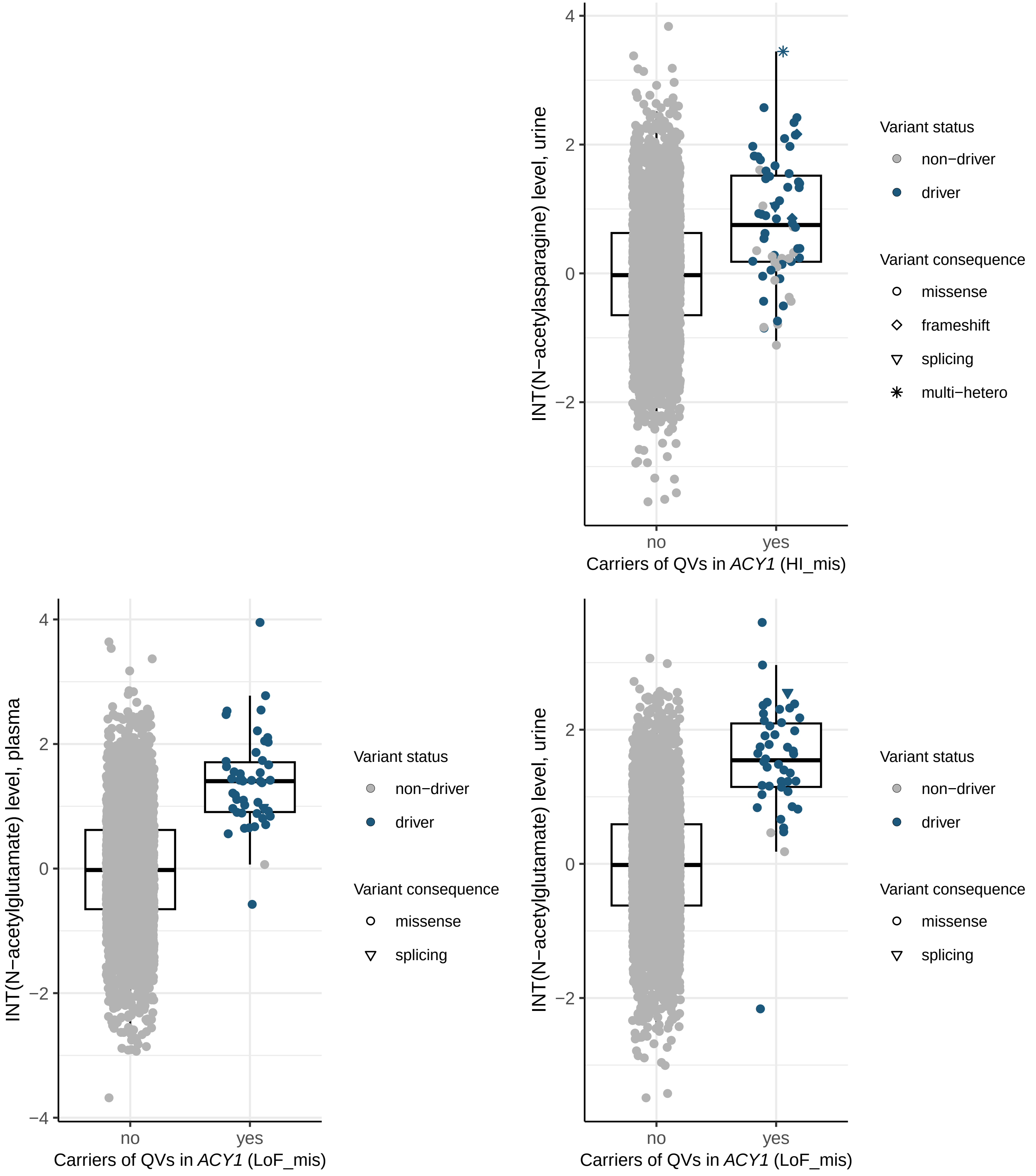
INT(N−acetylasparagine) level, urine
Carriers of QVs in ACY1 (HI_mis)
no
yes
4
2
0
−2
Variant status
non−driver
driver
Variant consequence
missense
frameshift
splicing
multi−hetero
INT(N−acetylglutamate) level, plasma
Carriers of QVs in ACY1 (LoF_mis)
no
yes
4
2
0
−2
−4
Variant status
non−driver
driver
Variant consequence
missense
splicing
INT(N−acetylglutamate) level, urine
Carriers of QVs in ACY1 (LoF_mis)
no
yes
2
0
−2
Variant status
non−driver
driver
Variant consequence
missense
splicing

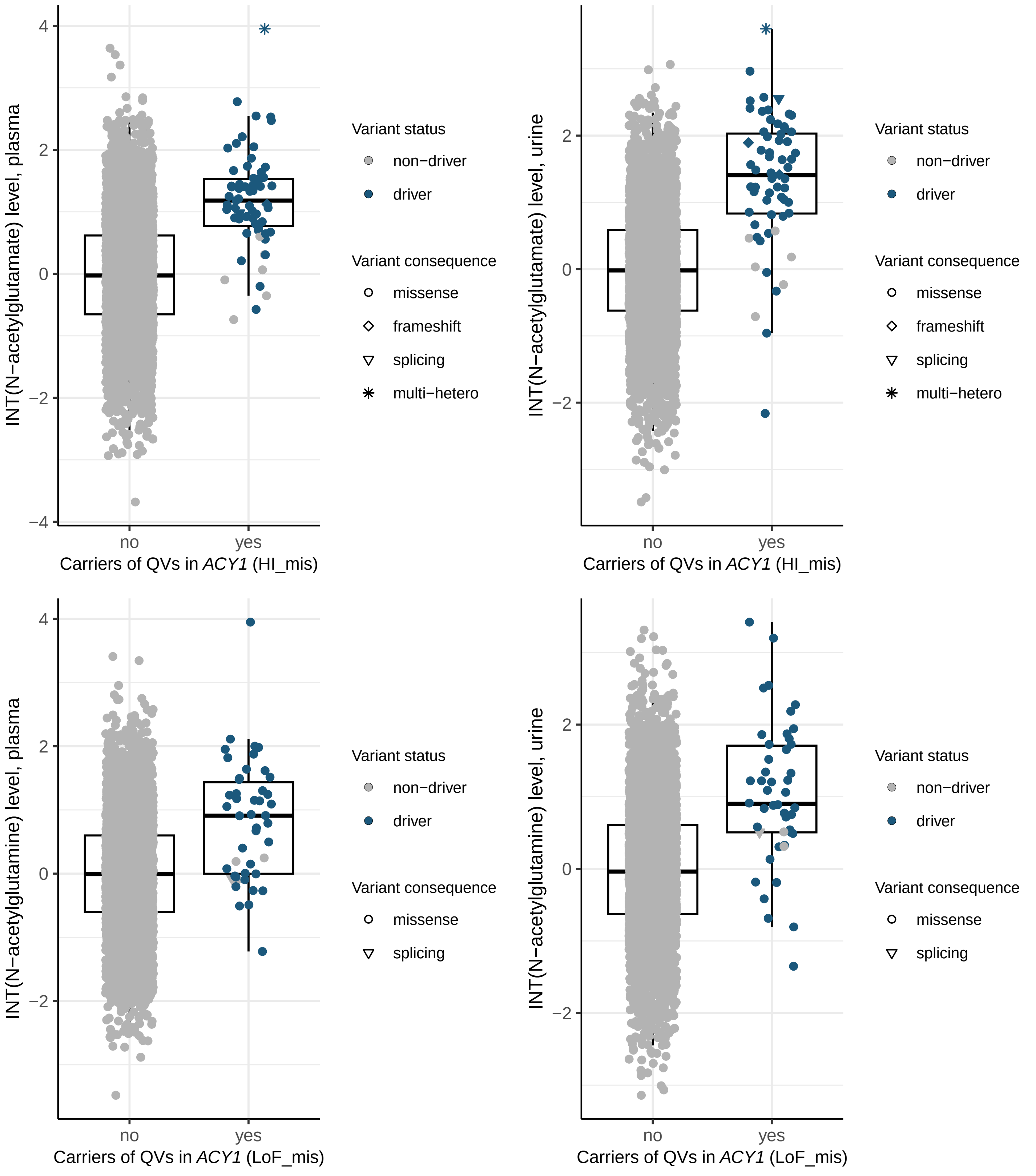
INT(N−acetylglutamate) level, plasma
Carriers of QVs in ACY1 (HI_mis)
INT(N−acetylglutamate) level, urine
Carriers of QVs in ACY1 (HI_mis)
INT(N−acetylglutamine) level, plasma
Carriers of QVs in ACY1 (LoF_mis)
INT(N−acetylglutamine) level, urine
Carriers of QVs in ACY1 (LoF_mis)
no
yes
Variant status
non−driver
driver
Variant consequence
missense
frameshift
splicing
multi−hetero
4
2
0
−2
−4

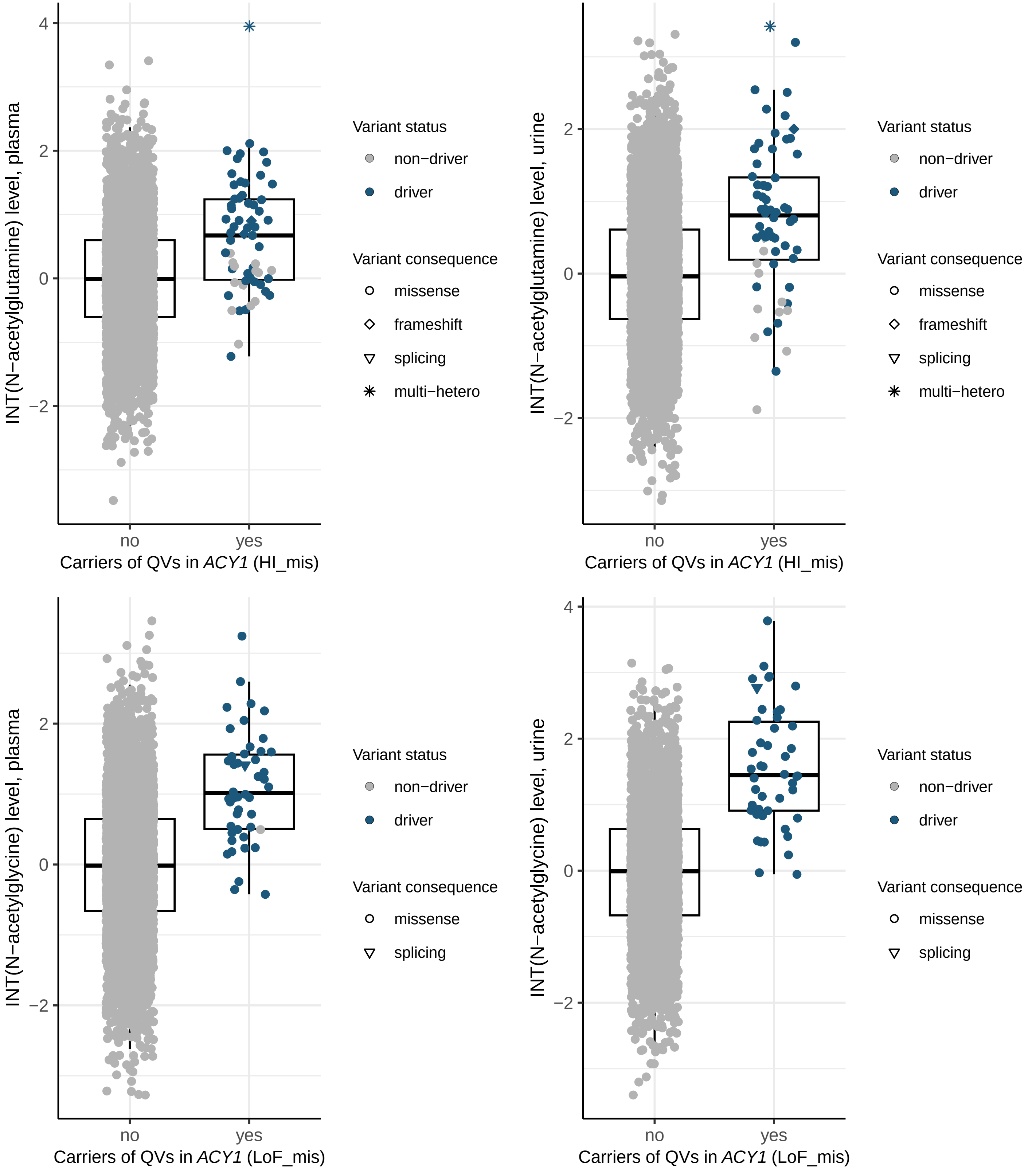
INT(N−acetylglutamine) level, plasma
4
2
0
−2
no
yes
Carriers of QVs in ACY1 (HI_mis)
Variant status
non−driver
driver
Variant consequence
missense
frameshift
splicing
multi−hetero
INT(N−acetylglutamine) level, urine
2
0
−2
no
yes
Carriers of QVs in ACY1 (HI_mis)
Variant status
non−driver
driver
Variant consequence
missense
frameshift
splicing
multi−hetero
INT(N−acetylglycine) level, plasma
2
0
−2
no
yes
Carriers of QVs in ACY1 (LoF_mis)
Variant status
non−driver
driver
Variant consequence
missense
splicing
INT(N−acetylglycine) level, urine
4
2
0
−2
no
yes
Carriers of QVs in ACY1 (LoF_mis)
Variant status
non−driver
driver
Variant consequence
missense
splicing

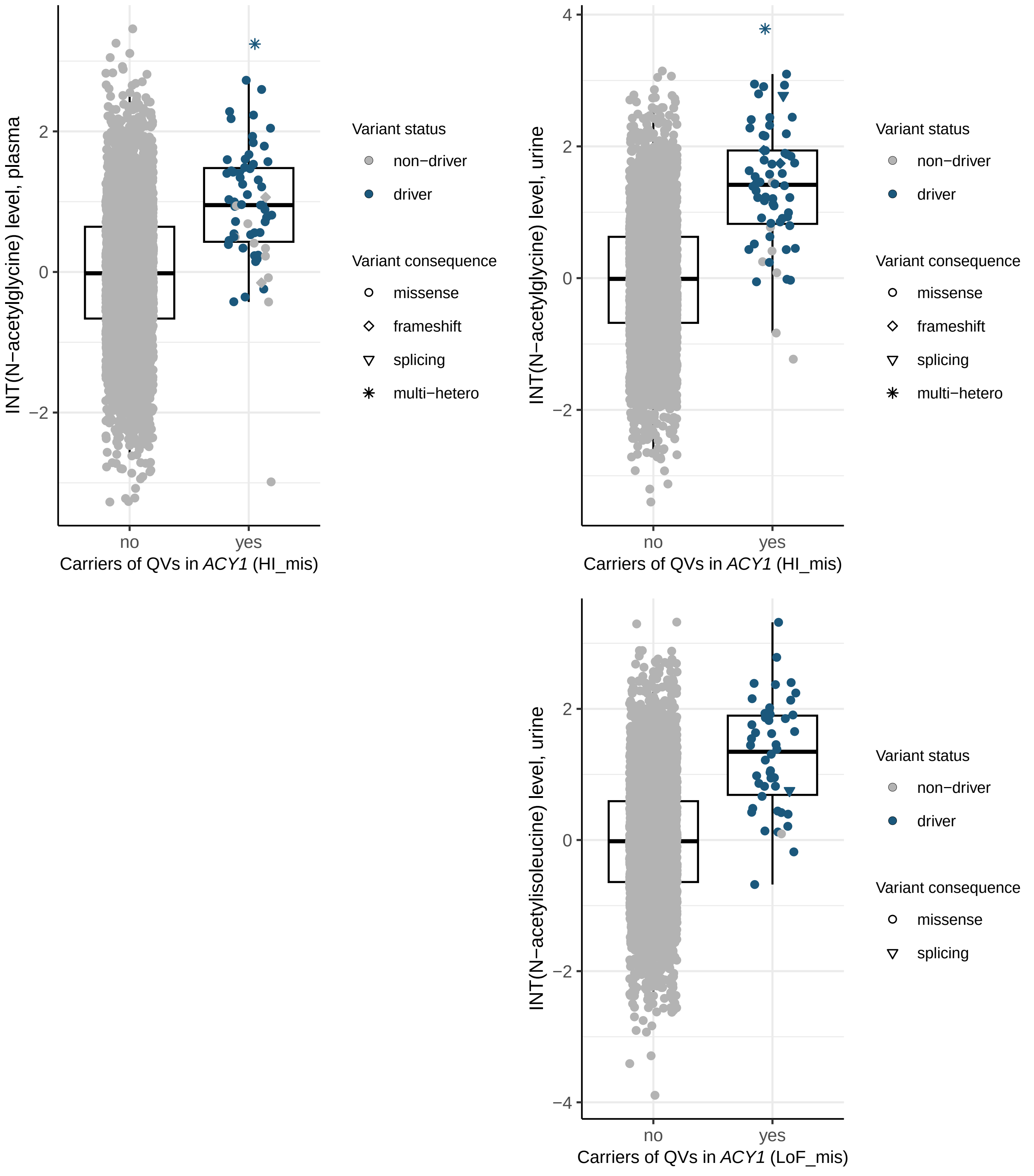
INT(N−acetylglycine) level, plasma
2
0
−2
no
yes
Carriers of QVs in ACY1 (HI_mis)
Variant status
non−driver
driver
Variant consequence
missense
frameshift
splicing
multi−hetero
INT(N−acetylglycine) level, urine
4
2
0
−2
no
yes
Carriers of QVs in ACY1 (HI_mis)
Variant status
non−driver
driver
Variant consequence
missense
frameshift
splicing
multi−hetero
INT(N−acetylisoleucine) level, urine
2
0
−2
−4
no
yes
Carriers of QVs in ACY1 (LoF_mis)
Variant status
non−driver
driver
Variant consequence
missense
splicing

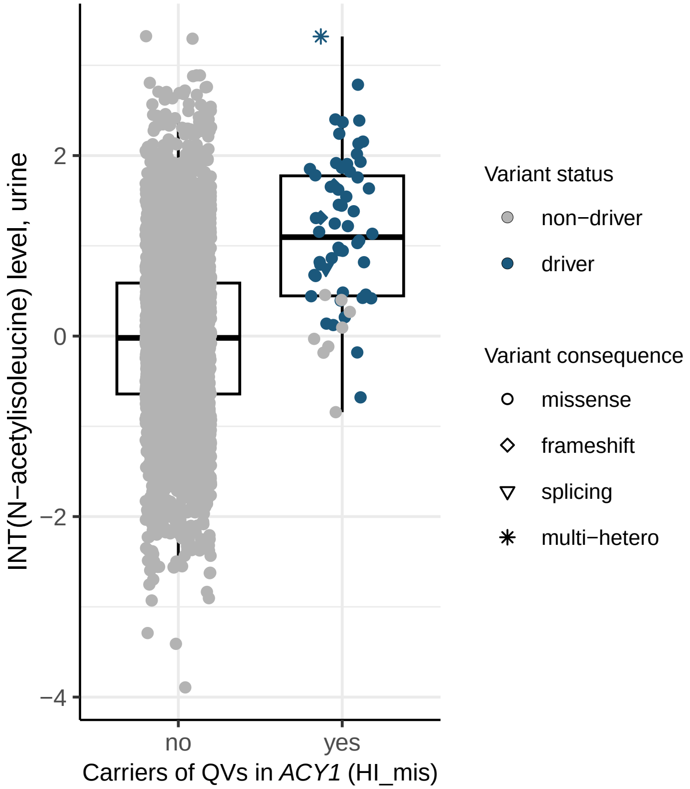
INT(N−acetylisoleucine) level, urine
2
0
−2
−4
no
yes
Carriers of QVs in *ACY1* (HI_mis)
Variant status
non−driver
driver
Variant consequence
missense
frameshift
splicing
multi−hetero

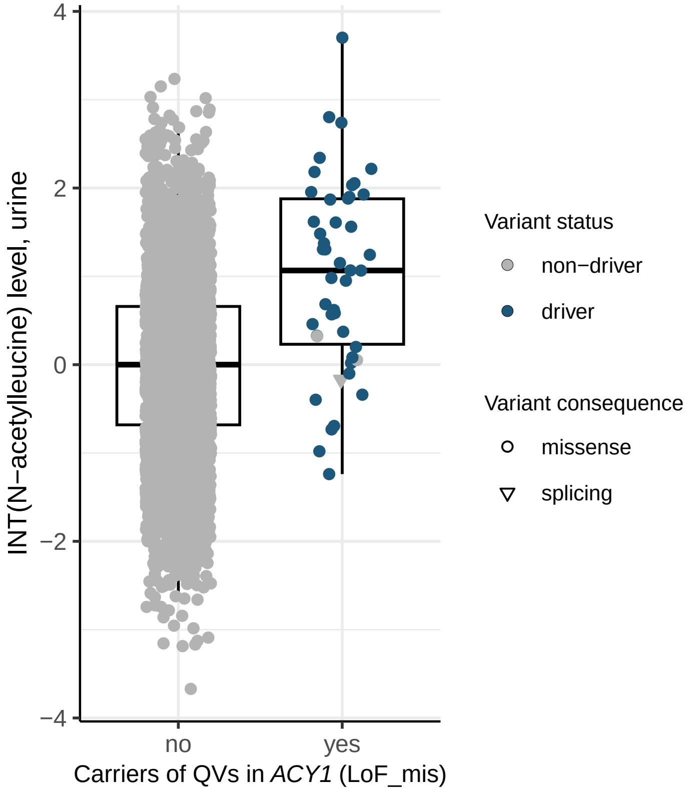
INT(N−acetylleucine) level, urine
4
2
0
−2
−4
no
yes
Carriers of QVs in *ACY1* (LoF_mis)
Variant status
non−driver
driver
Variant consequence
missense
splicing

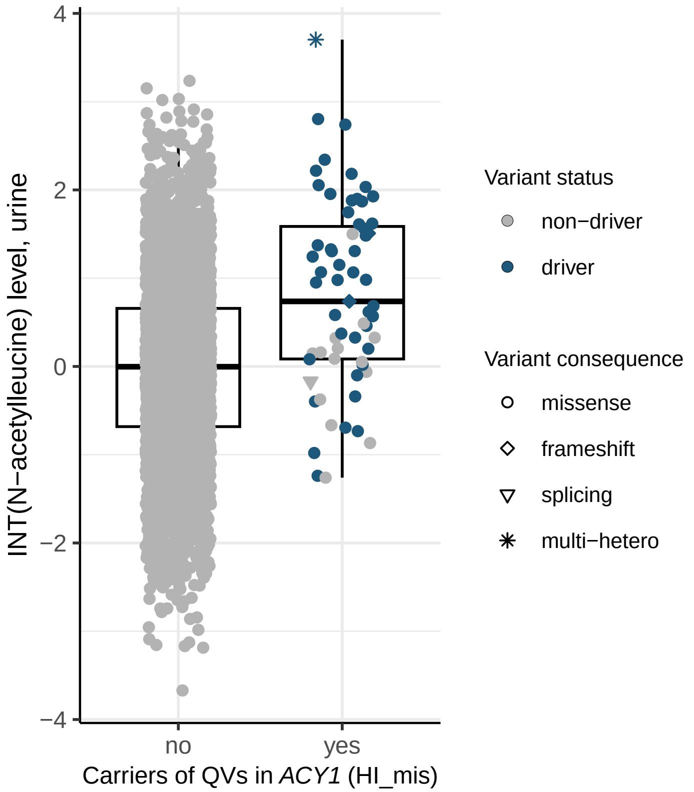
INT(N−acetylleucine) level, urine
4
2
0
−2
−4
no
yes
Carriers of QVs in ACY1 (HI_mis)
Variant status
non−driver
driver
Variant consequence
missense
frameshift
splicing
multi−hetero

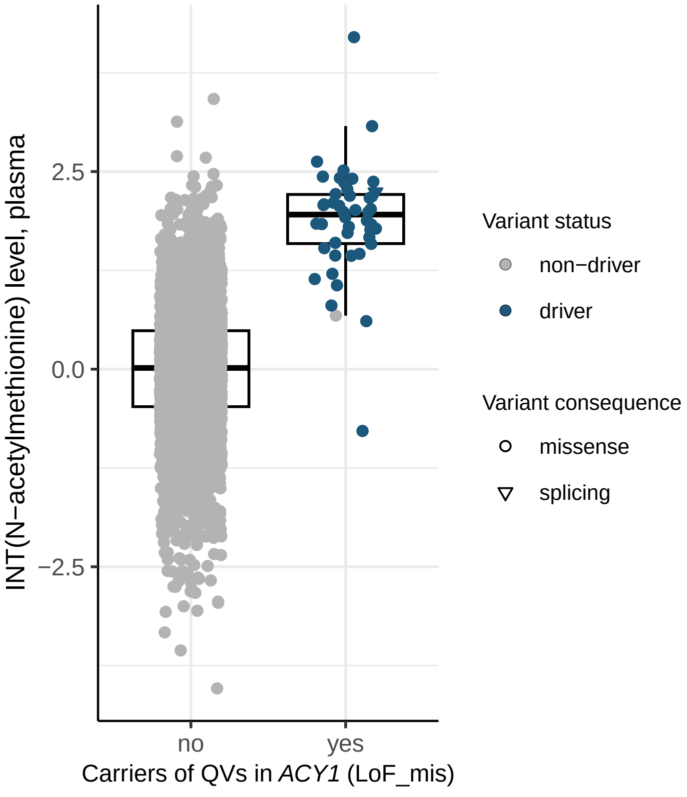
INT(N−acetylmethionine) level, plasma
2.5
0.0
−2.5
no
yes
Carriers of QVs in ACY1 (LoF_mis)
Variant status
non−driver
driver
Variant consequence
missense
splicing

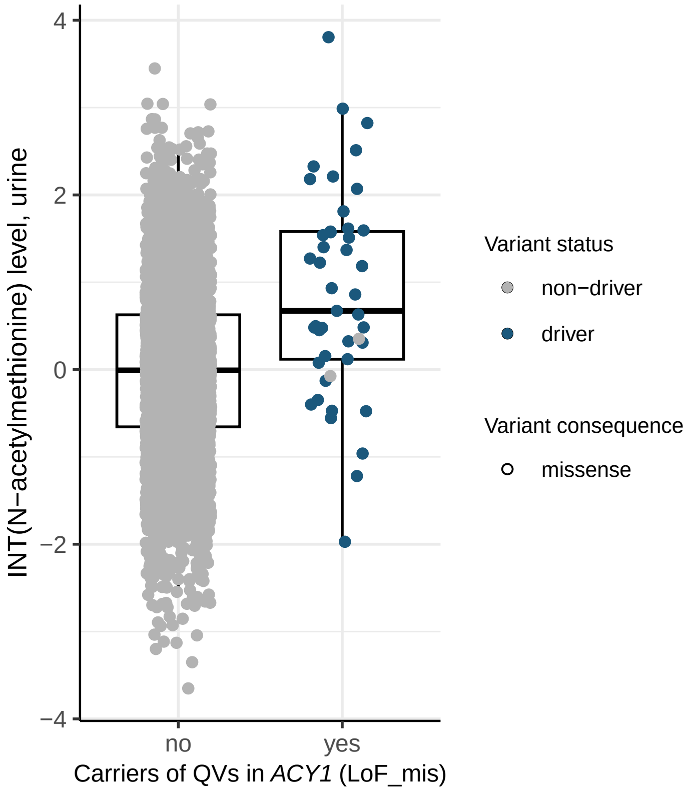
INT(N−acetylmethionine) level, urine
4
2
0
−2
−4
no
yes
Carriers of QVs in ACY1 (LoF_mis)
Variant status
non−driver
driver
Variant consequence
missense

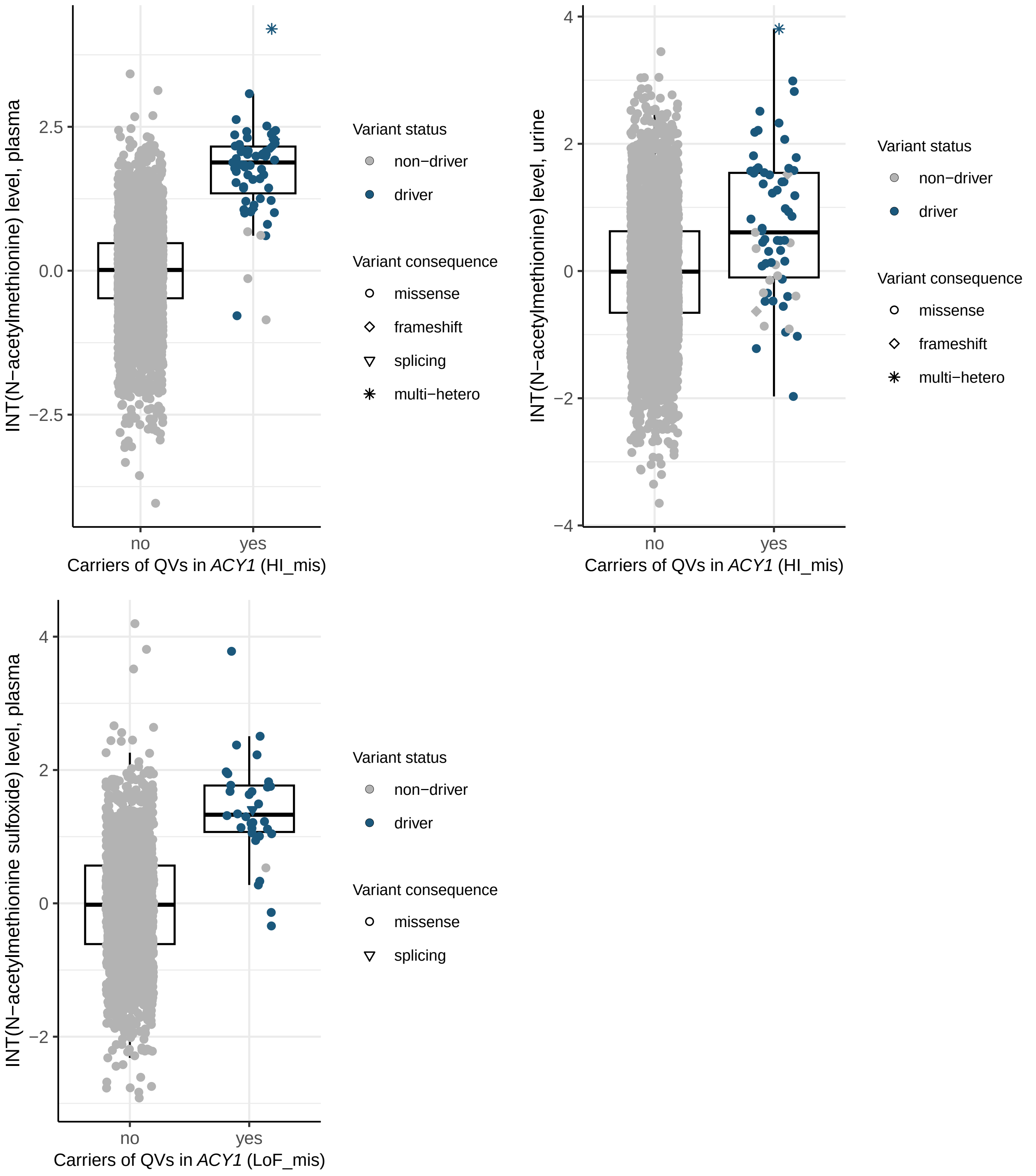
INT(N−acetylmethionine) level, plasma
2.5
0.0
−2.5
no
yes
Carriers of QVs in ACY1 (HI_mis)
Variant status
non−driver
driver
Variant consequence
missense
frameshift
splicing
multi−hetero
INT(N−acetylmethionine) level, urine
4
2
0
−2
−4
no
yes
Carriers of QVs in ACY1 (HI_mis)
Variant status
non−driver
driver
Variant consequence
missense
frameshift
multi−hetero
INT(N−acetylmethionine sulfoxide) level, plasma
4
2
0
−2
no
yes
Carriers of QVs in ACY1 (LoF_mis)
Variant status
non−driver
driver
Variant consequence
missense
splicing

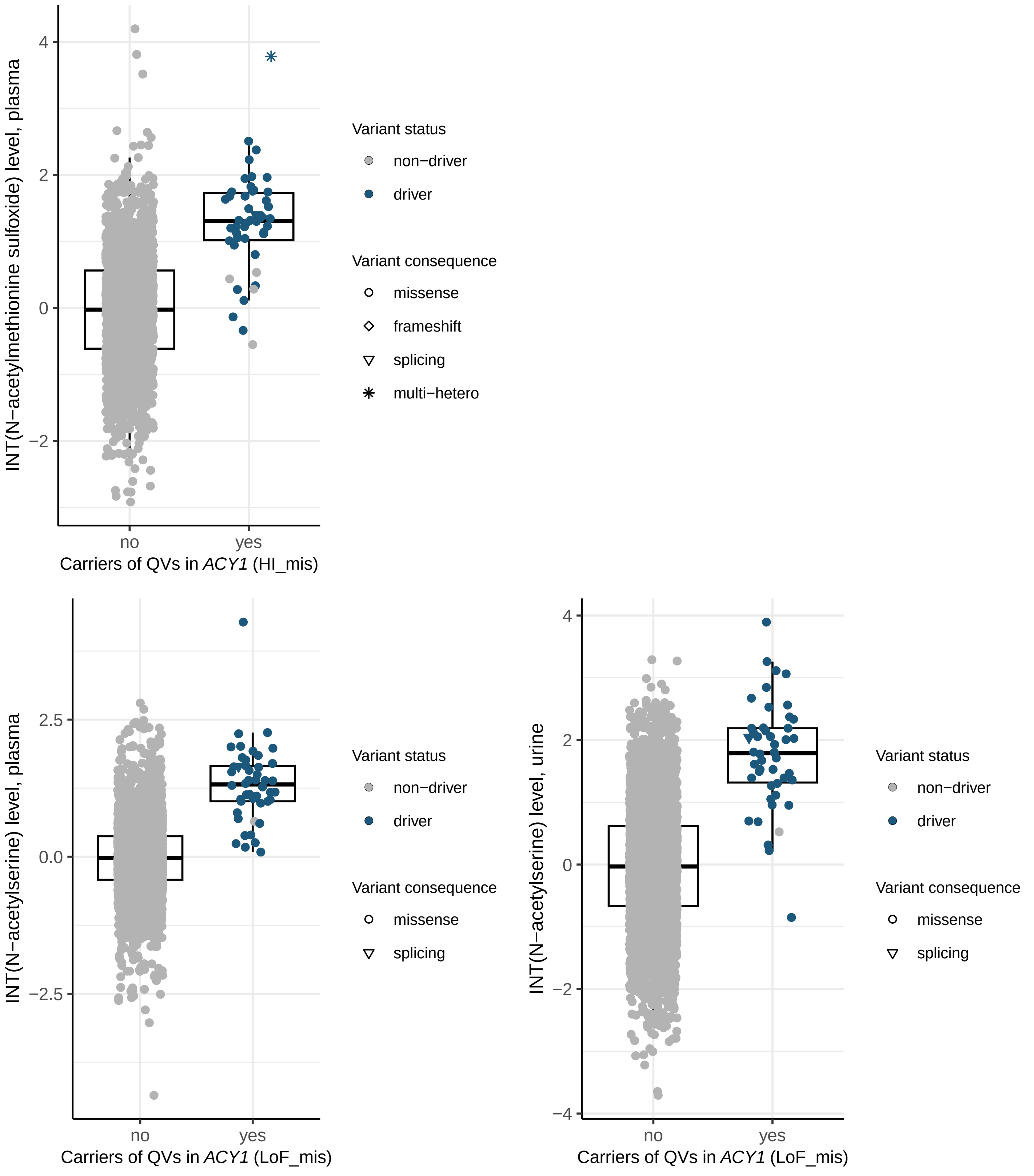
INT(N−acetylmethionine sulfoxide) level, plasma
4
2
0
−2
no
yes
Carriers of QVs in ACY1 (HI_mis)
Variant status
non−driver
driver
Variant consequence
missense
frameshift
splicing
multi−hetero
INT(N−acetylserine) level, plasma
2.5
0.0
−2.5
no
yes
Carriers of QVs in ACY1 (LoF_mis)
Variant status
non−driver
driver
Variant consequence
missense
splicing
INT(N−acetylserine) level, urine
4
2
0
−2
−4
no
yes
Carriers of QVs in ACY1 (LoF_mis)
Variant status
non−driver
driver
Variant consequence
missense
splicing

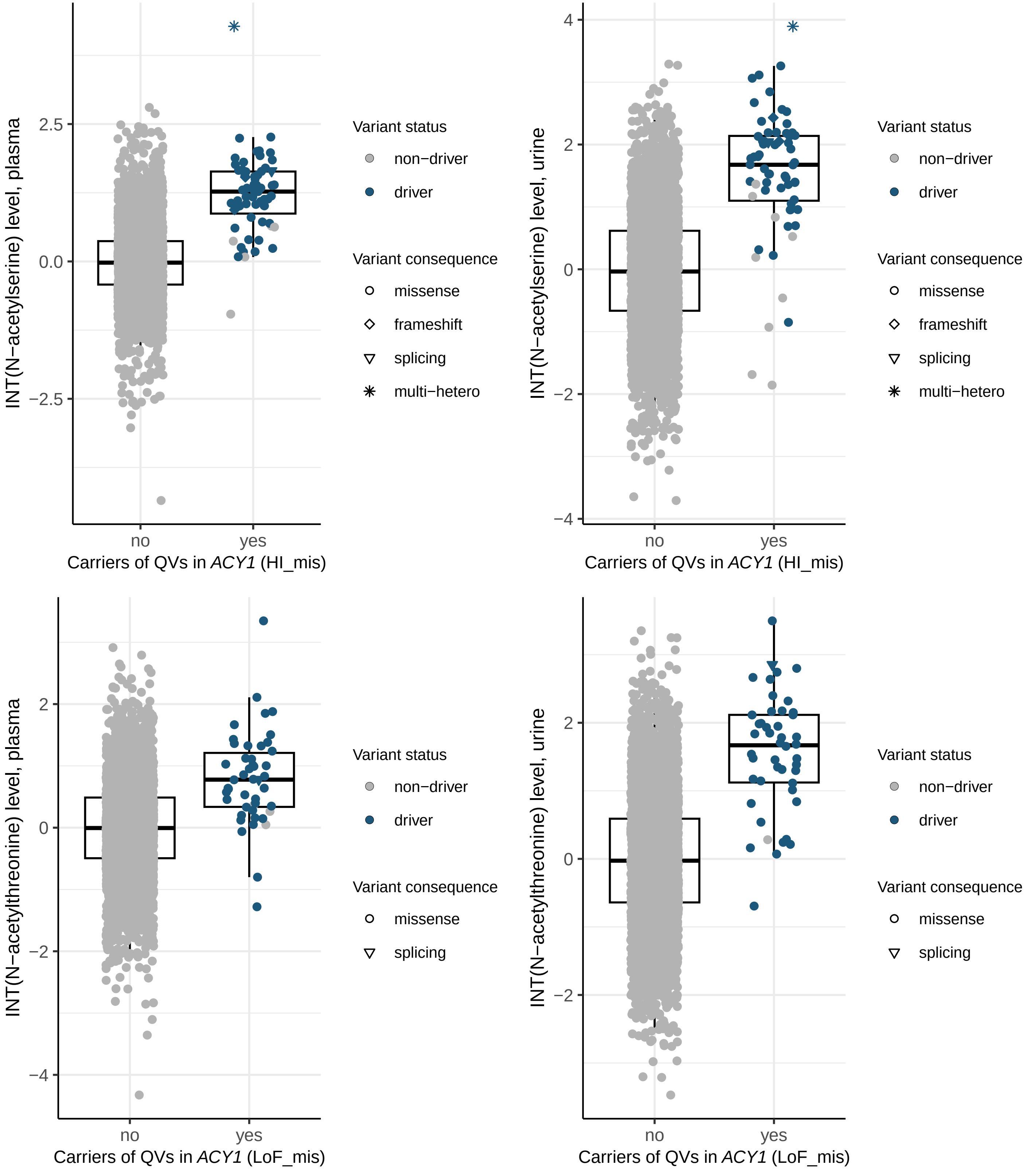
INT(N−acetylserine) level, plasma
2.5
0.0
−2.5
no
yes
Carriers of QVs in ACY1 (HI_mis)
INT(N−acetylserine) level, urine
4
2
0
−2
−4
no
yes
Carriers of QVs in ACY1 (HI_mis)
Variant status
non−driver
driver
Variant consequence
missense
frameshift
splicing
multi−hetero
INT(N−acetylthreonine) level, plasma
2
0
−2
−4
no
yes
Carriers of QVs in ACY1 (LoF_mis)
INT(N−acetylthreonine) level, urine
2
0
−2
no
yes
Carriers of QVs in ACY1 (LoF_mis)
Variant status
non−driver
driver
Variant consequence
missense
splicing

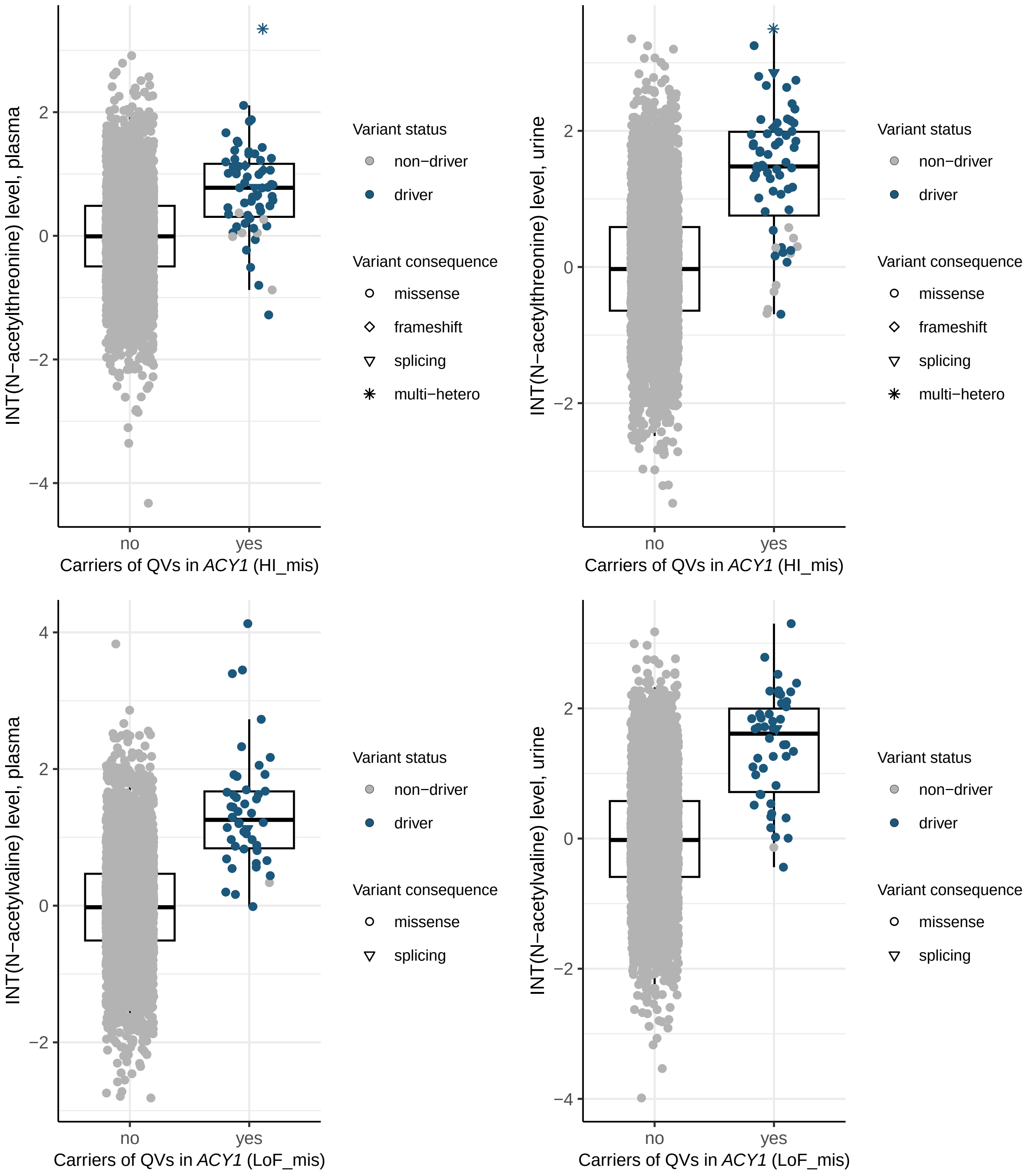
INT(N−acetylthreonine) level, plasma
Carriers of QVs in ACY1 (HI_mis)
no
yes
2
0
−2
−4
Variant status
non−driver
driver
Variant consequence
missense
frameshift
splicing
multi−hetero
INT(N−acetylthreonine) level, urine
Carriers of QVs in ACY1 (HI_mis)
INT(N−acetylvaline) level, plasma
Carriers of QVs in ACY1 (LoF_mis)
4
INT(N−acetylvaline) level, urine
Carriers of QVs in ACY1 (LoF_mis)

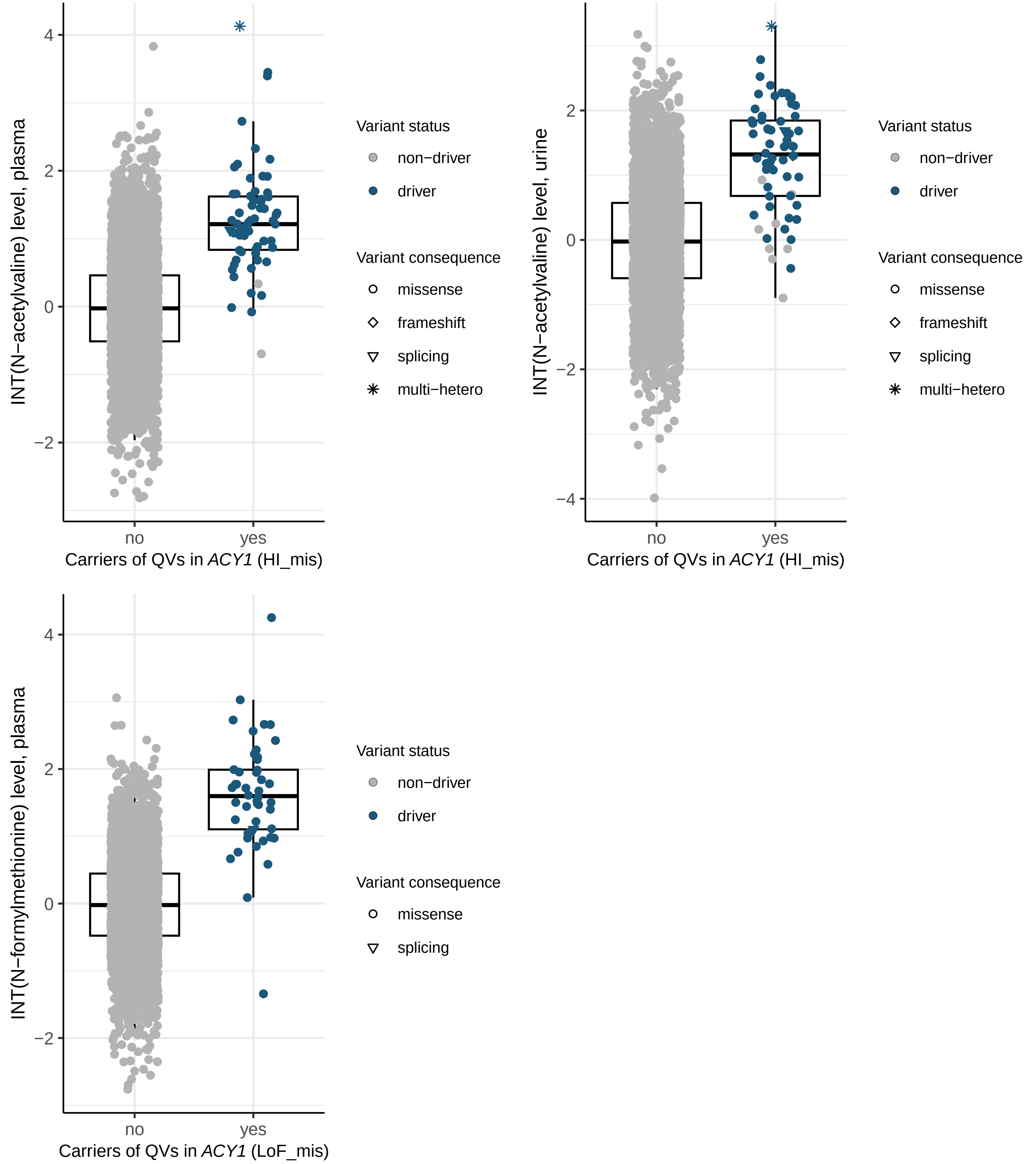
INT(N−acetylvaline) level, plasma
4
2
0
−2
no
yes
Carriers of QVs in ACY1 (HI_mis)
Variant status
non−driver
driver
Variant consequence
missense
frameshift
splicing
multi−hetero
INT(N−acetylvaline) level, urine
2
0
−2
−4
no
yes
Carriers of QVs in ACY1 (HI_mis)
Variant status
non−driver
driver
Variant consequence
missense
frameshift
splicing
multi−hetero
INT(N−formylmethionine) level, plasma
4
2
0
−2
no
yes
Carriers of QVs in ACY1 (LoF_mis)
Variant status
non−driver
driver
Variant consequence
missense
splicing

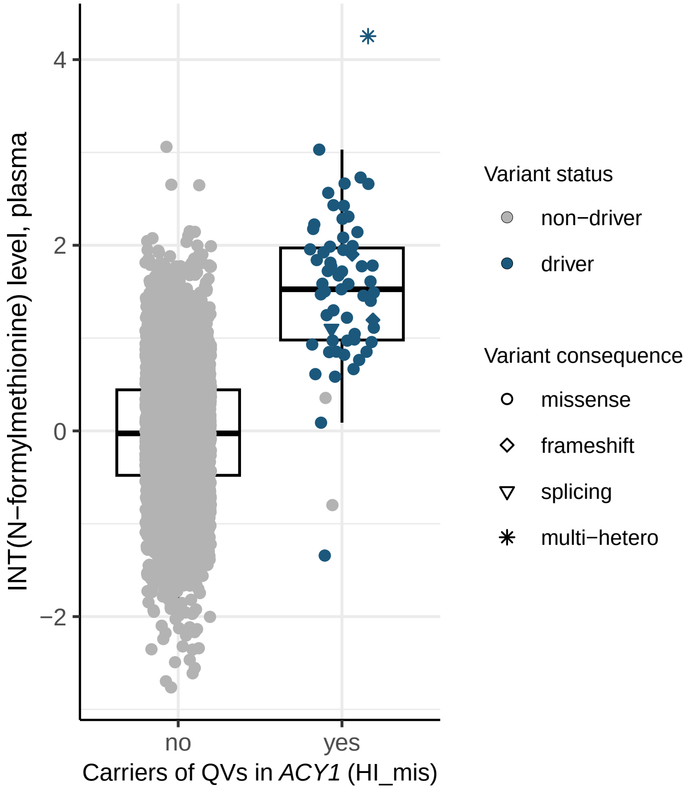

INT(N−formylmethionine) level, plasma
4
2
0
−2
no
yes
Carriers of QVs in *ACY1* (HI_mis)
Variant status
non−driver
driver
Variant consequence
missense
frameshift
splicing
multi−hetero

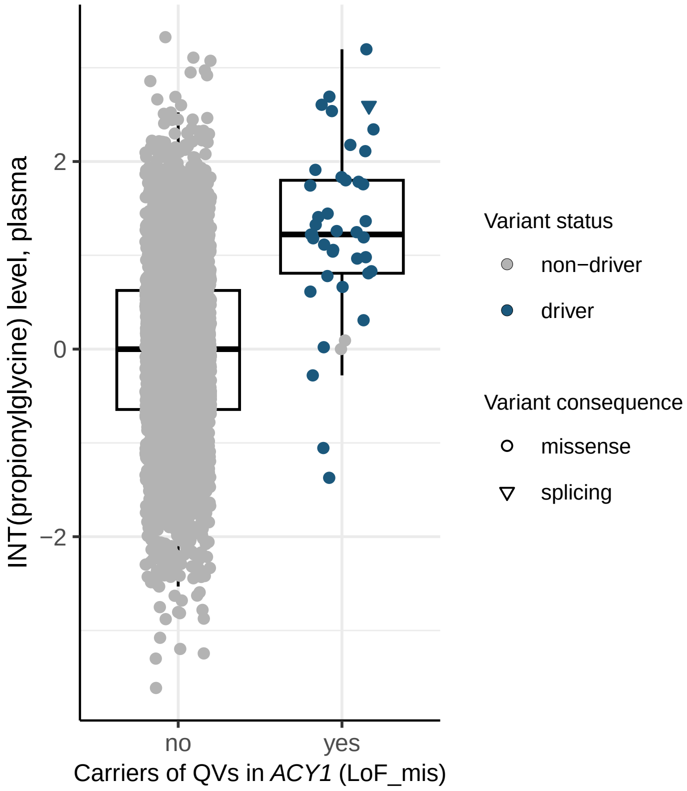

INT(propionylglycine) level, plasma
2
0
−2
no
yes
Carriers of QVs in *ACY1* (LoF_mis)
Variant status
non−driver
driver
Variant consequence
missense
splicing

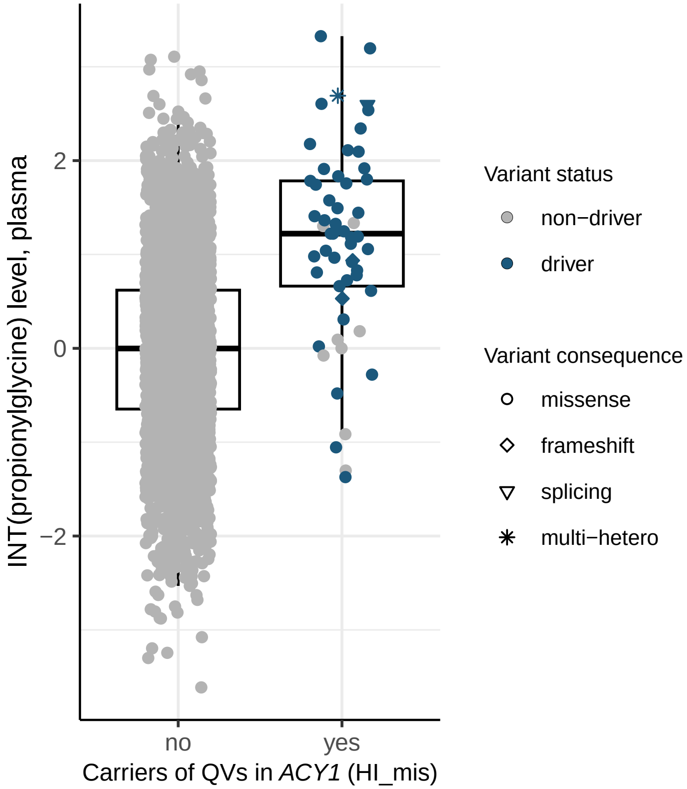
INT(propionylglycine) level, plasma
2
0
−2
no
yes
Carriers of QVs in *ACY1* (HI_mis)
Variant status
non−driver
driver
Variant consequence
missense
frameshift
splicing
multi−hetero

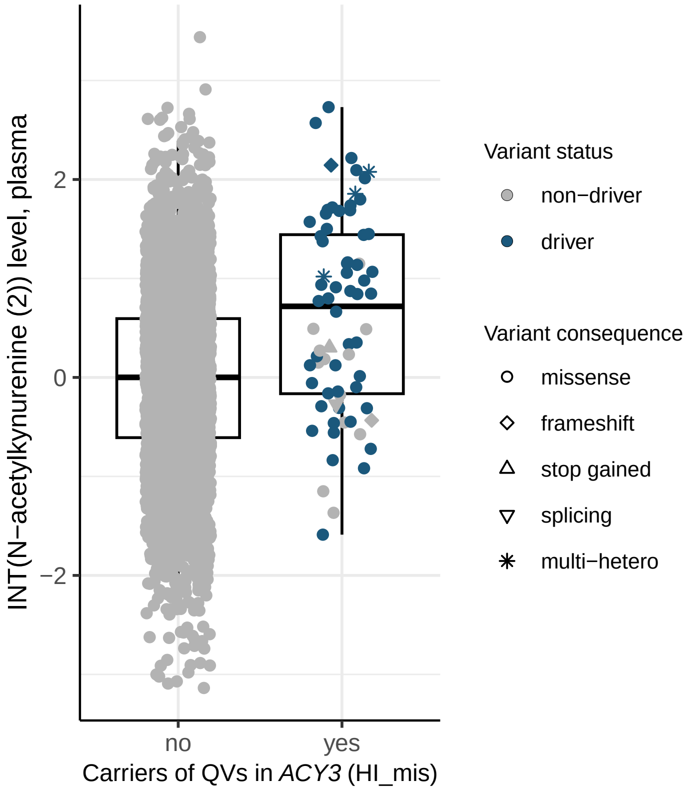
INT(N−acetylkynurenine (2)) level, plasma
2
0
−2
no
yes
Carriers of QVs in *ACY3* (HI_mis)
Variant status
non−driver
driver
Variant consequence
missense
frameshift
stop gained
splicing
multi−hetero

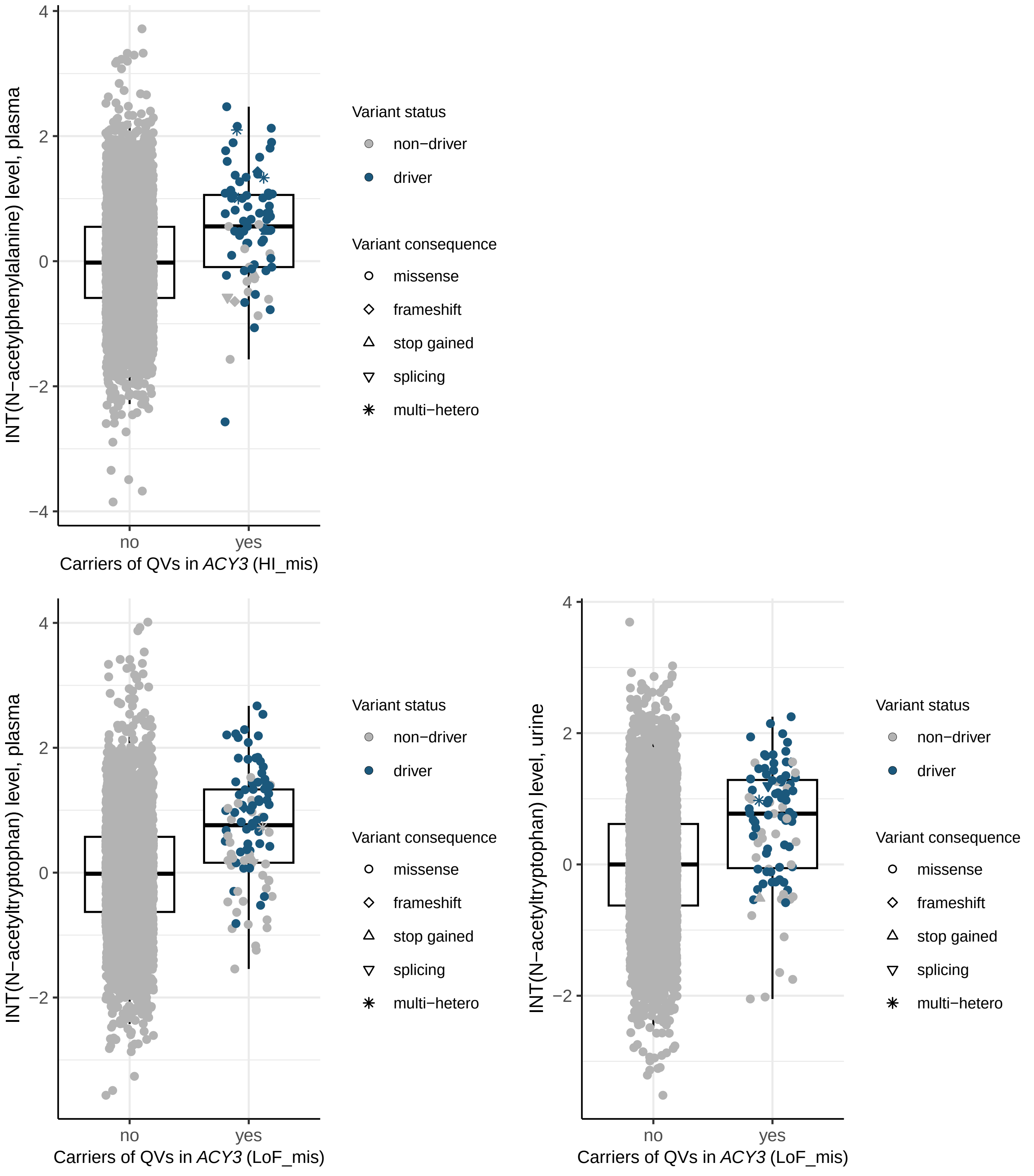

INT(N−acetylphenylalanine) level, plasma
Carriers of QVs in ACY3 (HI_mis)
INT(N−acetyltryptophan) level, plasma
Carriers of QVs in ACY3 (LoF_mis)
INT(N−acetyltryptophan) level, urine
Carriers of QVs in ACY3 (LoF_mis)
no
yes
Variant status
non−driver
driver
Variant consequence
missense
frameshift
stop gained
splicing
multi−hetero

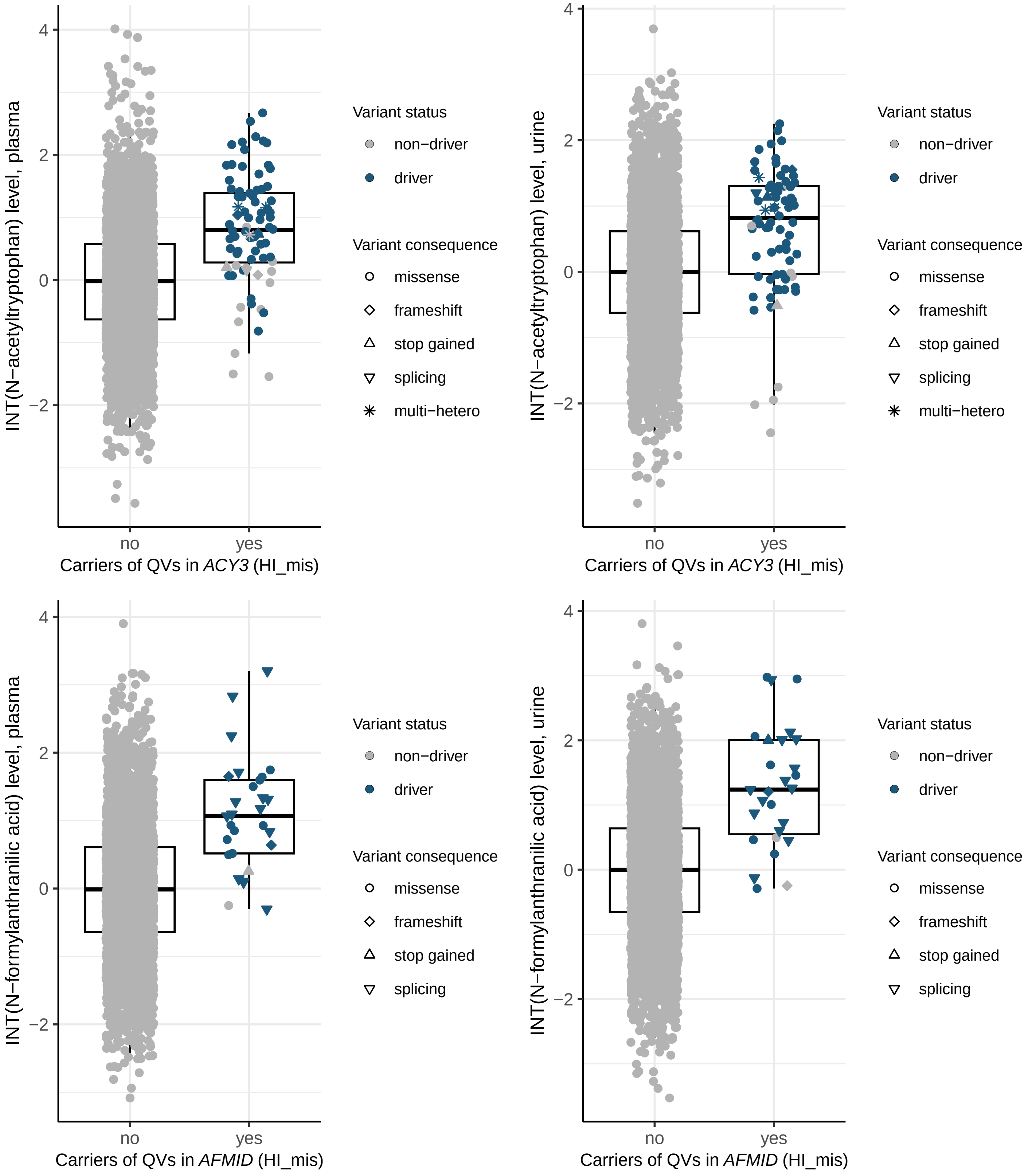

INT(N−acetyltryptophan) level, plasma
Carriers of QVs in ACY3 (HI_mis)
INT(N−acetyltryptophan) level, urine
Carriers of QVs in ACY3 (HI_mis)
INT(N−formylanthranilic acid) level, plasma
Carriers of QVs in AFMID (HI_mis)
INT(N−formylanthranilic acid) level, urine
Carriers of QVs in AFMID (HI_mis)
no
yes
4
2
0
−2
Variant status
non−driver
driver
Variant consequence
missense
frameshift
stop gained
splicing
multi−hetero

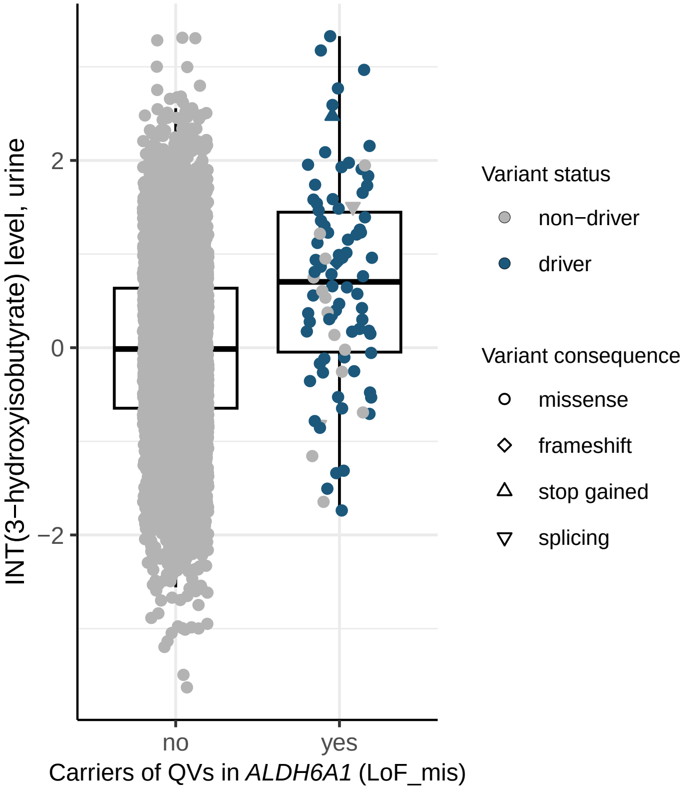
INT(3−hydroxyisobutyrate) level, urine
2
0
−2
no
yes
Carriers of QVs in *ALDH6A1* (LoF_mis)
Variant status
non−driver
driver
Variant consequence
missense
frameshift
stop gained
splicing

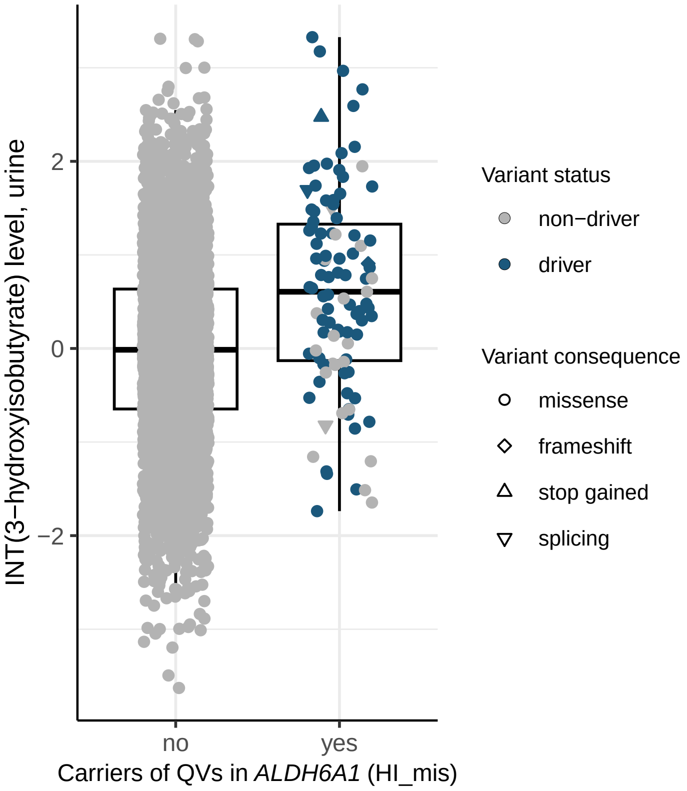
INT(3−hydroxyisobutyrate) level, urine
2
0
−2
no
yes
Carriers of QVs in *ALDH6A1* (HI_mis)
Variant status
non−driver
driver
Variant consequence
missense
frameshift
stop gained
splicing

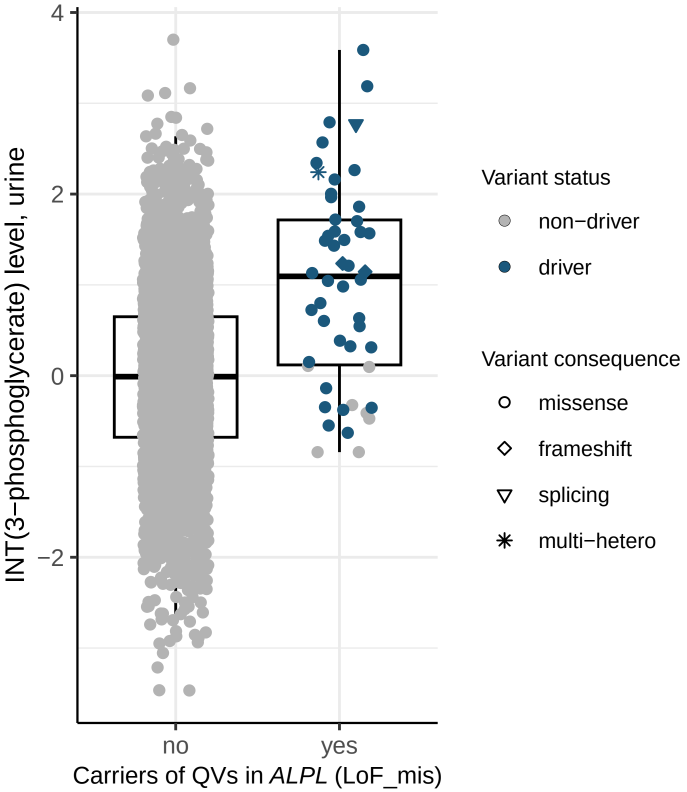

INT(3−phosphoglycerate) level, urine
4
2
0
−2
no
yes
Carriers of QVs in *ALPL* (LoF_mis)
Variant status
non−driver
driver
Variant consequence
missense
frameshift
splicing
multi−hetero

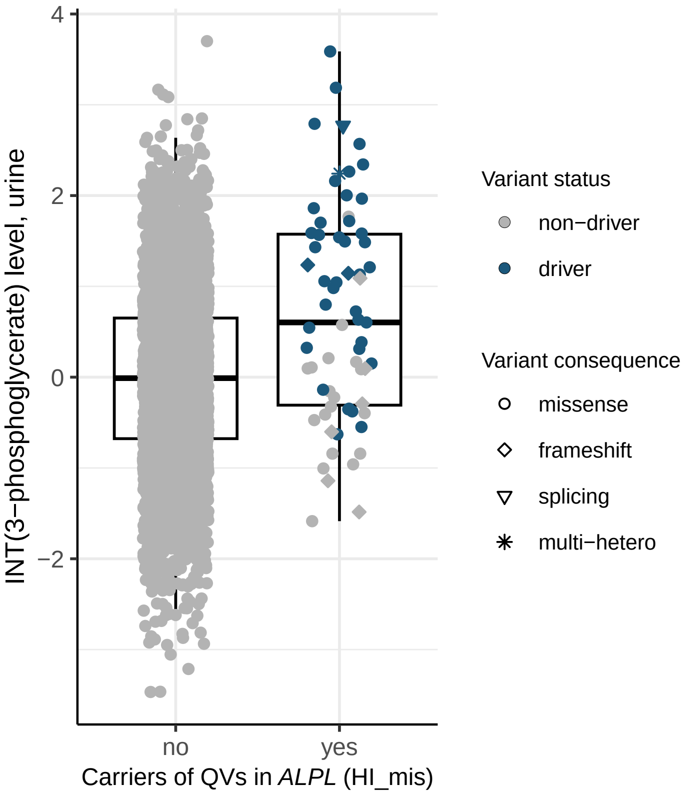

INT(3−phosphoglycerate) level, urine
4
2
0
−2
no
yes
Carriers of QVs in *ALPL* (HI_mis)
Variant status
non−driver
driver
Variant consequence
missense
frameshift
splicing
multi−hetero

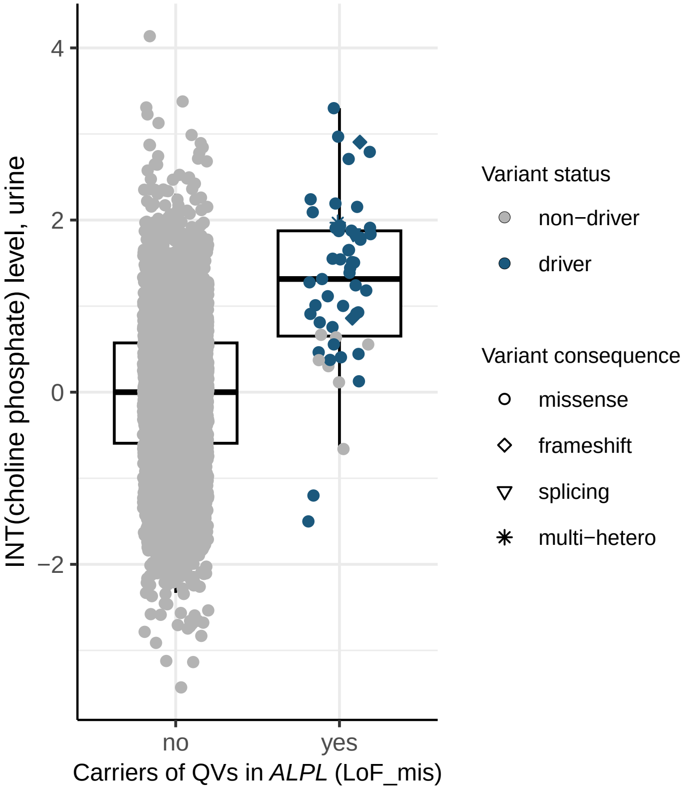

4
2
0
−2
INT(choline phosphate) level, urine
no
yes
Carriers of QVs in ALPL (LoF_mis)
Variant status
non−driver
driver
Variant consequence
missense
frameshift
splicing
multi−hetero

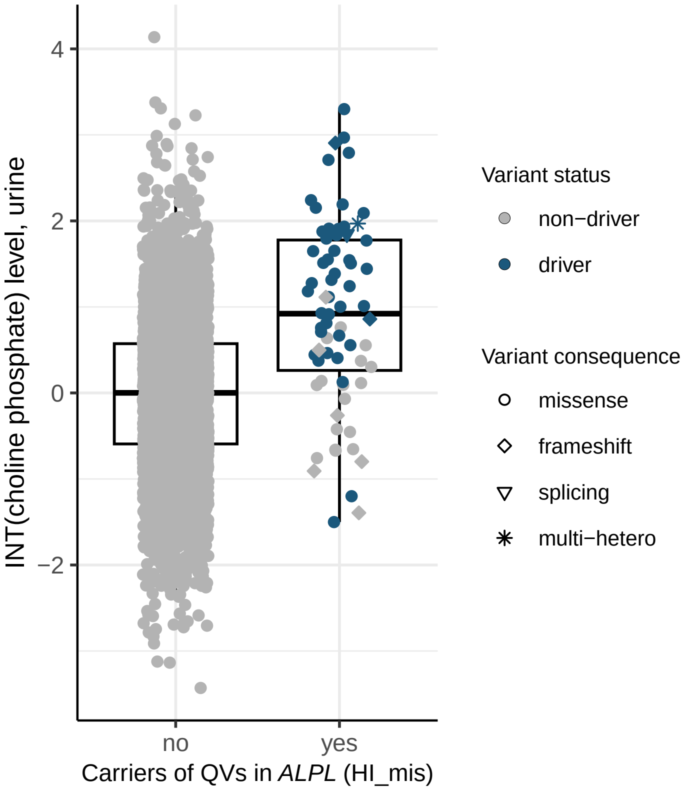

4
2
0
−2
INT(choline phosphate) level, urine
no
yes
Carriers of QVs in ALPL (HI_mis)
Variant status
non−driver
driver
Variant consequence
missense
frameshift
splicing
multi−hetero

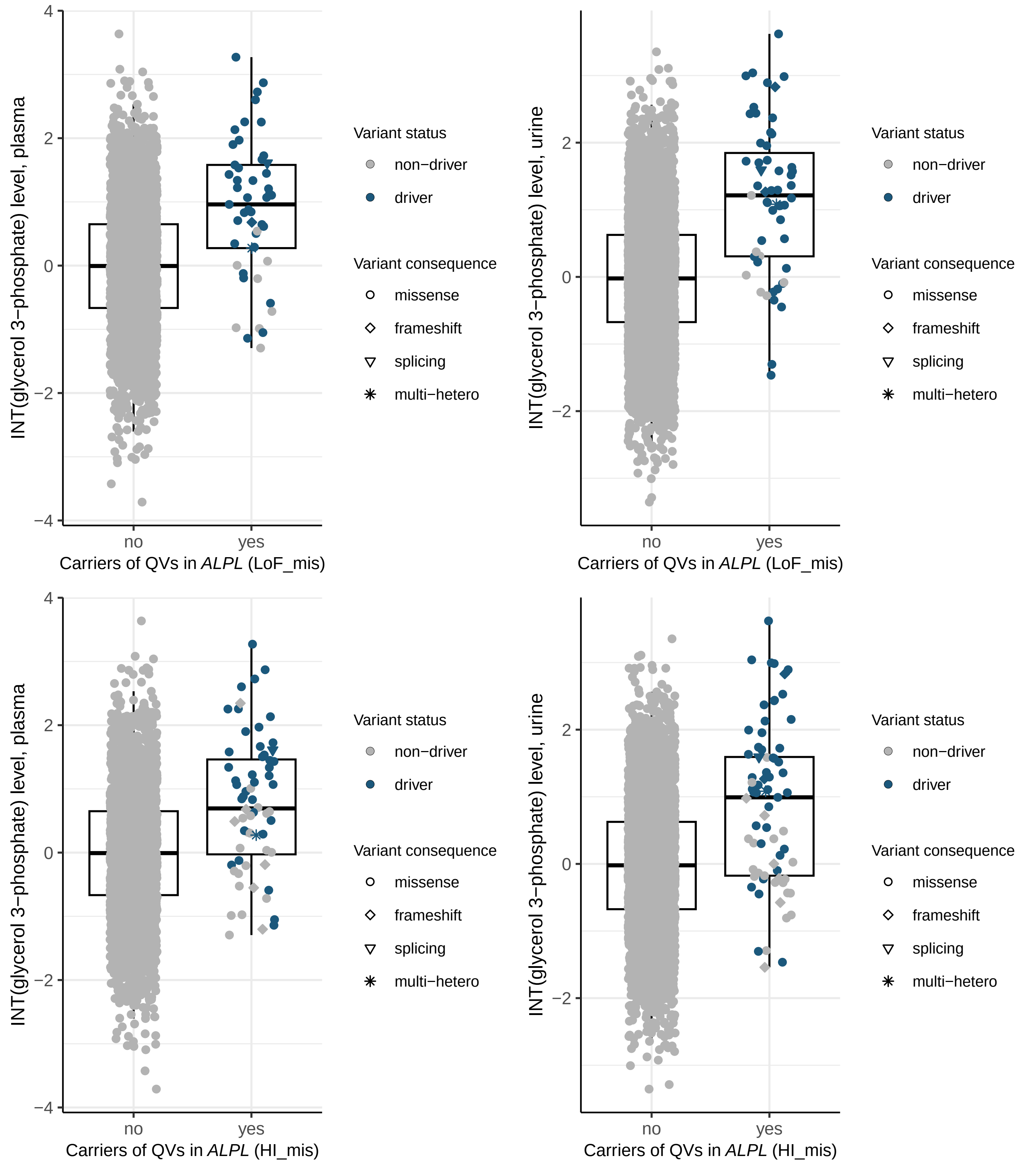
INT(glycerol 3−phosphate) level, plasma
INT(glycerol 3−phosphate) level, urine
Carriers of QVs in ALPL (LoF_mis)
Carriers of QVs in ALPL (HI_mis)
no
yes
4
2
0
−2
−4
Variant status
non−driver
driver
Variant consequence
missense
frameshift
splicing
multi−hetero

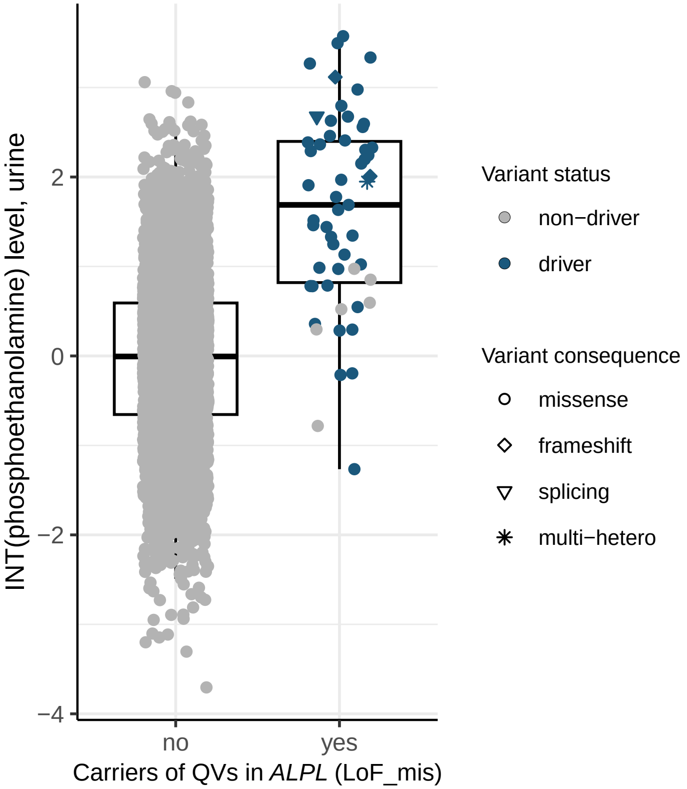
INT(phosphoethanolamine) level, urine
2
0
−2
−4
no
yes
Carriers of QVs in ALPL (LoF_mis)
Variant status
non−driver
driver
Variant consequence
missense
frameshift
splicing
multi−hetero

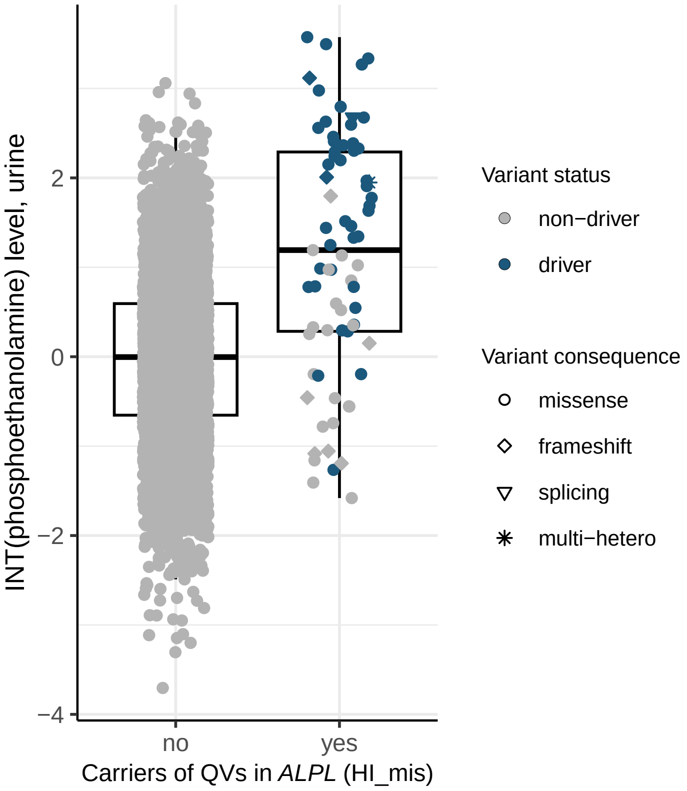
INT(phosphoethanolamine) level, urine
2
0
−2
−4
no
yes
Carriers of QVs in ALPL (HI_mis)
Variant status
non−driver
driver
Variant consequence
missense
frameshift
splicing
multi−hetero

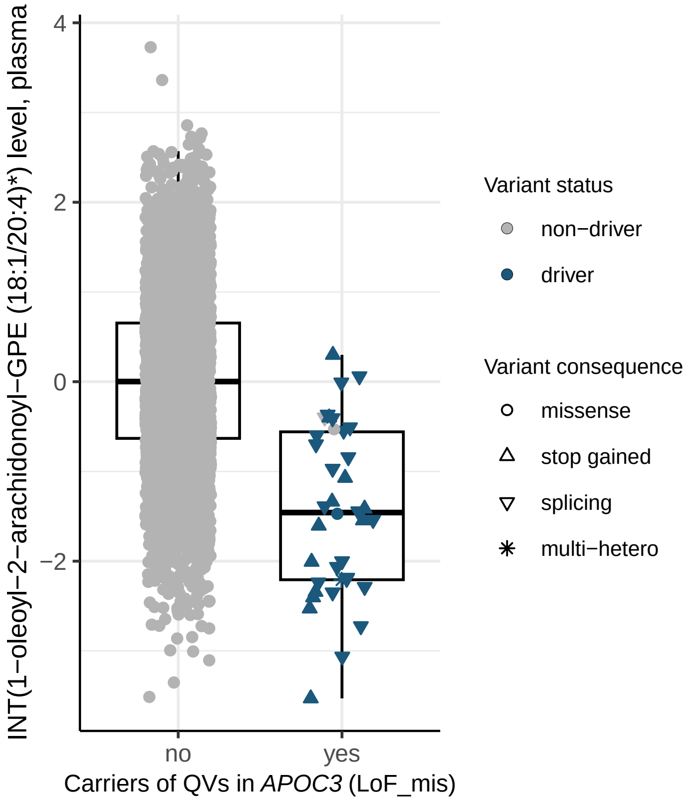
INT(1−oleoyl−2−arachidonoyl−GPE (18:1/20:4)*) level, plasma
4
2
0
−2
no
yes
Carriers of QVs in APOC3 (LoF_mis)
Variant status
non−driver
driver
Variant consequence
missense
stop gained
splicing
multi−hetero

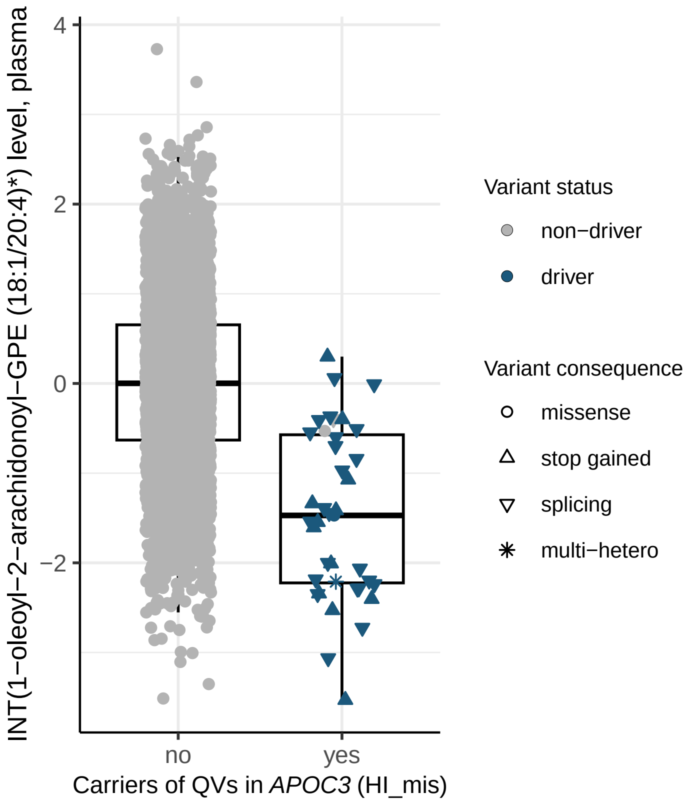
INT(1−oleoyl−2−arachidonoyl−GPE (18:1/20:4)*) level, plasma
4
2
0
−2
no
yes
Carriers of QVs in APOC3 (HI_mis)
Variant status
non−driver
driver
Variant consequence
missense
stop gained
splicing
multi−hetero

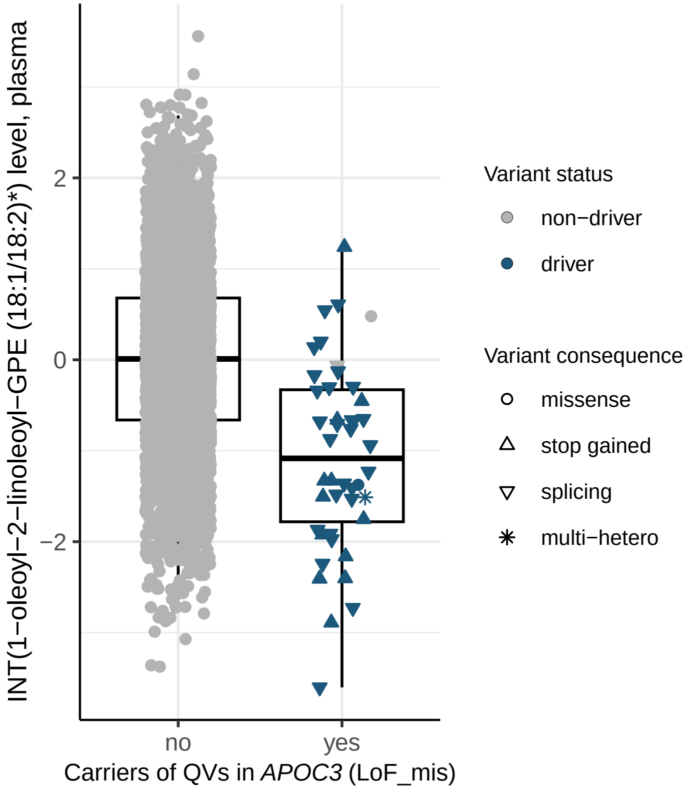
INT(1−oleoyl−2−linoleoyl−GPE (18:1/18:2)*) level, plasma
2
0
−2
no
yes
Carriers of QVs in APOC3 (LoF_mis)
Variant status
non−driver
driver
Variant consequence
missense
stop gained
splicing
multi−hetero

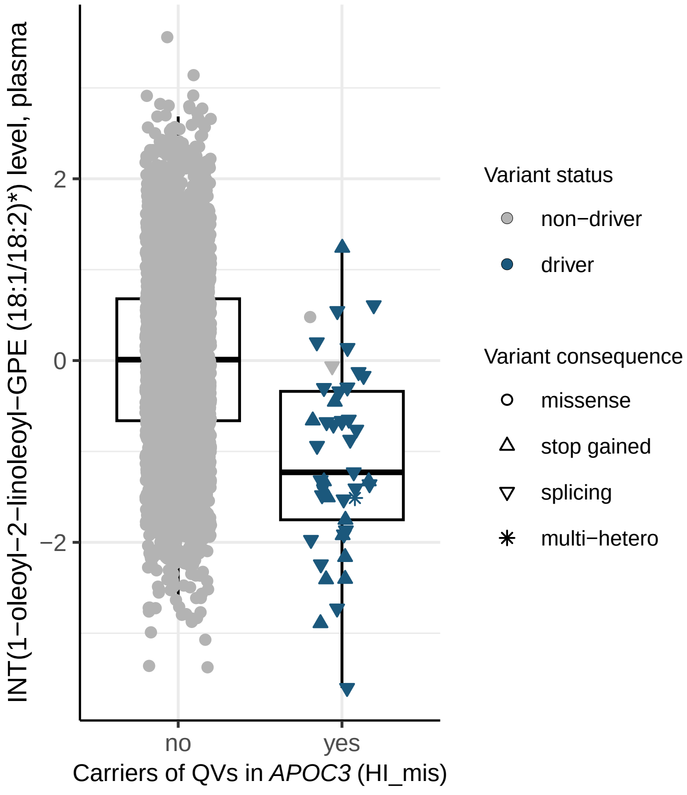
INT(1−oleoyl−2−linoleoyl−GPE (18:1/18:2)*) level, plasma
2
0
−2
no
yes
Carriers of QVs in APOC3 (HI_mis)
Variant status
non−driver
driver
Variant consequence
missense
stop gained
splicing
multi−hetero

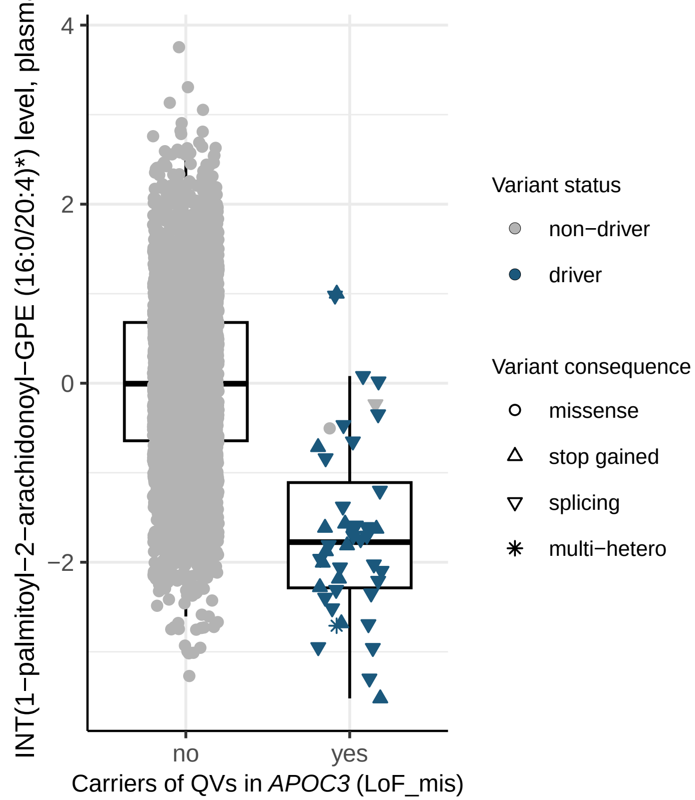
INT(1−palmitoyl−2−arachidonoyl−GPE (16:0/20:4)*) level, plasm
4
2
0
−2
no
yes
Carriers of QVs in *APOC3* (LoF_mis)
Variant status
non−driver
driver
Variant consequence
missense
stop gained
splicing
multi−hetero

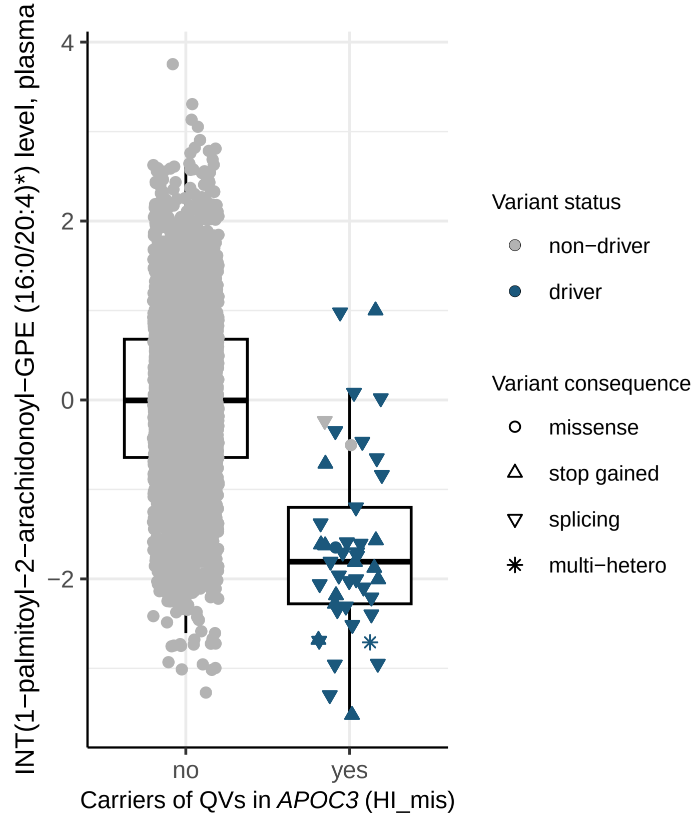
INT(1−palmitoyl−2−arachidonoyl−GPE (16:0/20:4)*) level, plasma
4
2
0
−2
no
yes
Carriers of QVs in *APOC3* (HI_mis)
Variant status
non−driver
driver
Variant consequence
missense
stop gained
splicing
multi−hetero

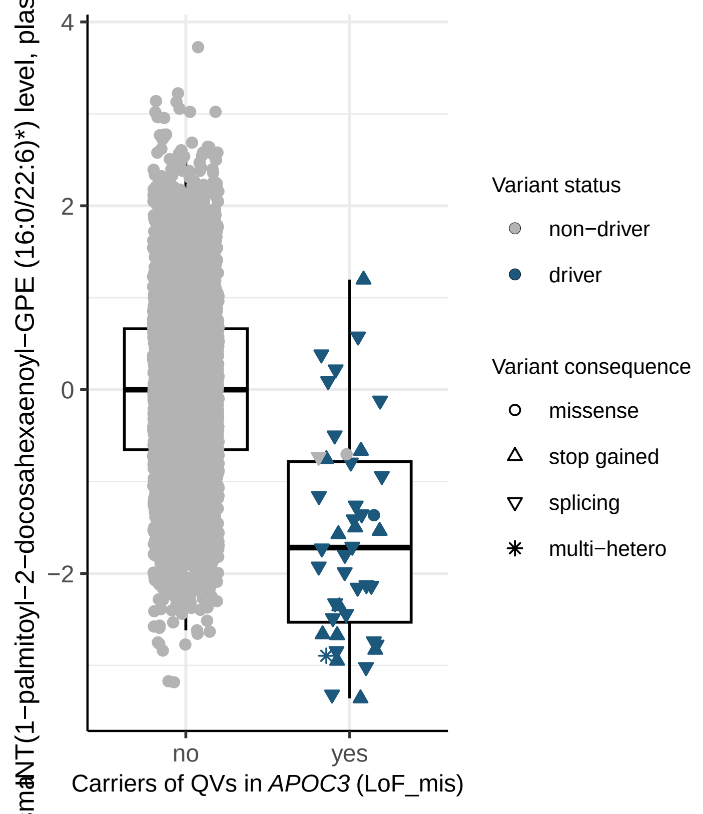
INT(1−palmitoyl−2−docosahexaenoyl−GPE (16:0/22:6)*) level, plas
4
2
0
−2
no
yes
Carriers of QVs in *APOC3* (LoF_mis)
Variant status
non−driver
driver
Variant consequence
missense
stop gained
splicing
multi−hetero

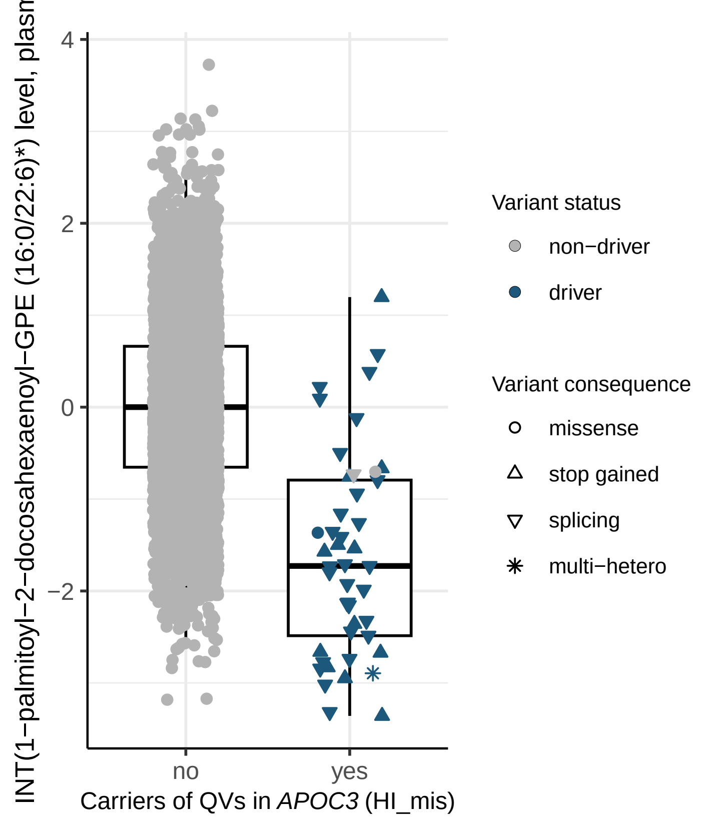
INT(1−palmitoyl−2−docosahexaenoyl−GPE (16:0/22:6)*) level, plasma
4
2
0
−2
no
yes
Carriers of QVs in *APOC3* (HI_mis)
Variant status
non−driver
driver
Variant consequence
missense
stop gained
splicing
multi−hetero

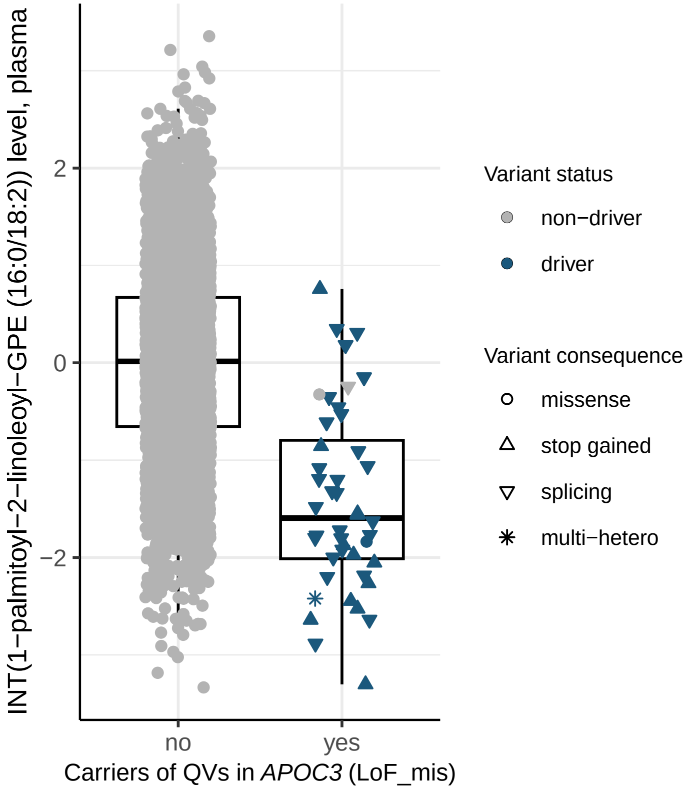

INT(1−palmitoyl−2−linoleoyl−GPE (16:0/18:2)) level, plasma
2
0
−2
no
yes
Carriers of QVs in *APOC3* (LoF_mis)
Variant status
non−driver
driver
Variant consequence
missense
stop gained
splicing
multi−hetero

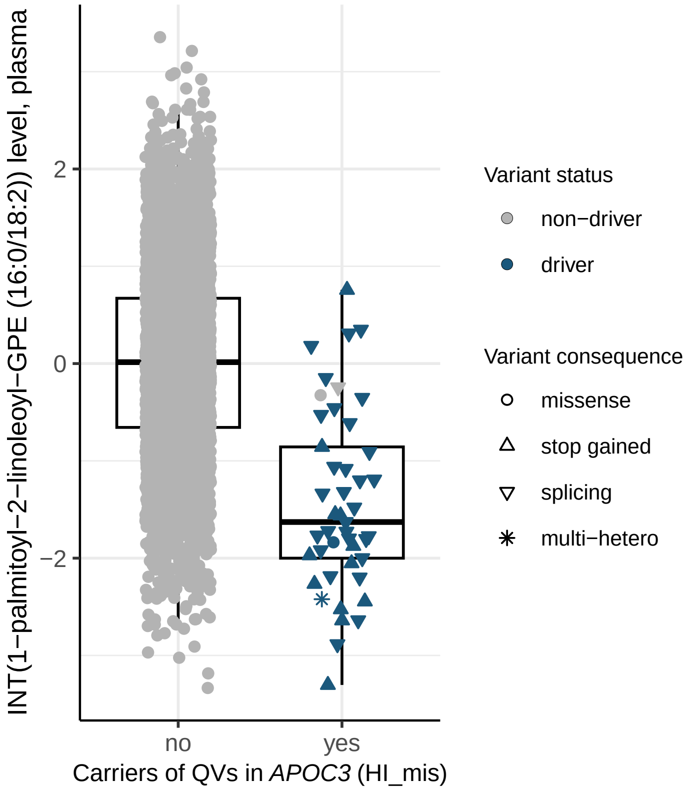

INT(1−palmitoyl−2−linoleoyl−GPE (16:0/18:2)) level, plasma
2
0
−2
no
yes
Carriers of QVs in *APOC3* (HI_mis)
Variant status
non−driver
driver
Variant consequence
missense
stop gained
splicing
multi−hetero

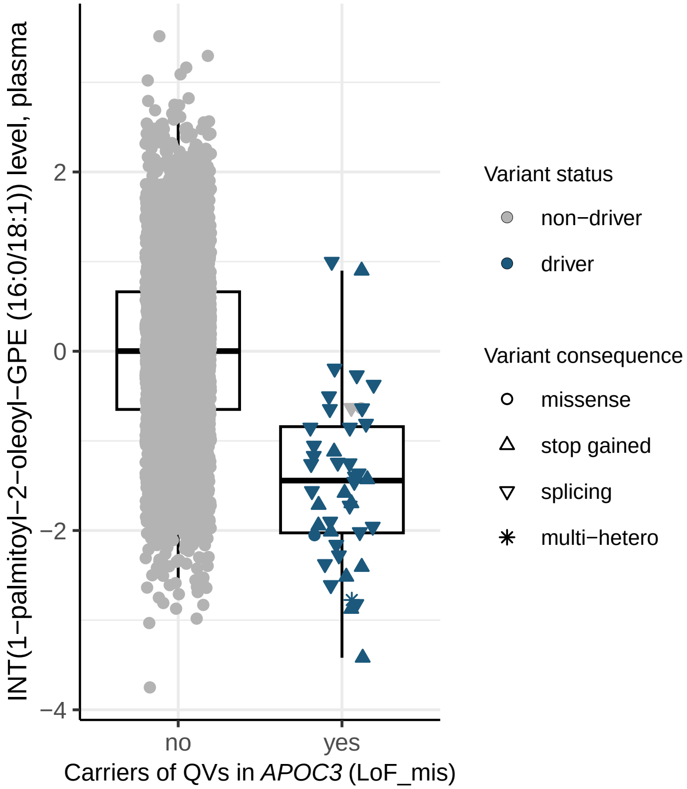

INT(1−palmitoyl−2−oleoyl−GPE (16:0/18:1)) level, plasma
2
0
−2
−4
no
yes
Carriers of QVs in *APOC3* (LoF_mis)
Variant status
non−driver
driver
Variant consequence
missense
stop gained
splicing
multi−hetero

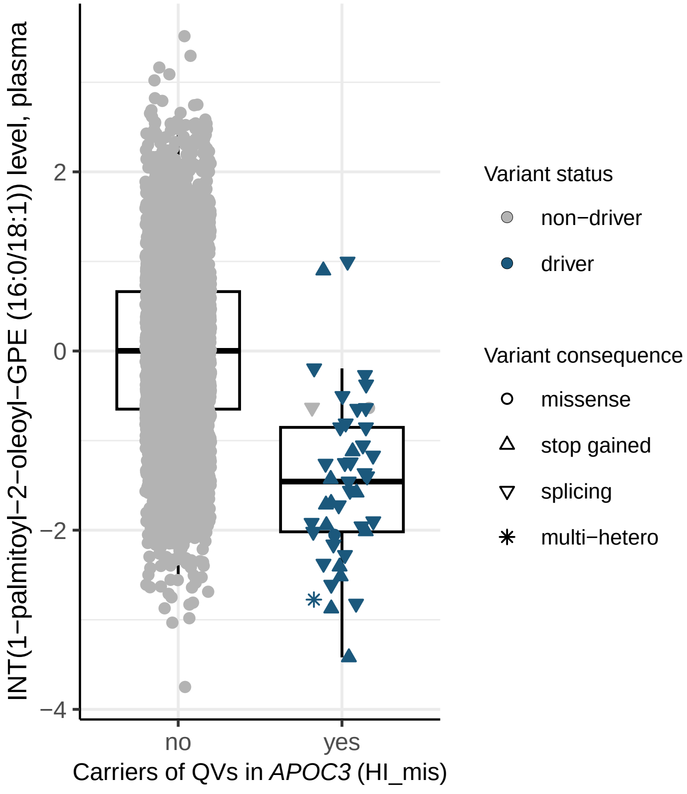

INT(1−palmitoyl−2−oleoyl−GPE (16:0/18:1)) level, plasma
2
0
−2
−4
no
yes
Carriers of QVs in *APOC3* (HI_mis)
Variant status
non−driver
driver
Variant consequence
missense
stop gained
splicing
multi−hetero

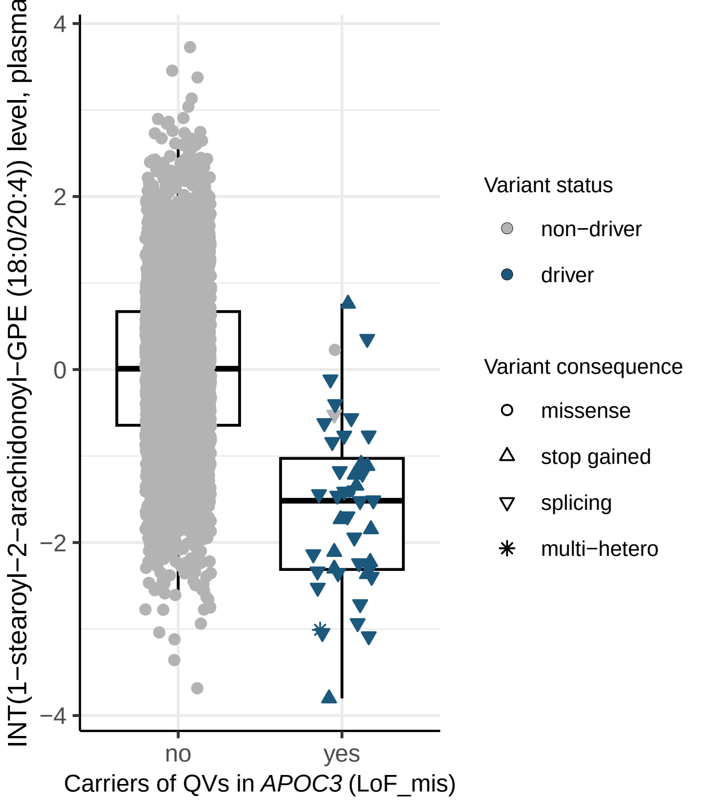
INT(1−stearoyl−2−arachidonoyl−GPE (18:0/20:4)) level, plasma
4
2
0
−2
−4
no
yes
Carriers of QVs in APOC3 (LoF_mis)
Variant status
non−driver
driver
Variant consequence
missense
stop gained
splicing
multi−hetero

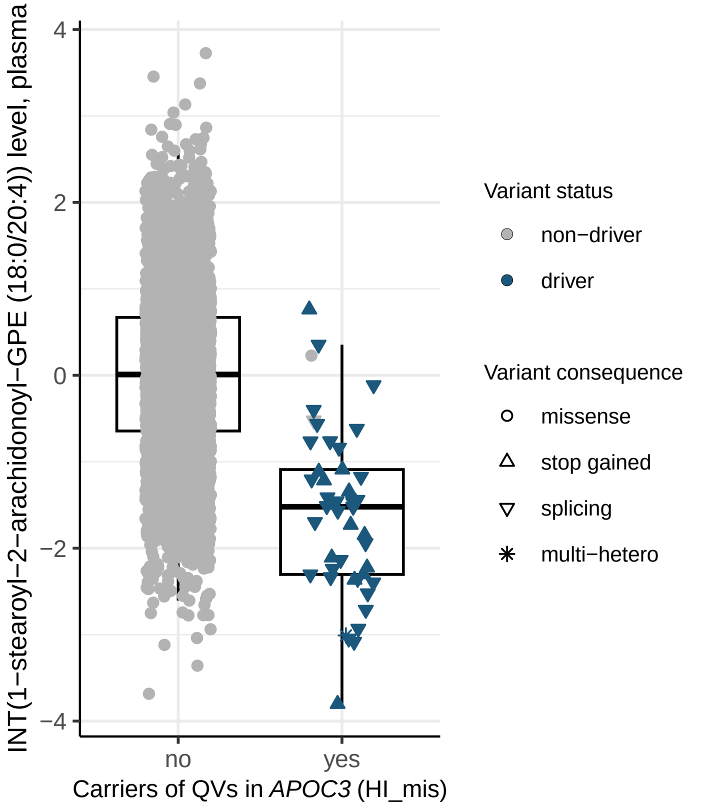
INT(1−stearoyl−2−arachidonoyl−GPE (18:0/20:4)) level, plasma
4
2
0
−2
−4
no
yes
Carriers of QVs in APOC3 (HI_mis)
Variant status
non−driver
driver
Variant consequence
missense
stop gained
splicing
multi−hetero

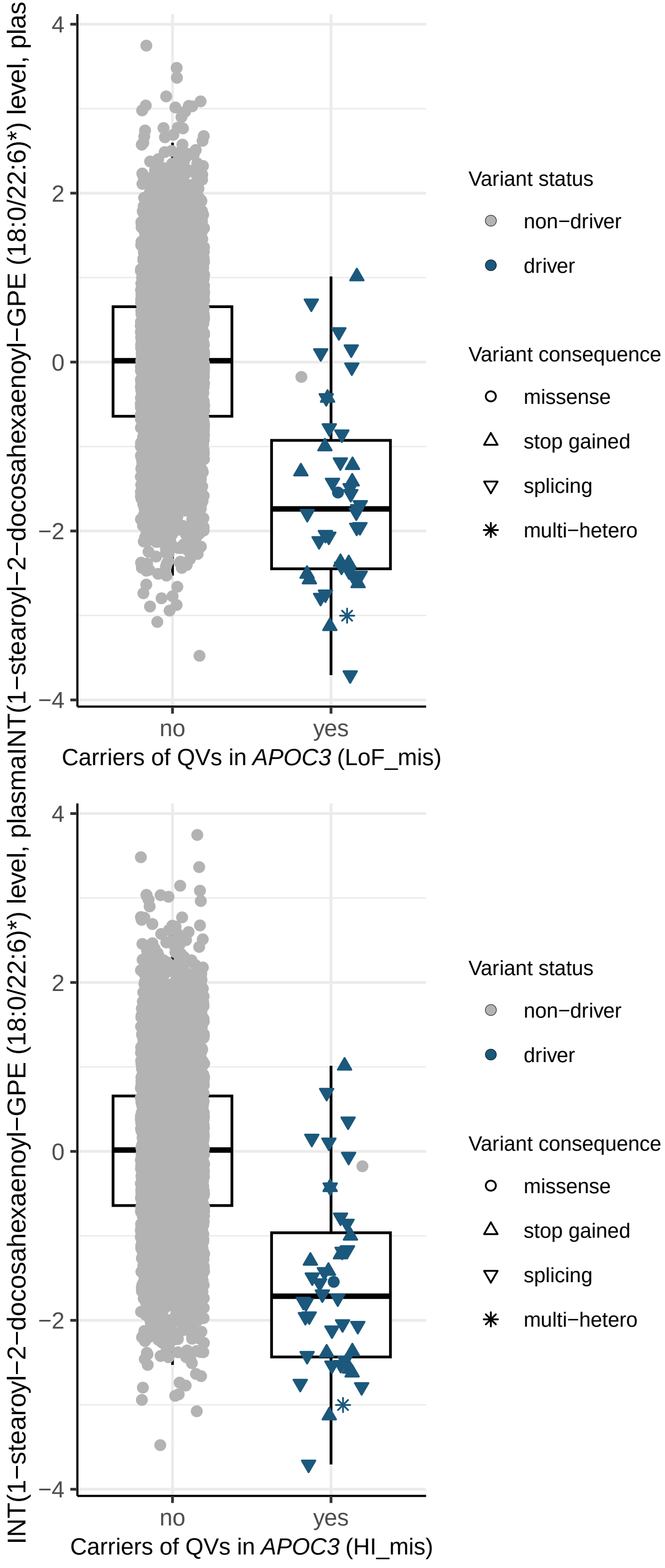
INT(1−stearoyl−2−docosahexaenoyl−GPE (18:0/22:6)*) level, plasmaINT(1−stearoyl−2−docosahexaenoyl−GPE (18:0/22:6)*) level, plas
4
2
0
−2
−4
no
yes
Carriers of QVs in APOC3 (LoF_mis)
Variant status
non−driver
driver
Variant consequence
missense
stop gained
splicing
multi−hetero
4
2
0
−2
−4
no
yes
Carriers of QVs in APOC3 (HI_mis)
Variant status
non−driver
driver
Variant consequence
missense
stop gained
splicing
multi−hetero

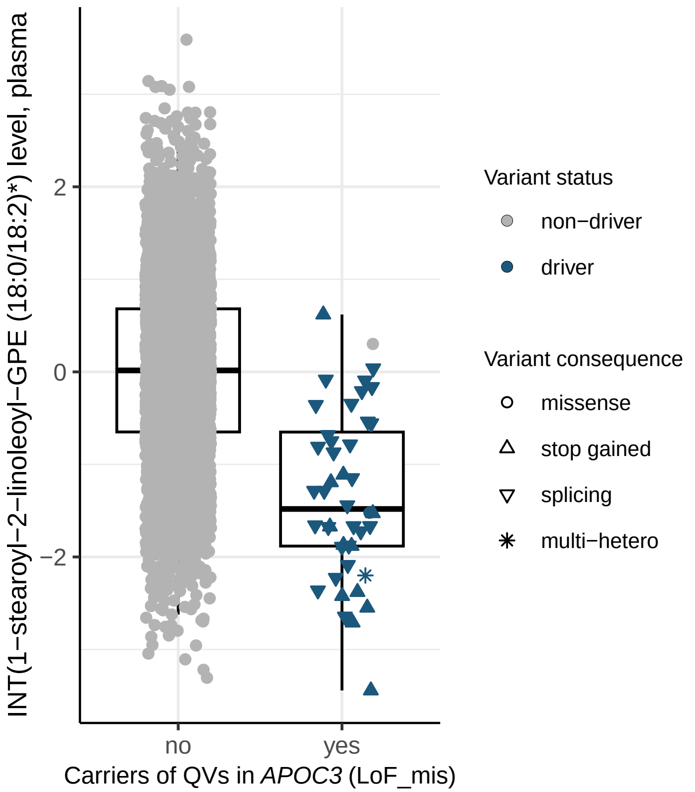
INT(1−stearoyl−2−linoleoyl−GPE (18:0/18:2)*) level, plasma
2
0
−2
no
yes
Carriers of QVs in APOC3 (LoF_mis)
Variant status
non−driver
driver
Variant consequence
missense
stop gained
splicing
multi−hetero

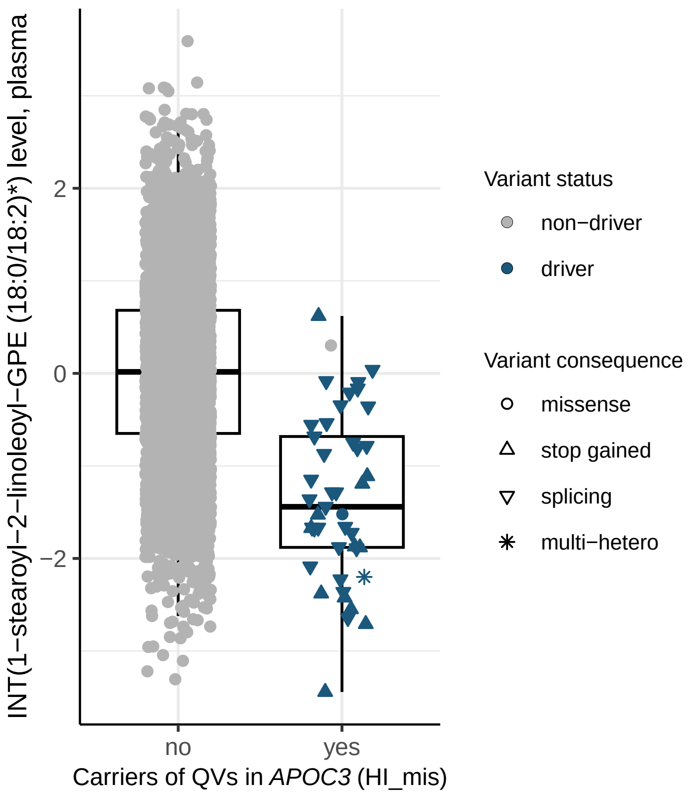
INT(1−stearoyl−2−linoleoyl−GPE (18:0/18:2)*) level, plasma
2
0
−2
no
yes
Carriers of QVs in APOC3 (HI_mis)
Variant status
non−driver
driver
Variant consequence
missense
stop gained
splicing
multi−hetero

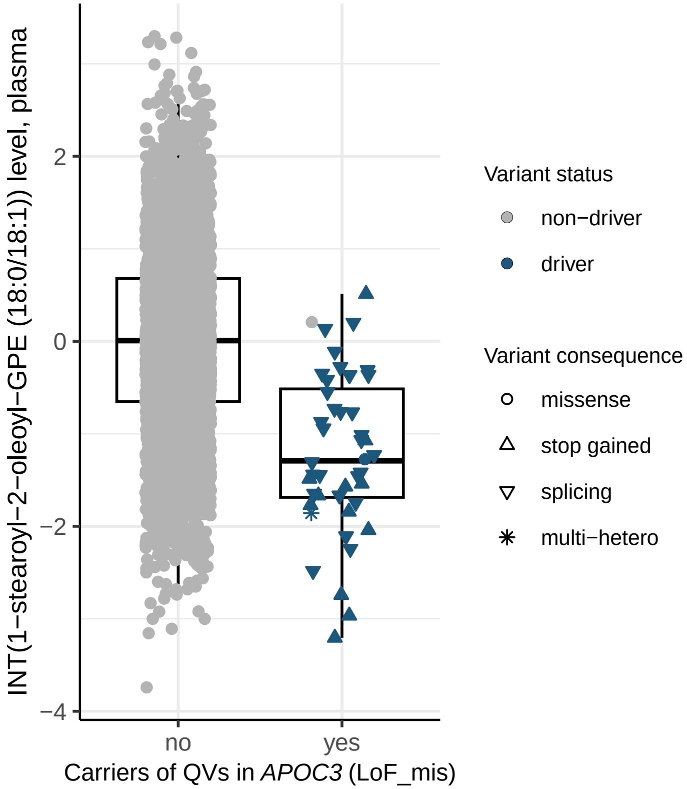
INT(1−stearoyl−2−oleoyl−GPE (18:0/18:1)) level, plasma
2
0
−2
−4
no
yes
Carriers of QVs in *APOC3* (LoF_mis)
Variant status
non−driver
driver
Variant consequence
missense
stop gained
splicing
multi−hetero

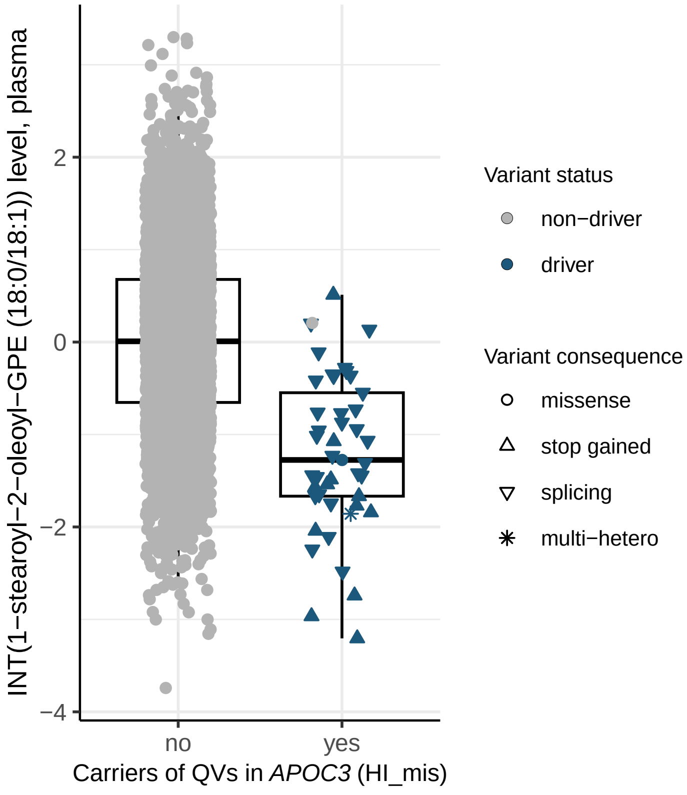
INT(1−stearoyl−2−oleoyl−GPE (18:0/18:1)) level, plasma
2
0
−2
−4
no
yes
Carriers of QVs in *APOC3* (HI_mis)
Variant status
non−driver
driver
Variant consequence
missense
stop gained
splicing
multi−hetero

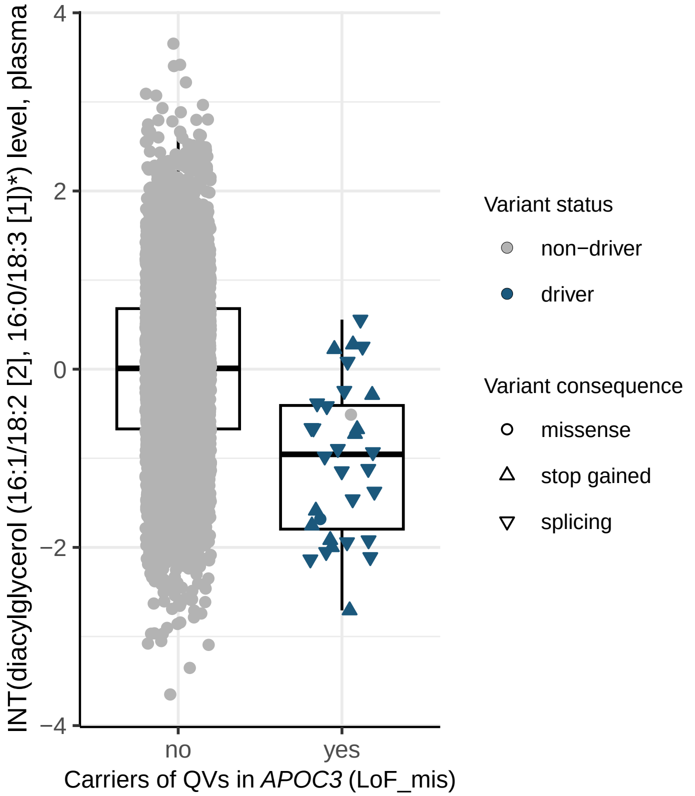

INT(diacylglycerol (16:1/18:2 [2], 16:0/18:3 [1])*) level, plasma
4
2
0
−2
−4
no
yes
Carriers of QVs in APOC3 (LoF_mis)
Variant status
non−driver
driver
Variant consequence
missense
stop gained
splicing

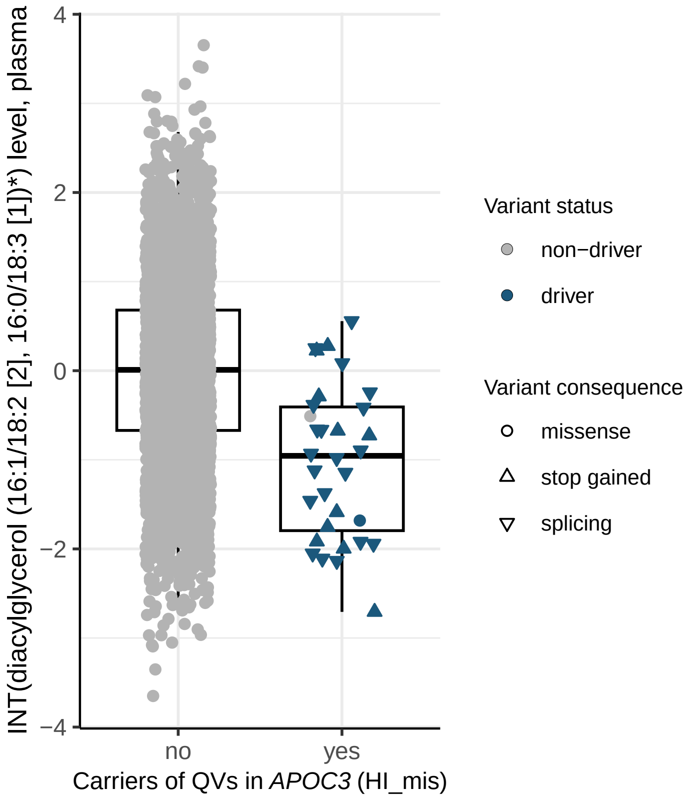

INT(diacylglycerol (16:1/18:2 [2], 16:0/18:3 [1])*) level, plasma
4
2
0
−2
−4
no
yes
Carriers of QVs in APOC3 (HI_mis)
Variant status
non−driver
driver
Variant consequence
missense
stop gained
splicing

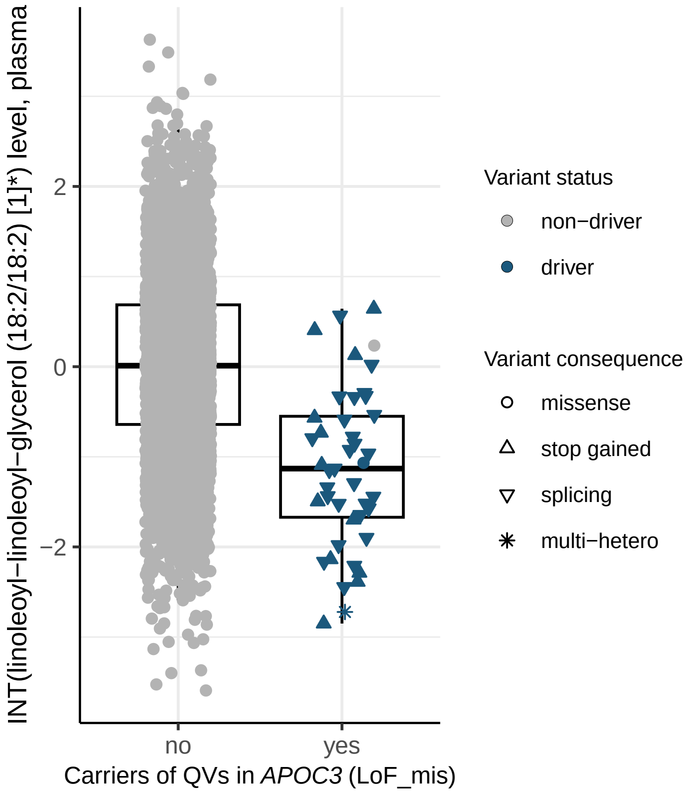
INT(linoleoyl−linoleoyl−glycerol (18:2/18:2) [1]*) level, plasma
2
0
−2
no
yes
Carriers of QVs in APOC3 (LoF_mis)
Variant status
non−driver
driver
Variant consequence
missense
stop gained
splicing
multi−hetero

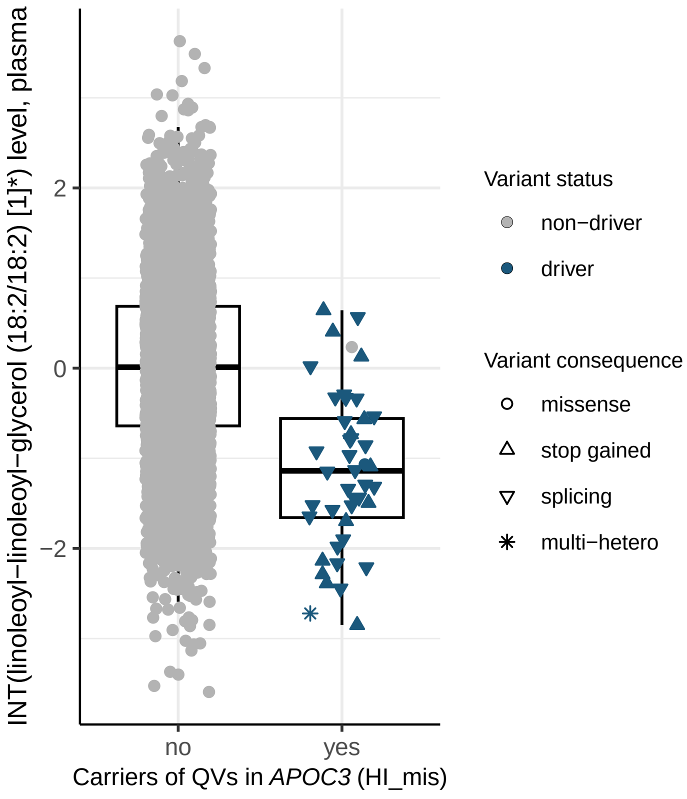
INT(linoleoyl−linoleoyl−glycerol (18:2/18:2) [1]*) level, plasma
2
0
−2
no
yes
Carriers of QVs in APOC3 (HI_mis)
Variant status
non−driver
driver
Variant consequence
missense
stop gained
splicing
multi−hetero

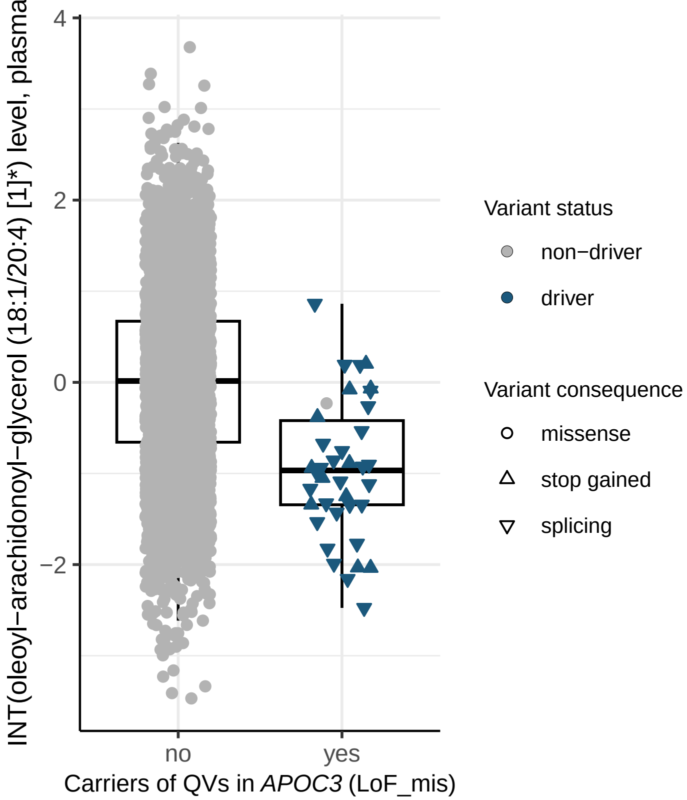
INT(oleoyl−arachidonoyl−glycerol (18:1/20:4) [1]*) level, plasma
4
2
0
−2
no
yes
Carriers of QVs in *APOC3* (LoF_mis)
Variant status
non−driver
driver
Variant consequence
missense
stop gained
splicing

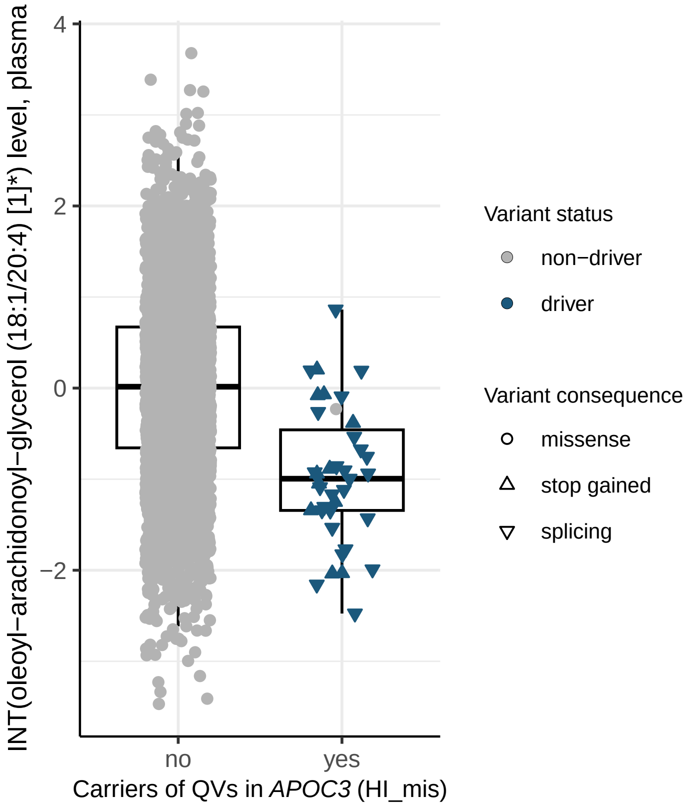
INT(oleoyl−arachidonoyl−glycerol (18:1/20:4) [1]*) level, plasma
4
2
0
−2
no
yes
Carriers of QVs in *APOC3* (HI_mis)
Variant status
non−driver
driver
Variant consequence
missense
stop gained
splicing

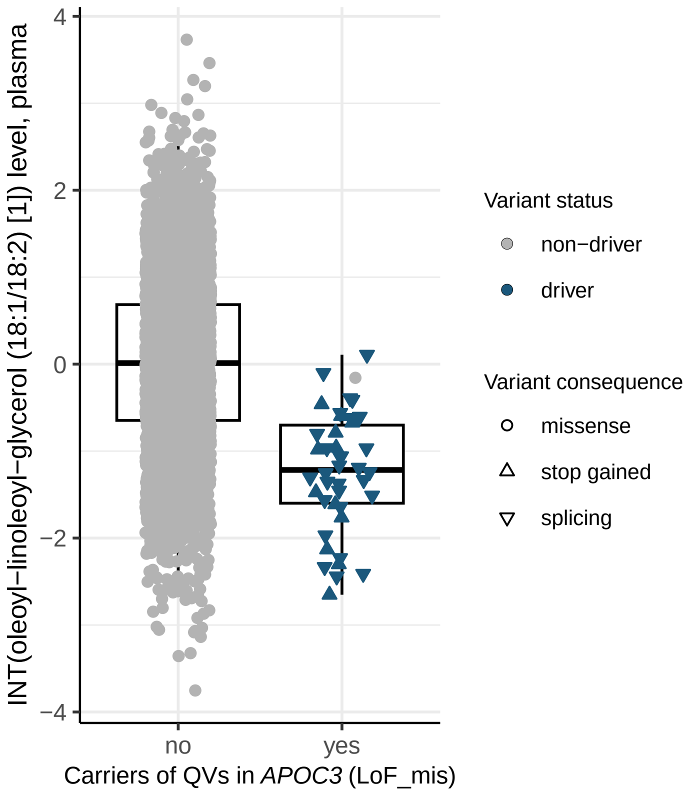
INT(oleoyl−linoleoyl−glycerol (18:1/18:2) [1]) level, plasma
4
2
0
−2
−4
no
yes
Carriers of QVs in APOC3 (LoF_mis)
Variant status
non−driver
driver
Variant consequence
missense
stop gained
splicing

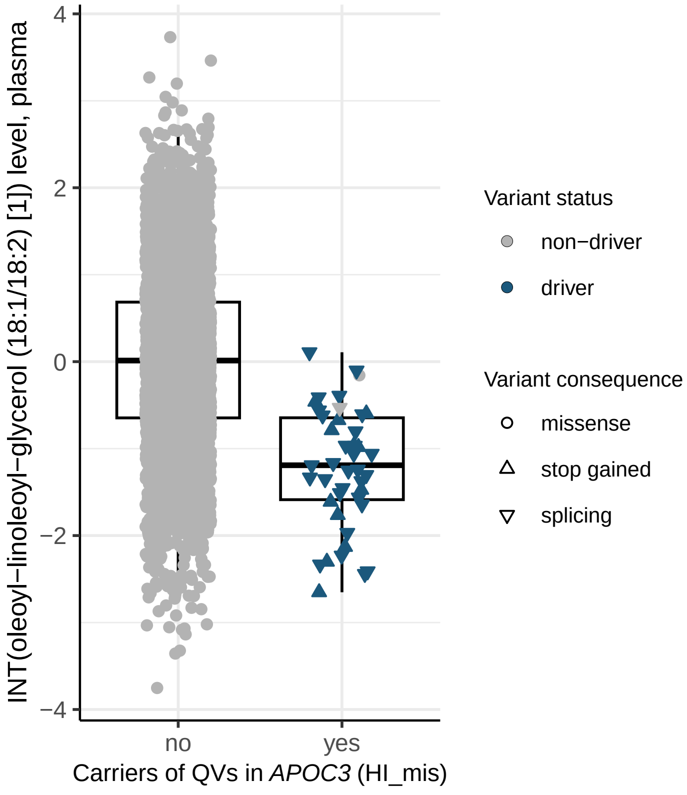
INT(oleoyl−linoleoyl−glycerol (18:1/18:2) [1]) level, plasma
4
2
0
−2
−4
no
yes
Carriers of QVs in APOC3 (HI_mis)
Variant status
non−driver
driver
Variant consequence
missense
stop gained
splicing

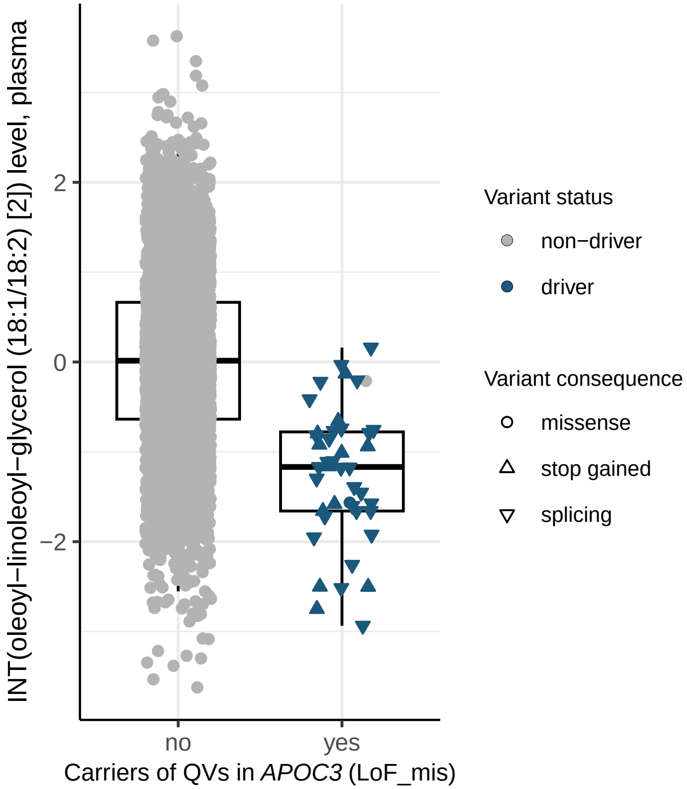
INT(oleoyl−linoleoyl−glycerol (18:1/18:2) [2]) level, plasma
2
0
−2
no
yes
Carriers of QVs in *APOC3* (LoF_mis)
Variant status
non−driver
driver
Variant consequence
missense
stop gained
splicing

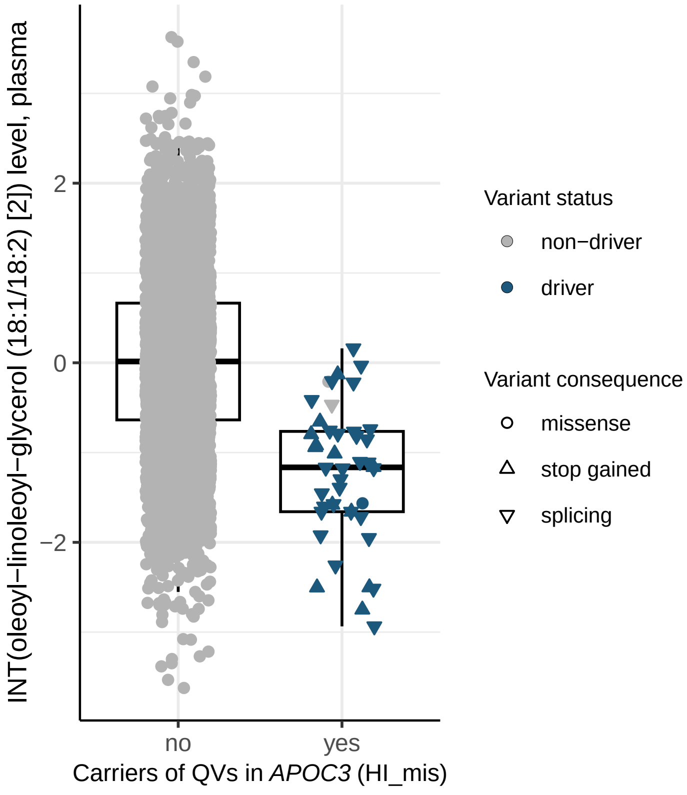
INT(oleoyl−linoleoyl−glycerol (18:1/18:2) [2]) level, plasma
2
0
−2
no
yes
Carriers of QVs in *APOC3* (HI_mis)
Variant status
non−driver
driver
Variant consequence
missense
stop gained
splicing

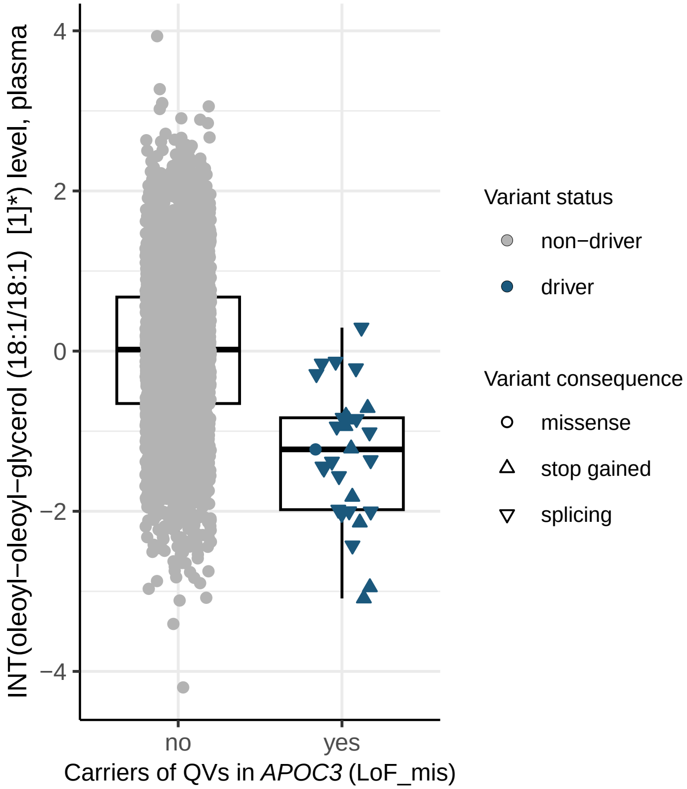
INT(oleoyl−oleoyl−glycerol (18:1/18:1) [1]*) level, plasma
4
2
0
−2
−4
no
yes
Carriers of QVs in APOC3 (LoF_mis)
Variant status
non−driver
driver
Variant consequence
missense
stop gained
splicing

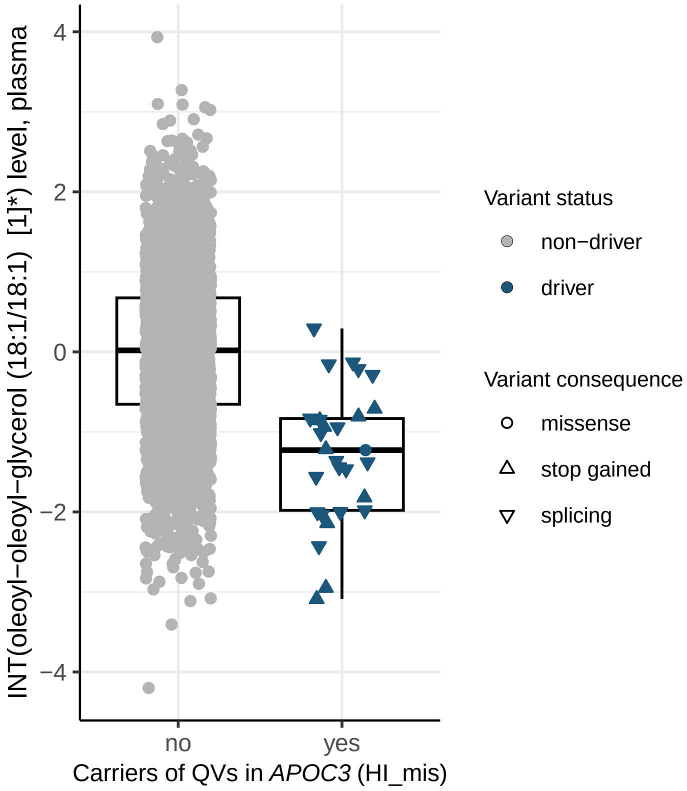
INT(oleoyl−oleoyl−glycerol (18:1/18:1) [1]*) level, plasma
4
2
0
−2
−4
no
yes
Carriers of QVs in APOC3 (HI_mis)
Variant status
non−driver
driver
Variant consequence
missense
stop gained
splicing

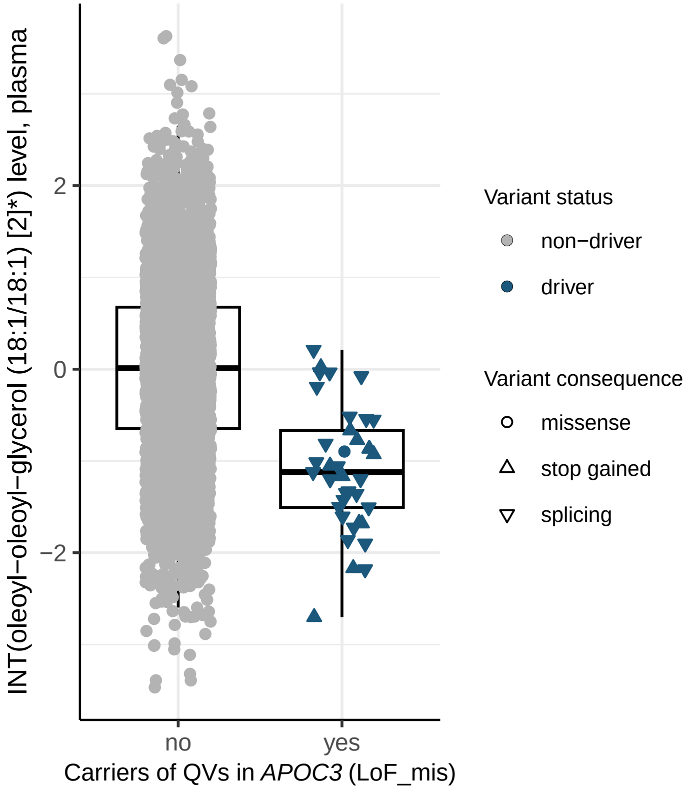
INT(oleoyl−oleoyl−glycerol (18:1/18:1) [2]*) level, plasma
2
0
−2
no
yes
Carriers of QVs in *APOC3* (LoF_mis)
Variant status
non−driver
driver
Variant consequence
missense
stop gained
splicing

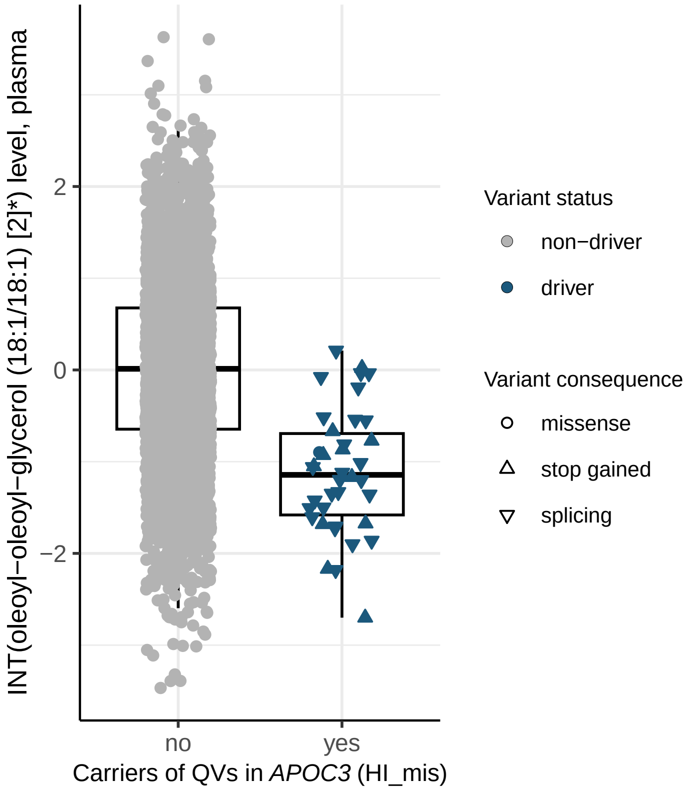
INT(oleoyl−oleoyl−glycerol (18:1/18:1) [2]*) level, plasma
2
0
−2
no
yes
Carriers of QVs in *APOC3* (HI_mis)
Variant status
non−driver
driver
Variant consequence
missense
stop gained
splicing

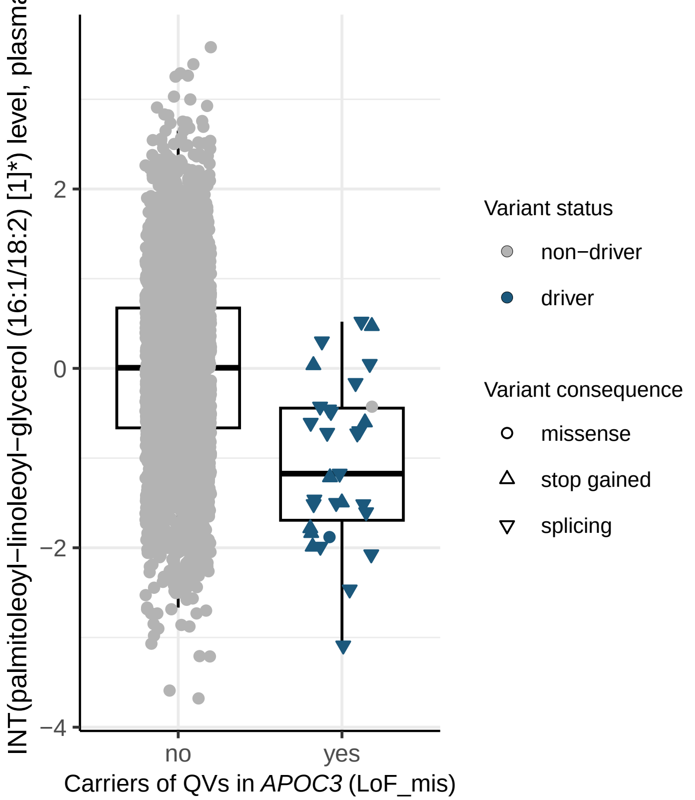
INT(palmitoleoyl−linoleoyl−glycerol (16:1/18:2) [1]*) level, plasma
2
0
−2
−4
no
yes
Carriers of QVs in APOC3 (LoF_mis)
Variant status
non−driver
driver
Variant consequence
missense
stop gained
splicing

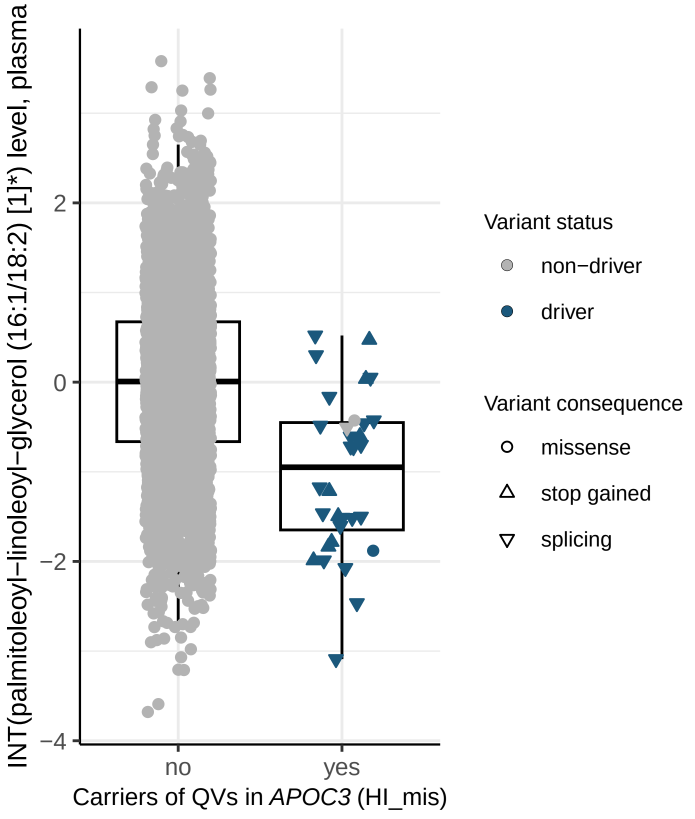
INT(palmitoleoyl−linoleoyl−glycerol (16:1/18:2) [1]*) level, plasma
2
0
−2
−4
no
yes
Carriers of QVs in APOC3 (HI_mis)
Variant status
non−driver
driver
Variant consequence
missense
stop gained
splicing

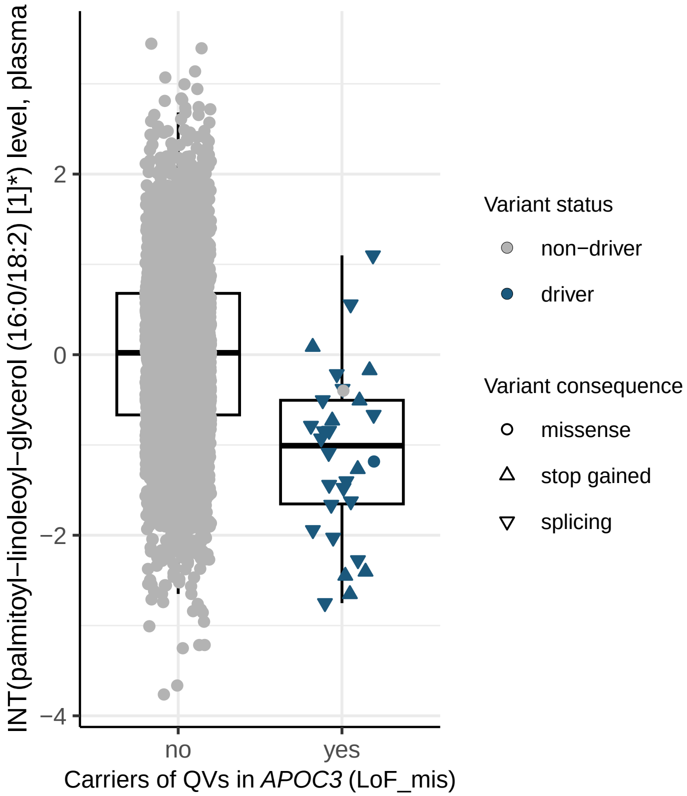
INT(palmitoyl−linoleoyl−glycerol (16:0/18:2) [1]*) level, plasma
2
0
−2
−4
no
yes
Carriers of QVs in *APOC3* (LoF_mis)
Variant status
non−driver
driver
Variant consequence
missense
stop gained
splicing

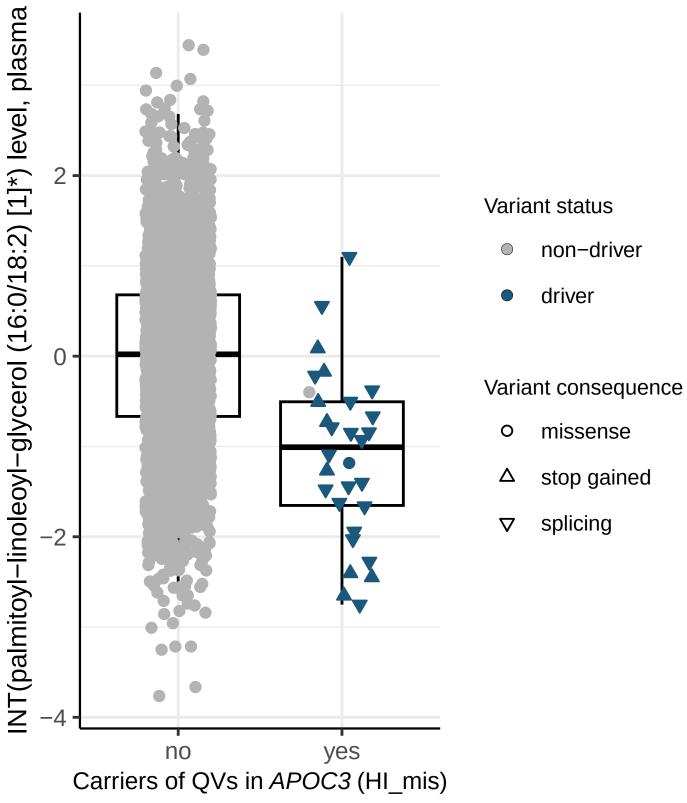
INT(palmitoyl−linoleoyl−glycerol (16:0/18:2) [1]*) level, plasma
2
0
−2
−4
no
yes
Carriers of QVs in *APOC3* (HI_mis)
Variant status
non−driver
driver
Variant consequence
missense
stop gained
splicing

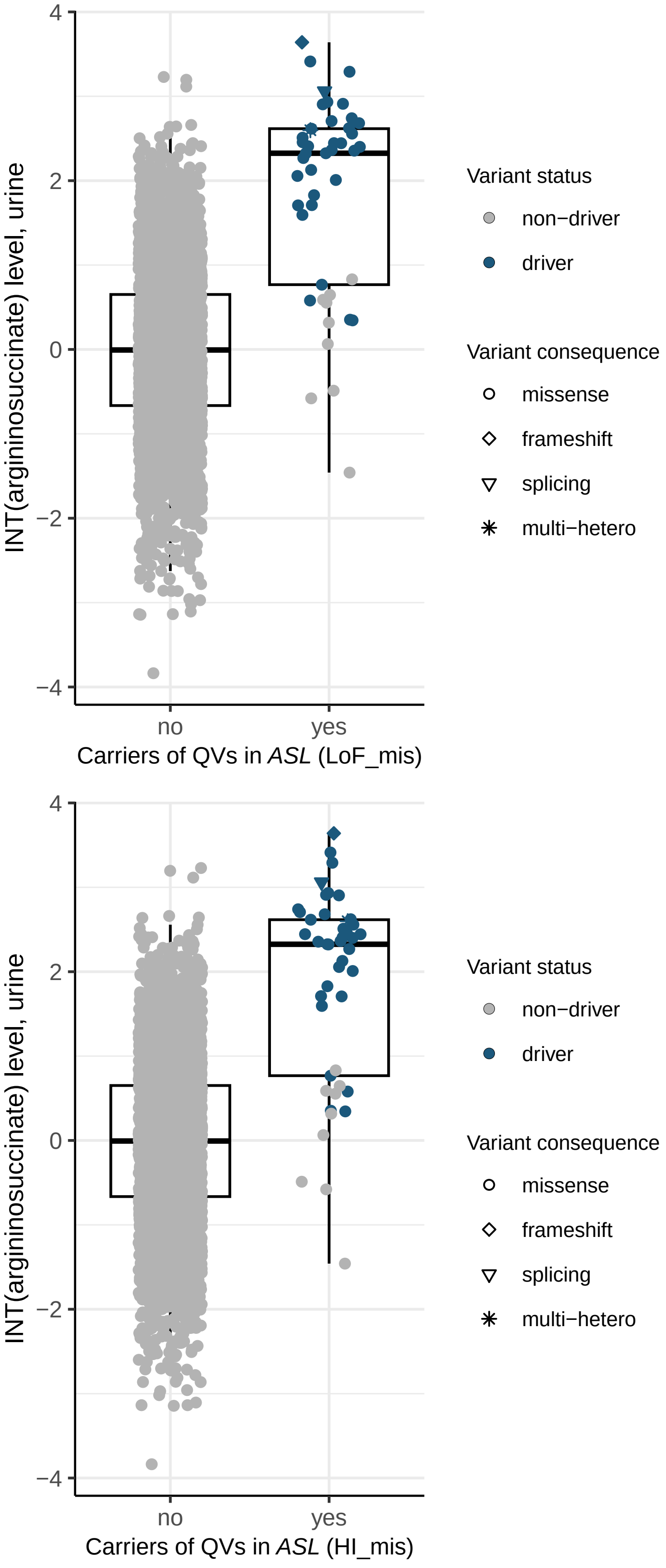
INT(argininosuccinate) level, urine
4
2
0
−2
−4
no
yes
Carriers of QVs in *ASL* (LoF_mis)
Variant status
non−driver
driver
Variant consequence
missense
frameshift
splicing
multi−hetero
INT(argininosuccinate) level, urine
4
2
0
−2
−4
no
yes
Carriers of QVs in *ASL* (HI_mis)
Variant status
non−driver
driver
Variant consequence
missense
frameshift
splicing
multi−hetero

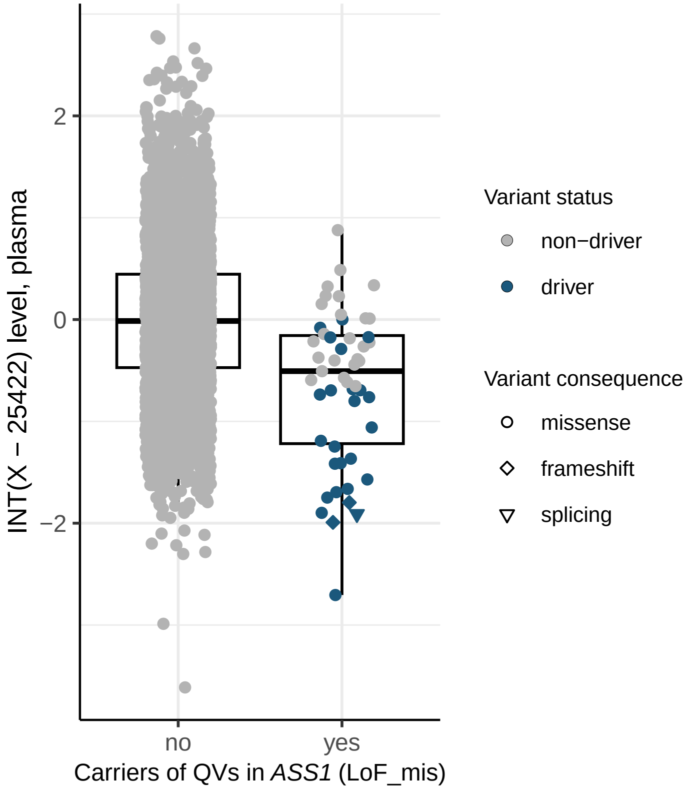

INT(X − 25422) level, plasma
2
0
−2
no
yes
Carriers of QVs in *ASS1* (LoF_mis)
Variant status
non−driver
driver
Variant consequence
missense
frameshift
splicing

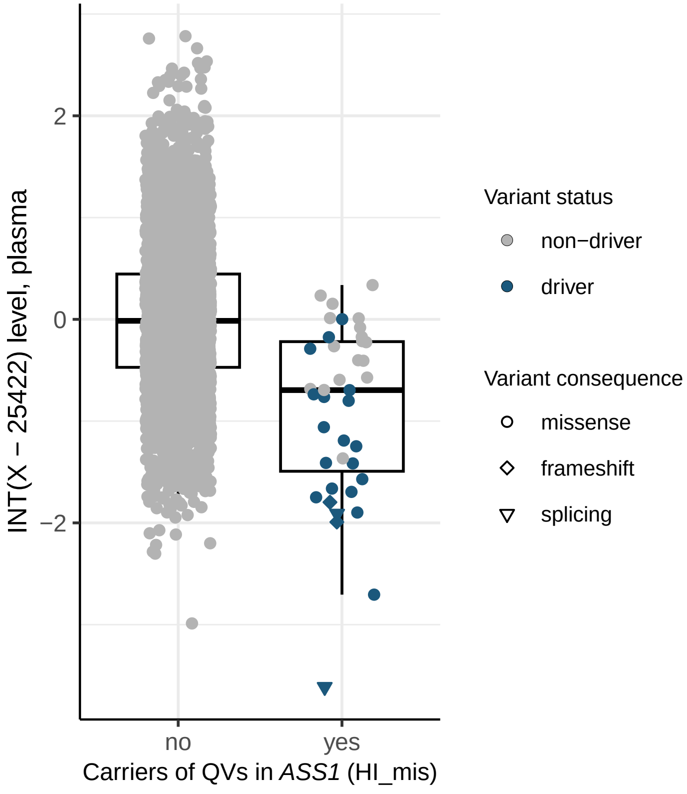

INT(X − 25422) level, plasma
2
0
−2
no
yes
Carriers of QVs in *ASS1* (HI_mis)
Variant status
non−driver
driver
Variant consequence
missense
frameshift
splicing

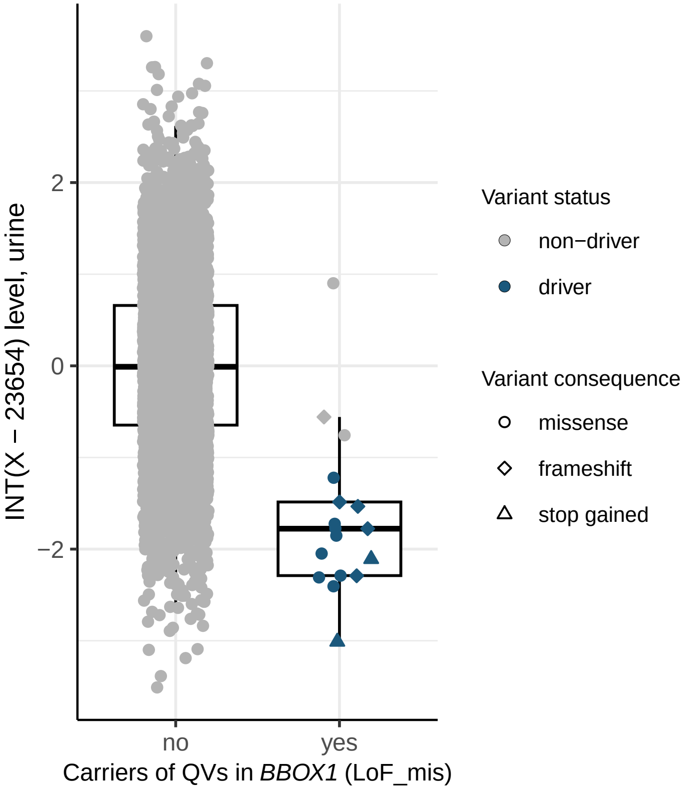

INT(X − 23654) level, urine
2
0
−2
no
yes
Carriers of QVs in *BBOX1* (LoF_mis)
Variant status
non−driver
driver
Variant consequence
missense
frameshift
stop gained

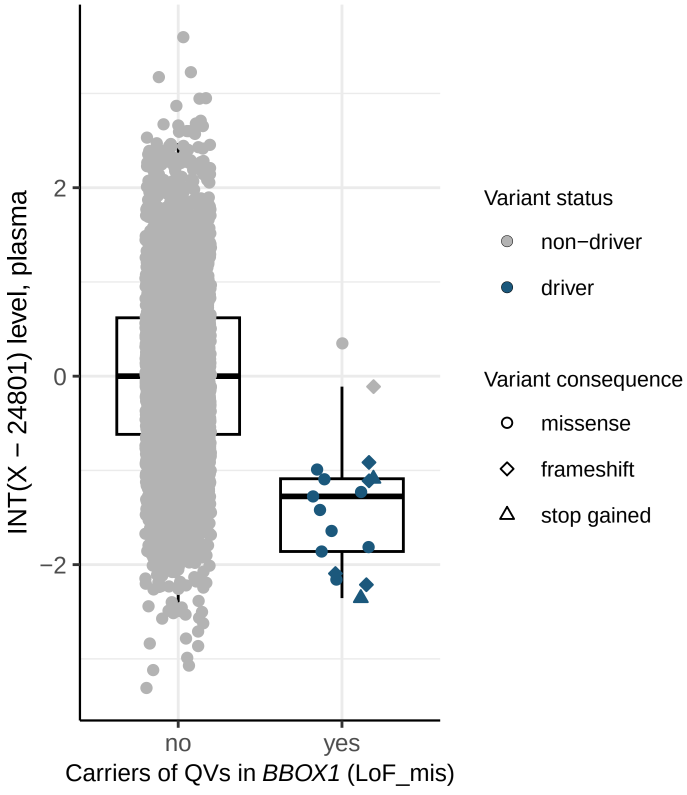

INT(X − 24801) level, plasma
2
0
−2
no
yes
Carriers of QVs in *BBOX1* (LoF_mis)
Variant status
non−driver
driver
Variant consequence
missense
frameshift
stop gained

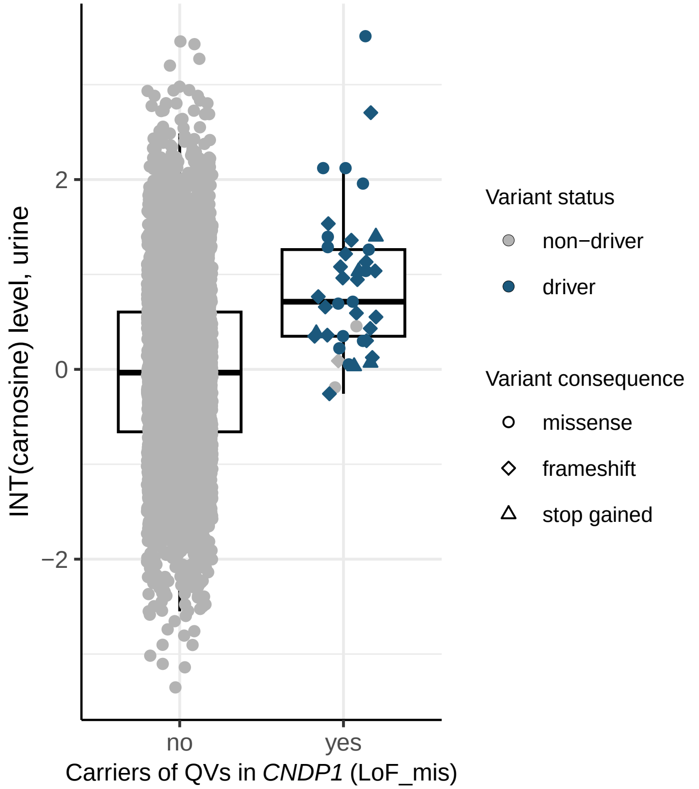

INT(carnosine) level, urine
2
0
−2
no
yes
Carriers of QVs in *CNDP1* (LoF_mis)
Variant status
non−driver
driver
Variant consequence
missense
frameshift
stop gained

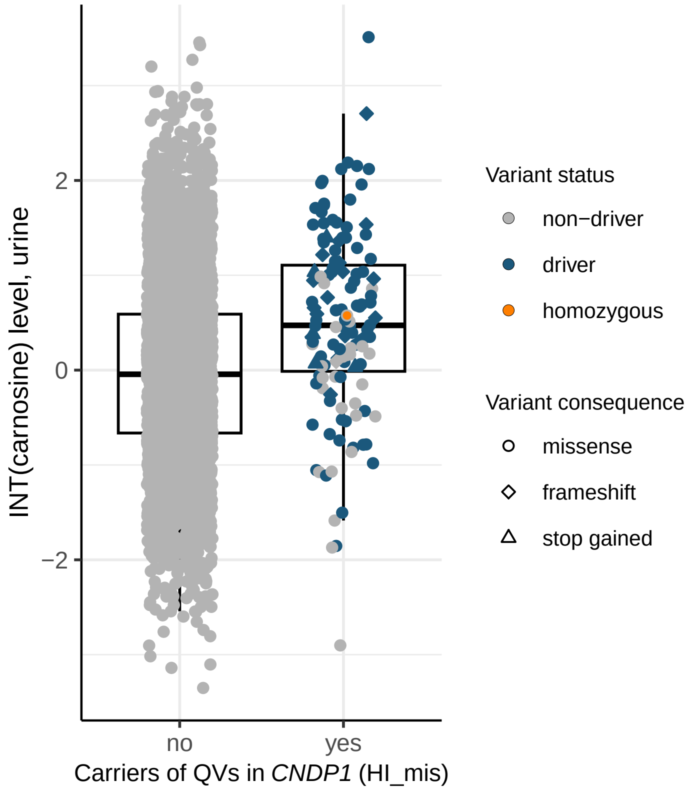

INT(carnosine) level, urine
2
0
−2
no
yes
Carriers of QVs in *CNDP1* (HI_mis)
Variant status
non−driver
driver
homozygous
Variant consequence
missense
frameshift
stop gained

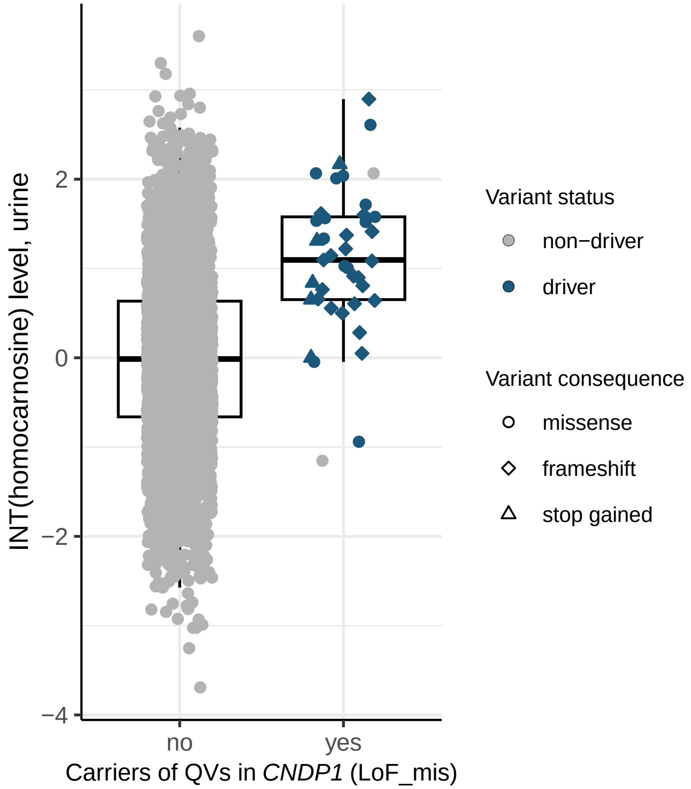
INT(homocarnosine) level, urine
2
0
−2
−4
no
yes
Carriers of QVs in CNDP1 (LoF_mis)
Variant status
non−driver
driver
Variant consequence
missense
frameshift
stop gained

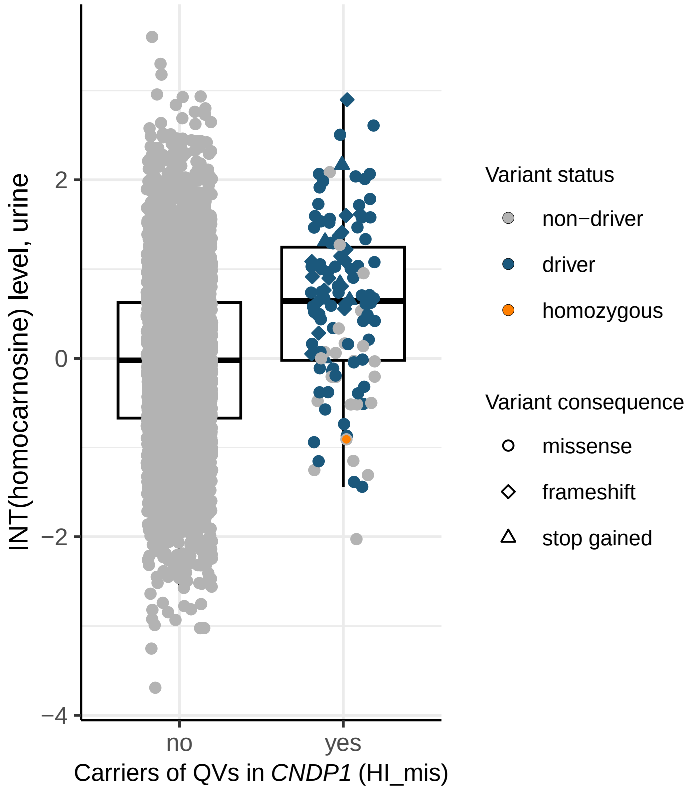
INT(homocarnosine) level, urine
2
0
−2
−4
no
yes
Carriers of QVs in CNDP1 (HI_mis)
Variant status
non−driver
driver
homozygous
Variant consequence
missense
frameshift
stop gained

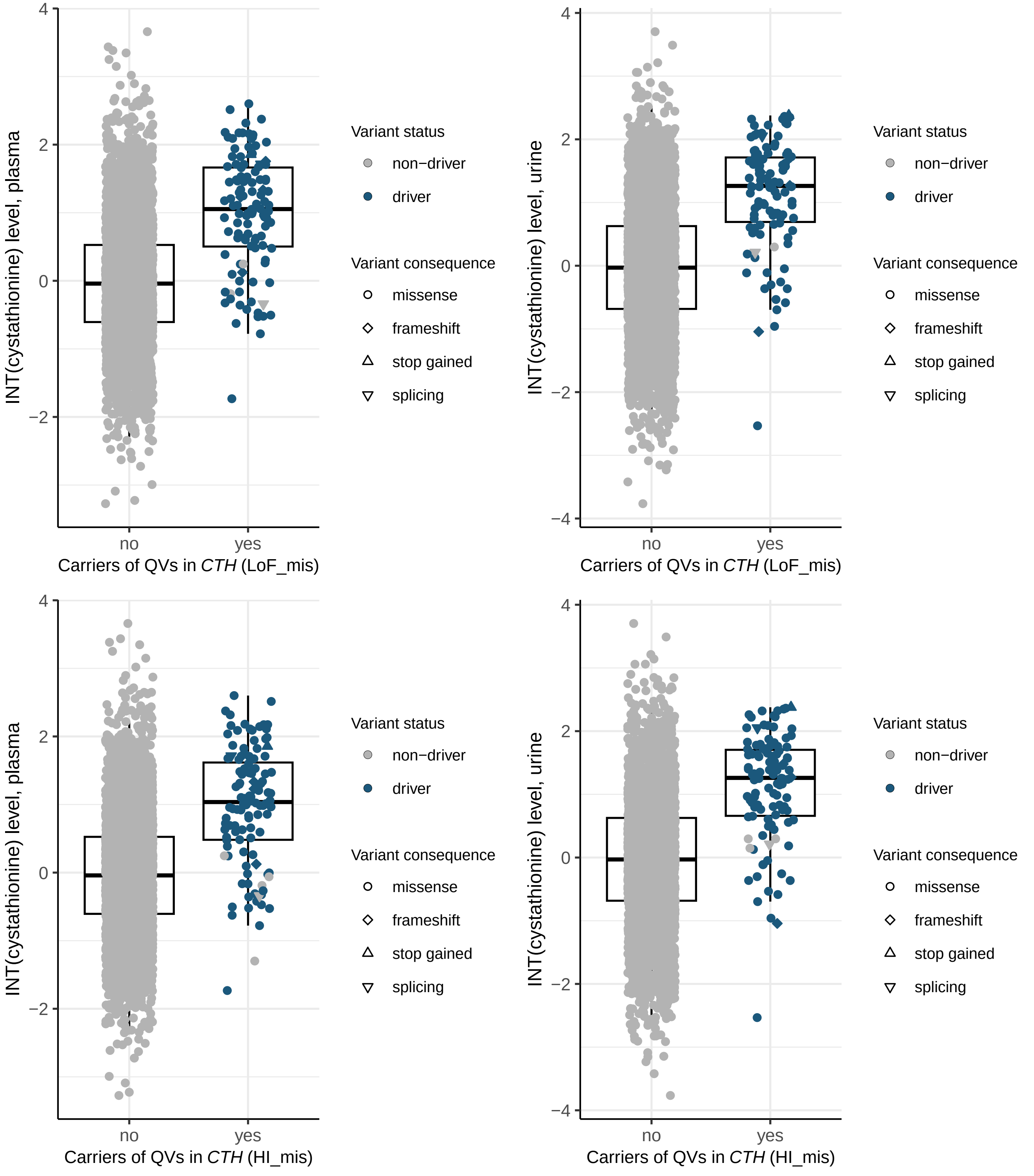

INT(cystathionine) level, plasma
Carriers of QVs in CTH (LoF_mis)
INT(cystathionine) level, urine
Carriers of QVs in CTH (LoF_mis)
INT(cystathionine) level, plasma
Carriers of QVs in CTH (HI_mis)
INT(cystathionine) level, urine
Carriers of QVs in CTH (HI_mis)
no
yes
Variant status
non−driver
driver
Variant consequence
missense
frameshift
stop gained
splicing

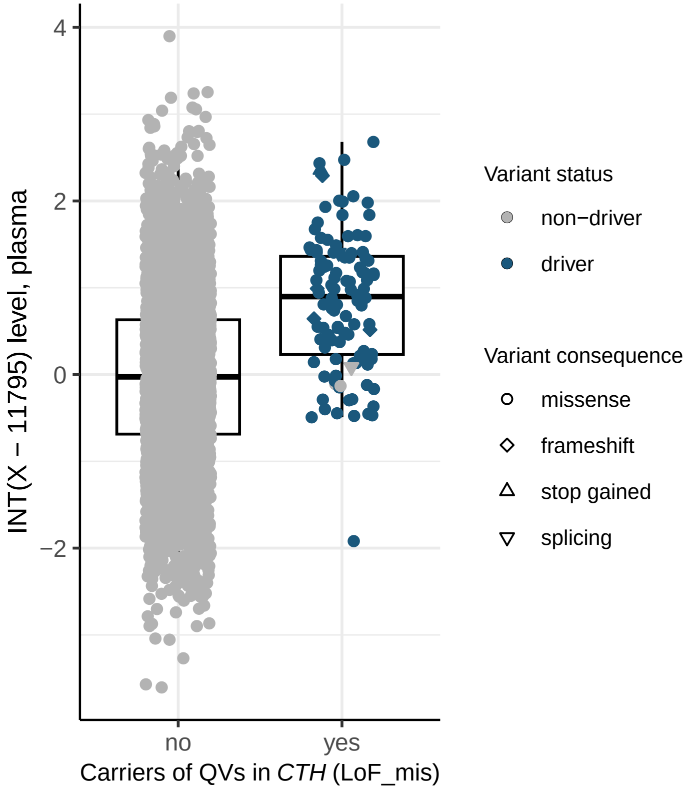
INT(X − 11795) level, plasma
4
2
0
−2
no
yes
Carriers of QVs in *CTH* (LoF_mis)
Variant status
non−driver
driver
Variant consequence
missense
frameshift
stop gained
splicing

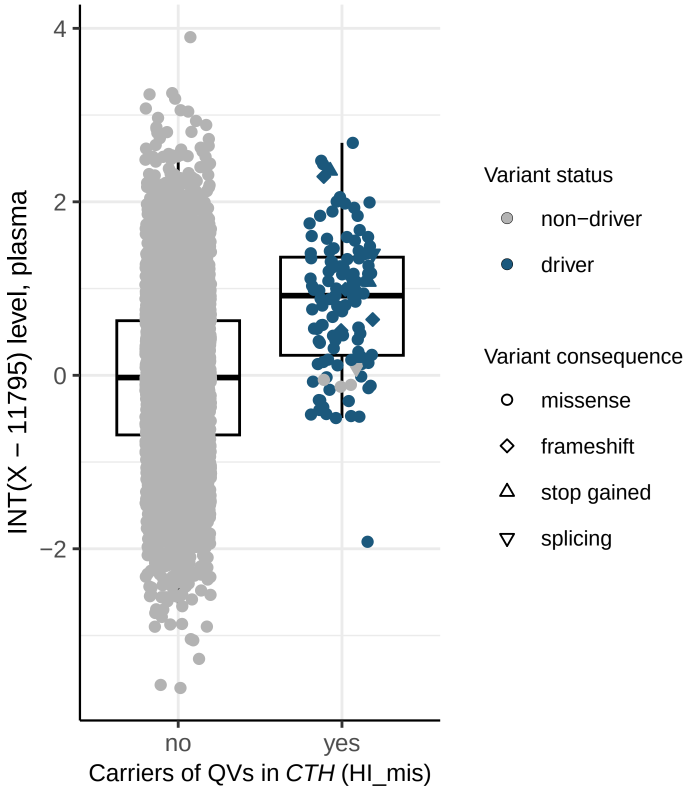
INT(X − 11795) level, plasma
4
2
0
−2
no
yes
Carriers of QVs in *CTH* (HI_mis)
Variant status
non−driver
driver
Variant consequence
missense
frameshift
stop gained
splicing

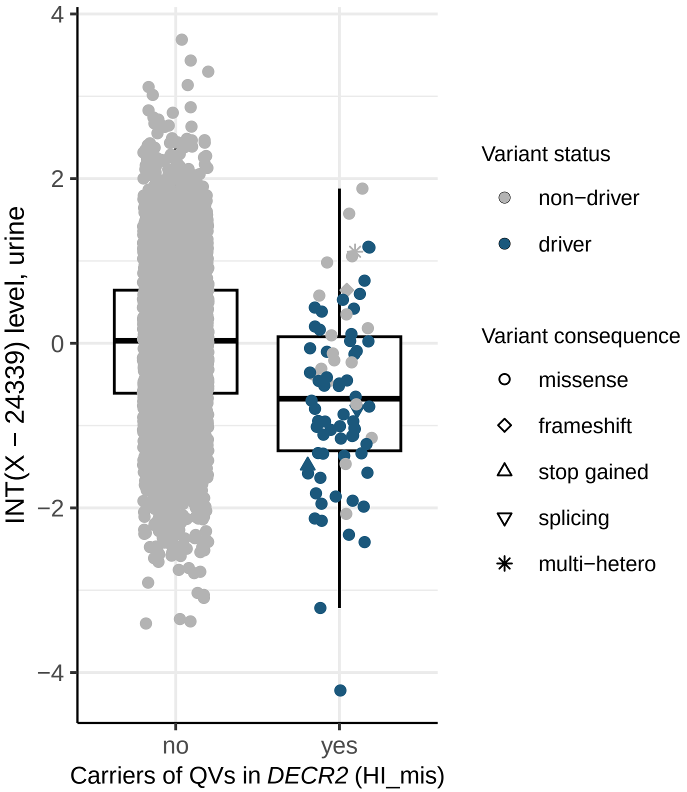
INT(X – 24339) level, urine
4
2
0
−2
−4
no
yes
Carriers of QVs in *DECR2* (HI_mis)
Variant status
non−driver
driver
Variant consequence
missense
frameshift
stop gained
splicing
multi−hetero

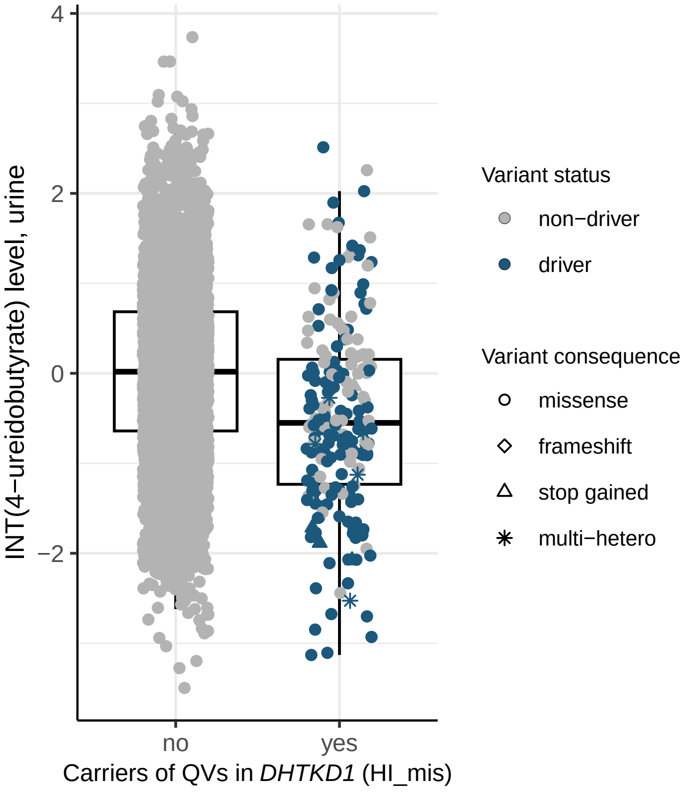
INT(4−ureidobutyrate) level, urine
4
2
0
−2
no
yes
Carriers of QVs in *DHTKD1* (HI_mis)
Variant status
non−driver
driver
Variant consequence
missense
frameshift
stop gained
multi−hetero

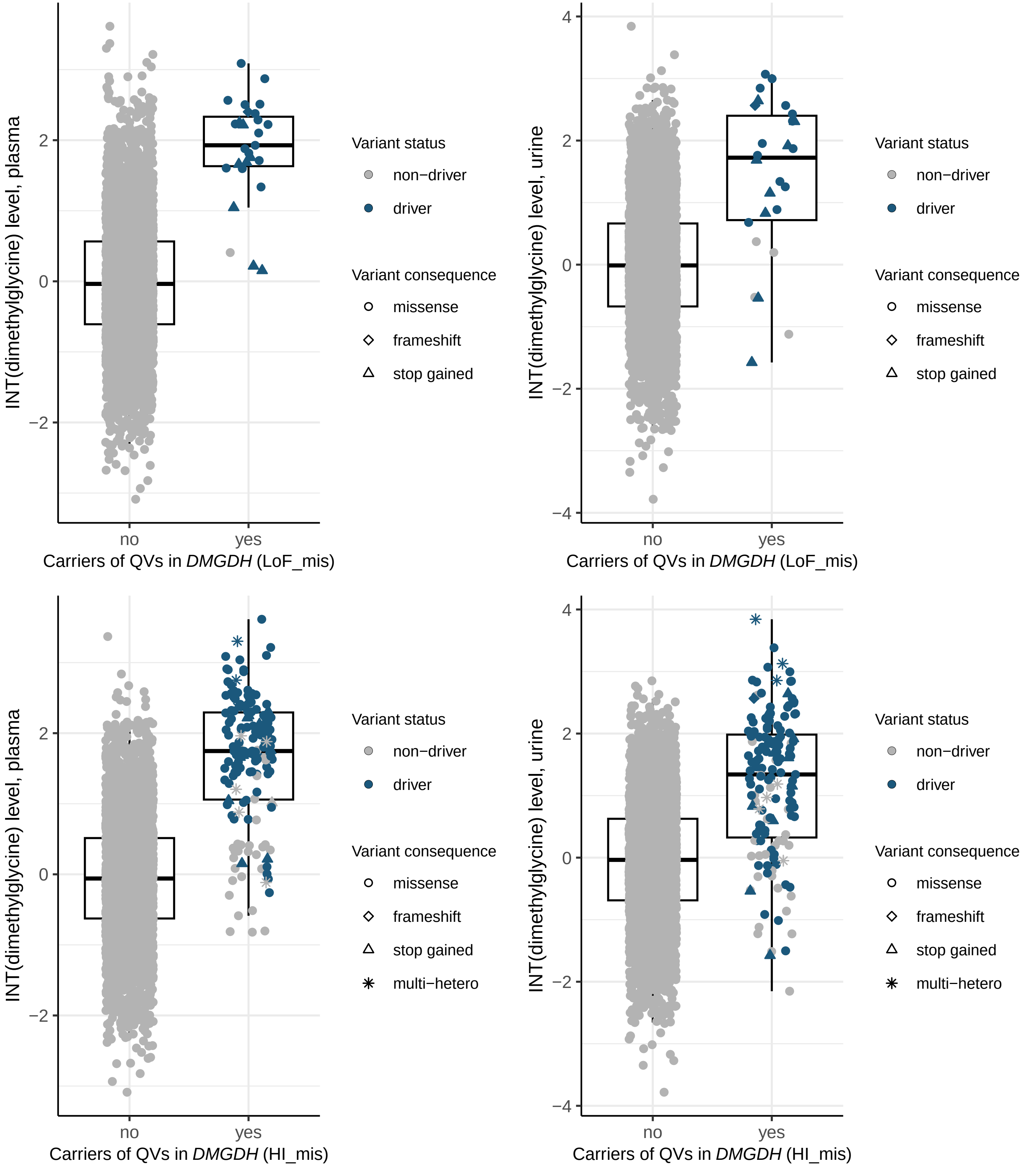
INT(dimethylglycine) level, plasma
INT(dimethylglycine) level, urine
Carriers of QVs in *DMGDH* (LoF_mis)
Carriers of QVs in *DMGDH* (HI_mis)
no
yes
Variant status
non−driver
driver
Variant consequence
missense
frameshift
stop gained
multi−hetero

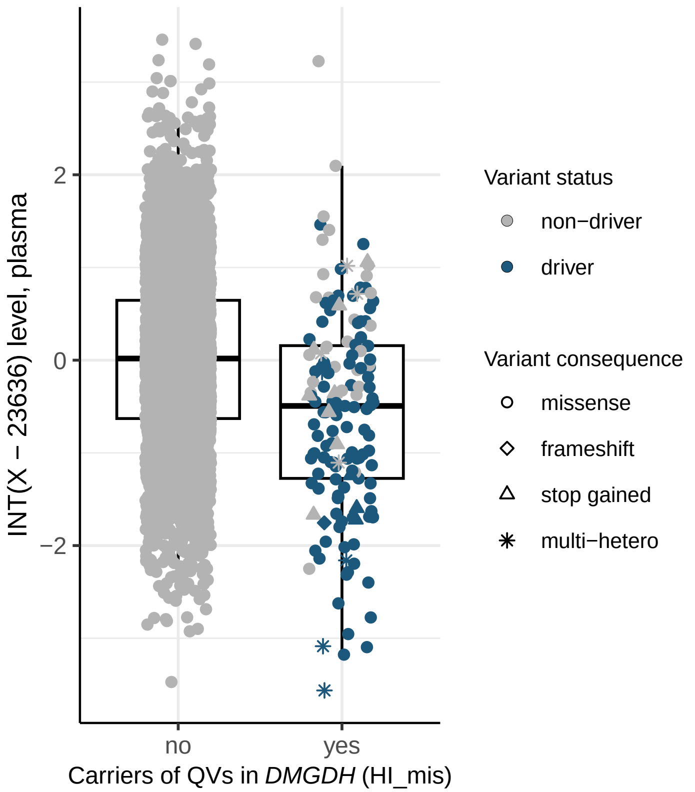

INT(X − 23636) level, plasma
2
0
−2
no
yes
Carriers of QVs in DMGDH (HI_mis)
Variant status
non−driver
driver
Variant consequence
missense
frameshift
stop gained
multi−hetero

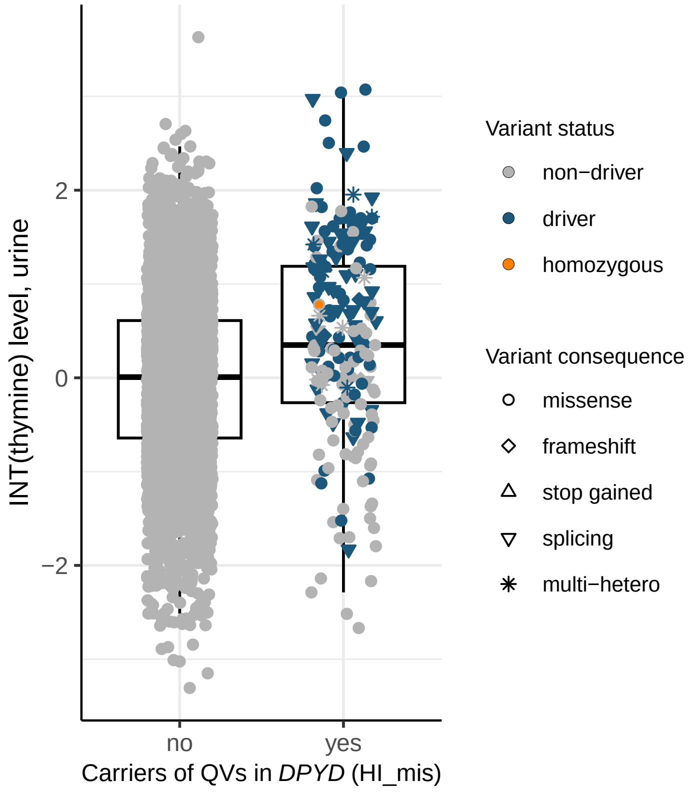

INT(thymine) level, urine
2
0
−2
no
yes
Carriers of QVs in DPYD (HI_mis)
Variant status
non−driver
driver
homozygous
Variant consequence
missense
frameshift
stop gained
splicing
multi−hetero

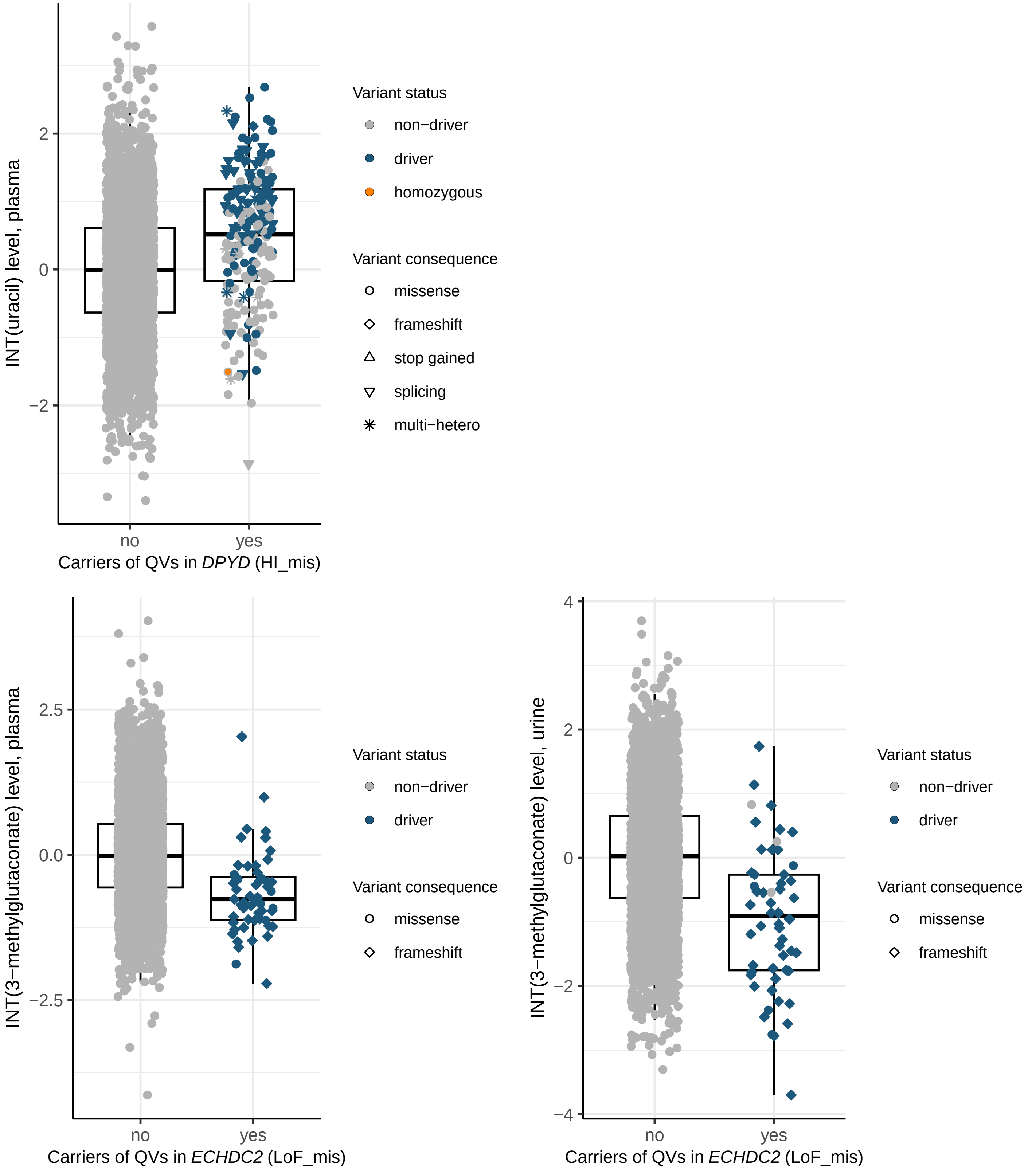

INT(uracil) level, plasma
2
0
−2
no
yes
Carriers of QVs in *DPYD* (HI_mis)
Variant status
non−driver
driver
homozygous
Variant consequence
missense
frameshift
stop gained
splicing
multi−hetero
INT(3−methylglutaconate) level, plasma
2.5
0.0
−2.5
no
yes
Carriers of QVs in *ECHDC2* (LoF_mis)
Variant status
non−driver
driver
Variant consequence
missense
frameshift
INT(3−methylglutaconate) level, urine
4
2
0
−2
−4
no
yes
Carriers of QVs in *ECHDC2* (LoF_mis)
Variant status
non−driver
driver
Variant consequence
missense
frameshift

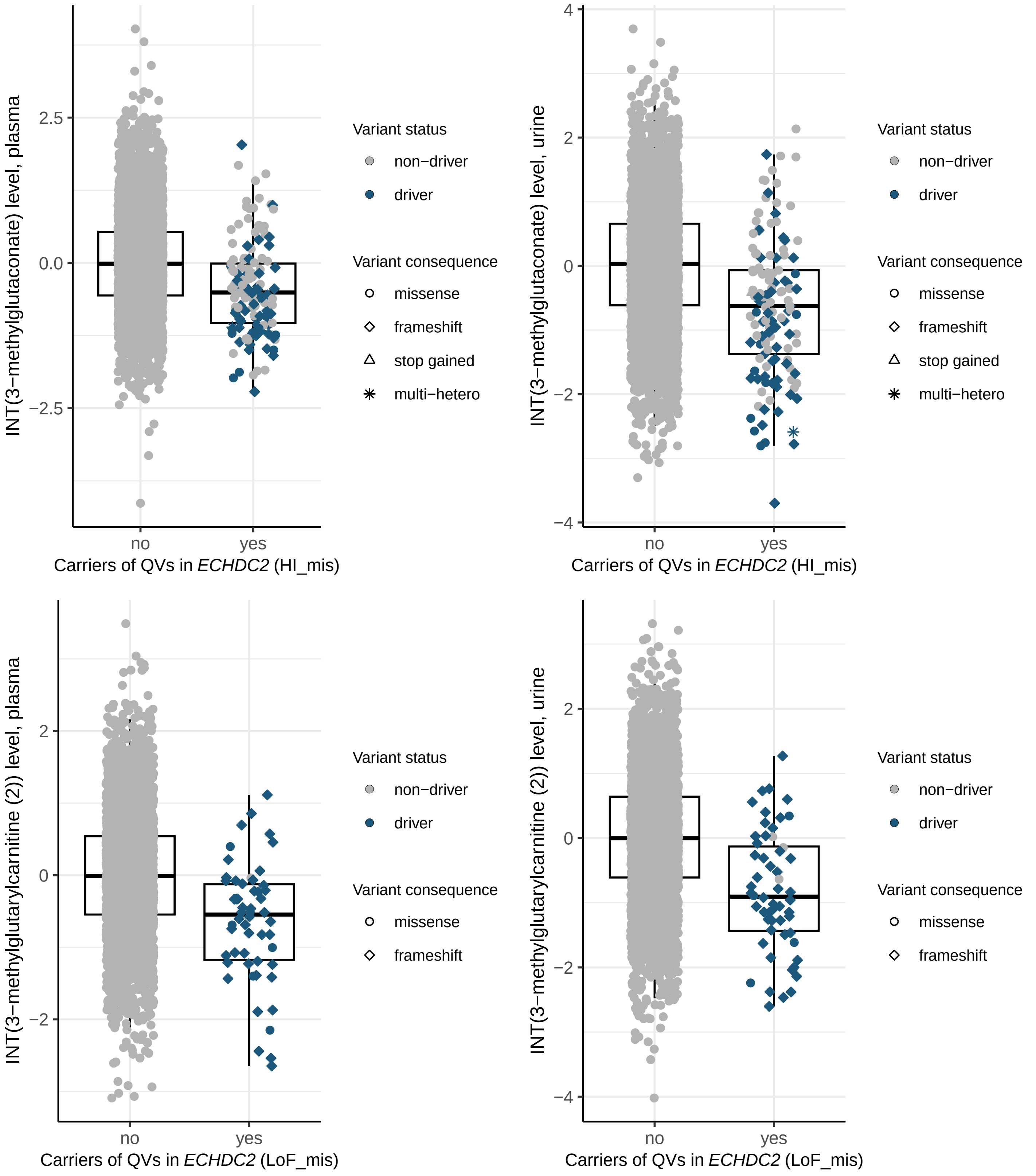
INT(3−methylglutaconate) level, plasma
2.5
0.0
−2.5
no
yes
Carriers of QVs in ECHDC2 (HI_mis)
INT(3−methylglutaconate) level, urine
4
2
0
−2
−4
no
yes
Carriers of QVs in ECHDC2 (HI_mis)
Variant status
non−driver
driver
Variant consequence
missense
frameshift
stop gained
multi−hetero
INT(3−methylglutarylcarnitine (2)) level, plasma
2
0
−2
no
yes
Carriers of QVs in ECHDC2 (LoF_mis)
INT(3−methylglutarylcarnitine (2)) level, urine
2
0
−2
−4
no
yes
Carriers of QVs in ECHDC2 (LoF_mis)
Variant status
non−driver
driver
Variant consequence
missense
frameshift

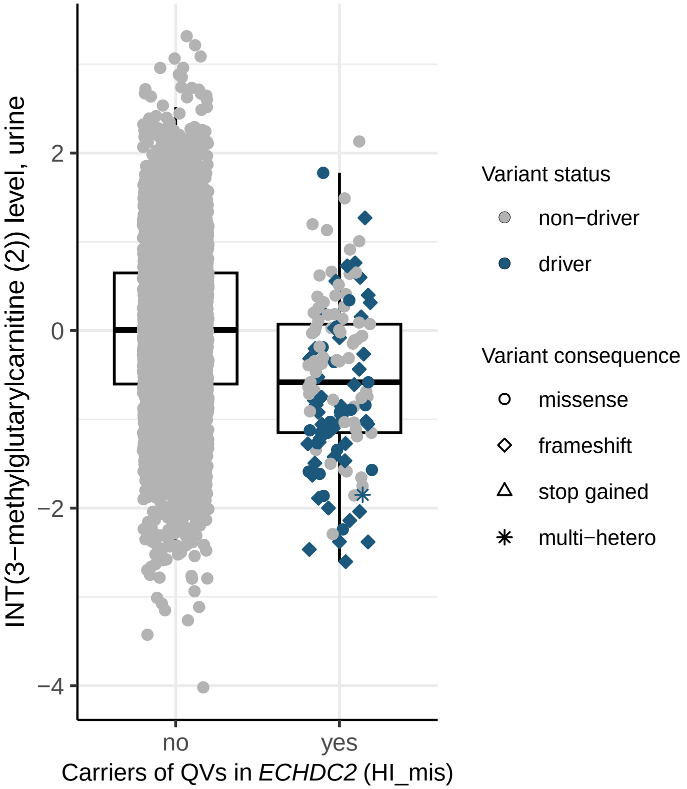
INT(3−methylglutarylcarnitine (2)) level, urine
2
0
−2
−4
no
yes
Carriers of QVs in *ECHDC2* (HI_mis)
Variant status
non−driver
driver
Variant consequence
missense
frameshift
stop gained
multi−hetero

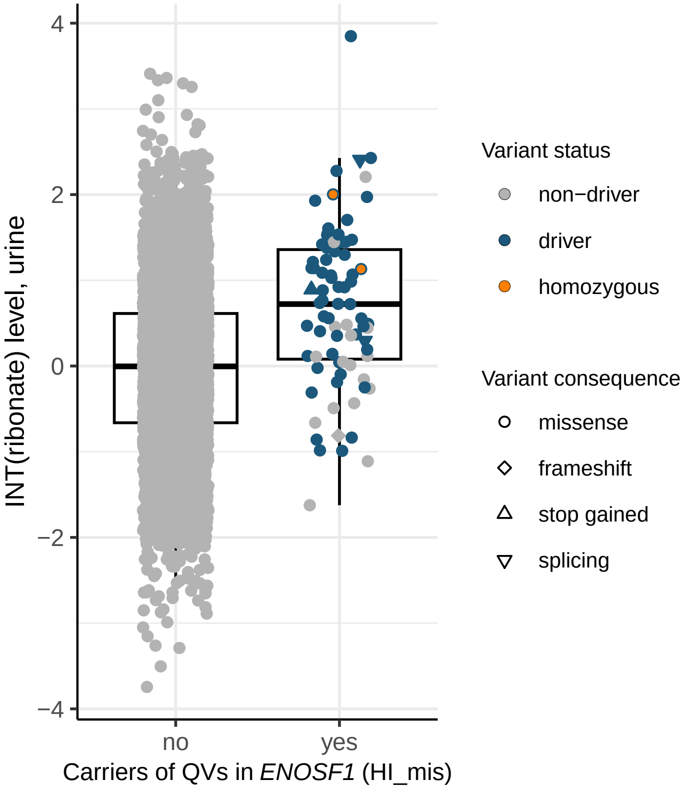
INT(ribonate) level, urine
4
2
0
−2
−4
no
yes
Carriers of QVs in *ENOSF1* (HI_mis)
Variant status
non−driver
driver
homozygous
Variant consequence
missense
frameshift
stop gained
splicing

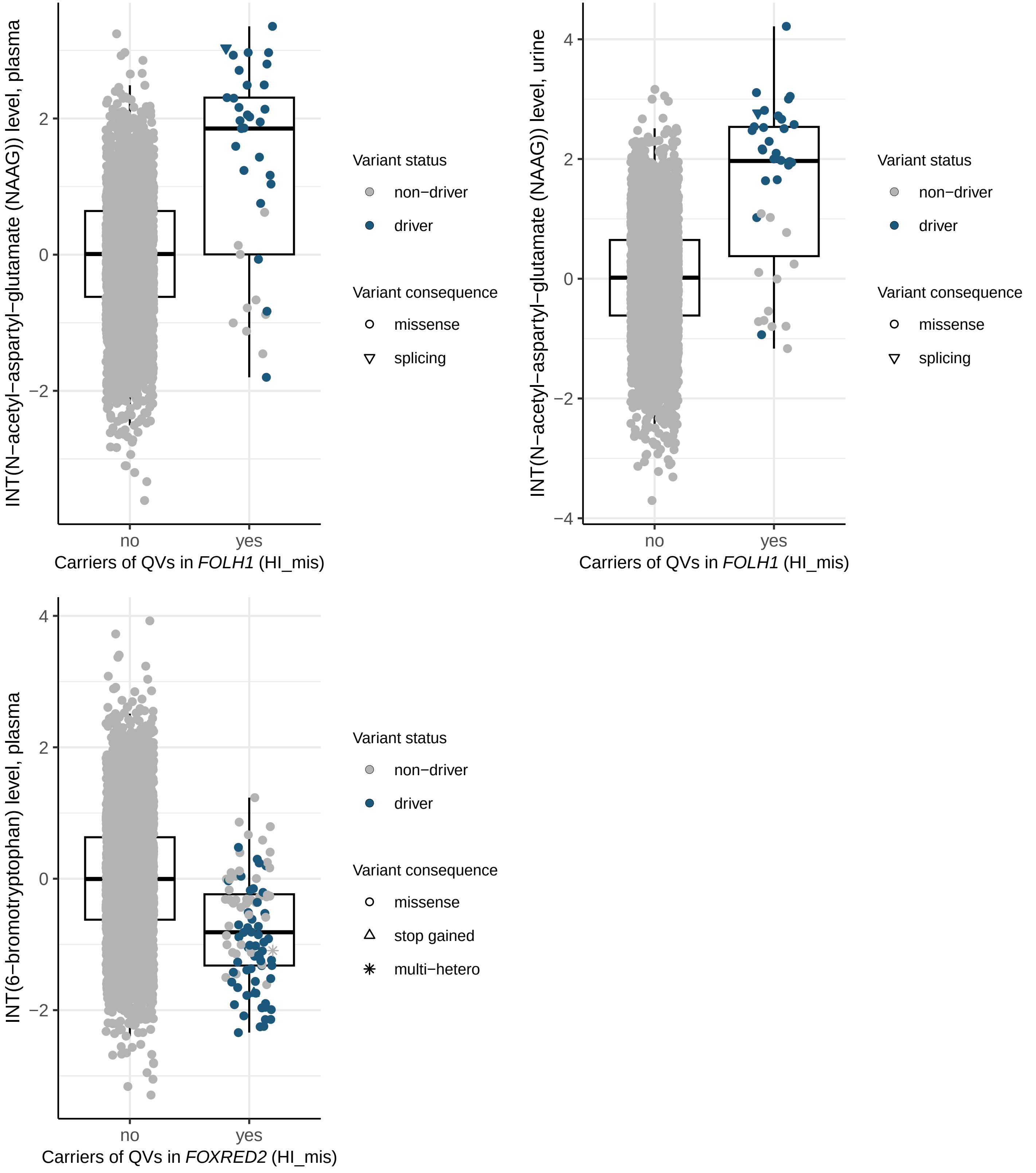

INT(N−acetyl−aspartyl−glutamate (NAAG)) level, plasma
2
0
−2
no
yes
Carriers of QVs in *FOLH1* (HI_mis)
Variant status
non−driver
driver
Variant consequence
missense
splicing
INT(N−acetyl−aspartyl−glutamate (NAAG)) level, urine
4
2
0
−2
−4
no
yes
Carriers of QVs in *FOLH1* (HI_mis)
Variant status
non−driver
driver
Variant consequence
missense
splicing
INT(6−bromotryptophan) level, plasma
4
2
0
−2
no
yes
Carriers of QVs in *FOXRED2* (HI_mis)
Variant status
non−driver
driver
Variant consequence
missense
stop gained
multi−hetero

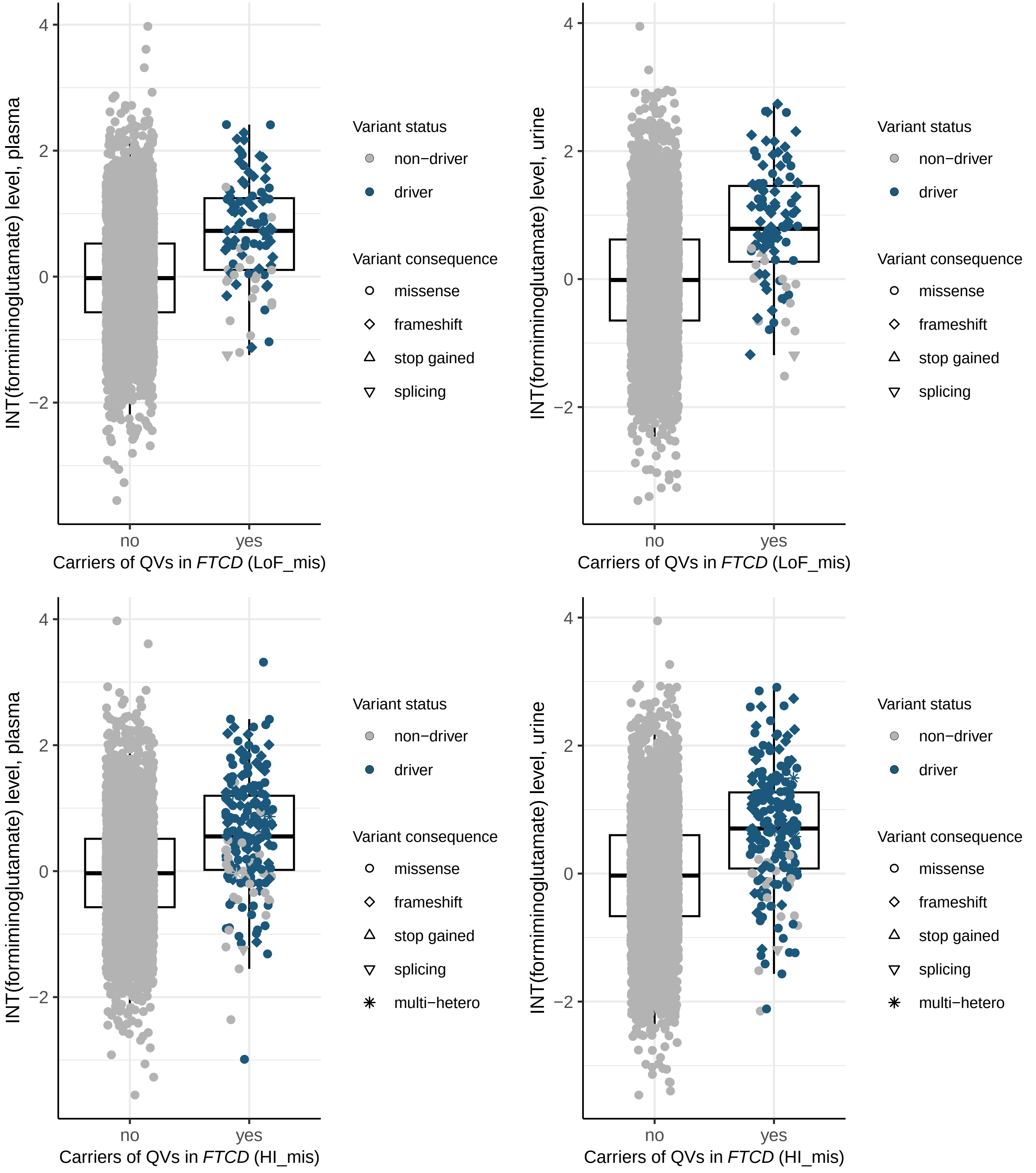
INT(formiminoglutamate) level, plasma
INT(formiminoglutamate) level, urine
4
2
0
−2
no
yes
Carriers of QVs in *FTCD* (LoF_mis)
Carriers of QVs in *FTCD* (HI_mis)
Variant status
non−driver
driver
Variant consequence
missense
frameshift
stop gained
splicing
multi−hetero

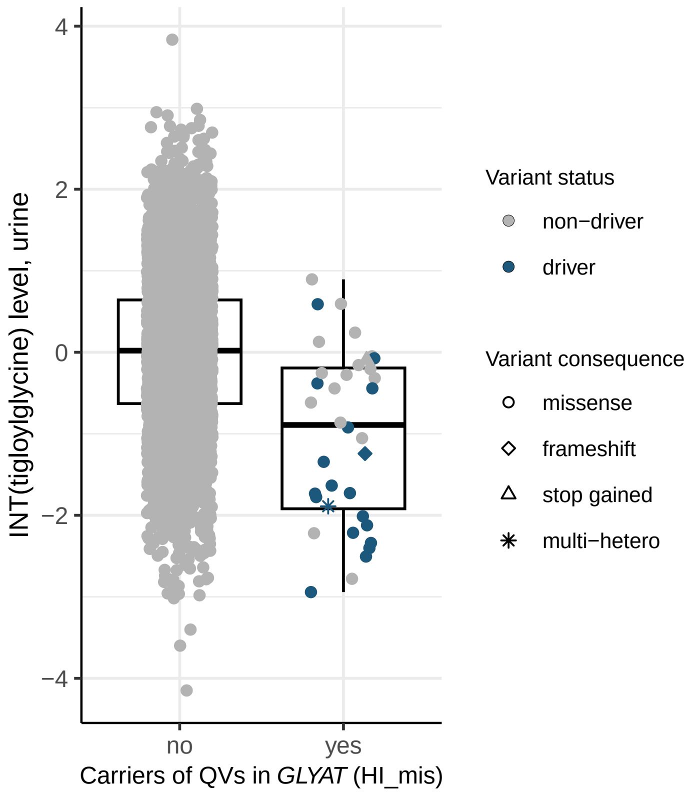

4
2
0
−2
−4
INT(tigloylglycine) level, urine
no
yes
Carriers of QVs in *GLYAT* (HI_mis)
Variant status
non−driver
driver
Variant consequence
missense
frameshift
stop gained
multi−hetero

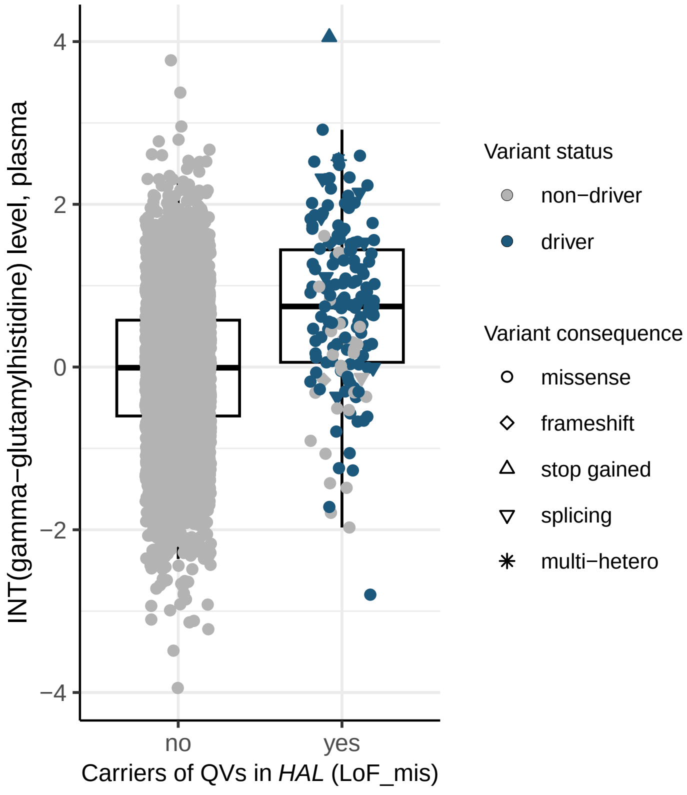

4
2
0
−2
−4
INT(gamma−glutamylhistidine) level, plasma
no
yes
Carriers of QVs in *HAL* (LoF_mis)
Variant status
non−driver
driver
Variant consequence
missense
frameshift
stop gained
splicing
multi−hetero

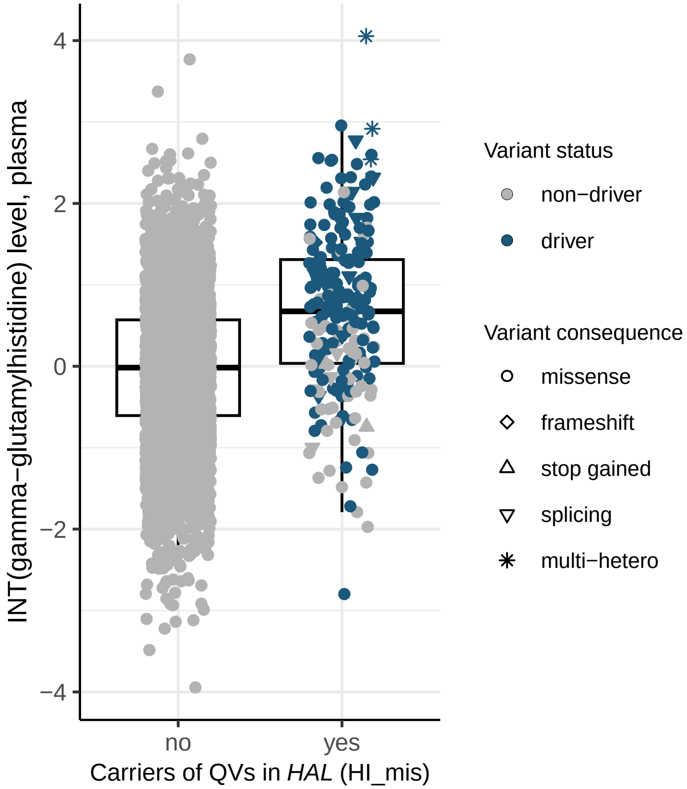

INT(gamma−glutamylhistidine) level, plasma
4
2
0
−2
−4
no
yes
Carriers of QVs in *HAL* (HI_mis)
Variant status
non−driver
driver
Variant consequence
missense
frameshift
stop gained
splicing
multi−hetero

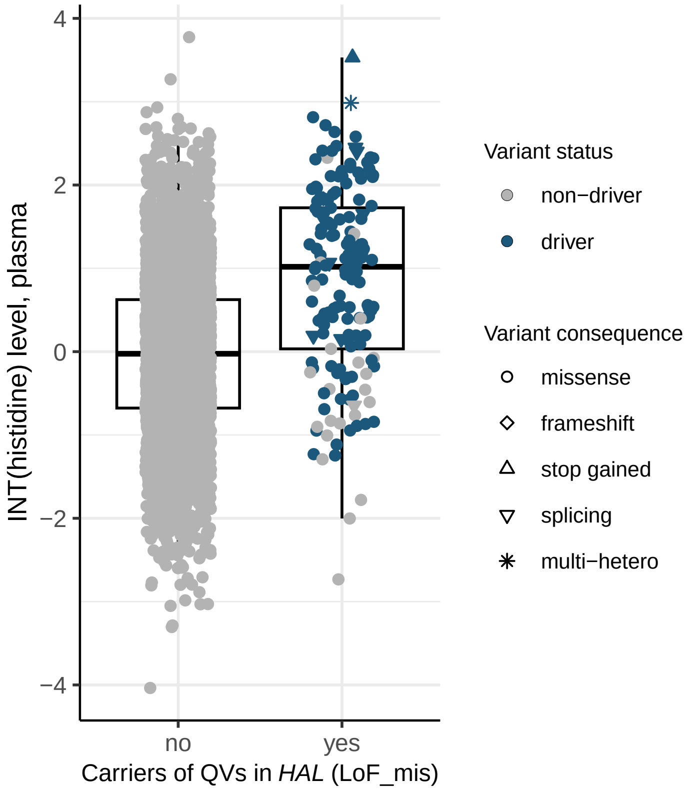

INT(histidine) level, plasma
4
2
0
−2
−4
no
yes
Carriers of QVs in *HAL* (LoF_mis)
Variant status
non−driver
driver
Variant consequence
missense
frameshift
stop gained
splicing
multi−hetero

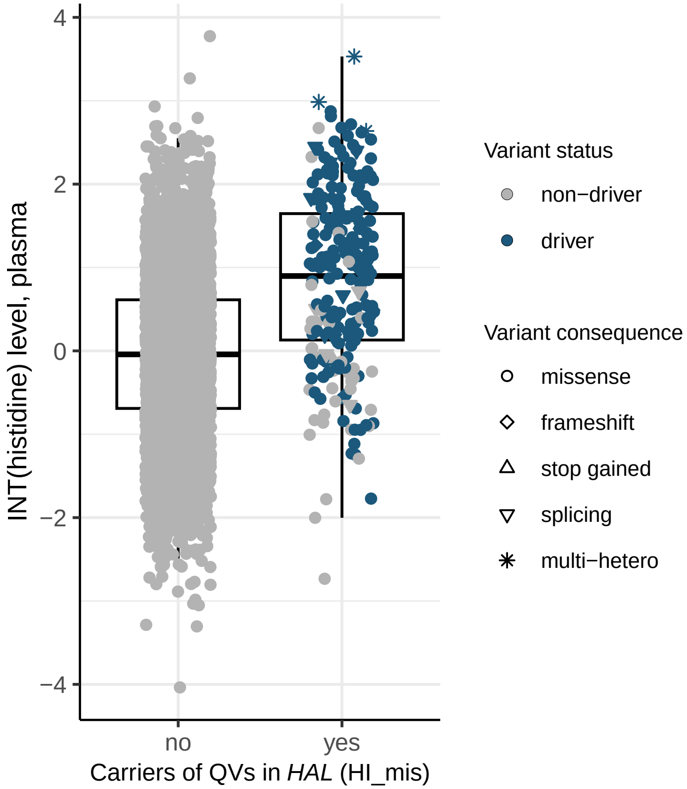

INT(histidine) level, plasma
4
2
0
−2
−4
no
yes
Carriers of QVs in *HAL* (HI_mis)
Variant status
non−driver
driver
Variant consequence
missense
frameshift
stop gained
splicing
multi−hetero

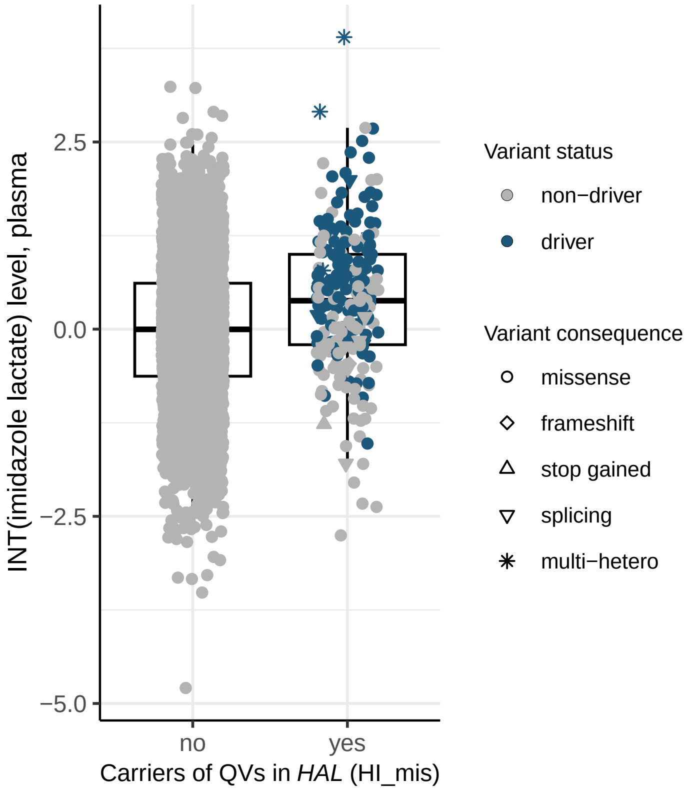

INT(imidazole lactate) level, plasma
2.5
0.0
−2.5
−5.0
no
yes
Carriers of QVs in *HAL* (HI_mis)
Variant status
non−driver
driver
Variant consequence
missense
frameshift
stop gained
splicing
multi−hetero

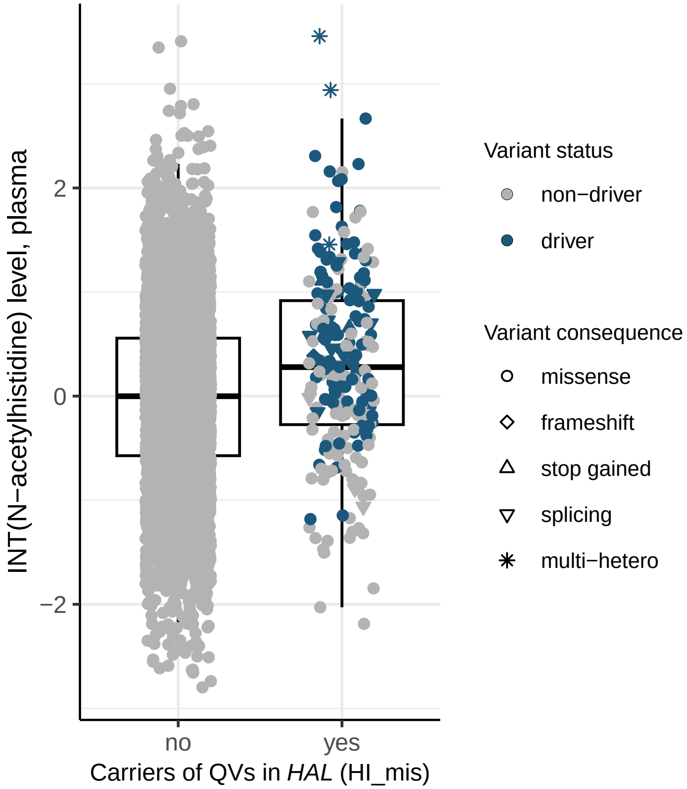

INT(N−acetylhistidine) level, plasma
2
0
−2
no
yes
Carriers of QVs in *HAL* (HI_mis)
Variant status
non−driver
driver
Variant consequence
missense
frameshift
stop gained
splicing
multi−hetero

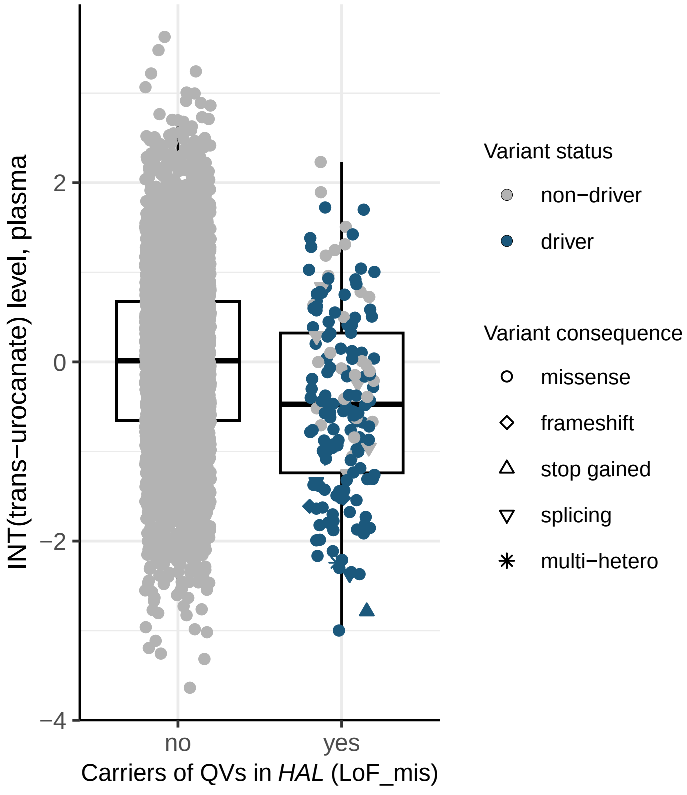

INT(trans−urocanate) level, plasma
2
0
−2
−4
no
yes
Carriers of QVs in *HAL* (LoF_mis)
Variant status
non−driver
driver
Variant consequence
missense
frameshift
stop gained
splicing
multi−hetero

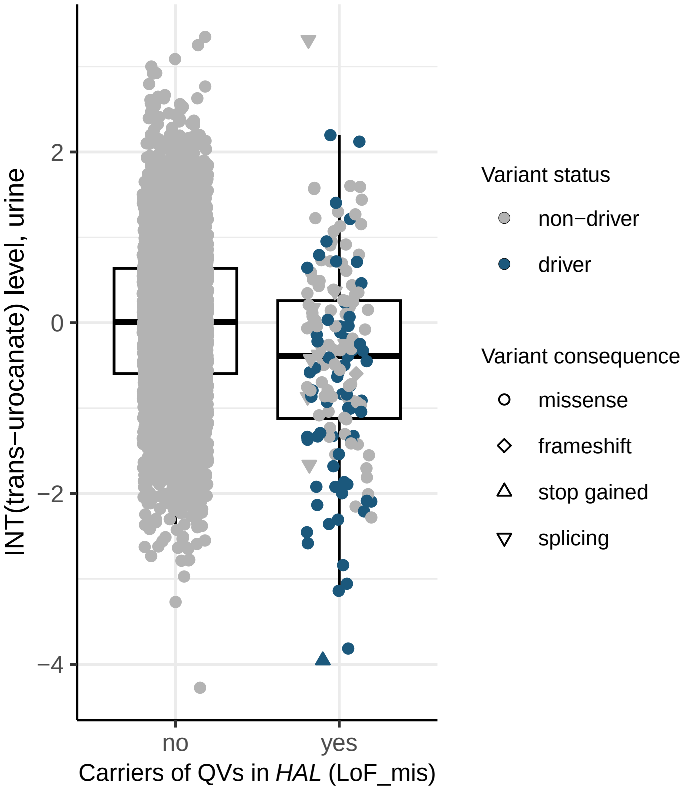

INT(trans−urocanate) level, urine
2
0
−2
−4
no
yes
Carriers of QVs in *HAL* (LoF_mis)
Variant status
non−driver
driver
Variant consequence
missense
frameshift
stop gained
splicing

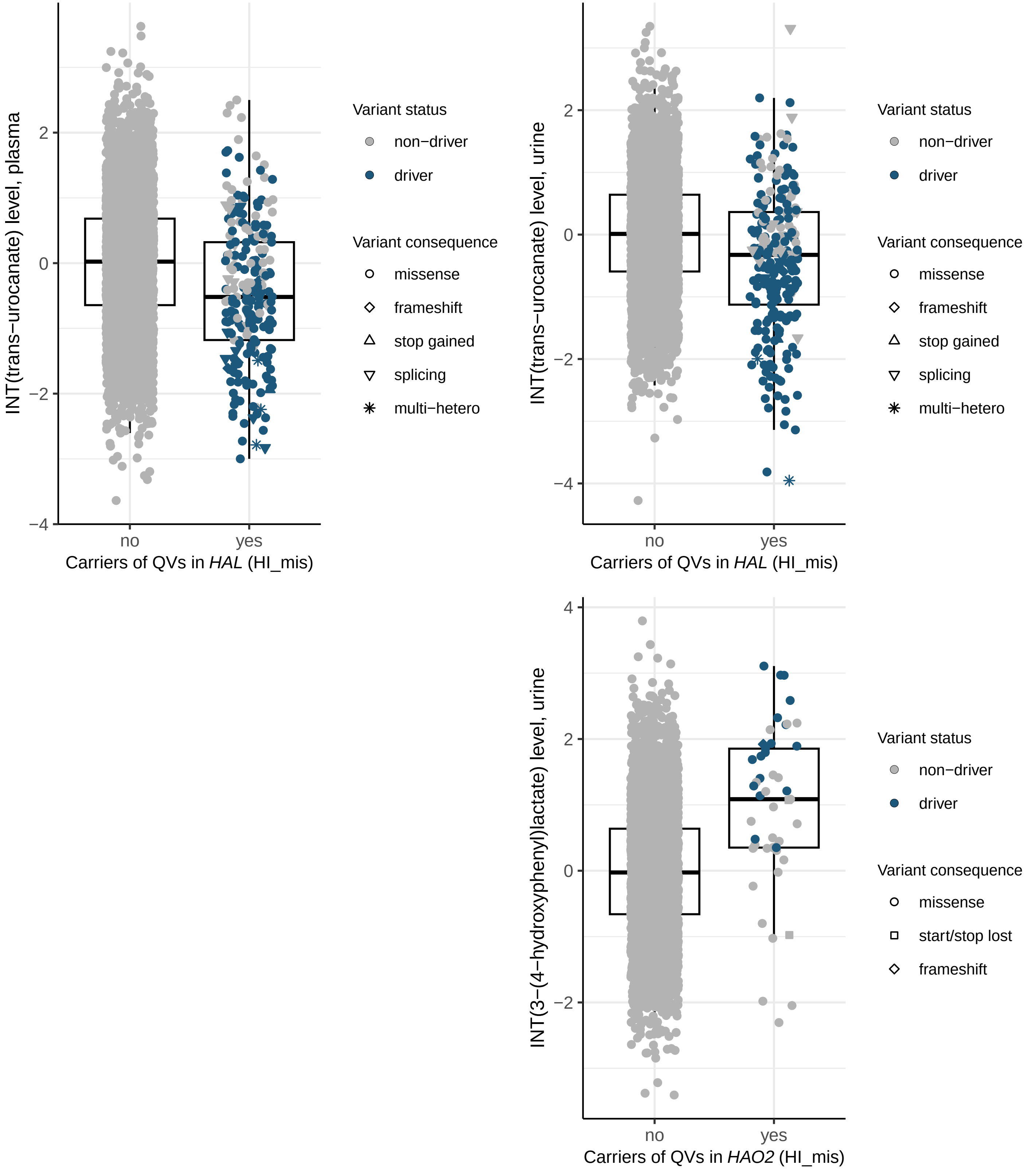

INT(trans−urocanate) level, plasma
Carriers of QVs in HAL (HI_mis)
no
yes
Variant status
non−driver
driver
Variant consequence
missense
frameshift
stop gained
splicing
multi−hetero
INT(trans−urocanate) level, urine
Carriers of QVs in HAL (HI_mis)
INT(3−(4−hydroxyphenyl)lactate) level, urine
Carriers of QVs in HAO2 (HI_mis)
start/stop lost

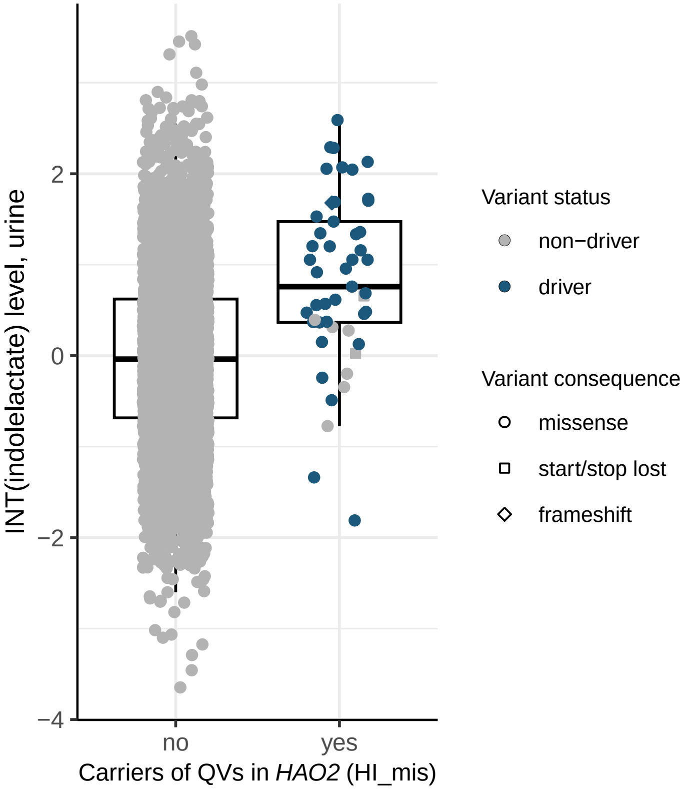

INT(indolelactate) level, urine
2
0
−2
−4
no
yes
Carriers of QVs in *HAO2* (HI_mis)
Variant status
non−driver
driver
Variant consequence
missense
start/stop lost
frameshift

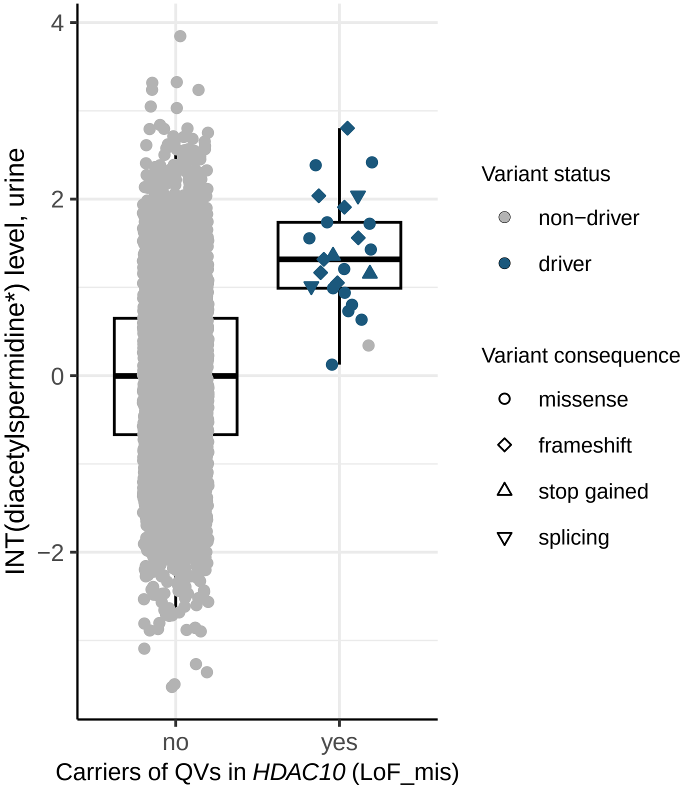

INT(diacetylspermidine*) level, urine
4
2
0
−2
no
yes
Carriers of QVs in *HDAC10* (LoF_mis)
Variant status
non−driver
driver
Variant consequence
missense
frameshift
stop gained
splicing

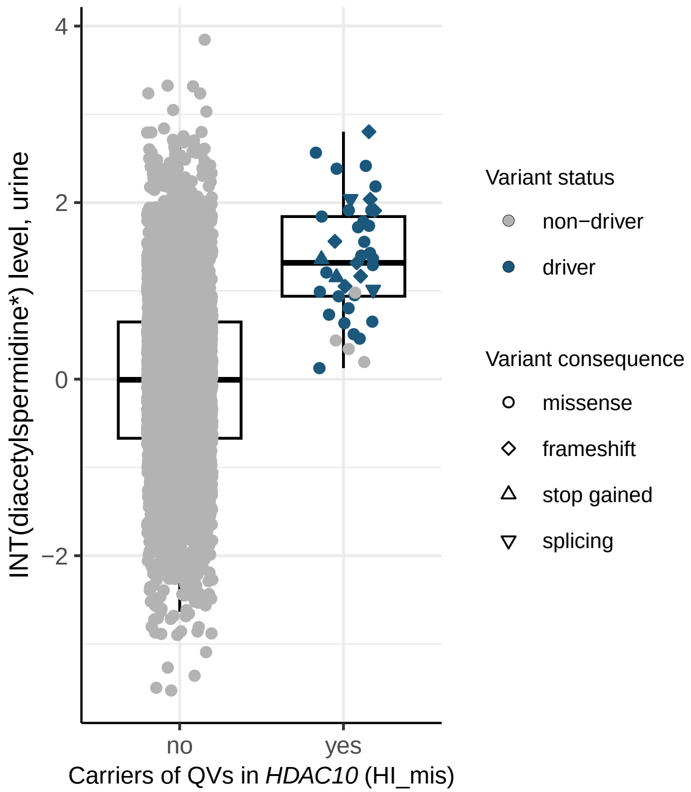
4
2
0
−2
INT(diacetylspermidine*) level, urine
no
yes
Carriers of QVs in *HDAC10* (HI_mis)
Variant status
non−driver
driver
Variant consequence
missense
frameshift
stop gained
splicing

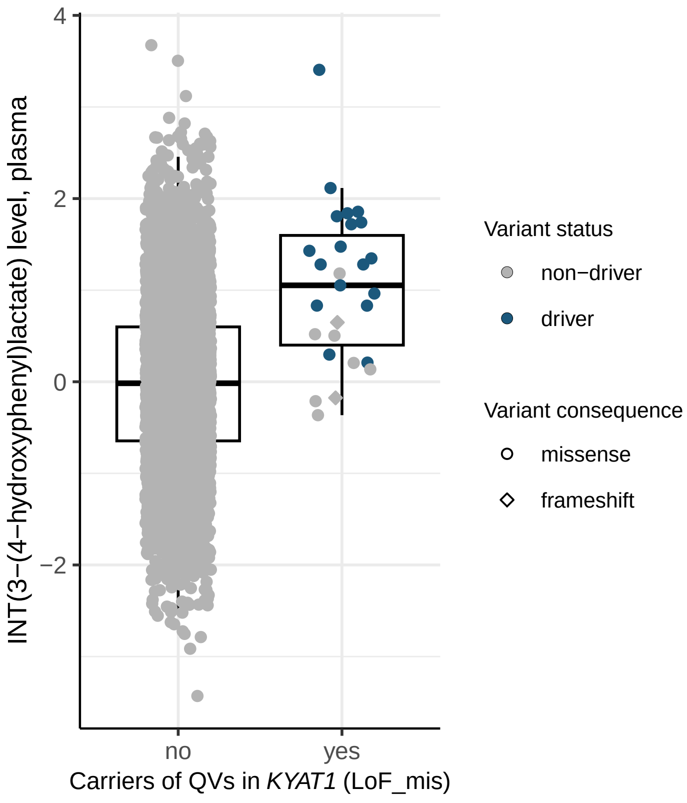
4
2
0
−2
INT(3−(4−hydroxyphenyl)lactate) level, plasma
no
yes
Carriers of QVs in *KYAT1* (LoF_mis)
Variant status
non−driver
driver
Variant consequence
missense
frameshift

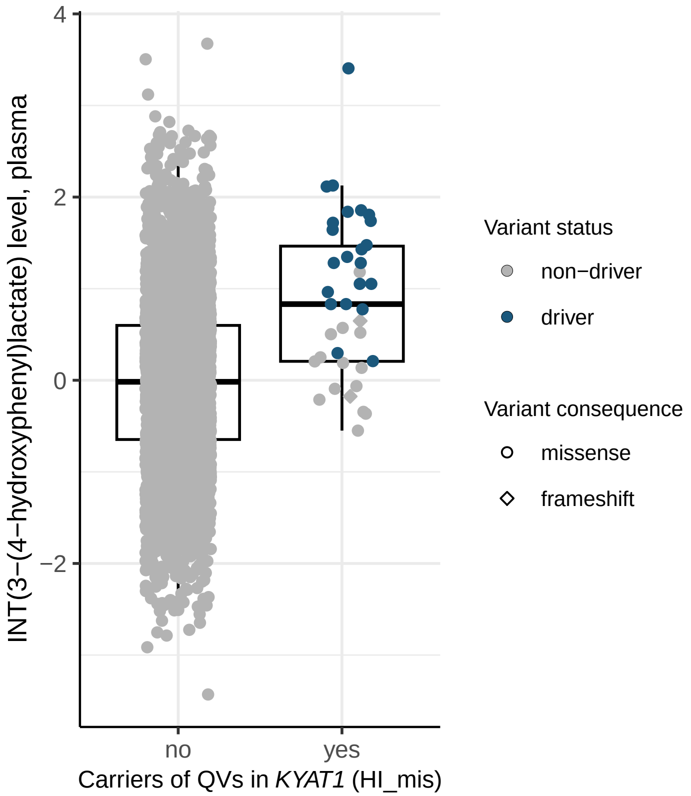

4
2
0
−2
INT(3−(4−hydroxyphenyl)lactate) level, plasma
no
yes
Carriers of QVs in *KYAT1* (HI_mis)
Variant status
non−driver
driver
Variant consequence
missense
frameshift

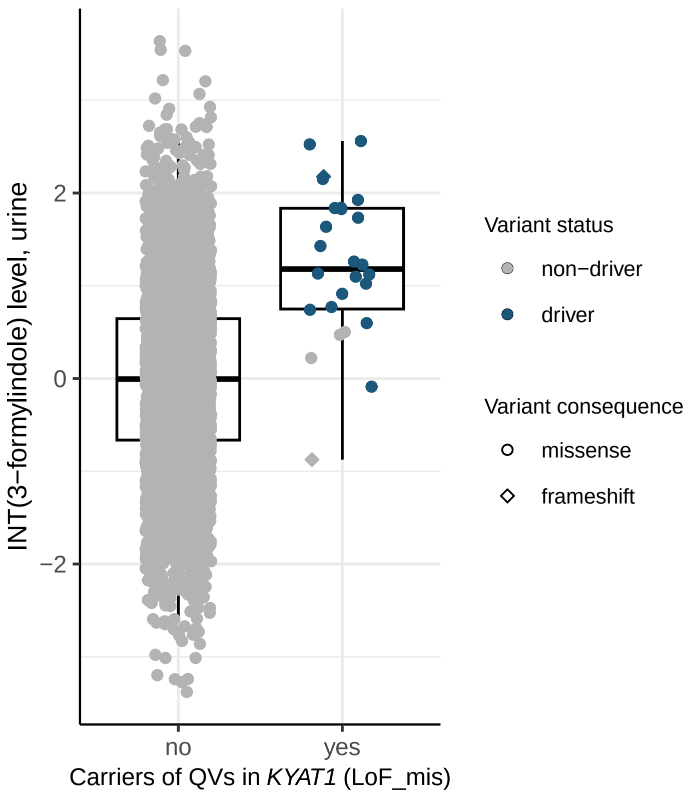

2
0
−2
INT(3−formylindole) level, urine
no
yes
Carriers of QVs in *KYAT1* (LoF_mis)
Variant status
non−driver
driver
Variant consequence
missense
frameshift

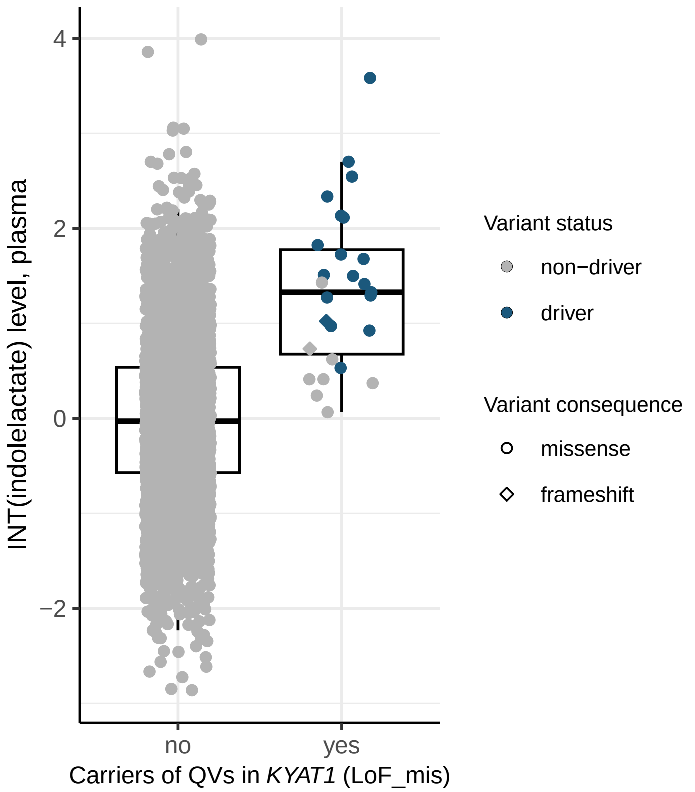
INT(indolelactate) level, plasma
4
2
0
−2
no
yes
Carriers of QVs in *KYAT1* (LoF_mis)
Variant status
non−driver
driver
Variant consequence
missense
frameshift

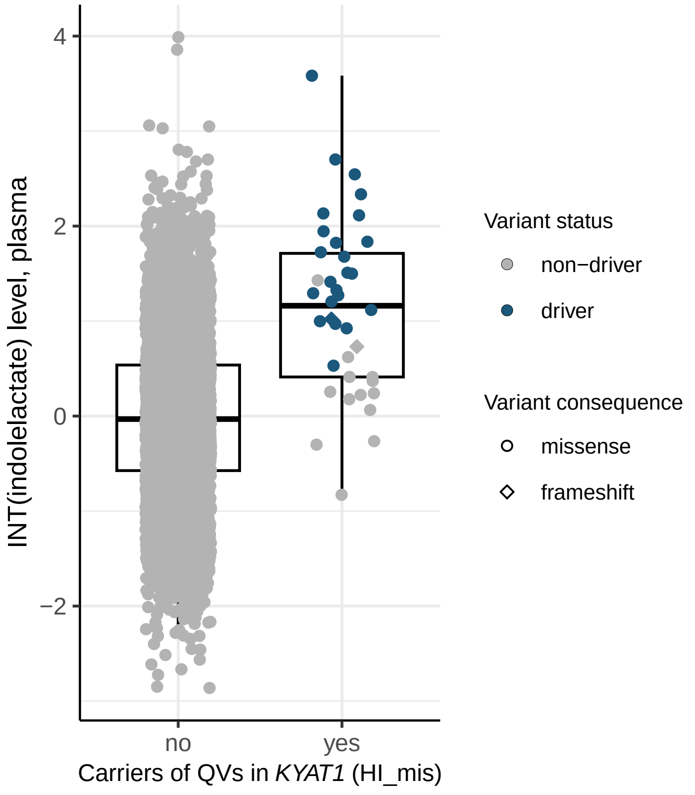
INT(indolelactate) level, plasma
4
2
0
−2
no
yes
Carriers of QVs in *KYAT1* (HI_mis)
Variant status
non−driver
driver
Variant consequence
missense
frameshift

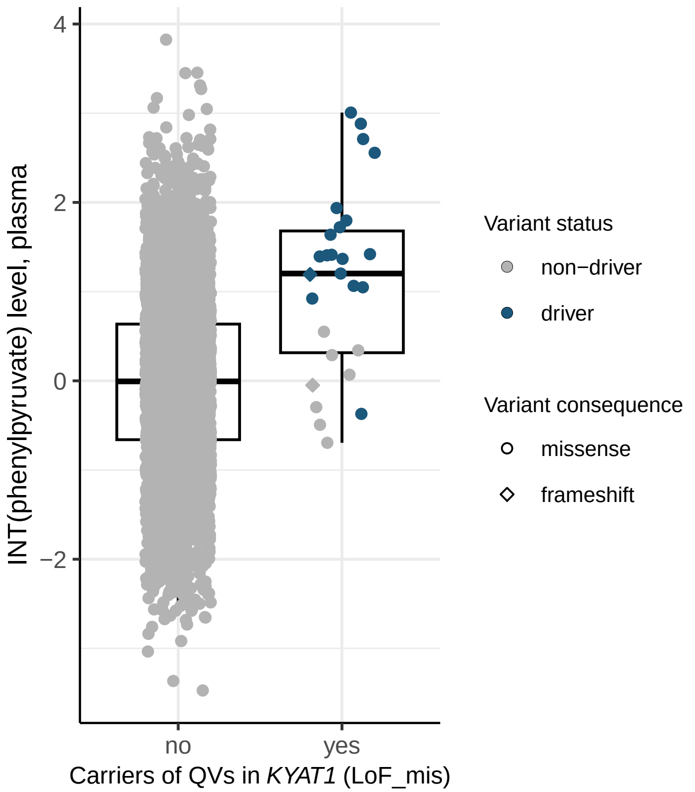
INT(phenylpyruvate) level, plasma
4
2
0
−2
no
yes
Carriers of QVs in *KYAT1* (LoF_mis)
Variant status
non−driver
driver
Variant consequence
missense
frameshift

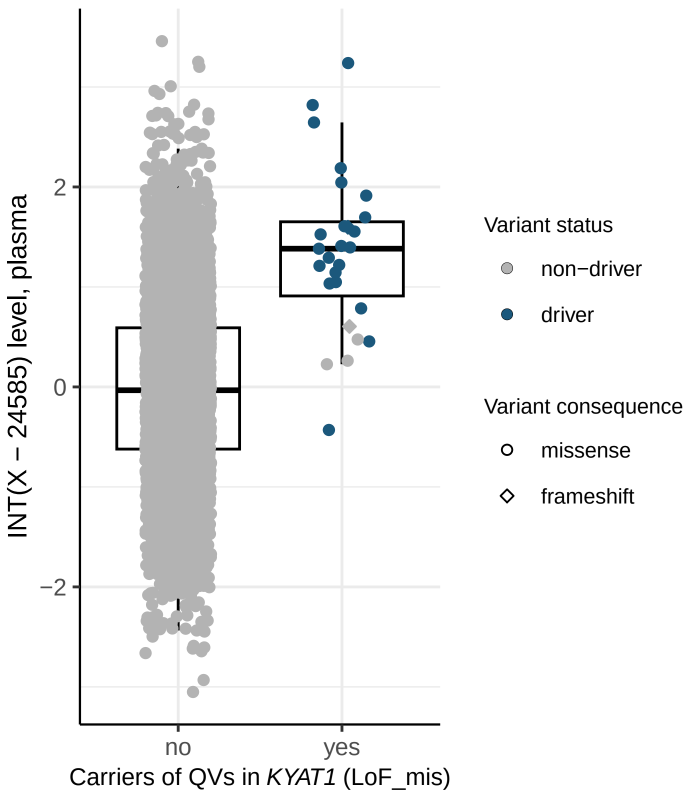
INT(X − 24585) level, plasma
2
0
−2
no
yes
Carriers of QVs in *KYAT1* (LoF_mis)
Variant status
non−driver
driver
Variant consequence
missense
frameshift

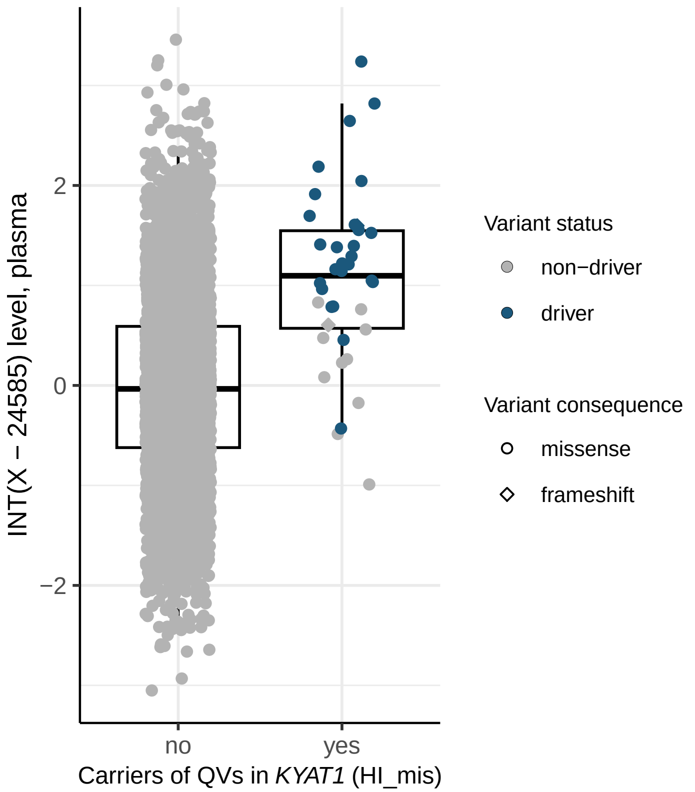
INT(X − 24585) level, plasma
2
0
−2
no
yes
Carriers of QVs in *KYAT1* (HI_mis)
Variant status
non−driver
driver
Variant consequence
missense
frameshift

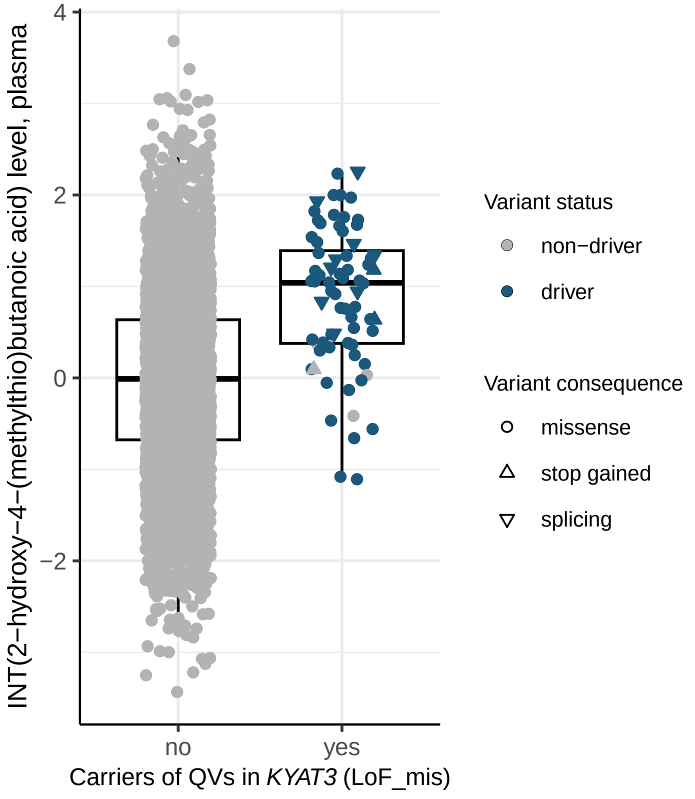
INT(2−hydroxy−4−(methylthio)butanoic acid) level, plasma
4
2
0
−2
no
yes
Carriers of QVs in *KYAT3* (LoF_mis)
Variant status
non−driver
driver
Variant consequence
missense
stop gained
splicing

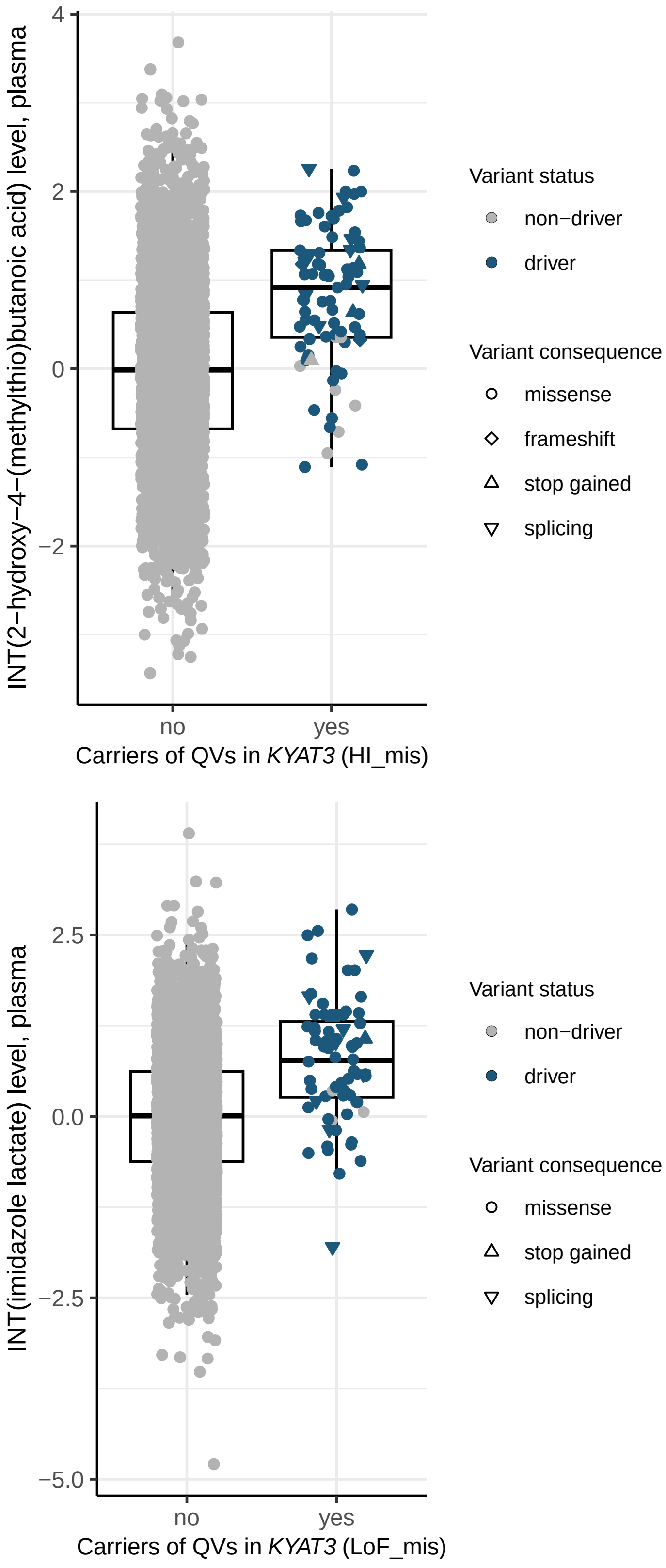

INT(2−hydroxy−4−(methylthio)butanoic acid) level, plasma
4
2
0
−2
no
yes
Carriers of QVs in KYAT3 (HI_mis)
Variant status
non−driver
driver
Variant consequence
missense
frameshift
stop gained
splicing
INT(imidazole lactate) level, plasma
2.5
0.0
−2.5
−5.0
no
yes
Carriers of QVs in KYAT3 (LoF_mis)
Variant status
non−driver
driver
Variant consequence
missense
stop gained
splicing

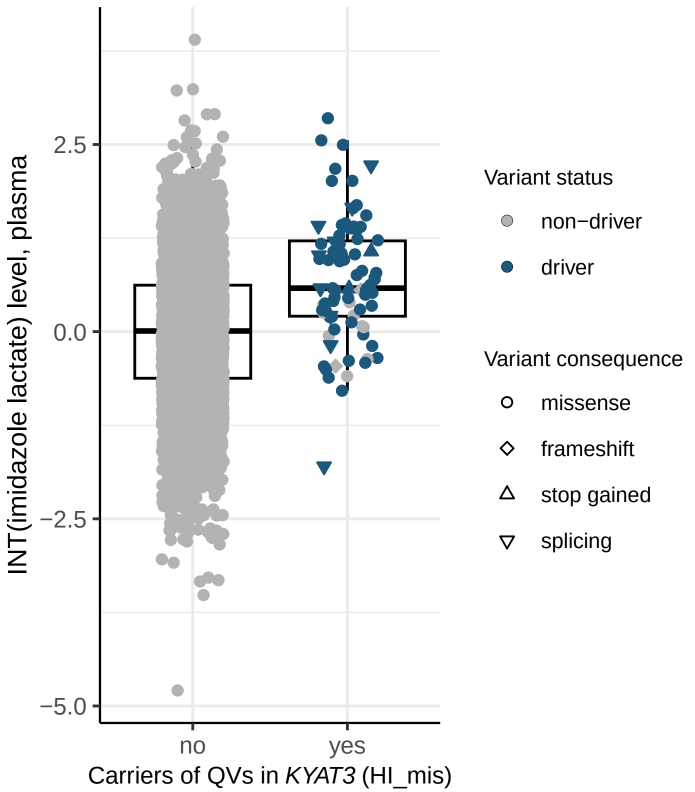

2.5
0.0
−2.5
−5.0
INT(imidazole lactate) level, plasma
no
yes
Carriers of QVs in KYAT3 (HI_mis)
Variant status
non−driver
driver
Variant consequence
missense
frameshift
stop gained
splicing

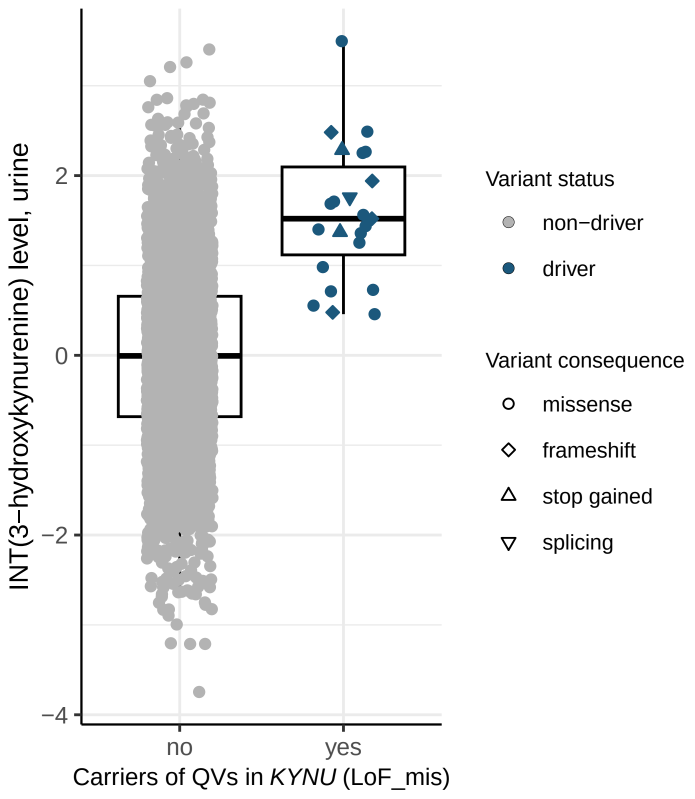

2
0
−2
−4
INT(3−hydroxykynurenine) level, urine
no
yes
Carriers of QVs in KYNU (LoF_mis)
Variant status
non−driver
driver
Variant consequence
missense
frameshift
stop gained
splicing

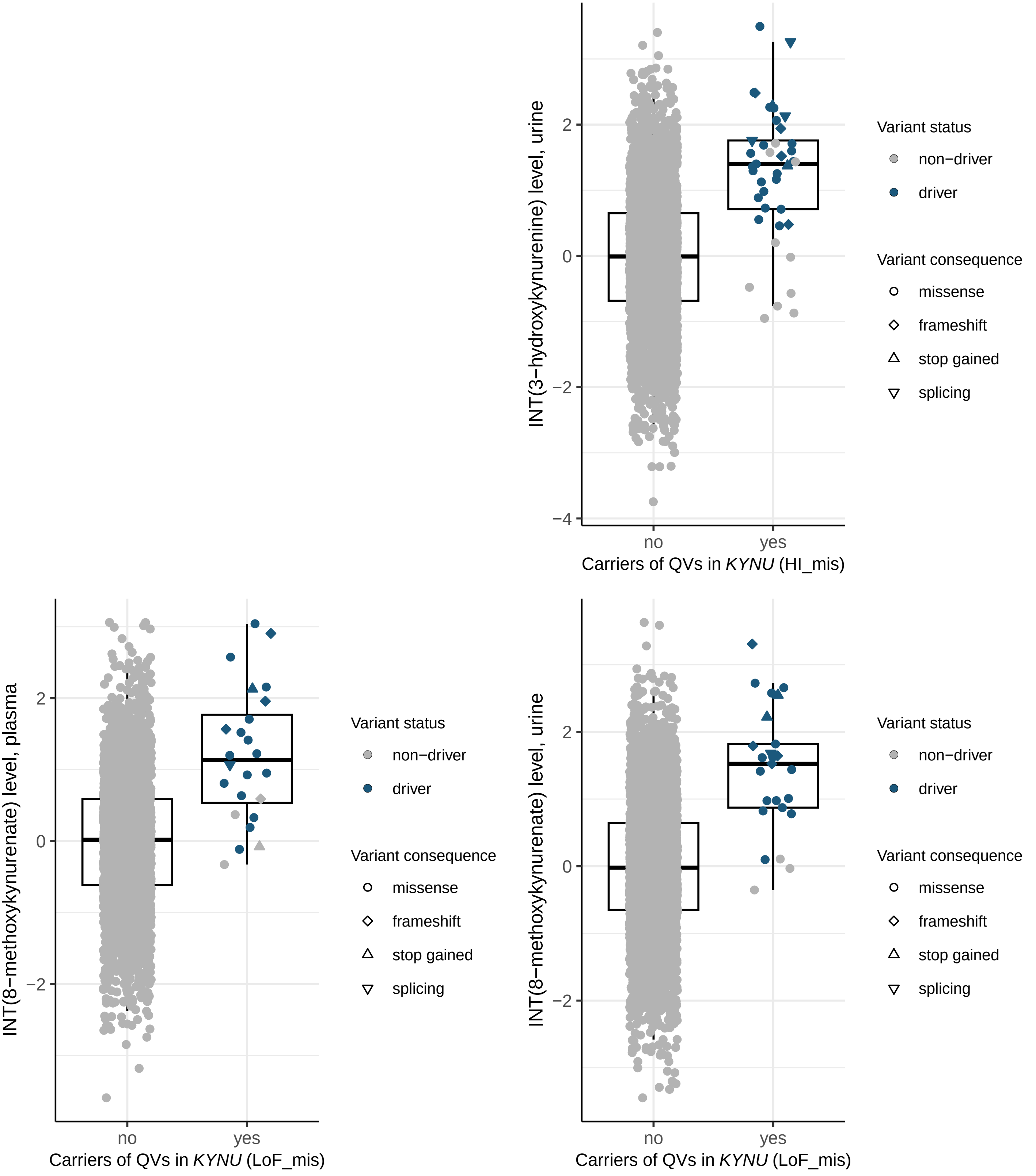

INT(3−hydroxykynurenine) level, urine
2
0
−2
−4
no
yes
Carriers of QVs in KYNU (HI_mis)
Variant status
non−driver
driver
Variant consequence
missense
frameshift
stop gained
splicing
INT(8−methoxykynurenate) level, plasma
2
0
−2
no
yes
Carriers of QVs in KYNU (LoF_mis)
Variant status
non−driver
driver
Variant consequence
missense
frameshift
stop gained
splicing
INT(8−methoxykynurenate) level, urine
2
0
−2
no
yes
Carriers of QVs in KYNU (LoF_mis)
Variant status
non−driver
driver
Variant consequence
missense
frameshift
stop gained
splicing

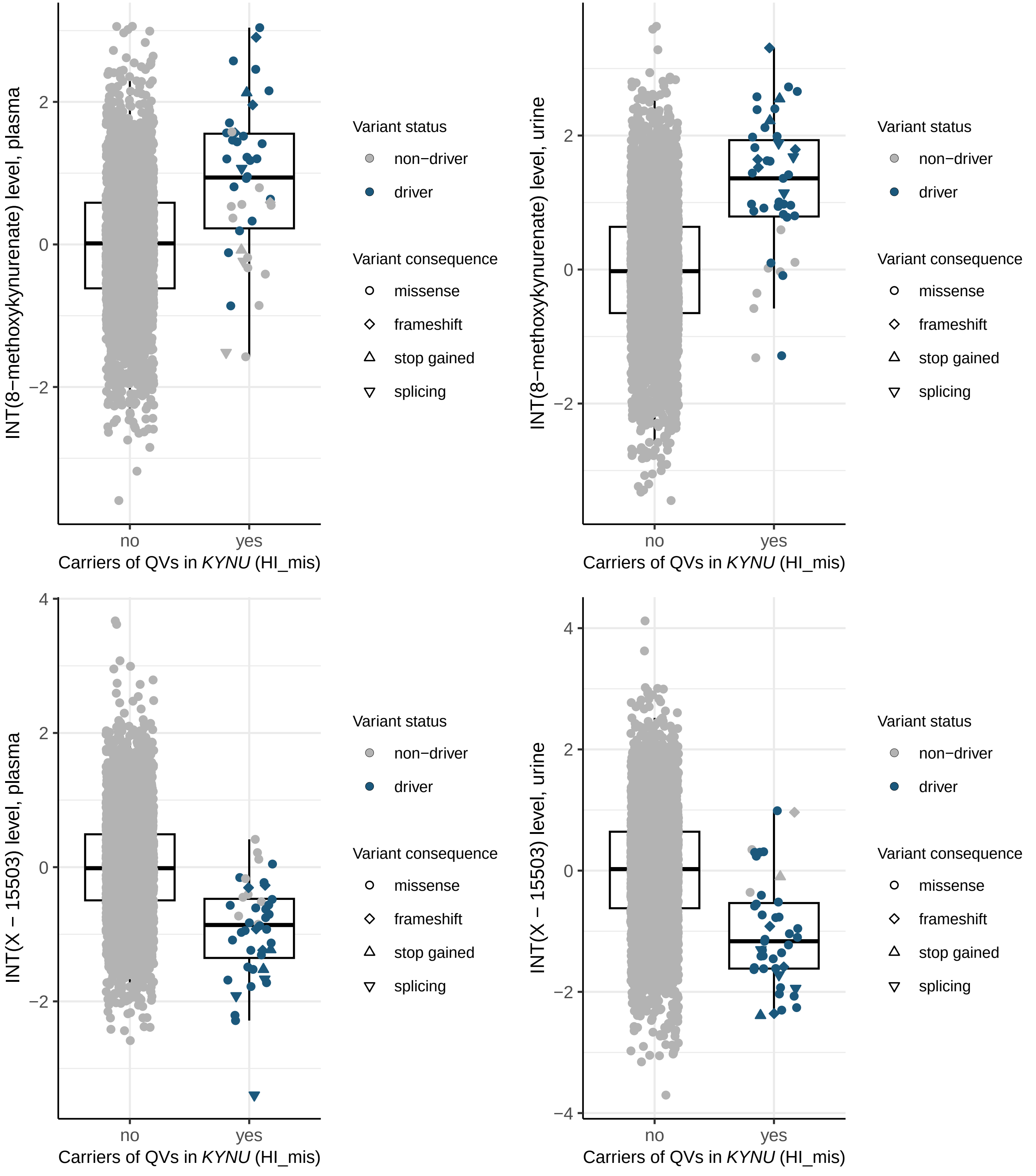
INT(8−methoxykynurenate) level, plasma
INT(8−methoxykynurenate) level, urine
INT(X − 15503) level, plasma
INT(X − 15503) level, urine
Carriers of QVs in *KYNU* (HI_mis)
no
yes
Variant status
non−driver
driver
Variant consequence
missense
frameshift
stop gained
splicing

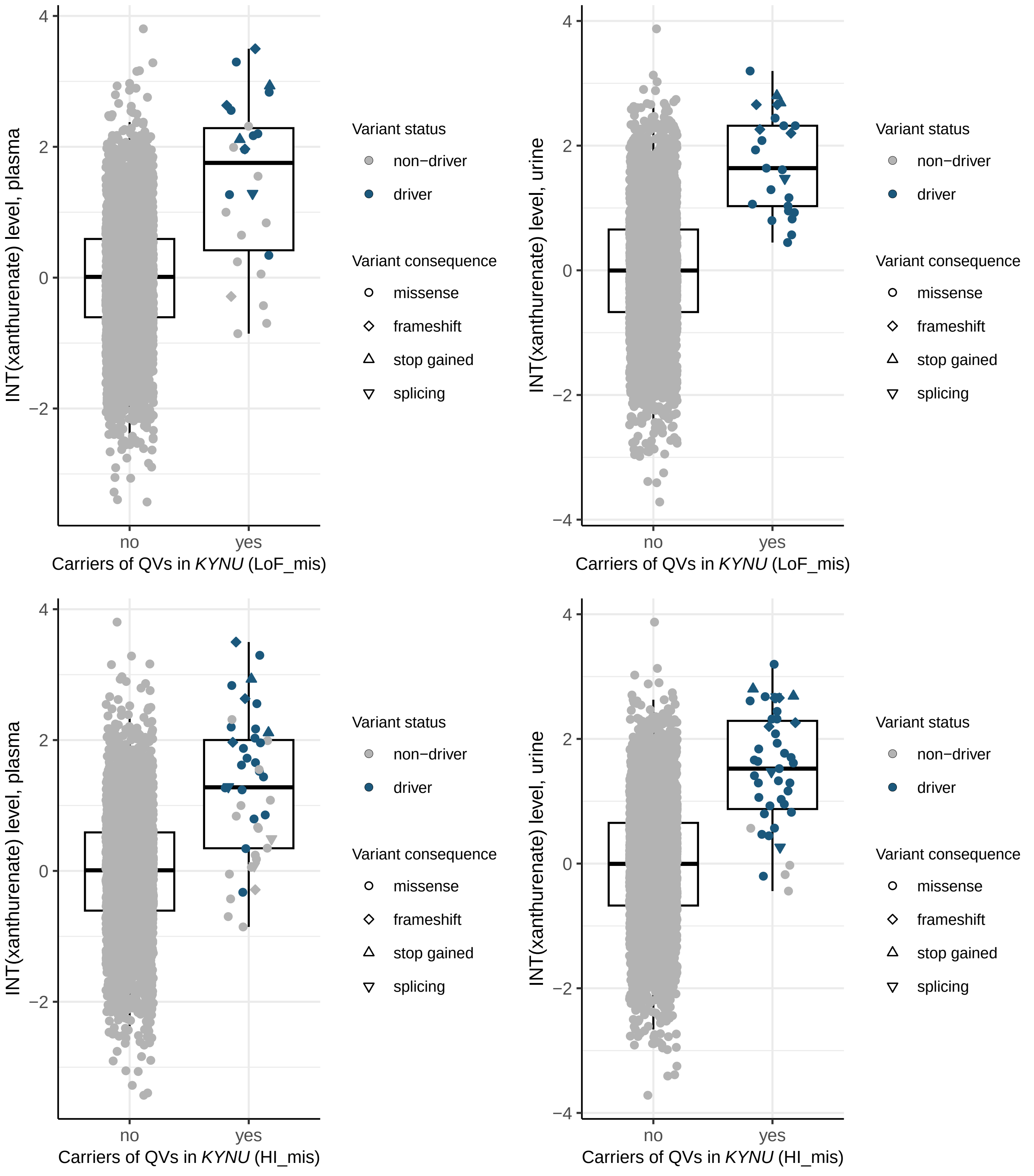
INT(xanthurenate) level, plasma
Carriers of QVs in KYNU (LoF_mis)
INT(xanthurenate) level, urine
Carriers of QVs in KYNU (LoF_mis)
INT(xanthurenate) level, plasma
Carriers of QVs in KYNU (HI_mis)
INT(xanthurenate) level, urine
Carriers of QVs in KYNU (HI_mis)
no
yes
4
2
0
−2
−4
Variant status
non−driver
driver
Variant consequence
missense
frameshift
stop gained
splicing

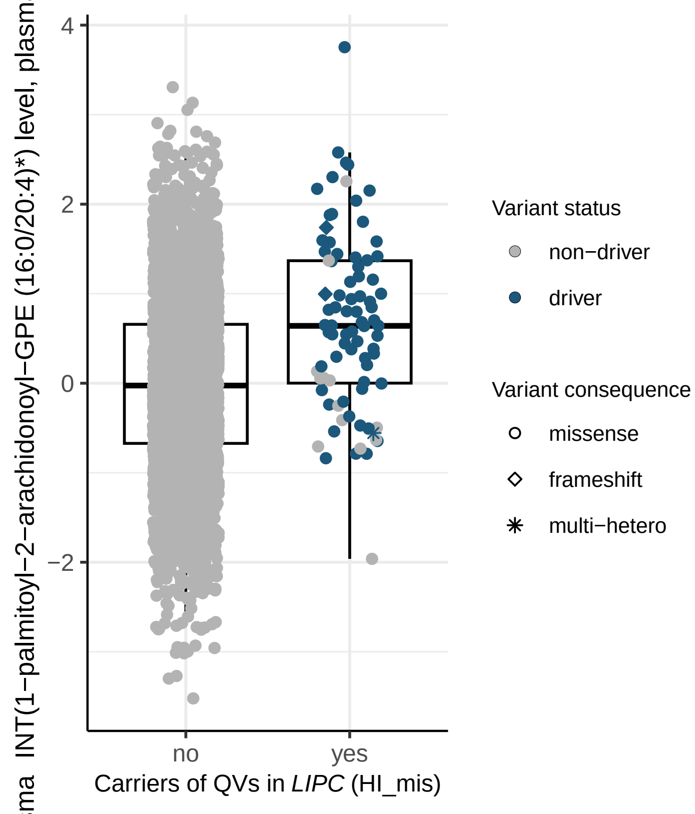
INT(1−palmitoyl−2−arachidonoyl−GPE (16:0/20:4)*) level, plasm
4
2
0
−2
no
yes
Carriers of QVs in *LIPC* (HI_mis)
Variant status
non−driver
driver
Variant consequence
missense
frameshift
multi−hetero

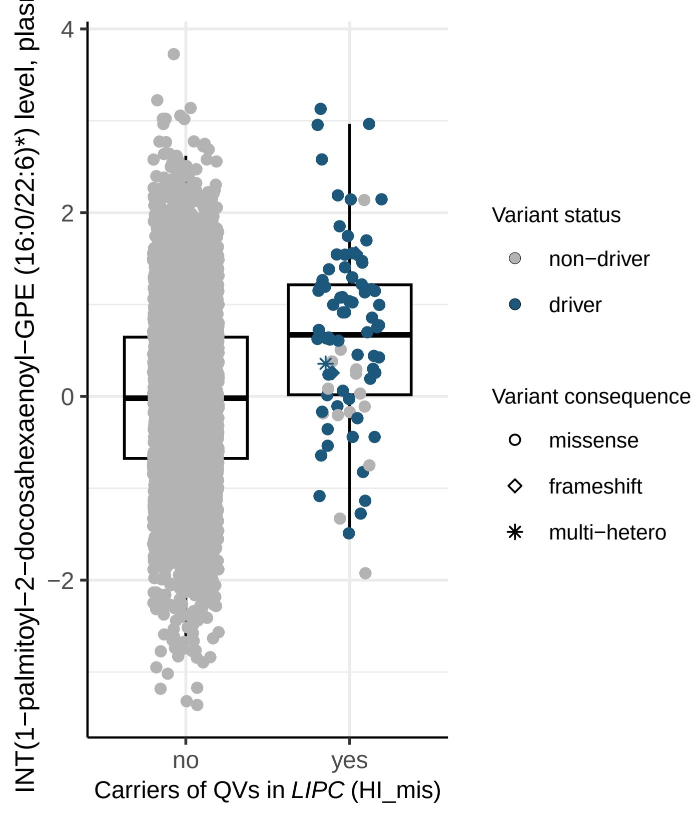
INT(1−palmitoyl−2−docosahexaenoyl−GPE (16:0/22:6)*) level, plasma
4
2
0
−2
no
yes
Carriers of QVs in *LIPC* (HI_mis)
Variant status
non−driver
driver
Variant consequence
missense
frameshift
multi−hetero

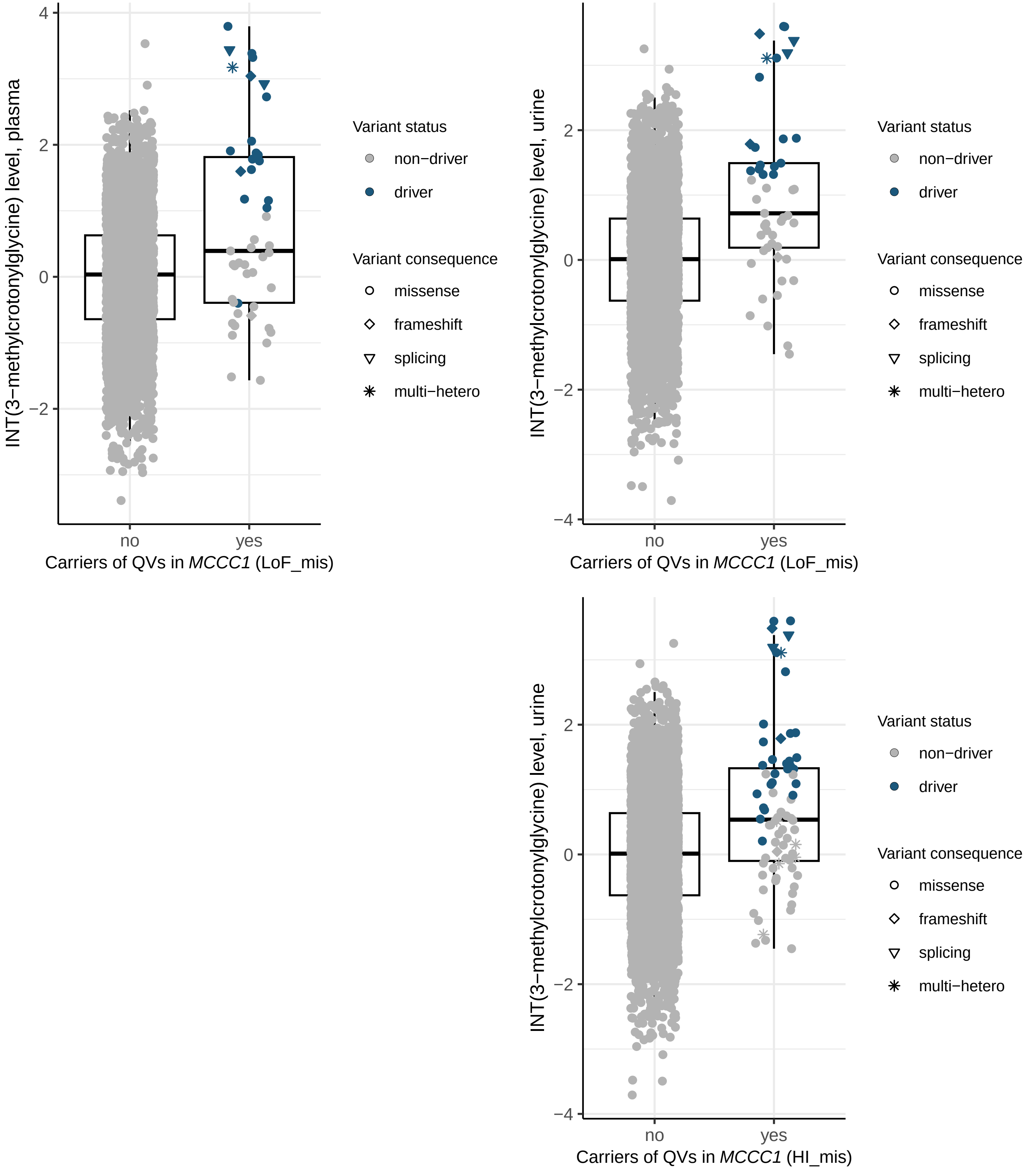

INT(3−methylcrotonylglycine) level, plasma
4
2
0
−2
no
yes
Carriers of QVs in MCCC1 (LoF_mis)
Variant status
non−driver
driver
Variant consequence
missense
frameshift
splicing
multi−hetero
INT(3−methylcrotonylglycine) level, urine
2
0
−2
−4
no
yes
Carriers of QVs in MCCC1 (LoF_mis)
Variant status
non−driver
driver
Variant consequence
missense
frameshift
splicing
multi−hetero
INT(3−methylcrotonylglycine) level, urine
2
0
−2
−4
no
yes
Carriers of QVs in MCCC1 (HI_mis)
Variant status
non−driver
driver
Variant consequence
missense
frameshift
splicing
multi−hetero

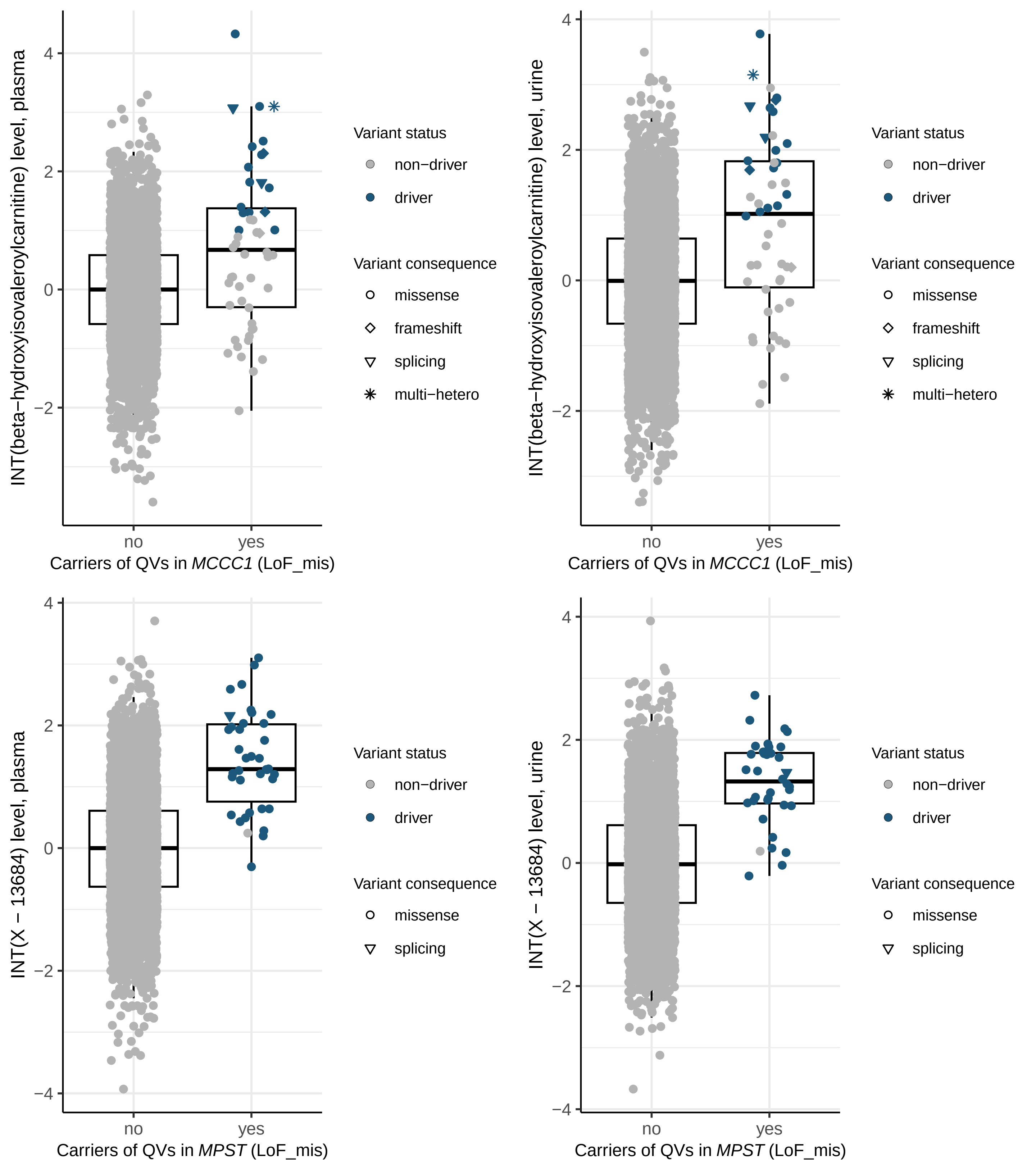

INT(beta−hydroxyisovaleroylcarnitine) level, plasma
4
2
0
−2
no
yes
Carriers of QVs in *MCCC1* (LoF_mis)
Variant status
non−driver
driver
Variant consequence
missense
frameshift
splicing
multi−hetero
INT(beta−hydroxyisovaleroylcarnitine) level, urine
4
2
0
−2
no
yes
Carriers of QVs in *MCCC1* (LoF_mis)
Variant status
non−driver
driver
Variant consequence
missense
frameshift
splicing
multi−hetero
INT(X − 13684) level, plasma
4
2
0
−2
−4
no
yes
Carriers of QVs in *MPST* (LoF_mis)
Variant status
non−driver
driver
Variant consequence
missense
splicing
INT(X − 13684) level, urine
4
2
0
−2
−4
no
yes
Carriers of QVs in *MPST* (LoF_mis)
Variant status
non−driver
driver
Variant consequence
missense
splicing

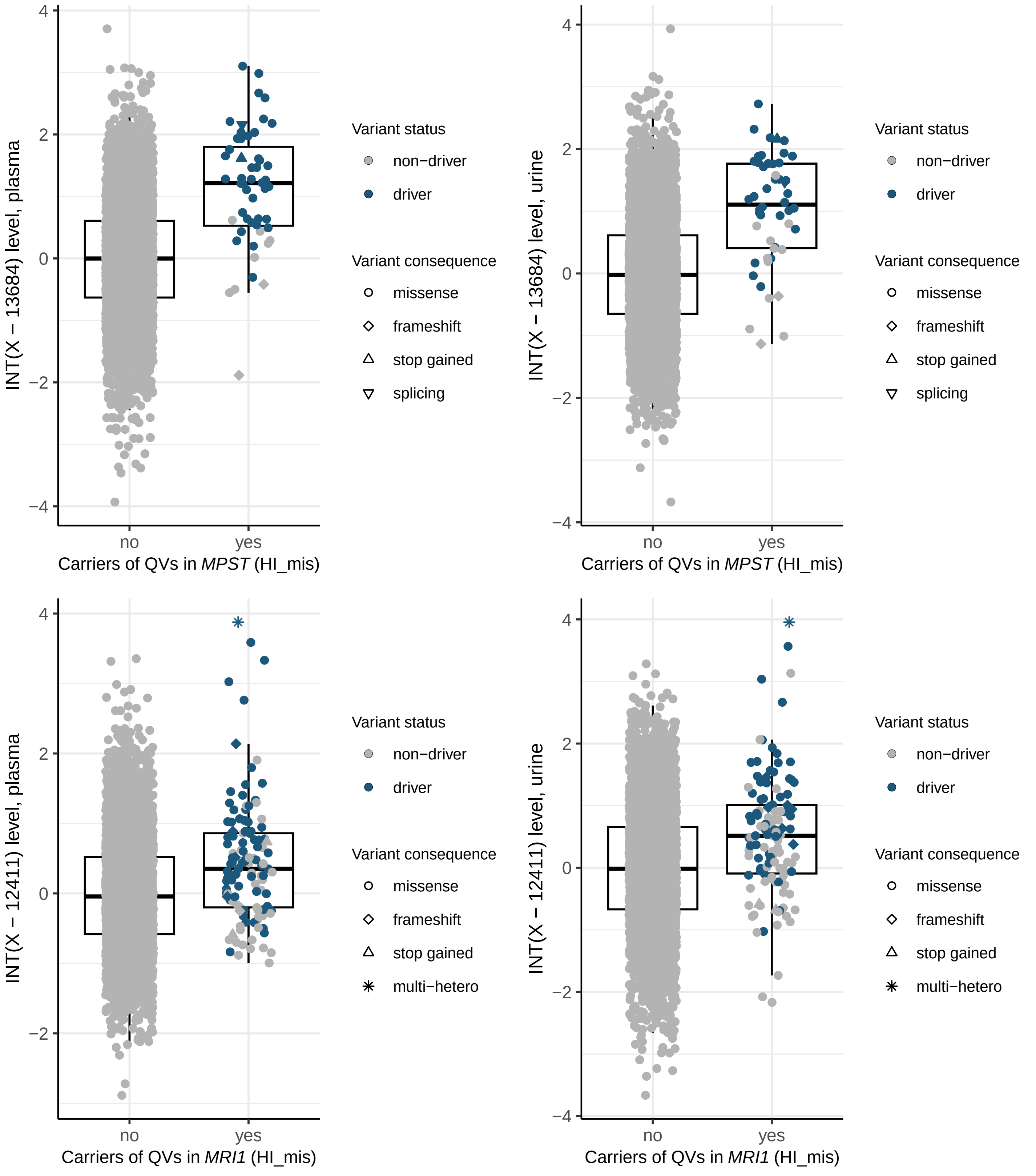

INT(X − 13684) level, plasma
Carriers of QVs in *MPST* (HI_mis)
INT(X − 13684) level, urine
Carriers of QVs in *MPST* (HI_mis)
INT(X − 12411) level, plasma
Carriers of QVs in *MRI1* (HI_mis)
INT(X − 12411) level, urine
Carriers of QVs in *MRI1* (HI_mis)
no
yes
Variant status
non−driver
driver
Variant consequence
missense
frameshift
stop gained
splicing
multi−hetero

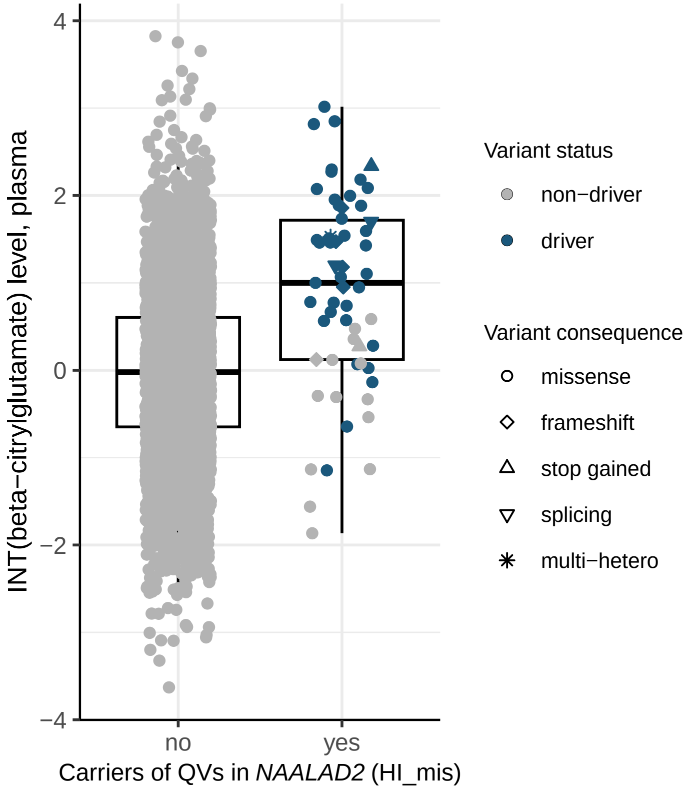

INT(beta−citrylglutamate) level, plasma
4
2
0
−2
−4
no
yes
Carriers of QVs in *NAALAD2* (HI_mis)
Variant status
non−driver
driver
Variant consequence
missense
frameshift
stop gained
splicing
multi−hetero

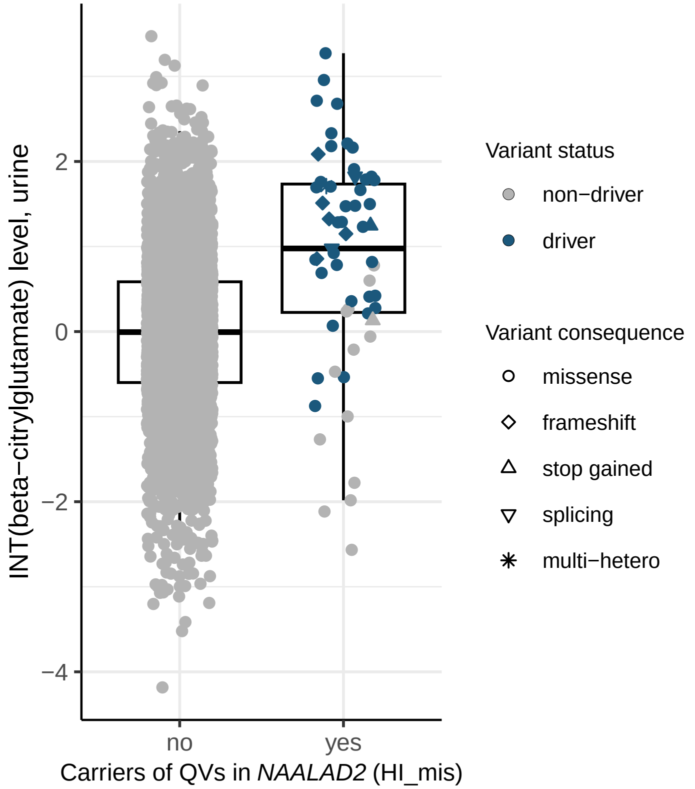

INT(beta−citrylglutamate) level, urine
2
0
−2
−4
no
yes
Carriers of QVs in *NAALAD2* (HI_mis)
Variant status
non−driver
driver
Variant consequence
missense
frameshift
stop gained
splicing
multi−hetero

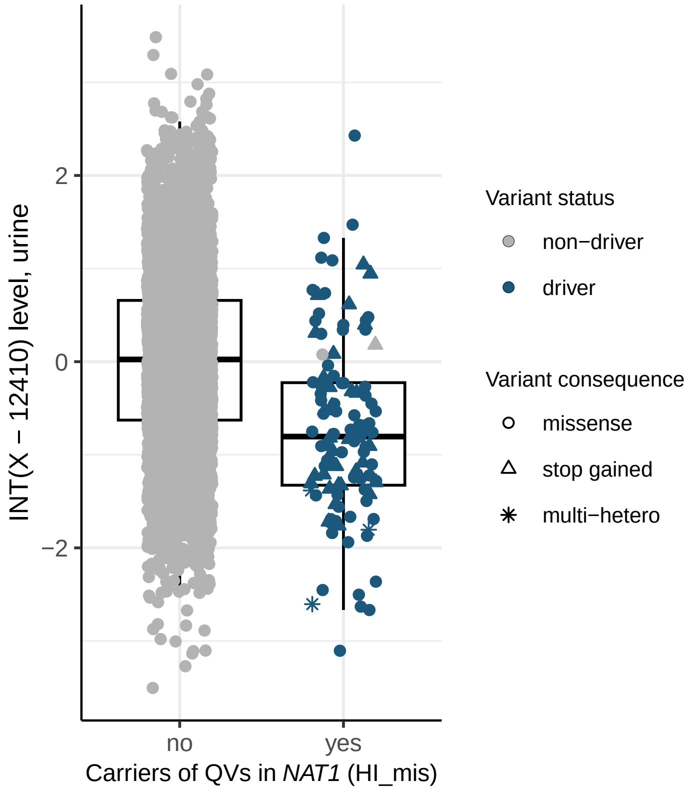

INT(X − 12410) level, urine
2
0
−2
no
yes
Carriers of QVs in *NAT1* (HI_mis)
Variant status
non−driver
driver
Variant consequence
missense
stop gained
multi−hetero

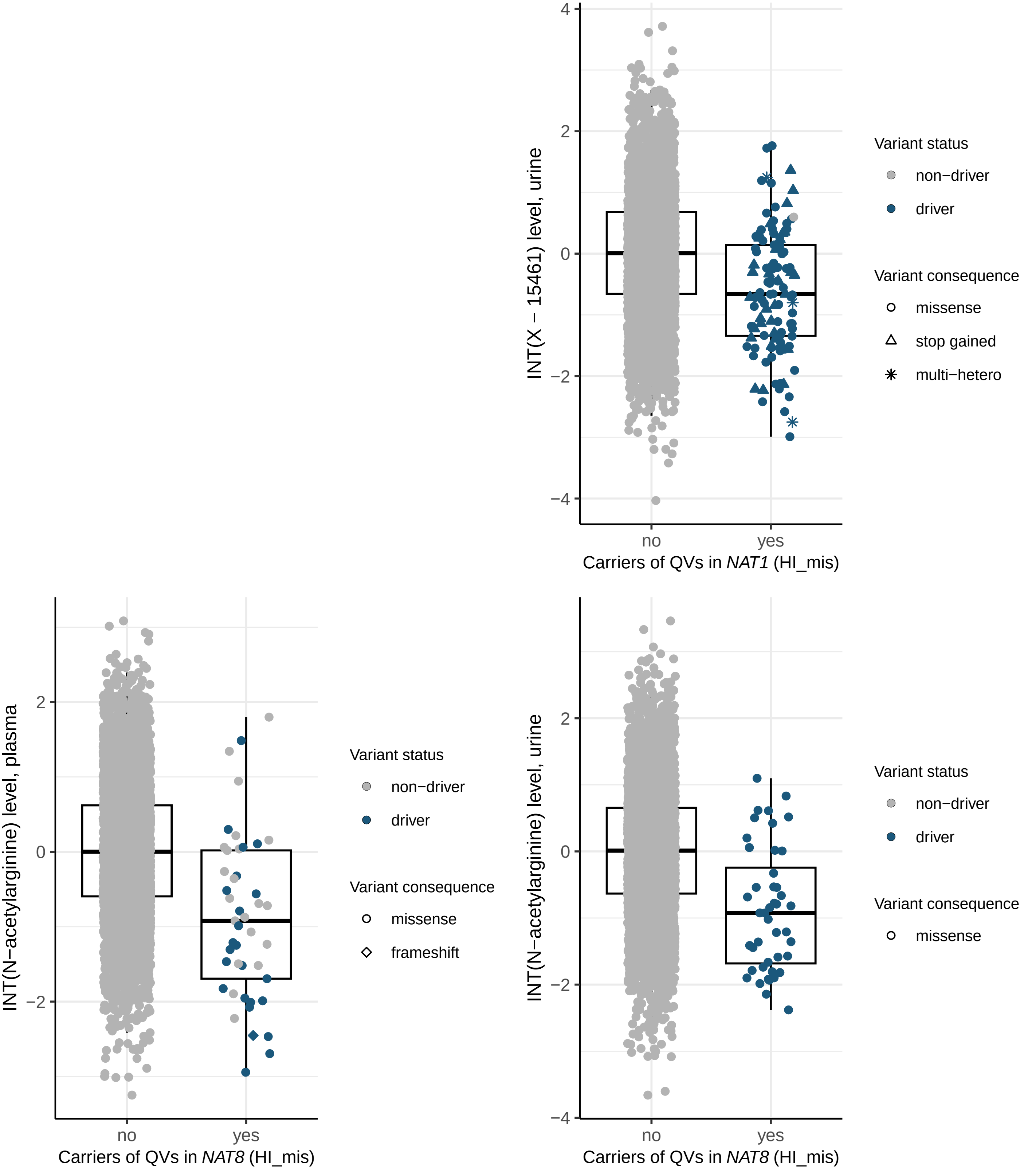

INT(X − 15461) level, urine
Carriers of QVs in *NAT1* (HI_mis)
no
yes
4
2
0
−2
−4
Variant status
non−driver
driver
Variant consequence
missense
stop gained
multi−hetero
INT(N−acetylarginine) level, plasma
Carriers of QVs in *NAT8* (HI_mis)
2
0
−2
Variant status
non−driver
driver
Variant consequence
missense
frameshift
INT(N−acetylarginine) level, urine
Carriers of QVs in *NAT8* (HI_mis)
2
0
−2
−4
Variant status
non−driver
driver
Variant consequence
missense

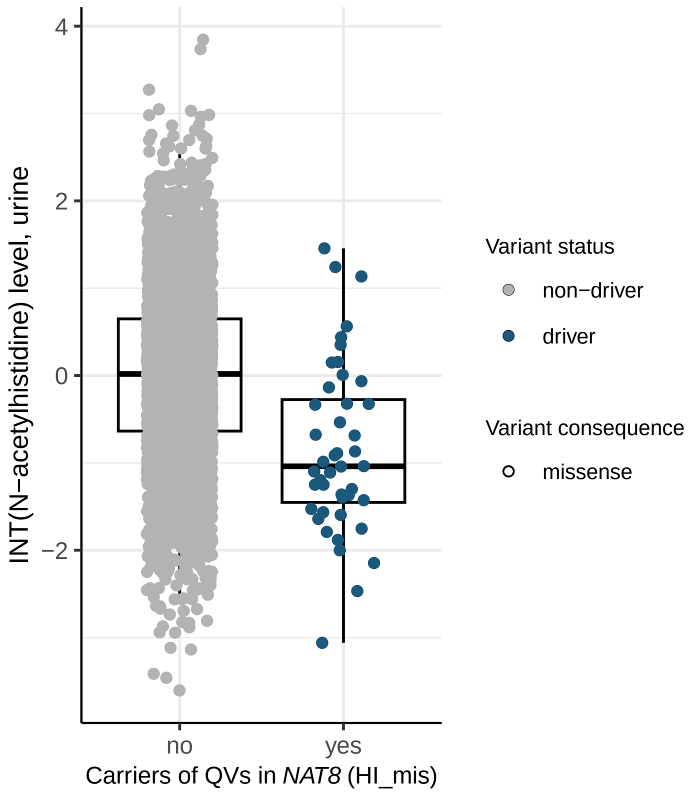
4
2
0
−2
INT(N−acetylhistidine) level, urine
no
yes
Carriers of QVs in *NAT8* (HI_mis)
Variant status
non−driver
driver
Variant consequence
missense

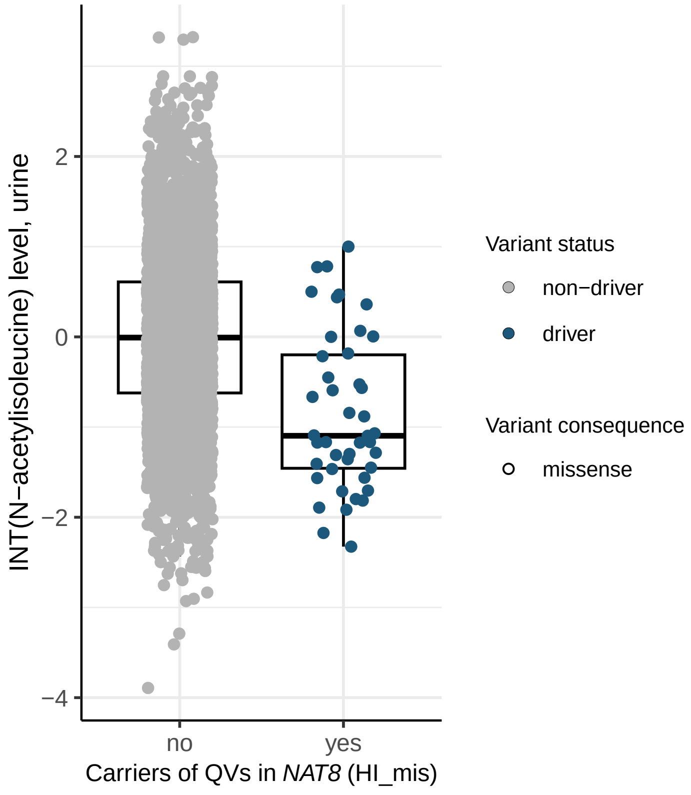
2
0
−2
−4
INT(N−acetylisoleucine) level, urine
no
yes
Carriers of QVs in *NAT8* (HI_mis)
Variant status
non−driver
driver
Variant consequence
missense

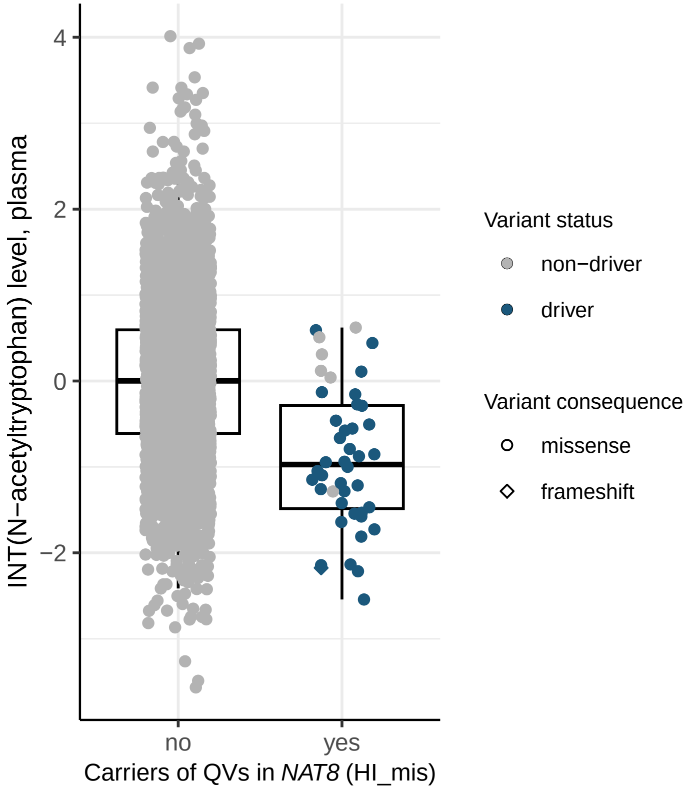
INT(N−acetyltryptophan) level, plasma
4
2
0
−2
no
yes
Carriers of QVs in *NAT8* (HI_mis)
Variant status
non−driver
driver
Variant consequence
missense
frameshift

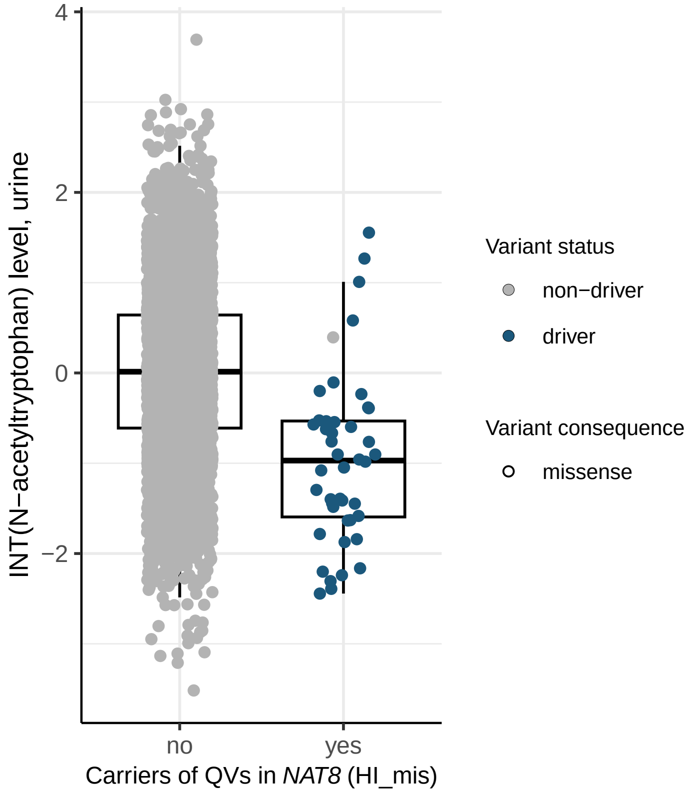
INT(N−acetyltryptophan) level, urine
4
2
0
−2
no
yes
Carriers of QVs in *NAT8* (HI_mis)
Variant status
non−driver
driver
Variant consequence
missense

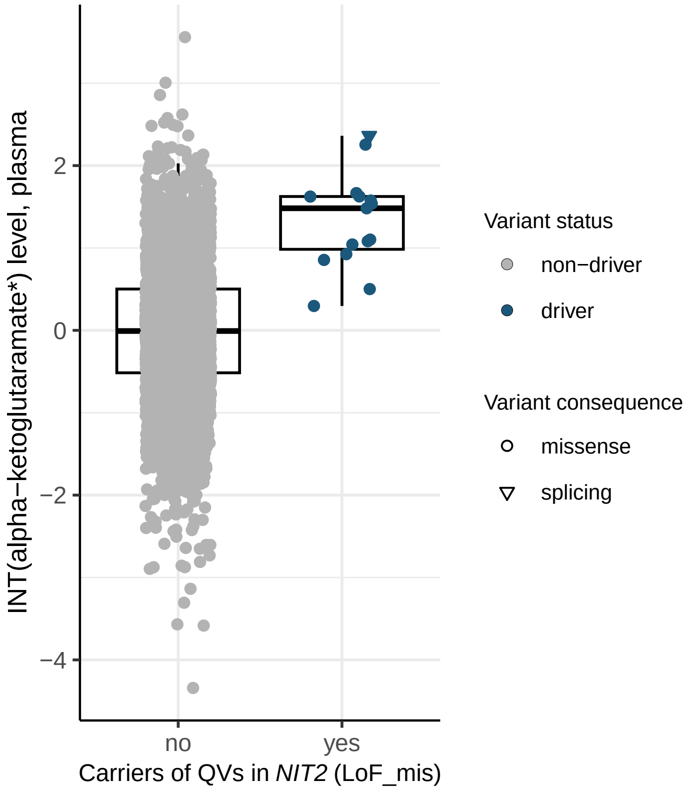
INT(alpha−ketoglutaramate*) level, plasma
2
0
−2
−4
no
yes
Carriers of QVs in *NIT2* (LoF_mis)
Variant status
non−driver
driver
Variant consequence
missense
splicing

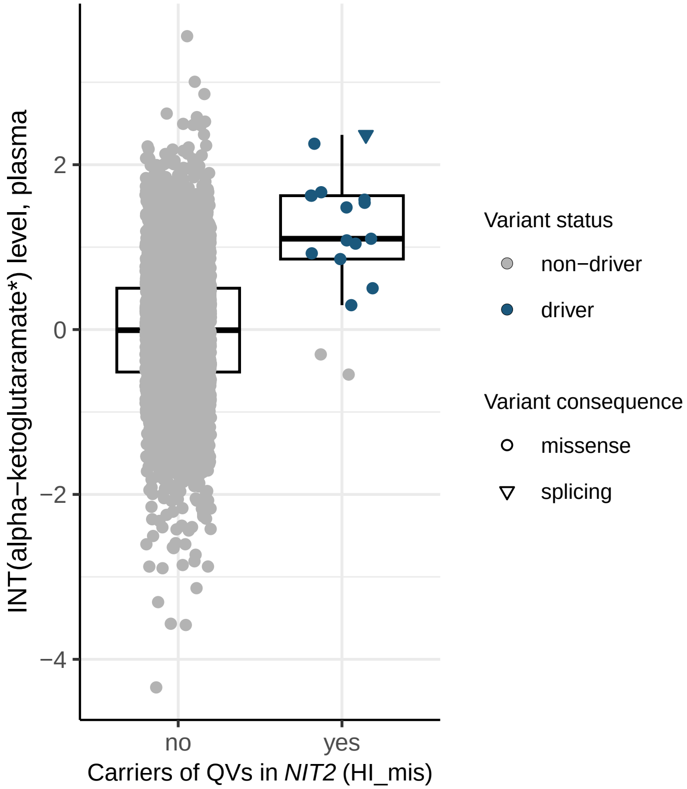

INT(alpha−ketoglutaramate*) level, plasma
2
0
−2
−4
no
yes
Carriers of QVs in NIT2 (HI_mis)
Variant status
non−driver
driver
Variant consequence
missense
splicing

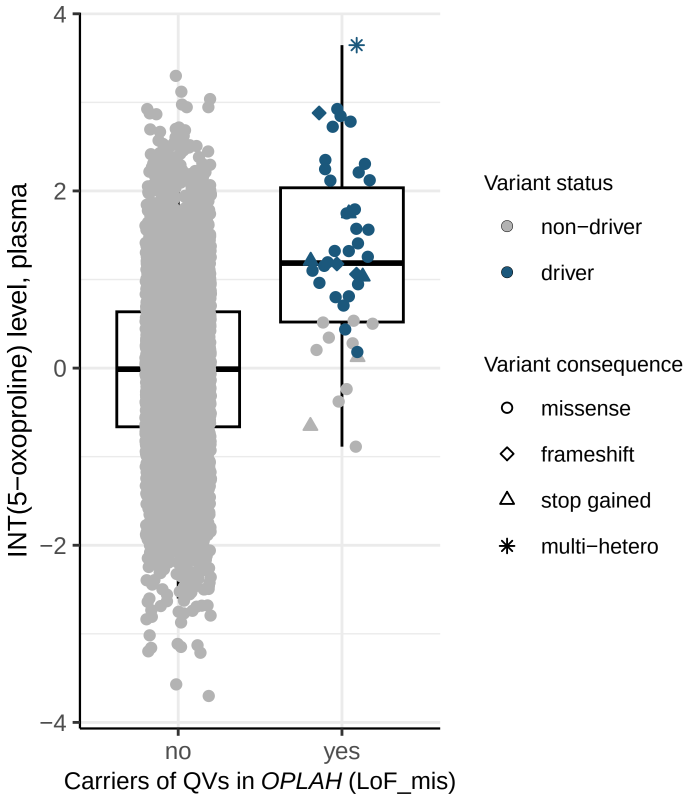

INT(5−oxoproline) level, plasma
4
2
0
−2
−4
no
yes
Carriers of QVs in OPLAH (LoF_mis)
Variant status
non−driver
driver
Variant consequence
missense
frameshift
stop gained
multi−hetero

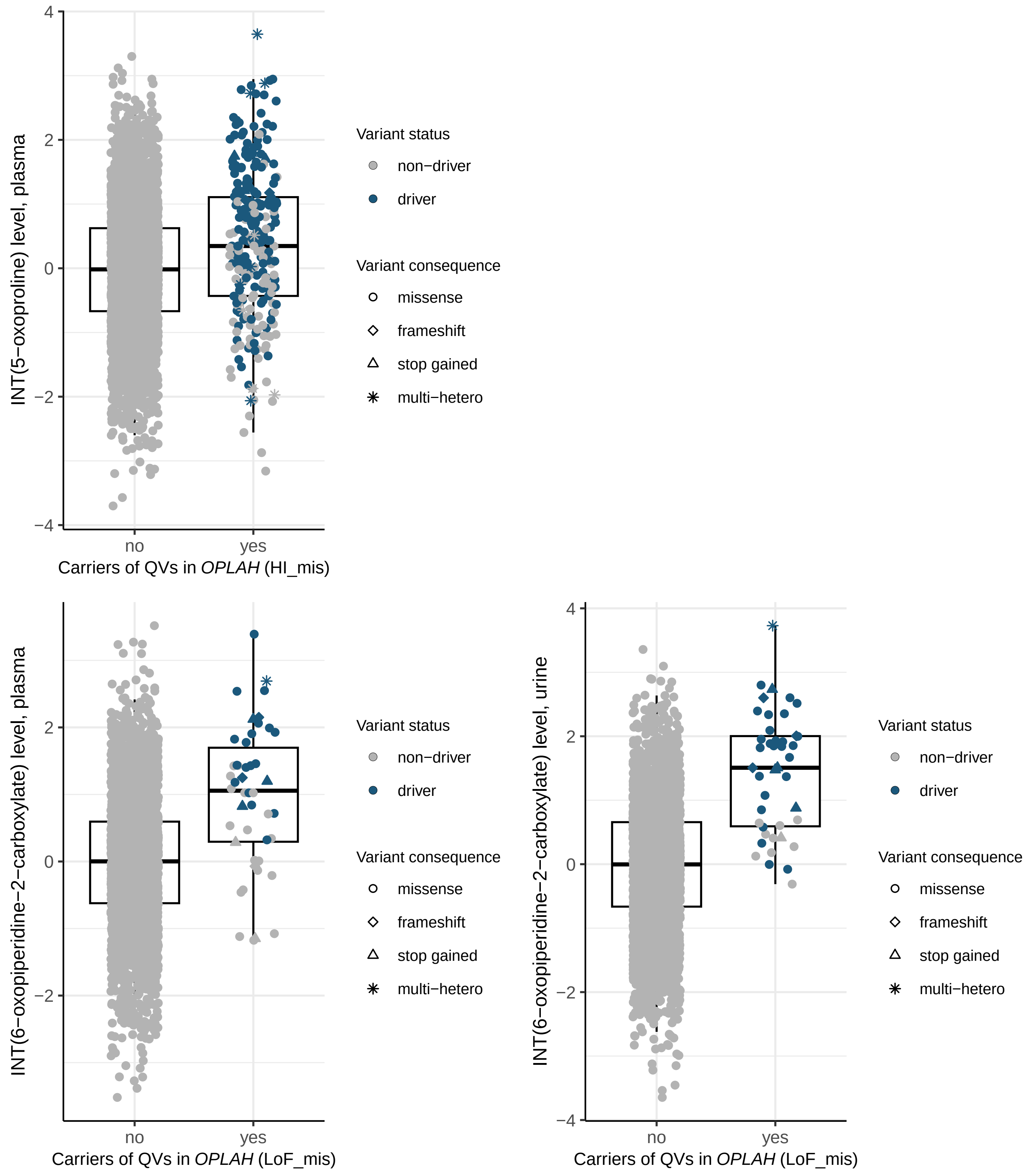

INT(5−oxoproline) level, plasma
Carriers of QVs in OPLAH (HI_mis)
INT(6−oxopiperidine−2−carboxylate) level, plasma
Carriers of QVs in OPLAH (LoF_mis)
INT(6−oxopiperidine−2−carboxylate) level, urine
Carriers of QVs in OPLAH (LoF_mis)
no
yes
4
2
0
−2
−4
Variant status
non−driver
driver
Variant consequence
missense
frameshift
stop gained
multi−hetero

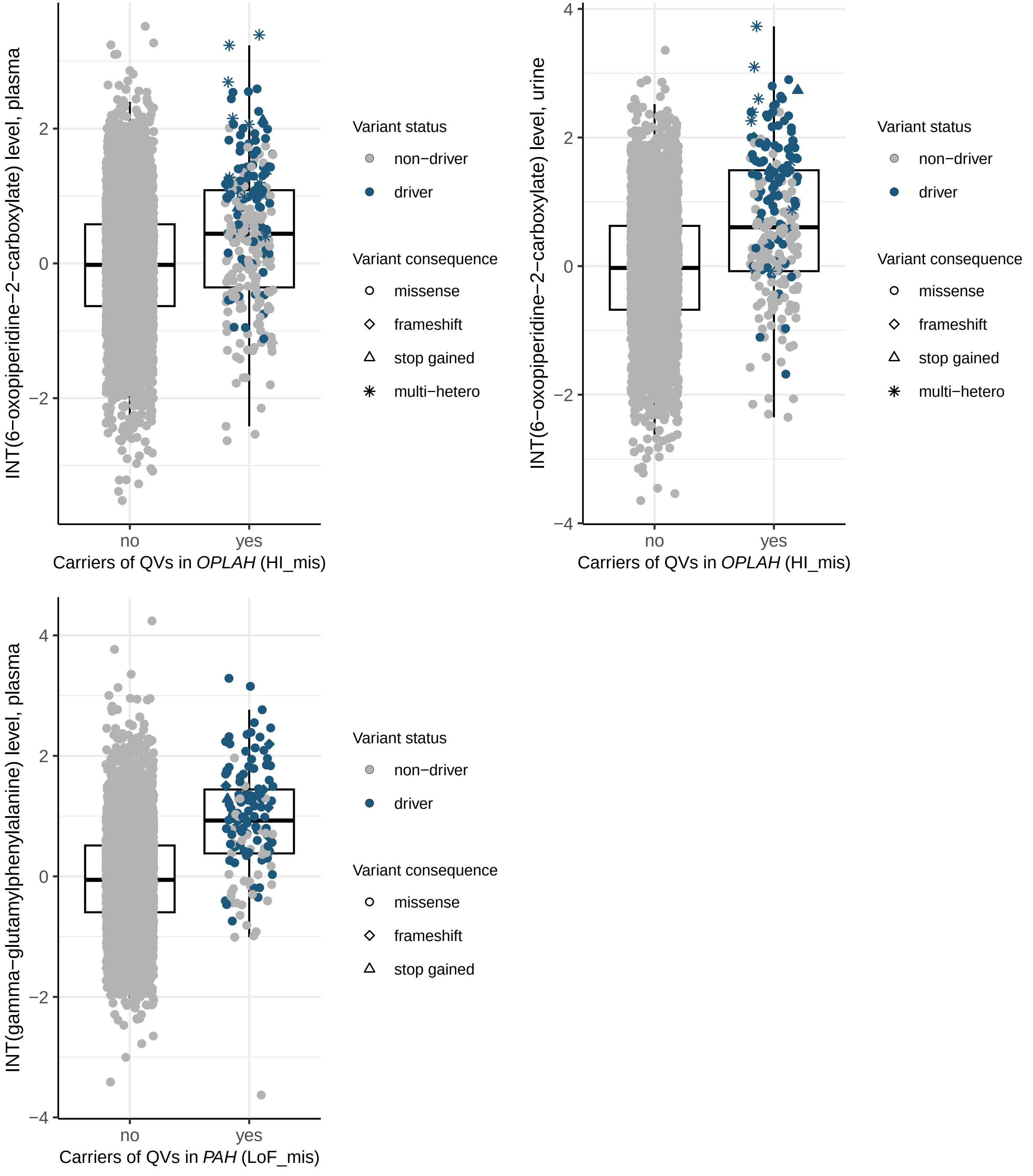

INT(6−oxopiperidine−2−carboxylate) level, plasma
2
0
−2
no
yes
Carriers of QVs in OPLAH (HI_mis)
Variant status
non−driver
driver
Variant consequence
missense
frameshift
stop gained
multi−hetero
INT(6−oxopiperidine−2−carboxylate) level, urine
4
2
0
−2
−4
no
yes
Carriers of QVs in OPLAH (HI_mis)
Variant status
non−driver
driver
Variant consequence
missense
frameshift
stop gained
multi−hetero
INT(gamma−glutamylphenylalanine) level, plasma
4
2
0
−2
−4
no
yes
Carriers of QVs in PAH (LoF_mis)
Variant status
non−driver
driver
Variant consequence
missense
frameshift
stop gained

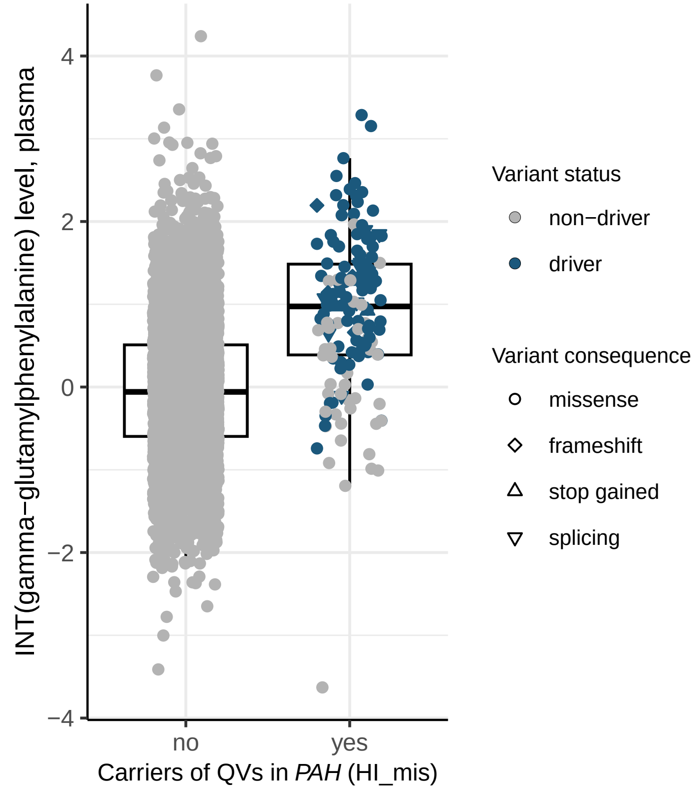

INT(gamma−glutamylphenylalanine) level, plasma
4
2
0
−2
−4
no
yes
Carriers of QVs in *PAH* (HI_mis)
Variant status
non−driver
driver
Variant consequence
missense
frameshift
stop gained
splicing

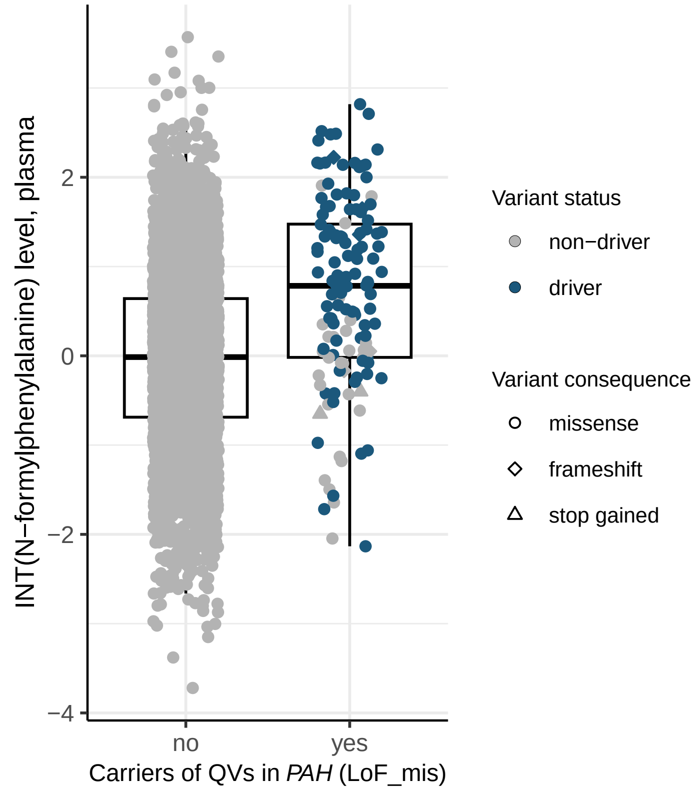

INT(N−formylphenylalanine) level, plasma
2
0
−2
−4
no
yes
Carriers of QVs in *PAH* (LoF_mis)
Variant status
non−driver
driver
Variant consequence
missense
frameshift
stop gained

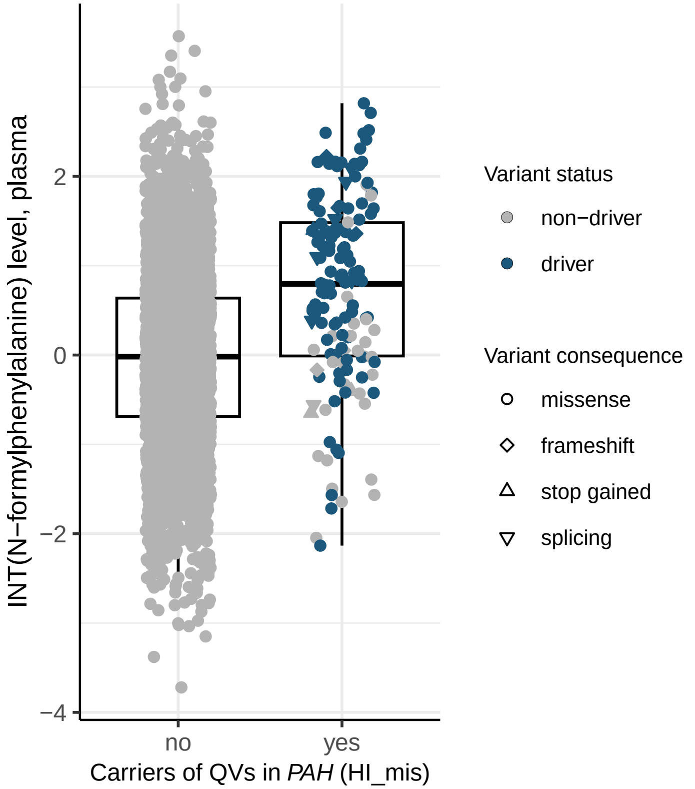
INT(N−formylphenylalanine) level, plasma
2
0
−2
−4
no
yes
Carriers of QVs in *PAH* (HI_mis)
Variant status
non−driver
driver
Variant consequence
missense
frameshift
stop gained
splicing

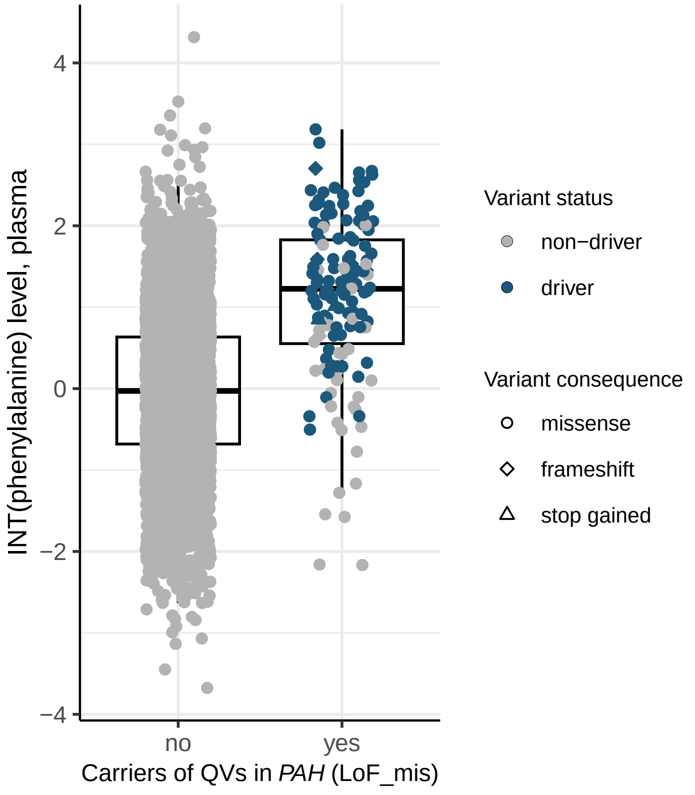
INT(phenylalanine) level, plasma
4
2
0
−2
−4
no
yes
Carriers of QVs in *PAH* (LoF_mis)
Variant status
non−driver
driver
Variant consequence
missense
frameshift
stop gained

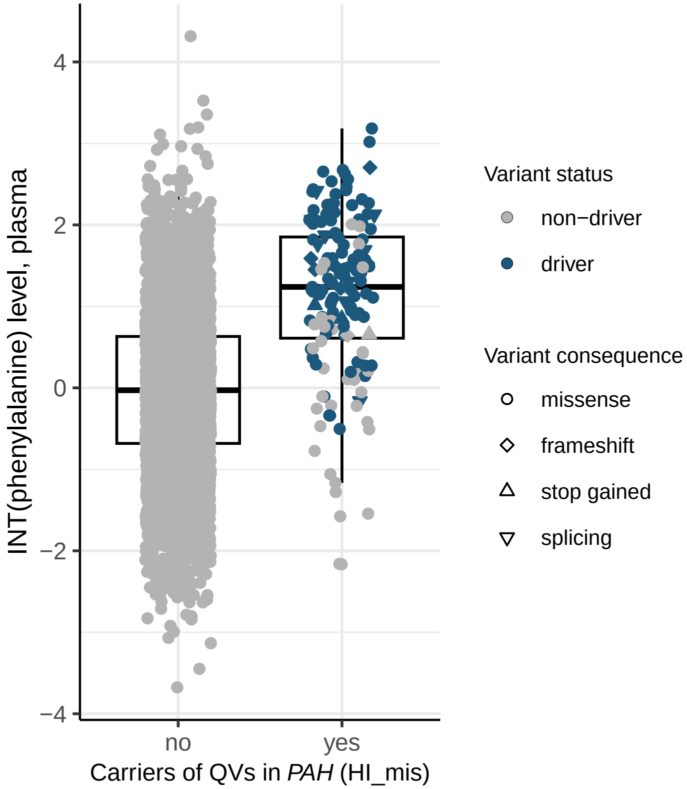

INT(phenylalanine) level, plasma
4
2
0
−2
−4
no
yes
Carriers of QVs in *PAH* (HI_mis)
Variant status
non−driver
driver
Variant consequence
missense
frameshift
stop gained
splicing

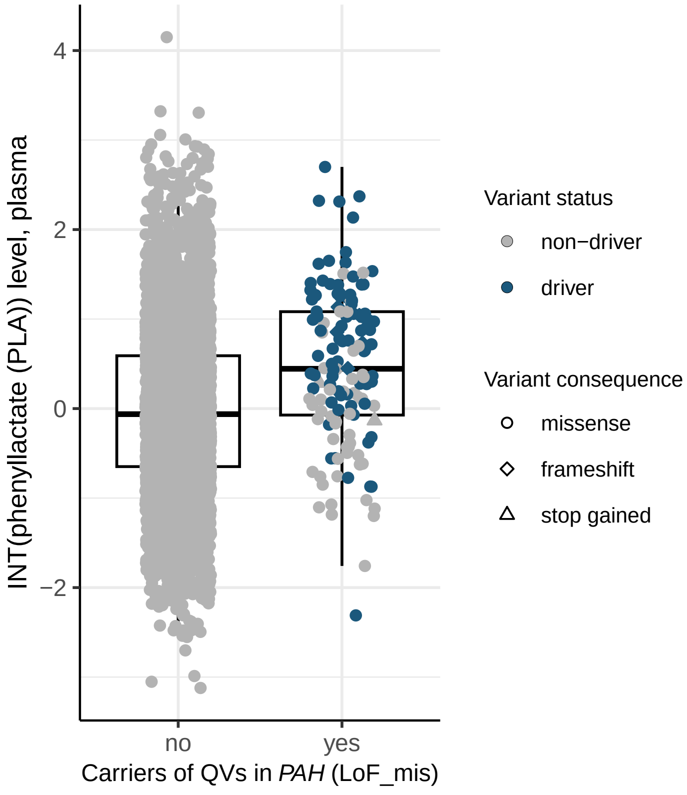

INT(phenyllactate (PLA)) level, plasma
4
2
0
−2
no
yes
Carriers of QVs in *PAH* (LoF_mis)
Variant status
non−driver
driver
Variant consequence
missense
frameshift
stop gained

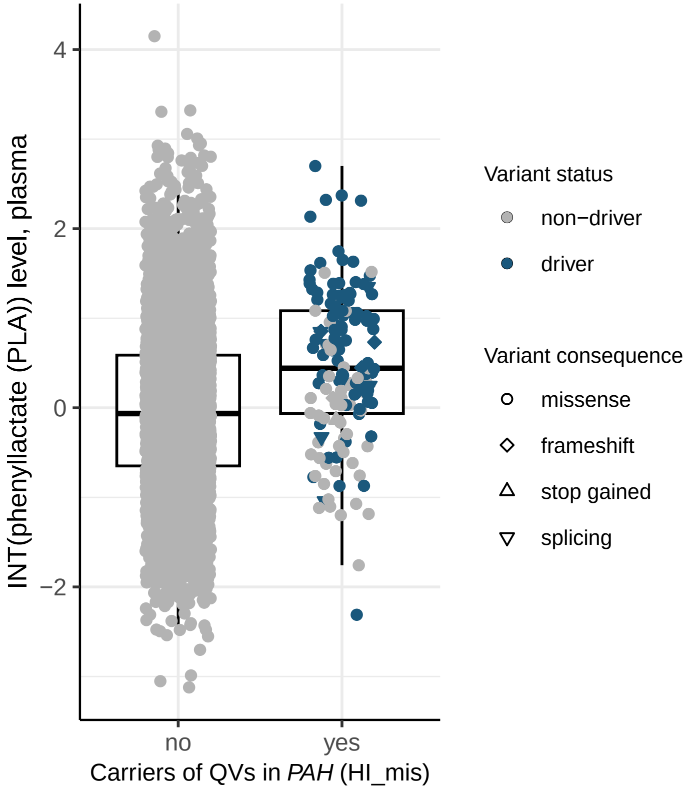
INT(phenyllactate (PLA)) level, plasma
4
2
0
−2
no
yes
Carriers of QVs in *PAH* (HI_mis)
Variant status
non−driver
driver
Variant consequence
missense
frameshift
stop gained
splicing

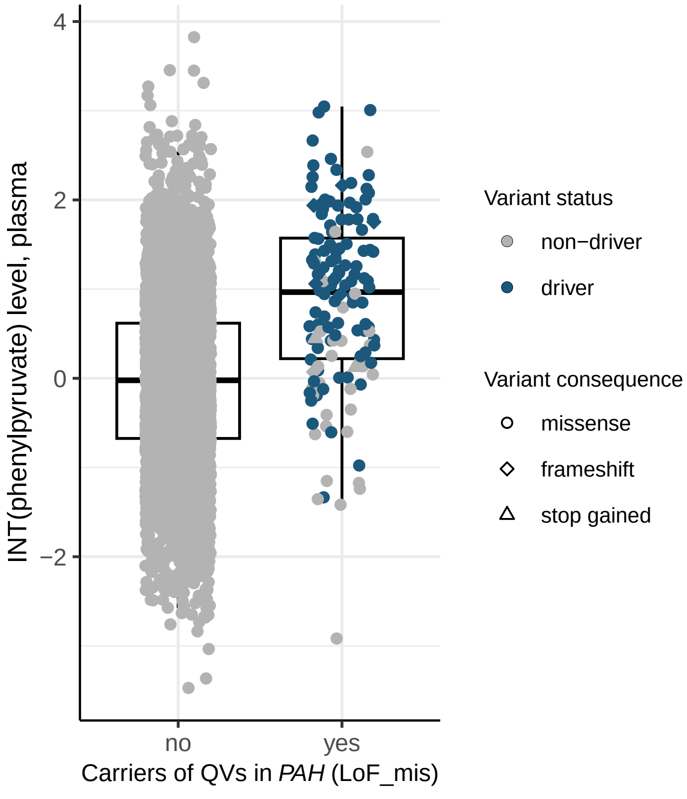
INT(phenylpyruvate) level, plasma
4
2
0
−2
no
yes
Carriers of QVs in *PAH* (LoF_mis)
Variant status
non−driver
driver
Variant consequence
missense
frameshift
stop gained

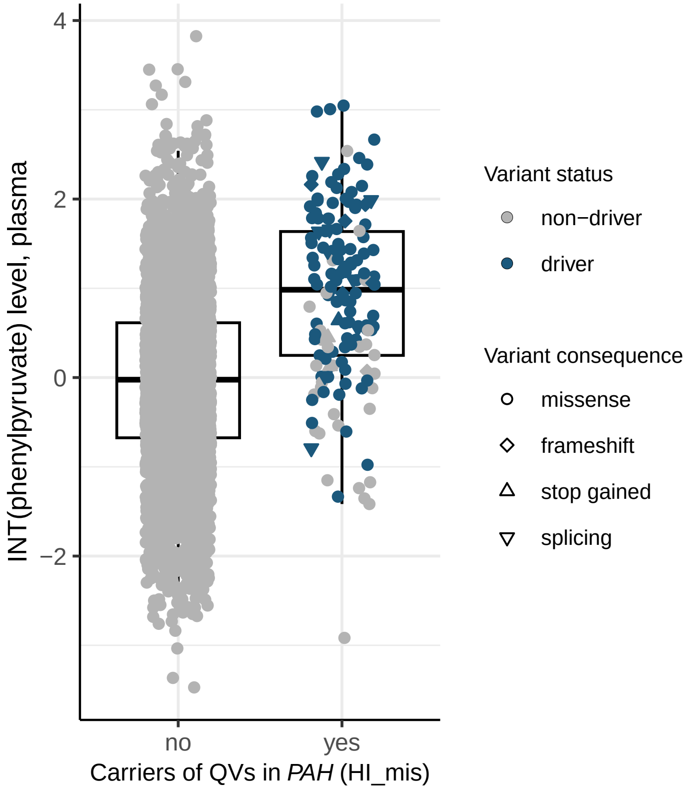

4
2
0
−2
INT(phenylpyruvate) level, plasma
no
yes
Carriers of QVs in PAH (HI_mis)
Variant status
non−driver
driver
Variant consequence
missense
frameshift
stop gained
splicing

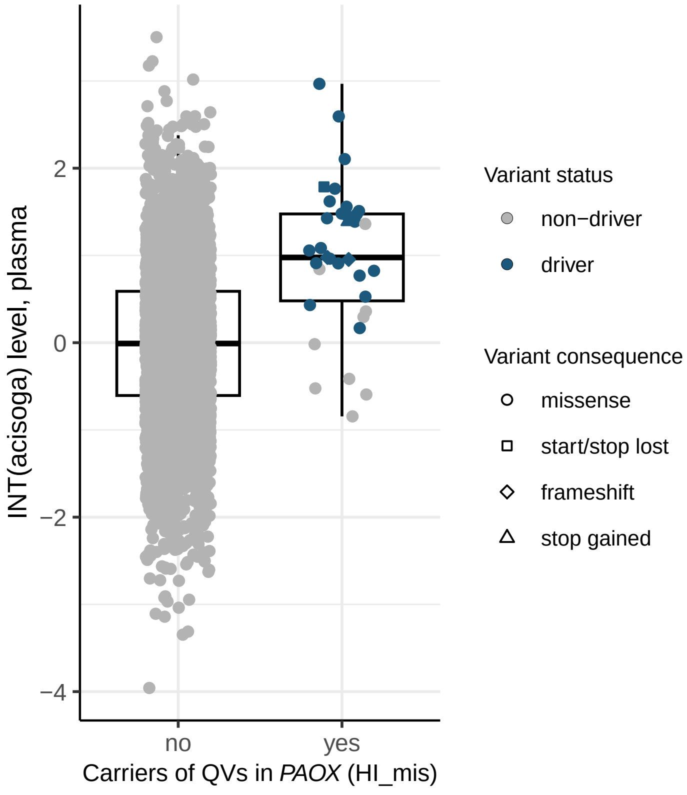

2
0
−2
−4
INT(acisoga) level, plasma
no
yes
Carriers of QVs in PAOX (HI_mis)
Variant status
non−driver
driver
Variant consequence
missense
start/stop lost
frameshift
stop gained

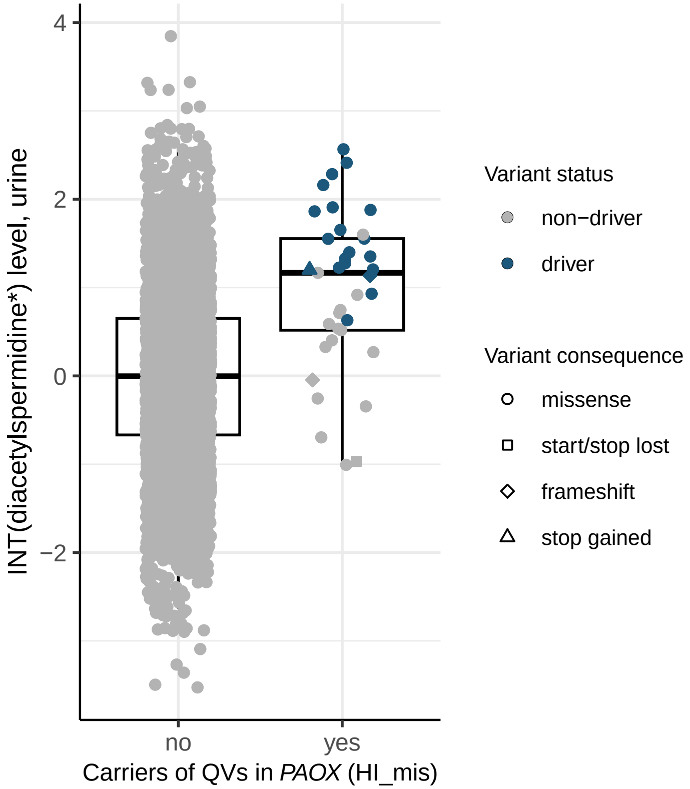
INT(diacetylspermidine*) level, urine
Carriers of QVs in *PAOX* (HI_mis)
4
2
0
−2
no
yes
Variant status
non−driver
driver
Variant consequence
missense
start/stop lost
frameshift
stop gained

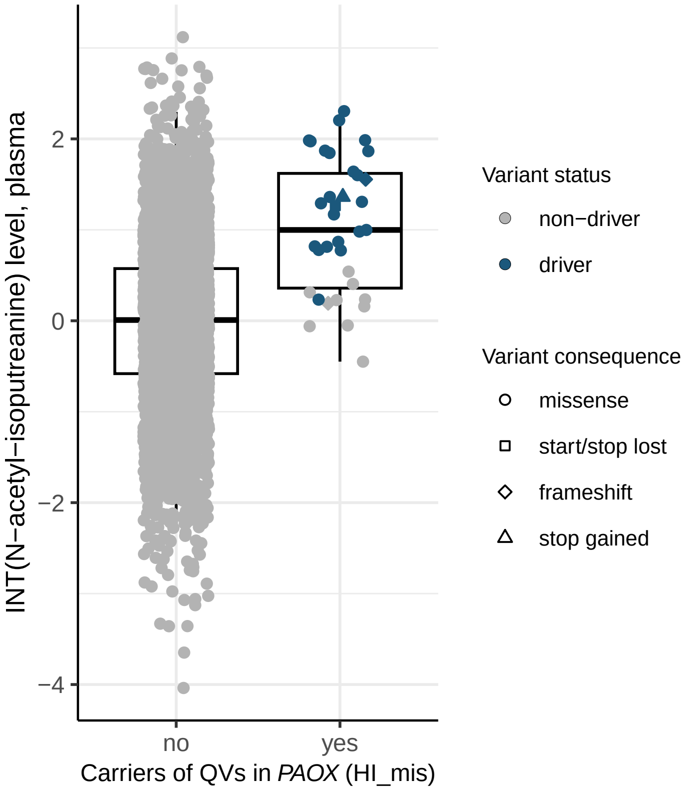
INT(N−acetyl−isoputreanine) level, plasma
Carriers of QVs in *PAOX* (HI_mis)
2
0
−2
−4
no
yes
Variant status
non−driver
driver
Variant consequence
missense
start/stop lost
frameshift
stop gained

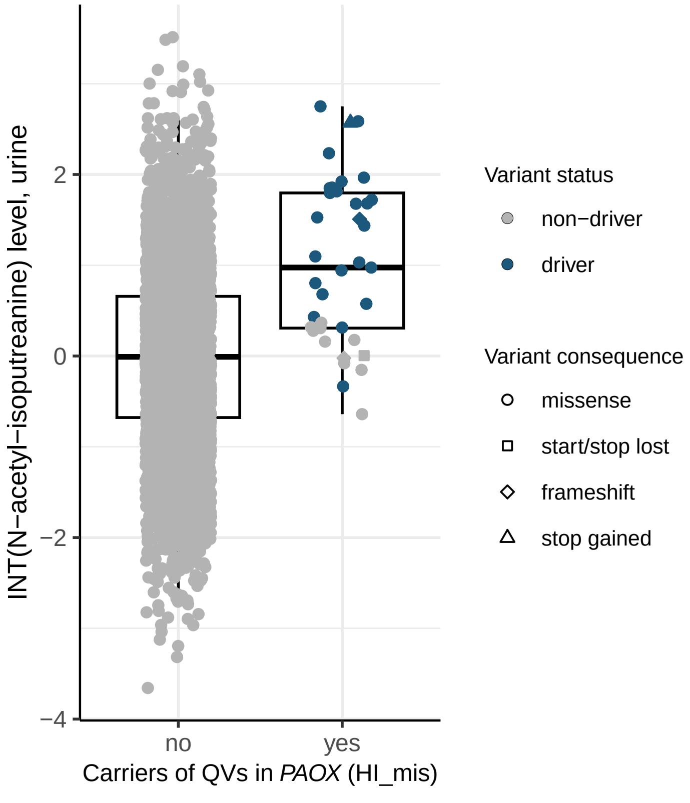
INT(N−acetyl−isoputreanine) level, urine
Carriers of QVs in *PAOX* (HI_mis)
2
0
−2
−4
no
yes
Variant status
non−driver
driver
Variant consequence
missense
start/stop lost
frameshift
stop gained

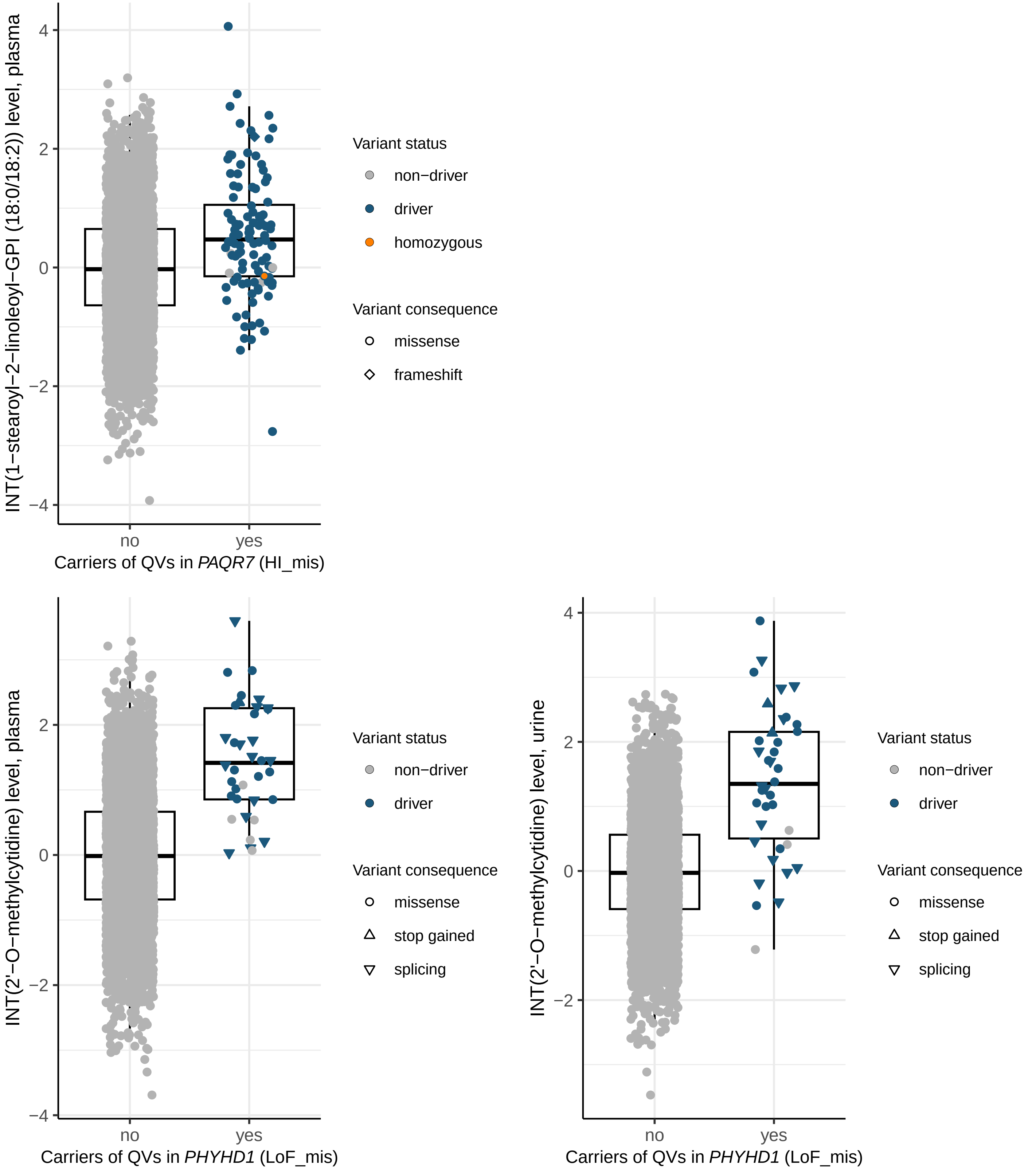
INT(1−stearoyl−2−linoleoyl−GPI (18:0/18:2)) level, plasma
4
2
0
−2
−4
no
yes
Carriers of QVs in PAQR7 (HI_mis)
Variant status
non−driver
driver
homozygous
Variant consequence
missense
frameshift
INT(2'−O−methylcytidine) level, plasma
2
0
−2
−4
no
yes
Carriers of QVs in PHYHD1 (LoF_mis)
Variant status
non−driver
driver
Variant consequence
missense
stop gained
splicing
INT(2'−O−methylcytidine) level, urine
4
2
0
−2
no
yes
Carriers of QVs in PHYHD1 (LoF_mis)
Variant status
non−driver
driver
Variant consequence
missense
stop gained
splicing

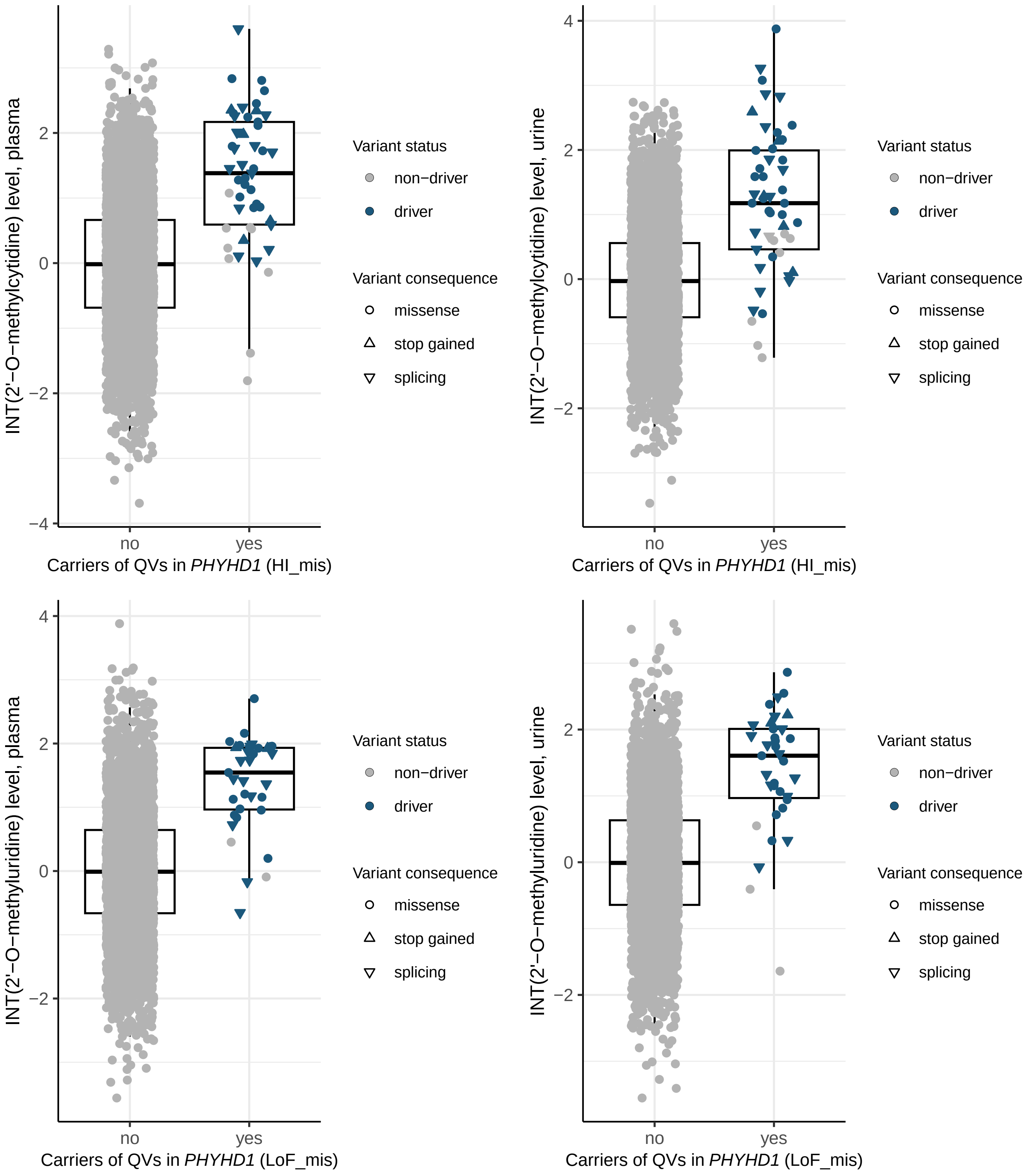

INT(2'−O−methylcytidine) level, plasma
Carriers of QVs in PHYHD1 (HI_mis)
INT(2'−O−methylcytidine) level, urine
Carriers of QVs in PHYHD1 (HI_mis)
INT(2'−O−methyluridine) level, plasma
Carriers of QVs in PHYHD1 (LoF_mis)
INT(2'−O−methyluridine) level, urine
Carriers of QVs in PHYHD1 (LoF_mis)
no
yes
Variant status
non−driver
driver
Variant consequence
missense
stop gained
splicing

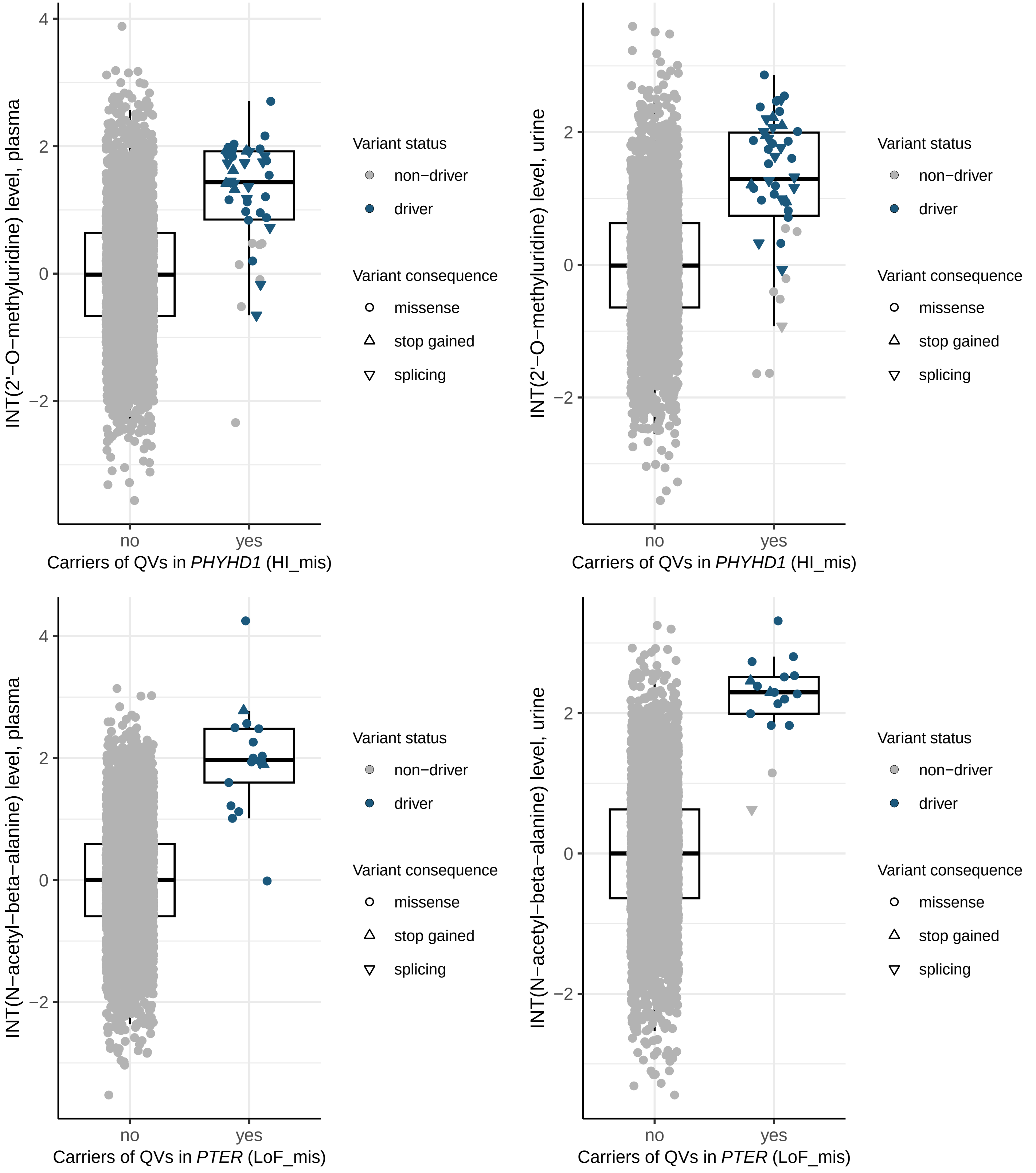
INT(2'−O−methyluridine) level, plasma
Carriers of QVs in PHYHD1 (HI_mis)
INT(2'−O−methyluridine) level, urine
Carriers of QVs in PHYHD1 (HI_mis)
INT(N−acetyl−beta−alanine) level, plasma
Carriers of QVs in PTER (LoF_mis)
INT(N−acetyl−beta−alanine) level, urine
Carriers of QVs in PTER (LoF_mis)
no
yes
Variant status
non−driver
driver
Variant consequence
missense
stop gained
splicing

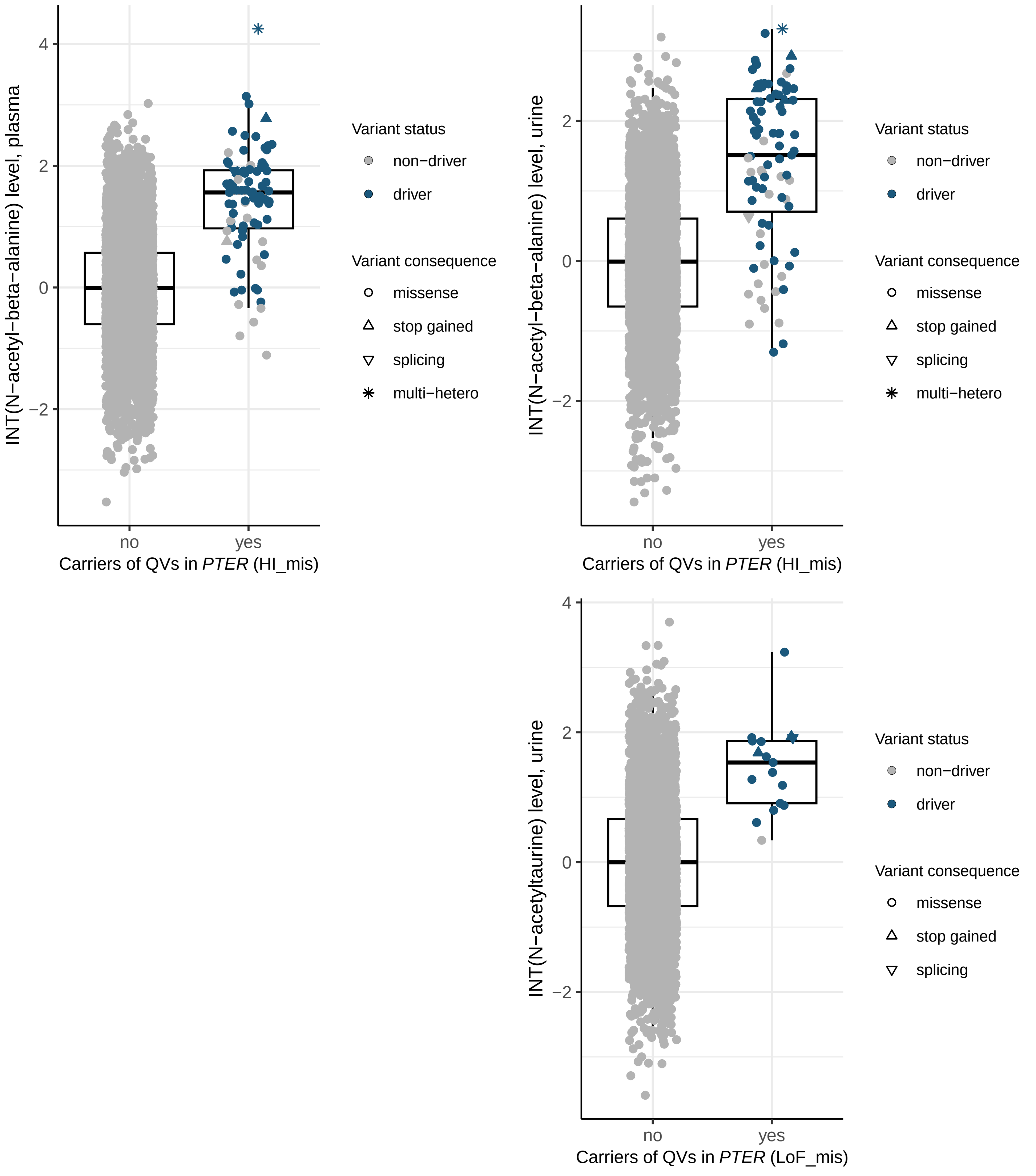

INT(N−acetyl−beta−alanine) level, plasma
Carriers of QVs in PTER (HI_mis)
no
yes
Variant status
non−driver
driver
Variant consequence
missense
stop gained
splicing
multi−hetero
INT(N−acetyl−beta−alanine) level, urine
Carriers of QVs in PTER (HI_mis)
INT(N−acetyltaurine) level, urine
Carriers of QVs in PTER (LoF_mis)

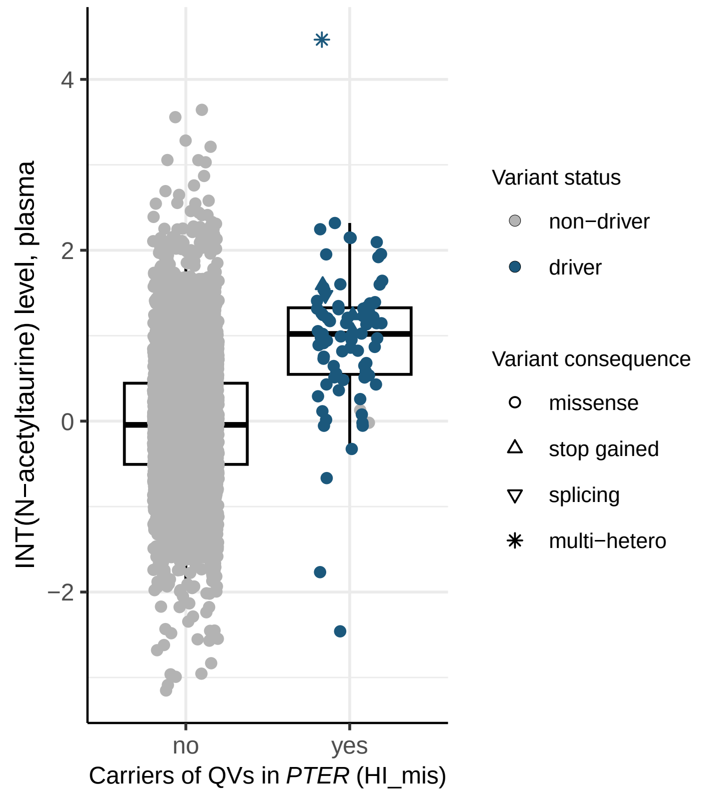
INT(N−acetyltaurine) level, plasma
4
2
0
−2
no
yes
Carriers of QVs in *PTER* (HI_mis)
Variant status
non−driver
driver
Variant consequence
missense
stop gained
splicing
multi−hetero

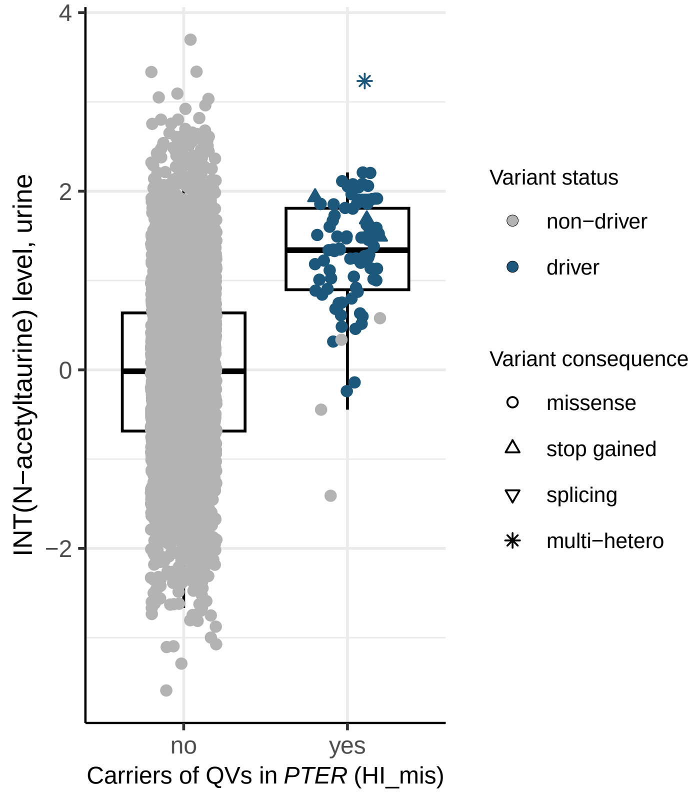
INT(N−acetyltaurine) level, urine
4
2
0
−2
no
yes
Carriers of QVs in *PTER* (HI_mis)
Variant status
non−driver
driver
Variant consequence
missense
stop gained
splicing
multi−hetero

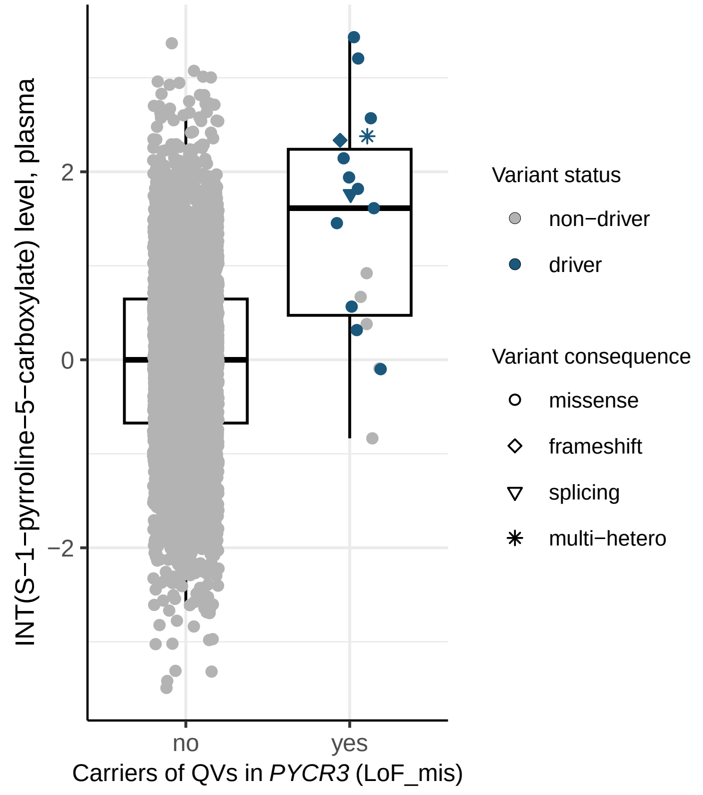
INT(S−1−pyrroline−5−carboxylate) level, plasma
2
0
−2
no
yes
Carriers of QVs in *PYCR3* (LoF_mis)
Variant status
non−driver
driver
Variant consequence
missense
frameshift
splicing
multi−hetero

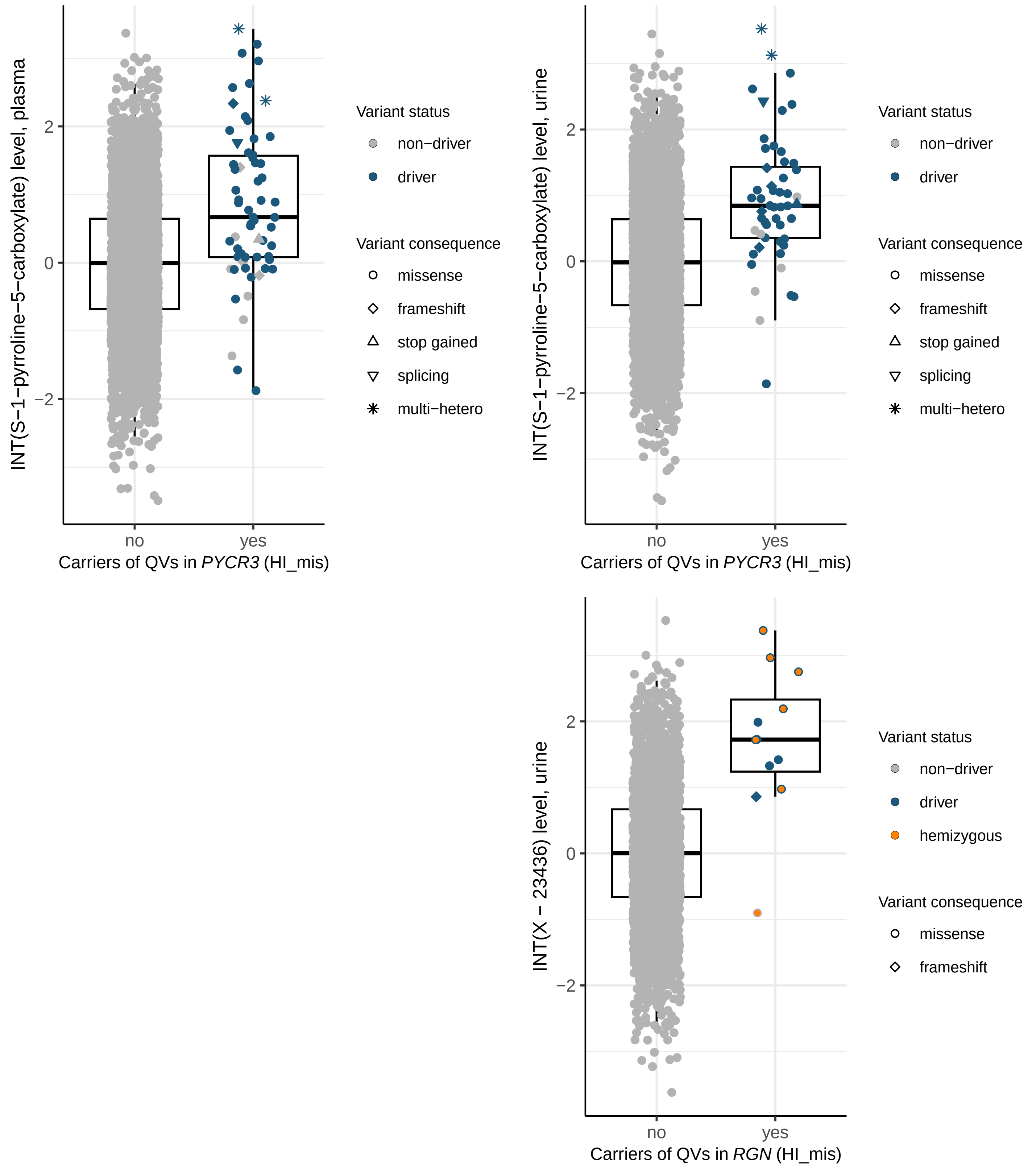
INT(S−1−pyrroline−5−carboxylate) level, plasma
2
0
−2
no
yes
Carriers of QVs in *PYCR3* (HI_mis)
Variant status
non−driver
driver
Variant consequence
missense
frameshift
stop gained
splicing
multi−hetero
INT(S−1−pyrroline−5−carboxylate) level, urine
2
0
−2
no
yes
Carriers of QVs in *PYCR3* (HI_mis)
Variant status
non−driver
driver
Variant consequence
missense
frameshift
stop gained
splicing
multi−hetero
INT(X − 23436) level, urine
2
0
−2
no
yes
Carriers of QVs in *RGN* (HI_mis)
Variant status
non−driver
driver
hemizygous
Variant consequence
missense
frameshift

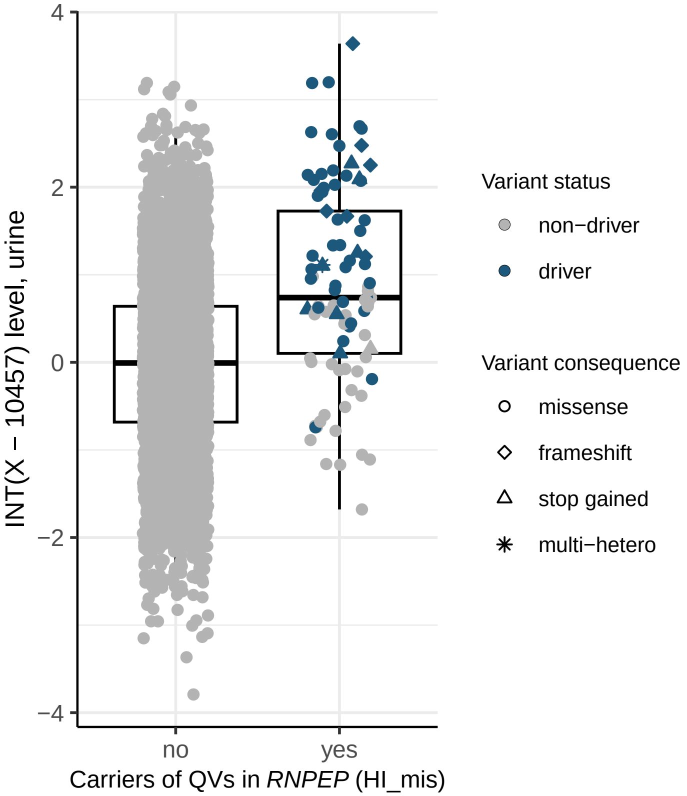
4
2
0
−2
−4
INT(X − 10457) level, urine
no
yes
Carriers of QVs in RNPEP (HI_mis)
Variant status
non−driver
driver
Variant consequence
missense
frameshift
stop gained
multi−hetero

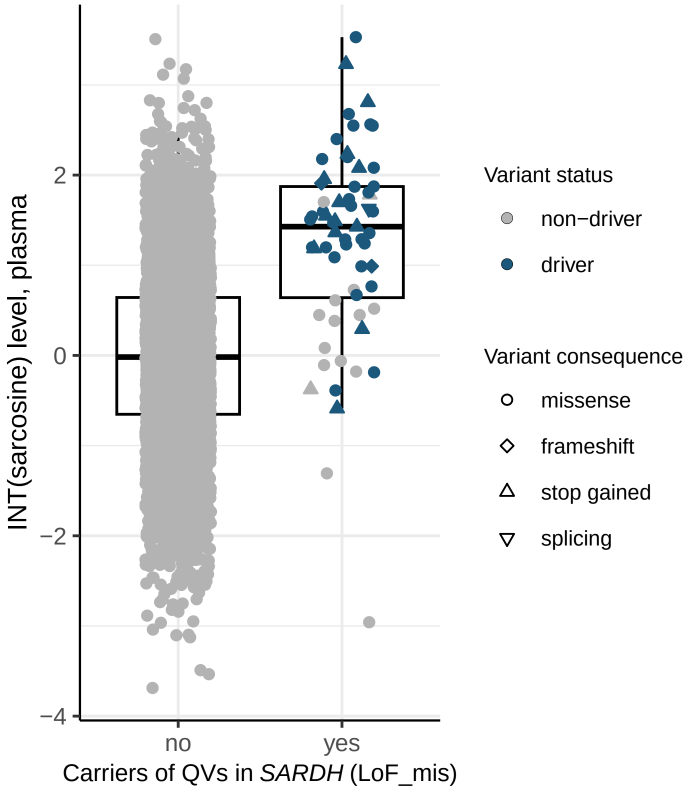
2
0
−2
−4
INT(sarcosine) level, plasma
no
yes
Carriers of QVs in SARDH (LoF_mis)
Variant status
non−driver
driver
Variant consequence
missense
frameshift
stop gained
splicing

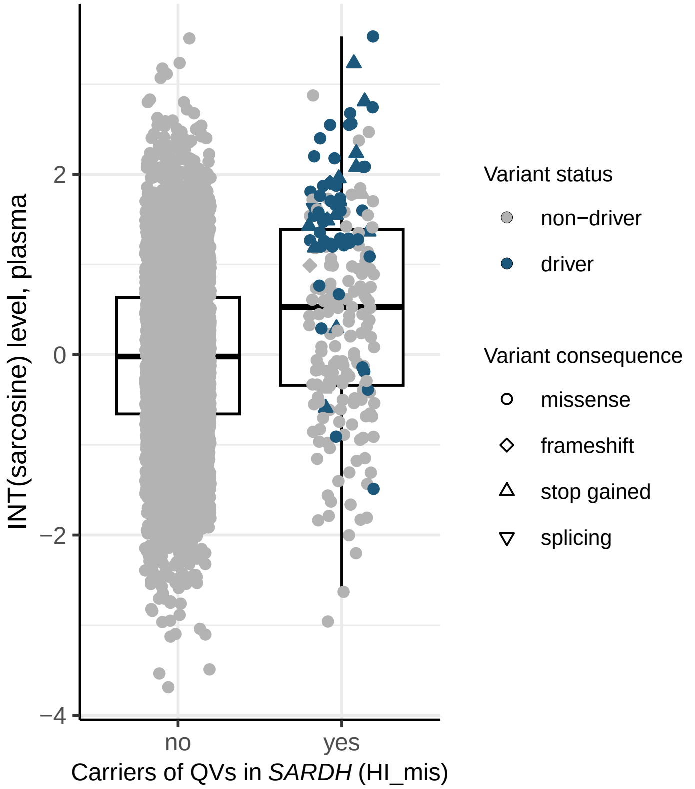
INT(sarcosine) level, plasma
2
0
−2
−4
no
yes
Carriers of QVs in *SARDH* (HI_mis)
Variant status
non−driver
driver
Variant consequence
missense
frameshift
stop gained
splicing

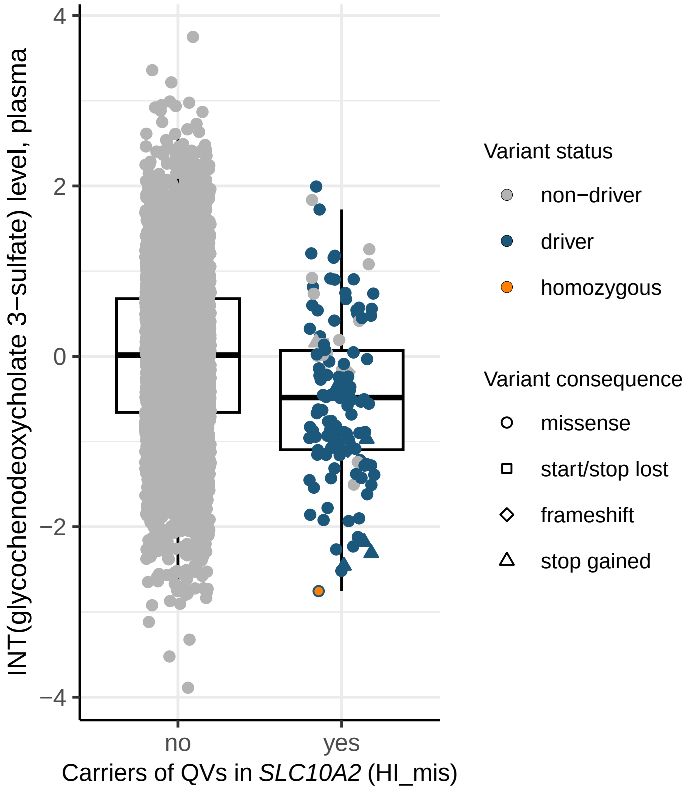
INT(glycochenodeoxycholate 3−sulfate) level, plasma
4
2
0
−2
−4
no
yes
Carriers of QVs in *SLC10A2* (HI_mis)
Variant status
non−driver
driver
homozygous
Variant consequence
missense
start/stop lost
frameshift
stop gained

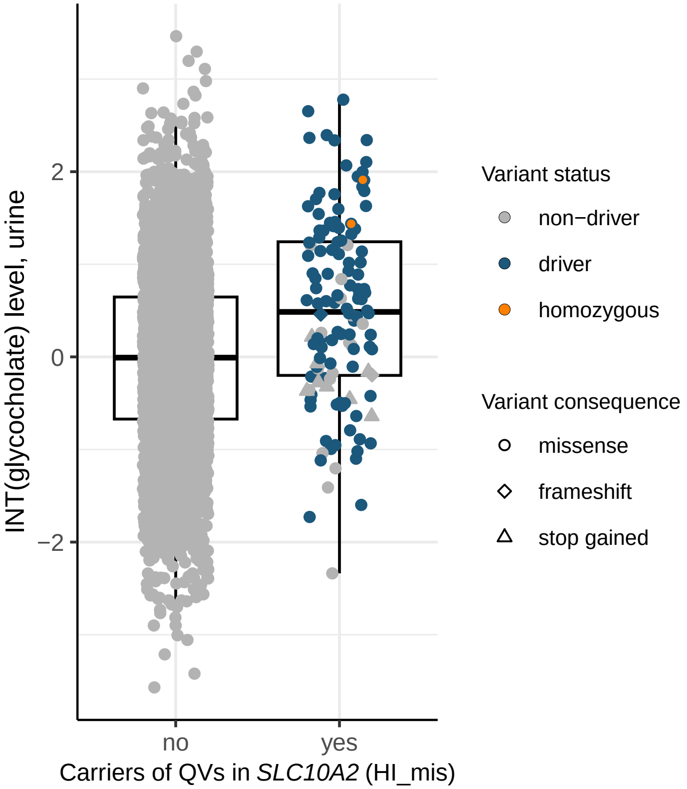
INT(glycocholate) level, urine
2
0
−2
no
yes
Carriers of QVs in *SLC10A2* (HI_mis)
Variant status
non−driver
driver
homozygous
Variant consequence
missense
frameshift
stop gained

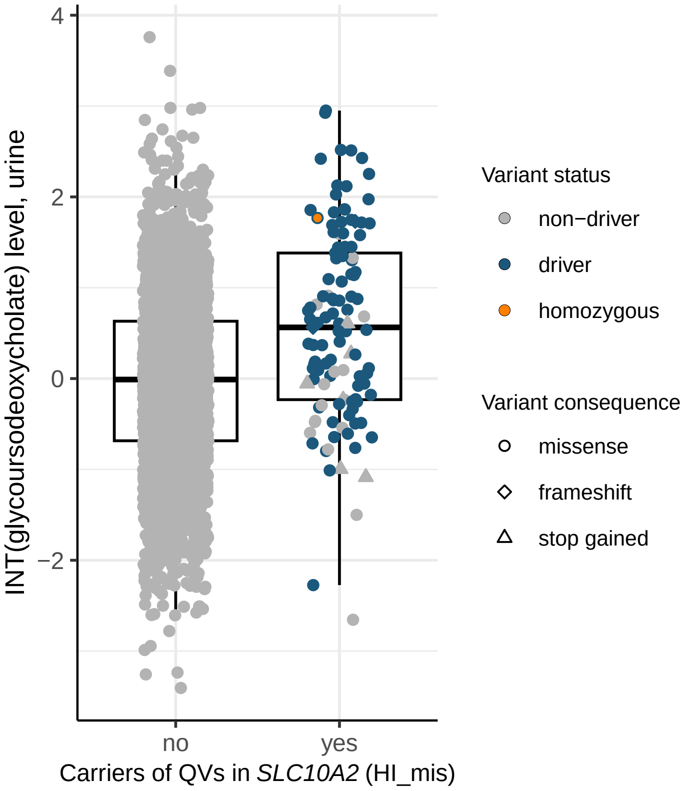
INT(glycoursodeoxycholate) level, urine
4
2
0
−2
no
yes
Carriers of QVs in *SLC10A2* (HI_mis)
Variant status
non−driver
driver
homozygous
Variant consequence
missense
frameshift
stop gained

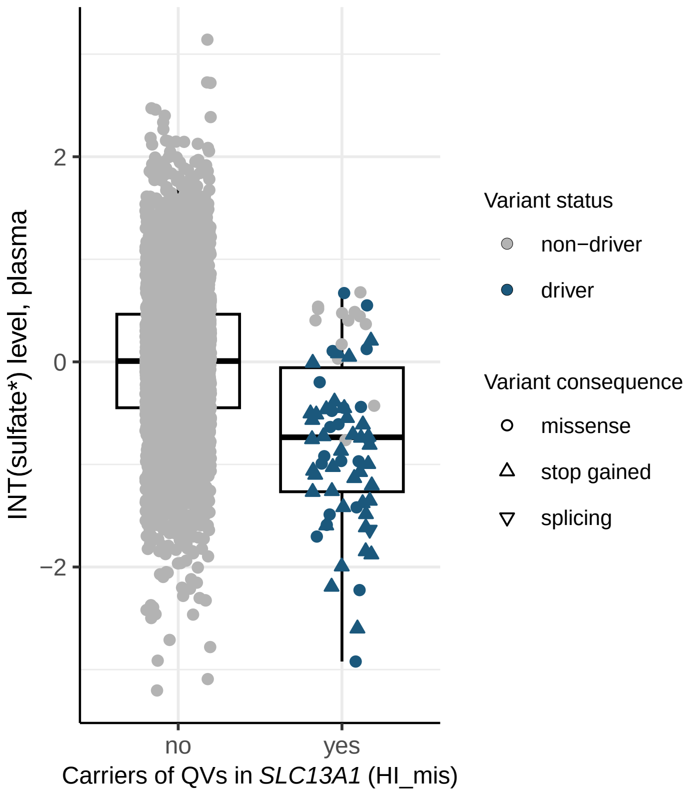
INT(sulfate*) level, plasma
2
0
−2
no
yes
Carriers of QVs in SLC13A1 (HI_mis)
Variant status
non−driver
driver
Variant consequence
missense
stop gained
splicing

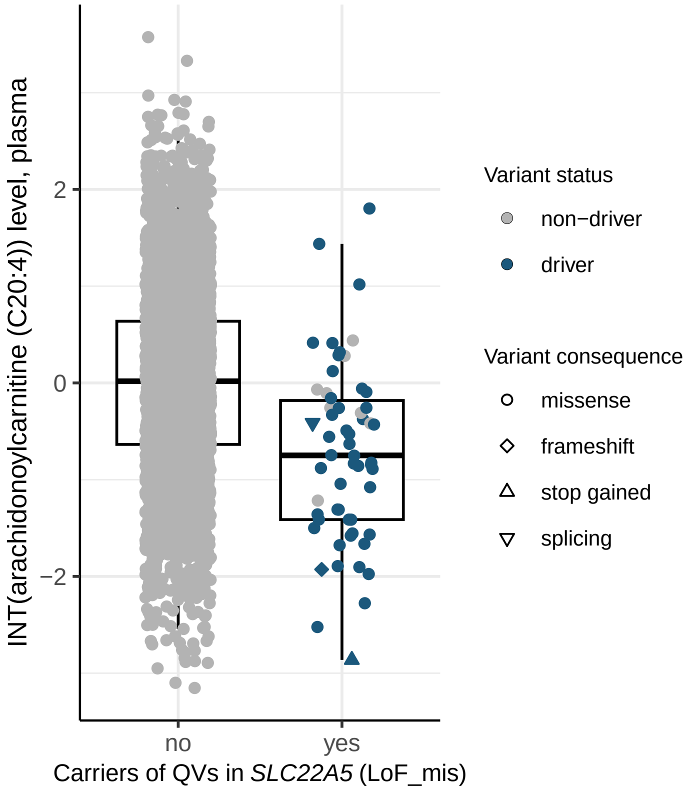
INT(arachidonoylcarnitine (C20:4)) level, plasma
2
0
−2
no
yes
Carriers of QVs in SLC22A5 (LoF_mis)
Variant status
non−driver
driver
Variant consequence
missense
frameshift
stop gained
splicing

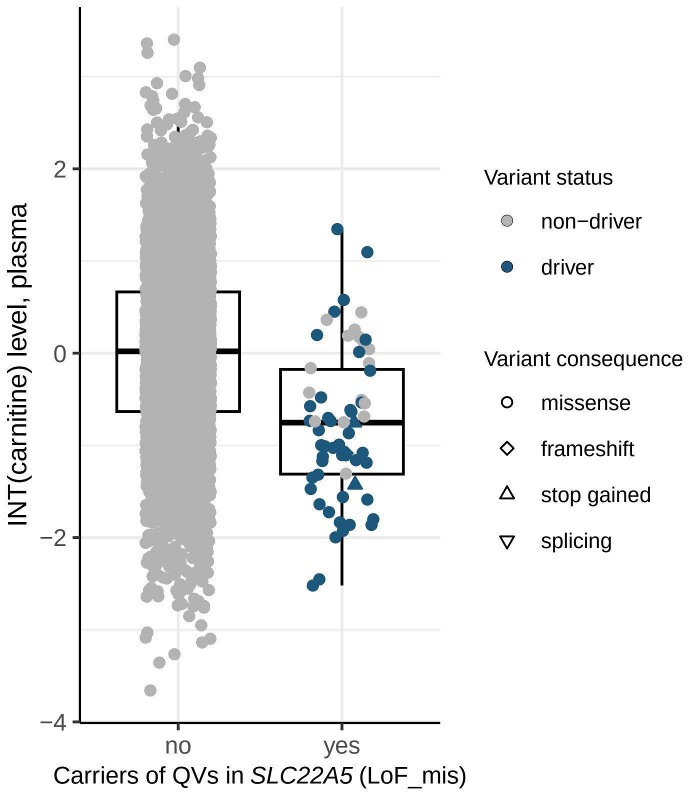

INT(carnitine) level, plasma
2
0
−2
−4
no
yes
Carriers of QVs in SLC22A5 (LoF_mis)
Variant status
non−driver
driver
Variant consequence
missense
frameshift
stop gained
splicing

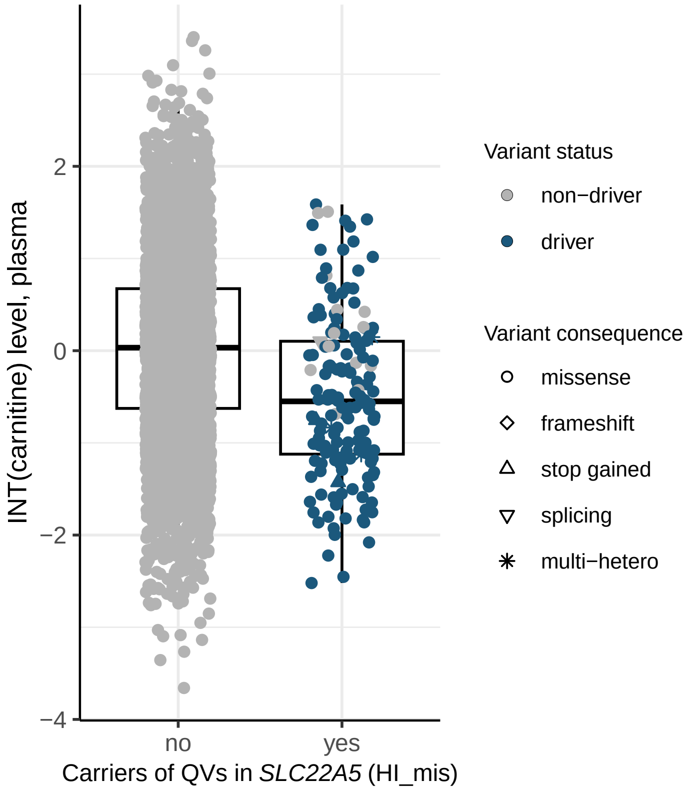

INT(carnitine) level, plasma
2
0
−2
−4
no
yes
Carriers of QVs in SLC22A5 (HI_mis)
Variant status
non−driver
driver
Variant consequence
missense
frameshift
stop gained
splicing
multi−hetero

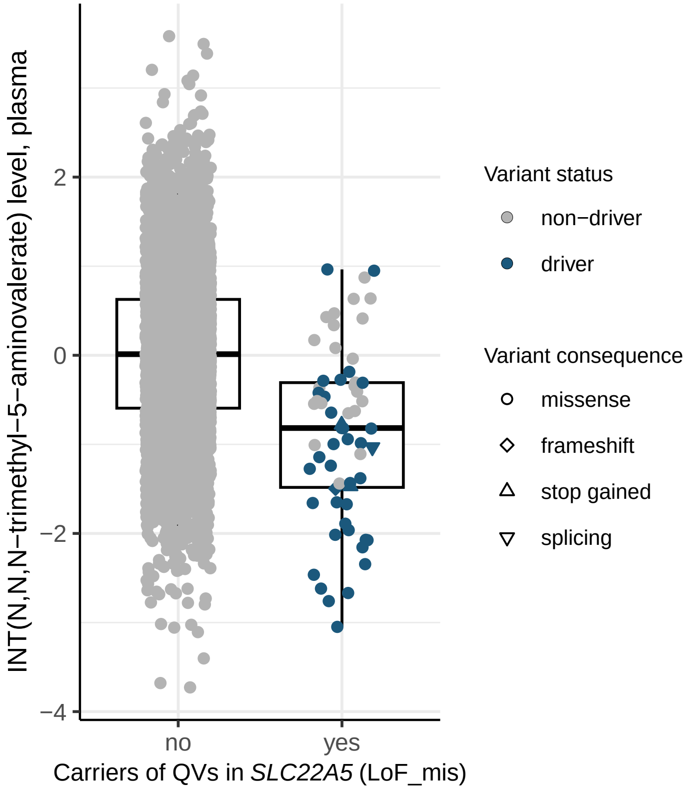
INT(N,N,N−trimethyl−5−aminovalerate) level, plasma
2
0
−2
−4
no
yes
Carriers of QVs in *SLC22A5* (LoF_mis)
Variant status
non−driver
driver
Variant consequence
missense
frameshift
stop gained
splicing

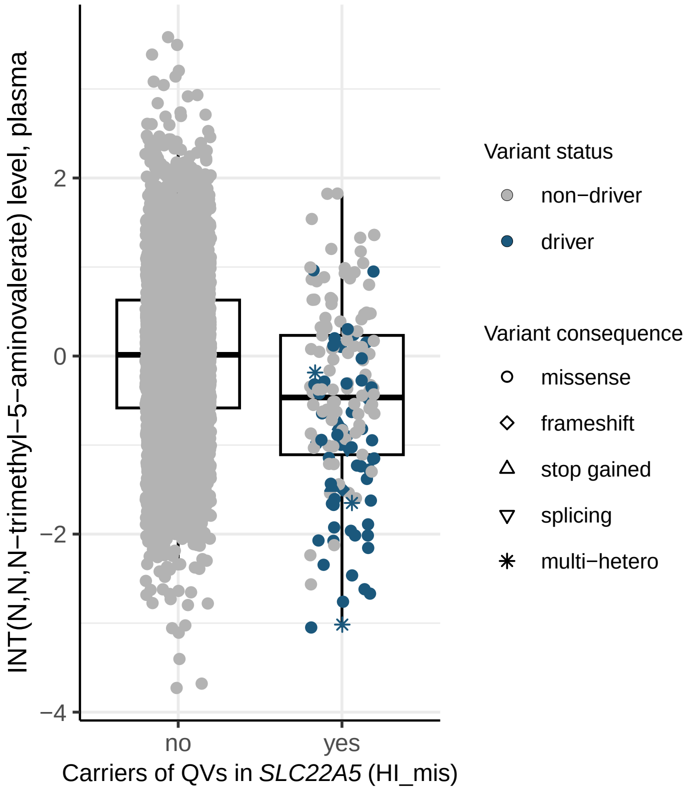
INT(N,N,N−trimethyl−5−aminovalerate) level, plasma
2
0
−2
−4
no
yes
Carriers of QVs in *SLC22A5* (HI_mis)
Variant status
non−driver
driver
Variant consequence
missense
frameshift
stop gained
splicing
multi−hetero

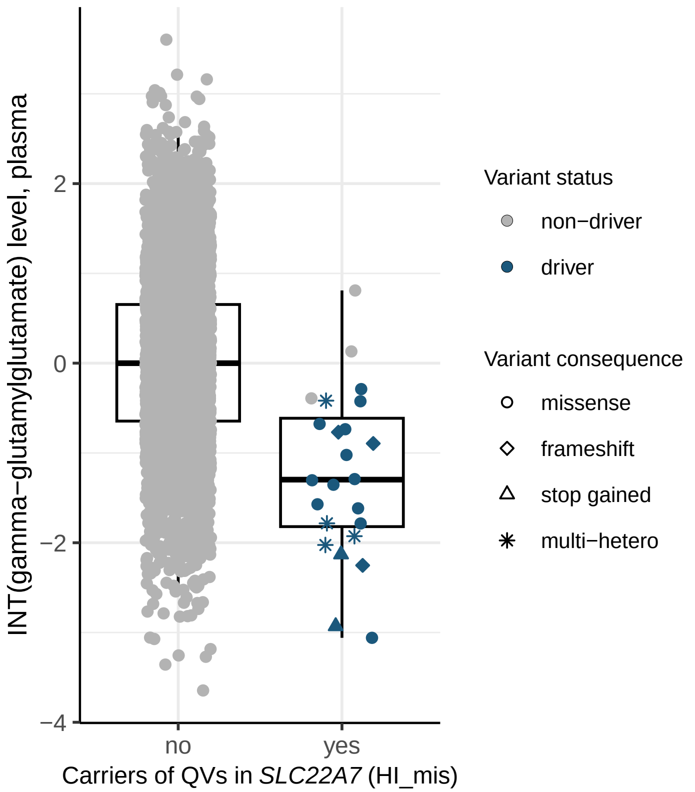
INT(gamma−glutamylglutamate) level, plasma
2
0
−2
−4
no
yes
Carriers of QVs in SLC22A7 (HI_mis)
Variant status
non−driver
driver
Variant consequence
missense
frameshift
stop gained
multi−hetero

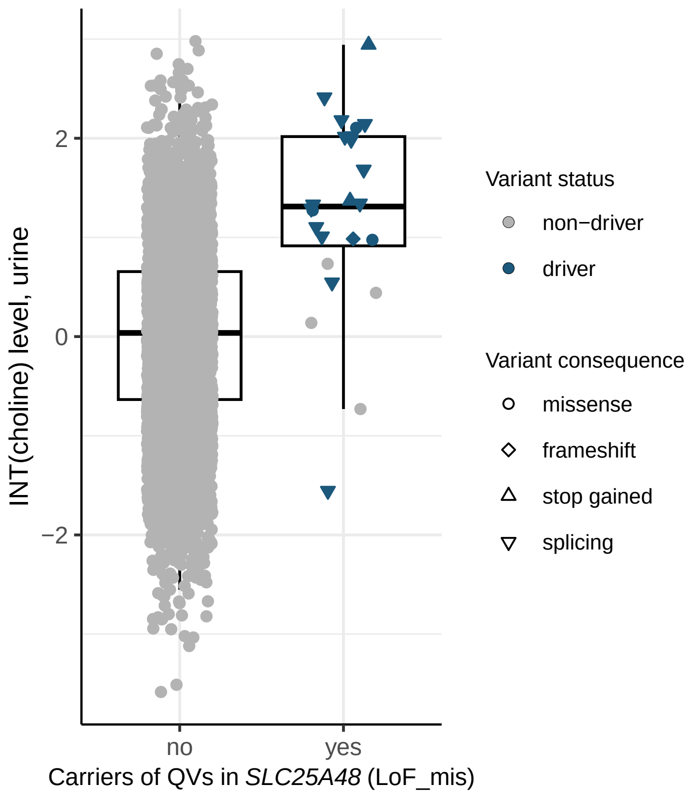
INT(choline) level, urine
2
0
−2
no
yes
Carriers of QVs in SLC25A48 (LoF_mis)
Variant status
non−driver
driver
Variant consequence
missense
frameshift
stop gained
splicing

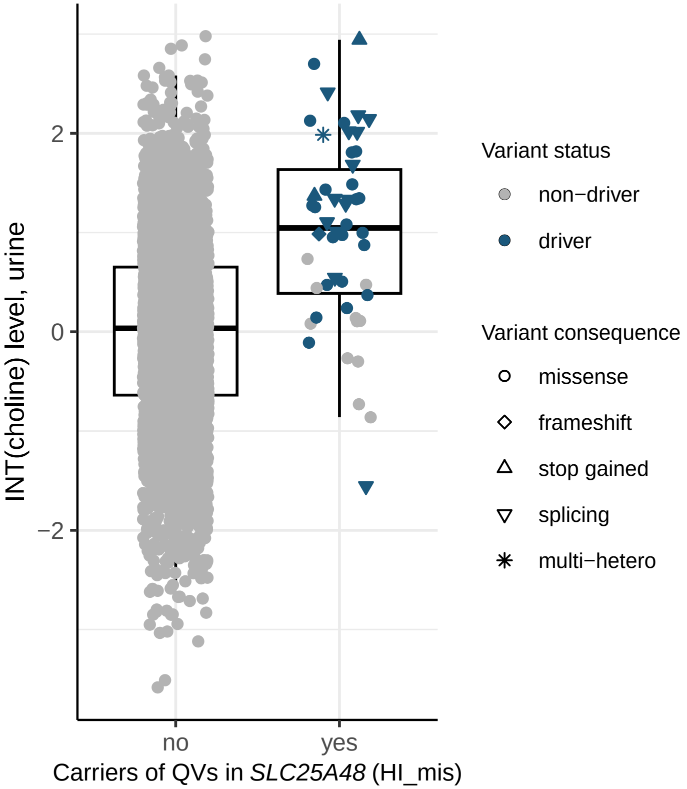
INT(choline) level, urine
2
0
−2
no
yes
Carriers of QVs in SLC25A48 (HI_mis)
Variant status
non−driver
driver
Variant consequence
missense
frameshift
stop gained
splicing
multi−hetero

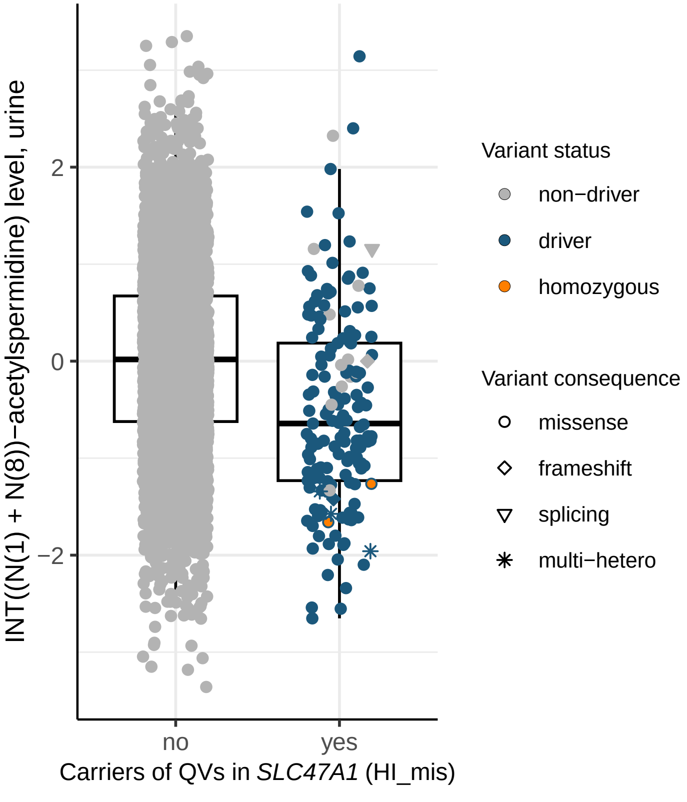
INT((N(1) + N(8))−acetylspermidine) level, urine
2
0
−2
no
yes
Carriers of QVs in SLC47A1 (HI_mis)
Variant status
non−driver
driver
homozygous
Variant consequence
missense
frameshift
splicing
multi−hetero

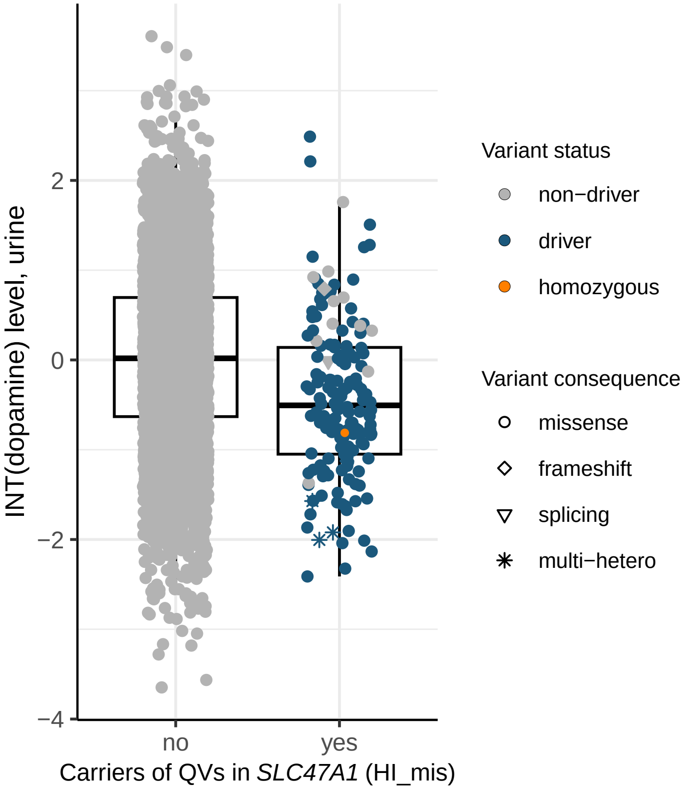
INT(dopamine) level, urine
2
0
−2
−4
no
yes
Carriers of QVs in SLC47A1 (HI_mis)
Variant status
non−driver
driver
homozygous
Variant consequence
missense
frameshift
splicing
multi−hetero

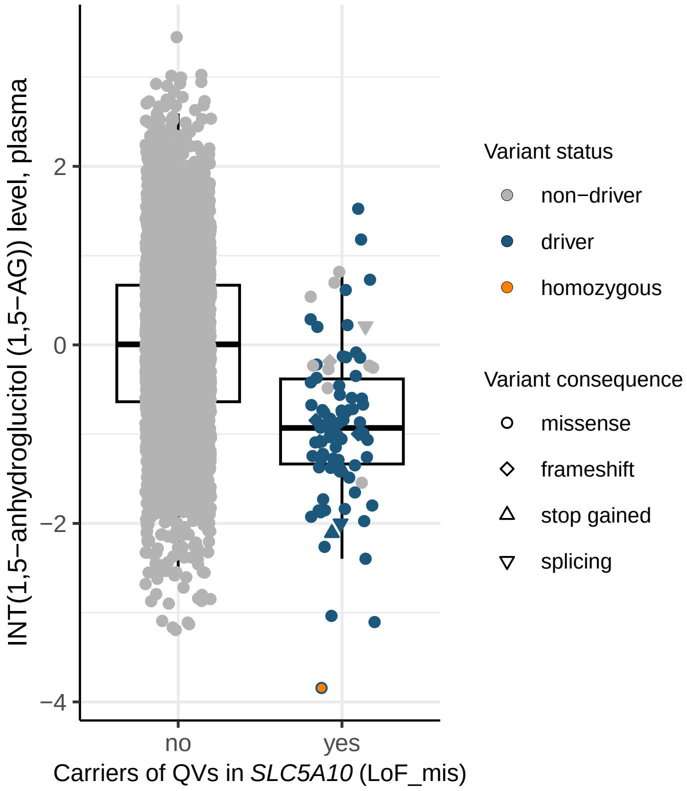
INT(1,5−anhydroglucitol (1,5−AG)) level, plasma
2
0
−2
−4
no
yes
Carriers of QVs in SLC5A10 (LoF_mis)
Variant status
non−driver
driver
homozygous
Variant consequence
missense
frameshift
stop gained
splicing

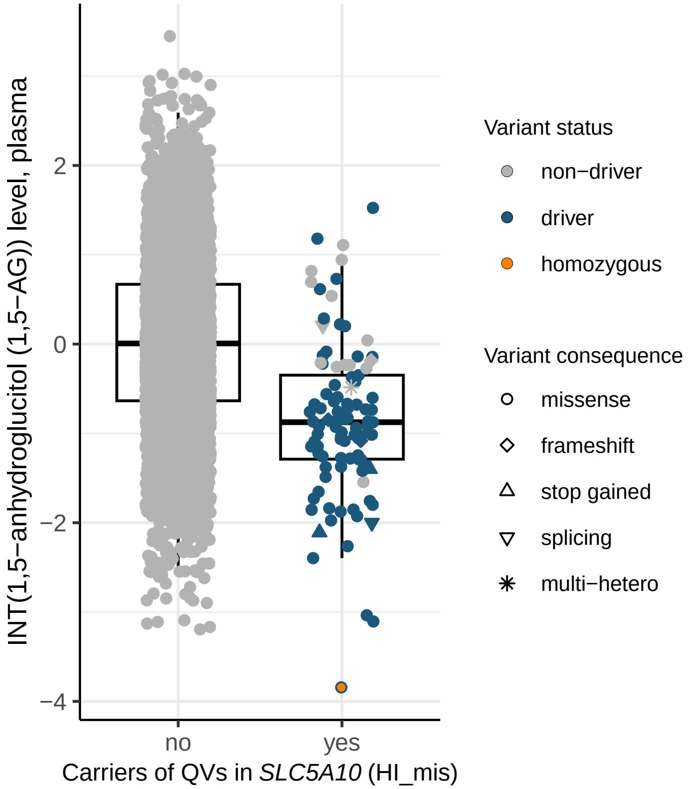

INT(1,5−anhydroglucitol (1,5−AG)) level, plasma
2
0
−2
−4
no
yes
Carriers of QVs in *SLC5A10* (HI_mis)
Variant status
non−driver
driver
homozygous
Variant consequence
missense
frameshift
stop gained
splicing
multi−hetero

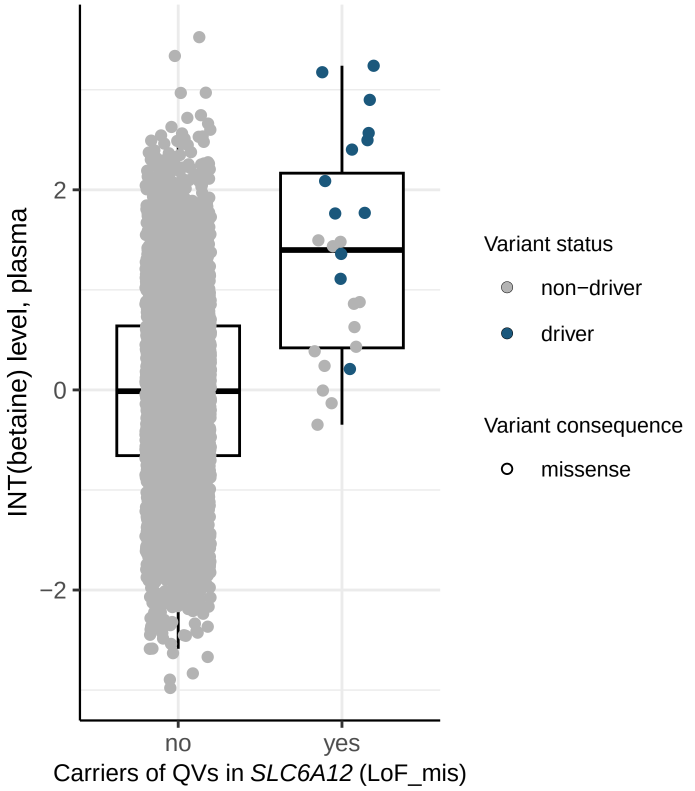

INT(betaine) level, plasma
2
0
−2
no
yes
Carriers of QVs in *SLC6A12* (LoF_mis)
Variant status
non−driver
driver
Variant consequence
missense

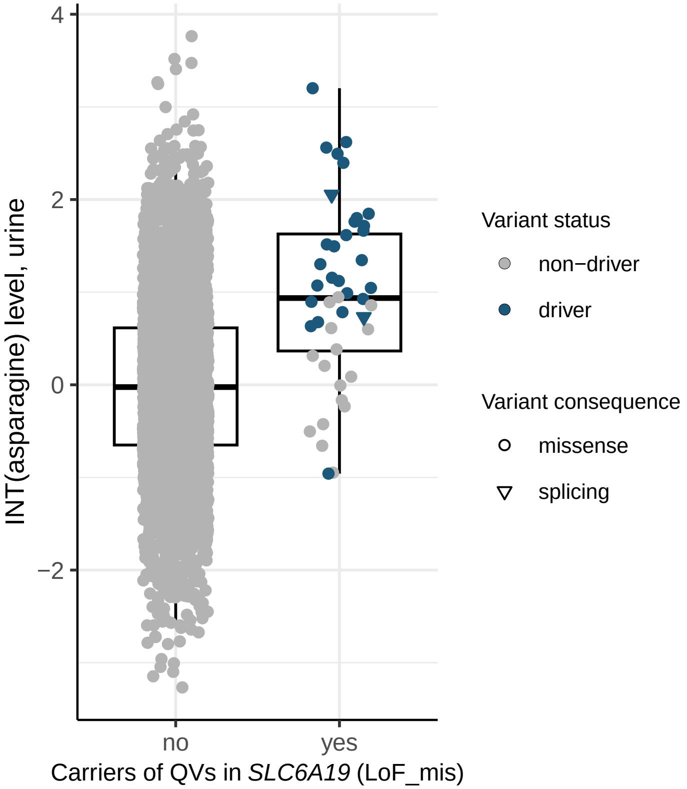
4
2
0
−2
INT(asparagine) level, urine
no
yes
Carriers of QVs in SLC6A19 (LoF_mis)
Variant status
non−driver
driver
Variant consequence
missense
splicing

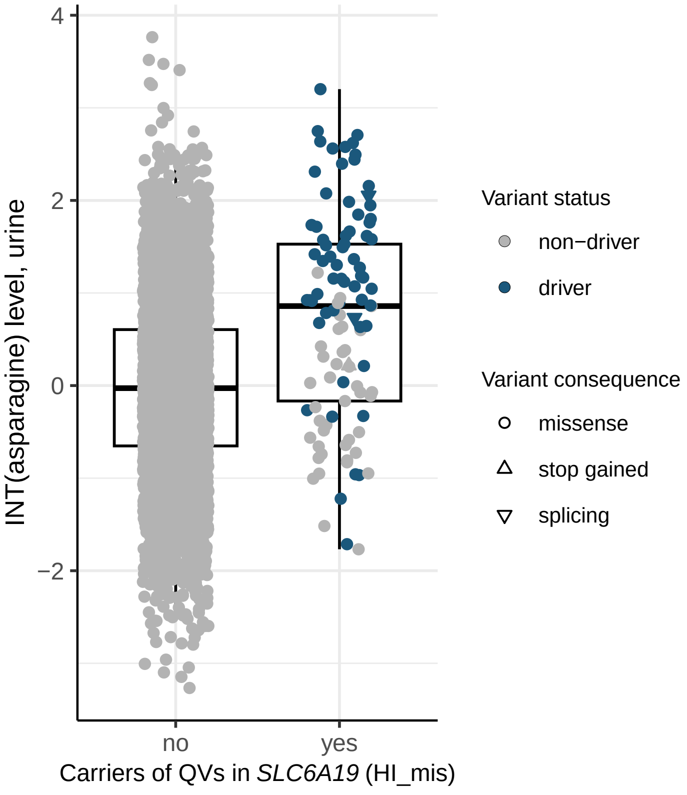
4
2
0
−2
INT(asparagine) level, urine
no
yes
Carriers of QVs in SLC6A19 (HI_mis)
Variant status
non−driver
driver
Variant consequence
missense
stop gained
splicing

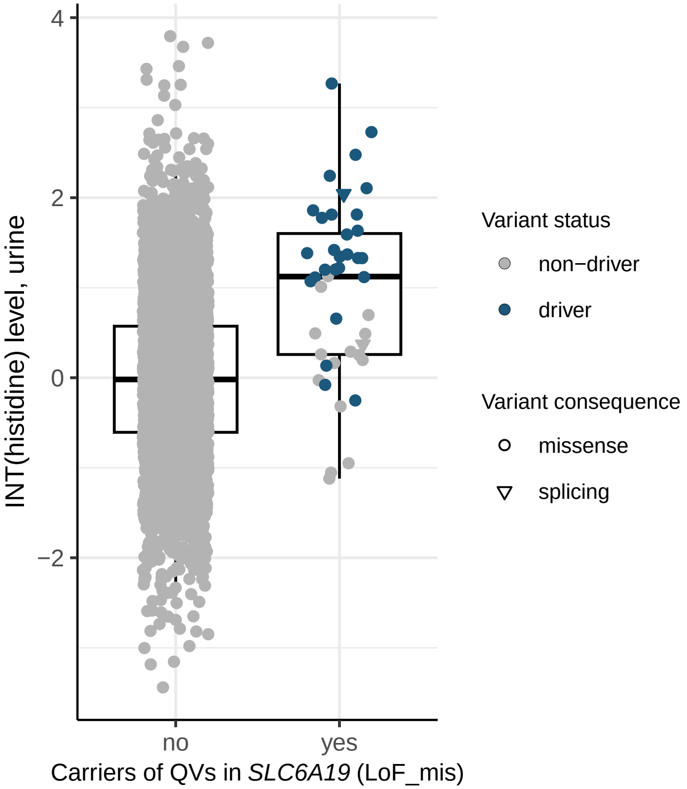
4
2
0
−2
INT(histidine) level, urine
no
yes
Carriers of QVs in SLC6A19 (LoF_mis)
Variant status
non−driver
driver
Variant consequence
missense
splicing

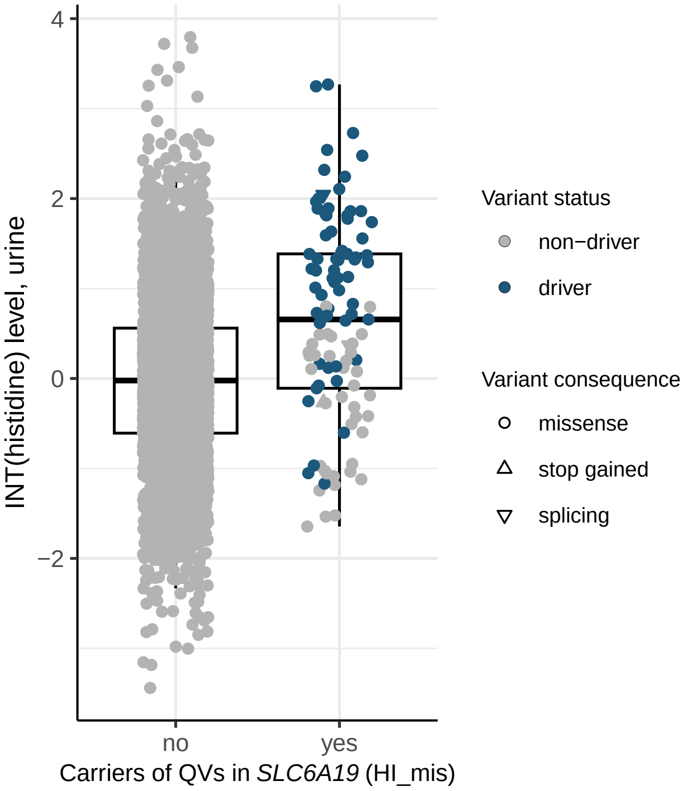
4
2
0
−2
INT(histidine) level, urine
no
yes
Carriers of QVs in SLC6A19 (HI_mis)
Variant status
non−driver
driver
Variant consequence
missense
stop gained
splicing

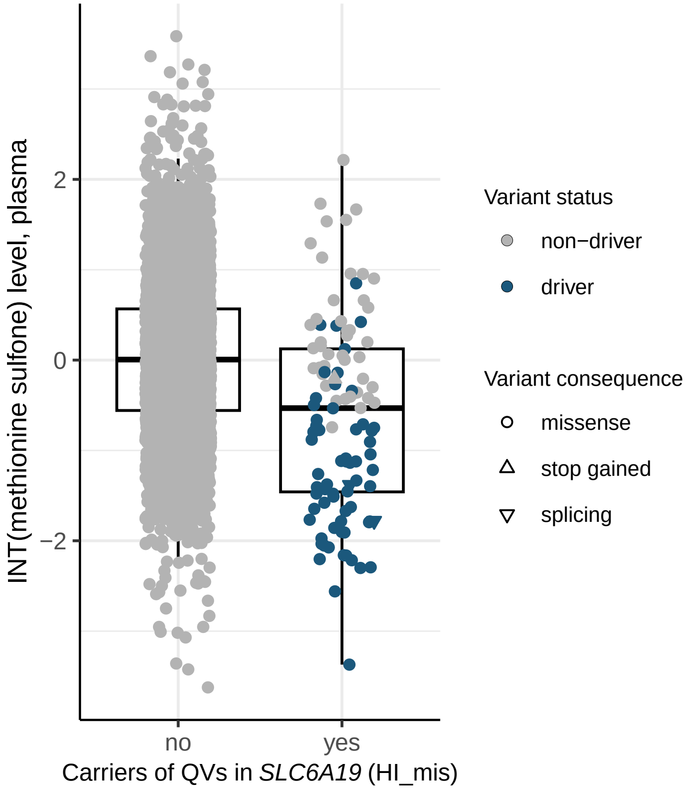
INT(methionine sulfone) level, plasma
2
0
−2
no
yes
Carriers of QVs in *SLC6A19* (HI_mis)
Variant status
non−driver
driver
Variant consequence
missense
stop gained
splicing

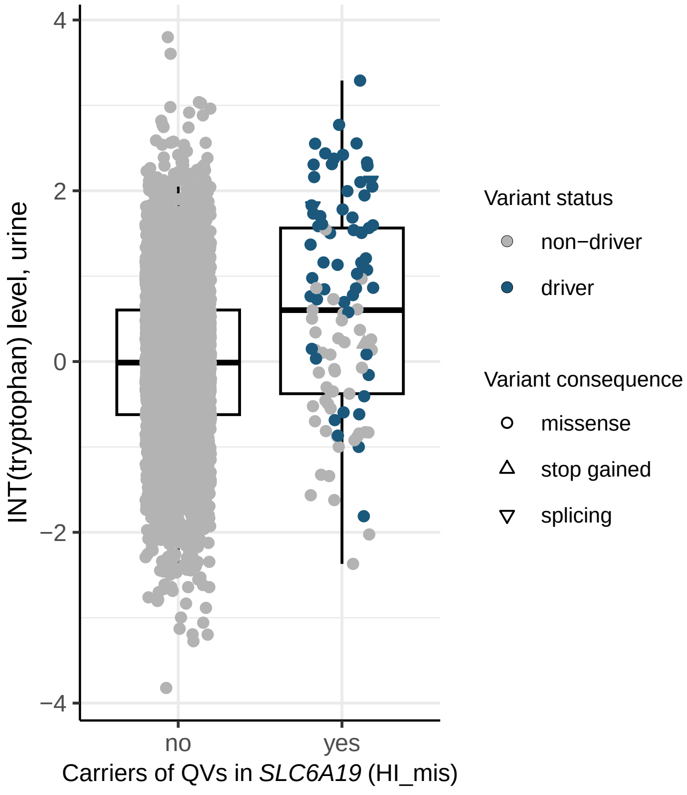
INT(tryptophan) level, urine
4
2
0
−2
−4
no
yes
Carriers of QVs in *SLC6A19* (HI_mis)
Variant status
non−driver
driver
Variant consequence
missense
stop gained
splicing

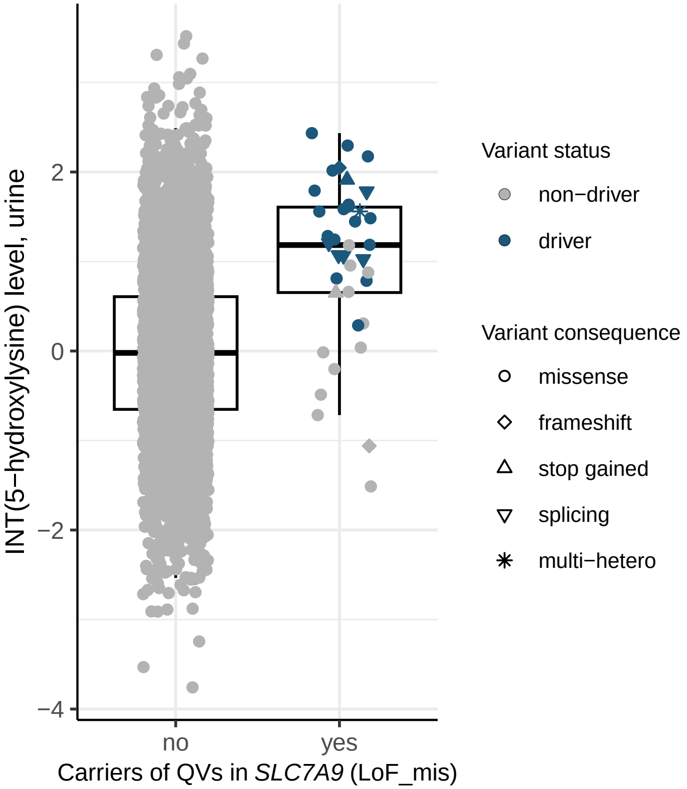
INT(5−hydroxylysine) level, urine
2
0
−2
−4
no
yes
Carriers of QVs in SLC7A9 (LoF_mis)
Variant status
non−driver
driver
Variant consequence
missense
frameshift
stop gained
splicing
multi−hetero

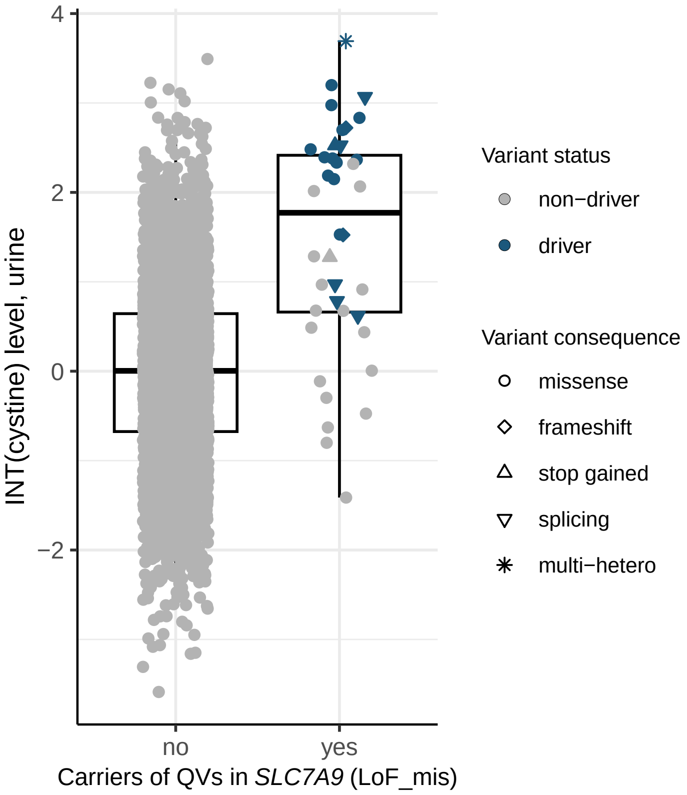
INT(cystine) level, urine
4
2
0
−2
no
yes
Carriers of QVs in SLC7A9 (LoF_mis)
Variant status
non−driver
driver
Variant consequence
missense
frameshift
stop gained
splicing
multi−hetero

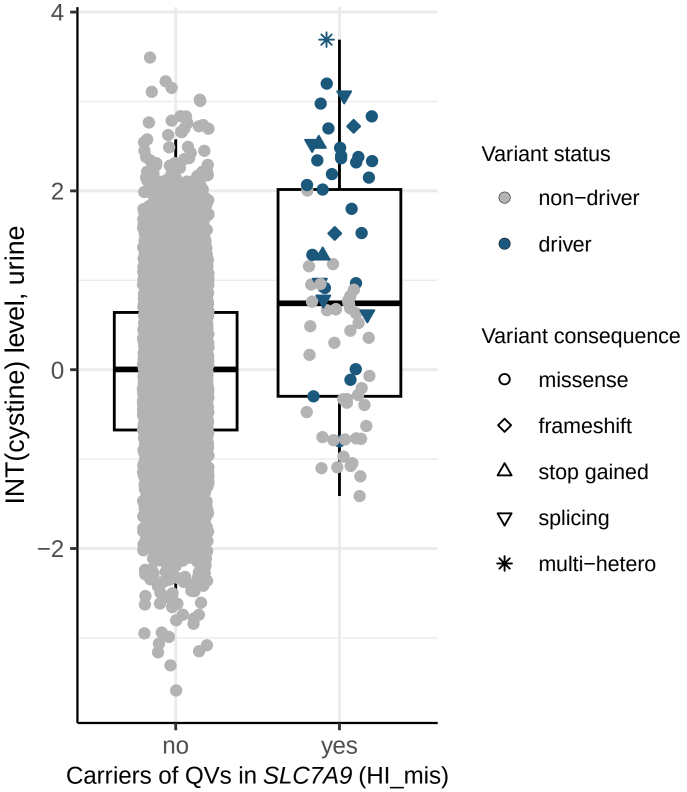
4
2
0
−2
INT(cystine) level, urine
no
yes
Carriers of QVs in SLC7A9 (HI_mis)
Variant status
non−driver
driver
Variant consequence
missense
frameshift
stop gained
splicing
multi−hetero

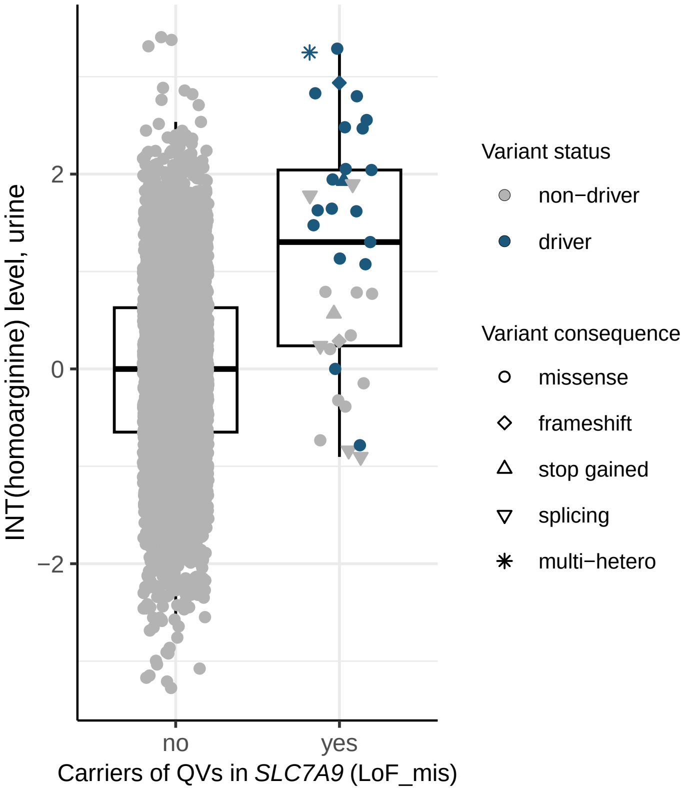
2
0
−2
INT(homoarginine) level, urine
no
yes
Carriers of QVs in SLC7A9 (LoF_mis)
Variant status
non−driver
driver
Variant consequence
missense
frameshift
stop gained
splicing
multi−hetero

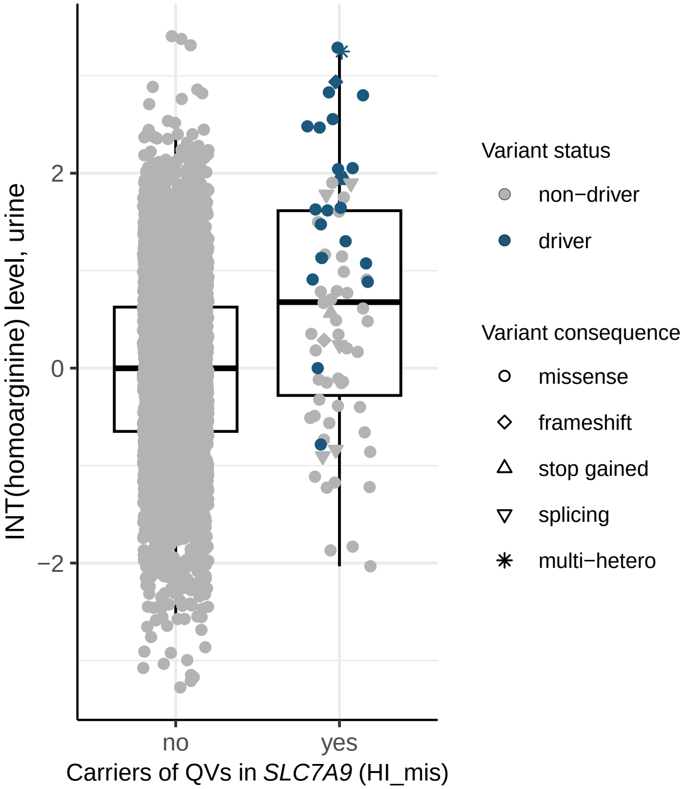
INT(homoarginine) level, urine
2
0
−2
no
yes
Carriers of QVs in SLC7A9 (HI_mis)
Variant status
non−driver
driver
Variant consequence
missense
frameshift
stop gained
splicing
multi−hetero

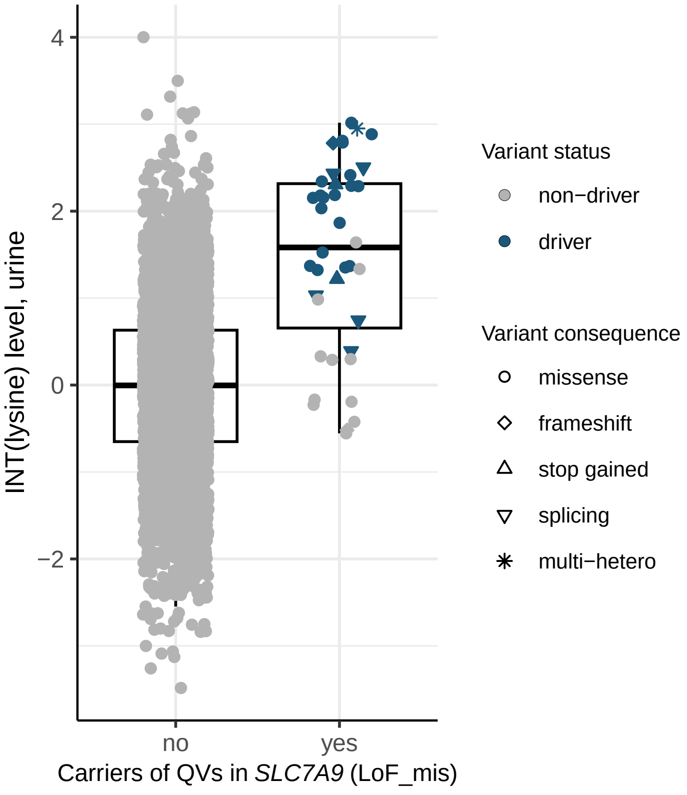
INT(lysine) level, urine
4
2
0
−2
no
yes
Carriers of QVs in SLC7A9 (LoF_mis)
Variant status
non−driver
driver
Variant consequence
missense
frameshift
stop gained
splicing
multi−hetero

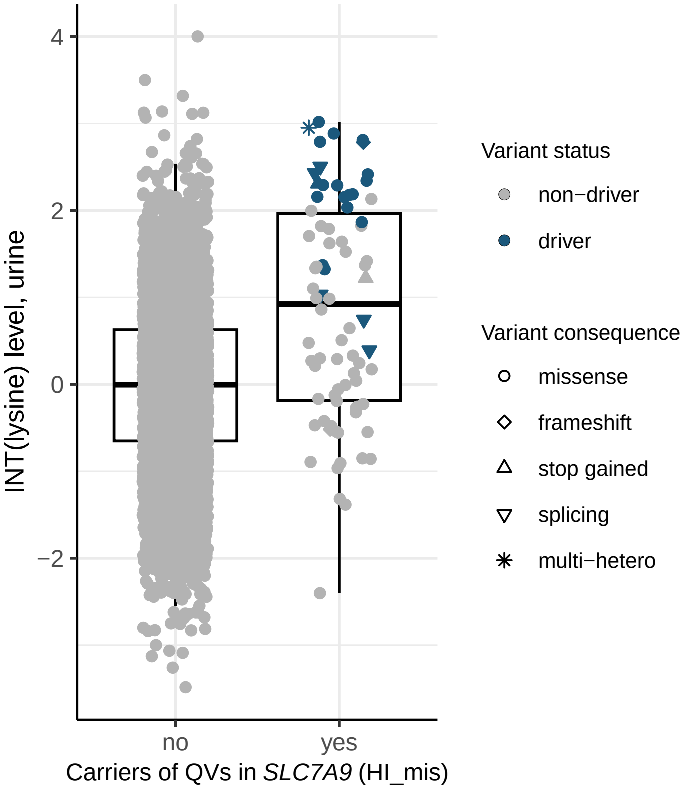
INT(lysine) level, urine
4
2
0
−2
no
yes
Carriers of QVs in *SLC7A9* (HI_mis)
Variant status
non−driver
driver
Variant consequence
missense
frameshift
stop gained
splicing
multi−hetero

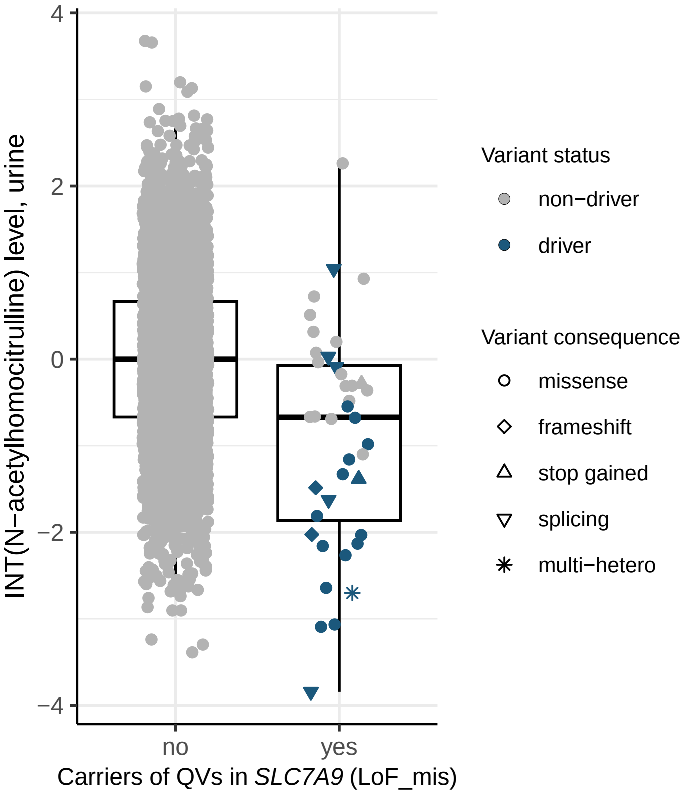
INT(N−acetylhomocitrulline) level, urine
4
2
0
−2
−4
no
yes
Carriers of QVs in *SLC7A9* (LoF_mis)
Variant status
non−driver
driver
Variant consequence
missense
frameshift
stop gained
splicing
multi−hetero

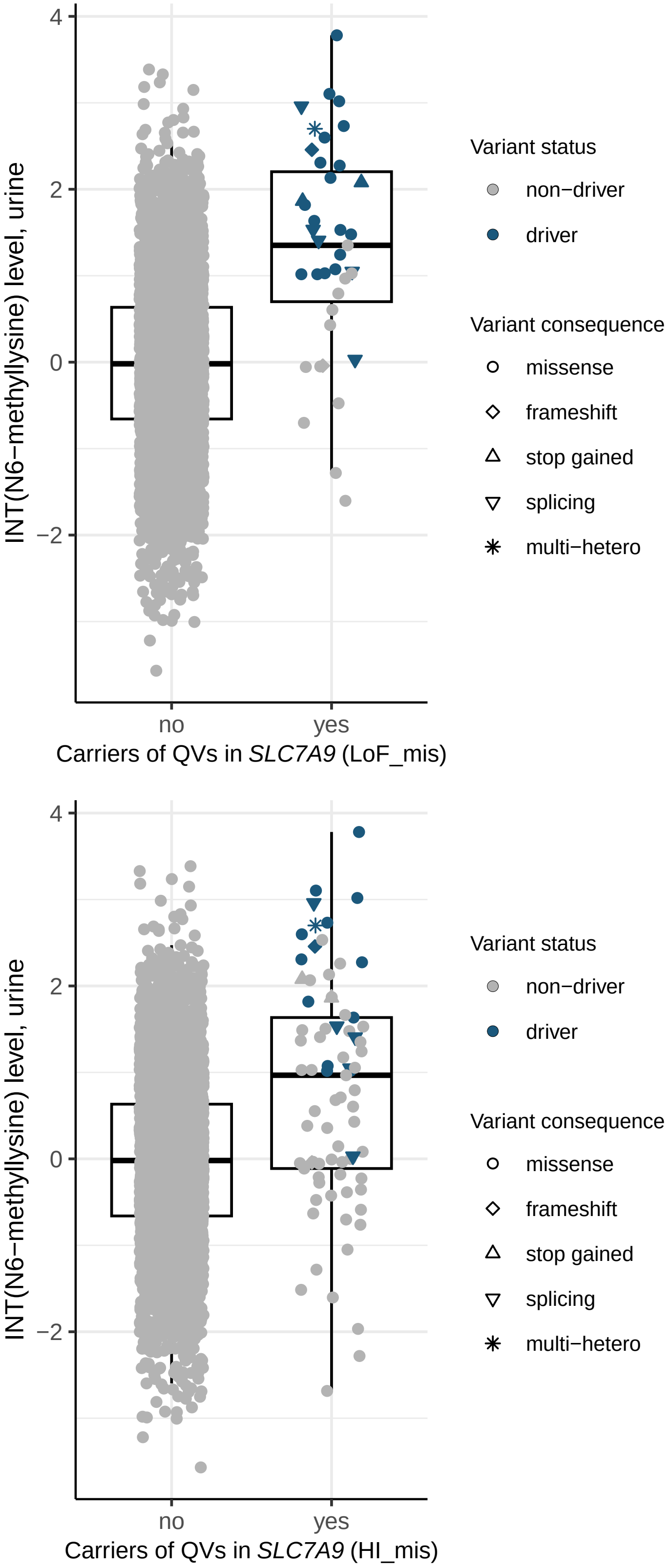

INT(N6−methyllysine) level, urine
4
2
0
−2
no
yes
Carriers of QVs in SLC7A9 (LoF_mis)
Variant status
non−driver
driver
Variant consequence
missense
frameshift
stop gained
splicing
multi−hetero
INT(N6−methyllysine) level, urine
4
2
0
−2
no
yes
Carriers of QVs in SLC7A9 (HI_mis)
Variant status
non−driver
driver
Variant consequence
missense
frameshift
stop gained
splicing
multi−hetero

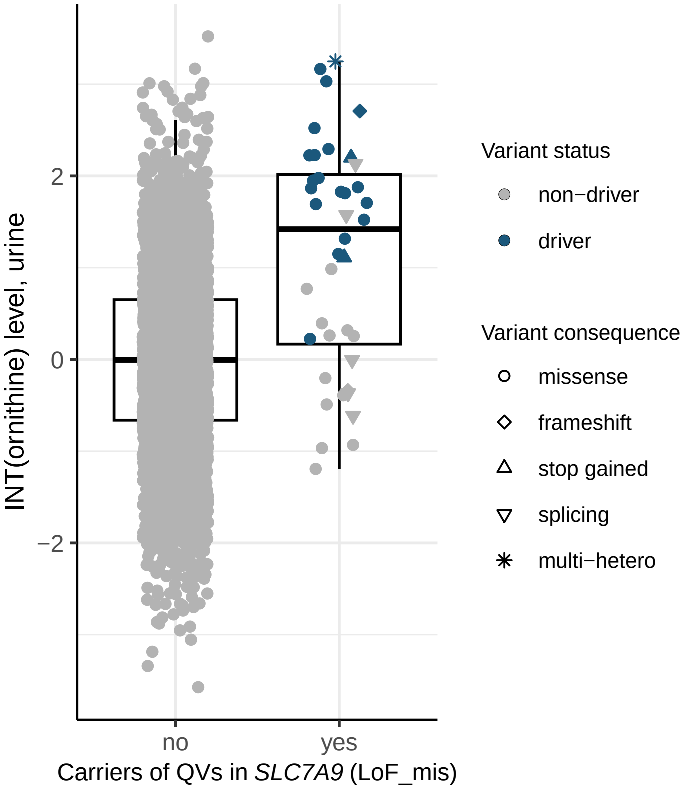
INT(ornithine) level, urine
2
0
−2
no
yes
Carriers of QVs in *SLC7A9* (LoF_mis)
Variant status
non−driver
driver
Variant consequence
missense
frameshift
stop gained
splicing
multi−hetero

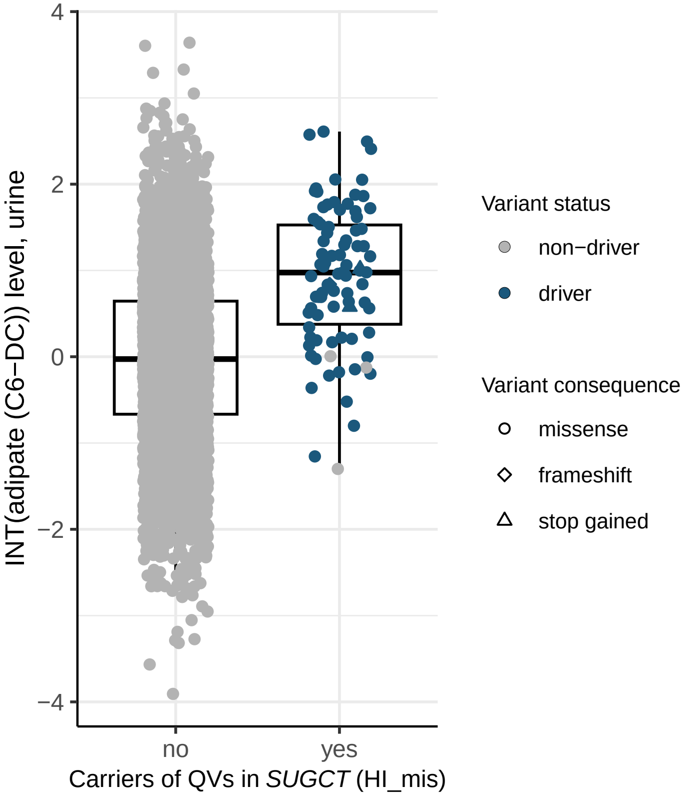
INT(adipate (C6−DC)) level, urine
4
2
0
−2
−4
no
yes
Carriers of QVs in *SUGCT* (HI_mis)
Variant status
non−driver
driver
Variant consequence
missense
frameshift
stop gained

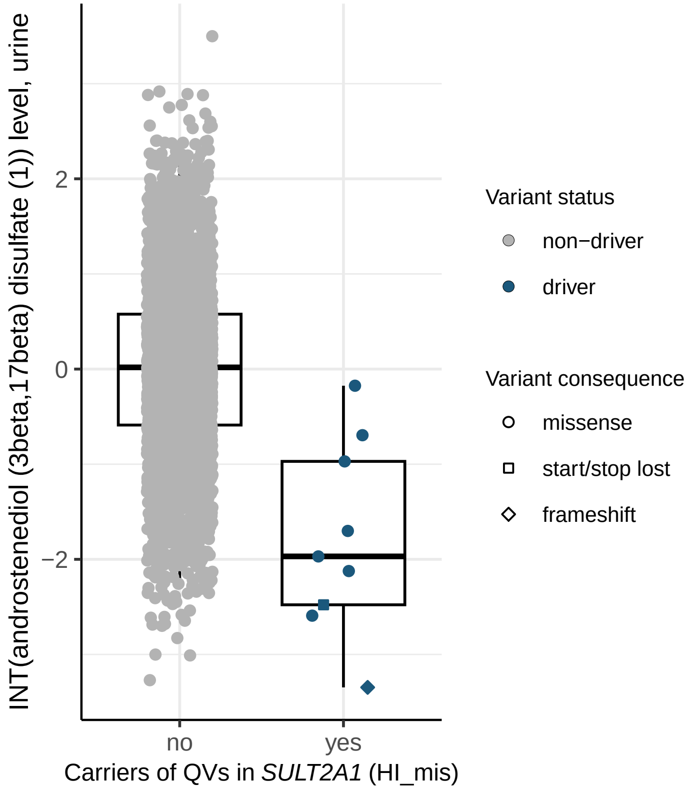
INT(androstenediol (3beta,17beta) disulfate (1)) level, urine
2
0
−2
no
yes
Carriers of QVs in SULT2A1 (HI_mis)
Variant status
non−driver
driver
Variant consequence
missense
start/stop lost
frameshift

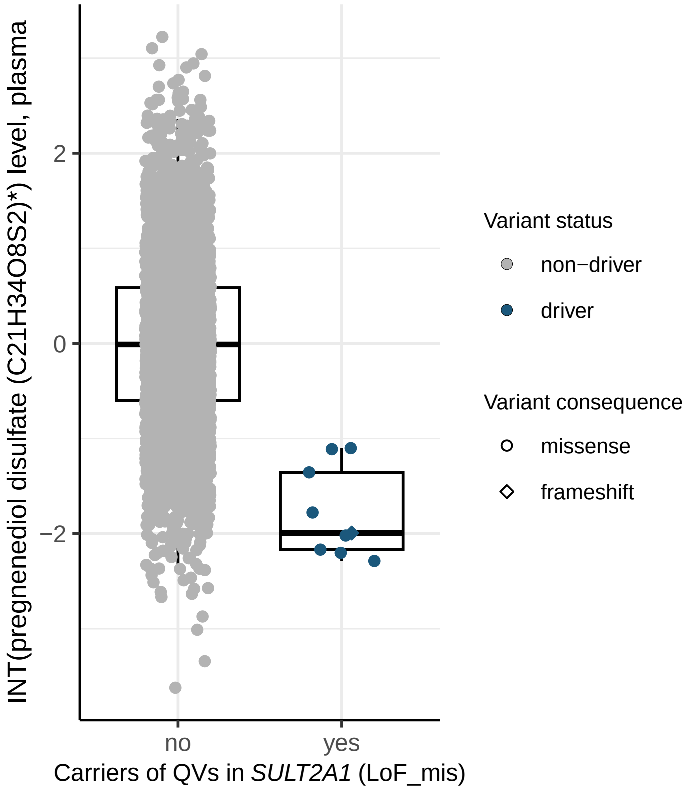
INT(pregnenediol disulfate (C21H34O8S2)*) level, plasma
2
0
−2
no
yes
Carriers of QVs in SULT2A1 (LoF_mis)
Variant status
non−driver
driver
Variant consequence
missense
frameshift

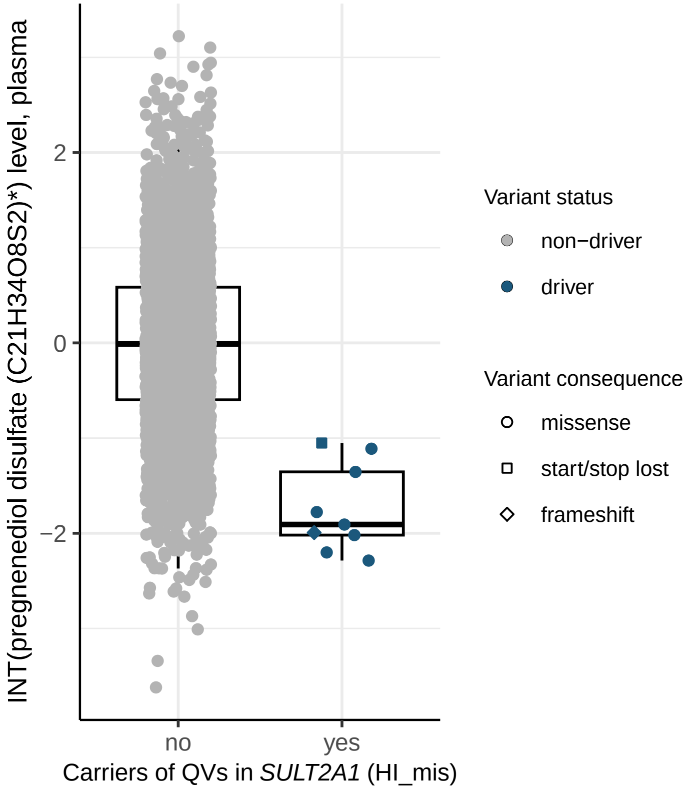
INT(pregnenediol disulfate (C21H34O8S2)*) level, plasma
2
0
−2
no
yes
Carriers of QVs in SULT2A1 (HI_mis)
Variant status
non−driver
driver
Variant consequence
missense
start/stop lost
frameshift

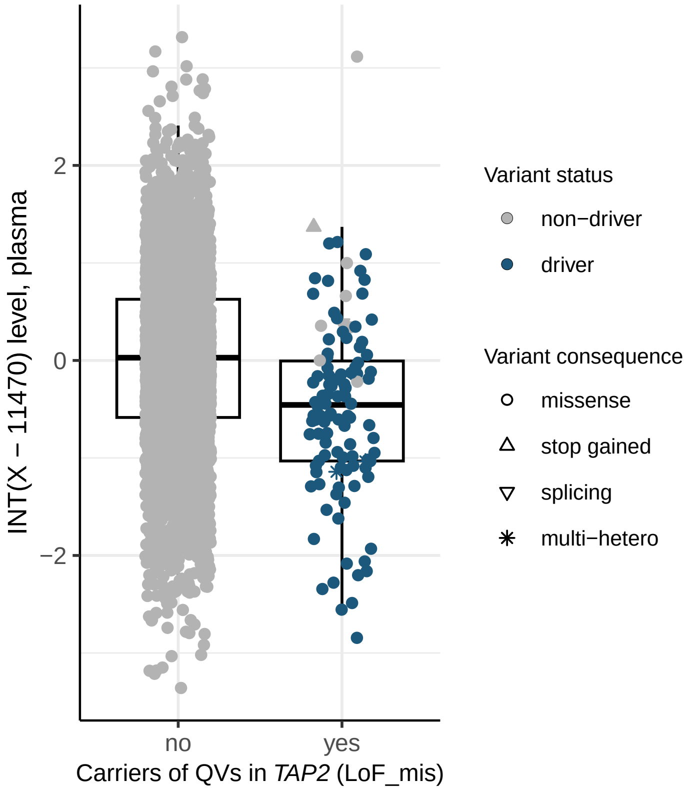
INT(X − 11470) level, plasma
2
0
−2
no
yes
Carriers of QVs in TAP2 (LoF_mis)
Variant status
non−driver
driver
Variant consequence
missense
stop gained
splicing
multi−hetero

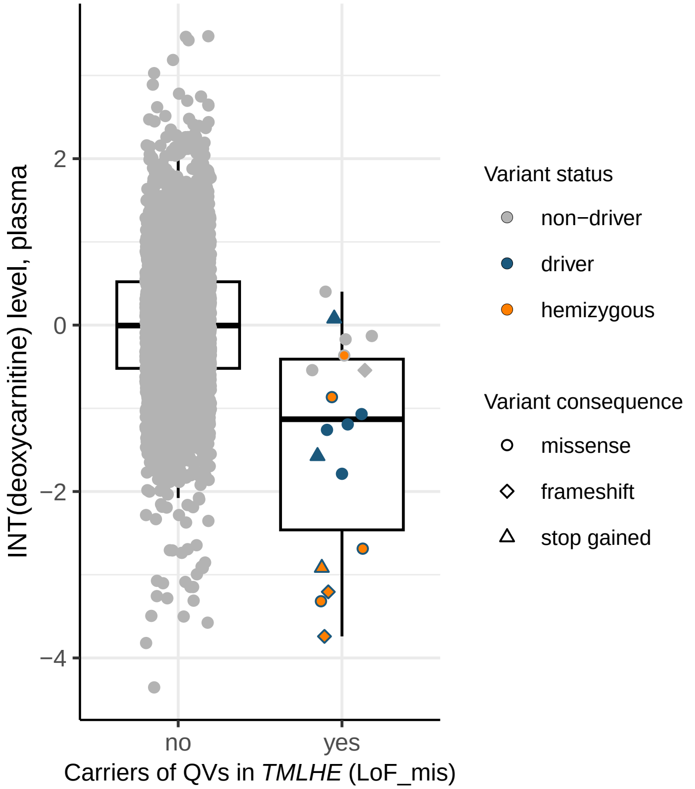
INT(deoxycarnitine) level, plasma
2
0
−2
−4
no
yes
Carriers of QVs in *TMLHE* (LoF_mis)
Variant status
non−driver
driver
hemizygous
Variant consequence
missense
frameshift
stop gained

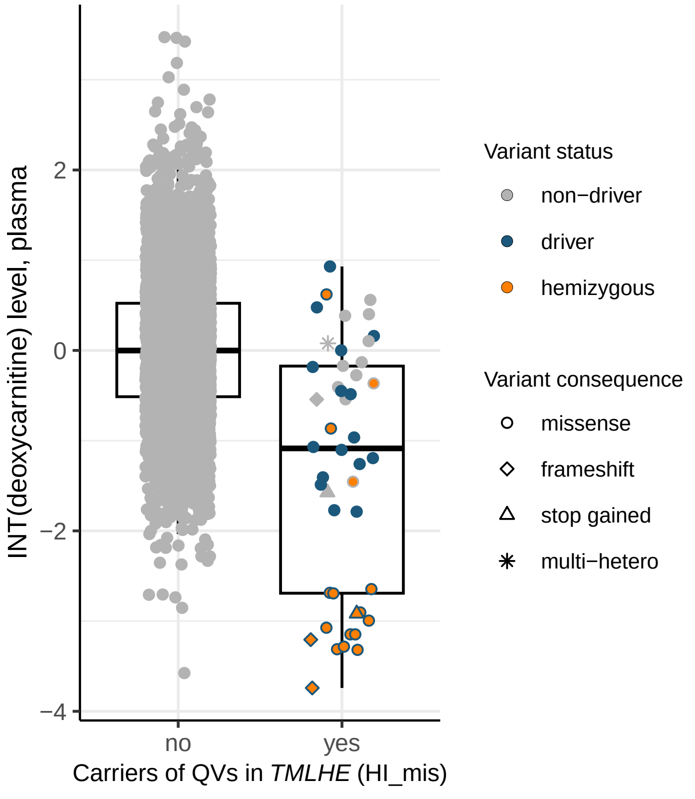
INT(deoxycarnitine) level, plasma
2
0
−2
−4
no
yes
Carriers of QVs in *TMLHE* (HI_mis)
Variant status
non−driver
driver
hemizygous
Variant consequence
missense
frameshift
stop gained
multi−hetero

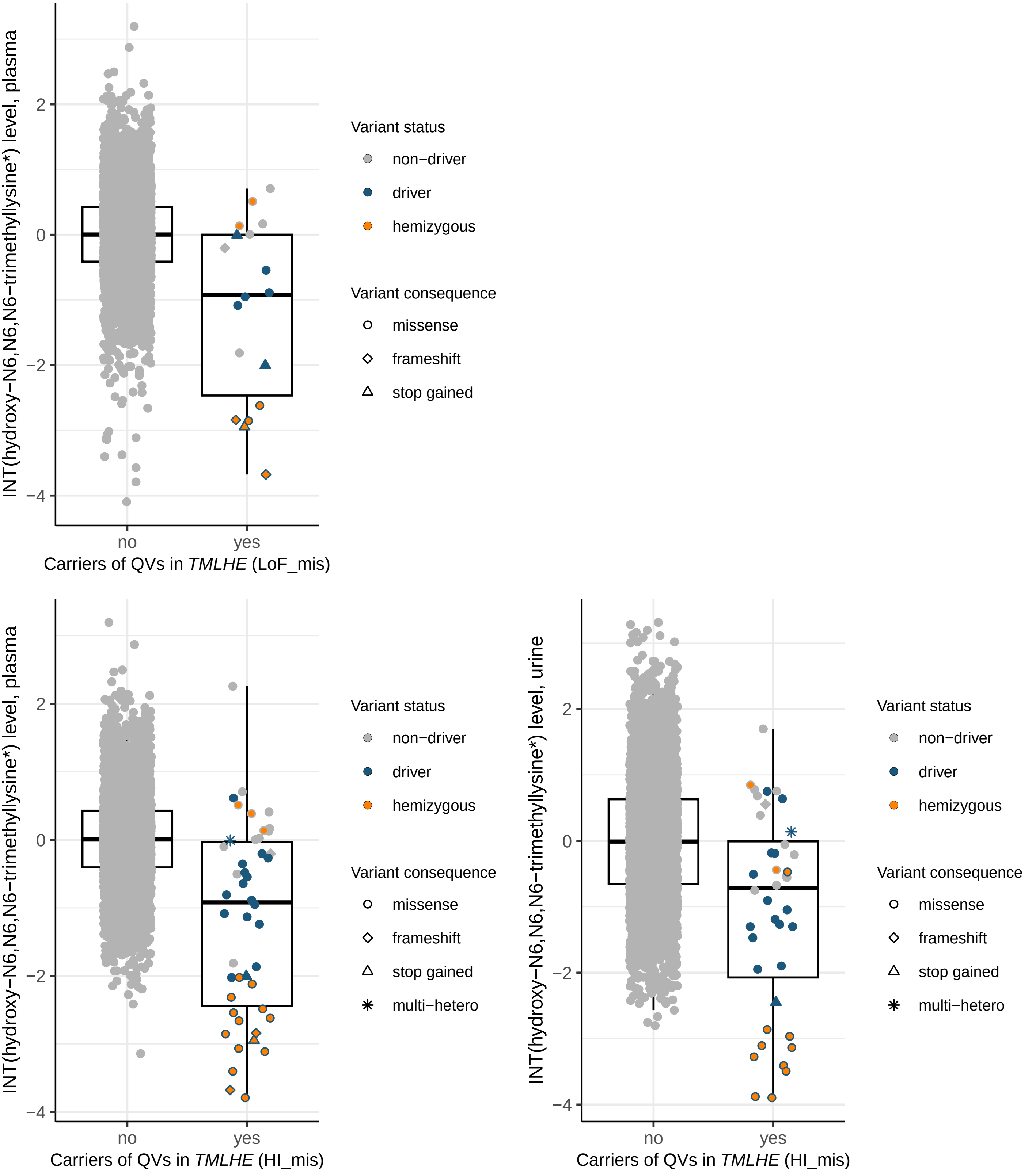

INT(hydroxy−N6,N6,N6−trimethyllysine*) level, plasma
2
0
−2
−4
no
yes
Carriers of QVs in *TMLHE* (LoF_mis)
Variant status
non−driver
driver
hemizygous
Variant consequence
missense
frameshift
stop gained
INT(hydroxy−N6,N6,N6−trimethyllysine*) level, plasma
Carriers of QVs in *TMLHE* (HI_mis)
multi−hetero
INT(hydroxy−N6,N6,N6−trimethyllysine*) level, urine
Carriers of QVs in *TMLHE* (HI_mis)

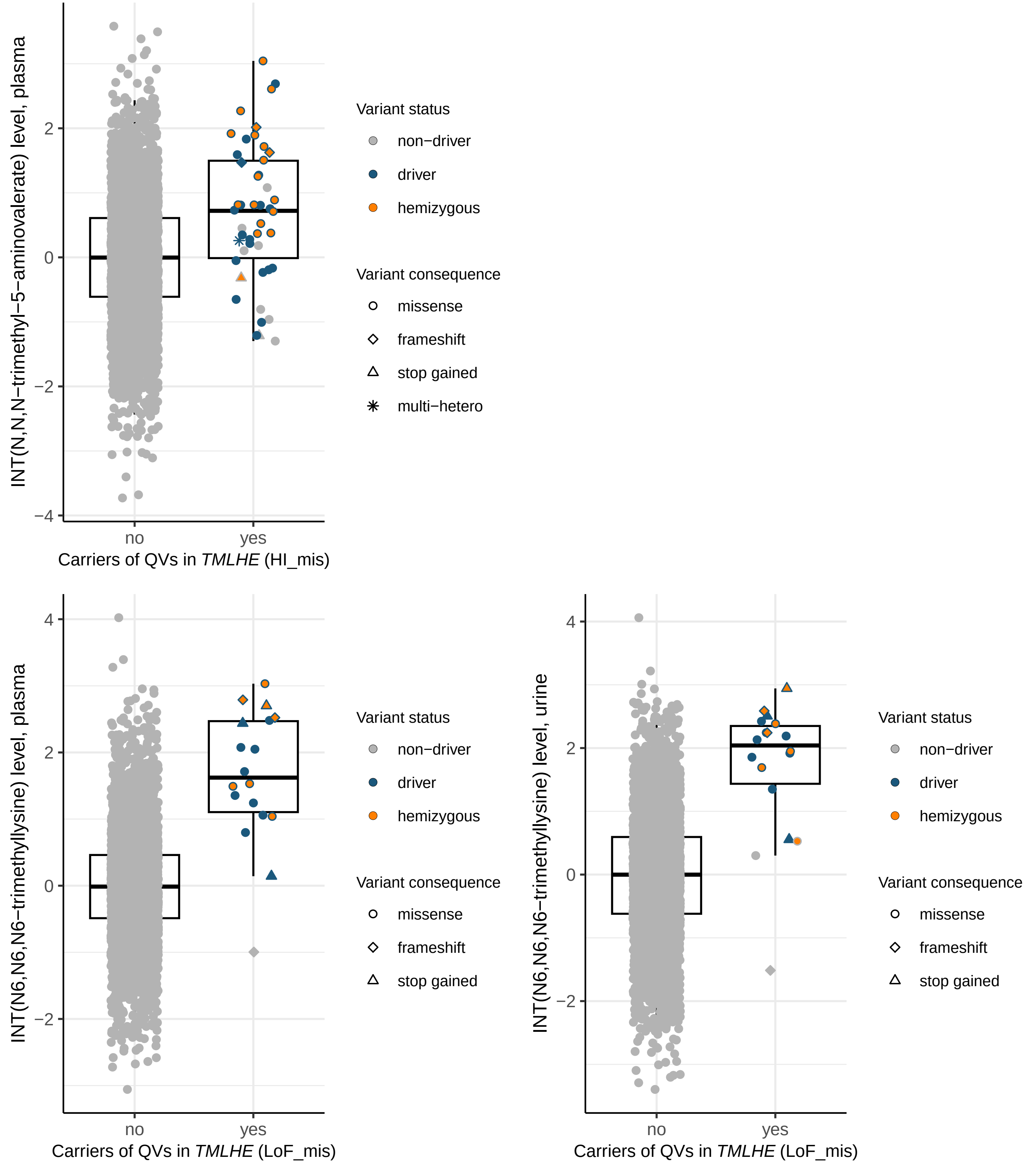

INT(N,N,N-trimethyl-5-aminovalerate) level, plasma
−4
−2
0
2
no
yes
Carriers of QVs in *TMLHE* (HI_mis)
Variant status
non−driver
driver
hemizygous
Variant consequence
missense
frameshift
stop gained
multi−hetero
INT(N6,N6,N6-trimethyllysine) level, plasma
−2
0
2
4
no
yes
Carriers of QVs in *TMLHE* (LoF_mis)
Variant status
non−driver
driver
hemizygous
Variant consequence
missense
frameshift
stop gained
INT(N6,N6,N6-trimethyllysine) level, urine
−2
0
2
4
no
yes
Carriers of QVs in *TMLHE* (LoF_mis)
Variant status
non−driver
driver
hemizygous
Variant consequence
missense
frameshift
stop gained

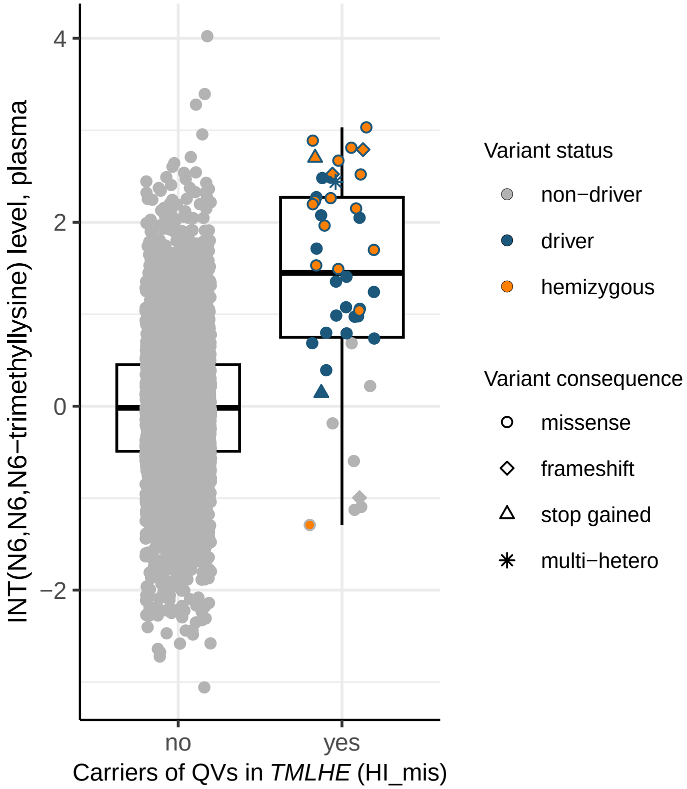
INT(N6,N6,N6−trimethyllysine) level, plasma
4
2
0
−2
no
yes
Carriers of QVs in *TMLHE* (HI_mis)
Variant status
non−driver
driver
hemizygous
Variant consequence
missense
frameshift
stop gained
multi−hetero

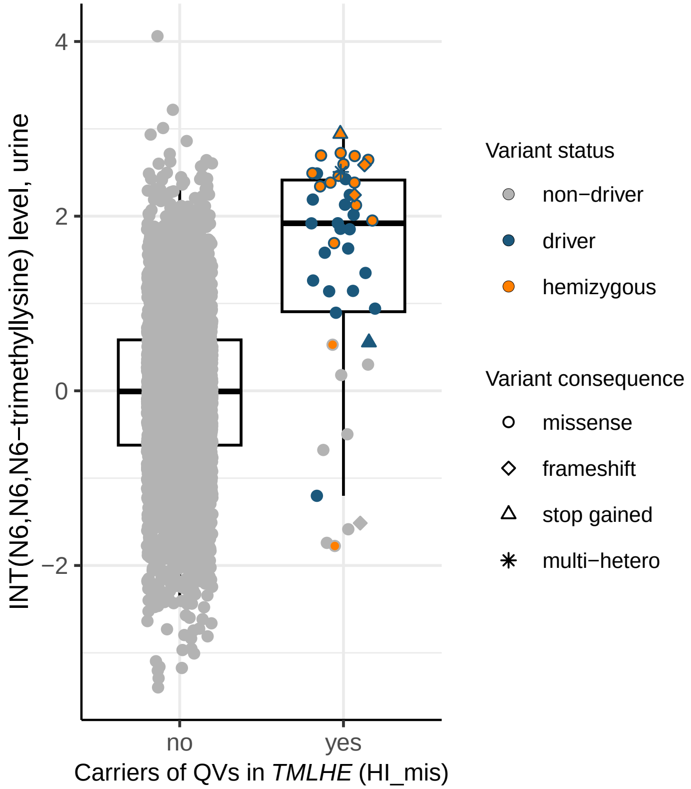
INT(N6,N6,N6−trimethyllysine) level, urine
4
2
0
−2
no
yes
Carriers of QVs in *TMLHE* (HI_mis)
Variant status
non−driver
driver
hemizygous
Variant consequence
missense
frameshift
stop gained
multi−hetero

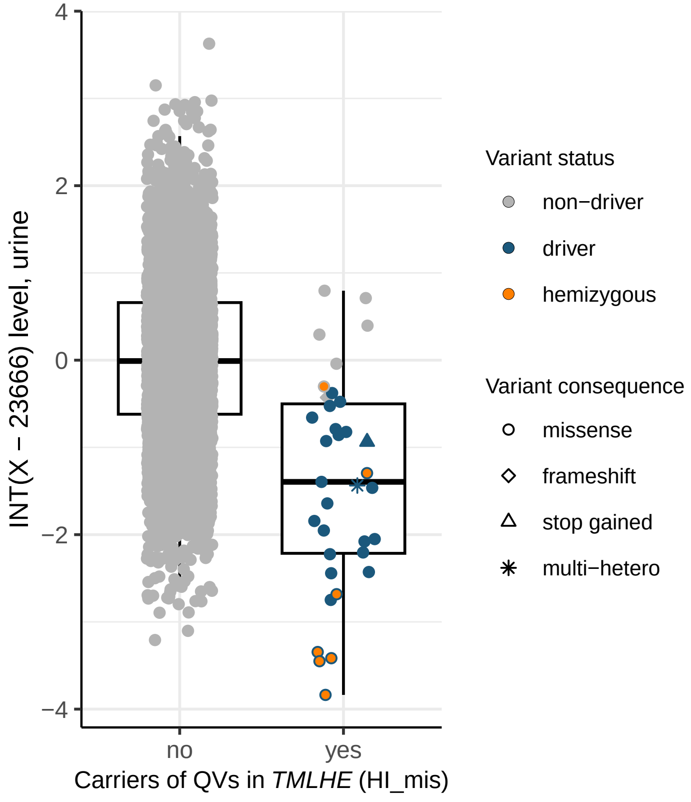
INT(X − 23666) level, urine
4
2
0
−2
−4
no
yes
Carriers of QVs in *TMLHE* (HI_mis)
Variant status
non−driver
driver
hemizygous
Variant consequence
missense
frameshift
stop gained
multi−hetero

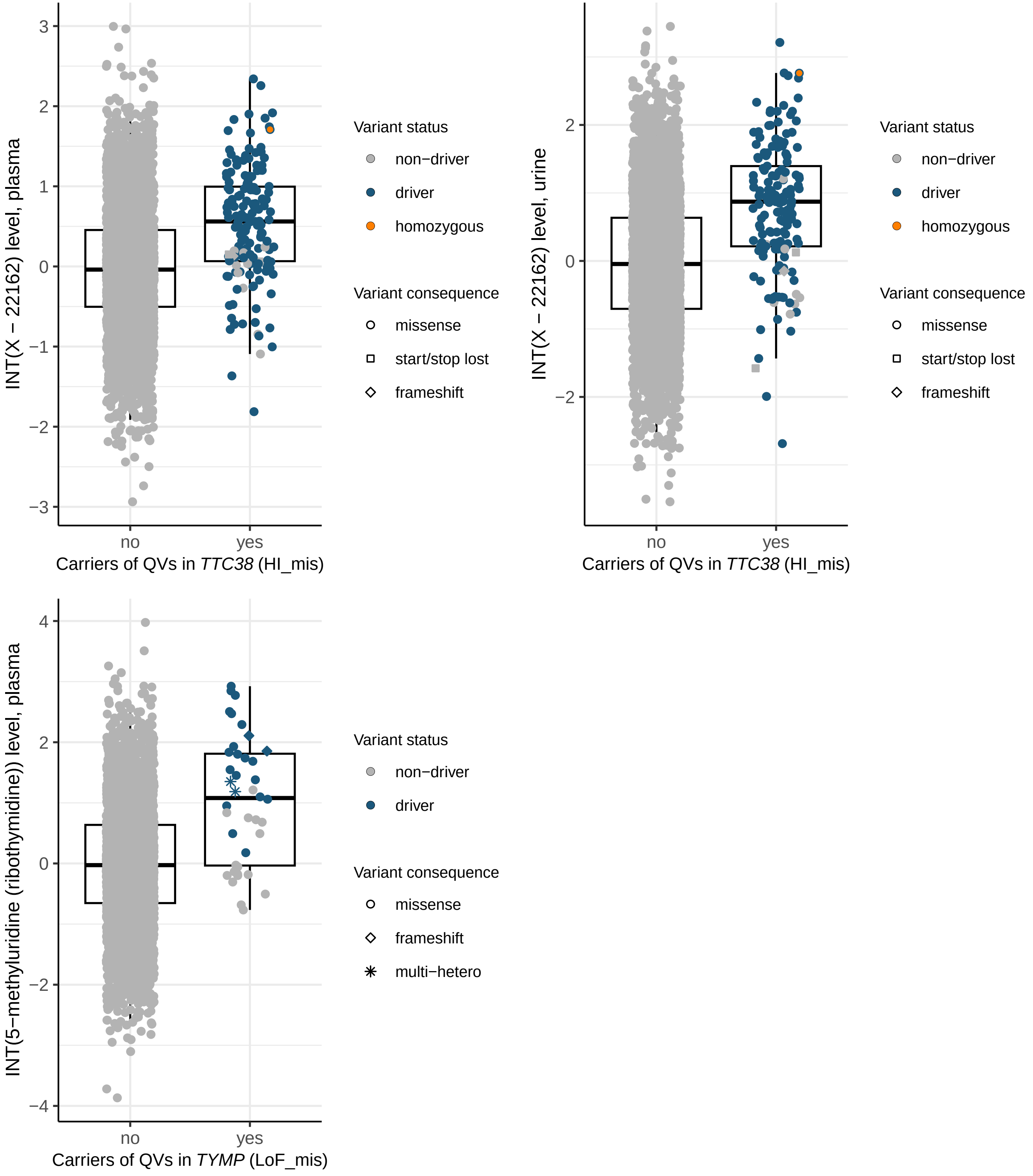

INT(X − 22162) level, plasma
Carriers of QVs in TTC38 (HI_mis)
no
yes
Variant status
non−driver
driver
homozygous
Variant consequence
missense
start/stop lost
frameshift
INT(X − 22162) level, urine
Carriers of QVs in TTC38 (HI_mis)
INT(5−methyluridine (ribothymidine)) level, plasma
Carriers of QVs in TYMP (LoF_mis)
multi−hetero

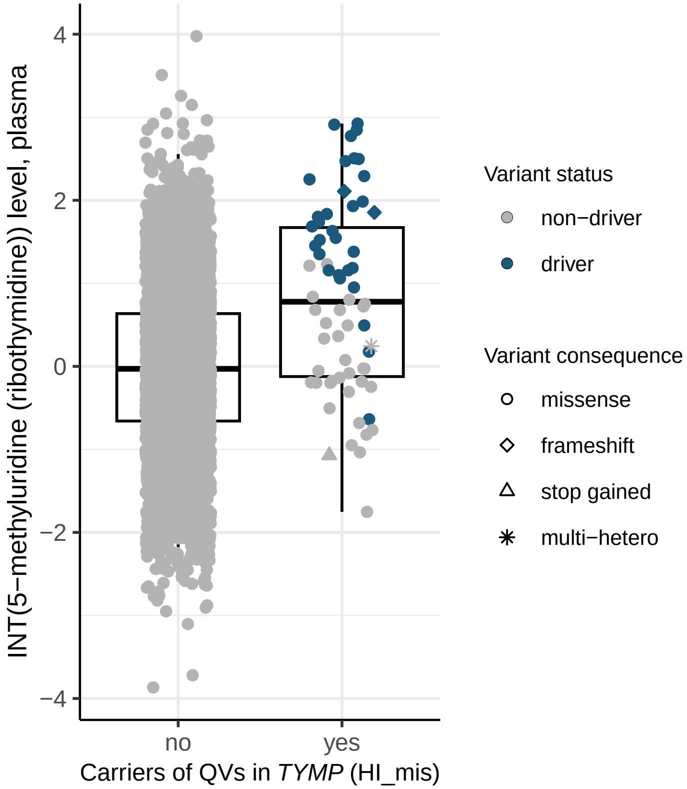
INT(5−methyluridine (ribothymidine)) level, plasma
4
2
0
−2
−4
no
yes
Carriers of QVs in *TYMP* (HI_mis)
Variant status
non−driver
driver
Variant consequence
missense
frameshift
stop gained
multi−hetero

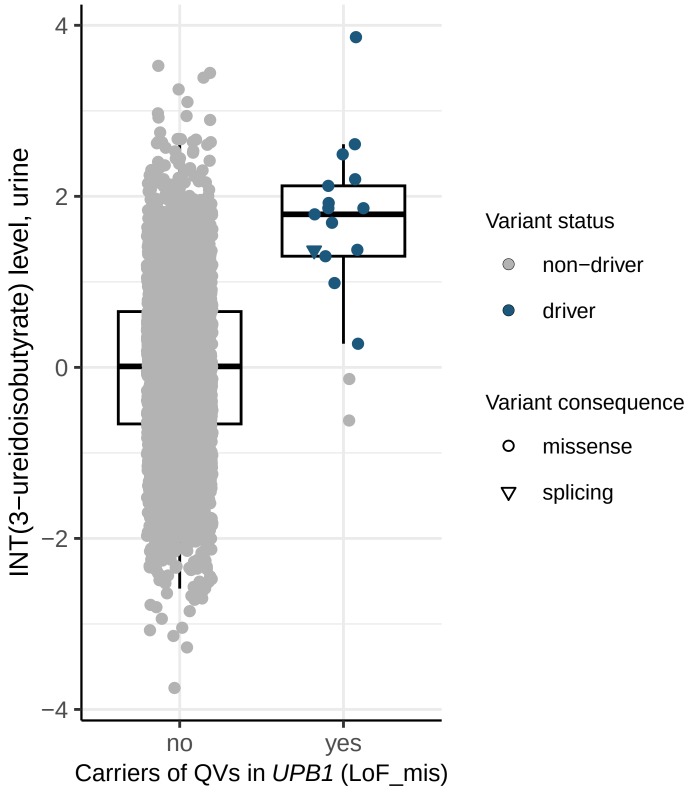
INT(3−ureidoisobutyrate) level, urine
4
2
0
−2
−4
no
yes
Carriers of QVs in *UPB1* (LoF_mis)
Variant status
non−driver
driver
Variant consequence
missense
splicing

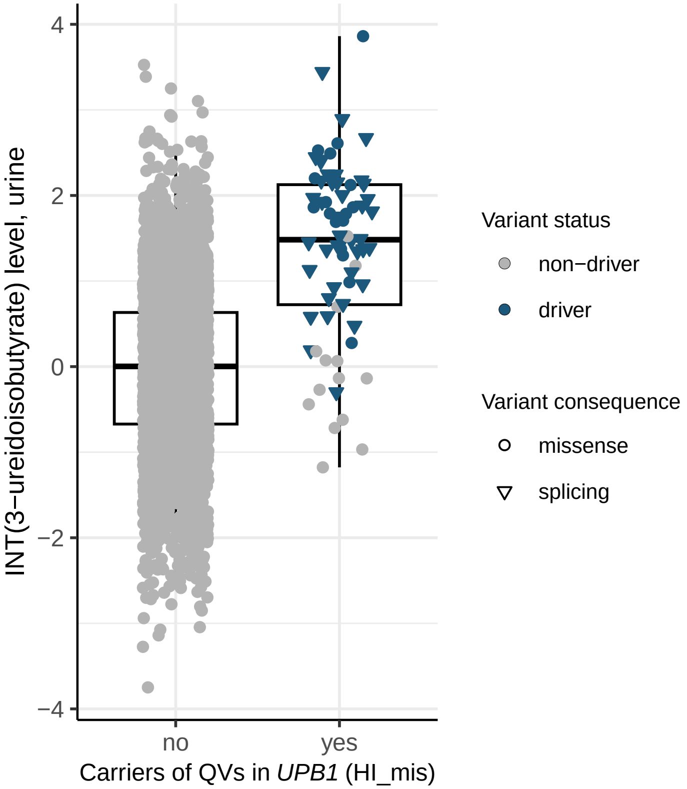

INT(3−ureidoisobutyrate) level, urine
4
2
0
−2
−4
no
yes
Carriers of QVs in *UPB1* (HI_mis)
Variant status
non−driver
driver
Variant consequence
missense
splicing

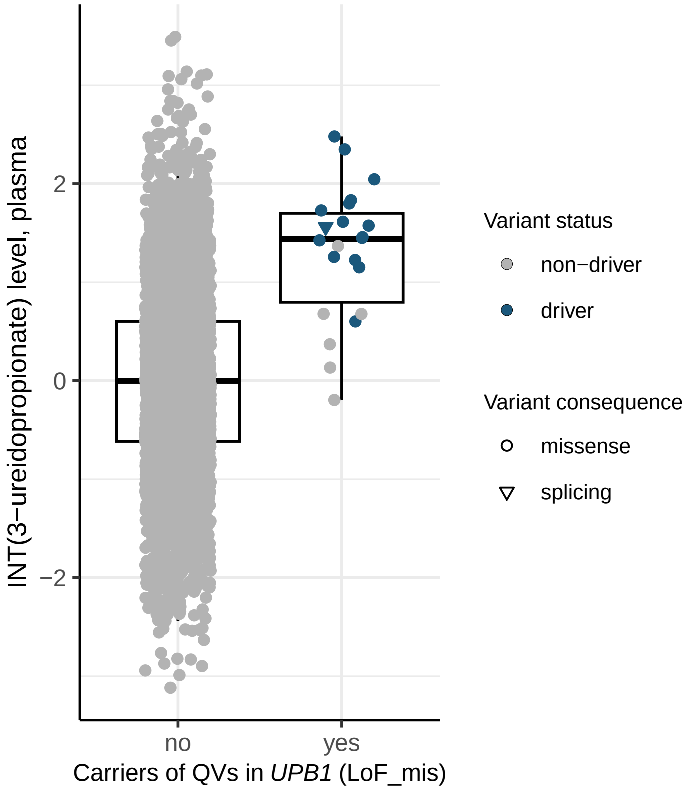

INT(3−ureidopropionate) level, plasma
2
0
−2
no
yes
Carriers of QVs in *UPB1* (LoF_mis)
Variant status
non−driver
driver
Variant consequence
missense
splicing

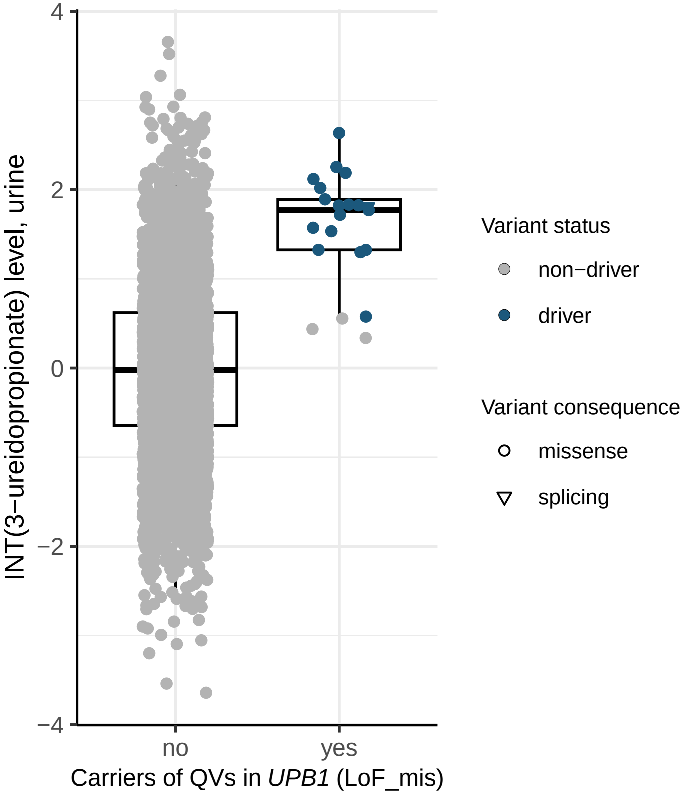

INT(3−ureidopropionate) level, urine
4
2
0
−2
−4
no
yes
Carriers of QVs in *UPB1* (LoF_mis)
Variant status
non−driver
driver
Variant consequence
missense
splicing

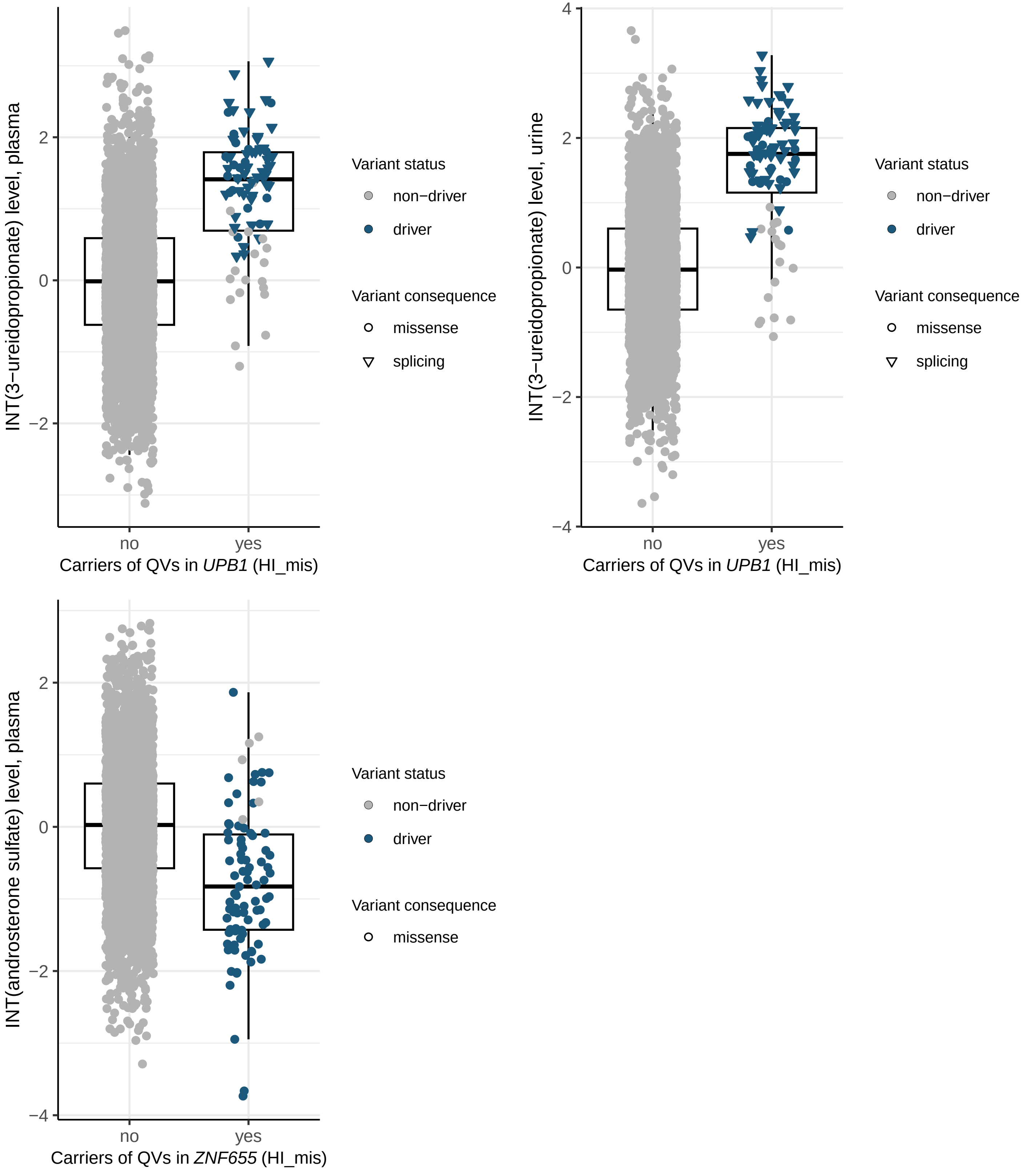

INT(3−ureidopropionate) level, plasma
2
0
−2
no
yes
Carriers of QVs in *UPB1* (HI_mis)
Variant status
non−driver
driver
Variant consequence
missense
splicing
INT(3−ureidopropionate) level, urine
4
2
0
−2
−4
no
yes
Carriers of QVs in *UPB1* (HI_mis)
Variant status
non−driver
driver
Variant consequence
missense
splicing
INT(androsterone sulfate) level, plasma
2
0
−2
−4
no
yes
Carriers of QVs in *ZNF655* (HI_mis)
Variant status
non−driver
driver
Variant consequence
missense

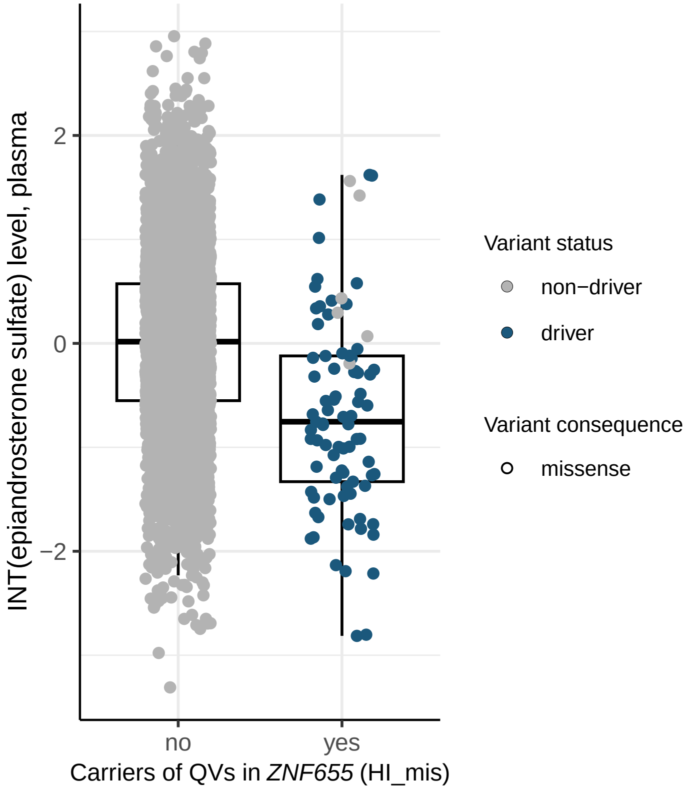
INT(epiandrosterone sulfate) level, plasma
2
0
−2
no
yes
Carriers of QVs in *ZNF655* (HI_mis)
Variant status
non−driver
driver
Variant consequence
missense
