# Supplementary material for: Coupling metabolomics and exome sequencing reveals graded effects of rare damaging heterozygous variants on gene function and human traits
Source: Nat Genet. 2025 Jan 2;57(1):193–205. doi: 10.1038/s41588-024-01965-7 (PMC11735408; doi:10.1038/s41588-024-01965-7)
Supplement: Supplementary file 6 — Contribution of individual QVs to their gene-based association signal with plasma and urine metabolite levels. [file 41588_2024_1965_MOESM6_ESM.pdf]

Supplementary Data 2: Contribution of individual QVs to their gene-based association signal with plasma and urine metabolite levels.

For each significant gene-metabolite pair in plasma and/or in urine (sorted by gene and metabolite's biochemical name), the symbols visualize the $-\log_{10}$(P-value) (y-axis) for the successive aggregation of the most influential QVs with respect to the forward selection procedure (Bomba et al. 2022, PMID: 35568032, Methods) based on burden tests for both masks (LoF_mis on the left, HI_mis on the right). The number of QVs aggregated for burden testing is given on the x-axis. Symbol shape indicates the variant's consequence. The symbol color and size reflect the effect size and the P-value of the variant based on its single-variant association test. The gray dashed lines represent the significance threshold ($-\log_{10}$(5.04e-9) for plasma and $-\log_{10}$(4.46e-9) for urine), the total $-\log_{10}$(P-value) of the aggregate variant test including all QVs in the respective gene and mask, and the $-\log_{10}$(lowest P-value) that can be reached by aggregating only the driver variants from the forward selection procedure. Hence, the variants sorted on the left, which provide the lowest P-value when aggregated, represent the driver variants (for further variant annotation see **Supplementary Tables 7a**,**b**).

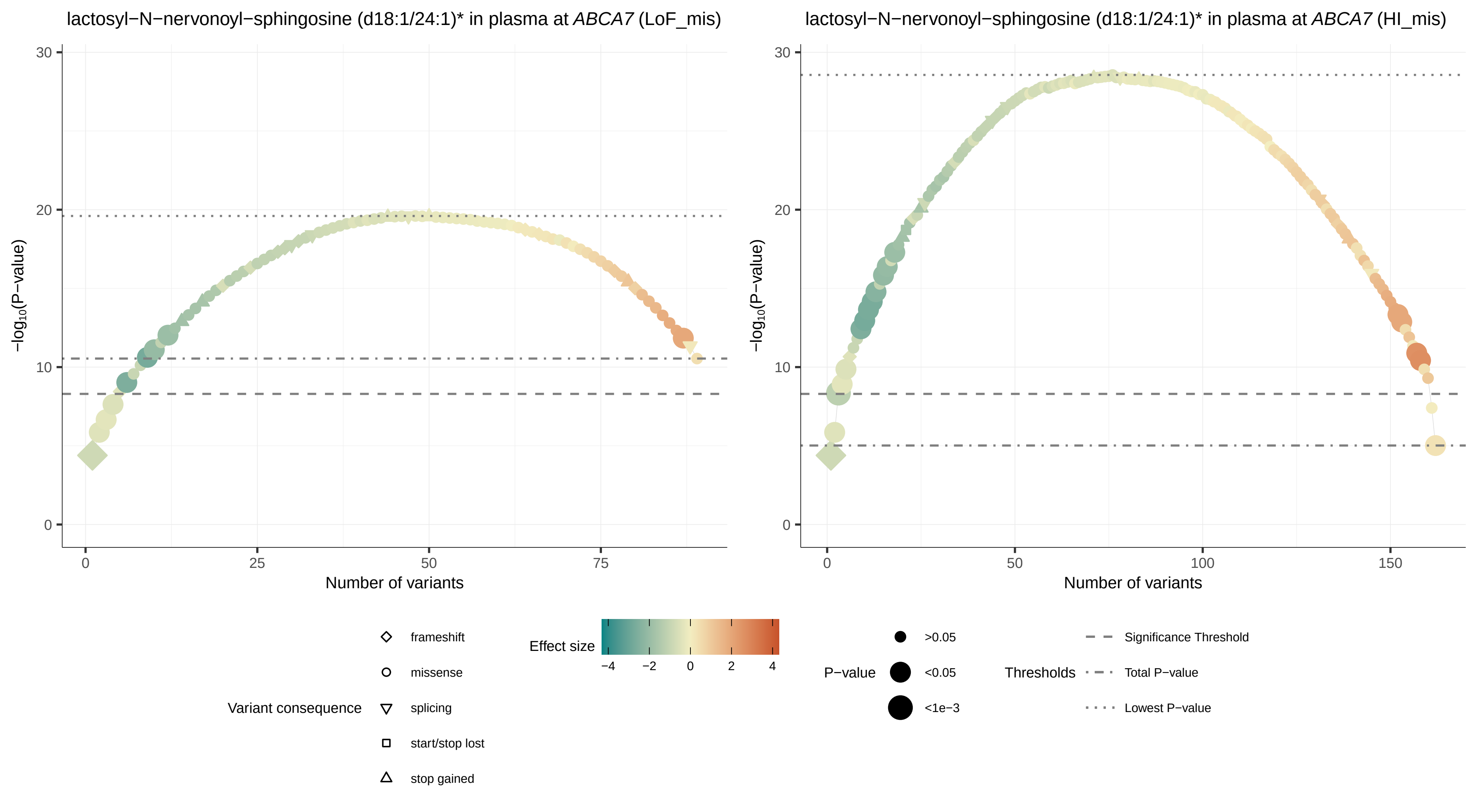

lactosyl−N−nervonoyl−sphingosine (d18:1/24:1)* in plasma at ABCA7 (LoF_mis)
lactosyl−N−nervonoyl−sphingosine (d18:1/24:1)* in plasma at ABCA7 (HI_mis)
−log10(P−value)
Number of variants
0
10
20
30
0
25
50
75
0
50
100
150
Variant consequence
frameshift
missense
splicing
start/stop lost
stop gained
Effect size
−4
−2
0
2
4
P−value
>0.05
<0.05
<1e−3
Thresholds
Significance Threshold
Total P−value
Lowest P−value

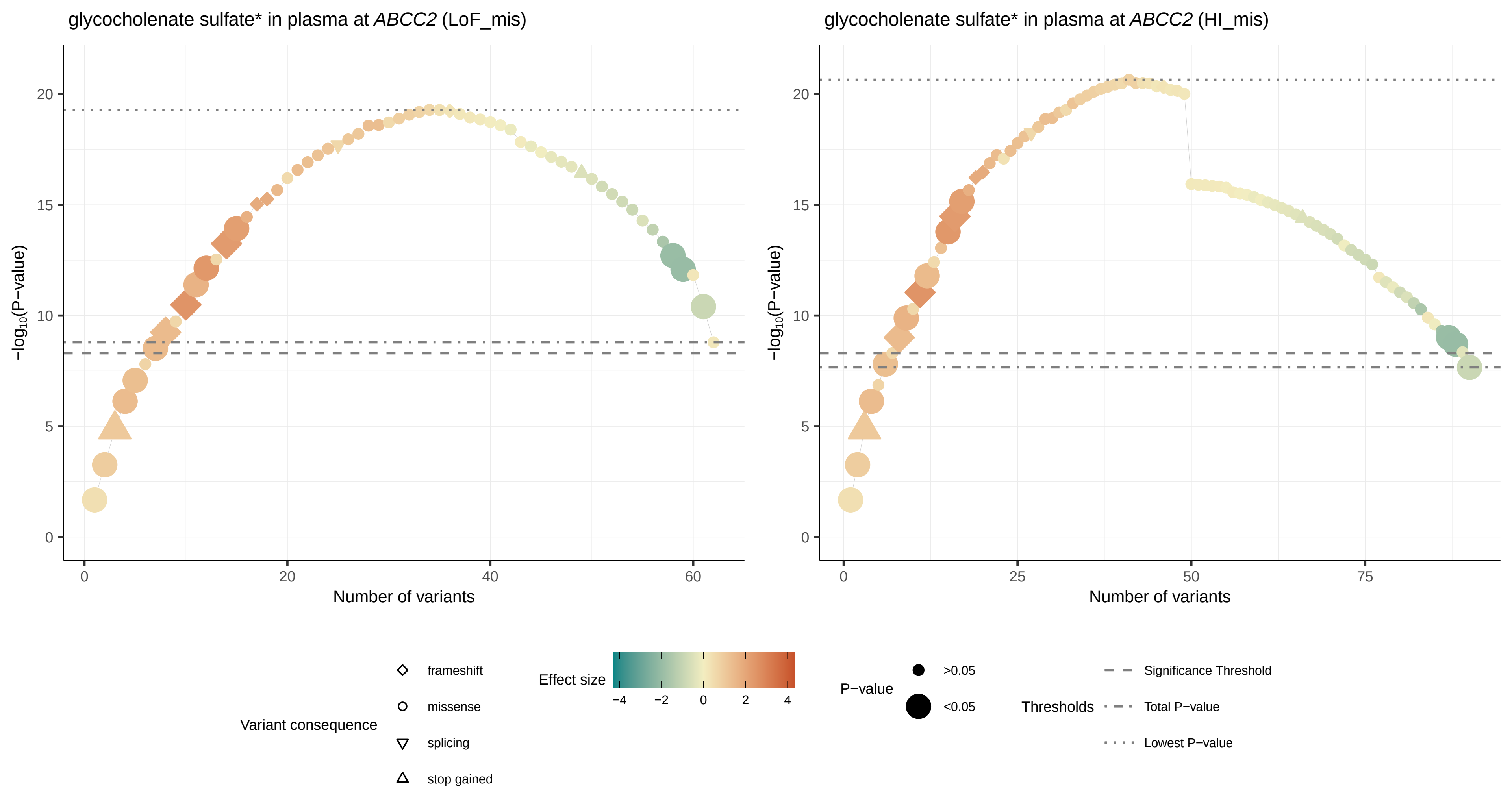

glycocholenate sulfate* in plasma at ABCC2 (LoF_mis)
glycocholenate sulfate* in plasma at ABCC2 (HI_mis)
−log10(P−value)
Number of variants
Variant consequence
frameshift
missense
splicing
stop gained
Effect size
−4
−2
0
2
4
P−value
>0.05
<0.05
Thresholds
Significance Threshold
Total P−value
Lowest P−value

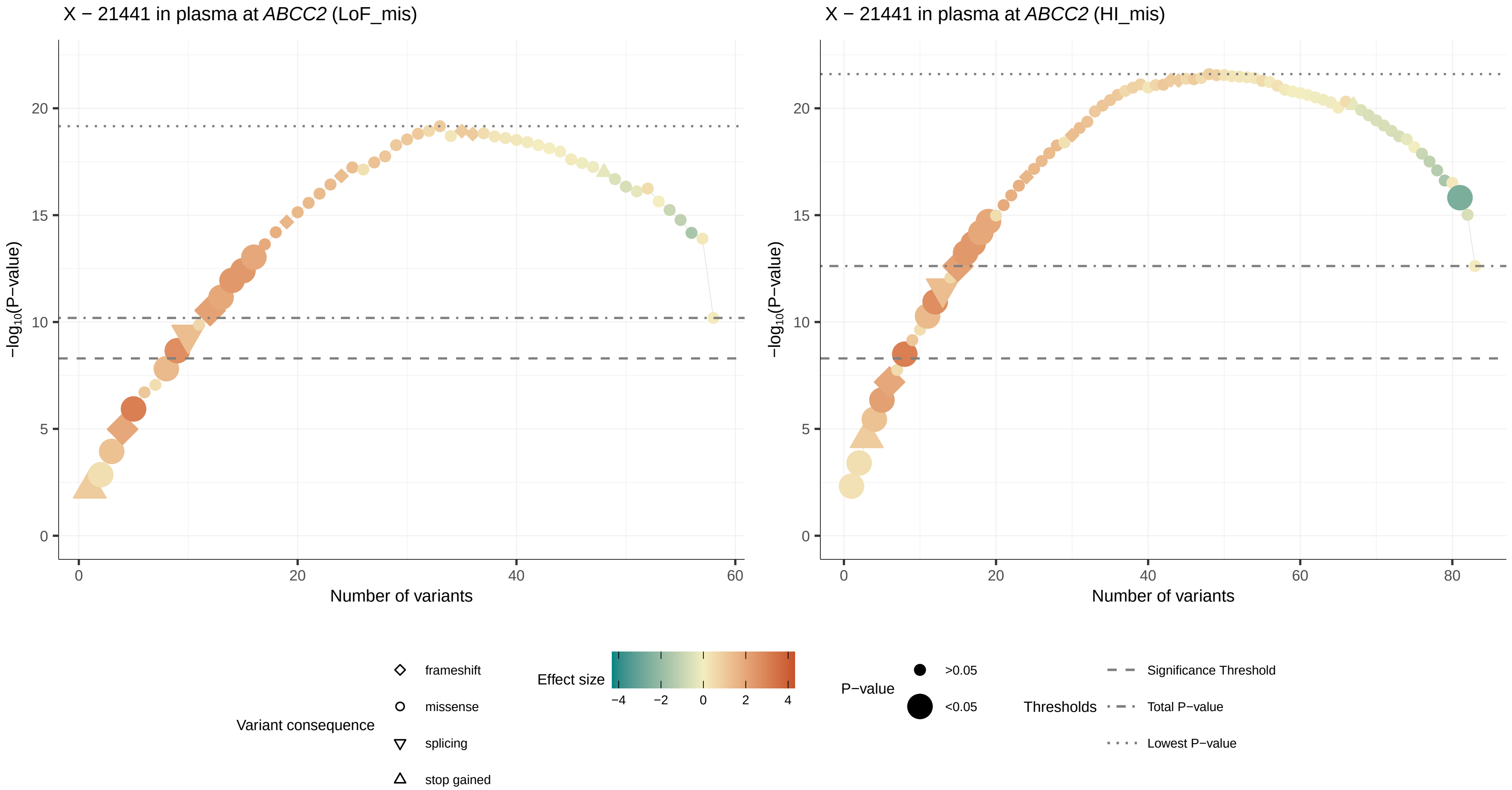
X − 21441 in plasma at ABCC2 (LoF_mis)
X − 21441 in plasma at ABCC2 (HI_mis)
−log10(P−value)
Number of variants
0
5
10
15
20
0
20
40
60
80
Variant consequence
frameshift
missense
splicing
stop gained
Effect size
−4
−2
0
2
4
P−value
>0.05
<0.05
Thresholds
Significance Threshold
Total P−value
Lowest P−value

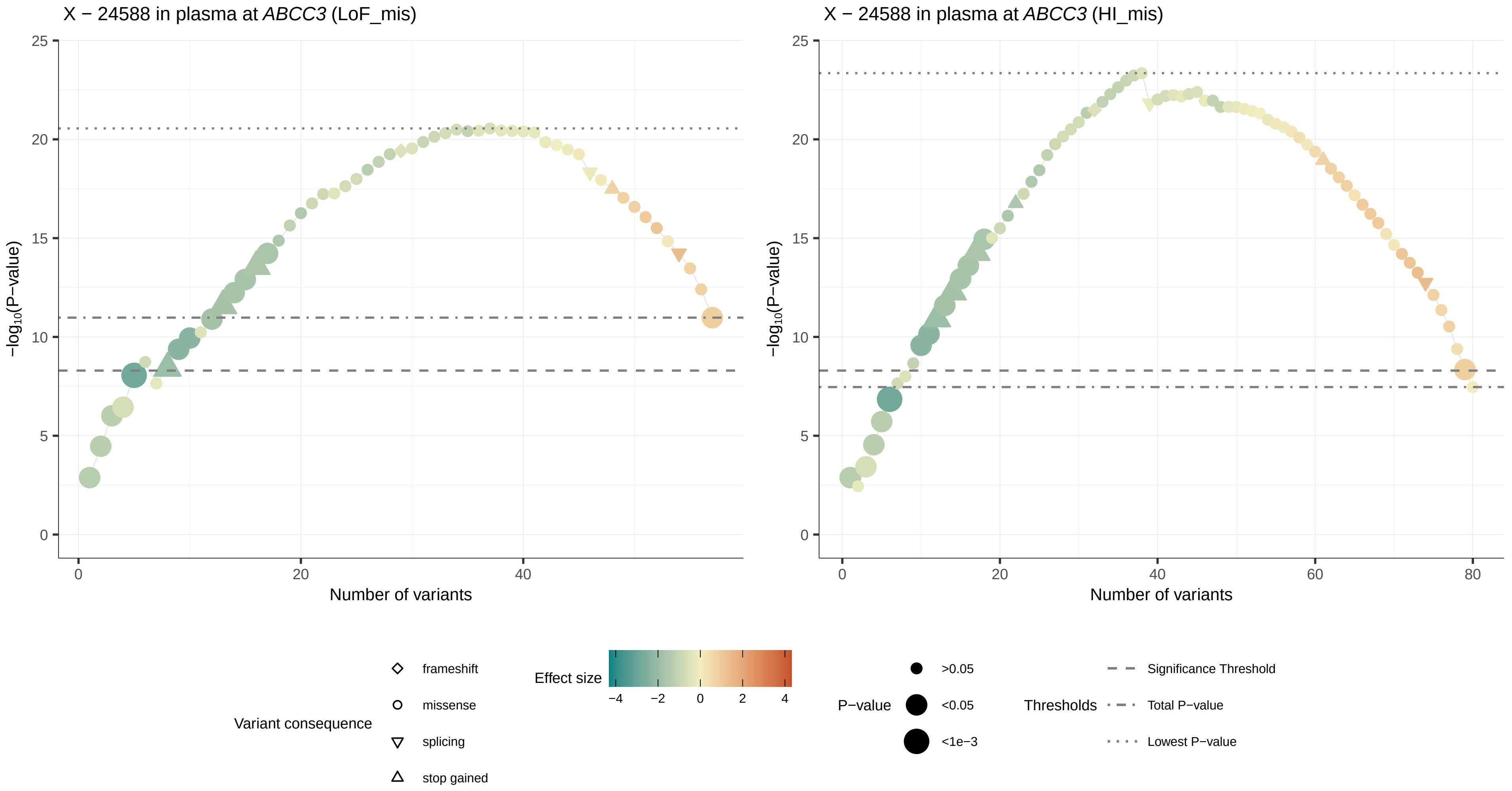

X − 24588 in plasma at *ABCC3* (LoF_mis)
X − 24588 in plasma at *ABCC3* (HI_mis)
−log10(P−value)
Number of variants
0
5
10
15
20
25
0
20
40
60
80
Variant consequence
frameshift
missense
splicing
stop gained
Effect size
−4
−2
0
2
4
P−value
>0.05
<0.05
<1e−3
Thresholds
Significance Threshold
Total P−value
Lowest P−value

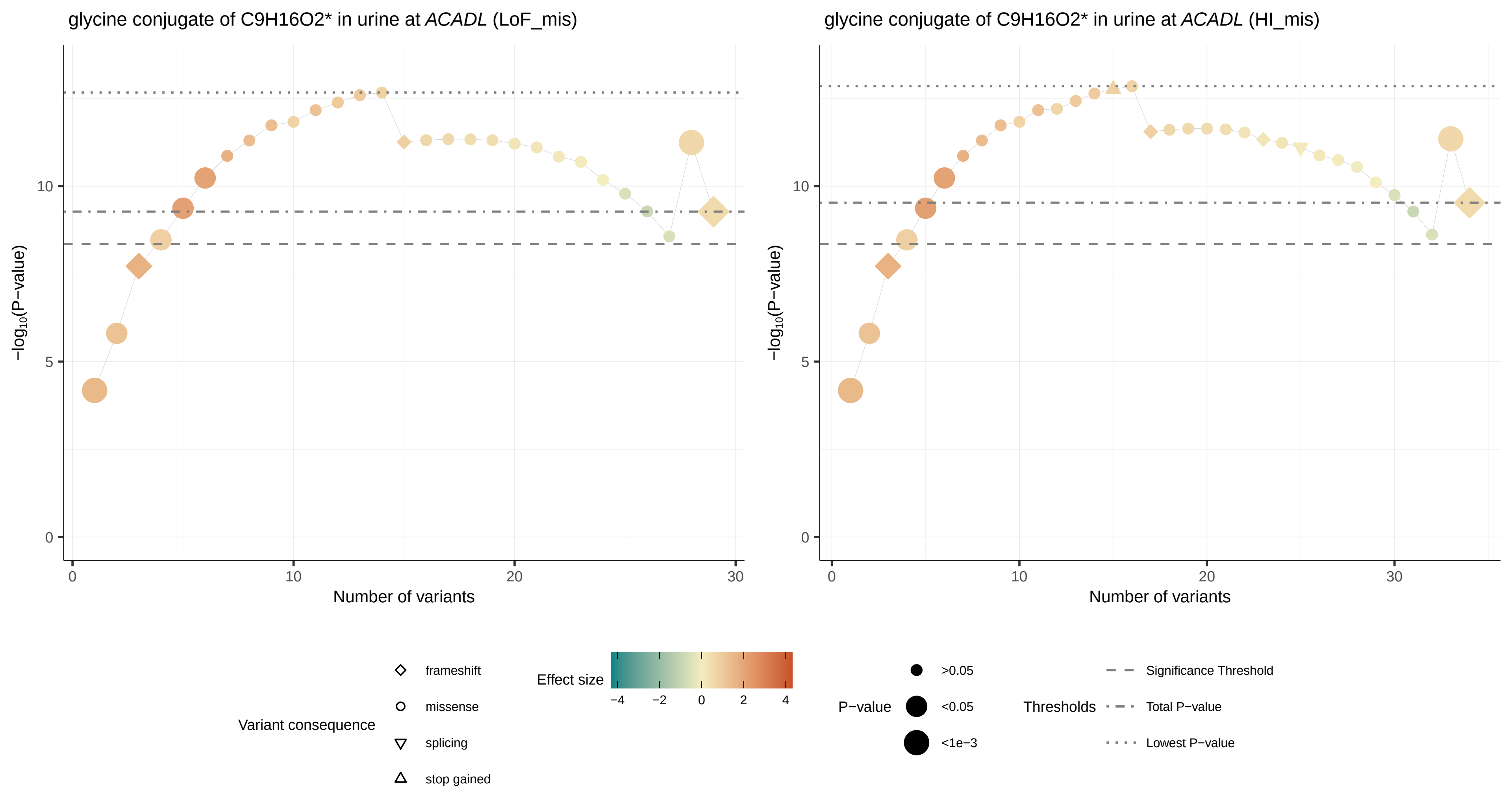

glycine conjugate of C9H16O2* in urine at ACADL (LoF_mis)
glycine conjugate of C9H16O2* in urine at ACADL (HI_mis)
−log10(P−value)
0
5
10
20
30
Number of variants
Variant consequence
frameshift
missense
splicing
stop gained
Effect size
−4
−2
0
2
4
P−value
>0.05
<0.05
<1e−3
Thresholds
Significance Threshold
Total P−value
Lowest P−value

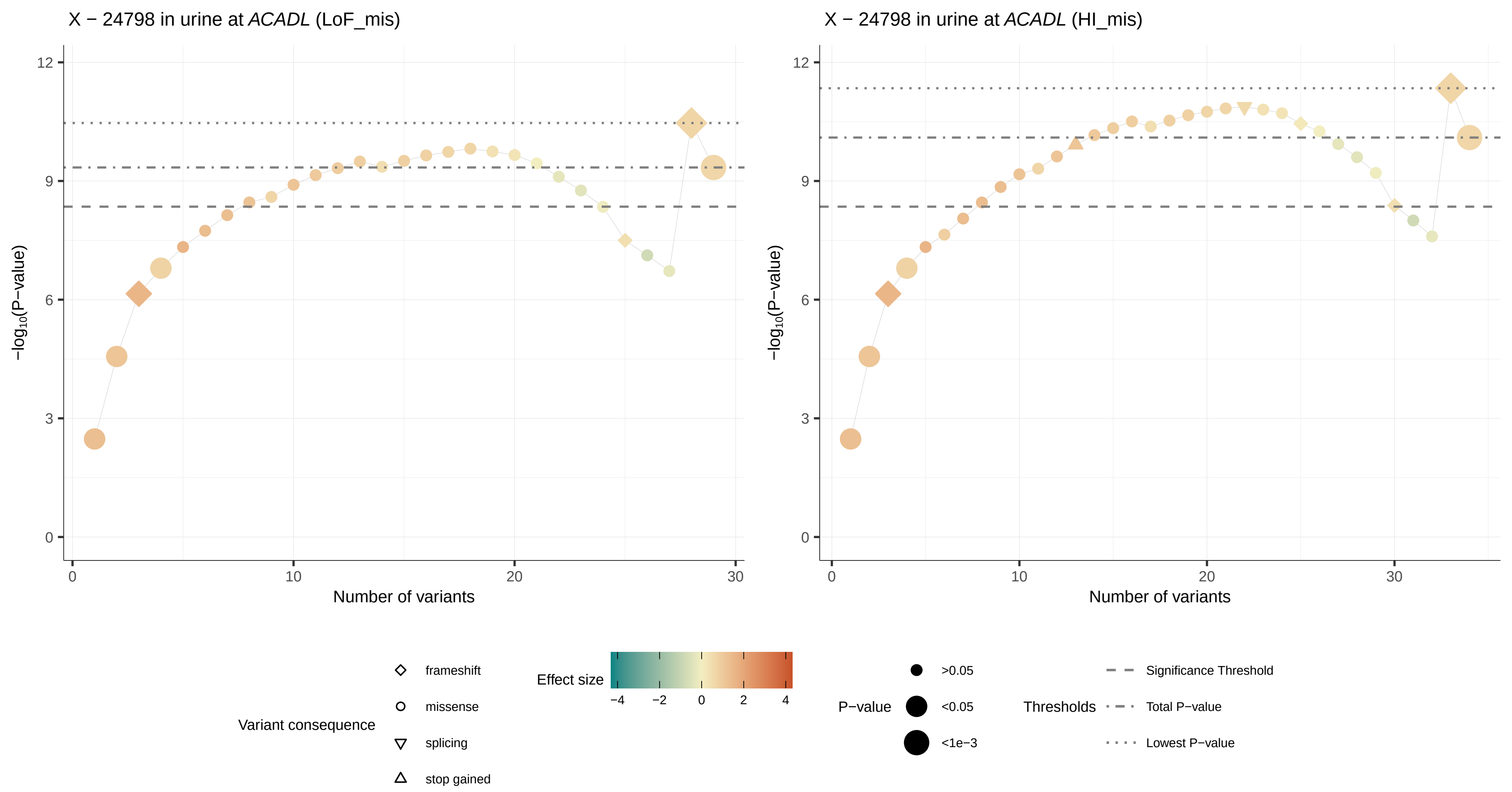

X − 24798 in urine at *ACADL* (LoF_mis)
X − 24798 in urine at *ACADL* (HI_mis)
−log10(P−value)
Number of variants
0
3
6
9
12
0
10
20
30
Variant consequence
frameshift
missense
splicing
stop gained
Effect size
−4
−2
0
2
4
P−value
>0.05
<0.05
<1e−3
Thresholds
Significance Threshold
Total P−value
Lowest P−value

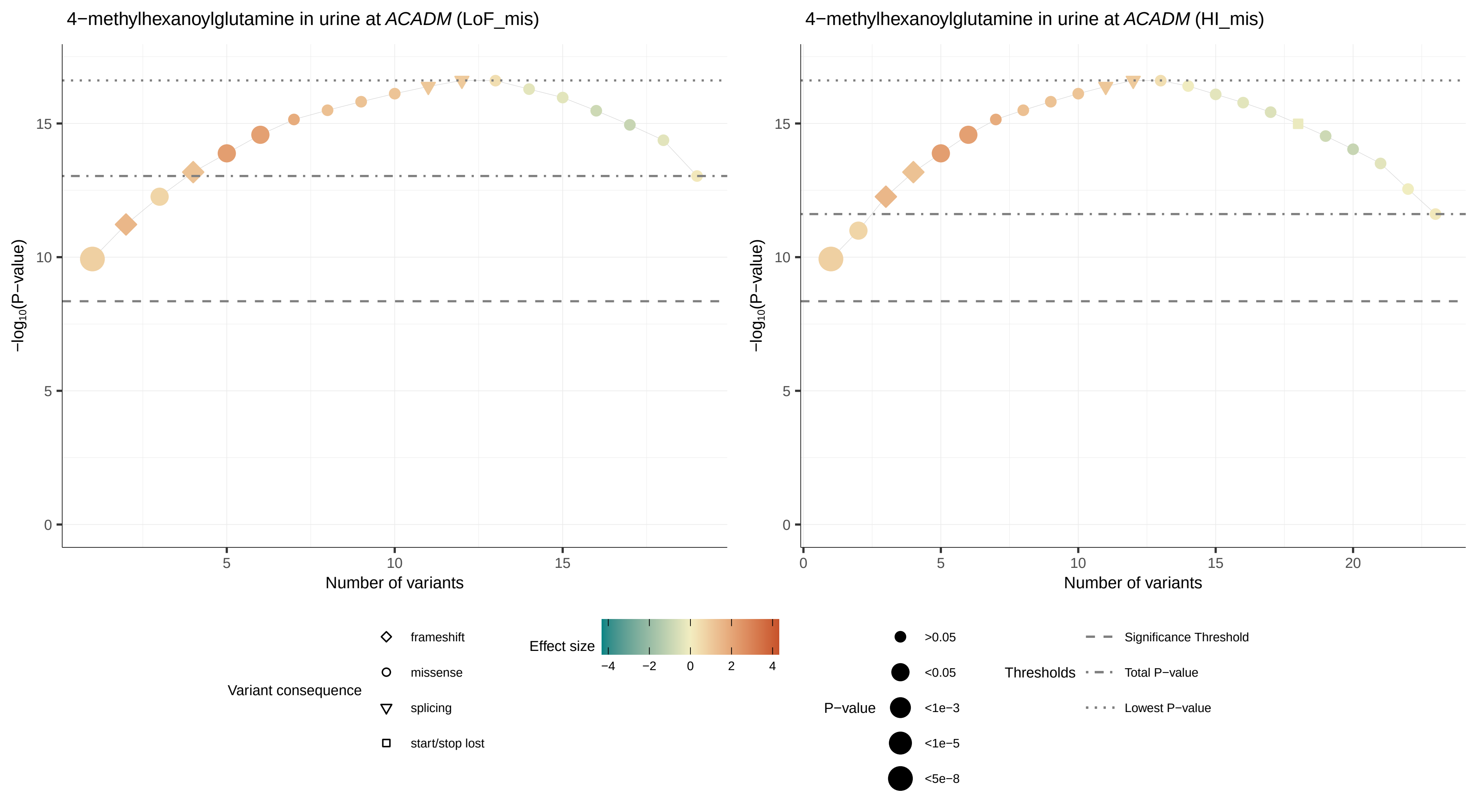
4−methylhexanoylglutamine in urine at ACADM (LoF_mis)
4−methylhexanoylglutamine in urine at ACADM (HI_mis)
−log10(P−value)
Number of variants
Variant consequence
frameshift
missense
splicing
start/stop lost
Effect size
P−value
>0.05
<0.05
<1e−3
<1e−5
<5e−8
Thresholds
Significance Threshold
Total P−value
Lowest P−value

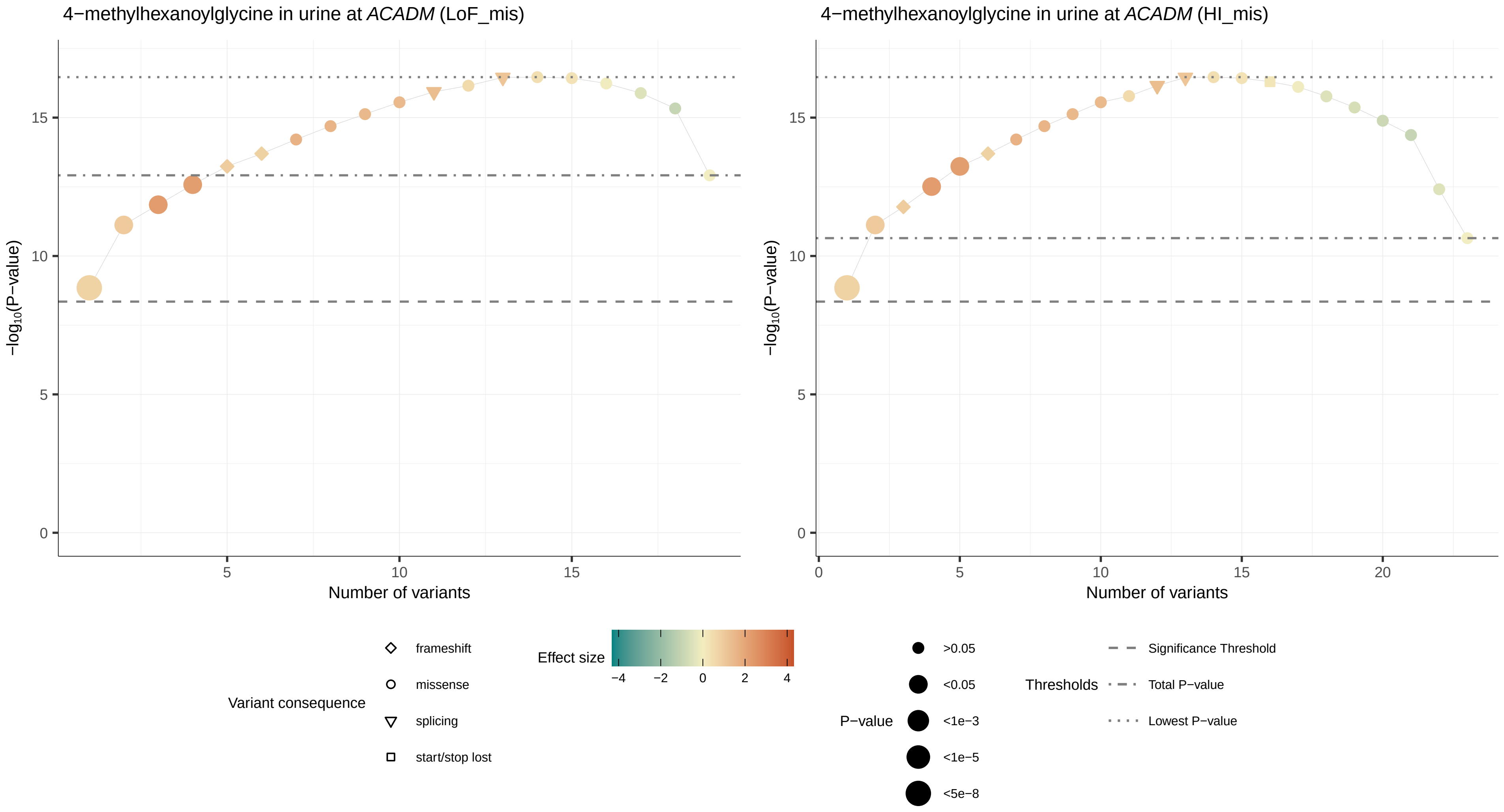
4−methylhexanoylglycine in urine at ACADM (LoF_mis)
4−methylhexanoylglycine in urine at ACADM (HI_mis)
−log10(P−value)
Number of variants
Variant consequence
frameshift
missense
splicing
start/stop lost
Effect size
−4
−2
0
2
4
P−value
>0.05
<0.05
<1e−3
<1e−5
<5e−8
Thresholds
Significance Threshold
Total P−value
Lowest P−value

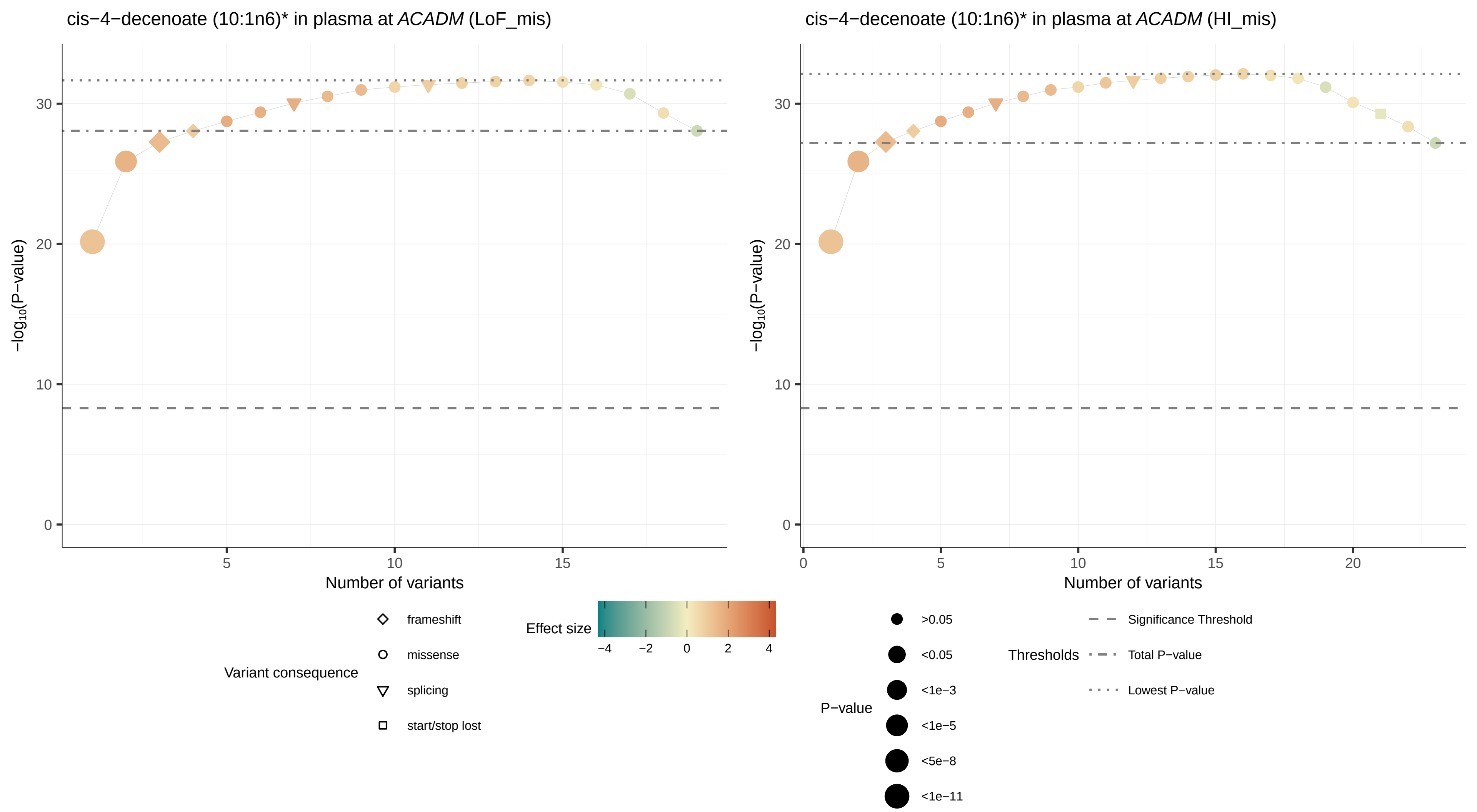

cis−4−decenoate (10:1n6)* in plasma at ACADM (LoF_mis)
cis−4−decenoate (10:1n6)* in plasma at ACADM (HI_mis)
−log10(P−value)
Number of variants
0
10
20
30
5
10
15
20
Variant consequence
frameshift
missense
splicing
start/stop lost
Effect size
−4
−2
0
2
4
P−value
>0.05
<0.05
<1e−3
<1e−5
<5e−8
<1e−11
Thresholds
Significance Threshold
Total P−value
Lowest P−value

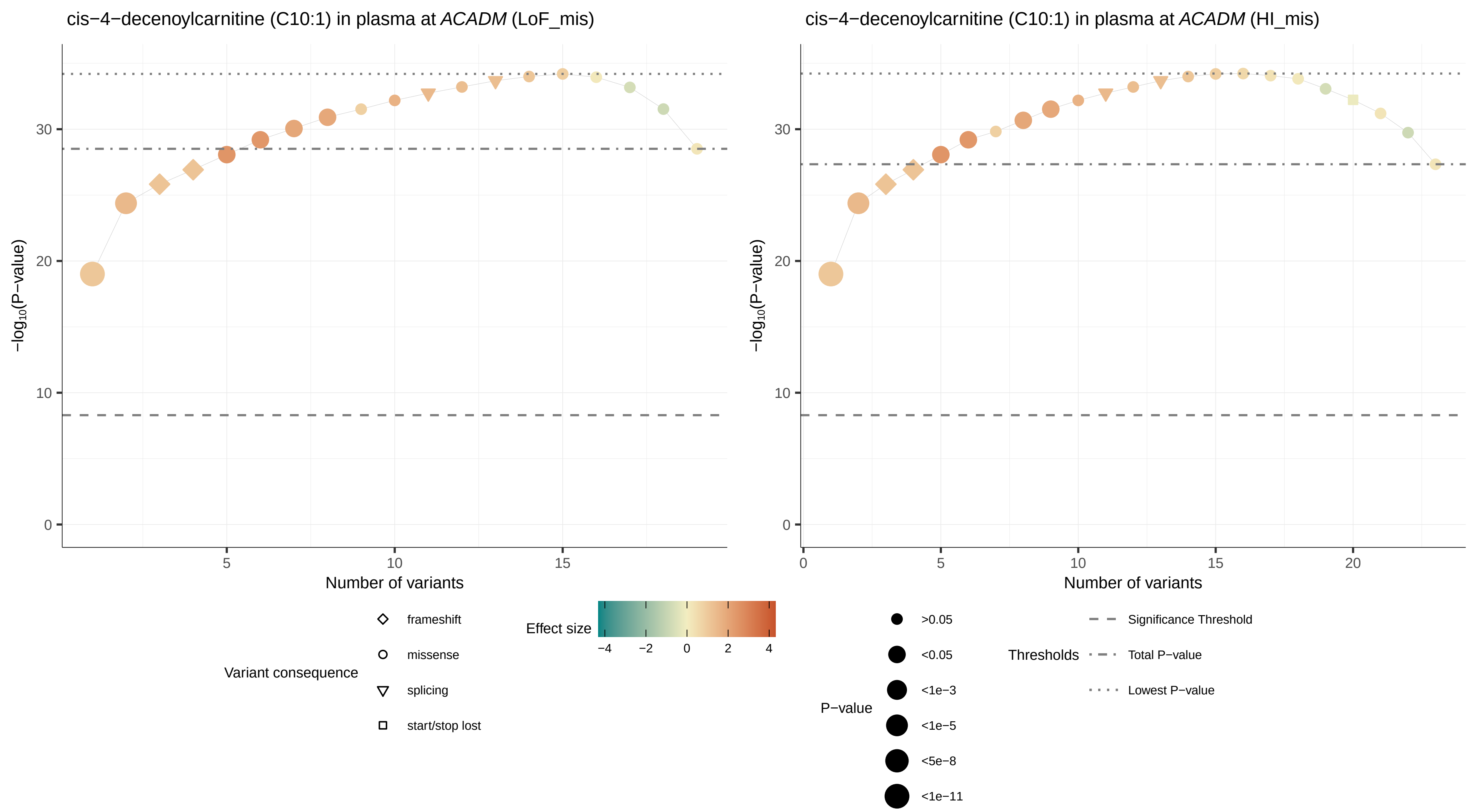

cis−4−decenoylcarnitine (C10:1) in plasma at ACADM (LoF_mis)
cis−4−decenoylcarnitine (C10:1) in plasma at ACADM (HI_mis)
−log10(P−value)
Number of variants
0
10
20
30
5
15
Variant consequence
frameshift
missense
splicing
start/stop lost
Effect size
−4
−2
2
4
P−value
>0.05
<0.05
<1e−3
<1e−5
<5e−8
<1e−11
Thresholds
Significance Threshold
Total P−value
Lowest P−value

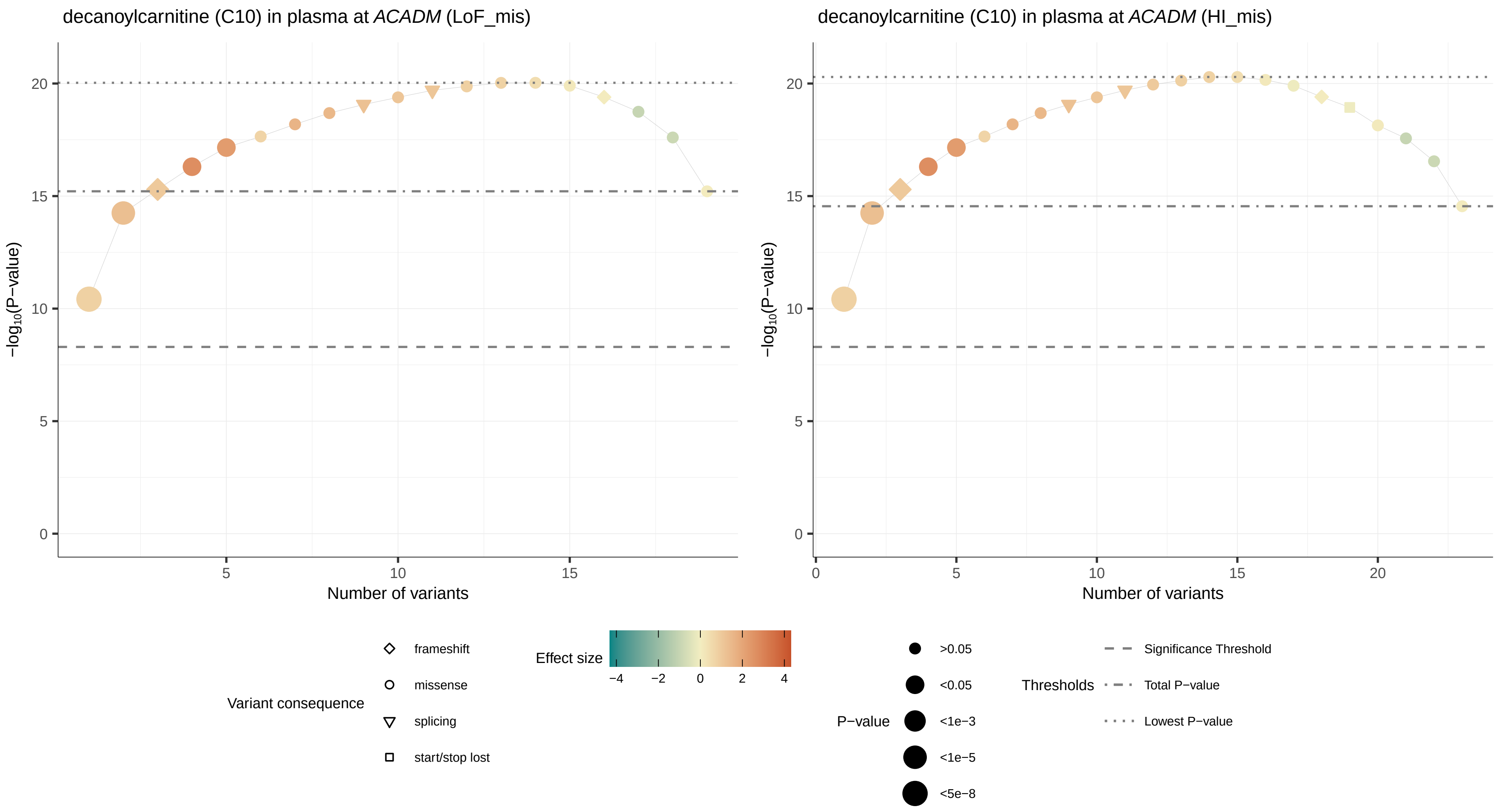
decanoylcarnitine (C10) in plasma at ACADM (LoF_mis)
decanoylcarnitine (C10) in plasma at ACADM (HI_mis)
−log10(P−value)
Number of variants
Variant consequence
frameshift
missense
splicing
start/stop lost
Effect size
P−value
>0.05
<0.05
<1e−3
<1e−5
<5e−8
Thresholds
Significance Threshold
Total P−value
Lowest P−value

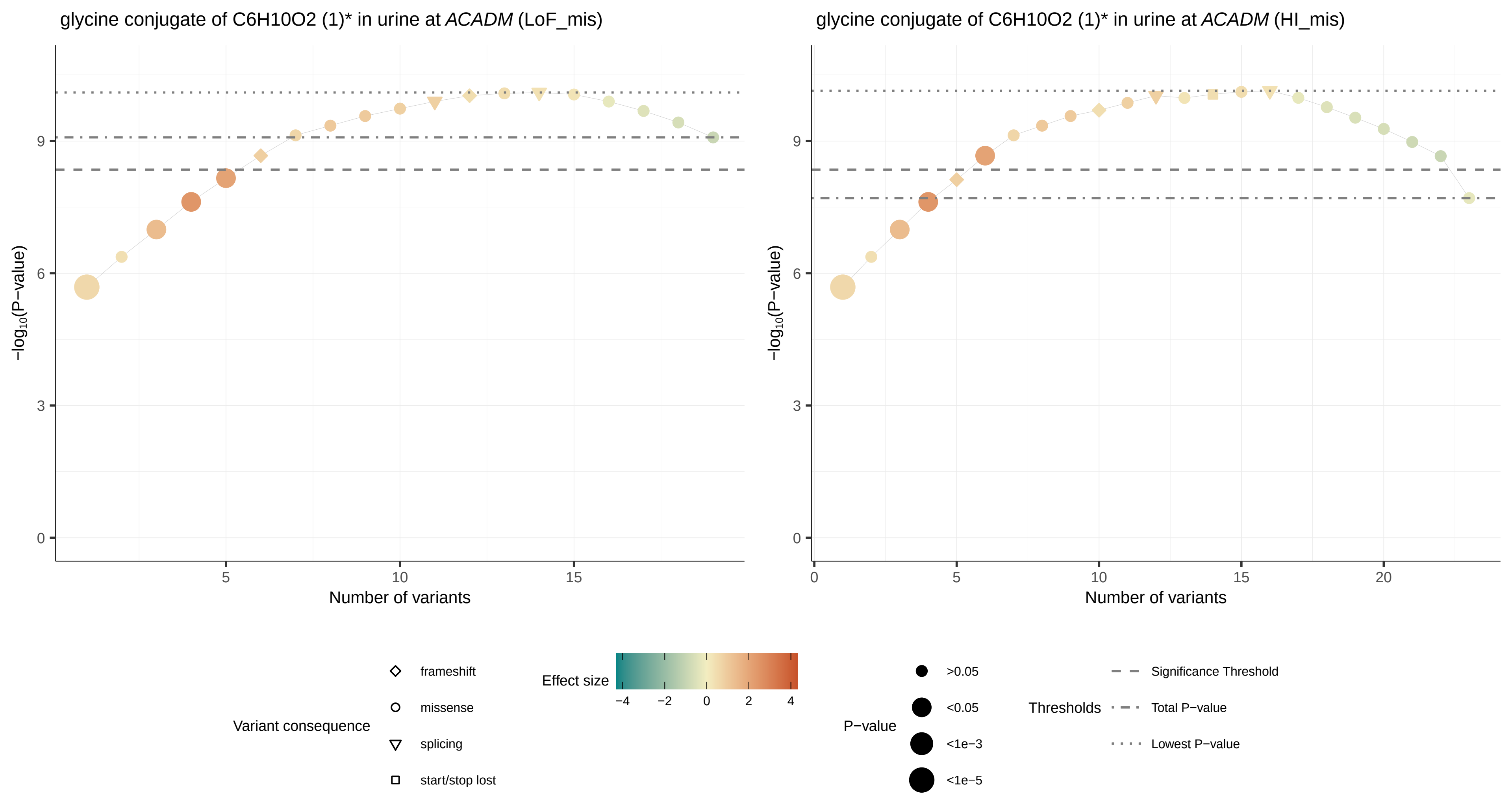

glycine conjugate of C6H10O2 (1)* in urine at ACADM (LoF_mis)
glycine conjugate of C6H10O2 (1)* in urine at ACADM (HI_mis)
−log10(P−value)
Number of variants
0
3
6
9
5
10
15
20
Variant consequence
frameshift
missense
splicing
start/stop lost
Effect size
−4
−2
0
2
4
P−value
>0.05
<0.05
<1e−3
<1e−5
Thresholds
Significance Threshold
Total P−value
Lowest P−value

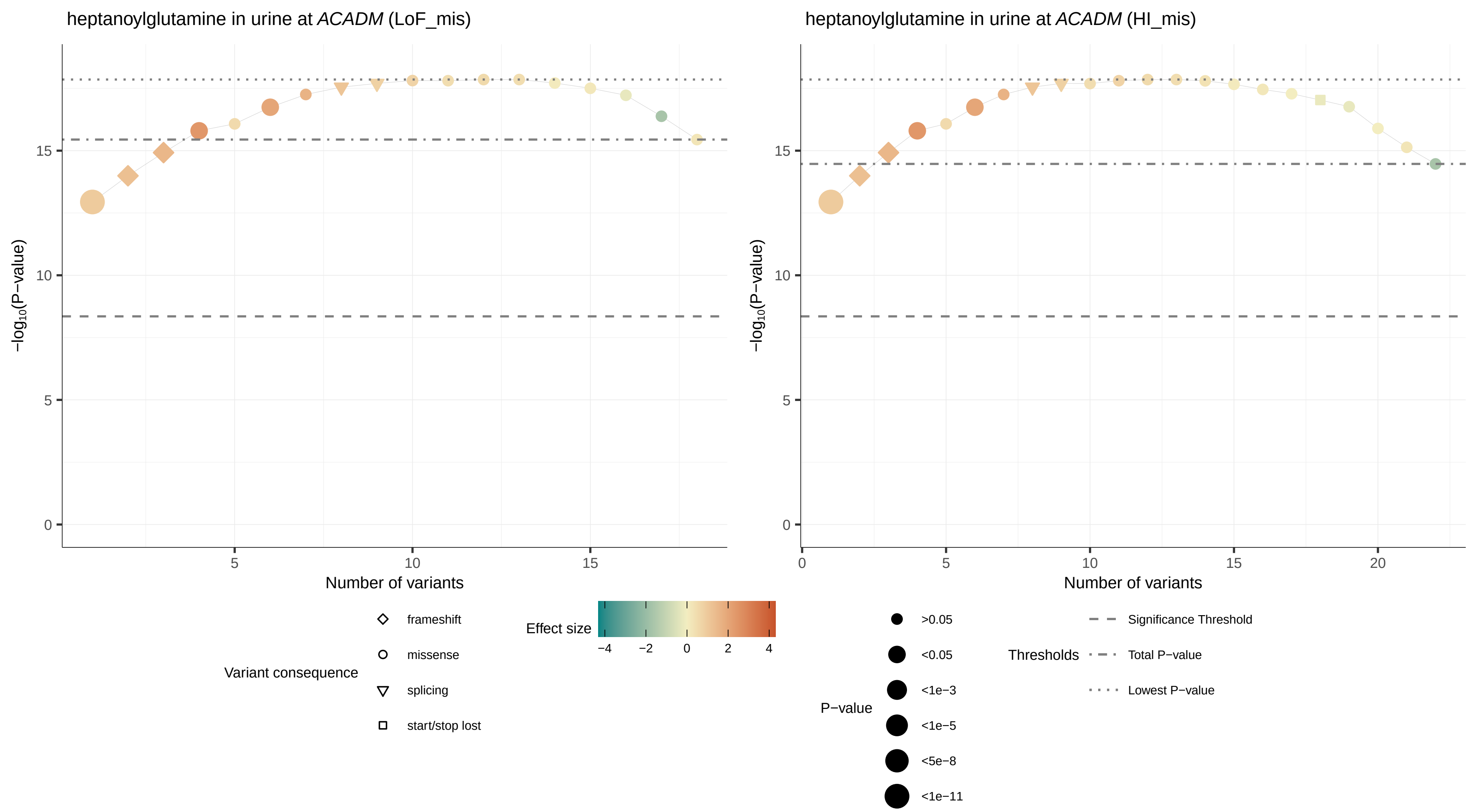

heptanoylglutamine in urine at ACADM (LoF_mis)
heptanoylglutamine in urine at ACADM (HI_mis)
−log10(P−value)
Number of variants
Variant consequence
frameshift
missense
splicing
start/stop lost
Effect size
P−value
>0.05
<0.05
<1e−3
<1e−5
<5e−8
<1e−11
Thresholds
Significance Threshold
Total P−value
Lowest P−value

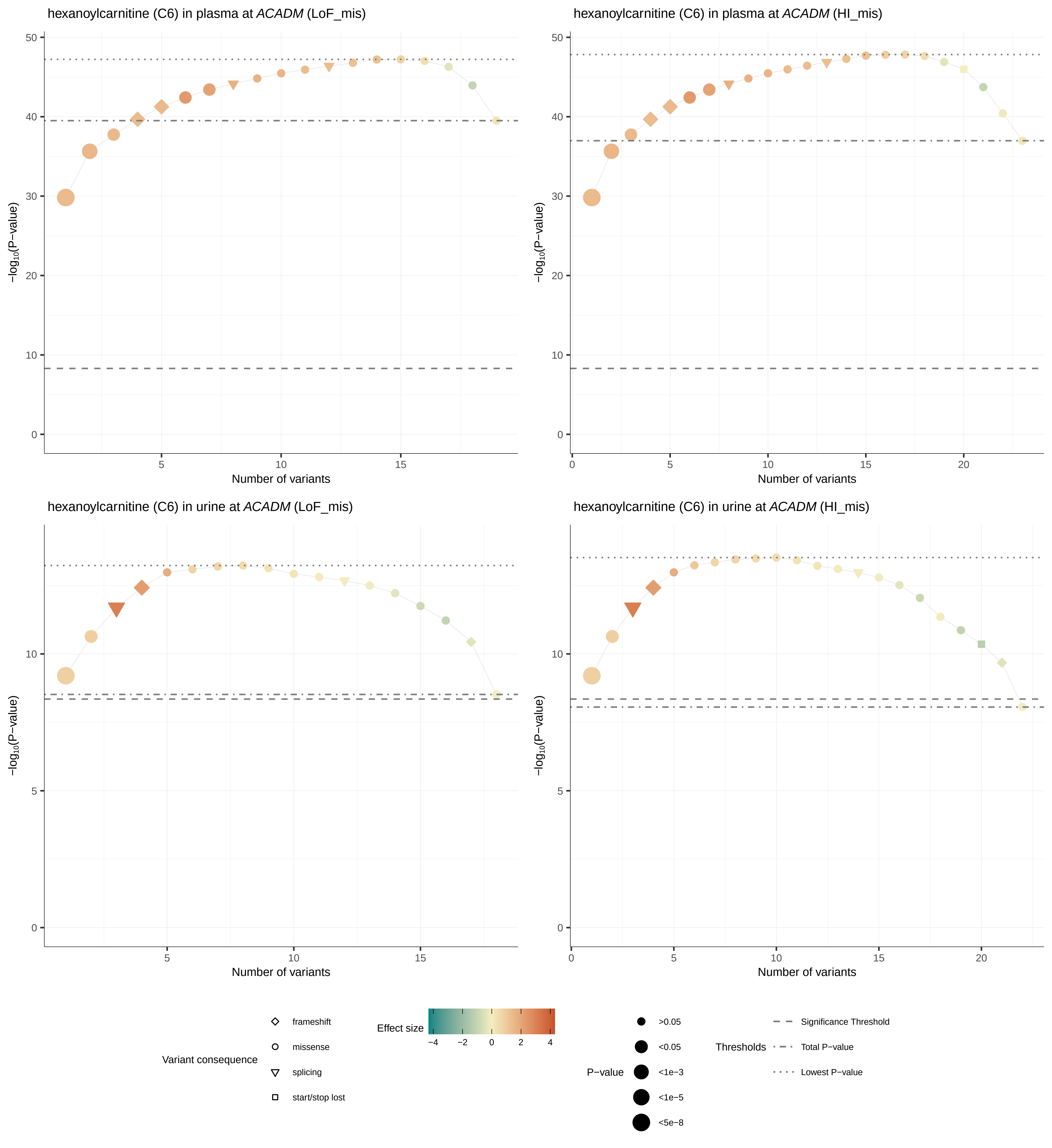
hexanoylcarnitine (C6) in plasma at ACADM (LoF_mis)
hexanoylcarnitine (C6) in plasma at ACADM (HI_mis)
hexanoylcarnitine (C6) in urine at ACADM (LoF_mis)
hexanoylcarnitine (C6) in urine at ACADM (HI_mis)
−log10(P−value)
Number of variants
Variant consequence
frameshift
missense
splicing
start/stop lost
Effect size
−4
−2
0
2
4
P−value
>0.05
<0.05
<1e−3
<1e−5
<5e−8
Thresholds
Significance Threshold
Total P−value
Lowest P−value

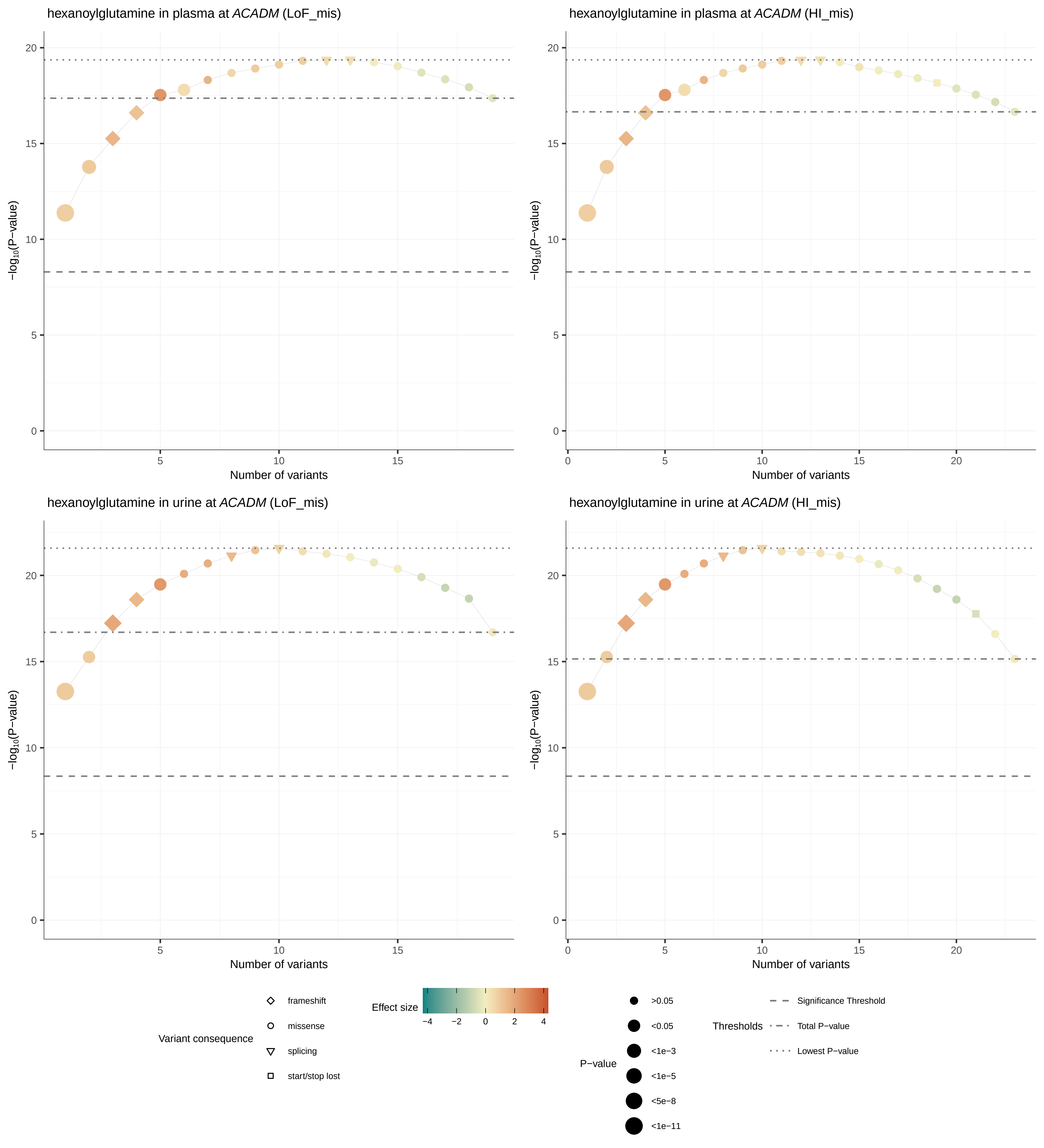

hexanoylglutamine in plasma at ACADM (LoF_mis)
hexanoylglutamine in plasma at ACADM (HI_mis)
hexanoylglutamine in urine at ACADM (LoF_mis)
hexanoylglutamine in urine at ACADM (HI_mis)
−log10(P−value)
Number of variants
Variant consequence
frameshift
missense
splicing
start/stop lost
Effect size
−4
−2
0
2
4
P−value
>0.05
<0.05
<1e−3
<1e−5
<5e−8
<1e−11
Thresholds
Significance Threshold
Total P−value
Lowest P−value

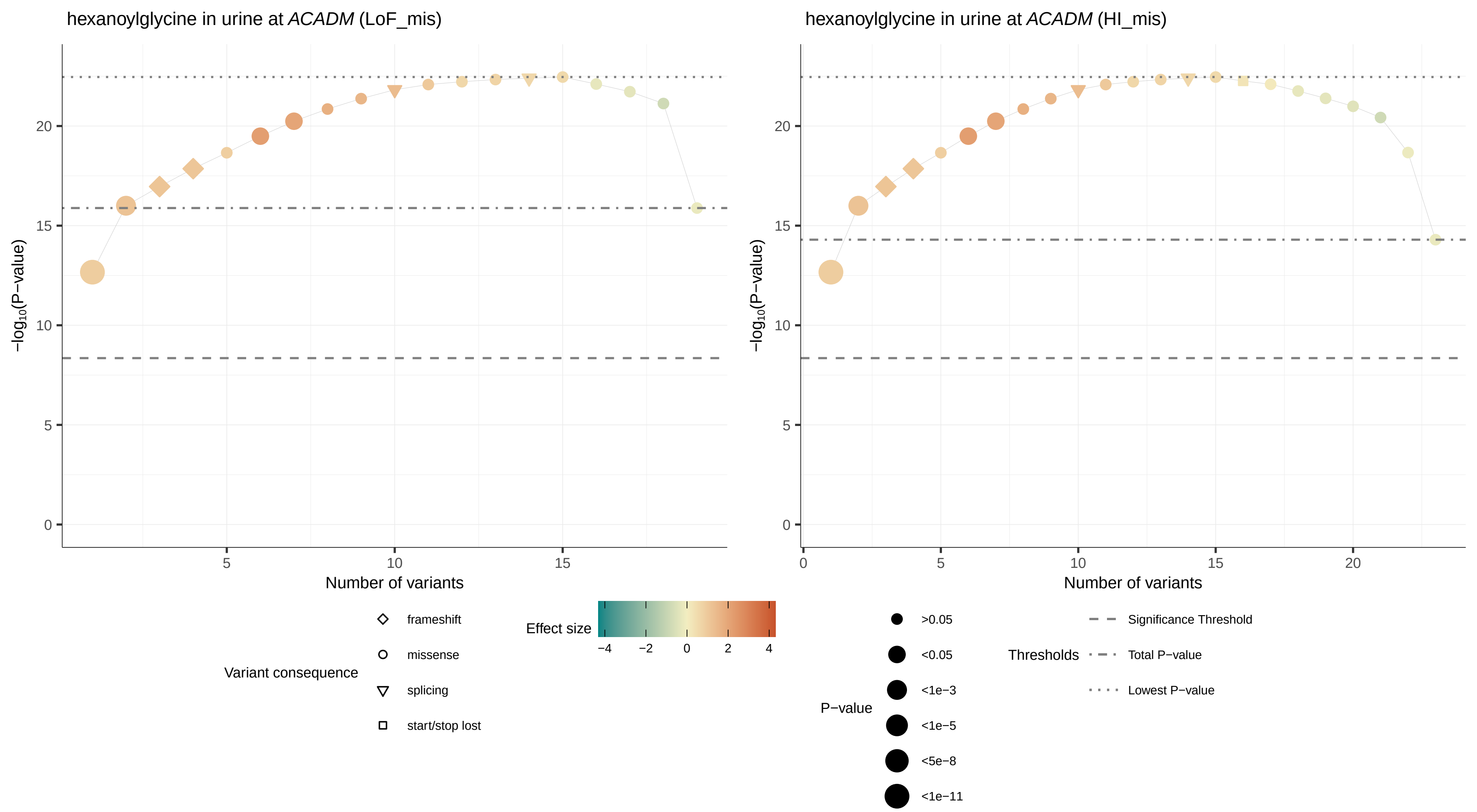

hexanoylglycine in urine at ACADM (LoF_mis)
hexanoylglycine in urine at ACADM (HI_mis)
−log10(P−value)
Number of variants
0
5
10
15
20
Variant consequence
frameshift
missense
splicing
start/stop lost
Effect size
−4
−2
0
2
4
P−value
>0.05
<0.05
<1e−3
<1e−5
<5e−8
<1e−11
Thresholds
Significance Threshold
Total P−value
Lowest P−value

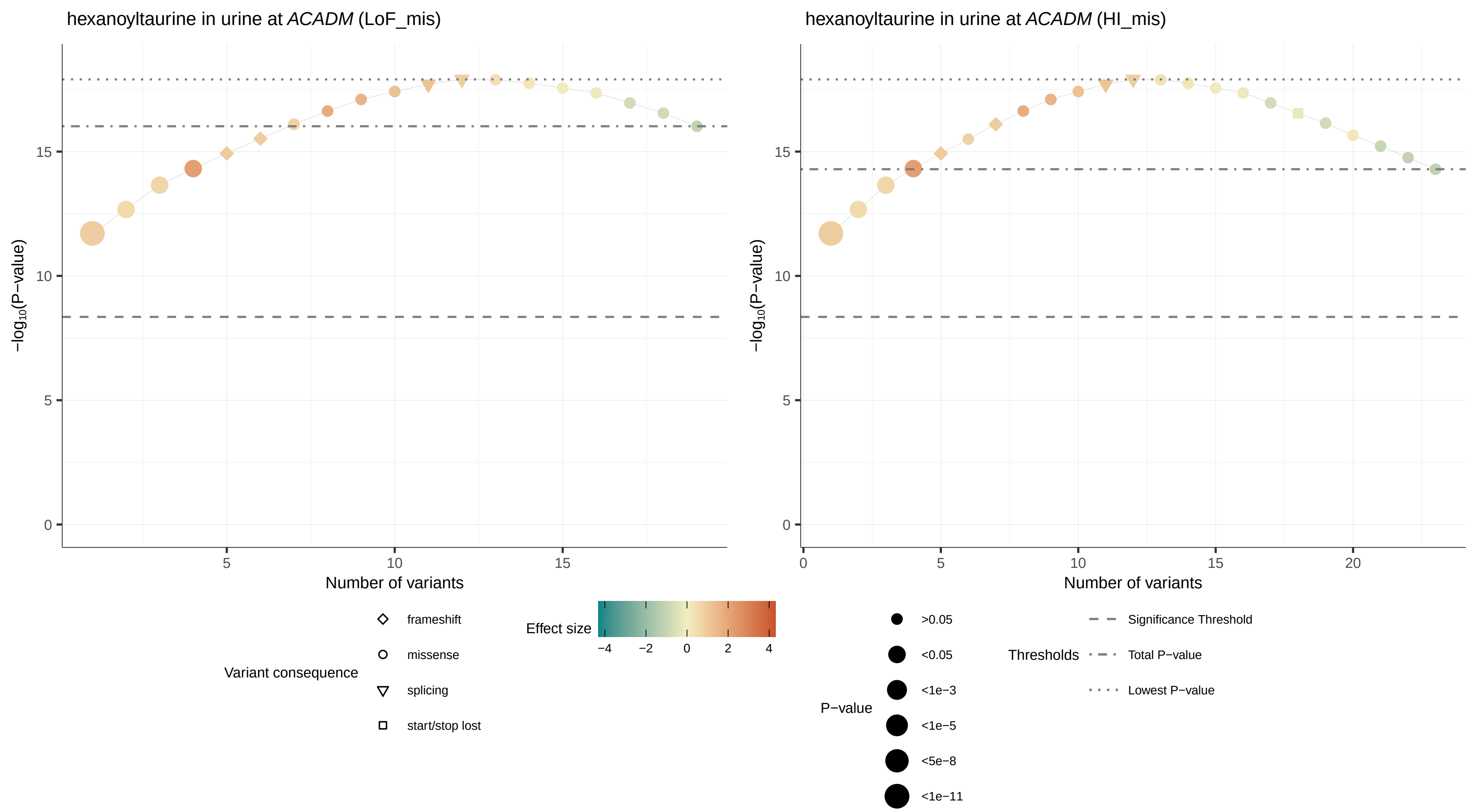

hexanoyltaurine in urine at *ACADM* (LoF_mis)
hexanoyltaurine in urine at *ACADM* (HI_mis)
−log10(P−value)
Number of variants
0
5
10
15
20
Variant consequence
frameshift
missense
splicing
start/stop lost
Effect size
−4
−2
0
2
4
P−value
>0.05
<0.05
<1e−3
<1e−5
<5e−8
<1e−11
Thresholds
Significance Threshold
Total P−value
Lowest P−value

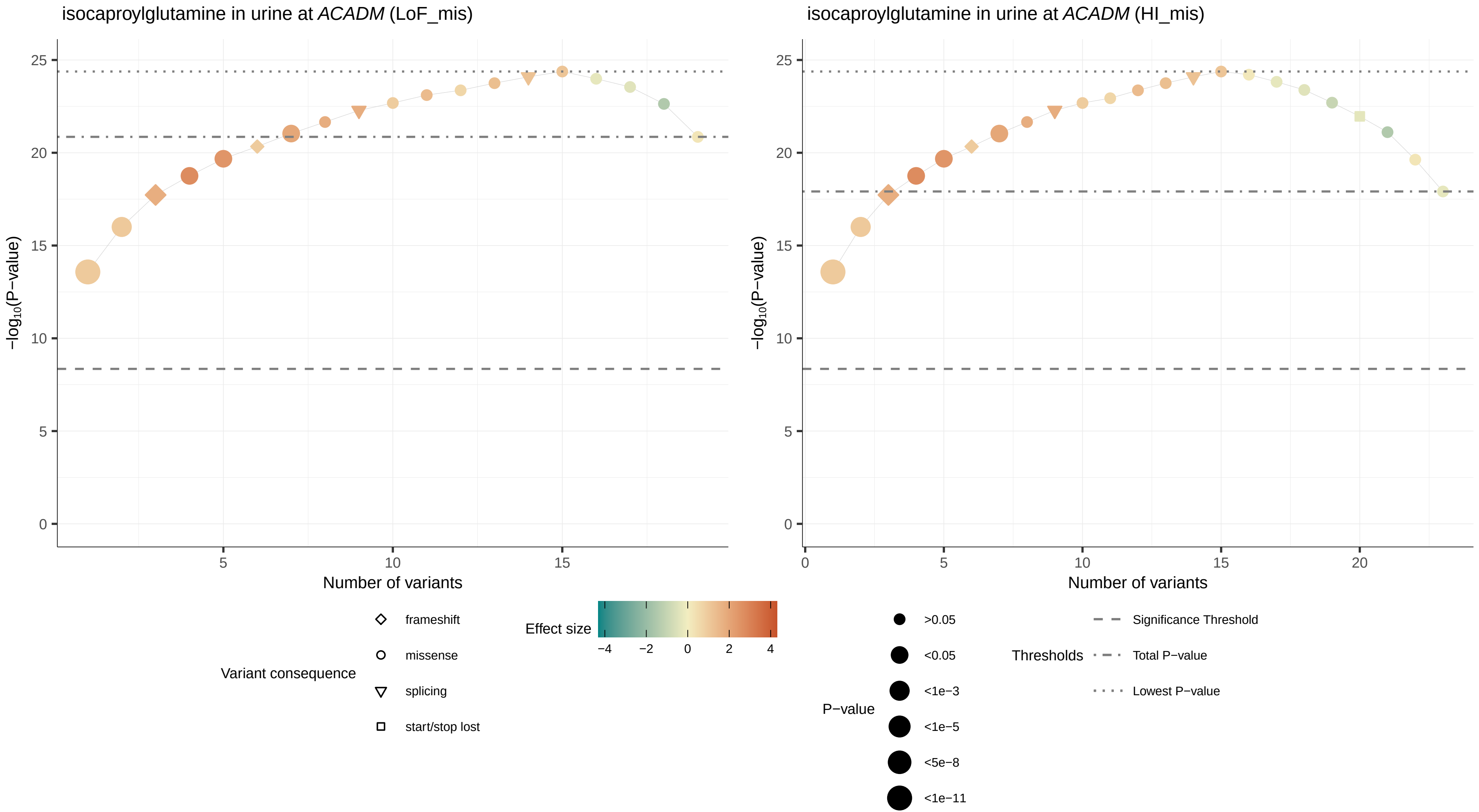

isocaproylglutamine in urine at ACADM (LoF_mis)
isocaproylglutamine in urine at ACADM (HI_mis)
−log10(P−value)
Number of variants
0
5
10
15
20
25
Variant consequence
frameshift
missense
splicing
start/stop lost
Effect size
−4
−2
0
2
4
P−value
>0.05
<0.05
<1e−3
<1e−5
<5e−8
<1e−11
Thresholds
Significance Threshold
Total P−value
Lowest P−value

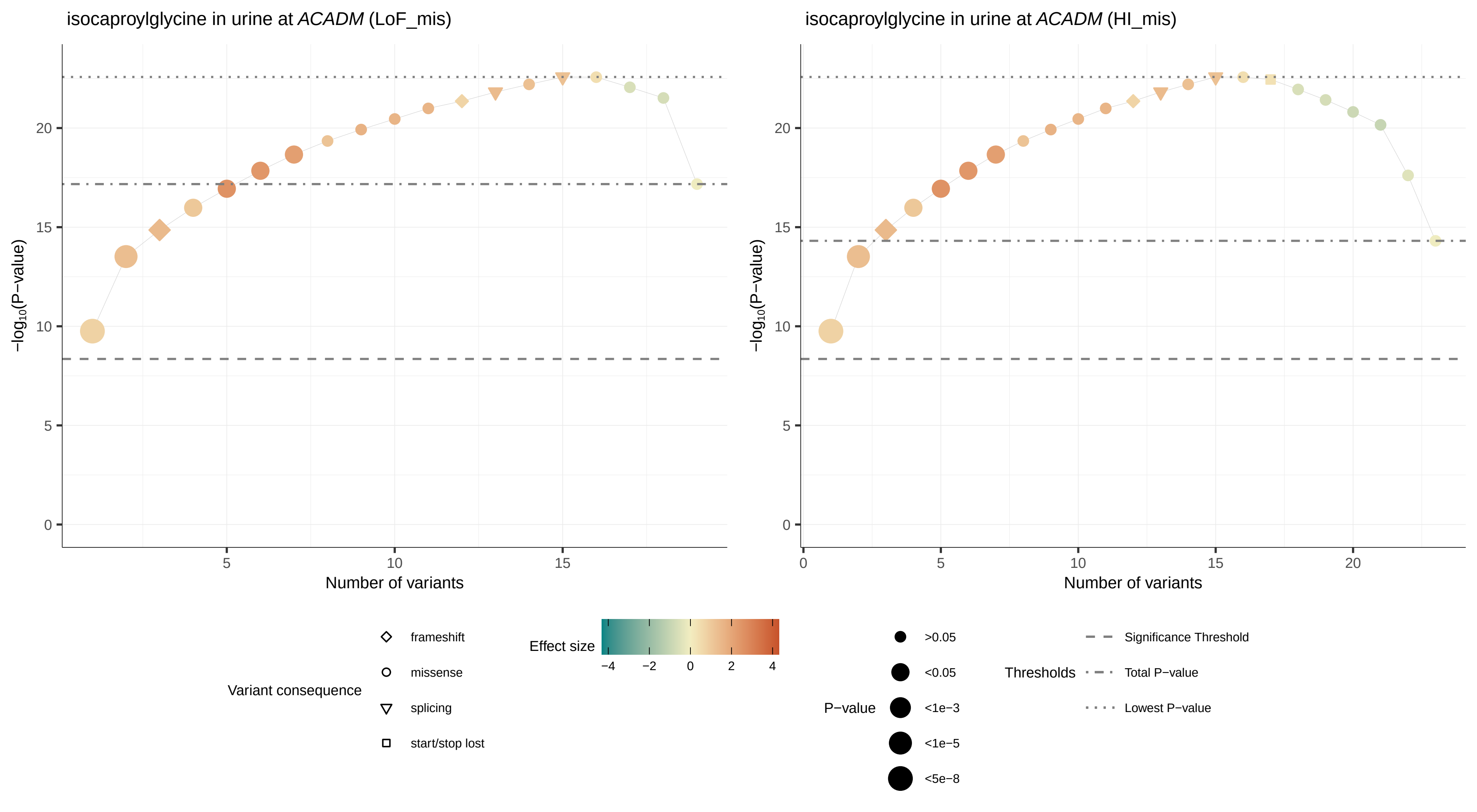

isocaproylglycine in urine at ACADM (LoF_mis)
isocaproylglycine in urine at ACADM (HI_mis)
−log10(P−value)
Number of variants
0
5
10
15
20
Variant consequence
frameshift
missense
splicing
start/stop lost
Effect size
−4
−2
0
2
4
P−value
>0.05
<0.05
<1e−3
<1e−5
<5e−8
Thresholds
Significance Threshold
Total P−value
Lowest P−value

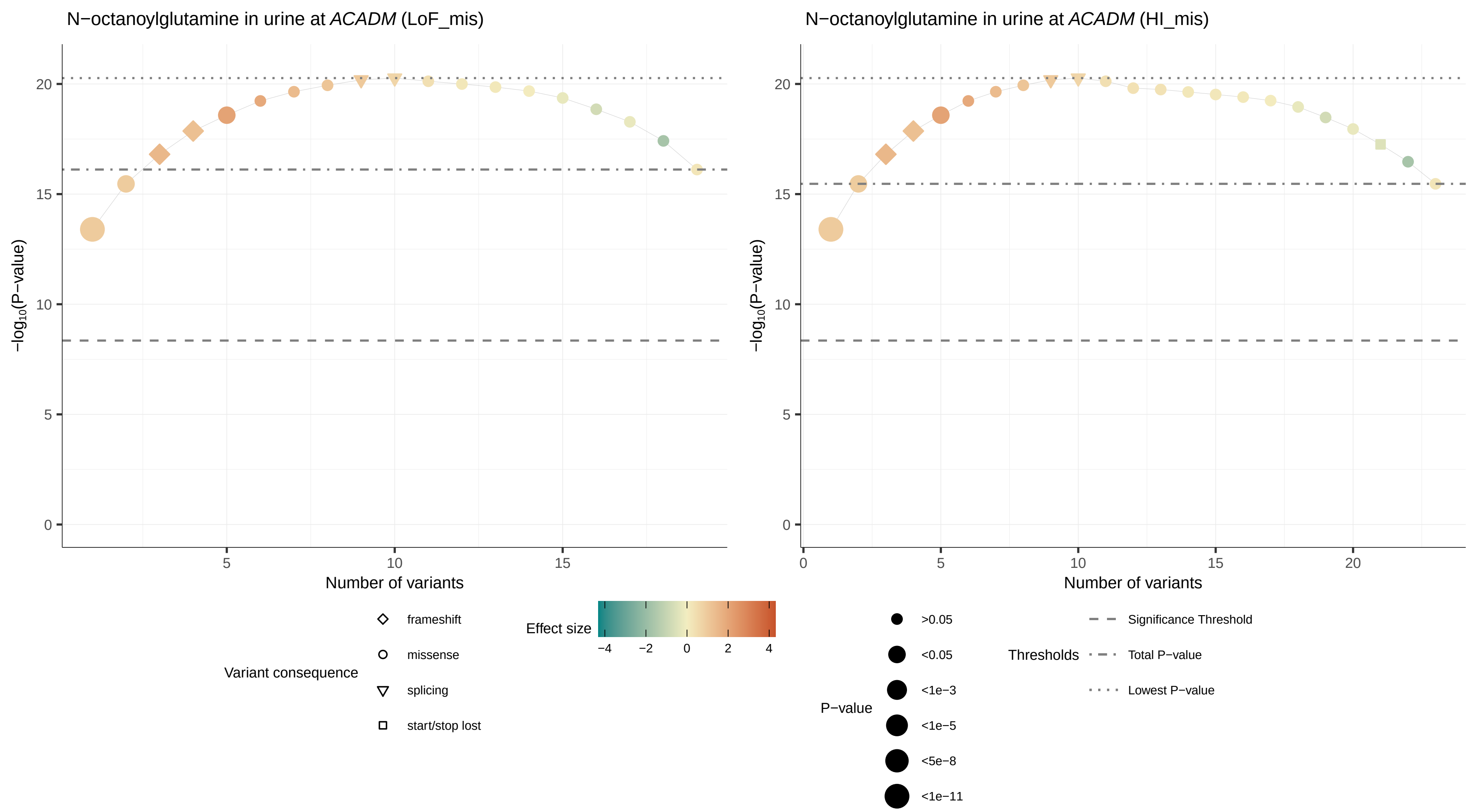

N−octanoylglutamine in urine at *ACADM* (LoF_mis)
N−octanoylglutamine in urine at *ACADM* (HI_mis)
−log10(P−value)
Number of variants
0
5
10
15
20
Variant consequence
frameshift
missense
splicing
start/stop lost
Effect size
−4
−2
0
2
4
P−value
>0.05
<0.05
<1e−3
<1e−5
<5e−8
<1e−11
Thresholds
Significance Threshold
Total P−value
Lowest P−value

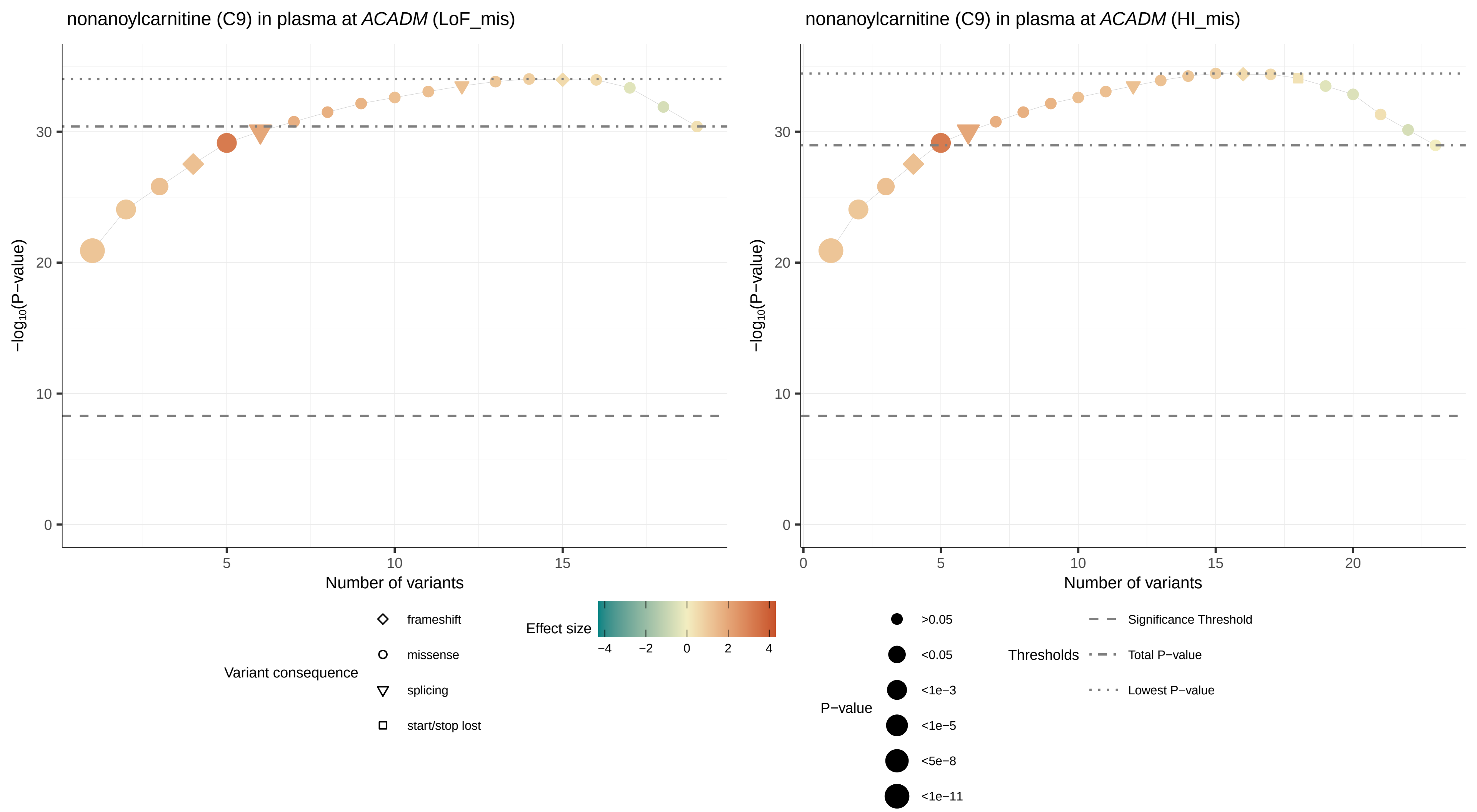

nonanoylcarnitine (C9) in plasma at ACADM (LoF_mis)
nonanoylcarnitine (C9) in plasma at ACADM (HI_mis)
−log10(P−value)
0
10
20
30
5
10
15
20
Number of variants
Variant consequence
frameshift
missense
splicing
start/stop lost
Effect size
−4
−2
0
2
4
P−value
>0.05
<0.05
<1e−3
<1e−5
<5e−8
<1e−11
Thresholds
Significance Threshold
Total P−value
Lowest P−value

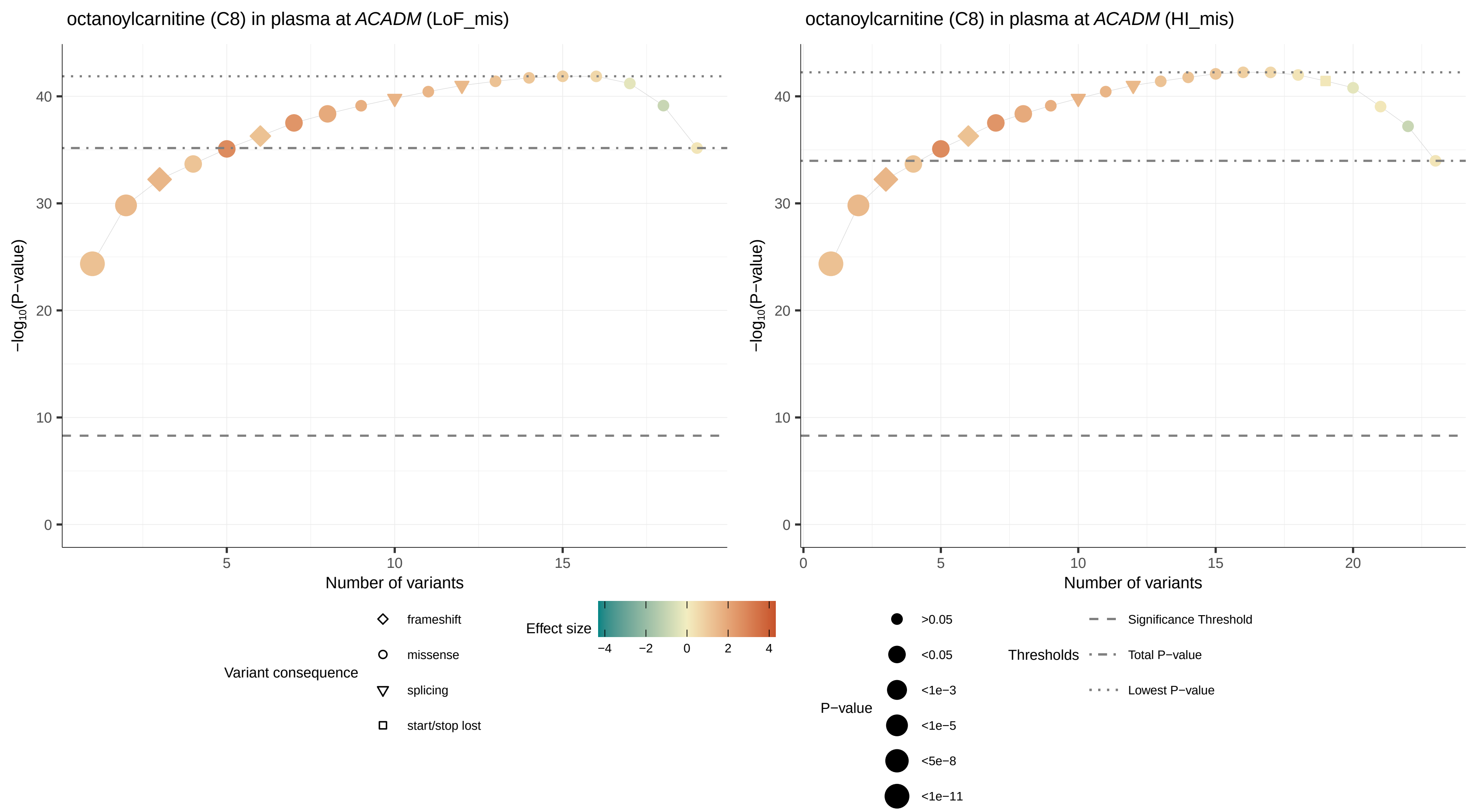

octanoylcarnitine (C8) in plasma at ACADM (LoF_mis)
octanoylcarnitine (C8) in plasma at ACADM (HI_mis)
−log10(P−value)
Number of variants
Variant consequence
frameshift
missense
splicing
start/stop lost
Effect size
−4
−2
0
2
4
P−value
>0.05
<0.05
<1e−3
<1e−5
<5e−8
<1e−11
Thresholds
Significance Threshold
Total P−value
Lowest P−value

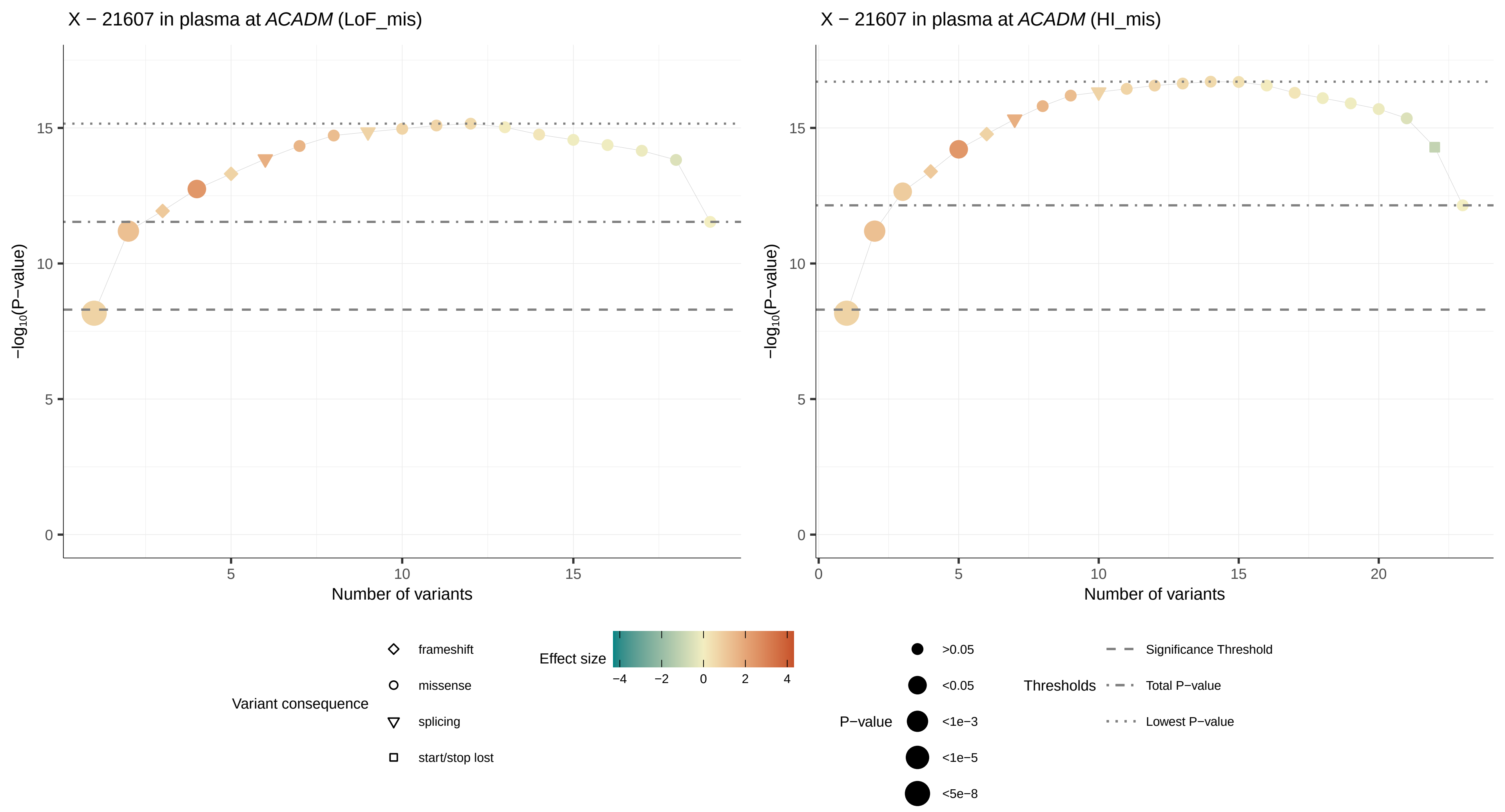

X − 21607 in plasma at ACADM (LoF_mis)
X − 21607 in plasma at ACADM (HI_mis)
−log10(P−value)
0
5
10
15
20
Number of variants
Variant consequence
frameshift
missense
splicing
start/stop lost
Effect size
−4
−2
0
2
4
P−value
>0.05
<0.05
<1e−3
<1e−5
<5e−8
Thresholds
Significance Threshold
Total P−value
Lowest P−value

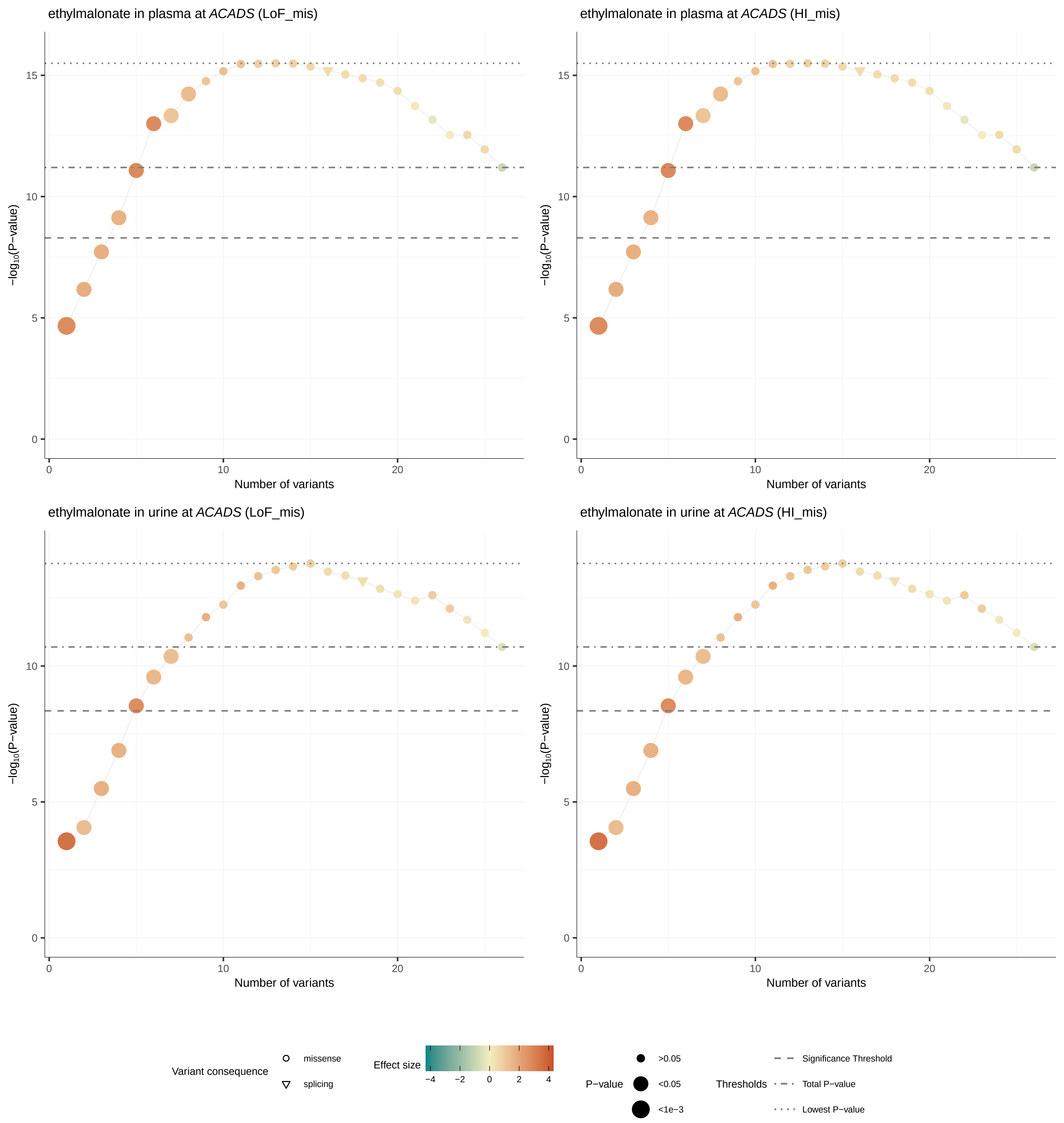
ethylmalonate in plasma at ACADS (LoF_mis)
ethylmalonate in plasma at ACADS (HI_mis)
ethylmalonate in urine at ACADS (LoF_mis)
ethylmalonate in urine at ACADS (HI_mis)
−log10(P−value)
Number of variants
Variant consequence
missense
splicing
Effect size
P−value
>0.05
<0.05
<1e−3
Thresholds
Significance Threshold
Total P−value
Lowest P−value

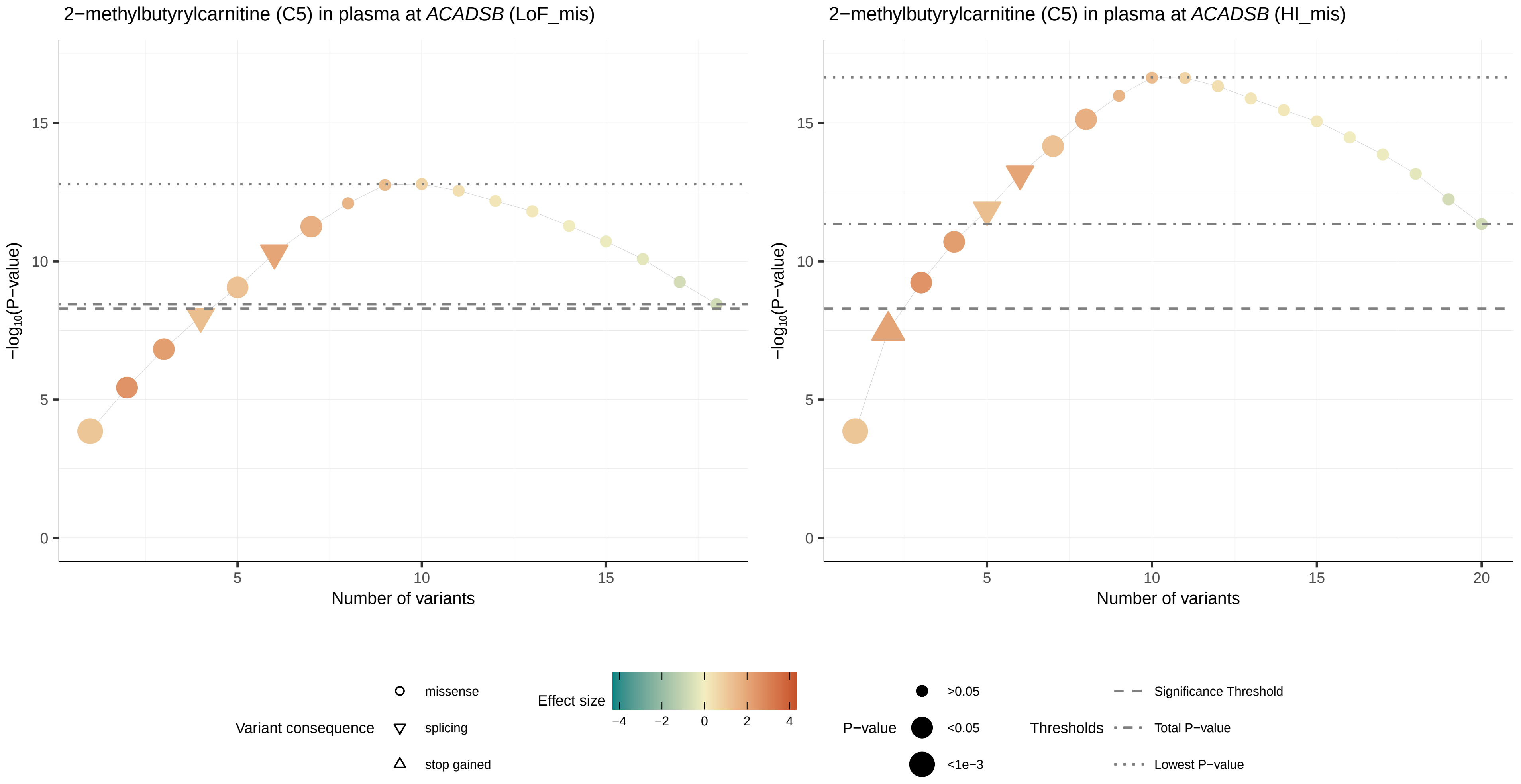

2−methylbutyrylcarnitine (C5) in plasma at ACADSB (LoF_mis)
2−methylbutyrylcarnitine (C5) in plasma at ACADSB (HI_mis)
−log10(P−value)
Number of variants
Variant consequence
missense
splicing
stop gained
Effect size
P−value
>0.05
<0.05
<1e−3
Thresholds
Significance Threshold
Total P−value
Lowest P−value

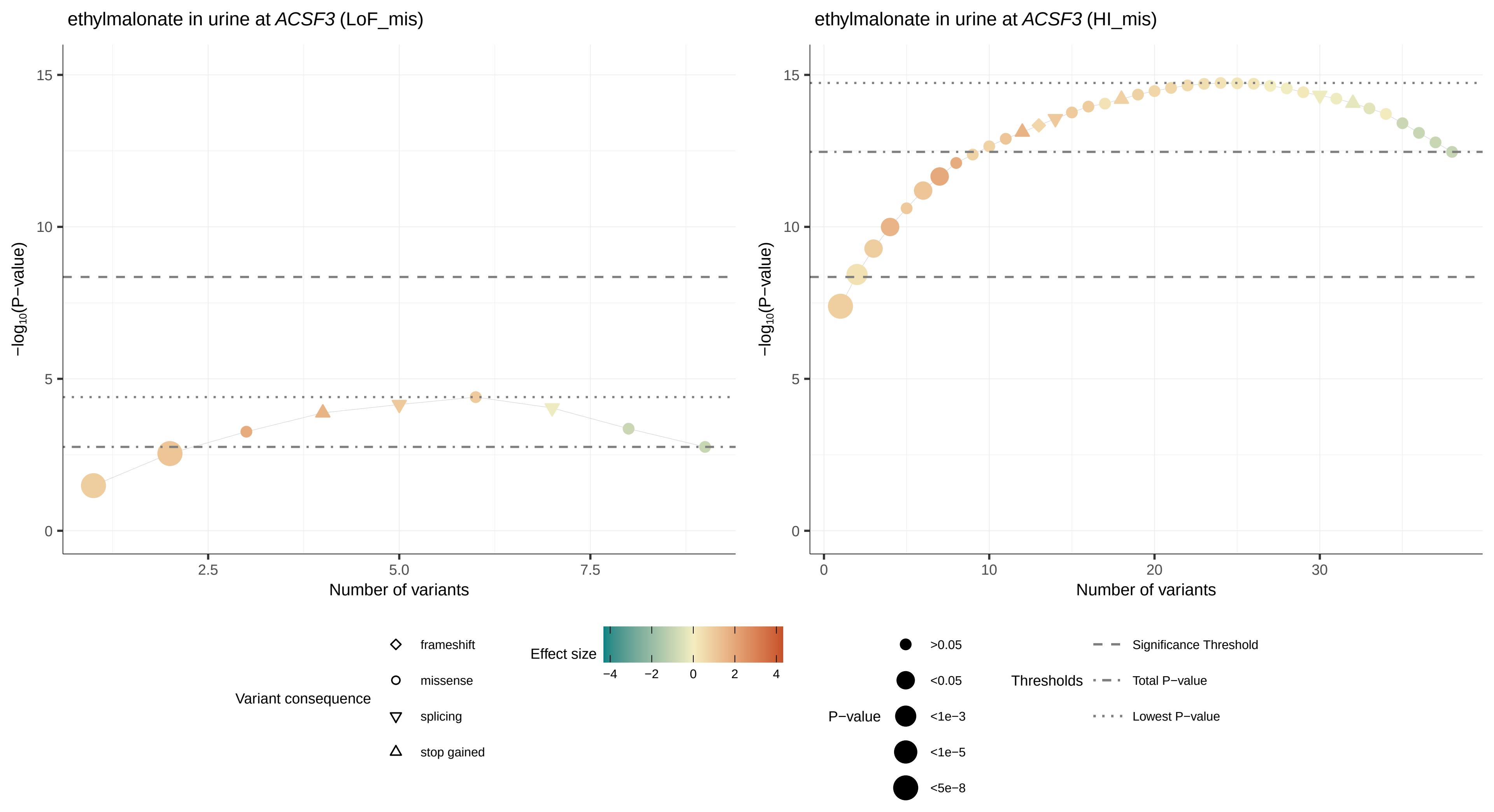
ethylmalonate in urine at ACSF3 (LoF_mis)
ethylmalonate in urine at ACSF3 (HI_mis)
−log10(P−value)
Number of variants
Variant consequence
frameshift
missense
splicing
stop gained
Effect size
P−value
>0.05
<0.05
<1e−3
<1e−5
<5e−8
Thresholds
Significance Threshold
Total P−value
Lowest P−value

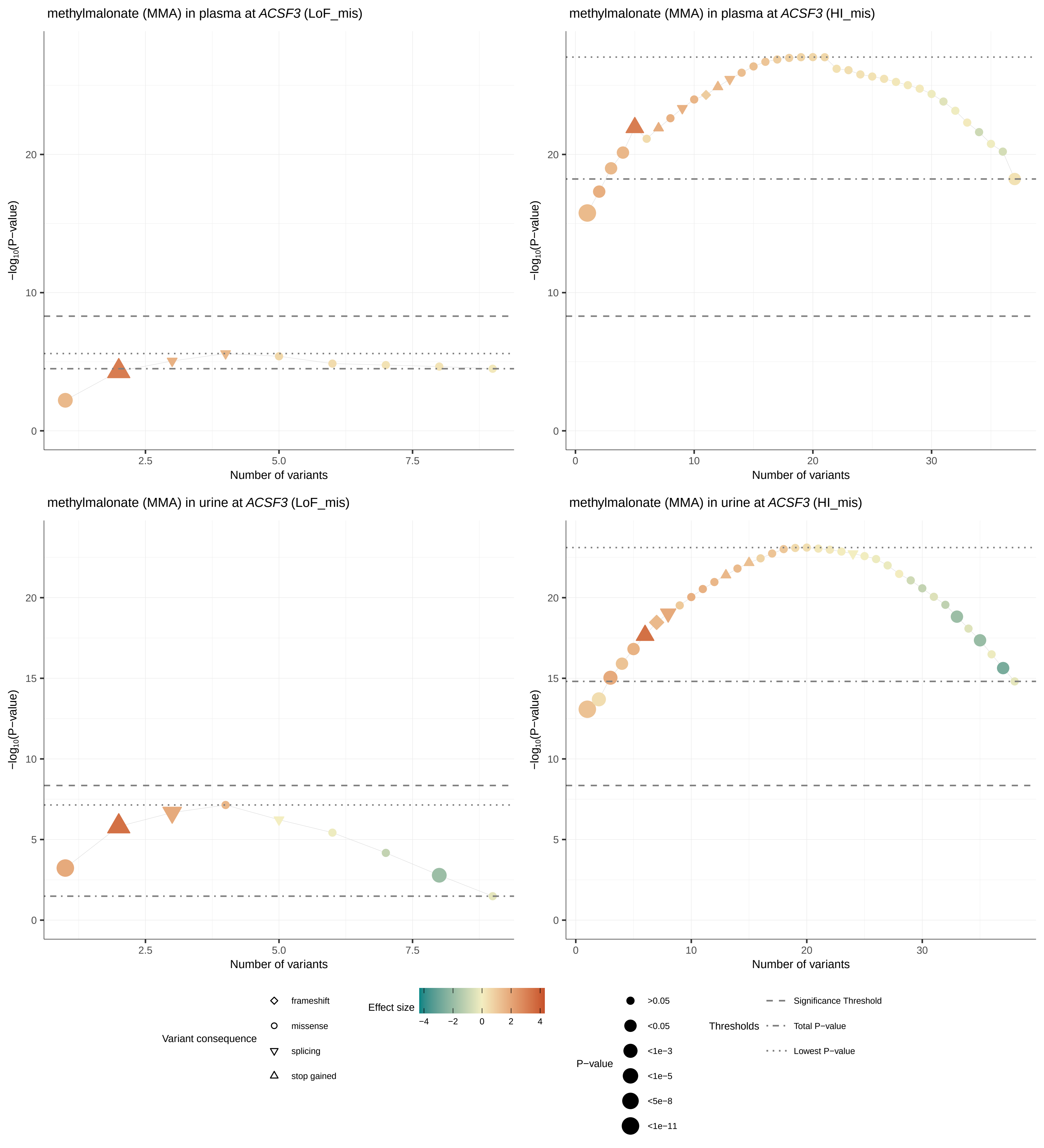
methylmalonate (MMA) in plasma at ACSF3 (LoF_mis)
methylmalonate (MMA) in plasma at ACSF3 (HI_mis)
methylmalonate (MMA) in urine at ACSF3 (LoF_mis)
methylmalonate (MMA) in urine at ACSF3 (HI_mis)
−log10(P−value)
Number of variants
Variant consequence
frameshift
missense
splicing
stop gained
Effect size
−4
−2
0
2
4
P−value
>0.05
<0.05
<1e−3
<1e−5
<5e−8
<1e−11
Thresholds
Significance Threshold
Total P−value
Lowest P−value

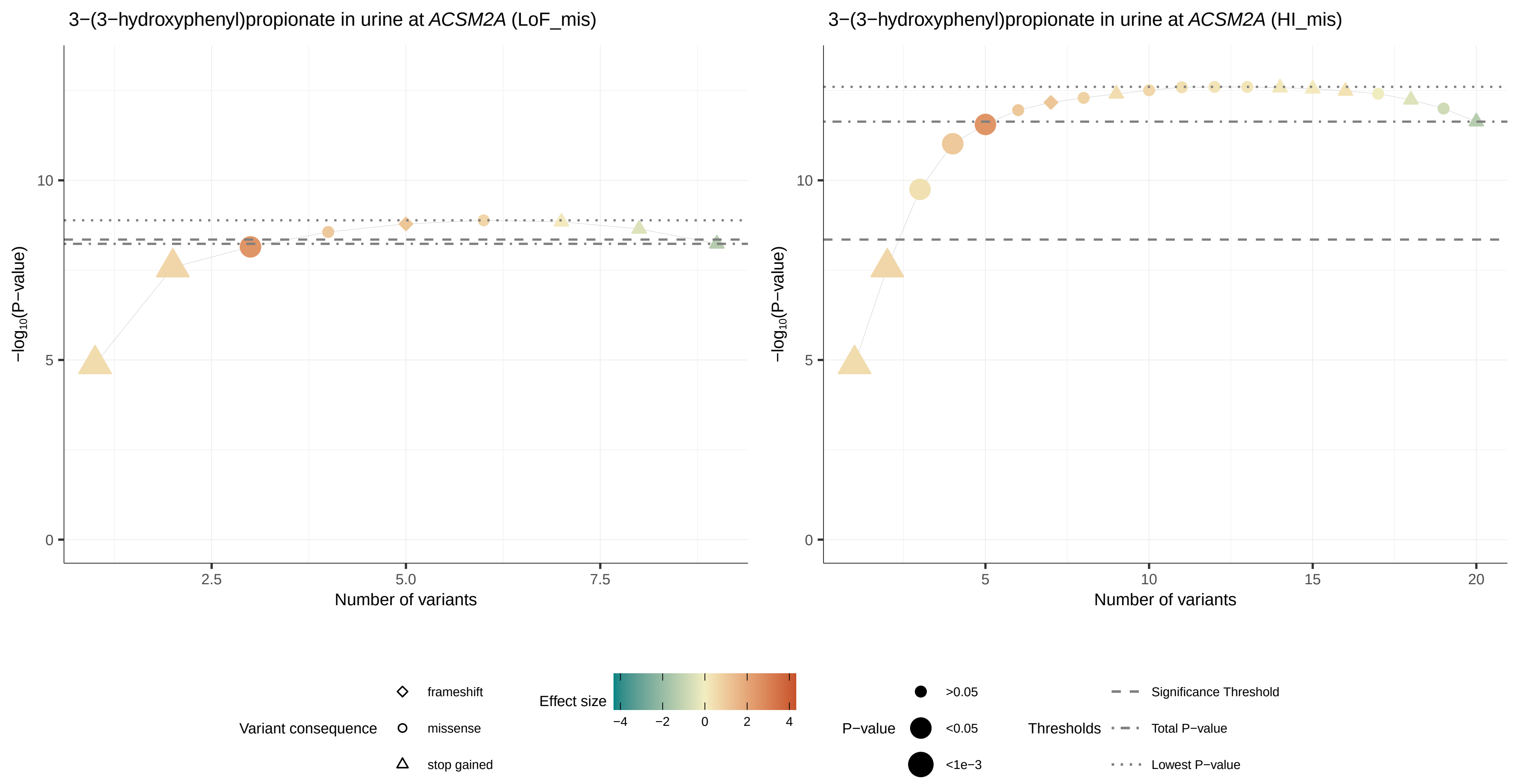

3−(3−hydroxyphenyl)propionate in urine at ACSM2A (LoF_mis)
3−(3−hydroxyphenyl)propionate in urine at ACSM2A (HI_mis)
−log10(P−value)
Number of variants
Variant consequence
frameshift
missense
stop gained
Effect size
−4
−2
0
2
4
P−value
>0.05
<0.05
<1e−3
Thresholds
Significance Threshold
Total P−value
Lowest P−value

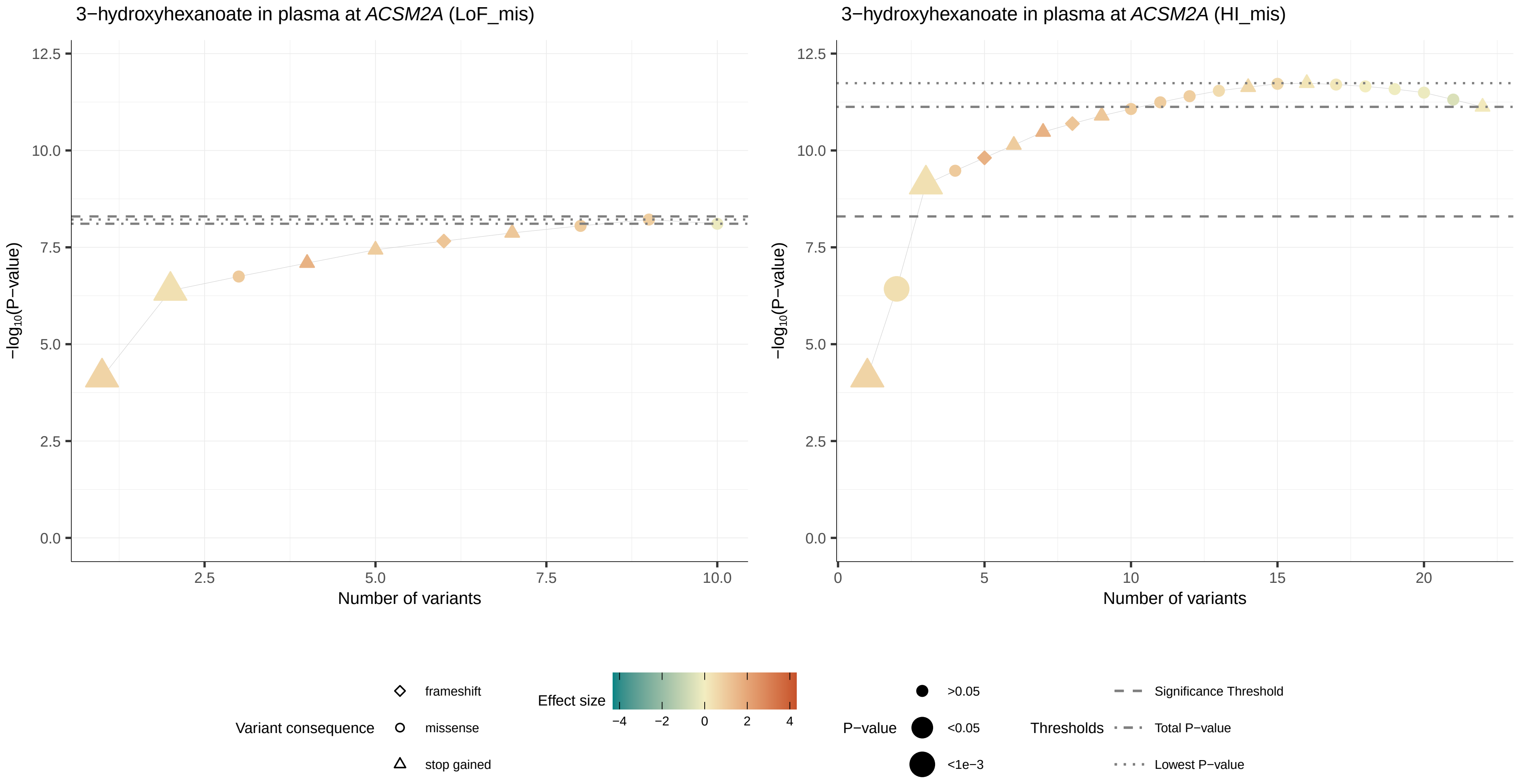

3−hydroxyhexanoate in plasma at ACSM2A (LoF_mis)
3−hydroxyhexanoate in plasma at ACSM2A (HI_mis)
−log10(P−value)
Number of variants
12.5
10.0
7.5
5.0
2.5
0.0
Variant consequence
frameshift
missense
stop gained
Effect size
−4
−2
0
2
4
P−value
>0.05
<0.05
<1e−3
Thresholds
Significance Threshold
Total P−value
Lowest P−value

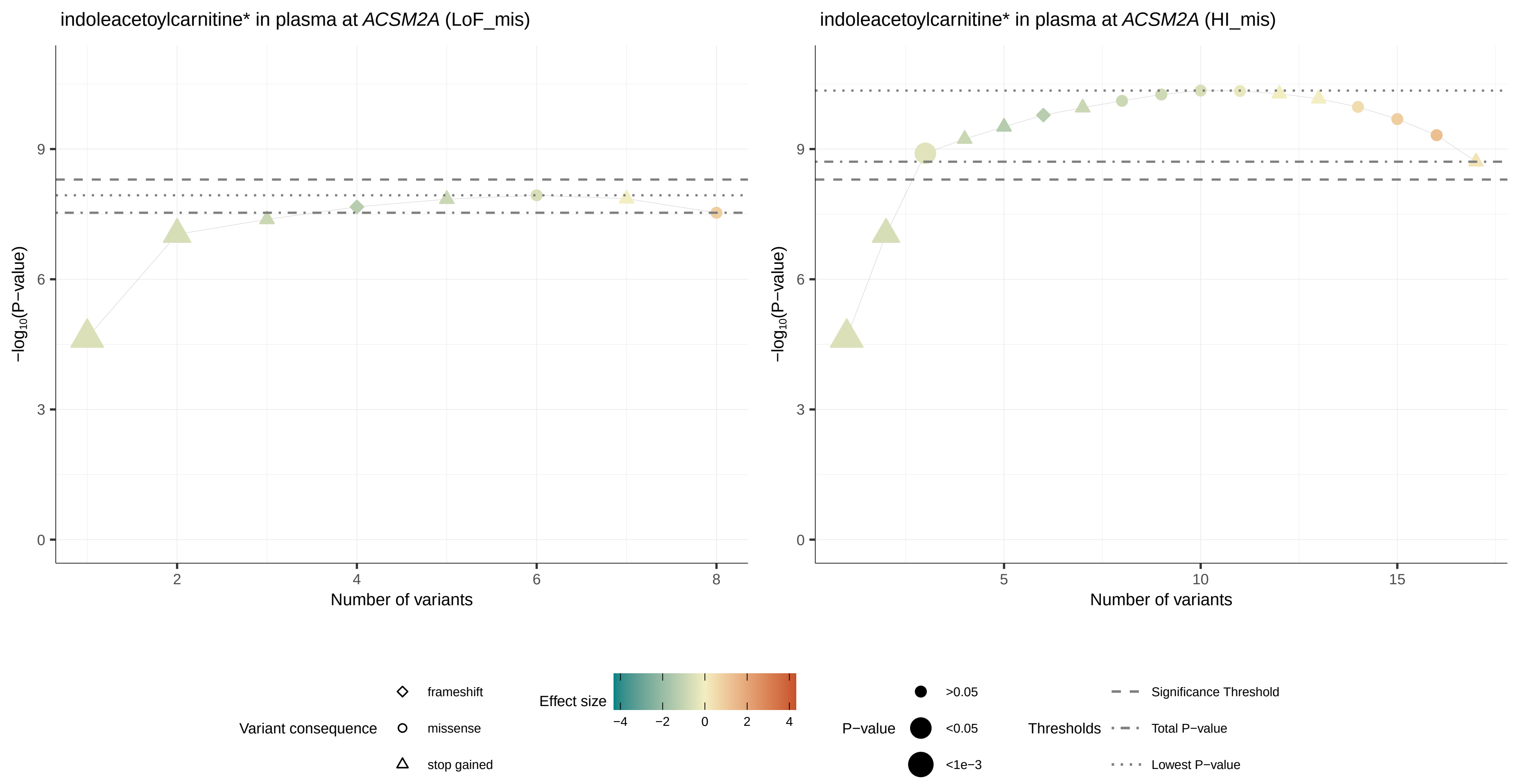

indoleacetoylcarnitine* in plasma at ACSM2A (LoF_mis)
indoleacetoylcarnitine* in plasma at ACSM2A (HI_mis)
−log10(P−value)
Number of variants
Variant consequence
frameshift
missense
stop gained
Effect size
−4
−2
0
2
4
P−value
>0.05
<0.05
<1e−3
Thresholds
Significance Threshold
Total P−value
Lowest P−value

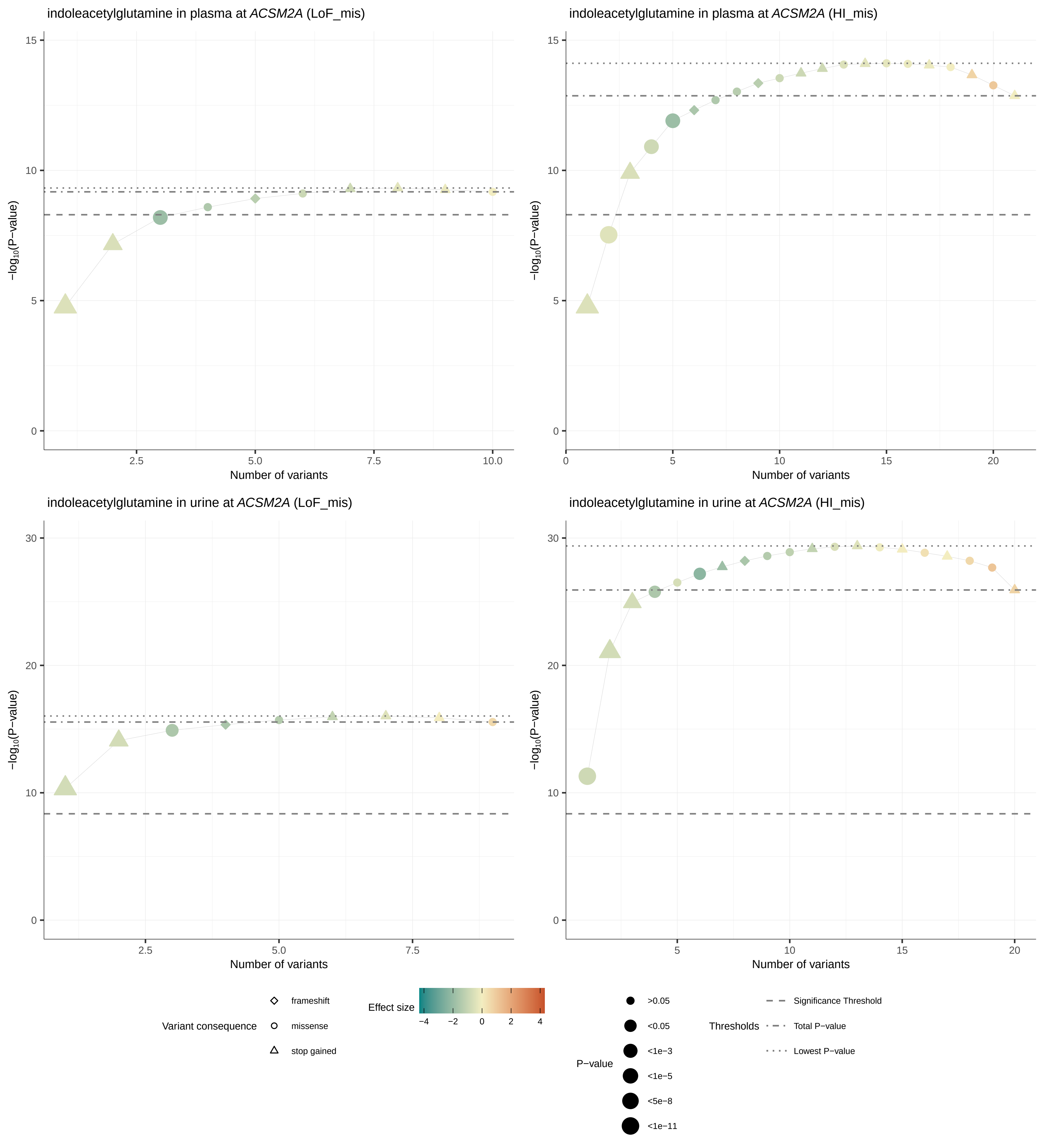
indoleacetylglutamine in plasma at ACSM2A (LoF_mis)
indoleacetylglutamine in plasma at ACSM2A (HI_mis)
indoleacetylglutamine in urine at ACSM2A (LoF_mis)
indoleacetylglutamine in urine at ACSM2A (HI_mis)
−log10(P−value)
Number of variants
Variant consequence
frameshift
missense
stop gained
Effect size
−4
−2
0
2
4
P−value
>0.05
<0.05
<1e−3
<1e−5
<5e−8
<1e−11
Thresholds
Significance Threshold
Total P−value
Lowest P−value

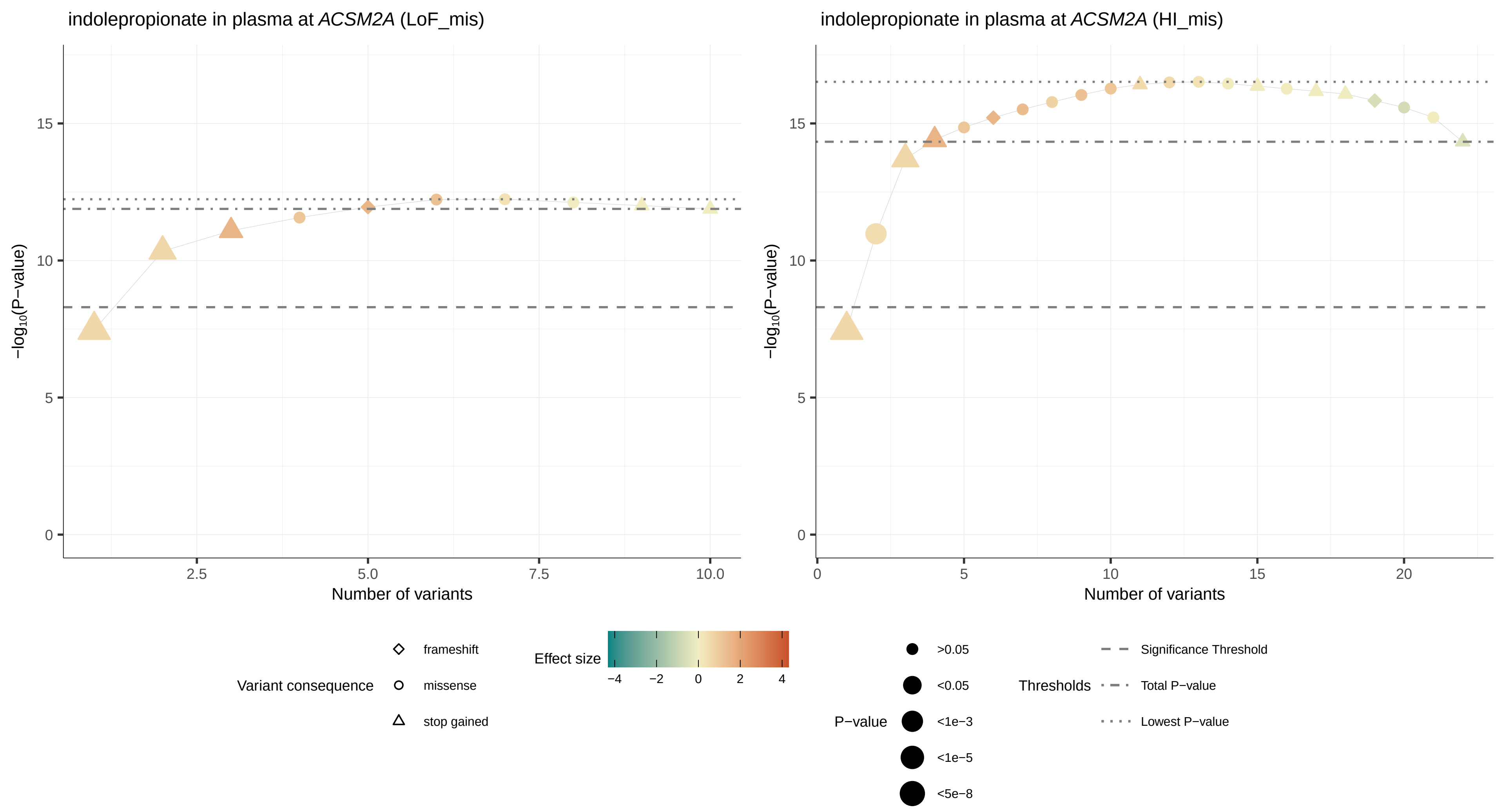
indolepropionate in plasma at ACSM2A (LoF_mis)
indolepropionate in plasma at ACSM2A (HI_mis)
−log10(P−value)
Number of variants
0
5
10
15
2.5
5.0
7.5
10.0
20
Variant consequence
frameshift
missense
stop gained
Effect size
−4
−2
0
2
4
P−value
>0.05
<0.05
<1e−3
<1e−5
<5e−8
Thresholds
Significance Threshold
Total P−value
Lowest P−value

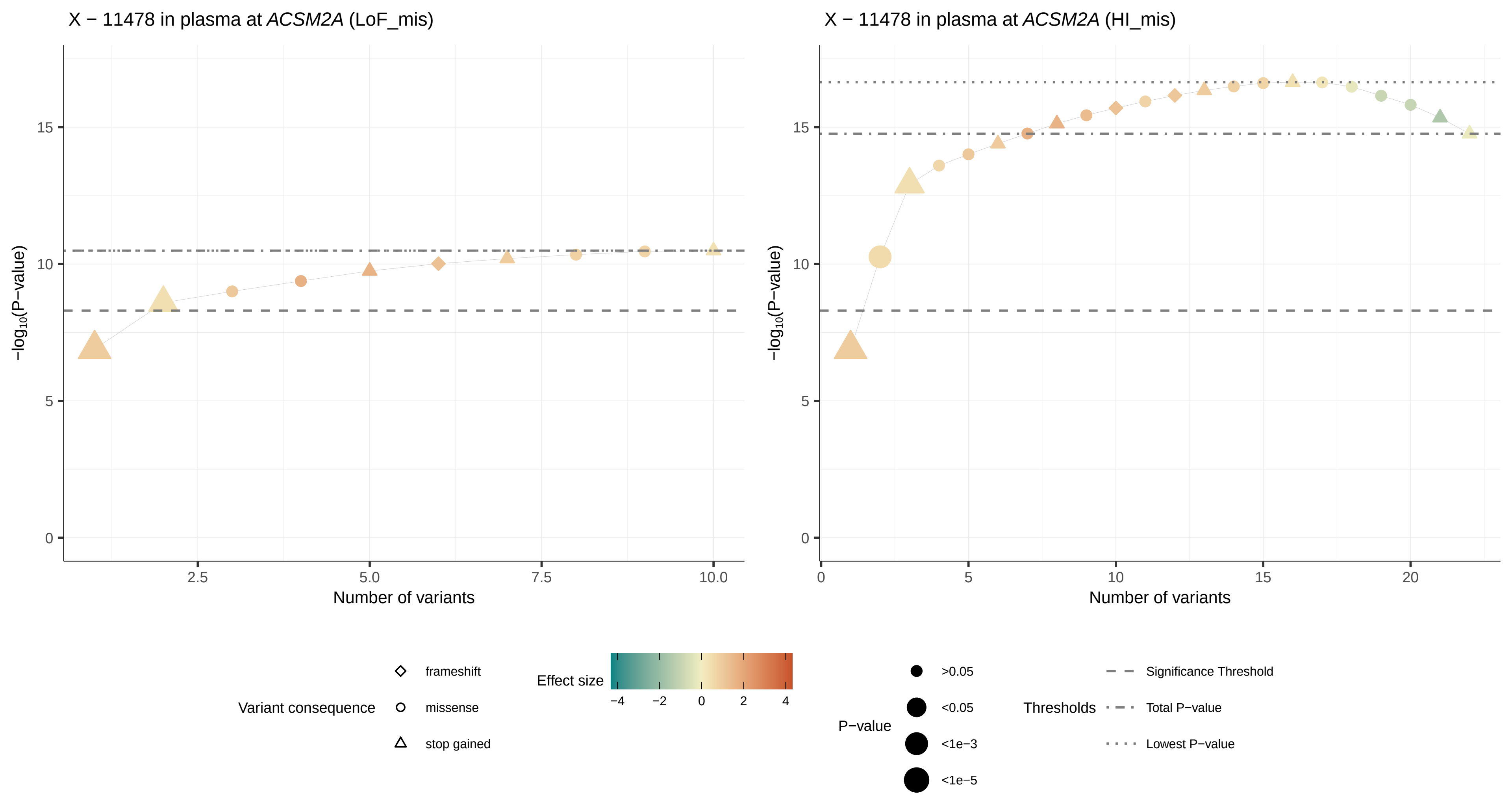
X − 11478 in plasma at ACSM2A (LoF_mis)
X − 11478 in plasma at ACSM2A (HI_mis)
−log10(P−value)
Number of variants
0
5
10
15
2.5
5.0
7.5
10.0
20
Variant consequence
frameshift
missense
stop gained
Effect size
−4
−2
2
4
P−value
>0.05
<0.05
<1e−3
<1e−5
Thresholds
Significance Threshold
Total P−value
Lowest P−value

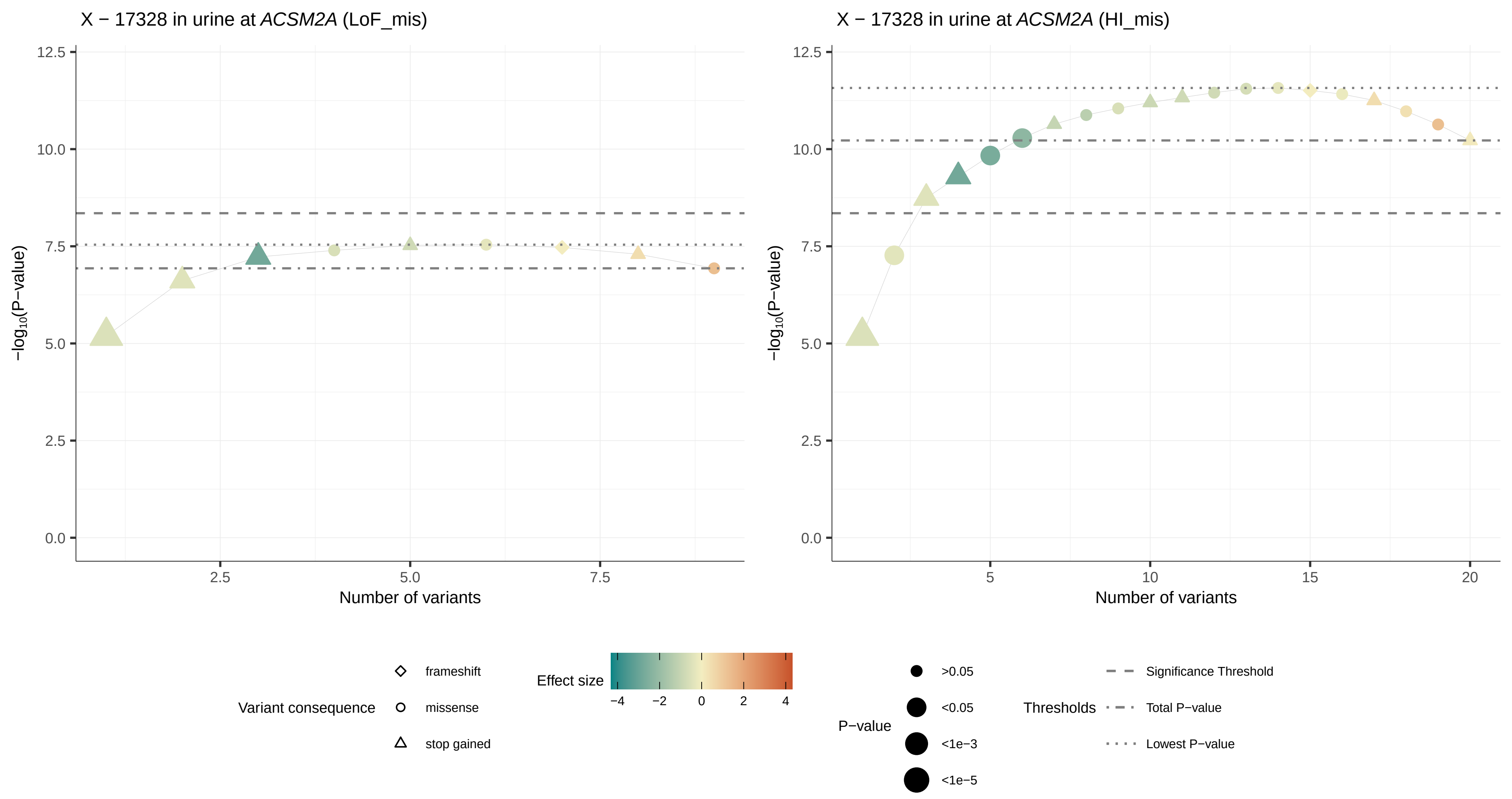

X − 17328 in urine at *ACSM2A* (LoF_mis)
X − 17328 in urine at *ACSM2A* (HI_mis)
−log10(P−value)
Number of variants
Variant consequence
frameshift
missense
stop gained
Effect size
P−value
>0.05
<0.05
<1e−3
<1e−5
Thresholds
Significance Threshold
Total P−value
Lowest P−value

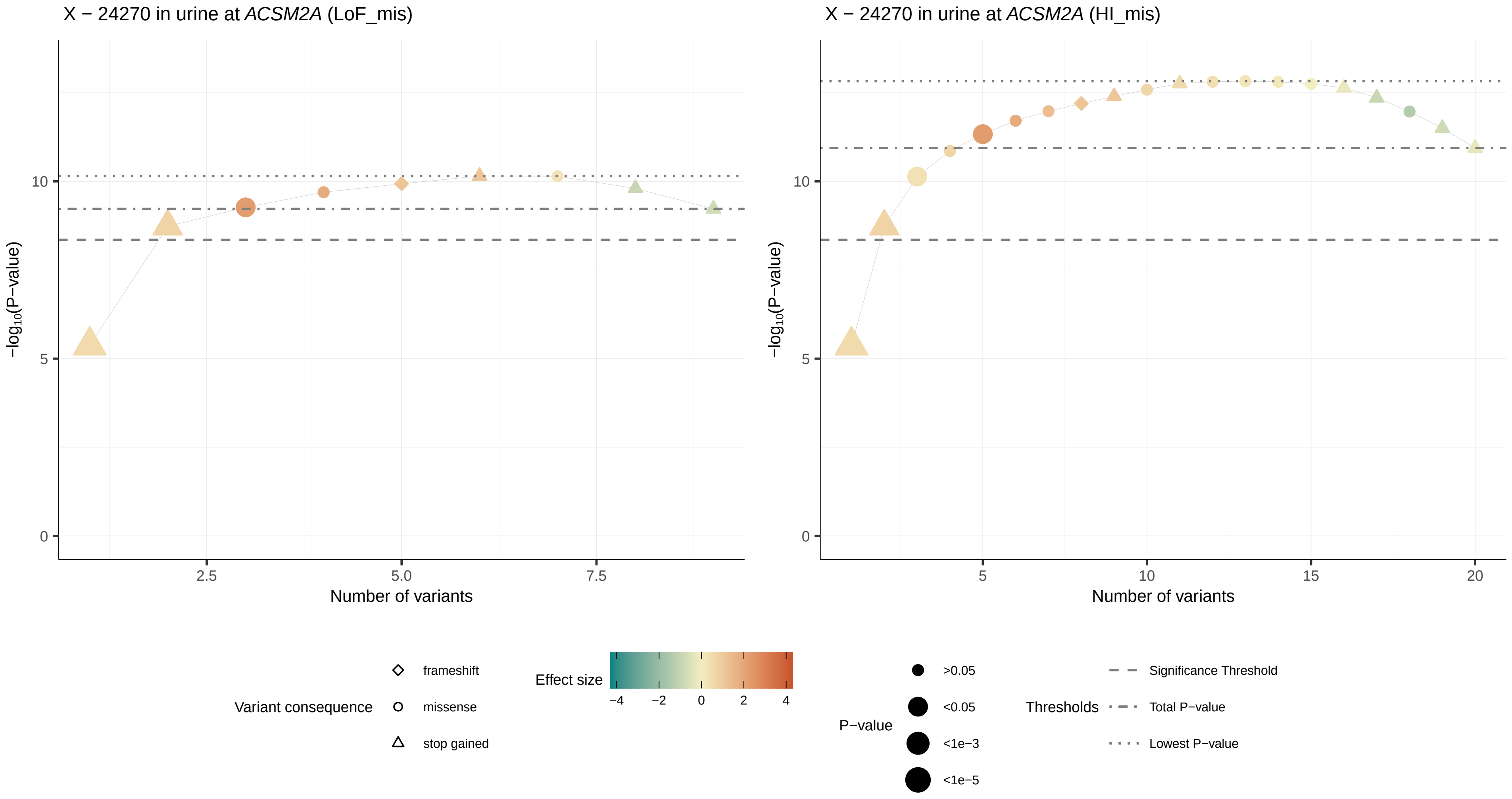

X − 24270 in urine at ACSM2A (LoF_mis)
X − 24270 in urine at ACSM2A (HI_mis)
−log10(P−value)
Number of variants
Variant consequence
frameshift
missense
stop gained
Effect size
−4
−2
0
2
4
P−value
>0.05
<0.05
<1e−3
<1e−5
Thresholds
Significance Threshold
Total P−value
Lowest P−value

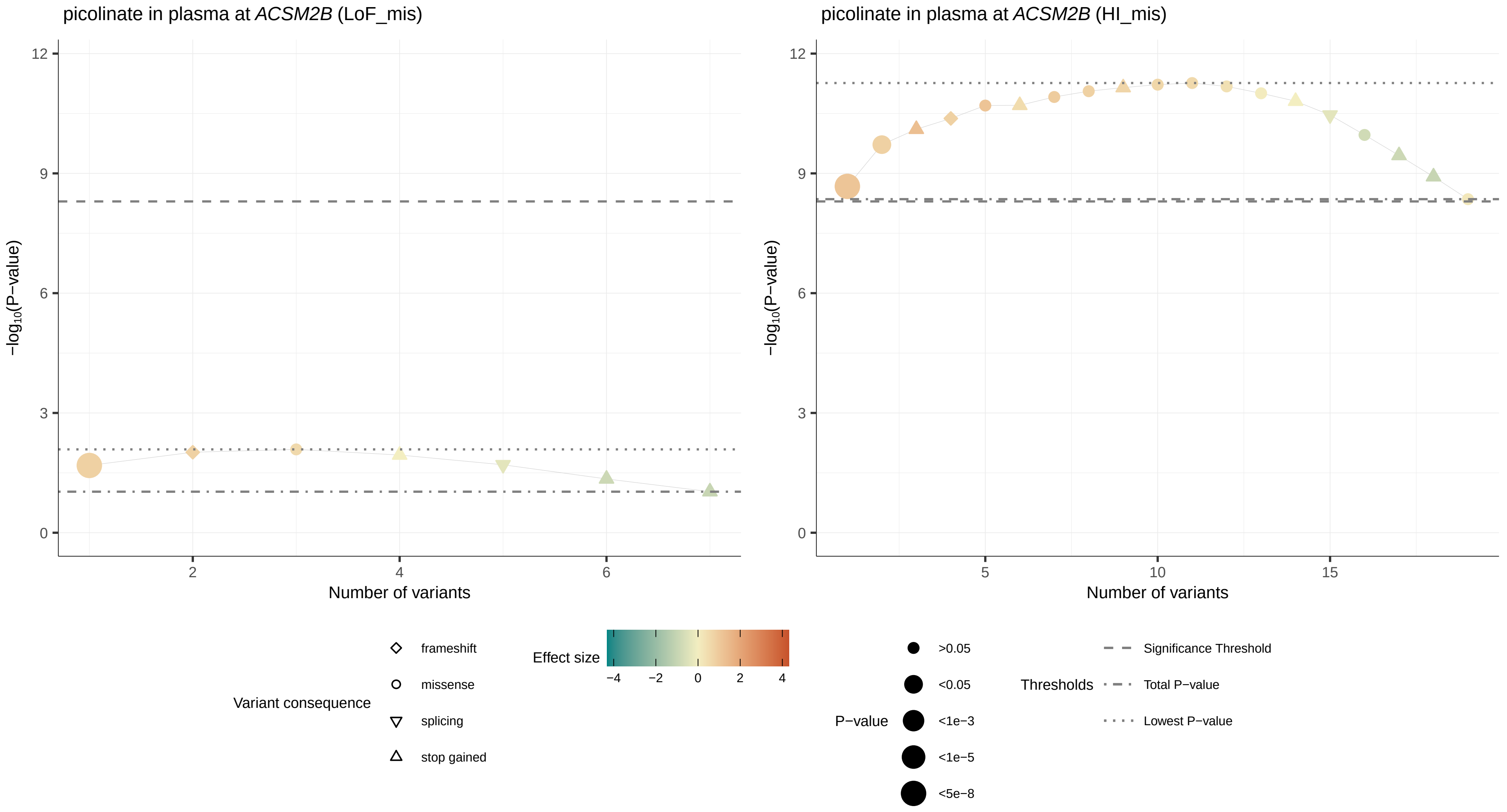

picolinate in plasma at ACSM2B (LoF_mis)
picolinate in plasma at ACSM2B (HI_mis)
−log10(P−value)
Number of variants
0
3
6
9
12
2
4
6
5
10
15
Variant consequence
frameshift
missense
splicing
stop gained
Effect size
−4
−2
0
2
4
P−value
>0.05
<0.05
<1e−3
<1e−5
<5e−8
Thresholds
Significance Threshold
Total P−value
Lowest P−value

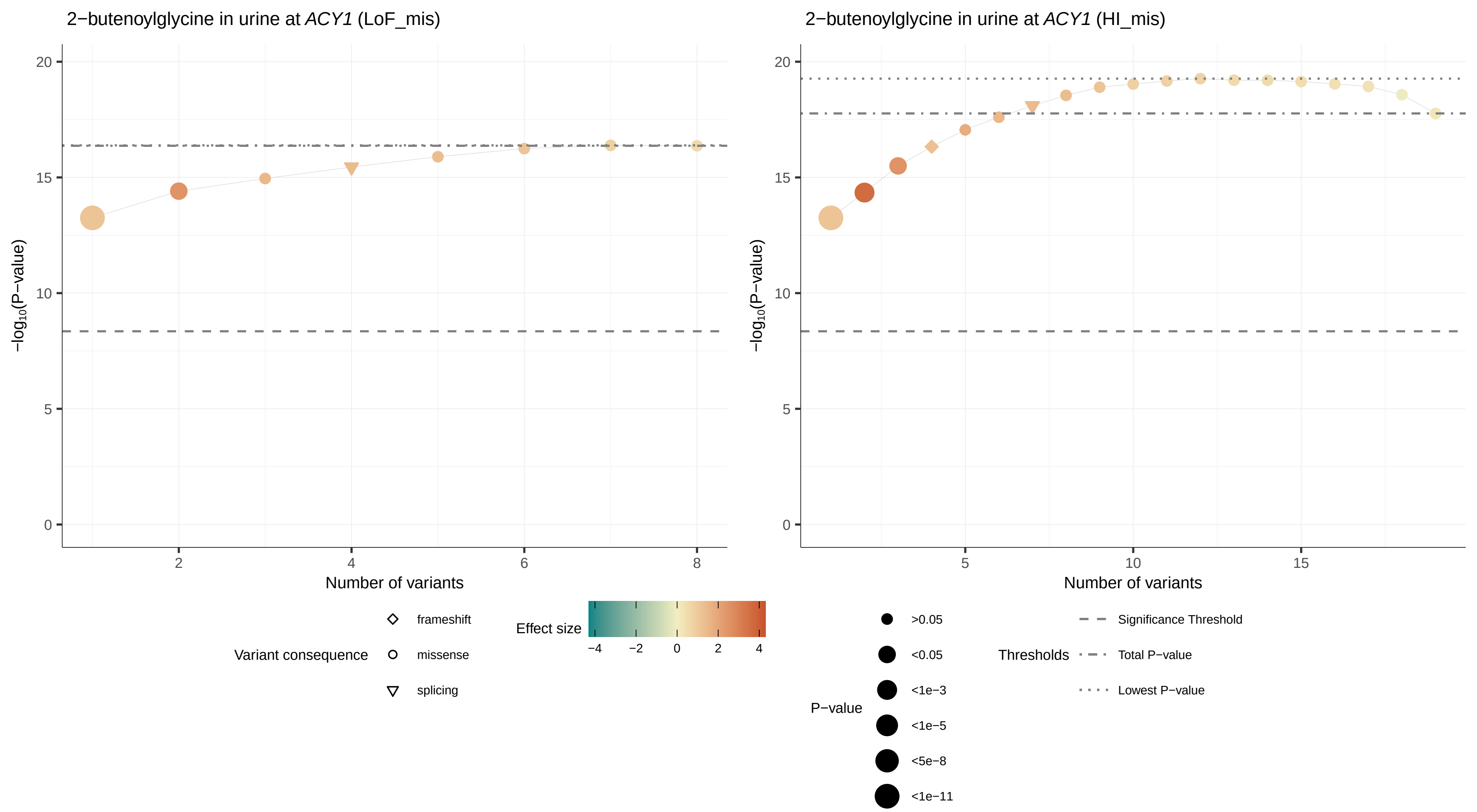

2−butenoylglycine in urine at ACY1 (LoF_mis)
2−butenoylglycine in urine at ACY1 (HI_mis)
−log10(P−value)
Number of variants
Variant consequence
frameshift
missense
splicing
Effect size
P−value
>0.05
<0.05
<1e−3
<1e−5
<5e−8
<1e−11
Thresholds
Significance Threshold
Total P−value
Lowest P−value

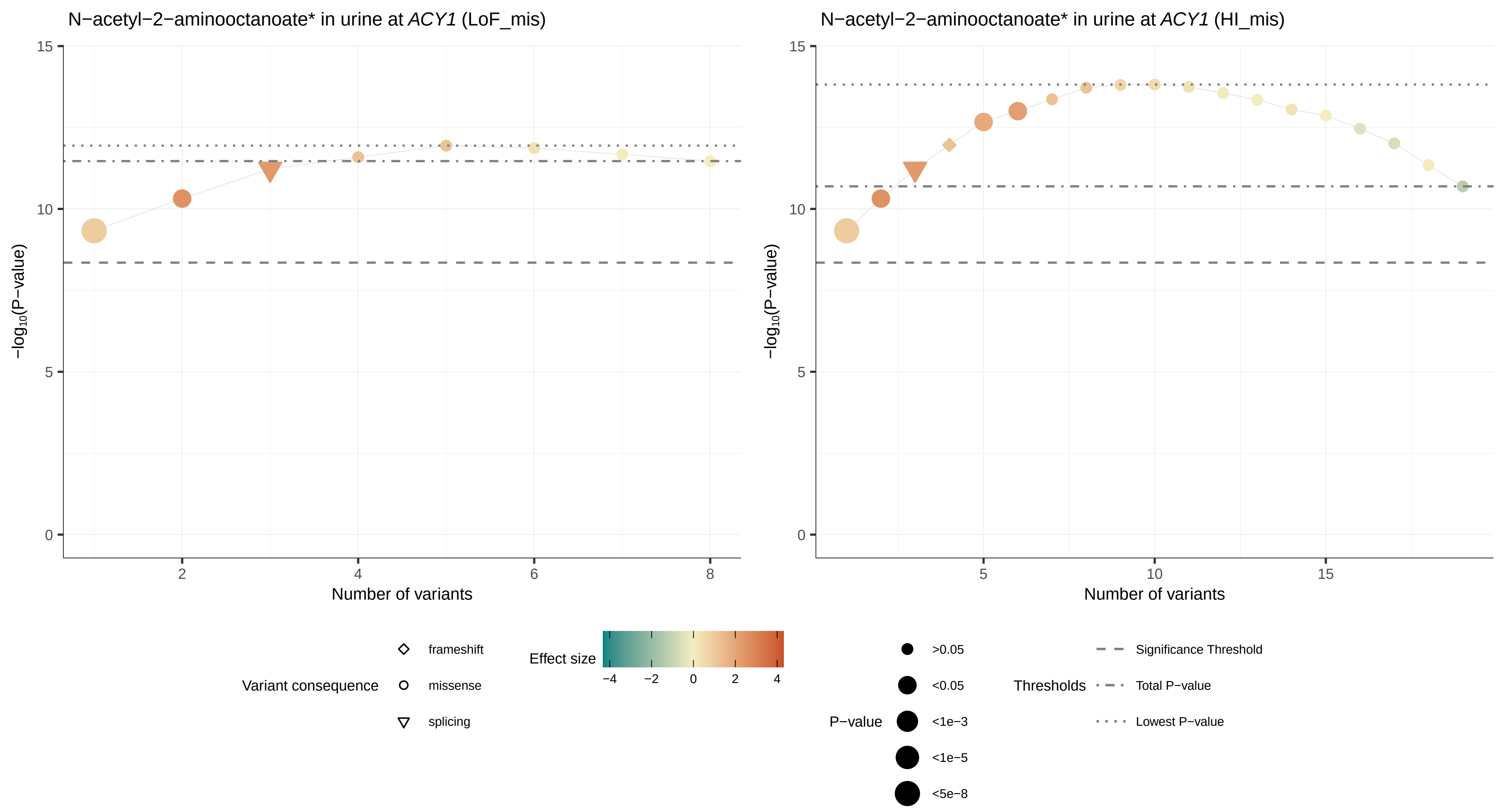

N−acetyl−2−aminooctanoate* in urine at ACY1 (LoF_mis)
N−acetyl−2−aminooctanoate* in urine at ACY1 (HI_mis)
−log10(P−value)
Number of variants
Variant consequence
frameshift
missense
splicing
Effect size
−4 −2 0 2 4
P−value
>0.05
<0.05
<1e−3
<1e−5
<5e−8
Thresholds
Significance Threshold
Total P−value
Lowest P−value

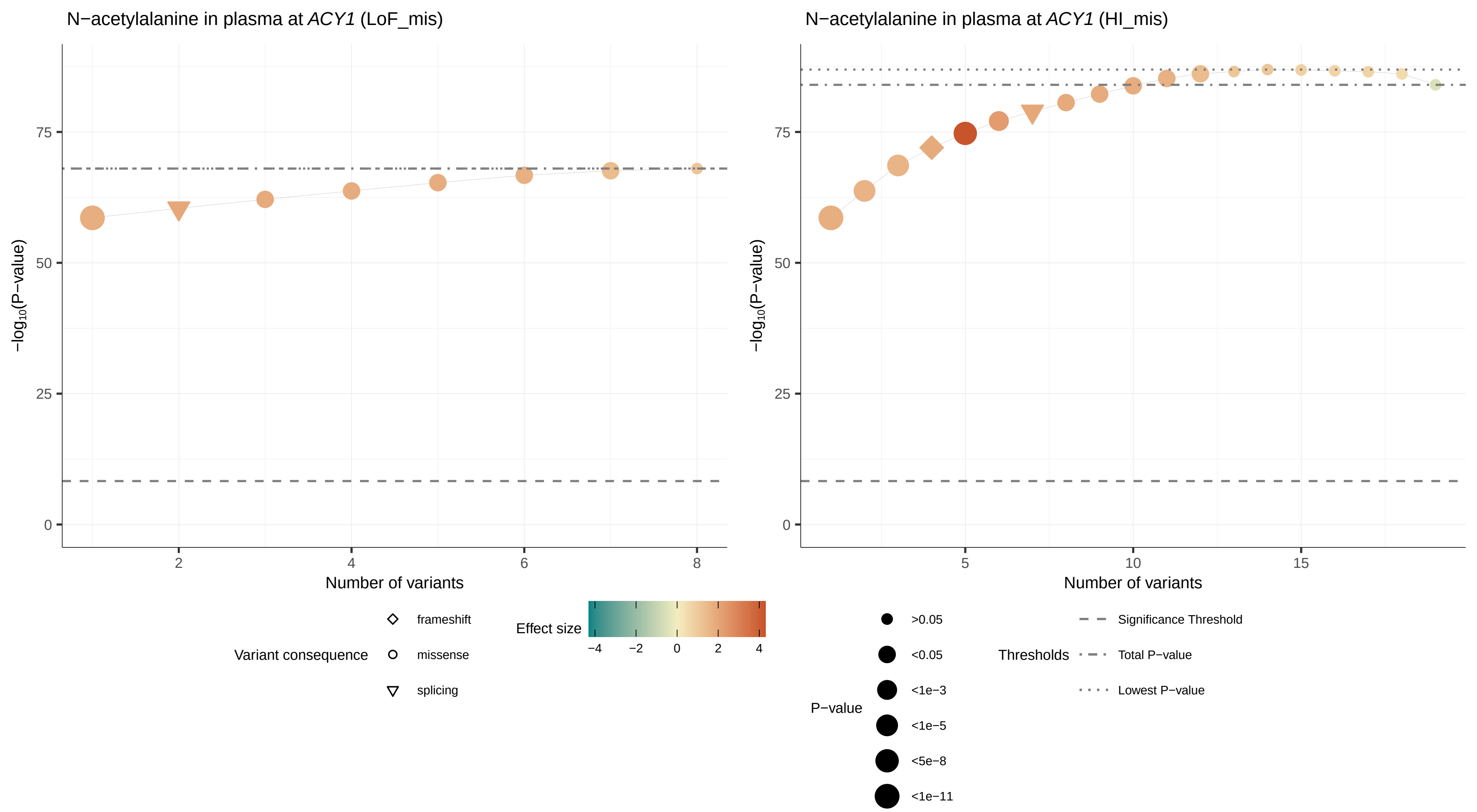
N−acetylalanine in plasma at *ACY1* (LoF_mis)
N−acetylalanine in plasma at *ACY1* (HI_mis)
−log10(P−value)
Number of variants
Variant consequence
frameshift
missense
splicing
Effect size
−4
−2
0
2
4
P−value
>0.05
<0.05
<1e−3
<1e−5
<5e−8
<1e−11
Thresholds
Significance Threshold
Total P−value
Lowest P−value

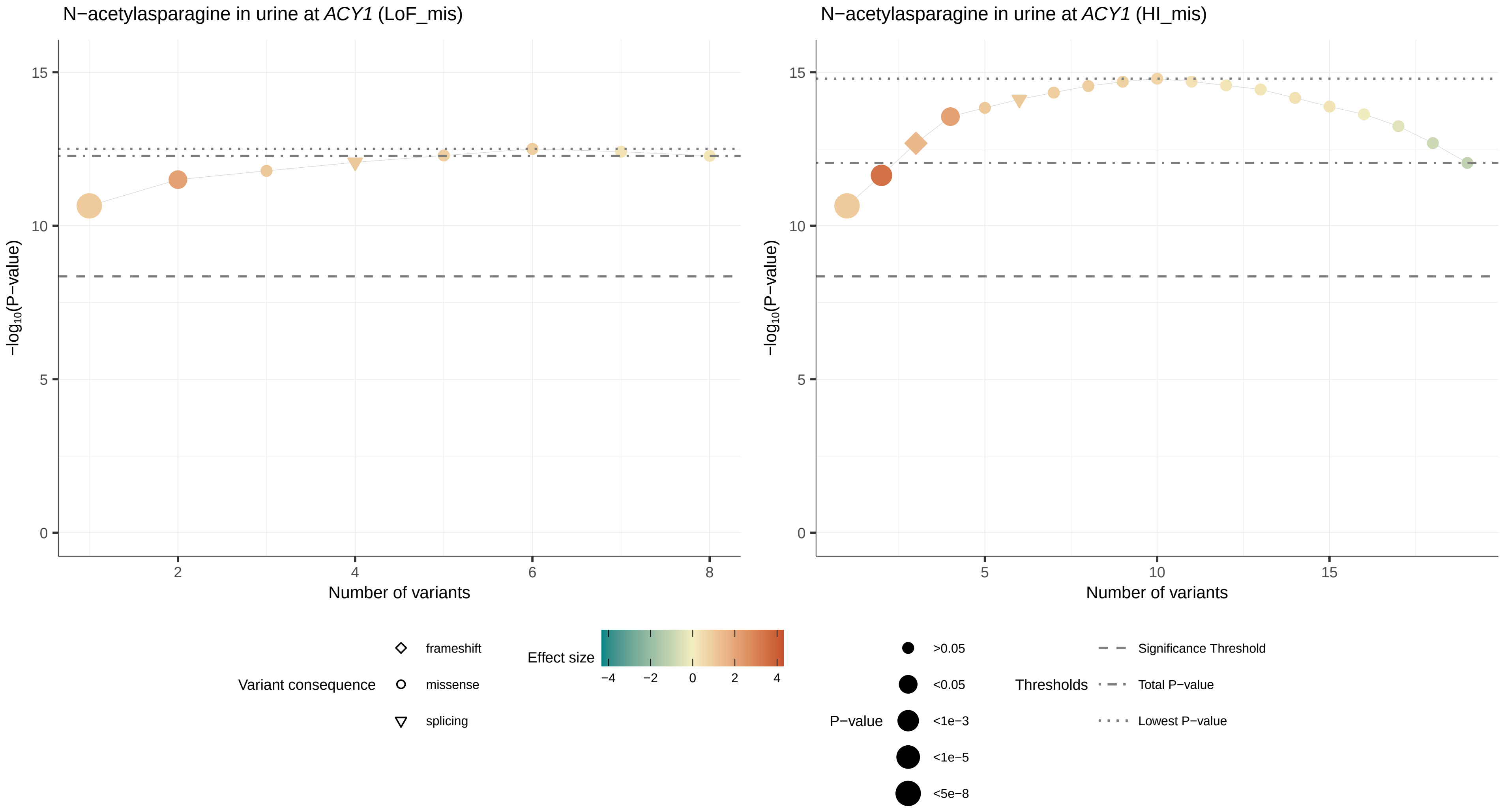

N−acetylasparagine in urine at ACY1 (LoF_mis)
N−acetylasparagine in urine at ACY1 (HI_mis)
−log10(P−value)
Number of variants
Variant consequence
frameshift
missense
splicing
Effect size
−4
−2
0
2
4
P−value
>0.05
<0.05
<1e−3
<1e−5
<5e−8
Thresholds
Significance Threshold
Total P−value
Lowest P−value

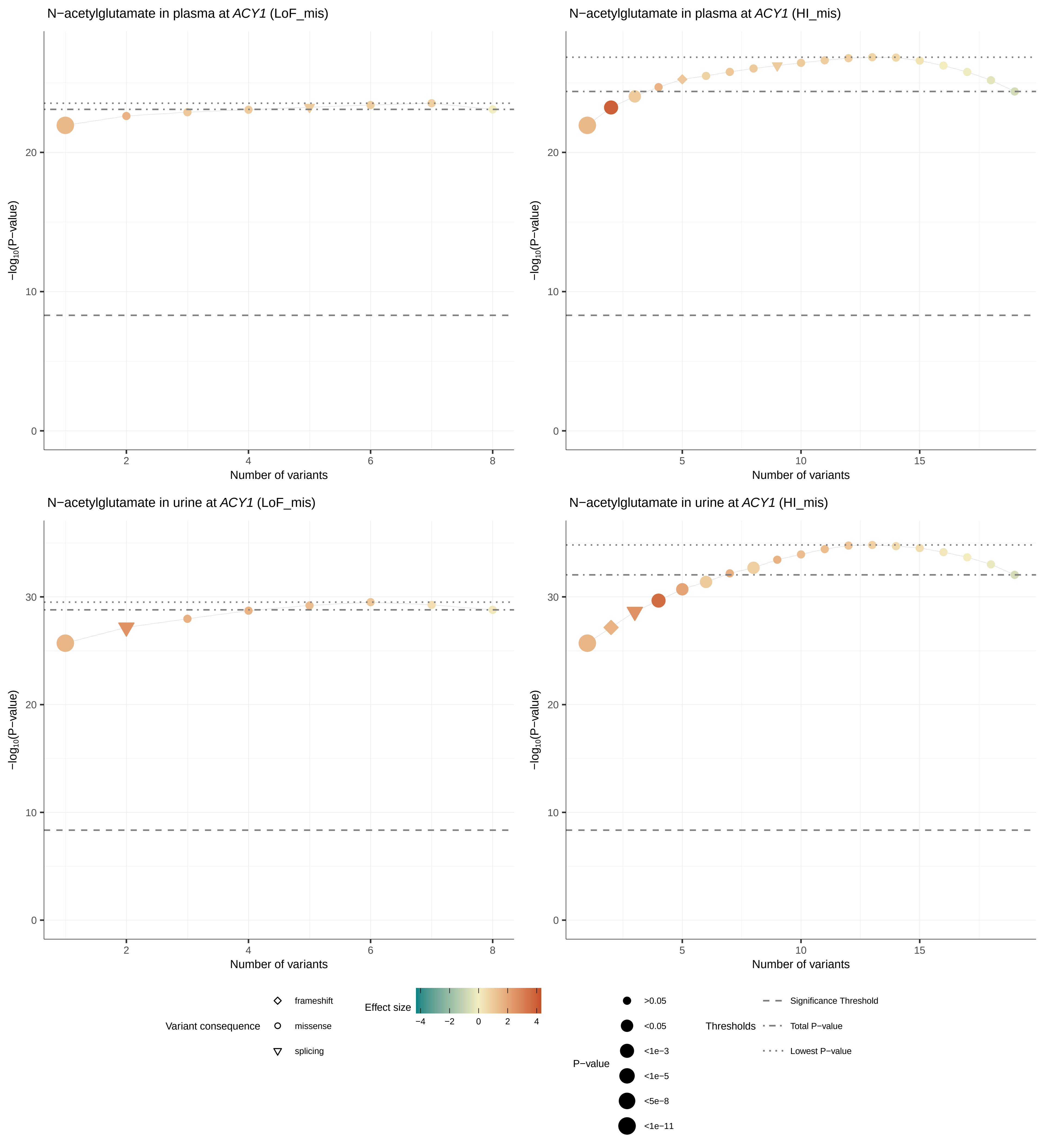

N−acetylglutamate in plasma at ACY1 (LoF_mis)
N−acetylglutamate in plasma at ACY1 (HI_mis)
N−acetylglutamate in urine at ACY1 (LoF_mis)
N−acetylglutamate in urine at ACY1 (HI_mis)
−log10(P−value)
Number of variants
Variant consequence
frameshift
missense
splicing
Effect size
−4
−2
0
2
4
P−value
>0.05
<0.05
<1e−3
<1e−5
<5e−8
<1e−11
Thresholds
Significance Threshold
Total P−value
Lowest P−value

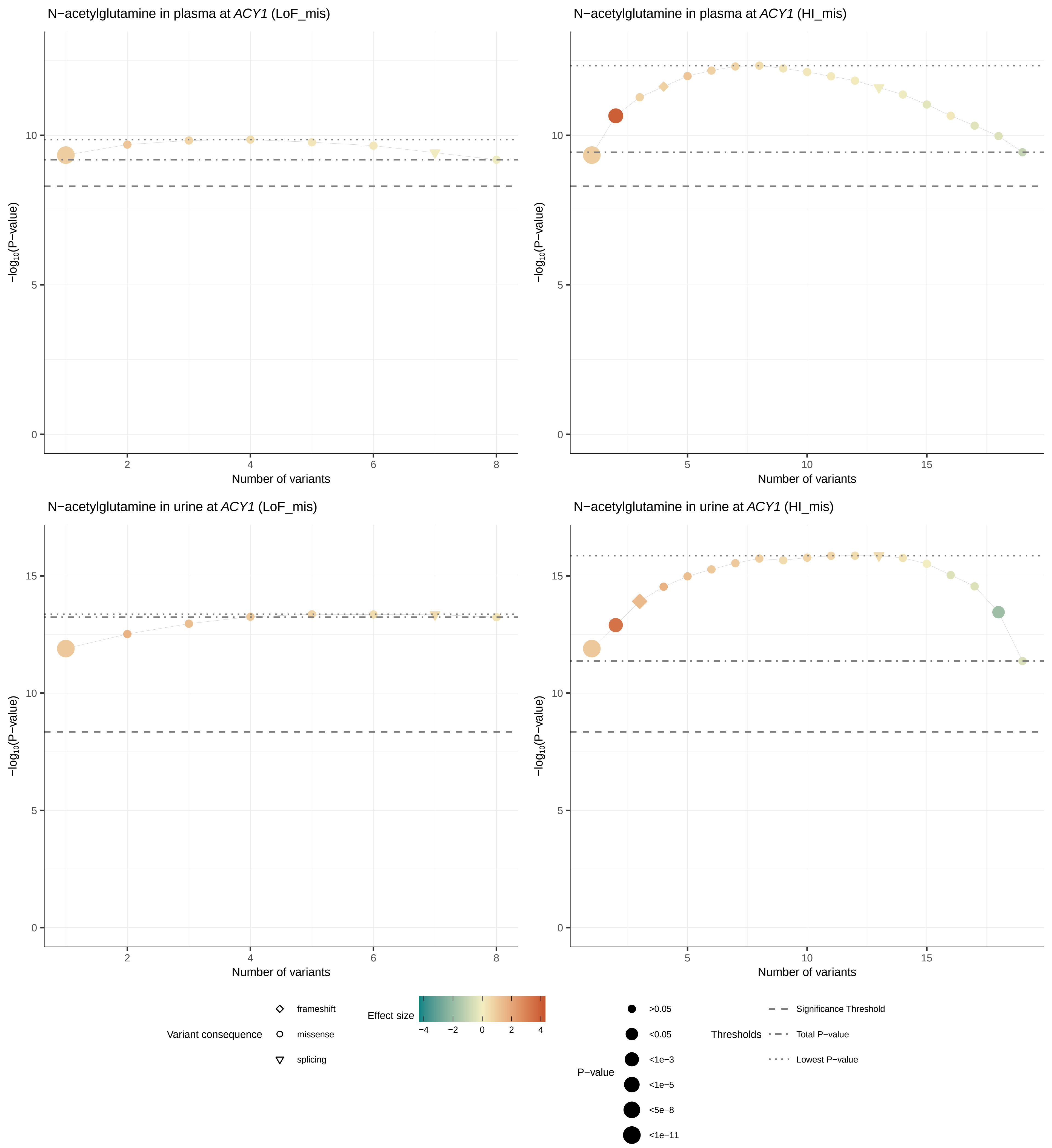

N−acetylglutamine in plasma at ACY1 (LoF_mis)
N−acetylglutamine in plasma at ACY1 (HI_mis)
N−acetylglutamine in urine at ACY1 (LoF_mis)
N−acetylglutamine in urine at ACY1 (HI_mis)
−log10(P−value)
Number of variants
Variant consequence
frameshift
missense
splicing
Effect size
−4
−2
0
2
4
P−value
>0.05
<0.05
<1e−3
<1e−5
<5e−8
<1e−11
Thresholds
Significance Threshold
Total P−value
Lowest P−value

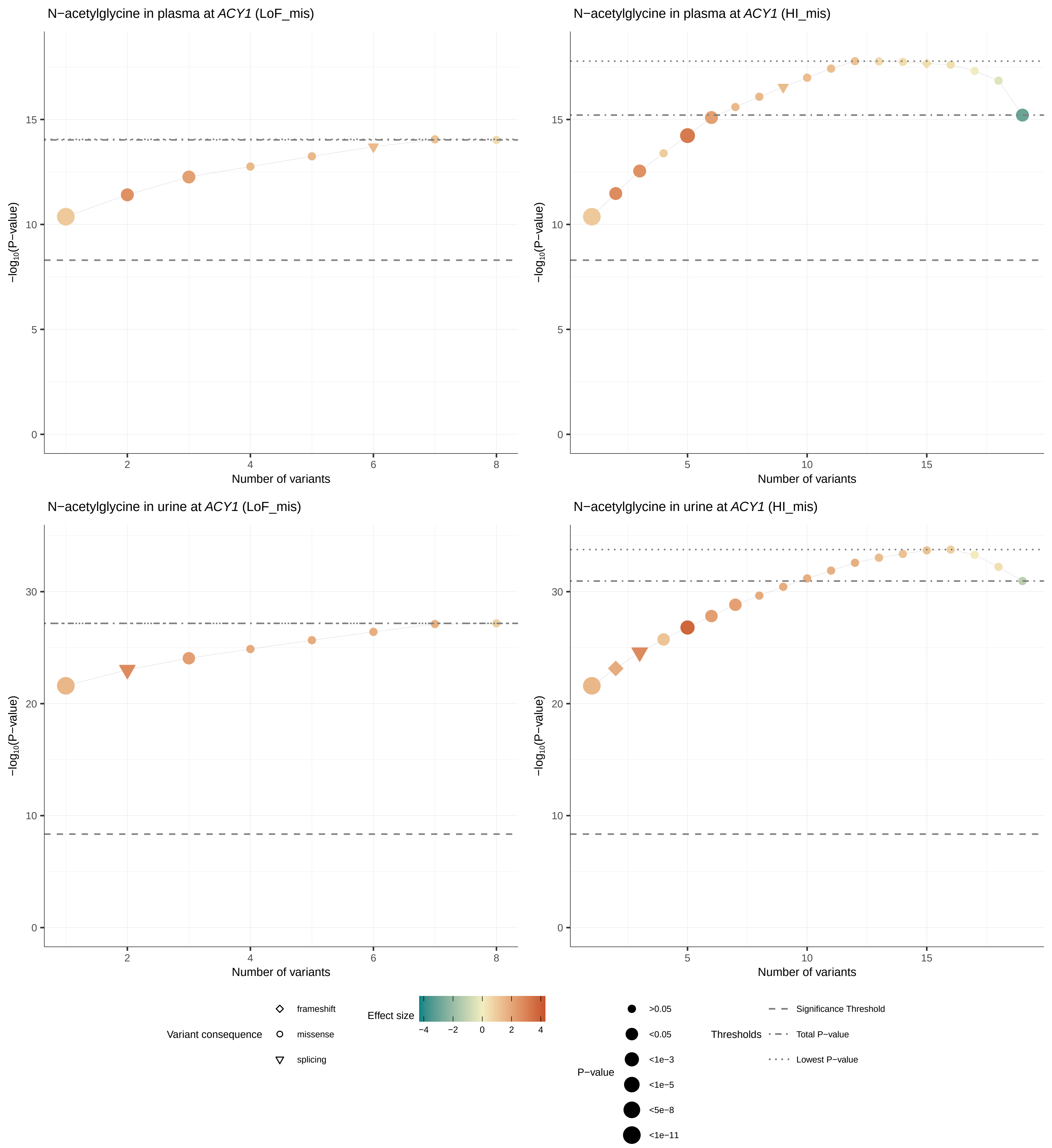

N−acetylglycine in plasma at ACY1 (LoF_mis)
N−acetylglycine in plasma at ACY1 (HI_mis)
N−acetylglycine in urine at ACY1 (LoF_mis)
N−acetylglycine in urine at ACY1 (HI_mis)
−log10(P−value)
Number of variants
Variant consequence
frameshift
missense
splicing
Effect size
−4
−2
0
2
4
P−value
>0.05
<0.05
<1e−3
<1e−5
<5e−8
<1e−11
Thresholds
Significance Threshold
Total P−value
Lowest P−value

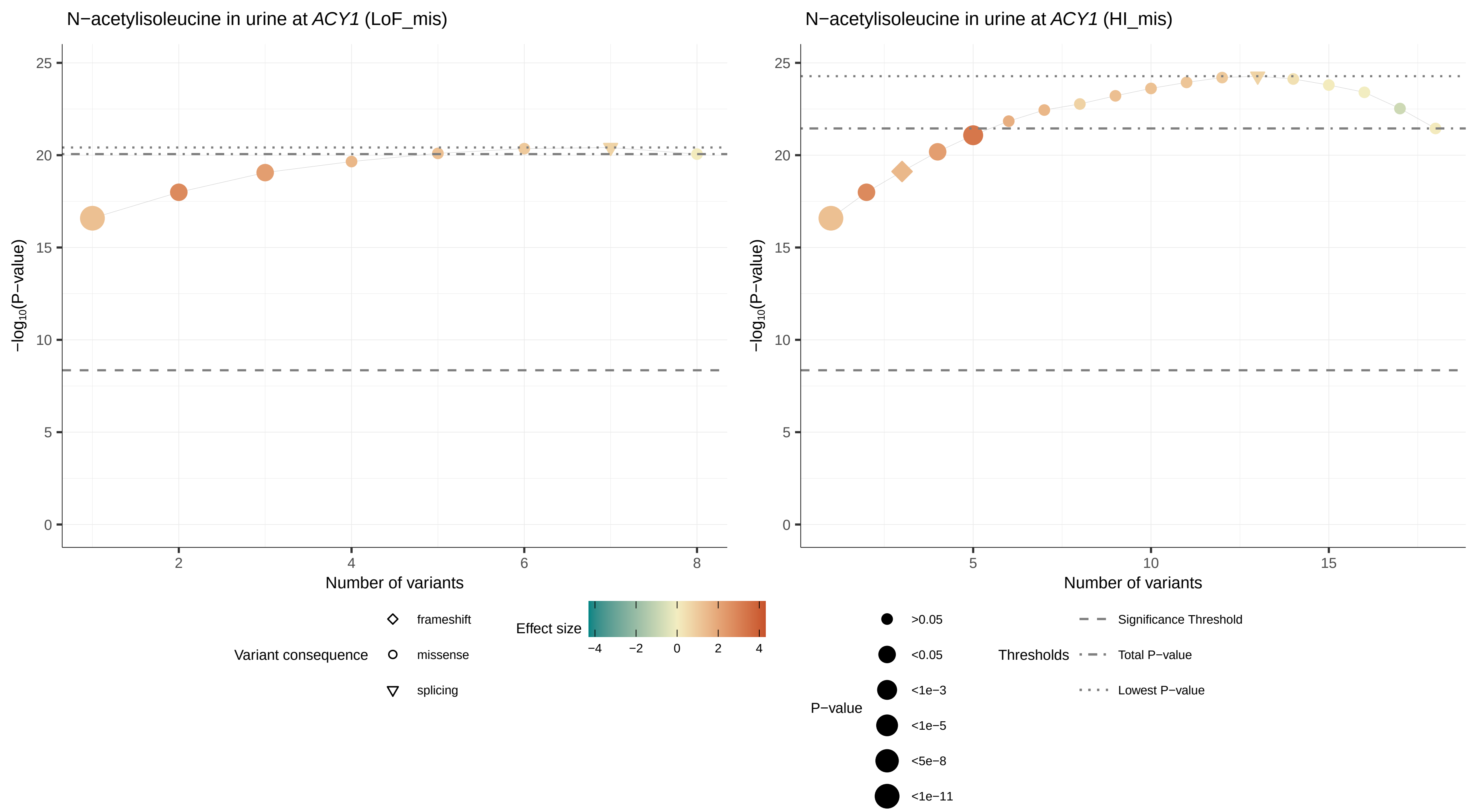
N−acetylisoleucine in urine at ACY1 (LoF_mis)
N−acetylisoleucine in urine at ACY1 (HI_mis)
−log10(P−value)
Number of variants
Variant consequence
frameshift
missense
splicing
Effect size
P−value
>0.05
<0.05
<1e−3
<1e−5
<5e−8
<1e−11
Thresholds
Significance Threshold
Total P−value
Lowest P−value

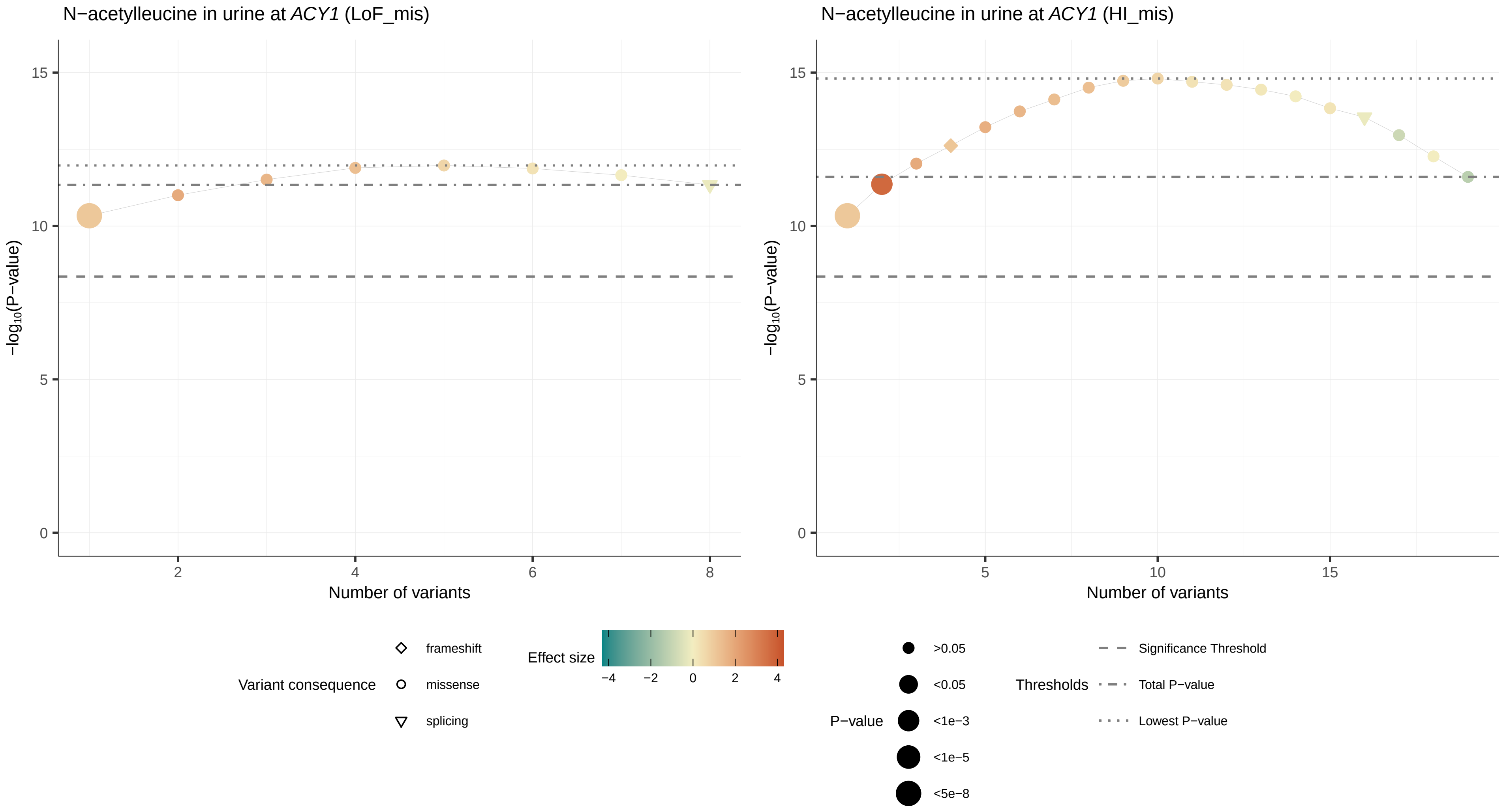

N−acetylleucine in urine at ACY1 (LoF_mis)
N−acetylleucine in urine at ACY1 (HI_mis)
−log10(P−value)
Number of variants
0
5
10
15
2
4
6
8
5
10
15
Variant consequence
frameshift
missense
splicing
Effect size
−4
−2
0
2
4
P−value
>0.05
<0.05
<1e−3
<1e−5
<5e−8
Thresholds
Significance Threshold
Total P−value
Lowest P−value

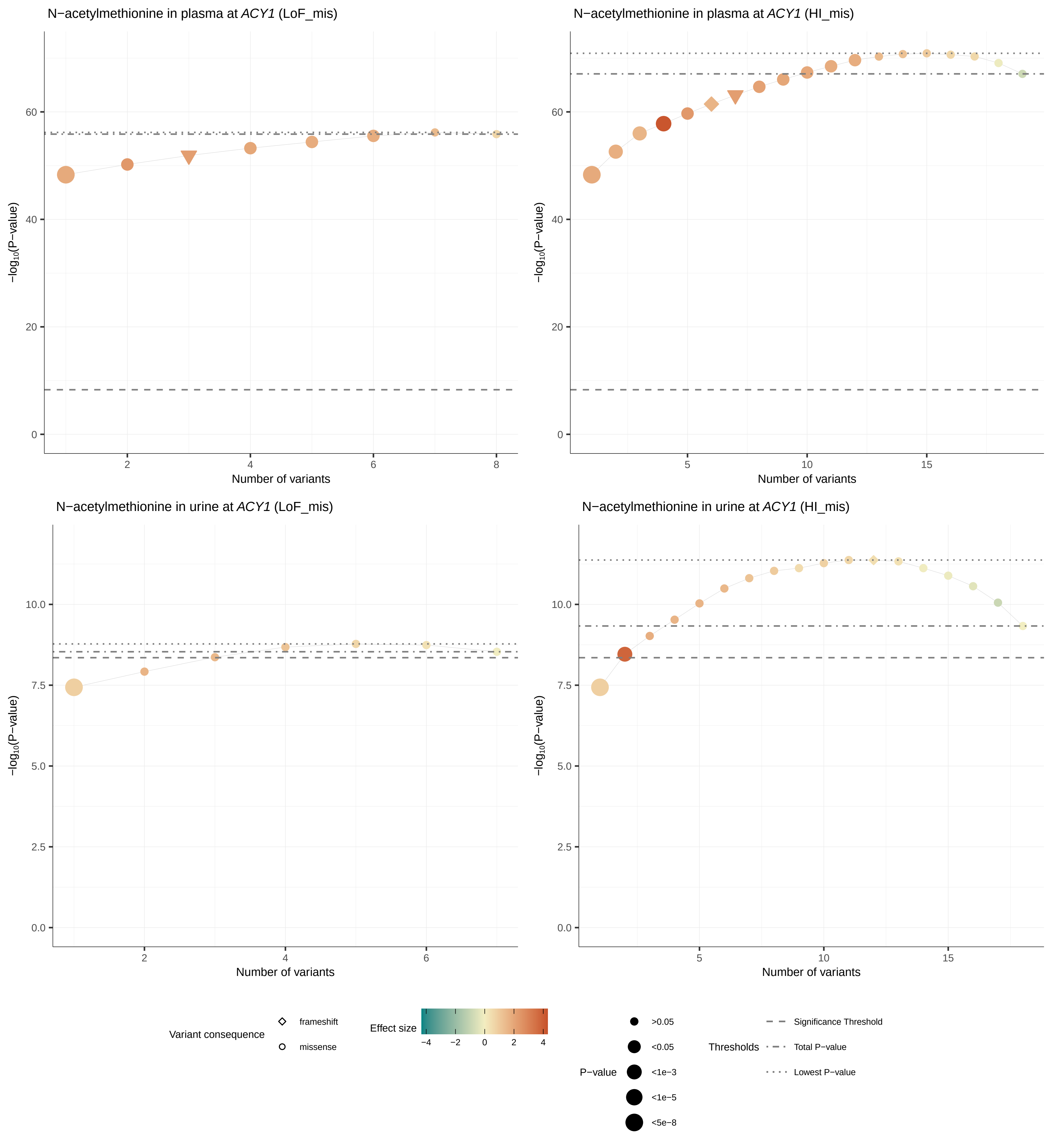
N−acetylmethionine in plasma at ACY1 (LoF_mis)
N−acetylmethionine in plasma at ACY1 (HI_mis)
N−acetylmethionine in urine at ACY1 (LoF_mis)
N−acetylmethionine in urine at ACY1 (HI_mis)
−log10(P−value)
Number of variants
Variant consequence
frameshift
missense
Effect size
−4
−2
0
2
4
P−value
>0.05
<0.05
<1e−3
<1e−5
<5e−8
Thresholds
Significance Threshold
Total P−value
Lowest P−value

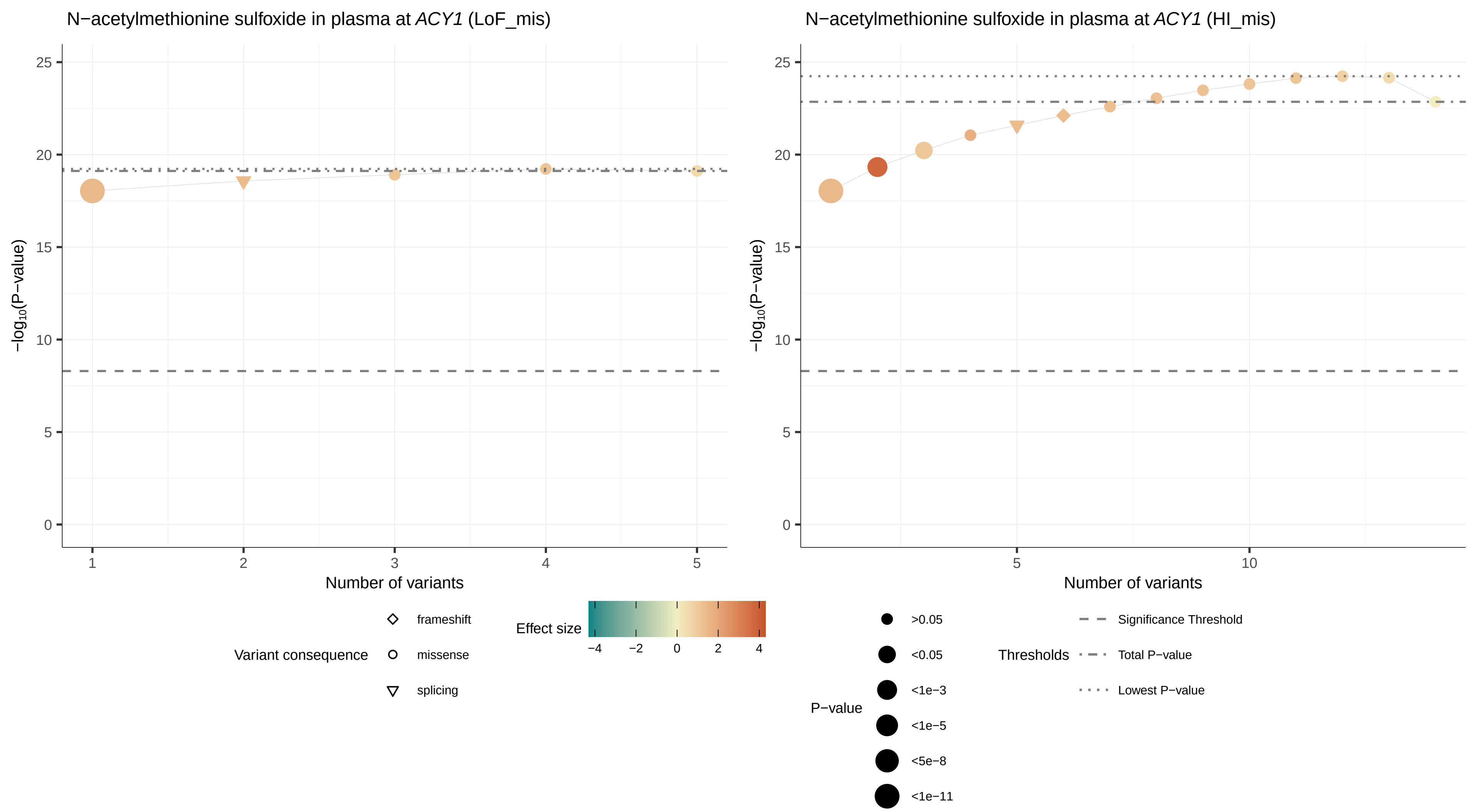

N−acetylmethionine sulfoxide in plasma at ACY1 (LoF_mis)
N−acetylmethionine sulfoxide in plasma at ACY1 (HI_mis)
−log10(P−value)
Number of variants
Variant consequence
frameshift
missense
splicing
Effect size
−4
−2
0
2
4
P−value
>0.05
<0.05
<1e−3
<1e−5
<5e−8
<1e−11
Thresholds
Significance Threshold
Total P−value
Lowest P−value

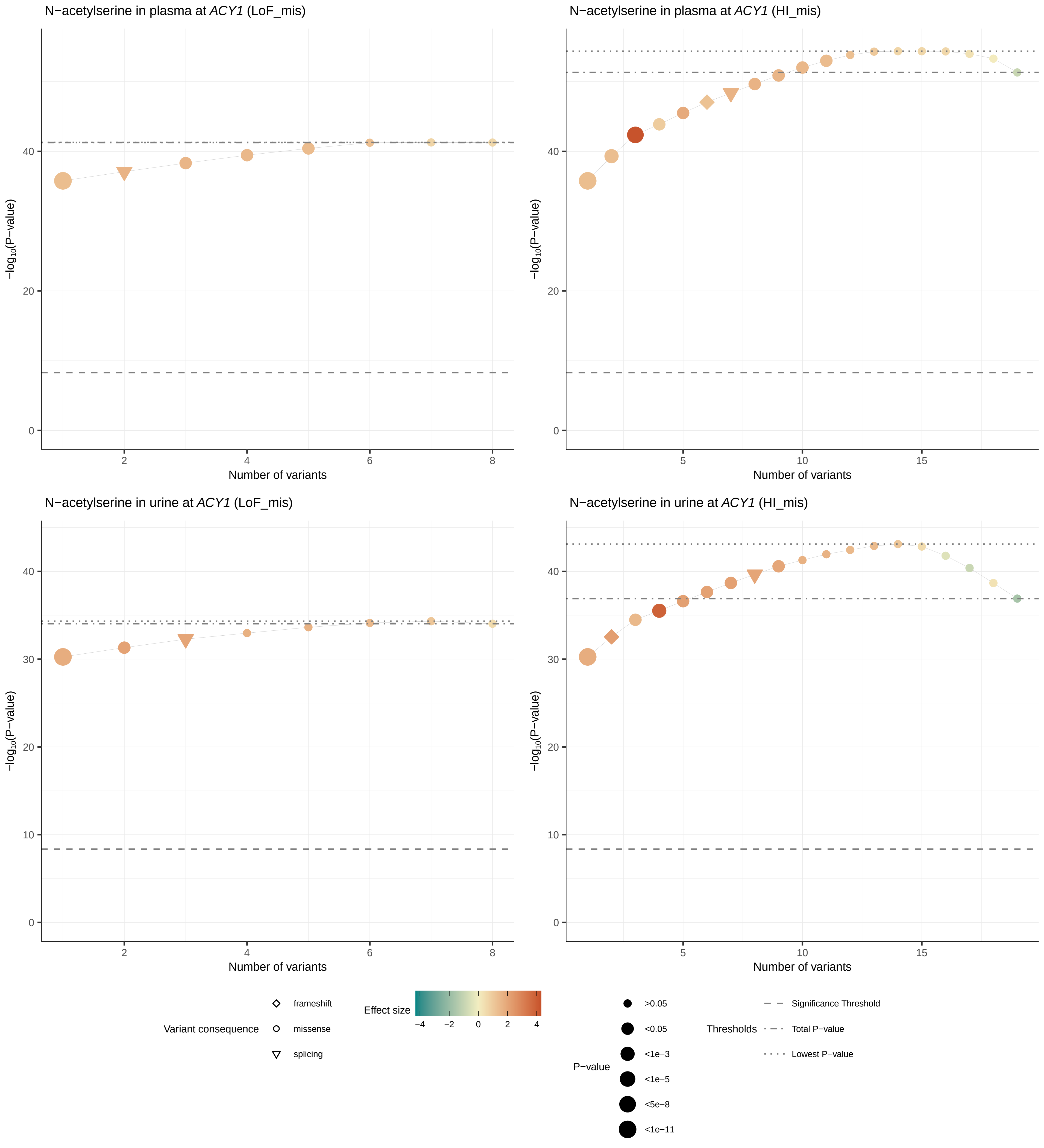

N−acetylserine in plasma at ACY1 (LoF_mis)
N−acetylserine in plasma at ACY1 (HI_mis)
N−acetylserine in urine at ACY1 (LoF_mis)
N−acetylserine in urine at ACY1 (HI_mis)
−log10(P−value)
Number of variants
Variant consequence
frameshift
missense
splicing
Effect size
−4
−2
0
2
4
P−value
>0.05
<0.05
<1e−3
<1e−5
<5e−8
<1e−11
Thresholds
Significance Threshold
Total P−value
Lowest P−value

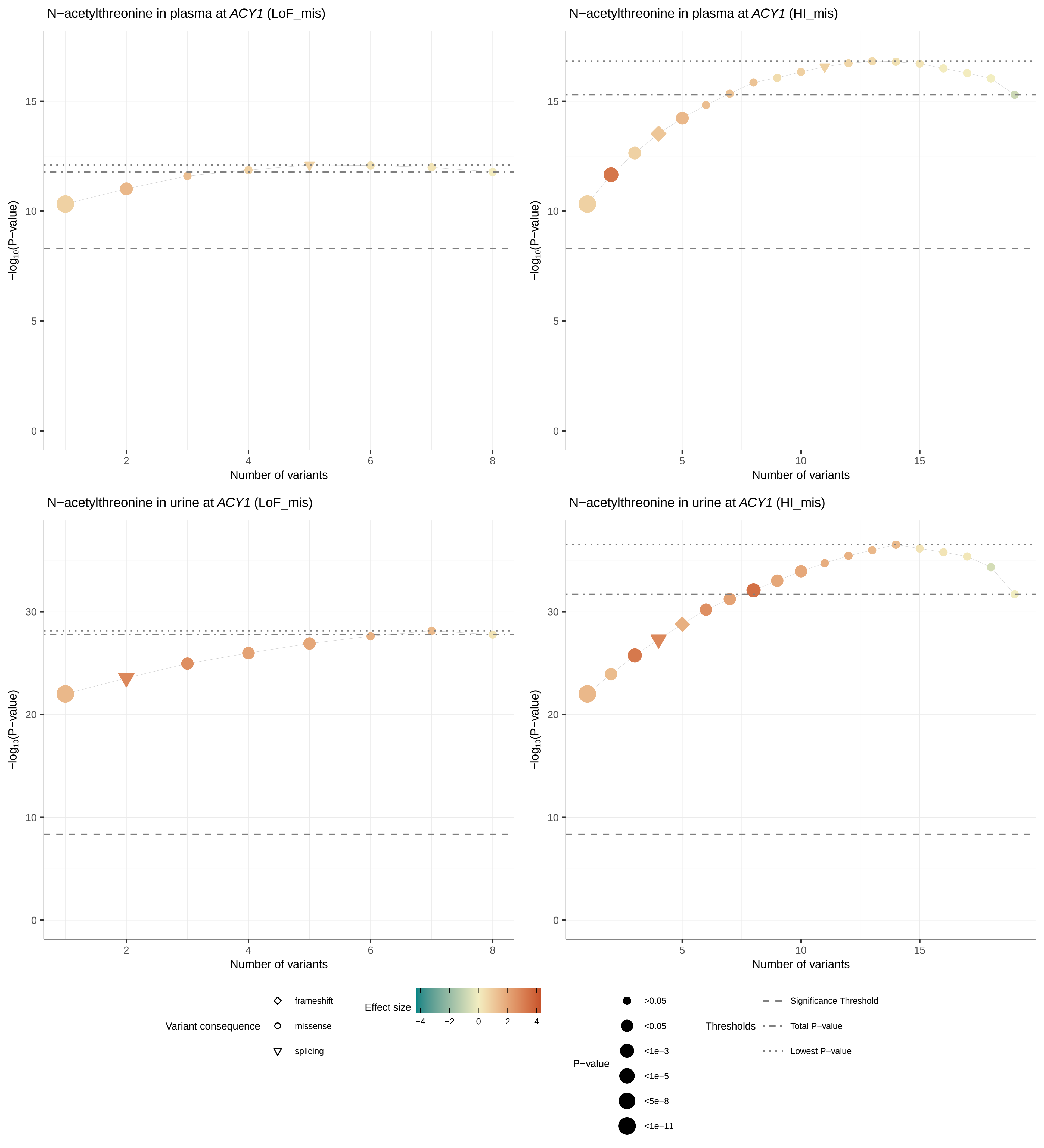

N−acetylthreonine in plasma at ACY1 (LoF_mis)
N−acetylthreonine in plasma at ACY1 (HI_mis)
N−acetylthreonine in urine at ACY1 (LoF_mis)
N−acetylthreonine in urine at ACY1 (HI_mis)
−log10(P−value)
Number of variants
Variant consequence
frameshift
missense
splicing
Effect size
−4
−2
0
2
4
P−value
>0.05
<0.05
<1e−3
<1e−5
<5e−8
<1e−11
Thresholds
Significance Threshold
Total P−value
Lowest P−value

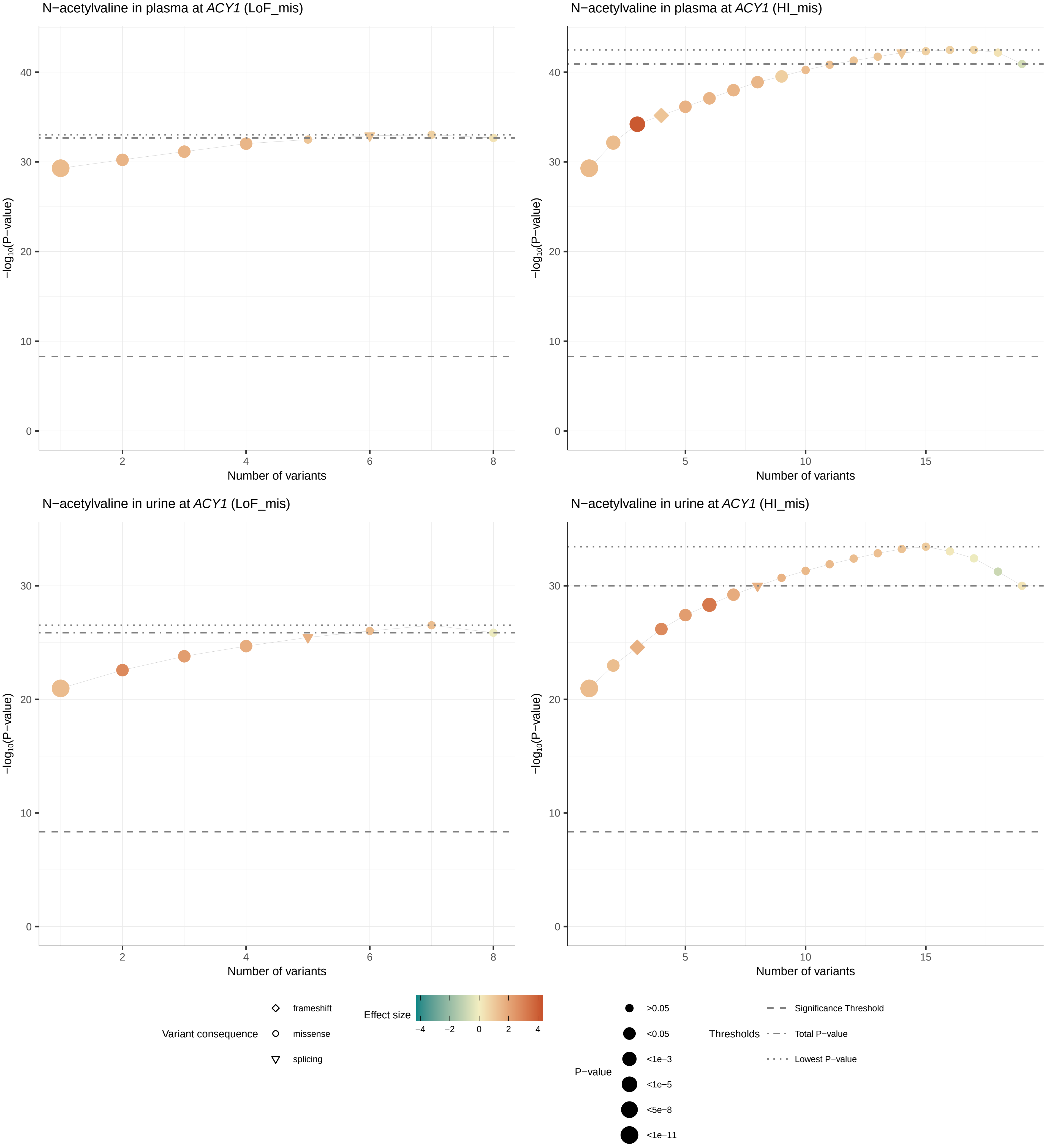

N−acetylvaline in plasma at ACY1 (LoF_mis)
N−acetylvaline in plasma at ACY1 (HI_mis)
N−acetylvaline in urine at ACY1 (LoF_mis)
N−acetylvaline in urine at ACY1 (HI_mis)
−log10(P−value)
Number of variants
Variant consequence
frameshift
missense
splicing
Effect size
−4
−2
0
2
4
P−value
>0.05
<0.05
<1e−3
<1e−5
<5e−8
<1e−11
Thresholds
Significance Threshold
Total P−value
Lowest P−value

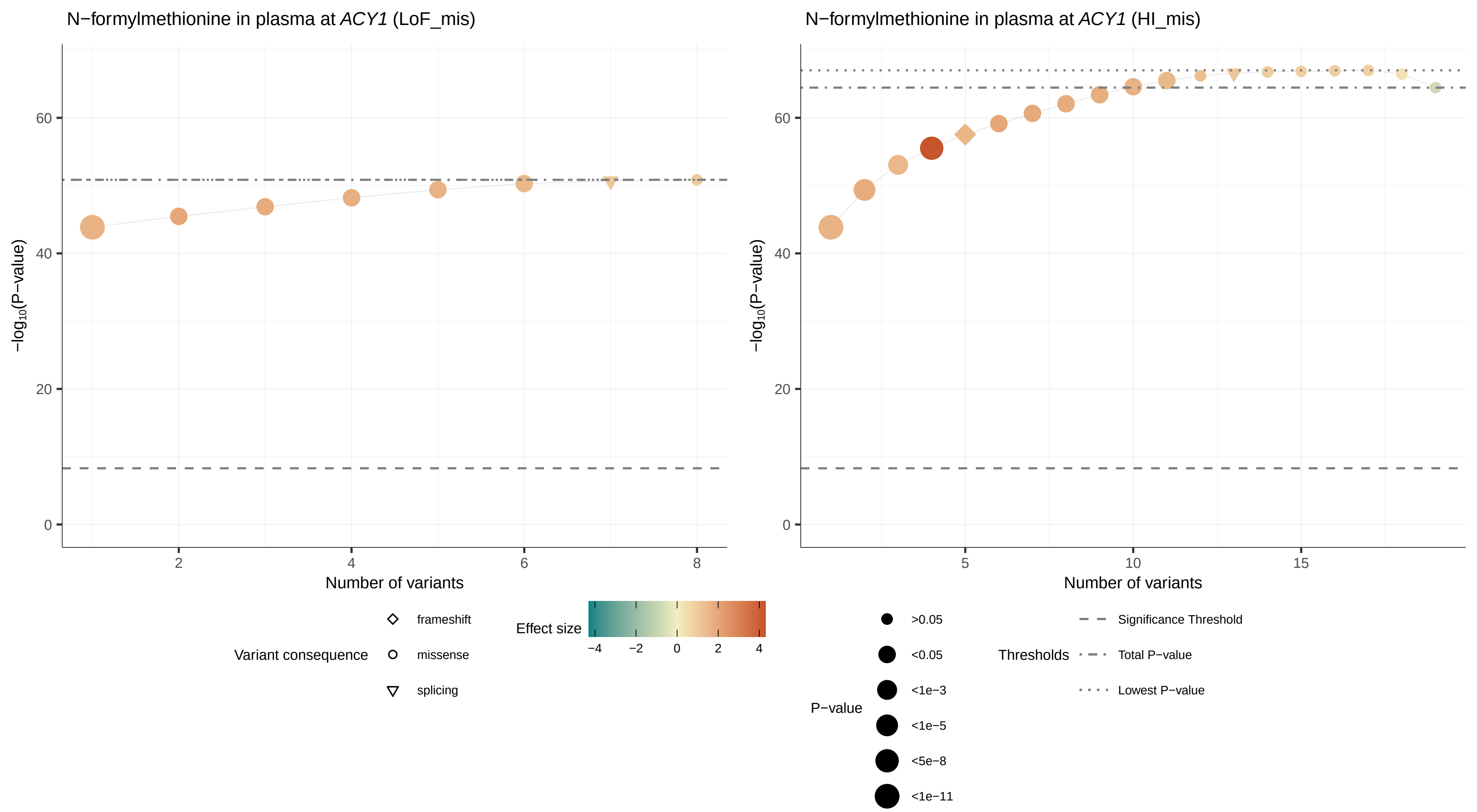

N−formylmethionine in plasma at ACY1 (LoF_mis)
N−formylmethionine in plasma at ACY1 (HI_mis)
−log10(P−value)
Number of variants
0
20
40
60
2
4
6
8
5
10
15
Variant consequence
frameshift
missense
splicing
Effect size
−4
−2
0
2
4
P−value
>0.05
<0.05
<1e−3
<1e−5
<5e−8
<1e−11
Thresholds
Significance Threshold
Total P−value
Lowest P−value

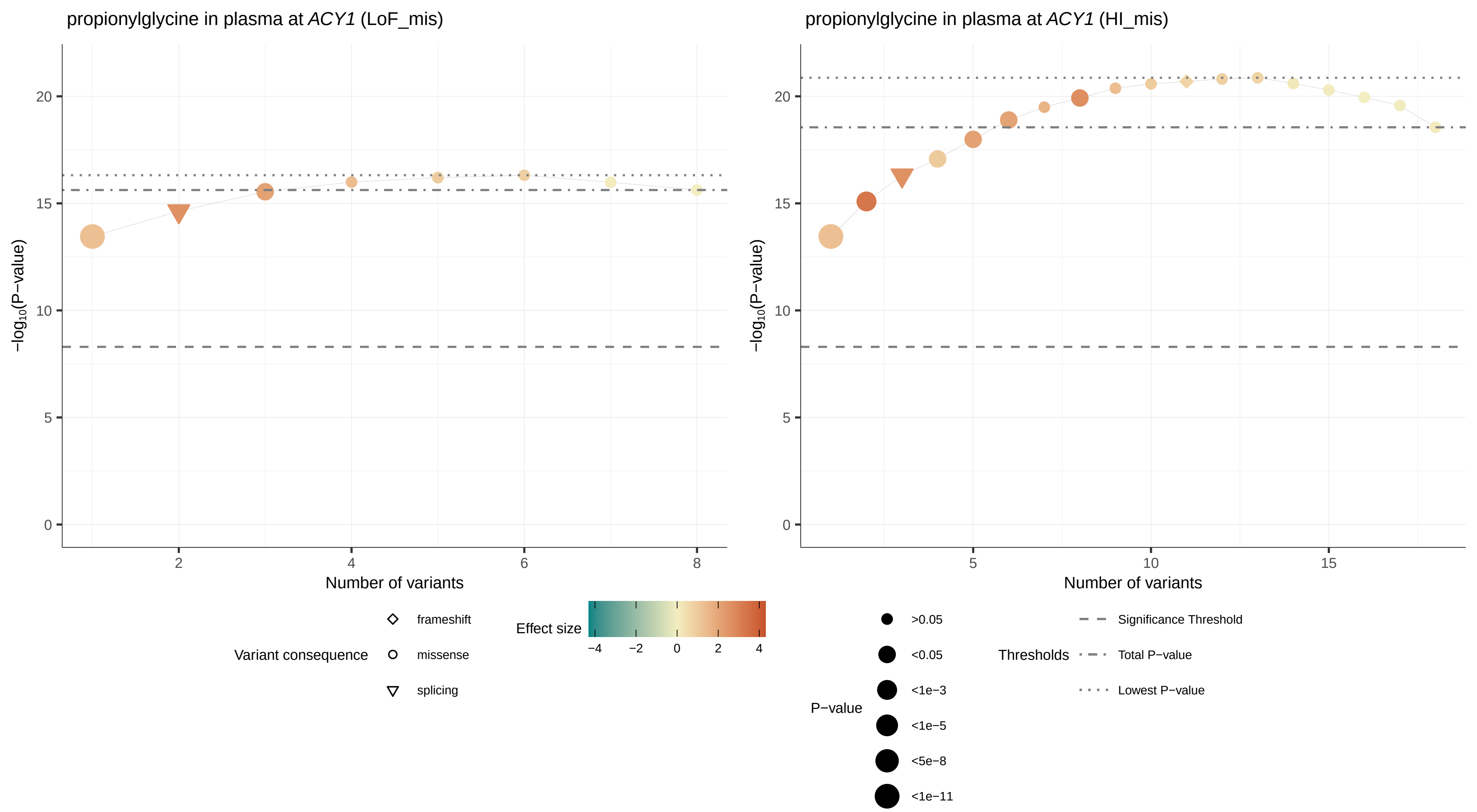

propionylglycine in plasma at *ACY1* (LoF_mis)
propionylglycine in plasma at *ACY1* (HI_mis)
$-\log_{10}$(P−value)
Number of variants
0
5
10
15
20
2
4
6
8
5
10
15
Variant consequence
frameshift
missense
splicing
Effect size
−4
−2
0
2
4
P−value
>0.05
<0.05
<1e−3
<1e−5
<5e−8
<1e−11
Thresholds
Significance Threshold
Total P−value
Lowest P−value

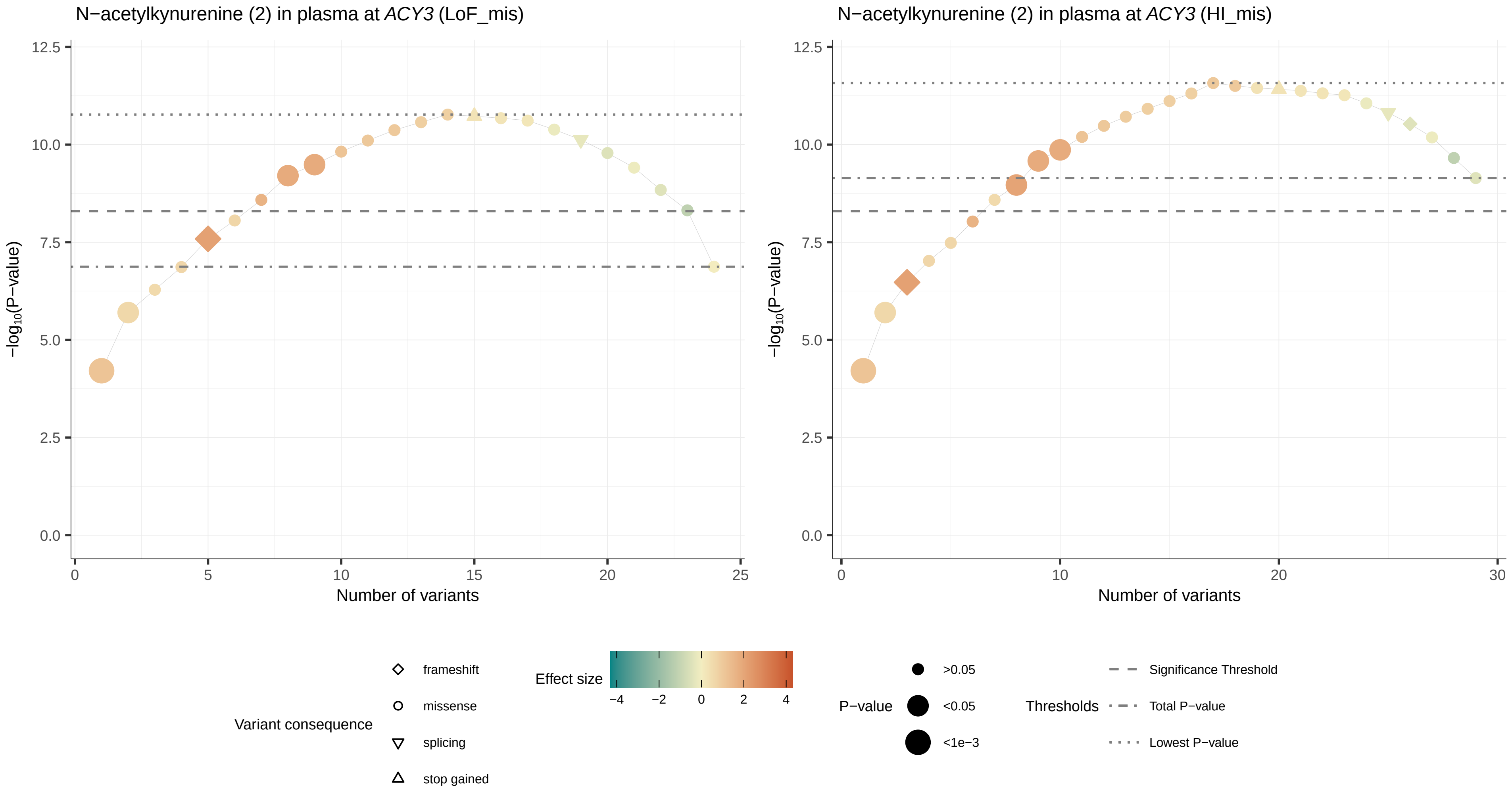
N−acetylkynurenine (2) in plasma at ACY3 (LoF_mis)
N−acetylkynurenine (2) in plasma at ACY3 (HI_mis)
−log10(P−value)
Number of variants
Variant consequence
frameshift
missense
splicing
stop gained
Effect size
P−value
>0.05
<0.05
<1e−3
Thresholds
Significance Threshold
Total P−value
Lowest P−value

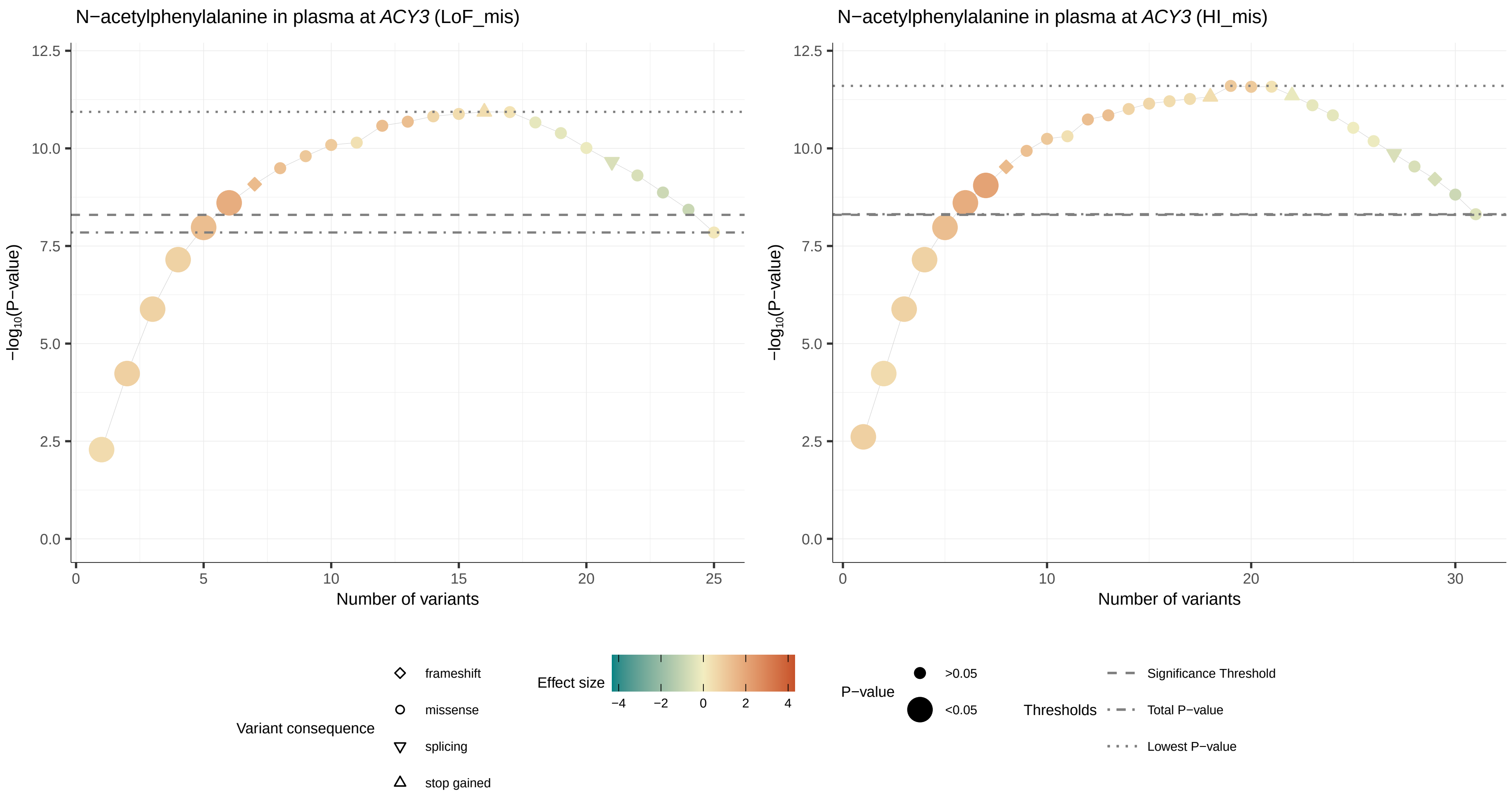
N−acetylphenylalanine in plasma at ACY3 (LoF_mis)
N−acetylphenylalanine in plasma at ACY3 (HI_mis)
−log10(P−value)
Number of variants
Variant consequence
frameshift
missense
splicing
stop gained
Effect size
P−value
>0.05
<0.05
Thresholds
Significance Threshold
Total P−value
Lowest P−value

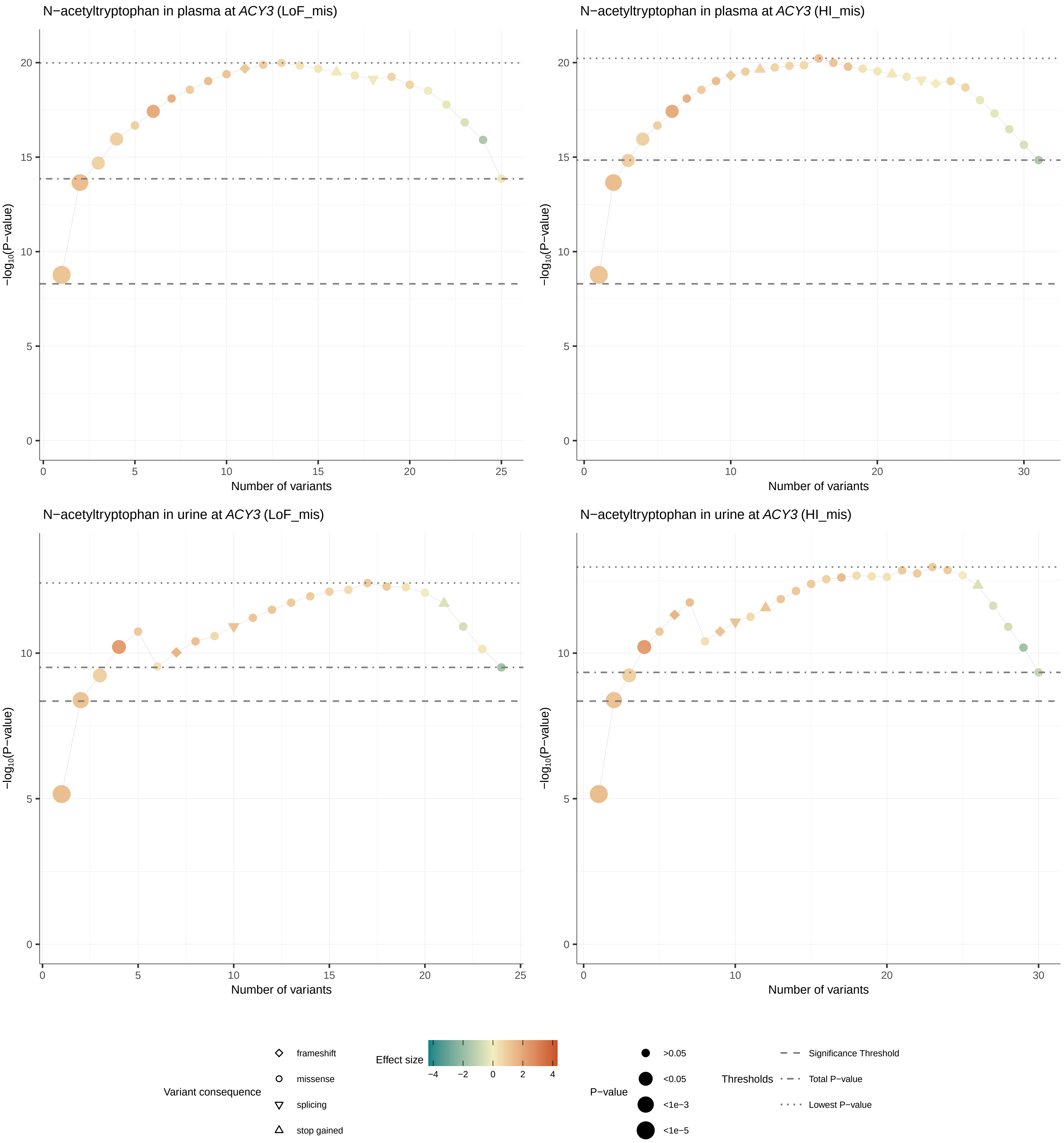
N−acetyltryptophan in plasma at ACY3 (LoF_mis)
N−acetyltryptophan in plasma at ACY3 (HI_mis)
N−acetyltryptophan in urine at ACY3 (LoF_mis)
N−acetyltryptophan in urine at ACY3 (HI_mis)
−log10(P−value)
Number of variants
Variant consequence
frameshift
missense
splicing
stop gained
Effect size
P−value
>0.05
<0.05
<1e−3
<1e−5
Thresholds
Significance Threshold
Total P−value
Lowest P−value

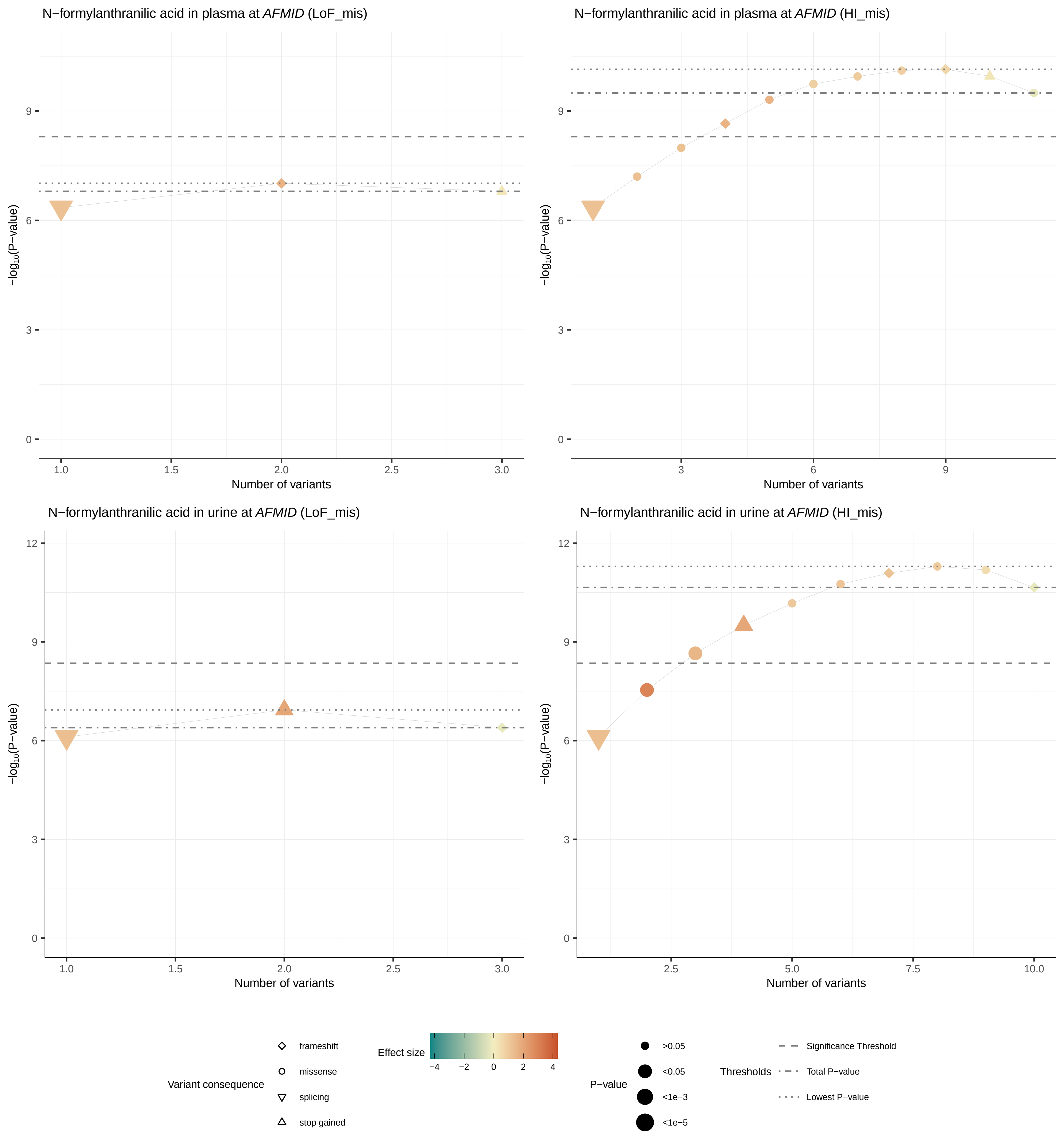

N−formylanthranilic acid in plasma at AFMID (LoF_mis)
N−formylanthranilic acid in plasma at AFMID (HI_mis)
N−formylanthranilic acid in urine at AFMID (LoF_mis)
N−formylanthranilic acid in urine at AFMID (HI_mis)
−log10(P−value)
Number of variants
Variant consequence
frameshift
missense
splicing
stop gained
Effect size
−4
−2
0
2
4
P−value
>0.05
<0.05
<1e−3
<1e−5
Thresholds
Significance Threshold
Total P−value
Lowest P−value

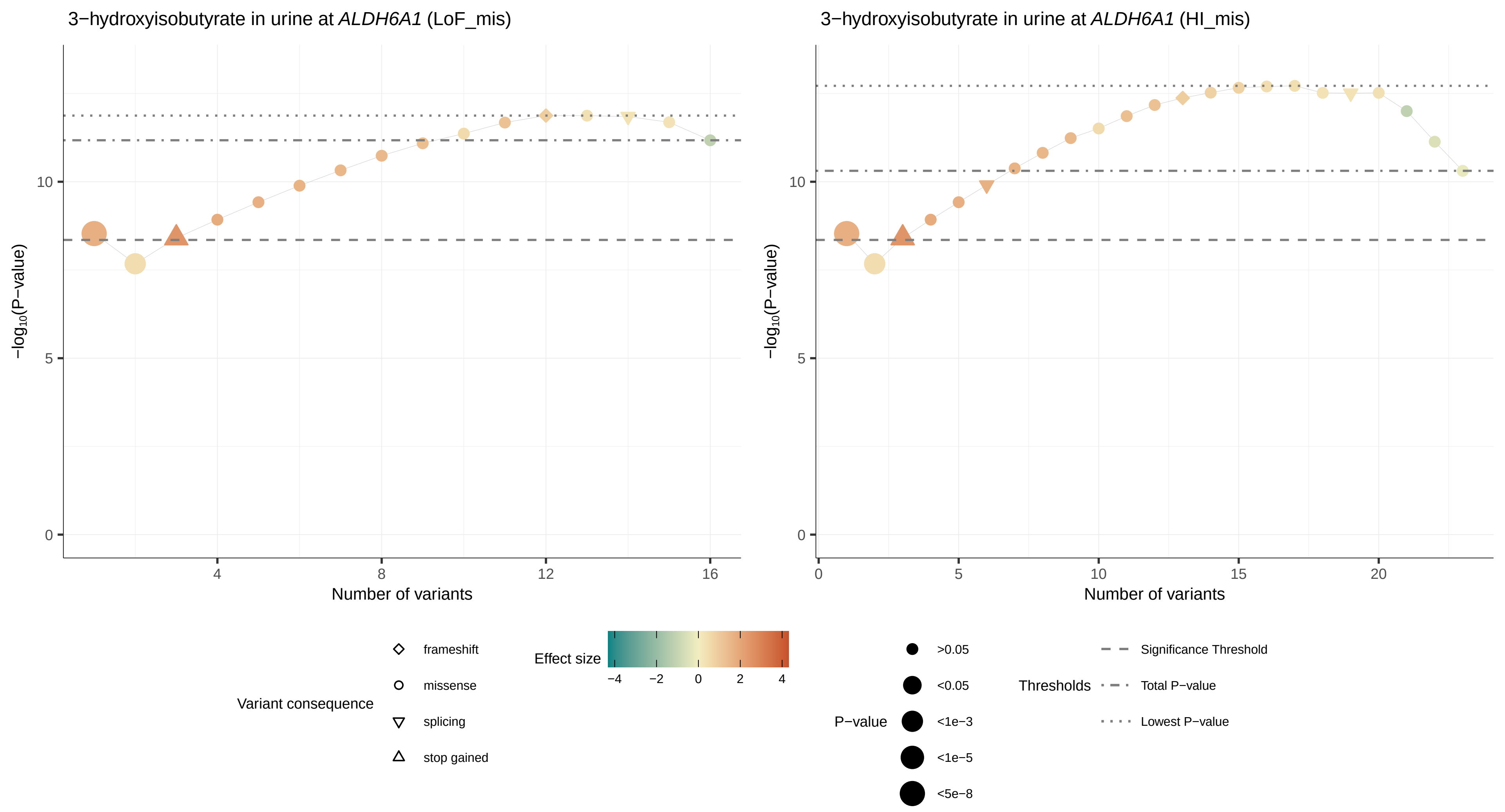

3−hydroxyisobutyrate in urine at ALDH6A1 (LoF_mis)
3−hydroxyisobutyrate in urine at ALDH6A1 (HI_mis)
−log10(P−value)
Number of variants
Variant consequence
frameshift
missense
splicing
stop gained
Effect size
P−value
>0.05
<0.05
<1e−3
<1e−5
<5e−8
Thresholds
Significance Threshold
Total P−value
Lowest P−value

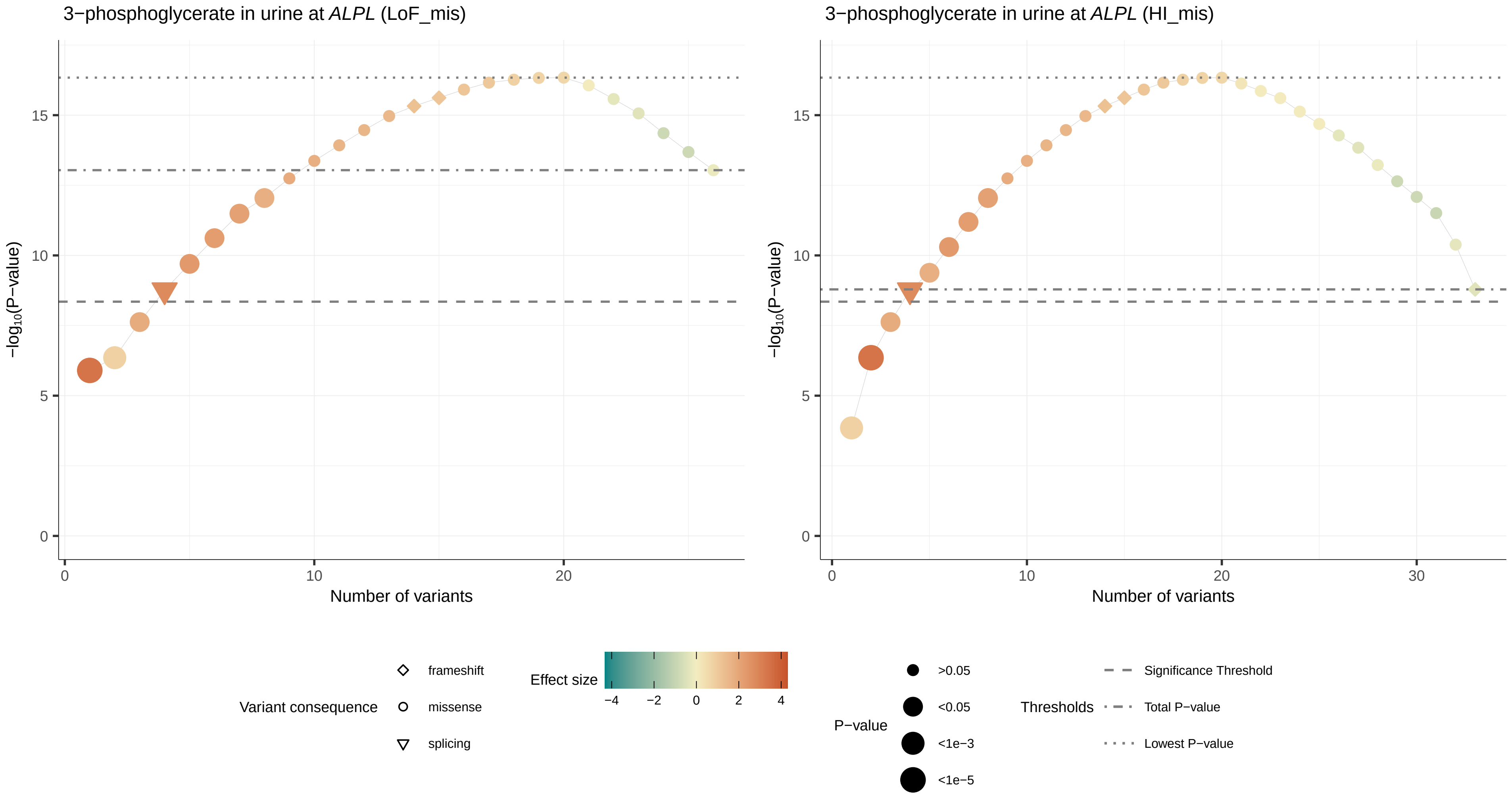
3−phosphoglycerate in urine at ALPL (LoF_mis)
3−phosphoglycerate in urine at ALPL (HI_mis)
−log10(P−value)
Number of variants
0
5
10
15
20
30
Variant consequence
frameshift
missense
splicing
Effect size
−4
−2
0
2
4
P−value
>0.05
<0.05
<1e−3
<1e−5
Thresholds
Significance Threshold
Total P−value
Lowest P−value

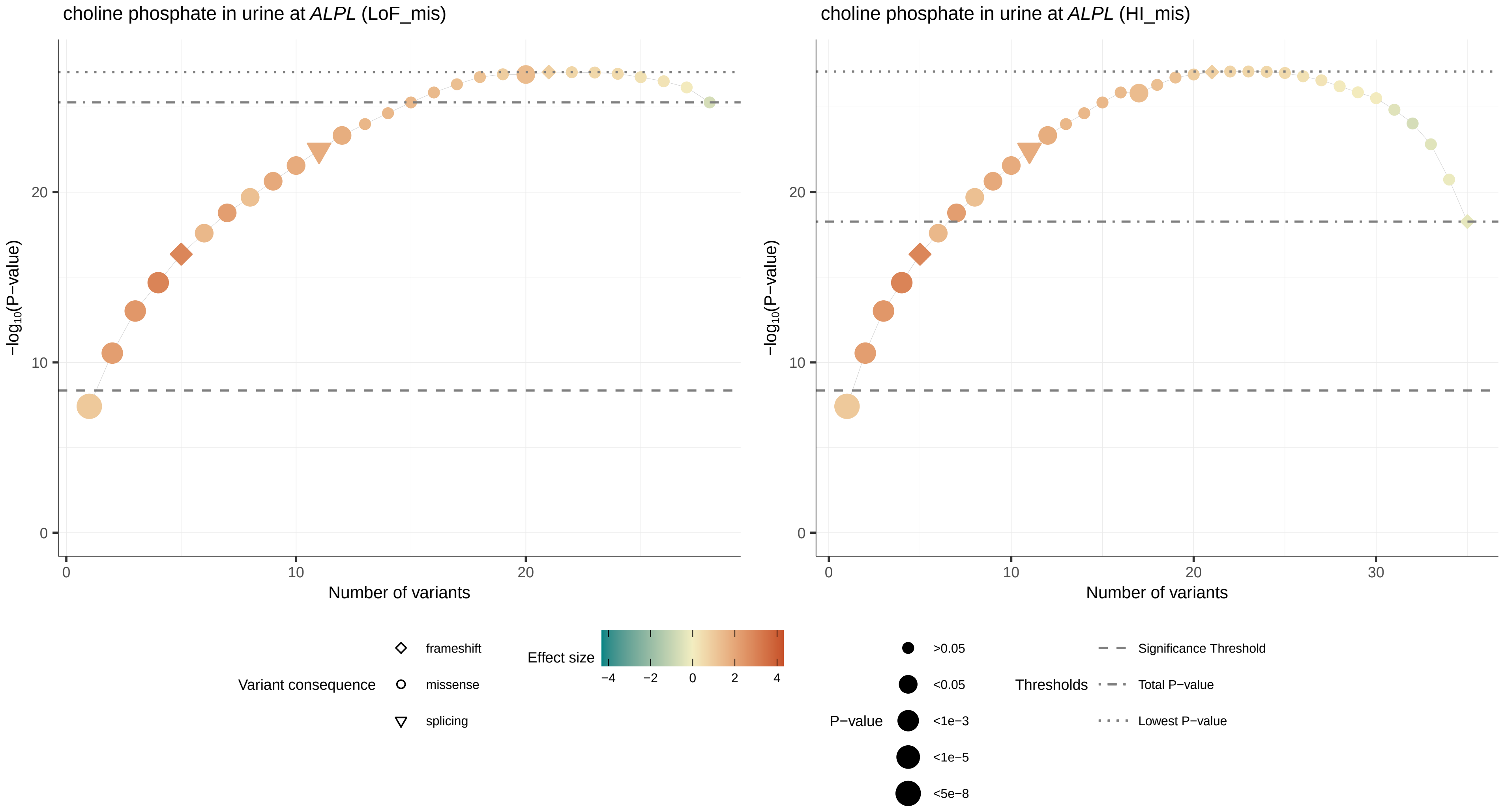
choline phosphate in urine at ALPL (LoF_mis)
choline phosphate in urine at ALPL (HI_mis)
−log10(P−value)
Number of variants
0
10
20
30
Variant consequence
frameshift
missense
splicing
Effect size
−4
−2
0
2
4
P−value
>0.05
<0.05
<1e−3
<1e−5
<5e−8
Thresholds
Significance Threshold
Total P−value
Lowest P−value

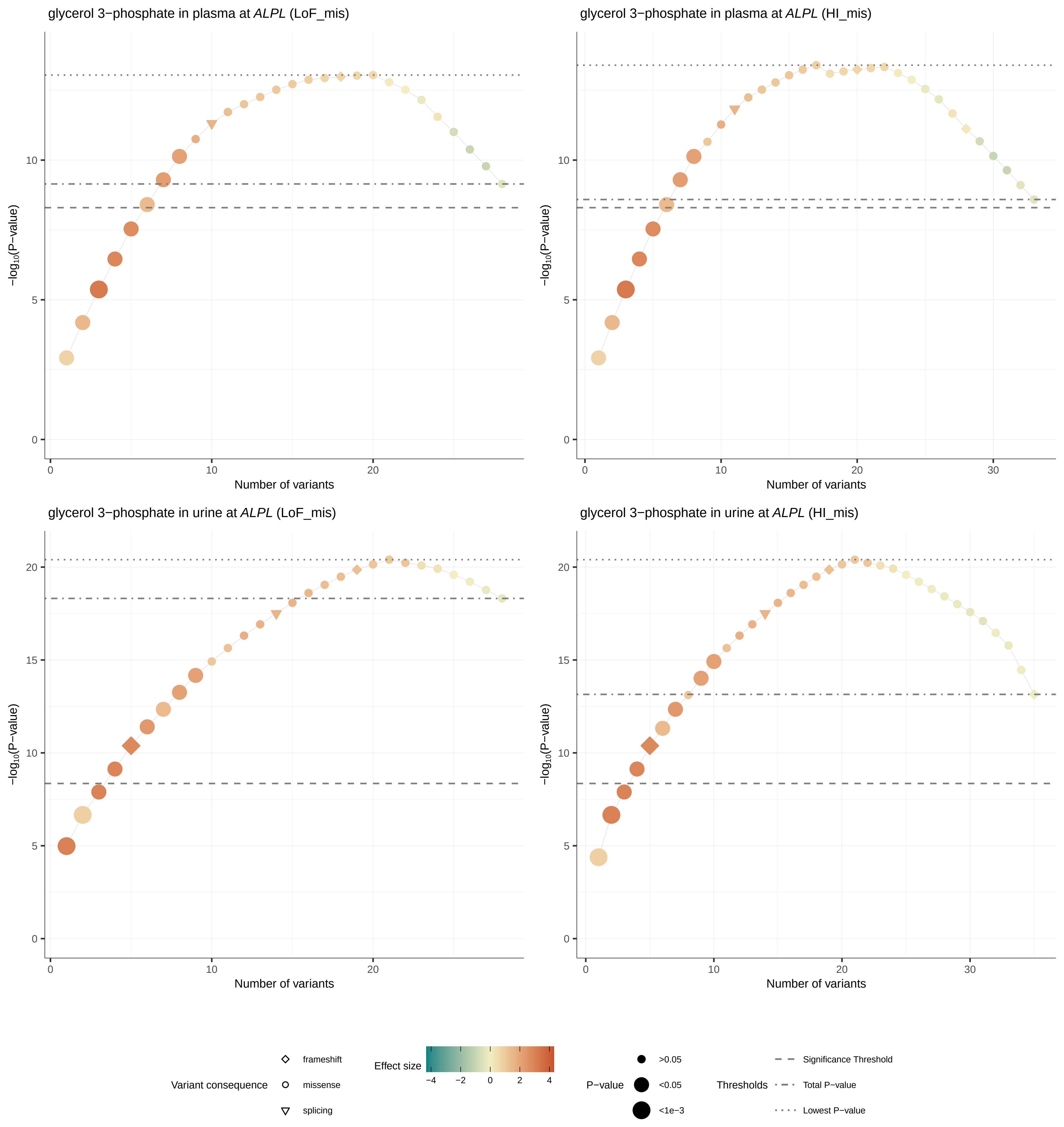

glycerol 3−phosphate in plasma at ALPL (LoF_mis)
glycerol 3−phosphate in plasma at ALPL (HI_mis)
glycerol 3−phosphate in urine at ALPL (LoF_mis)
glycerol 3−phosphate in urine at ALPL (HI_mis)
−log10(P−value)
Number of variants
Variant consequence
frameshift
missense
splicing
Effect size
−4
−2
0
2
4
P−value
>0.05
<0.05
<1e−3
Thresholds
Significance Threshold
Total P−value
Lowest P−value

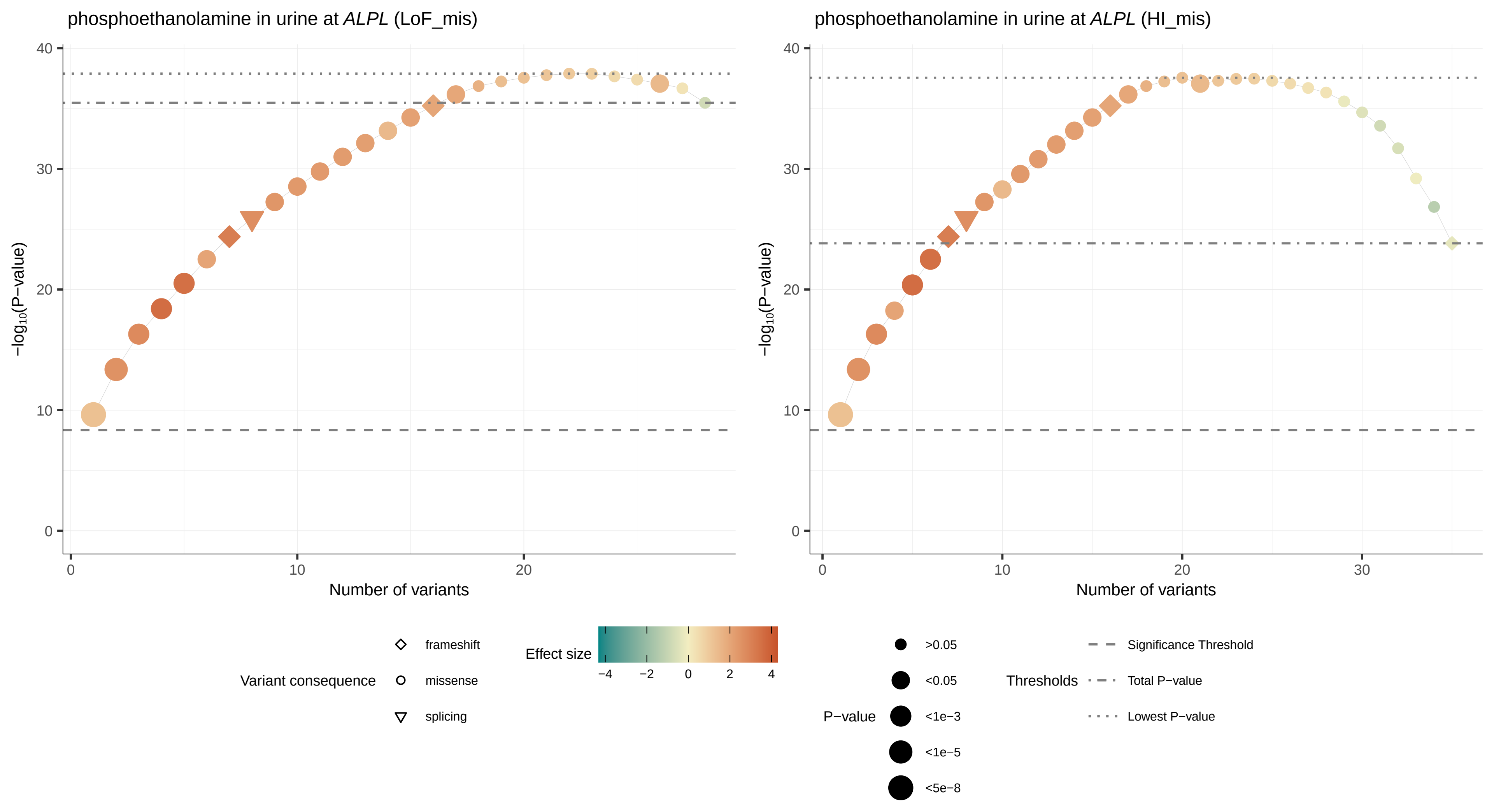

phosphoethanolamine in urine at ALPL (LoF_mis)
phosphoethanolamine in urine at ALPL (HI_mis)
−log10(P−value)
Number of variants
Variant consequence
frameshift
missense
splicing
Effect size
−4
−2
0
2
4
P−value
>0.05
<0.05
<1e−3
<1e−5
<5e−8
Thresholds
Significance Threshold
Total P−value
Lowest P−value

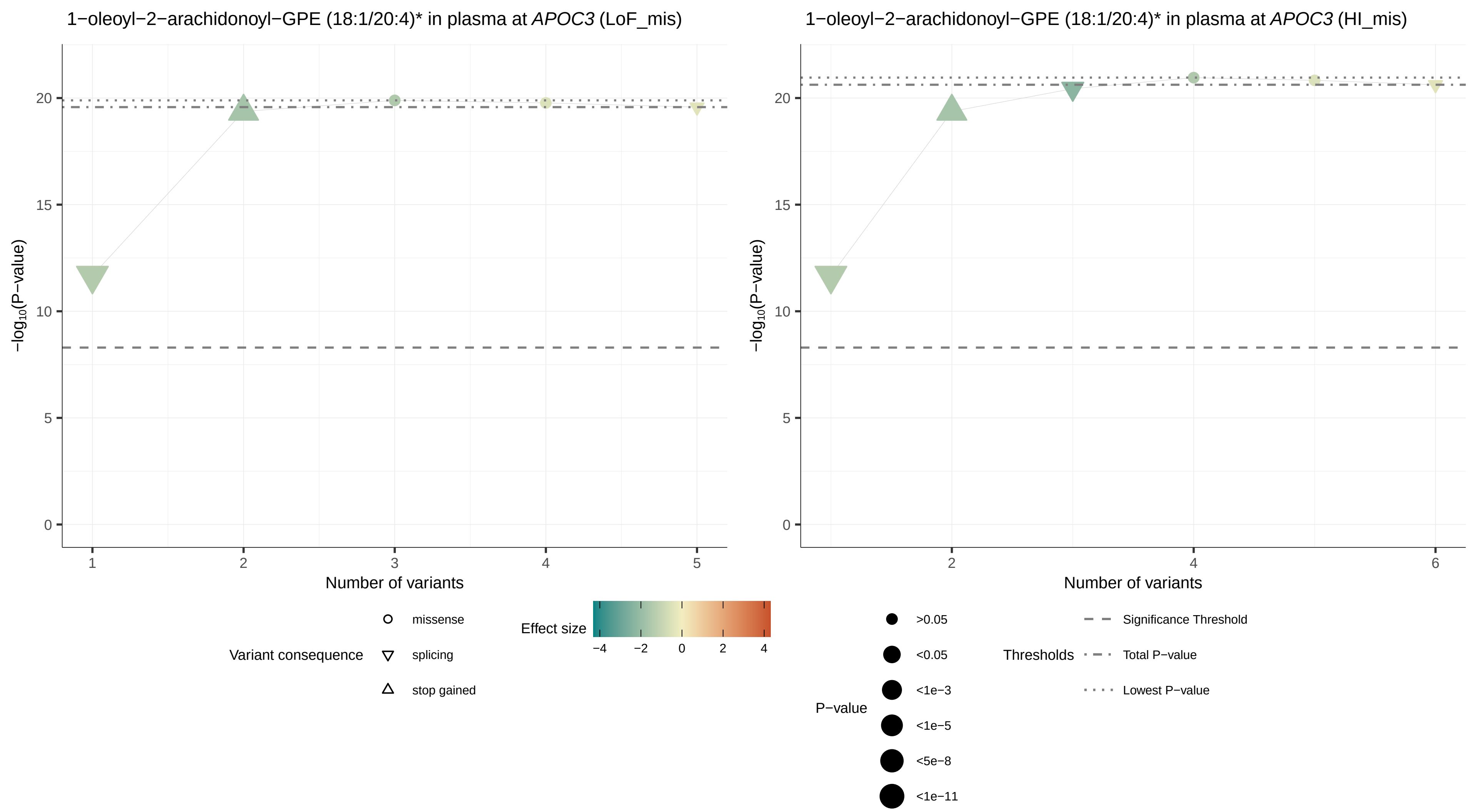

1−oleoyl−2−arachidonoyl−GPE (18:1/20:4)* in plasma at APOC3 (LoF_mis)
1−oleoyl−2−arachidonoyl−GPE (18:1/20:4)* in plasma at APOC3 (HI_mis)
−log10(P−value)
0
5
10
15
20
Number of variants
1
2
3
4
5
6
Variant consequence
missense
splicing
stop gained
Effect size
−4
−2
0
2
4
P−value
>0.05
<0.05
<1e−3
<1e−5
<5e−8
<1e−11
Thresholds
Significance Threshold
Total P−value
Lowest P−value

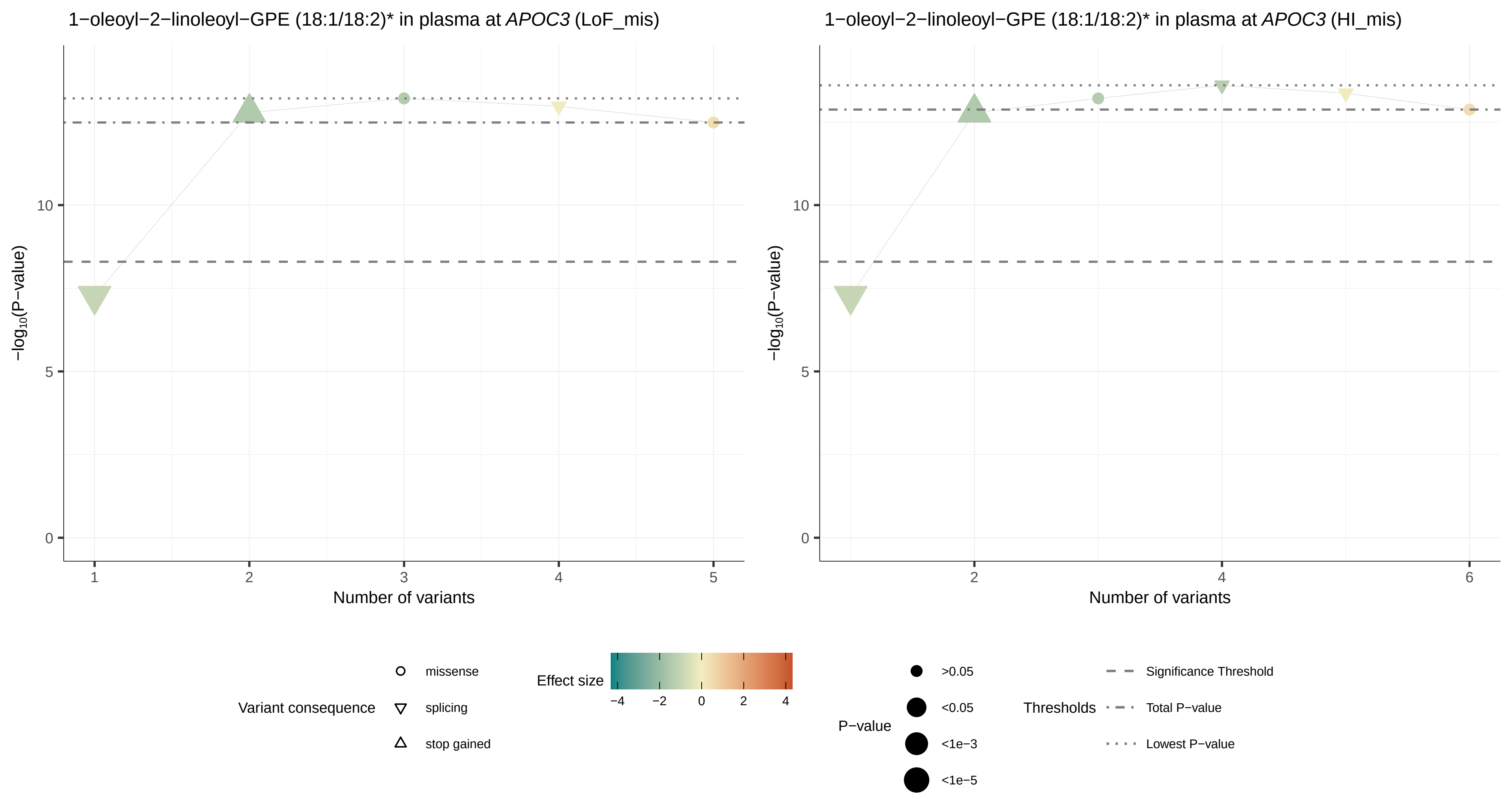

1−oleoyl−2−linoleoyl−GPE (18:1/18:2)* in plasma at APOC3 (LoF_mis)
1−oleoyl−2−linoleoyl−GPE (18:1/18:2)* in plasma at APOC3 (HI_mis)
−log10(P−value)
0
5
10
1
2
3
4
5
6
Number of variants
Variant consequence
missense
splicing
stop gained
Effect size
−4
−2
0
2
4
P−value
>0.05
<0.05
<1e−3
<1e−5
Thresholds
Significance Threshold
Total P−value
Lowest P−value

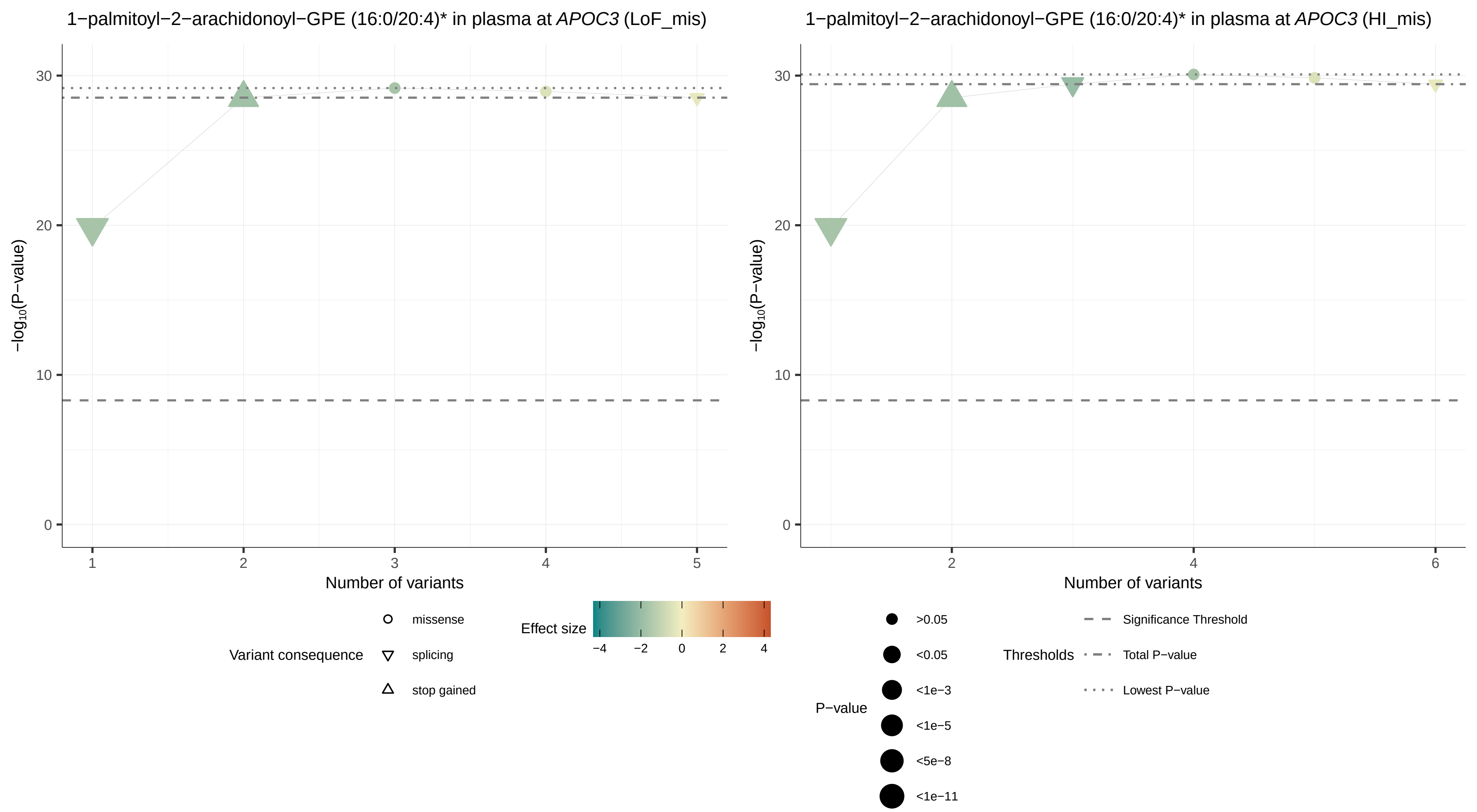
1−palmitoyl−2−arachidonoyl−GPE (16:0/20:4)* in plasma at APOC3 (LoF_mis)
1−palmitoyl−2−arachidonoyl−GPE (16:0/20:4)* in plasma at APOC3 (HI_mis)
−log10(P−value)
Number of variants
Variant consequence
missense
splicing
stop gained
Effect size
−4
−2
0
2
4
P−value
>0.05
<0.05
<1e−3
<1e−5
<5e−8
<1e−11
Thresholds
Significance Threshold
Total P−value
Lowest P−value

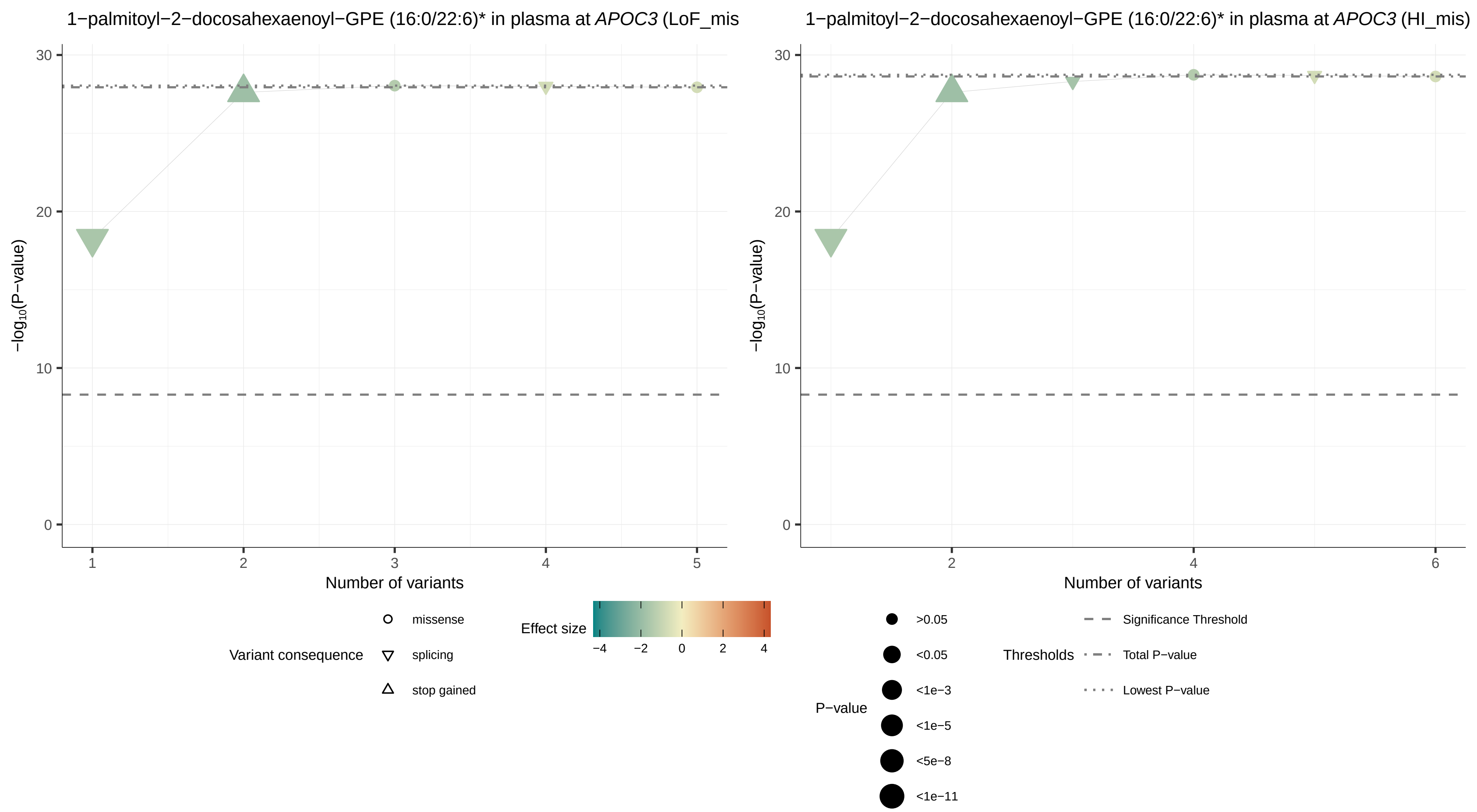

1−palmitoyl−2−docosahexaenoyl−GPE (16:0/22:6)* in plasma at APOC3 (LoF_mis
1−palmitoyl−2−docosahexaenoyl−GPE (16:0/22:6)* in plasma at APOC3 (HI_mis)
−log10(P−value)
Number of variants
Variant consequence
missense
splicing
stop gained
Effect size
−4
−2
0
2
4
P−value
>0.05
<0.05
<1e−3
<1e−5
<5e−8
<1e−11
Thresholds
Significance Threshold
Total P−value
Lowest P−value

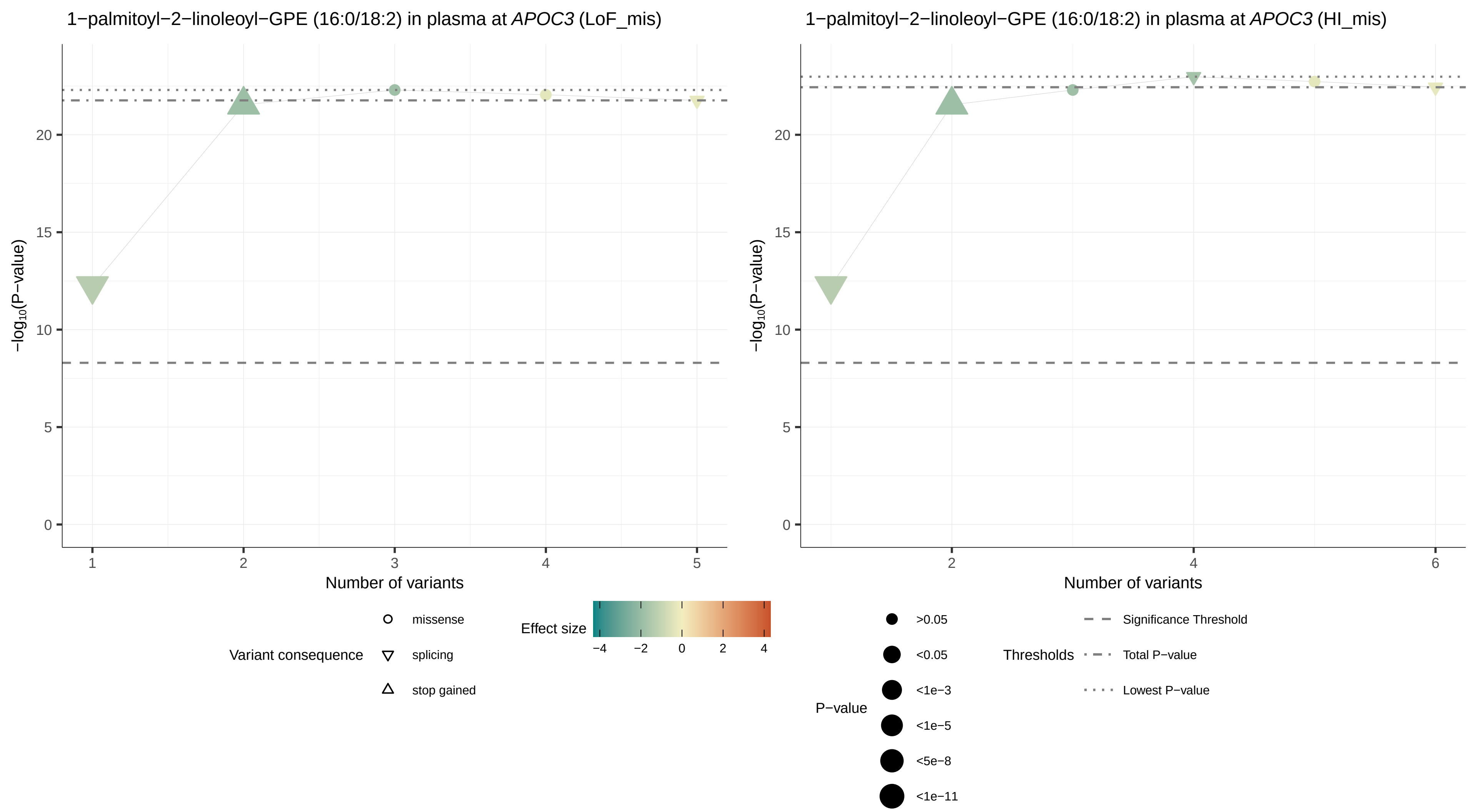

1−palmitoyl−2−linoleoyl−GPE (16:0/18:2) in plasma at APOC3 (LoF_mis)
1−palmitoyl−2−linoleoyl−GPE (16:0/18:2) in plasma at APOC3 (HI_mis)
−log10(P−value)
Number of variants
Variant consequence
missense
splicing
stop gained
Effect size
P−value
>0.05
<0.05
<1e−3
<1e−5
<5e−8
<1e−11
Thresholds
Significance Threshold
Total P−value
Lowest P−value

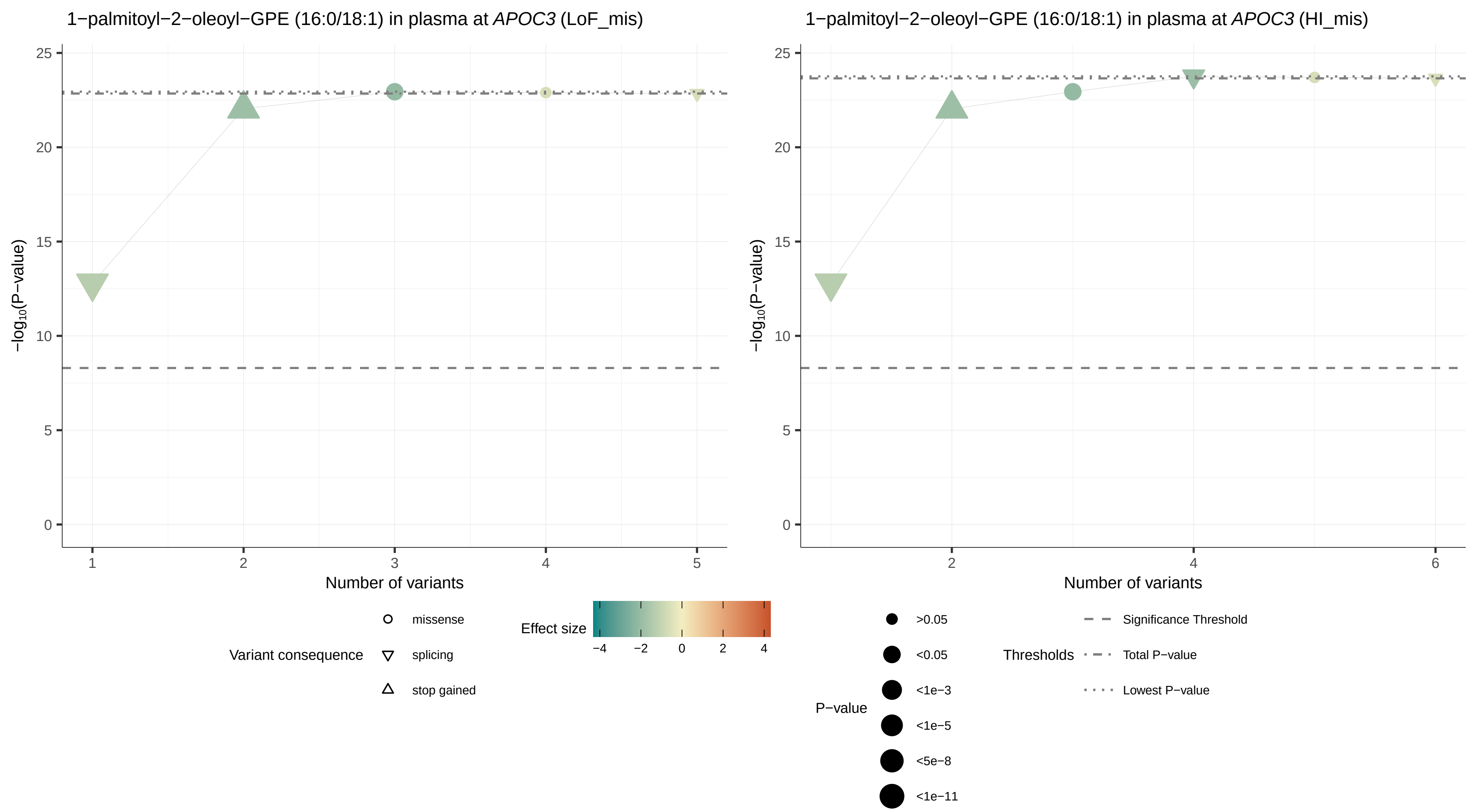

1−palmitoyl−2−oleoyl−GPE (16:0/18:1) in plasma at APOC3 (LoF_mis)
1−palmitoyl−2−oleoyl−GPE (16:0/18:1) in plasma at APOC3 (HI_mis)
−log10(P−value)
Number of variants
Variant consequence
missense
splicing
stop gained
Effect size
−4
−2
0
2
4
P−value
>0.05
<0.05
<1e−3
<1e−5
<5e−8
<1e−11
Thresholds
Significance Threshold
Total P−value
Lowest P−value

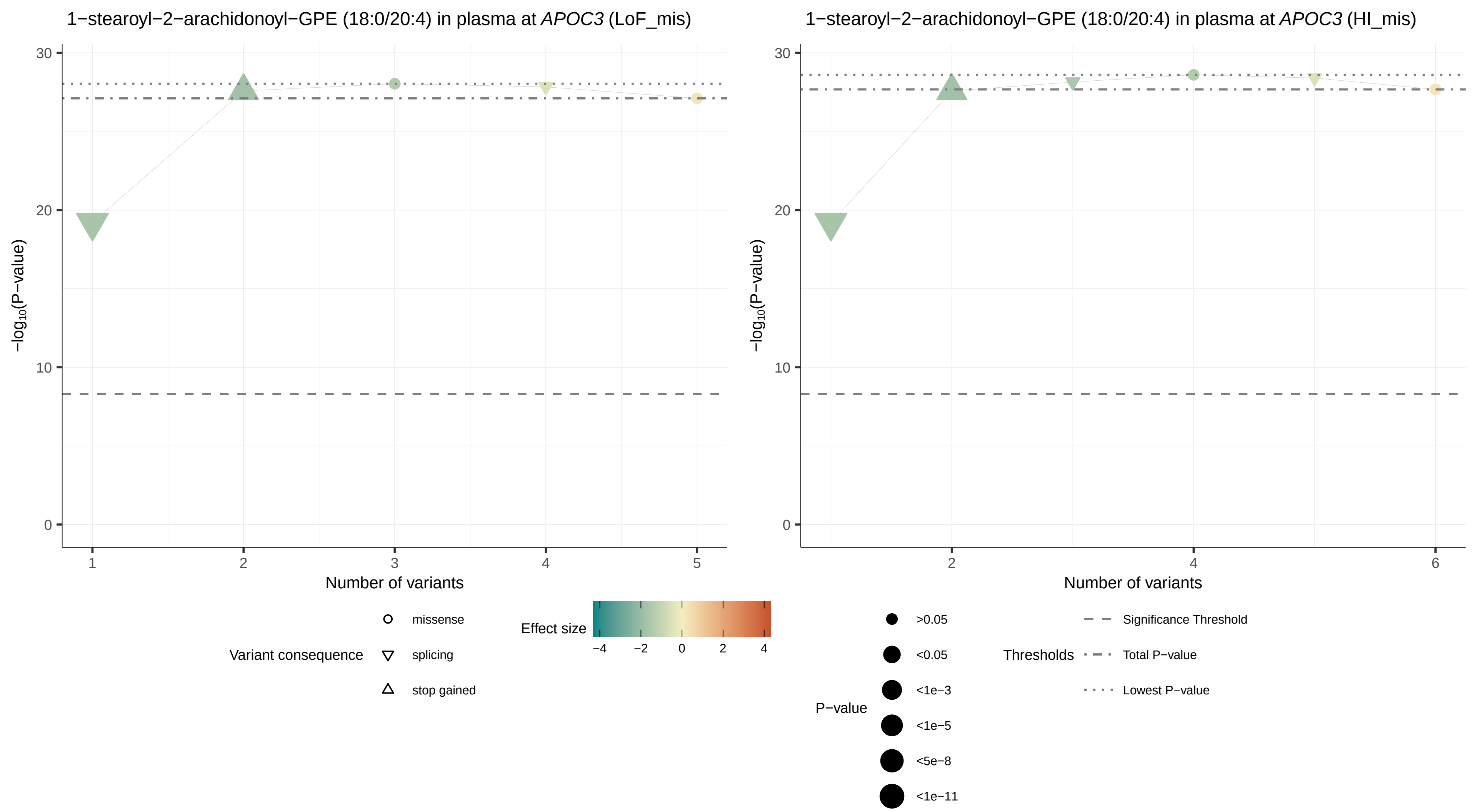

1−stearoyl−2−arachidonoyl−GPE (18:0/20:4) in plasma at APOC3 (LoF_mis)
1−stearoyl−2−arachidonoyl−GPE (18:0/20:4) in plasma at APOC3 (HI_mis)
−log10(P−value)
Number of variants
Variant consequence
missense
splicing
stop gained
Effect size
P−value
>0.05
<0.05
<1e−3
<1e−5
<5e−8
<1e−11
Thresholds
Significance Threshold
Total P−value
Lowest P−value

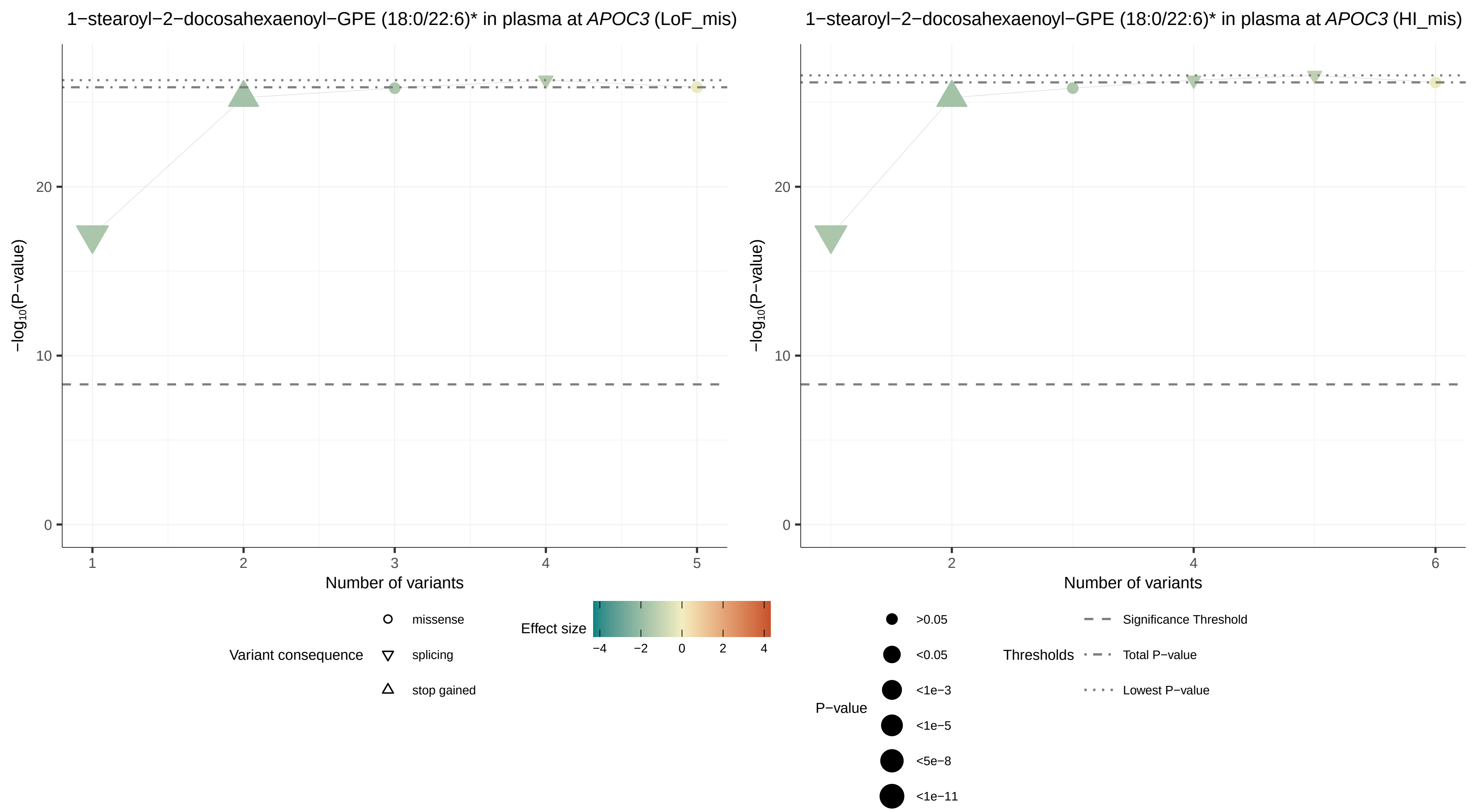

1−stearoyl−2−docosahexaenoyl−GPE (18:0/22:6)* in plasma at *APOC3* (LoF_mis)
1−stearoyl−2−docosahexaenoyl−GPE (18:0/22:6)* in plasma at *APOC3* (HI_mis)
−log10(P−value)
0
10
20
1
2
3
4
5
6
Number of variants
Variant consequence
missense
splicing
stop gained
Effect size
−4
−2
0
2
4
P−value
>0.05
<0.05
<1e−3
<1e−5
<5e−8
<1e−11
Thresholds
Significance Threshold
Total P−value
Lowest P−value

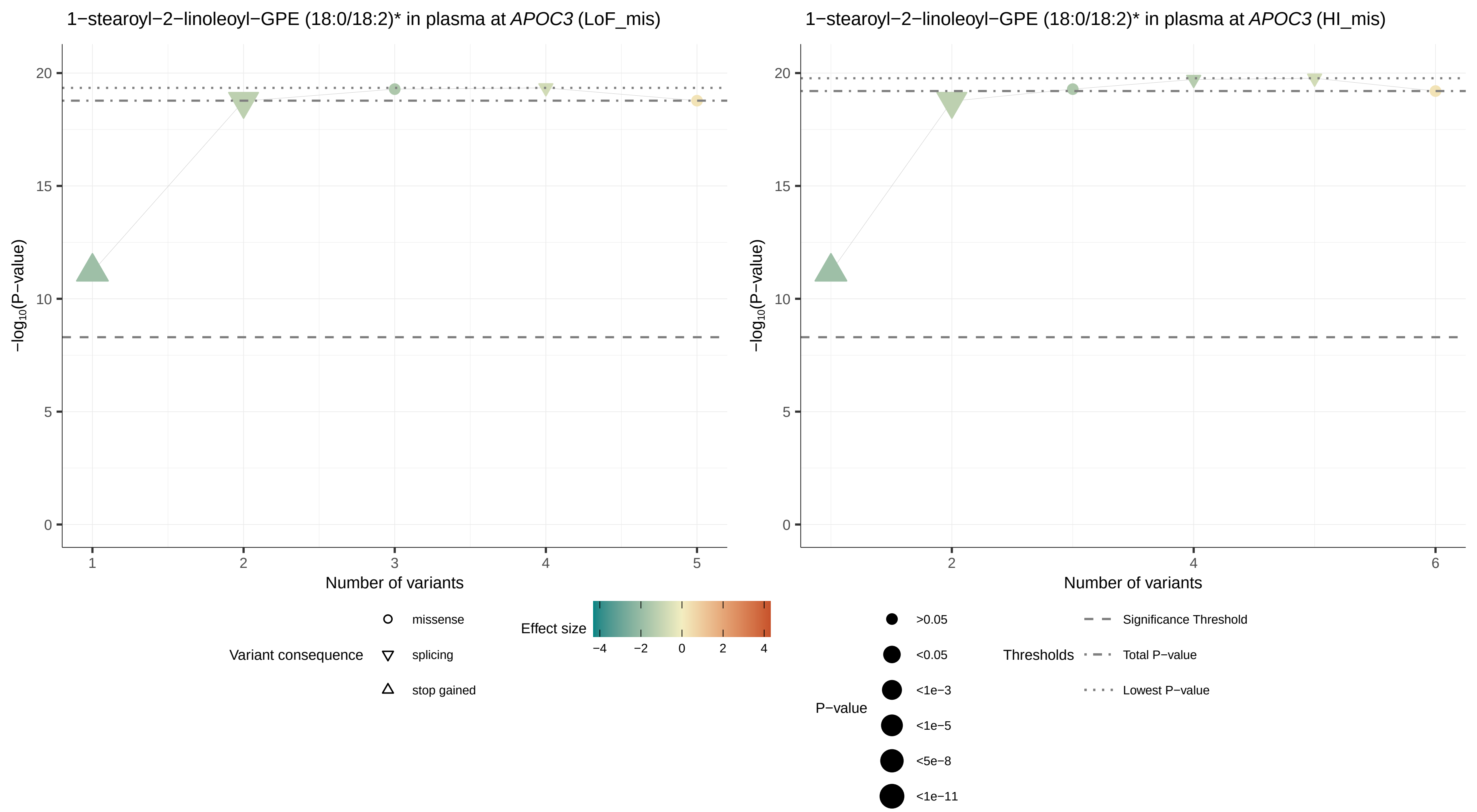
1−stearoyl−2−linoleoyl−GPE (18:0/18:2)* in plasma at APOC3 (LoF_mis)
1−stearoyl−2−linoleoyl−GPE (18:0/18:2)* in plasma at APOC3 (HI_mis)
−log10(P−value)
Number of variants
Variant consequence
missense
splicing
stop gained
Effect size
P−value
>0.05
<0.05
<1e−3
<1e−5
<5e−8
<1e−11
Thresholds
Significance Threshold
Total P−value
Lowest P−value

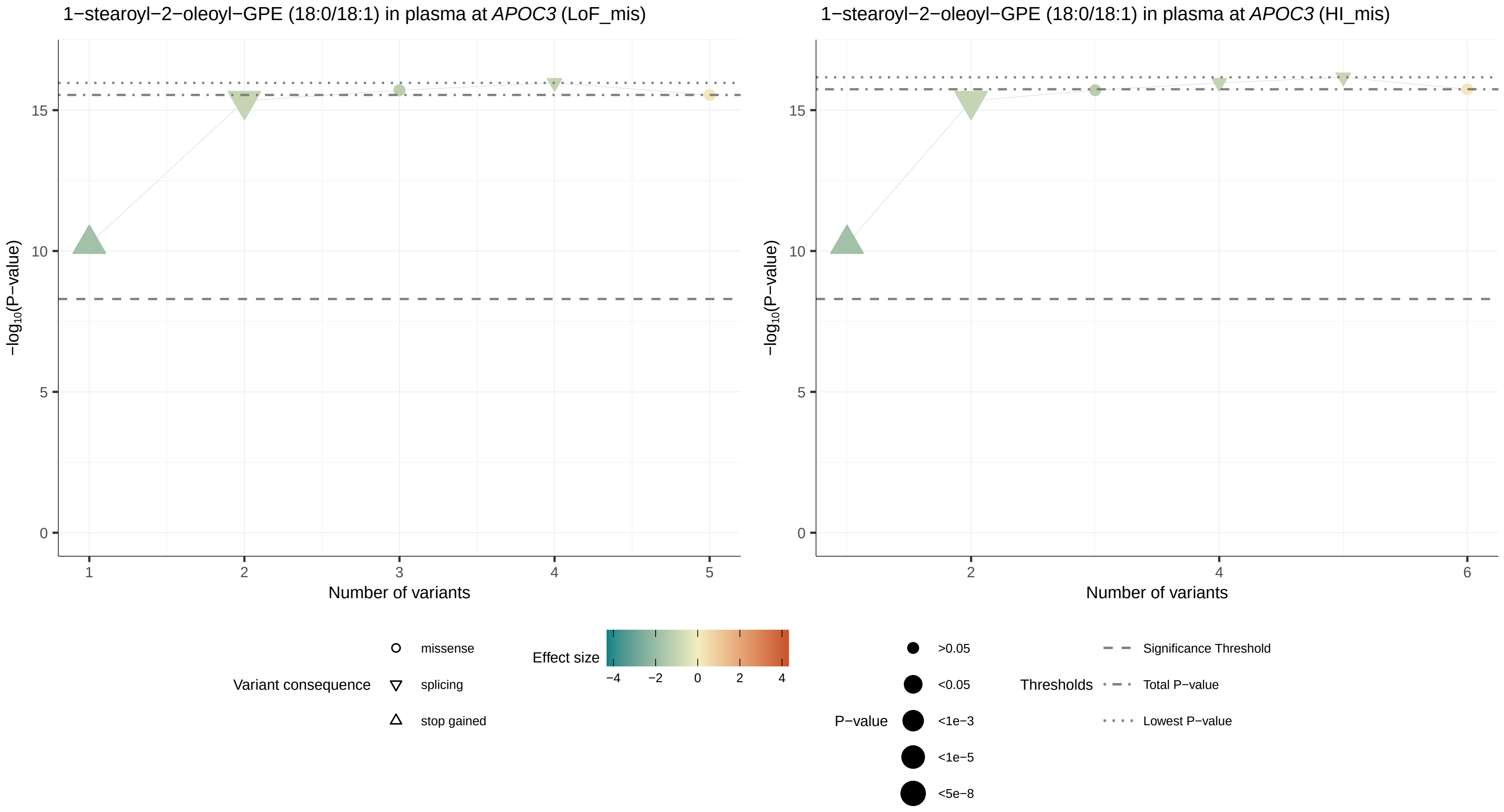
1−stearoyl−2−oleoyl−GPE (18:0/18:1) in plasma at APOC3 (LoF_mis)
1−stearoyl−2−oleoyl−GPE (18:0/18:1) in plasma at APOC3 (HI_mis)
−log10(P−value)
Number of variants
Variant consequence
missense
splicing
stop gained
Effect size
P−value
>0.05
<0.05
<1e−3
<1e−5
<5e−8
Thresholds
Significance Threshold
Total P−value
Lowest P−value

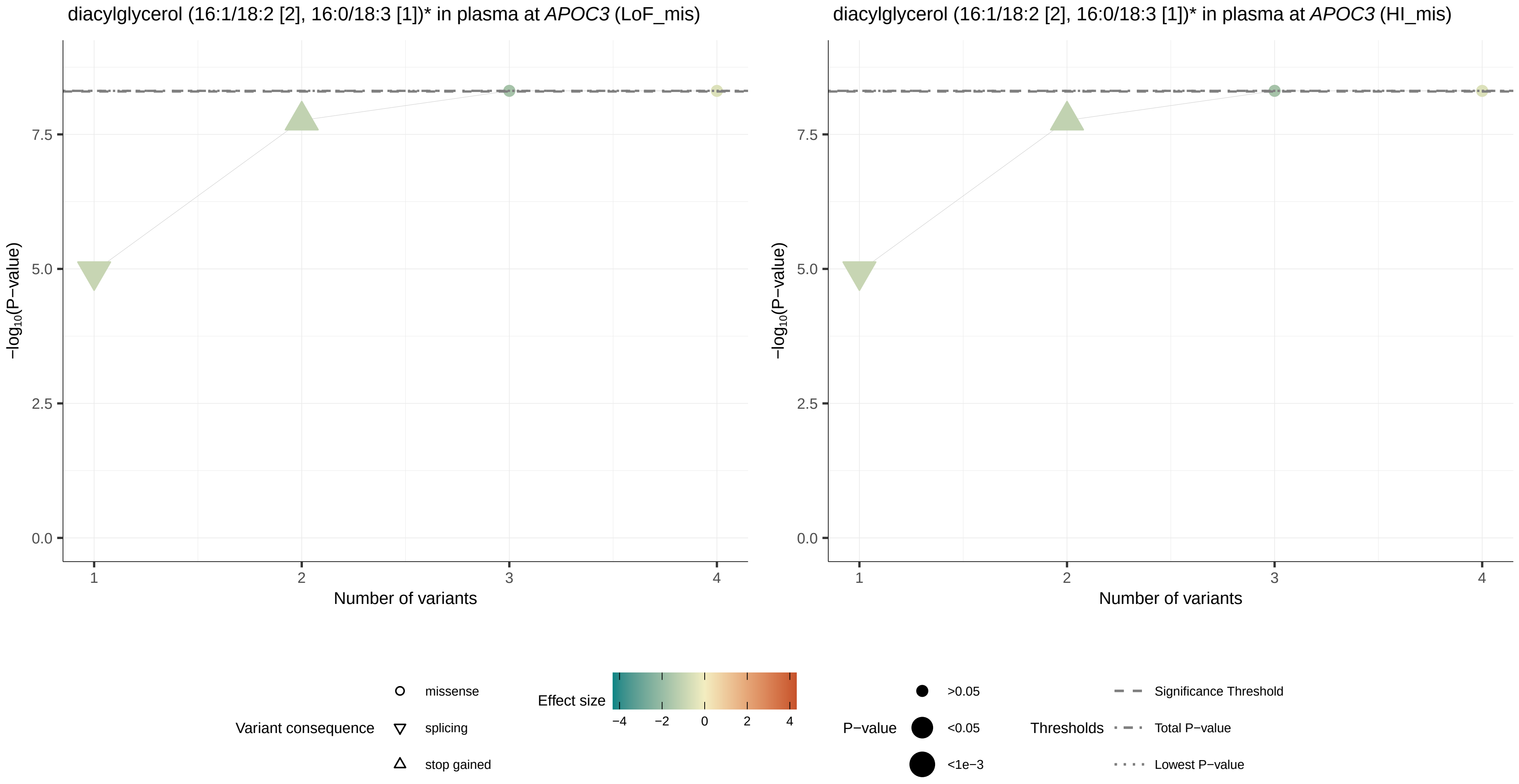

diacylglycerol (16:1/18:2 [2], 16:0/18:3 [1])* in plasma at APOC3 (LoF_mis)
diacylglycerol (16:1/18:2 [2], 16:0/18:3 [1])* in plasma at APOC3 (HI_mis)
−log10(P−value)
0.0
2.5
5.0
7.5
1
2
3
4
Number of variants
Variant consequence
missense
splicing
stop gained
Effect size
−4
−2
0
2
4
P−value
>0.05
<0.05
<1e−3
Thresholds
Significance Threshold
Total P−value
Lowest P−value

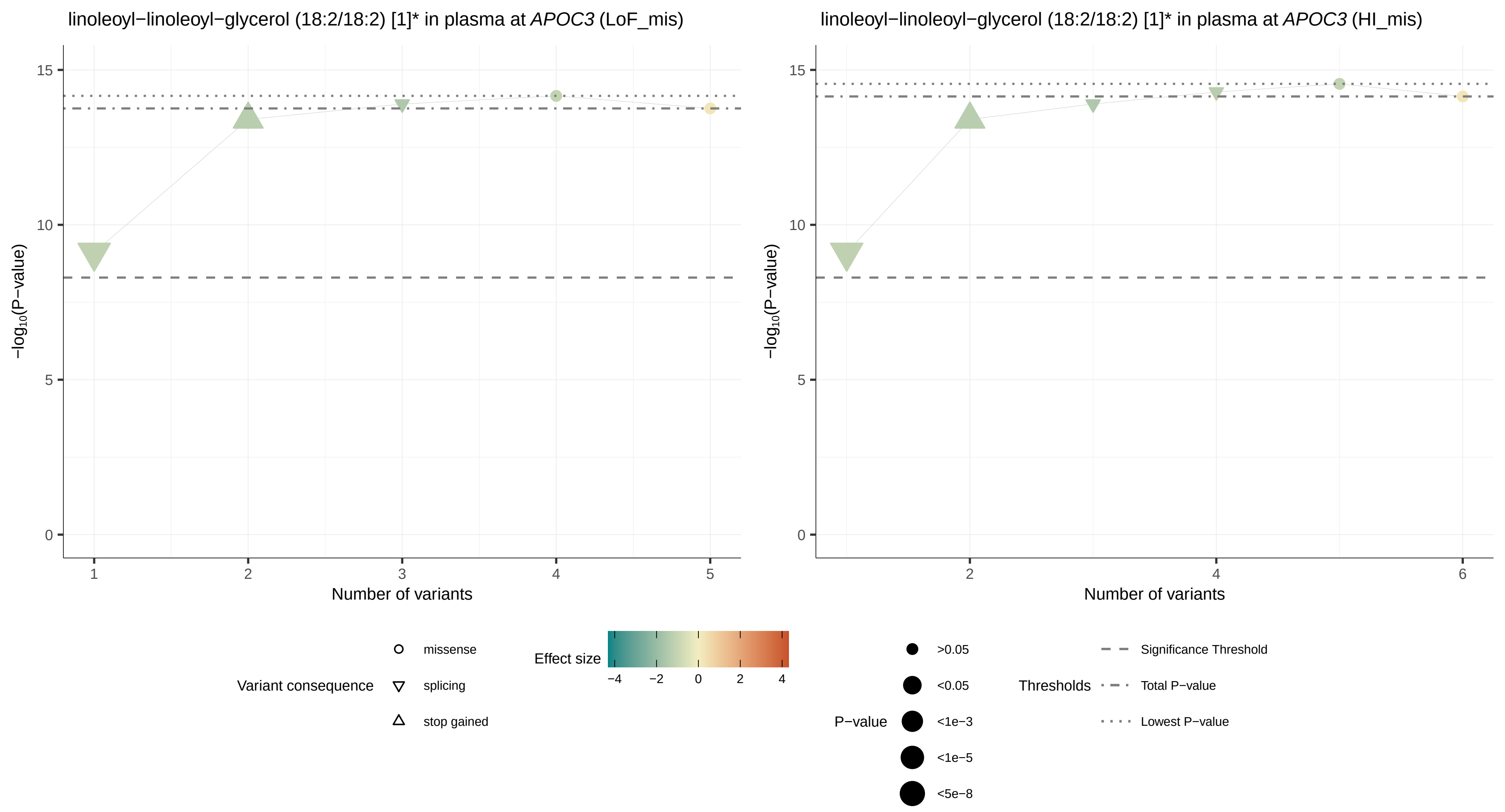

linoleoyl−linoleoyl−glycerol (18:2/18:2) [1]* in plasma at APOC3 (LoF_mis)
linoleoyl−linoleoyl−glycerol (18:2/18:2) [1]* in plasma at APOC3 (HI_mis)
−log10(P−value)
0
5
10
15
1
2
3
4
5
6
Number of variants
Variant consequence
missense
splicing
stop gained
Effect size
−4
−2
0
2
4
P−value
>0.05
<0.05
<1e−3
<1e−5
<5e−8
Thresholds
Significance Threshold
Total P−value
Lowest P−value

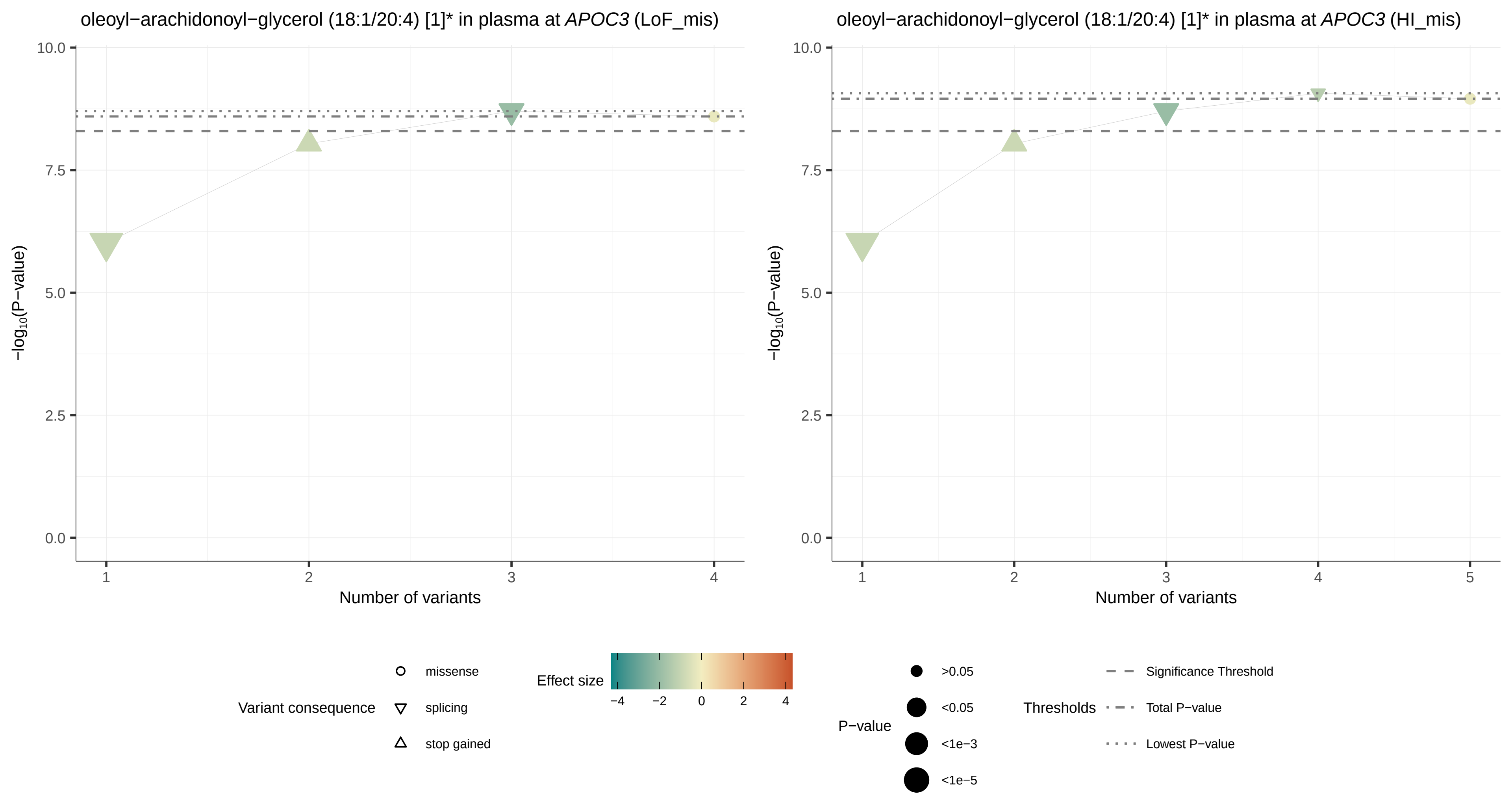

oleoyl−arachidonoyl−glycerol (18:1/20:4) [1]* in plasma at APOC3 (LoF_mis)
oleoyl−arachidonoyl−glycerol (18:1/20:4) [1]* in plasma at APOC3 (HI_mis)
10.0
7.5
5.0
2.5
0.0
−log10(P−value)
1
2
3
4
5
Number of variants
Variant consequence
missense
splicing
stop gained
Effect size
−4
−2
0
2
4
P−value
>0.05
<0.05
<1e−3
<1e−5
Thresholds
Significance Threshold
Total P−value
Lowest P−value

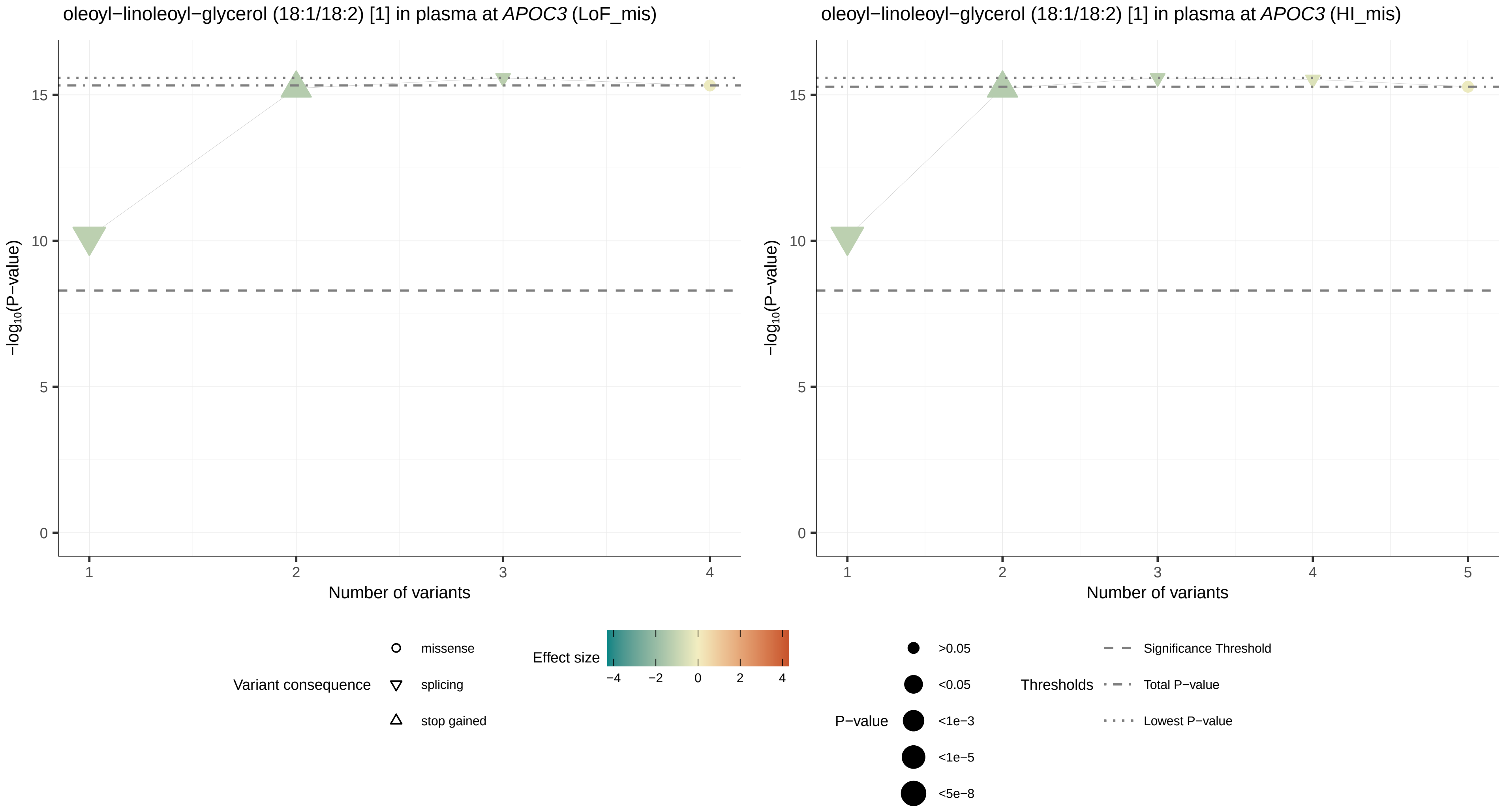

oleoyl−linoleoyl−glycerol (18:1/18:2) [1] in plasma at *APOC3* (LoF_mis)
oleoyl−linoleoyl−glycerol (18:1/18:2) [1] in plasma at *APOC3* (HI_mis)
−log10(P−value)
Number of variants
Variant consequence
missense
splicing
stop gained
Effect size
P−value
>0.05
<0.05
<1e−3
<1e−5
<5e−8
Thresholds
Significance Threshold
Total P−value
Lowest P−value

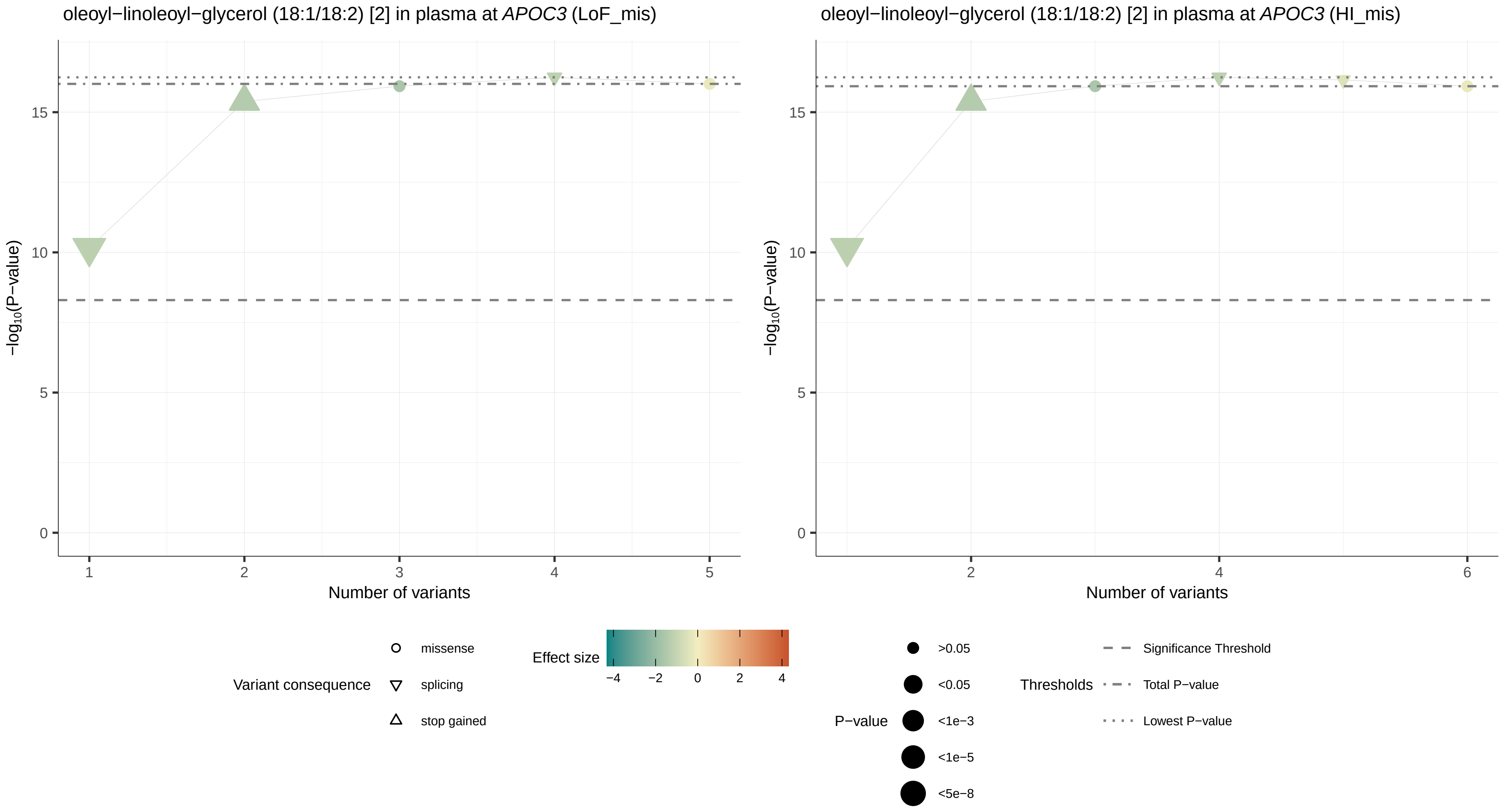
oleoyl−linoleoyl−glycerol (18:1/18:2) [2] in plasma at APOC3 (LoF_mis)
oleoyl−linoleoyl−glycerol (18:1/18:2) [2] in plasma at APOC3 (HI_mis)
−log10(P−value)
Number of variants
Variant consequence
missense
splicing
stop gained
Effect size
P−value
>0.05
<0.05
<1e−3
<1e−5
<5e−8
Thresholds
Significance Threshold
Total P−value
Lowest P−value

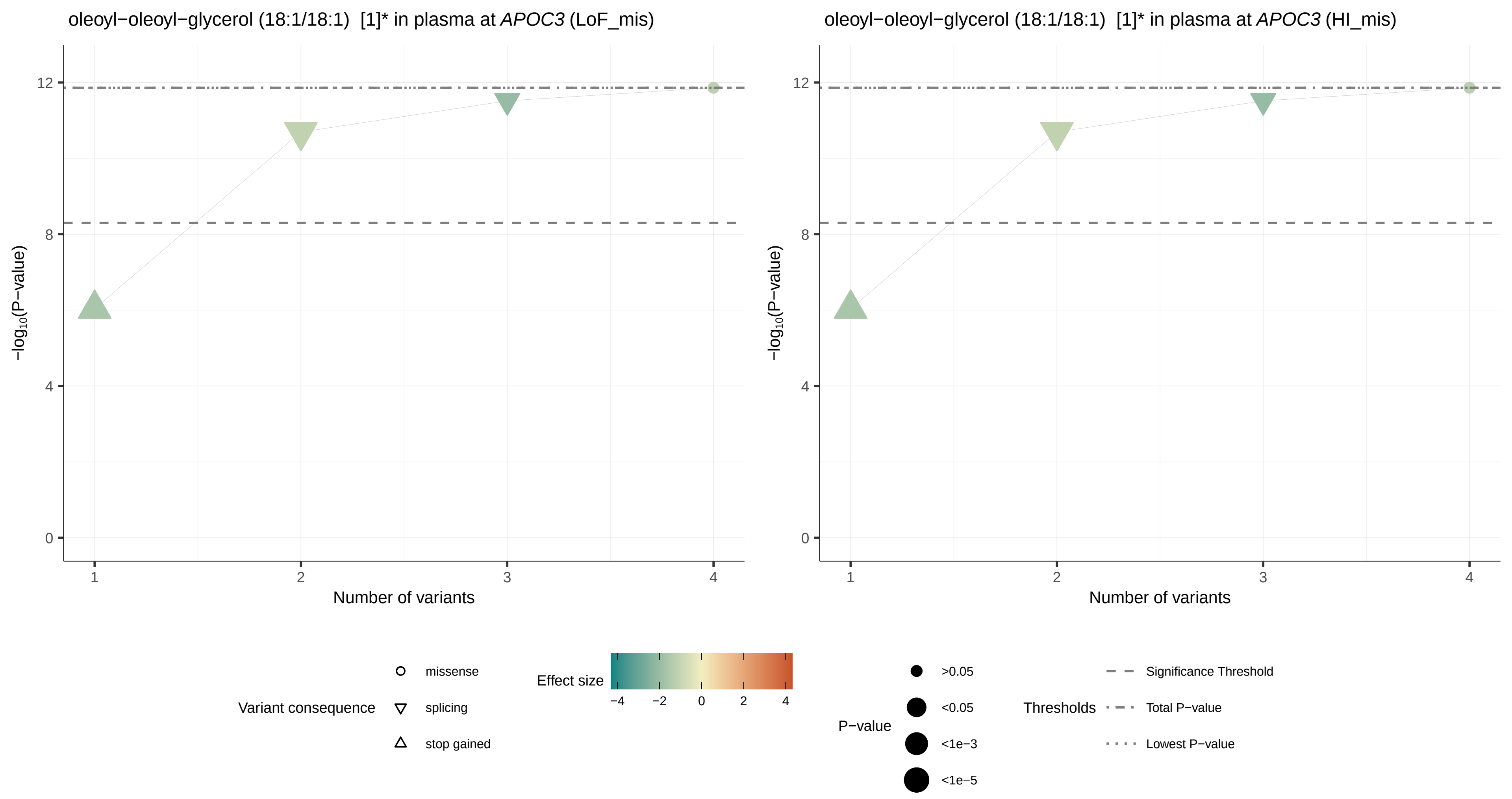

oleoyl−oleoyl−glycerol (18:1/18:1) [1]* in plasma at APOC3 (LoF_mis)
oleoyl−oleoyl−glycerol (18:1/18:1) [1]* in plasma at APOC3 (HI_mis)
−log10(P−value)
Number of variants
Variant consequence
missense
splicing
stop gained
Effect size
−4
−2
0
2
4
P−value
>0.05
<0.05
<1e−3
<1e−5
Thresholds
Significance Threshold
Total P−value
Lowest P−value

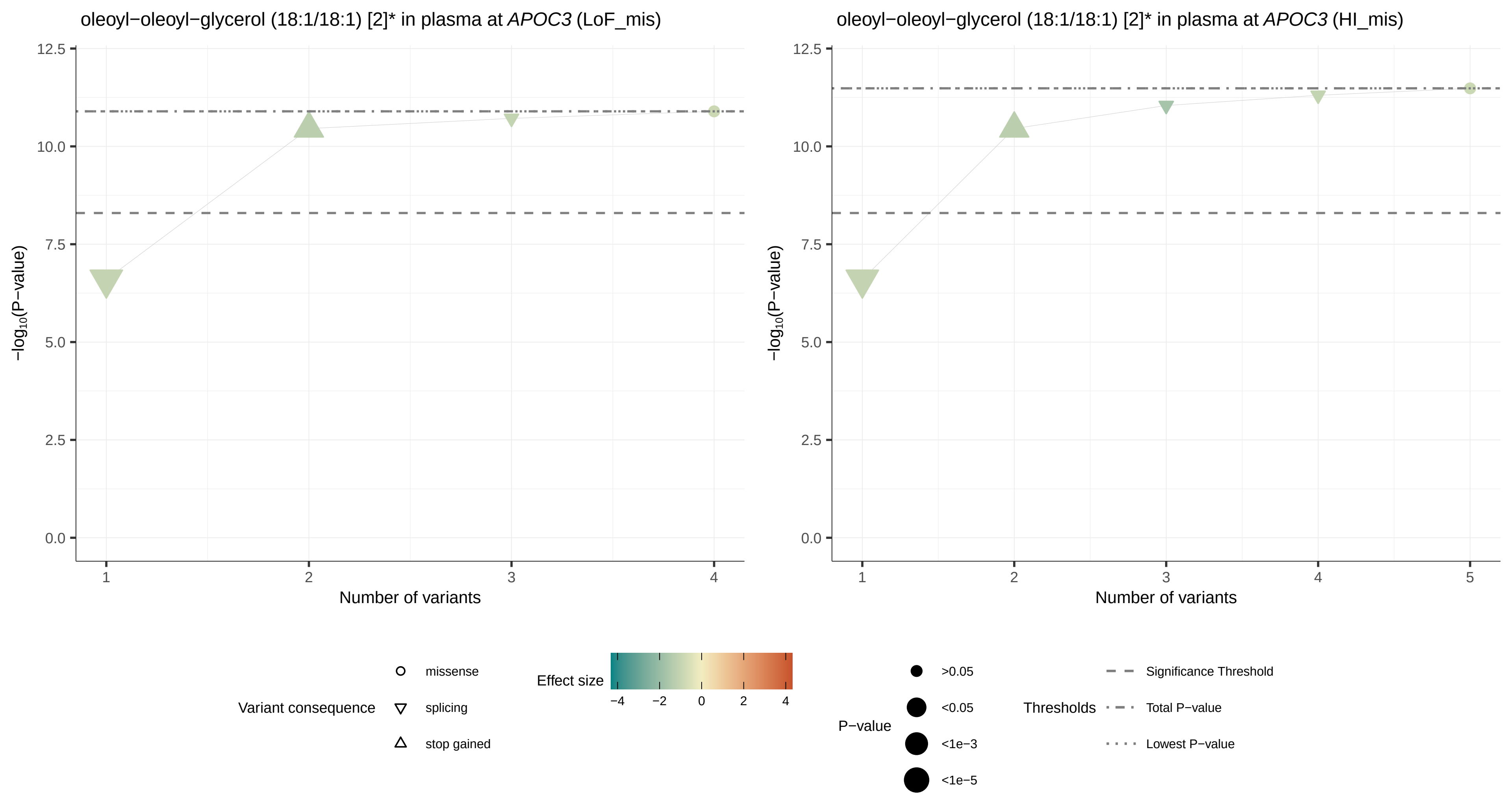
oleoyl−oleoyl−glycerol (18:1/18:1) [2]* in plasma at APOC3 (LoF_mis)
oleoyl−oleoyl−glycerol (18:1/18:1) [2]* in plasma at APOC3 (HI_mis)
−log10(P−value)
Number of variants
Variant consequence
missense
splicing
stop gained
Effect size
P−value
>0.05
<0.05
<1e−3
<1e−5
Thresholds
Significance Threshold
Total P−value
Lowest P−value

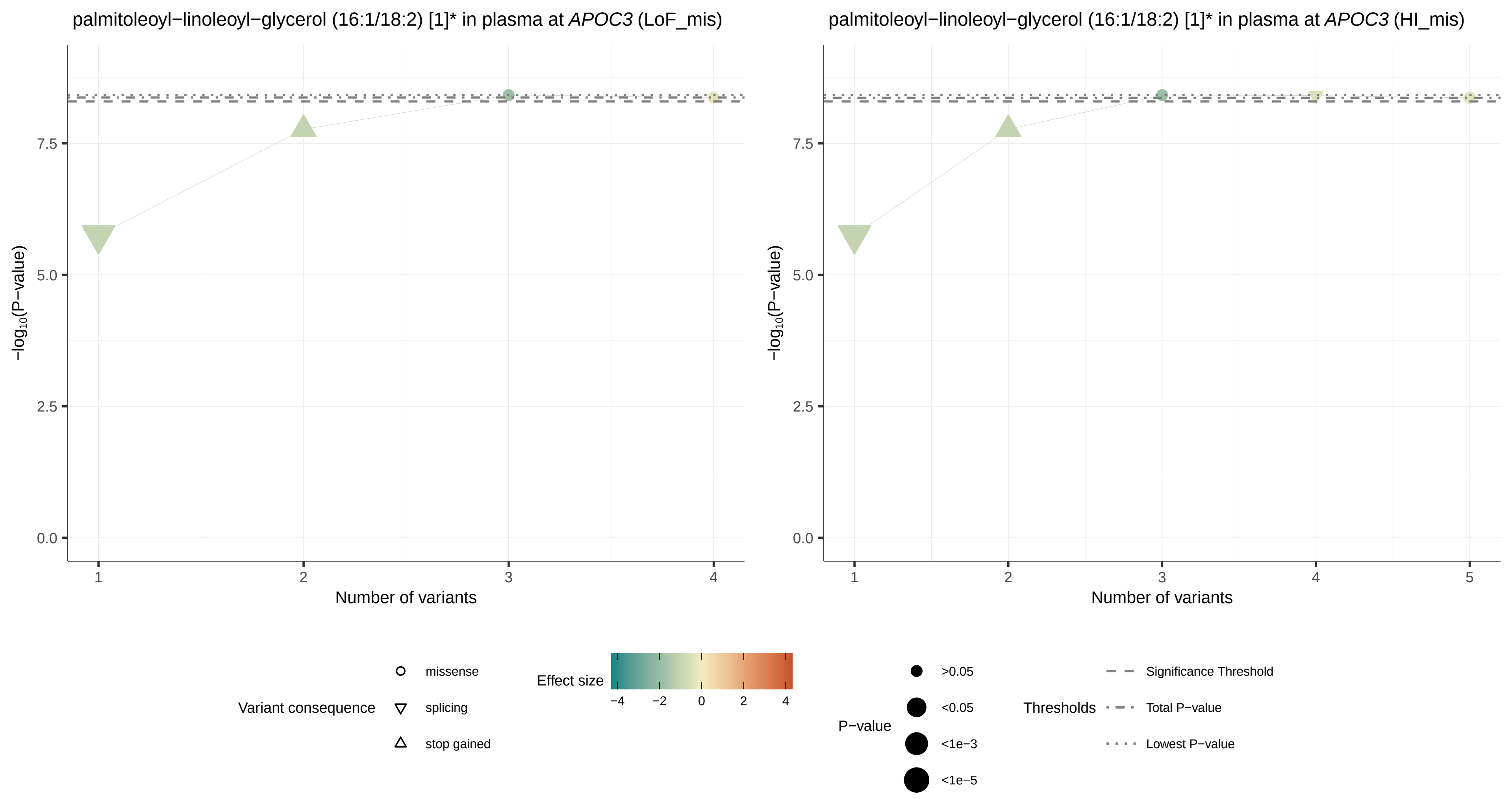

palmitoleoyl−linoleoyl−glycerol (16:1/18:2) [1]* in plasma at APOC3 (LoF_mis)
palmitoleoyl−linoleoyl−glycerol (16:1/18:2) [1]* in plasma at APOC3 (HI_mis)
−log10(P−value)
0.0
2.5
5.0
7.5
Number of variants
1
2
3
4
5
Variant consequence
missense
splicing
stop gained
Effect size
−4
−2
0
2
4
P−value
>0.05
<0.05
<1e−3
<1e−5
Thresholds
Significance Threshold
Total P−value
Lowest P−value

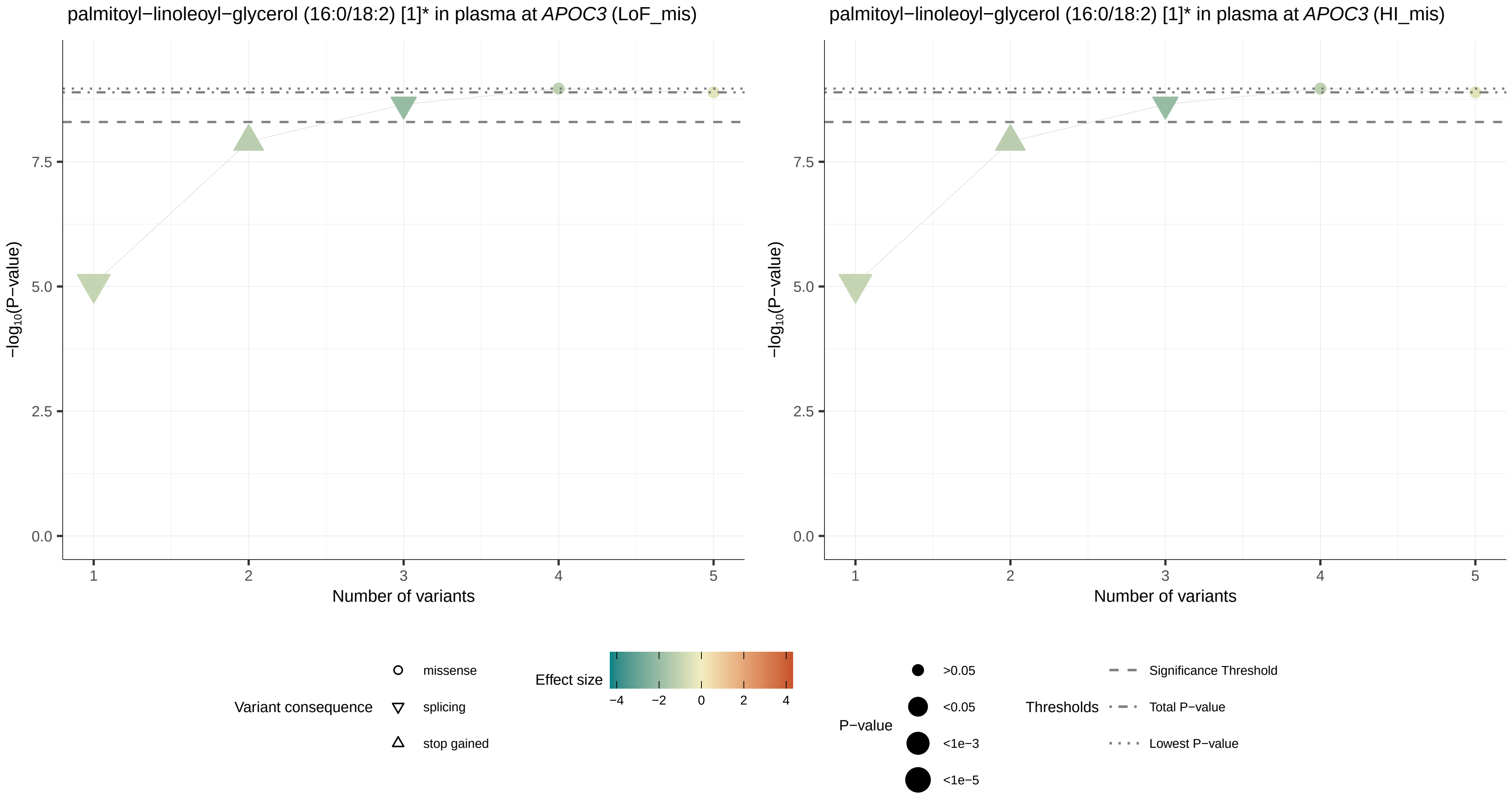

palmitoyl−linoleoyl−glycerol (16:0/18:2) [1]* in plasma at APOC3 (LoF_mis)
palmitoyl−linoleoyl−glycerol (16:0/18:2) [1]* in plasma at APOC3 (HI_mis)
−log10(P−value)
0.0
2.5
5.0
7.5
1
2
3
4
5
Number of variants
Variant consequence
missense
splicing
stop gained
Effect size
−4
−2
0
2
4
P−value
>0.05
<0.05
<1e−3
<1e−5
Thresholds
Significance Threshold
Total P−value
Lowest P−value

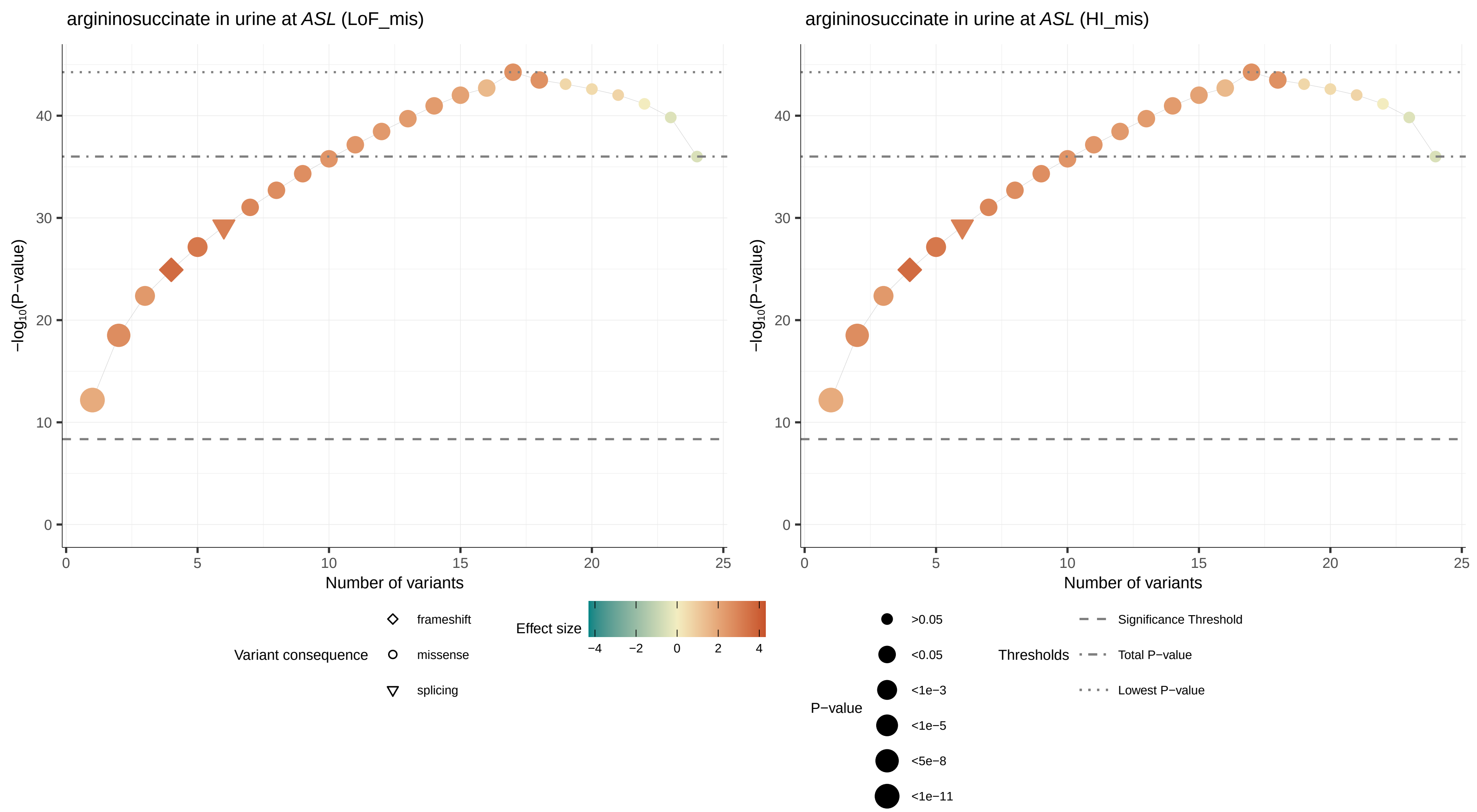

argininosuccinate in urine at *ASL* (LoF_mis)
argininosuccinate in urine at *ASL* (HI_mis)
−log10(P−value)
Number of variants
0
10
20
30
40
0
5
10
15
20
25
Variant consequence
frameshift
missense
splicing
Effect size
−4
−2
0
2
4
P−value
>0.05
<0.05
<1e−3
<1e−5
<5e−8
<1e−11
Thresholds
Significance Threshold
Total P−value
Lowest P−value

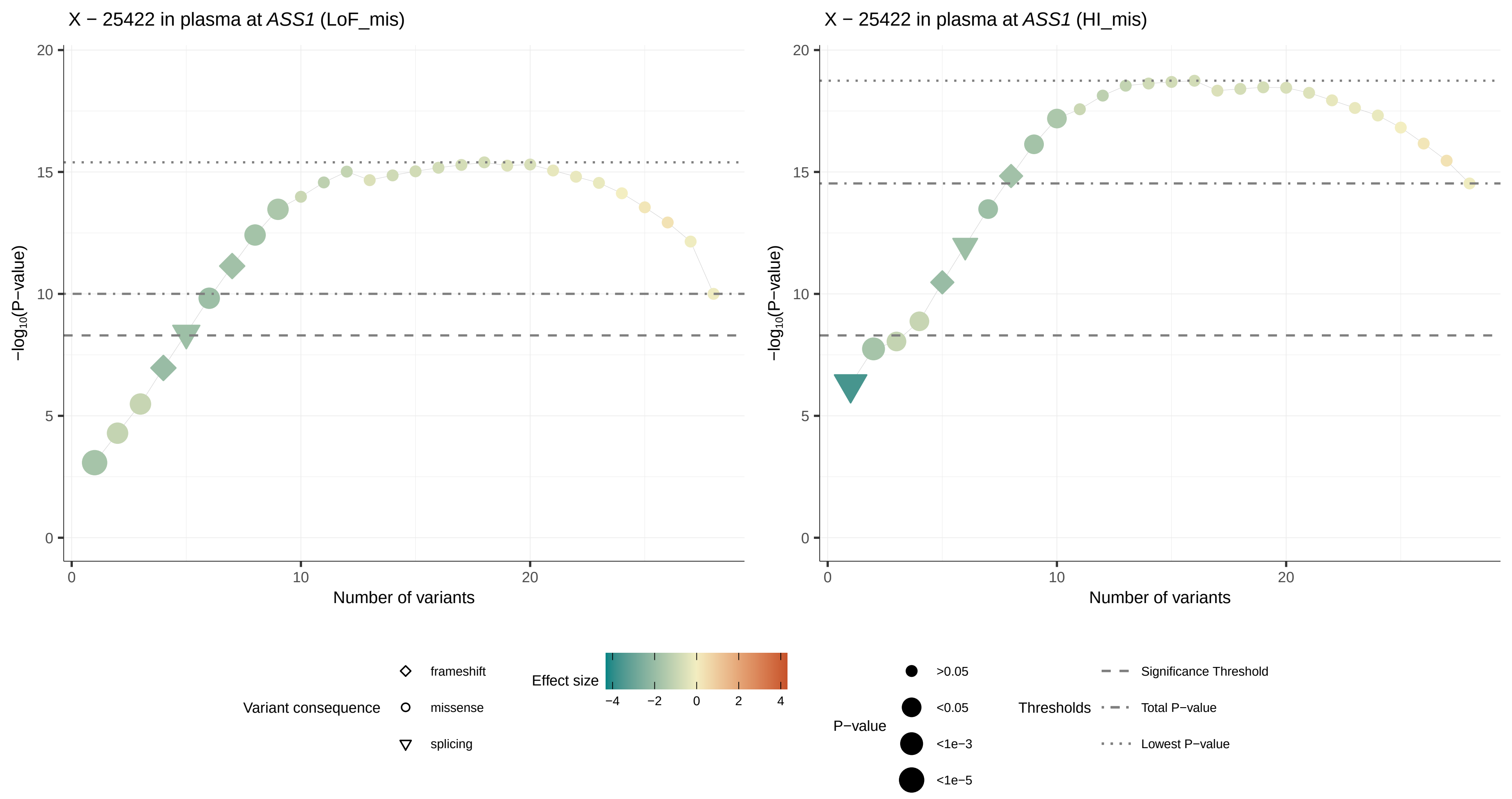

X − 25422 in plasma at ASS1 (LoF_mis)
X − 25422 in plasma at ASS1 (HI_mis)
−log10(P−value)
Number of variants
Variant consequence
frameshift
missense
splicing
Effect size
P−value
>0.05
<0.05
<1e−3
<1e−5
Thresholds
Significance Threshold
Total P−value
Lowest P−value

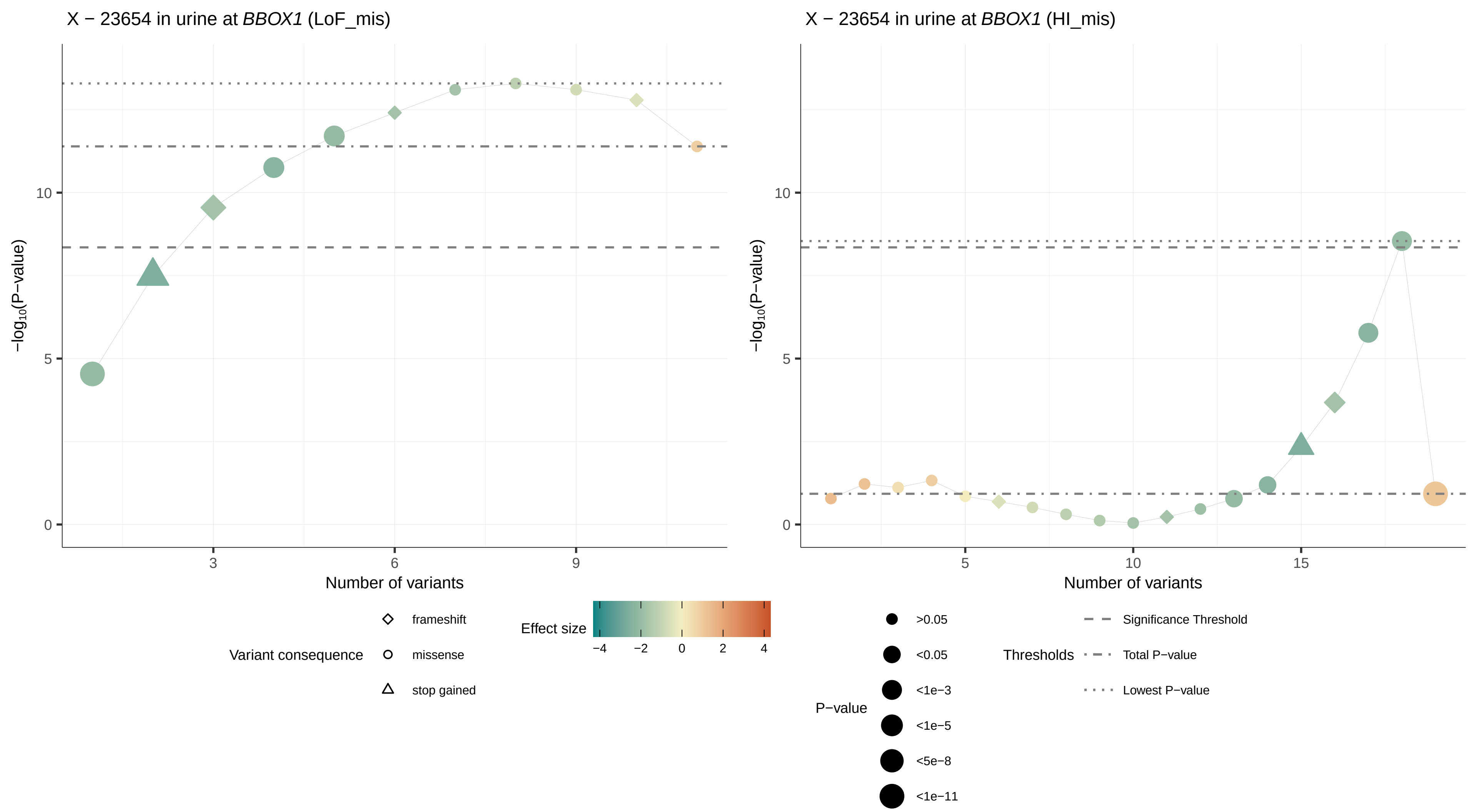

X − 23654 in urine at *BBOX1* (LoF_mis)
X − 23654 in urine at *BBOX1* (HI_mis)
−log10(P−value)
0
5
10
3
6
9
15
Number of variants
Variant consequence
frameshift
missense
stop gained
Effect size
−4
−2
0
2
4
P−value
>0.05
<0.05
<1e−3
<1e−5
<5e−8
<1e−11
Thresholds
Significance Threshold
Total P−value
Lowest P−value

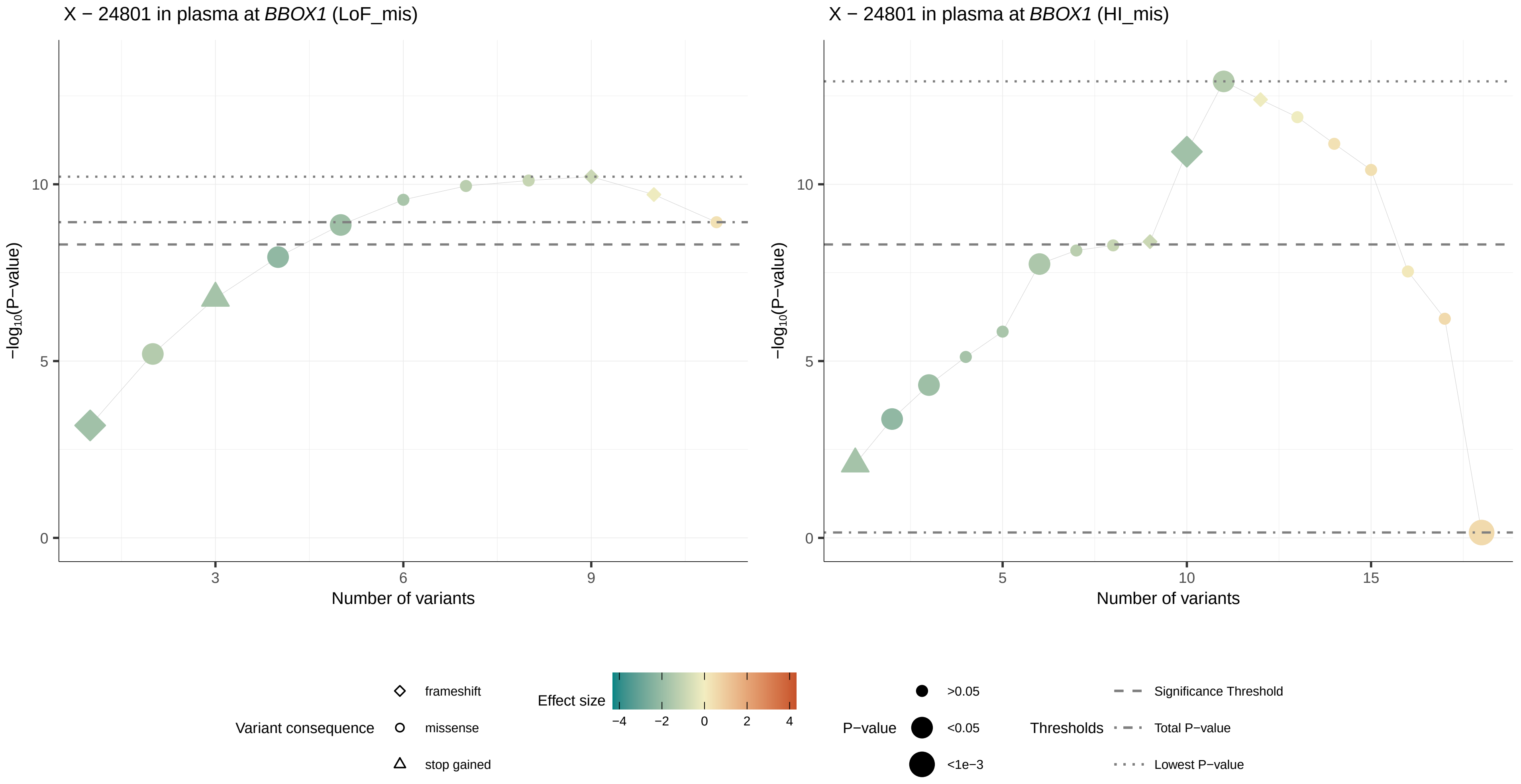

X − 24801 in plasma at BBOX1 (LoF_mis)
X − 24801 in plasma at BBOX1 (HI_mis)
−log10(P−value)
Number of variants
Variant consequence
frameshift
missense
stop gained
Effect size
−4
−2
0
2
4
P−value
>0.05
<0.05
<1e−3
Thresholds
Significance Threshold
Total P−value
Lowest P−value

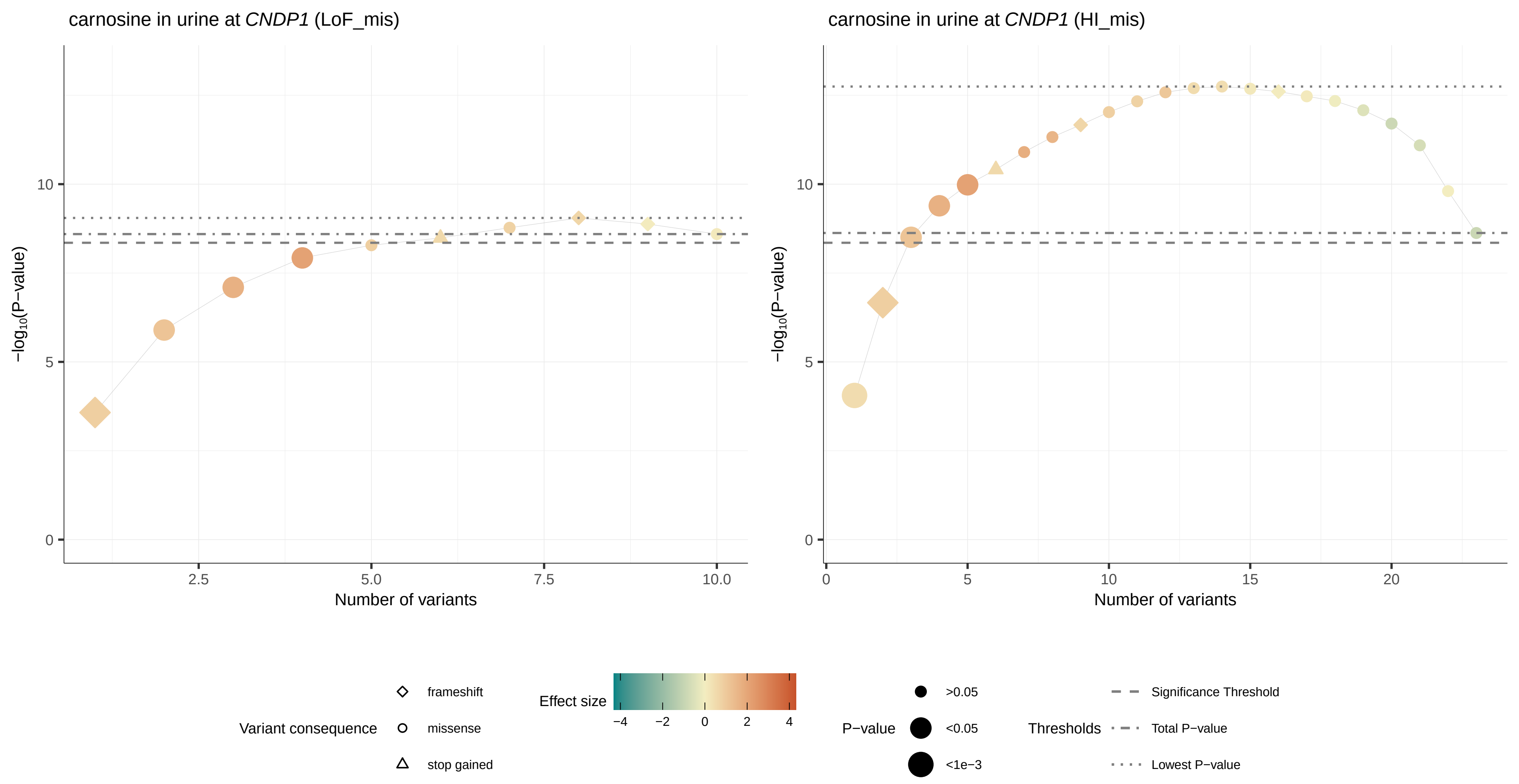

carnosine in urine at *CNDP1* (LoF_mis)
carnosine in urine at *CNDP1* (HI_mis)
−log10(P−value)
Number of variants
0
5
10
2.5
5.0
7.5
10.0
15
20
Variant consequence
frameshift
missense
stop gained
Effect size
−4
−2
0
2
4
P−value
>0.05
<0.05
<1e−3
Thresholds
Significance Threshold
Total P−value
Lowest P−value

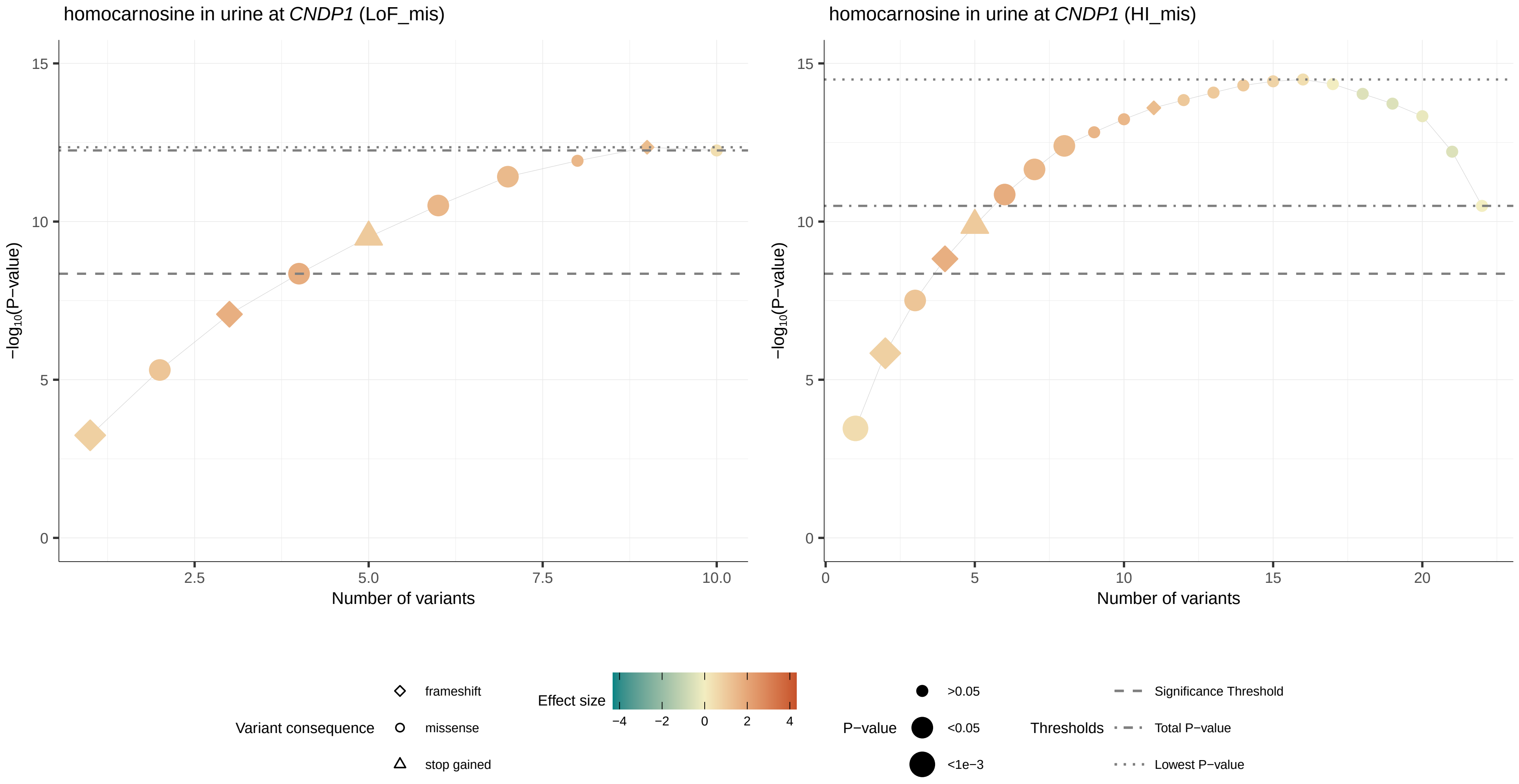
homocarnosine in urine at CNDP1 (LoF_mis)
homocarnosine in urine at CNDP1 (HI_mis)
−log10(P−value)
Number of variants
0
5
10
15
2.5
5.0
7.5
10.0
20
Variant consequence
frameshift
missense
stop gained
Effect size
−4
−2
0
2
4
P−value
>0.05
<0.05
<1e−3
Thresholds
Significance Threshold
Total P−value
Lowest P−value

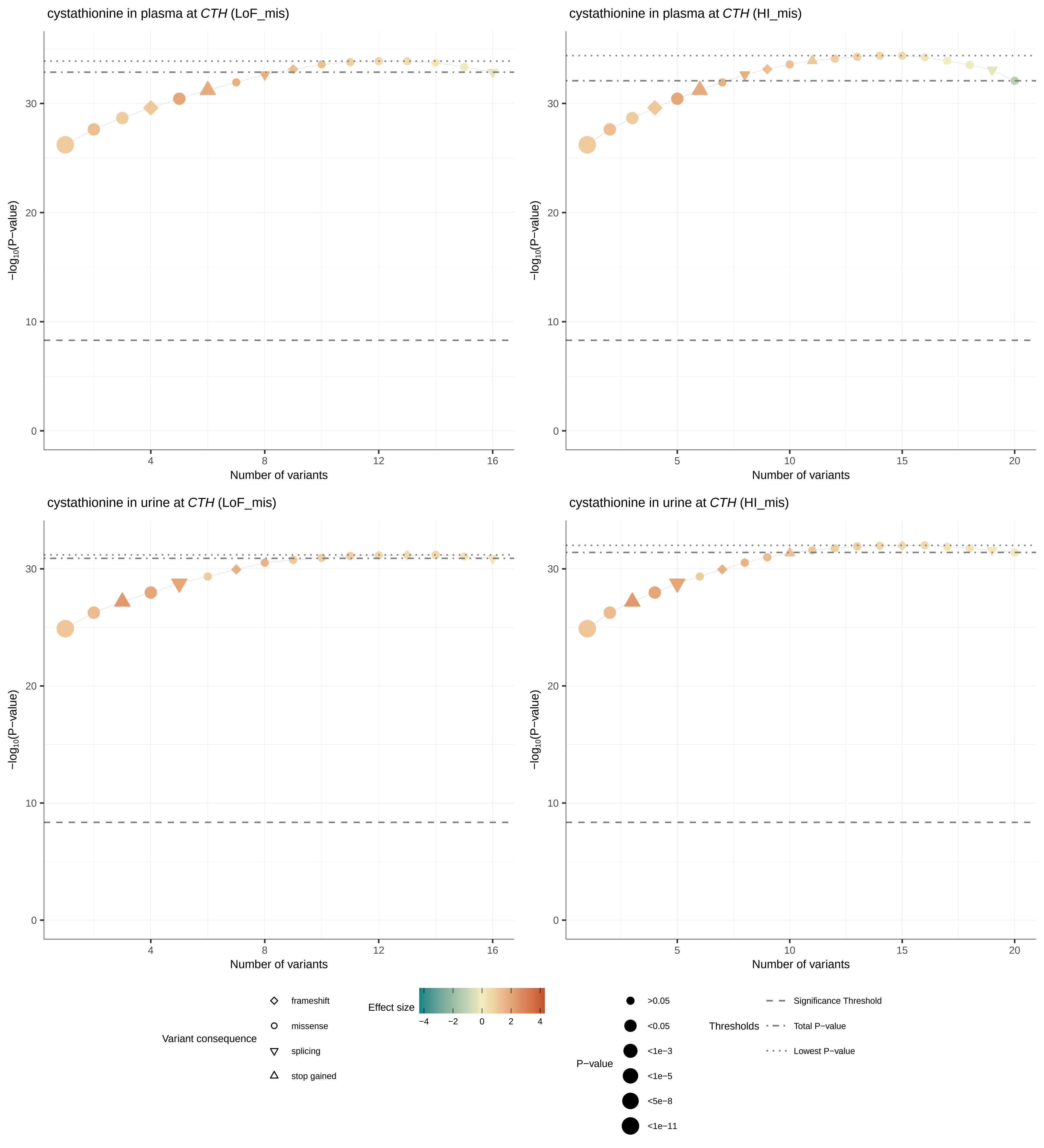
cystathionine in plasma at CTH (LoF_mis)
cystathionine in plasma at CTH (HI_mis)
cystathionine in urine at CTH (LoF_mis)
cystathionine in urine at CTH (HI_mis)
−log10(P−value)
Number of variants
Variant consequence
frameshift
missense
splicing
stop gained
Effect size
P−value
>0.05
<0.05
<1e−3
<1e−5
<5e−8
<1e−11
Thresholds
Significance Threshold
Total P−value
Lowest P−value

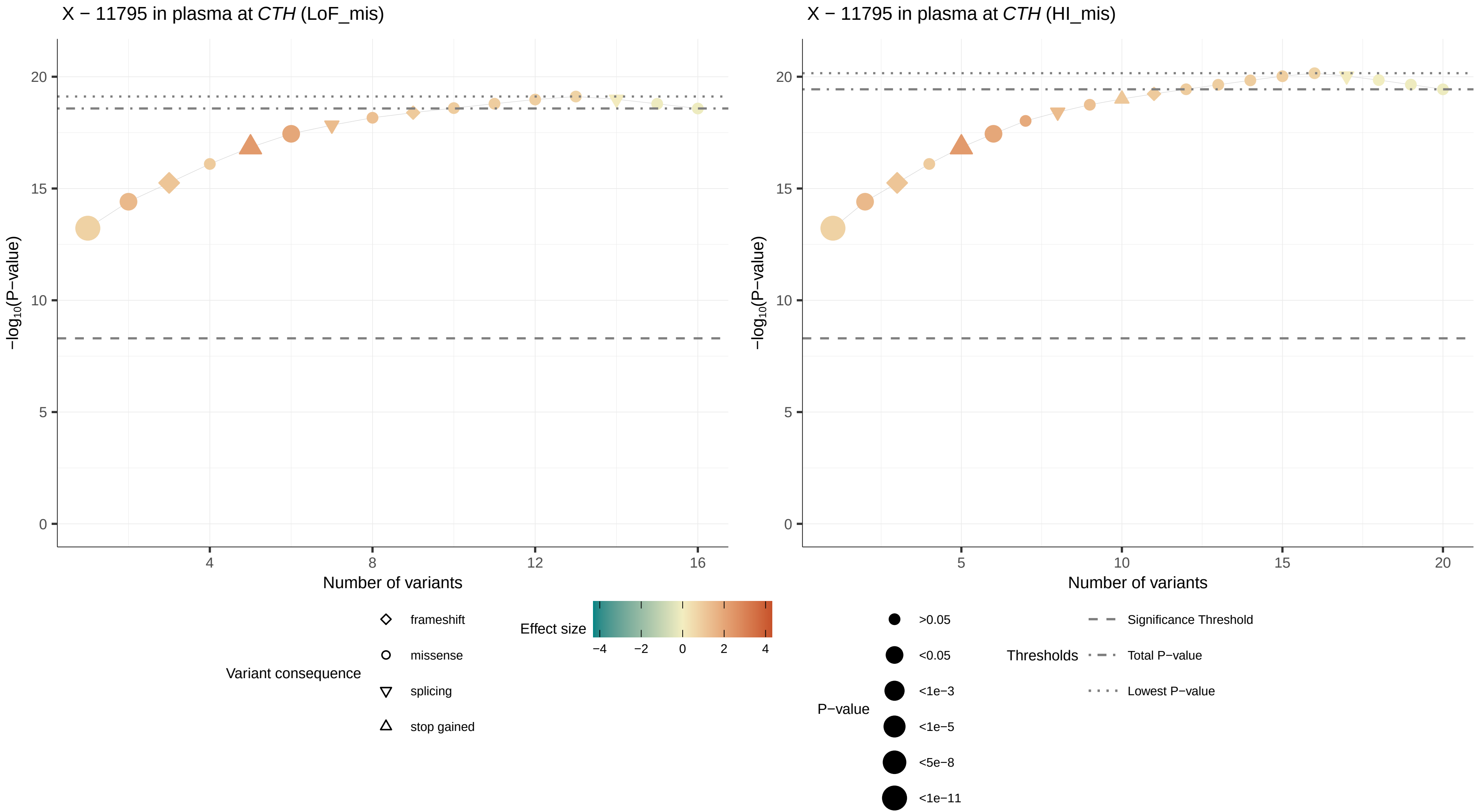

X − 11795 in plasma at *CTH* (LoF_mis)
X − 11795 in plasma at *CTH* (HI_mis)
−log10(P−value)
Number of variants
Variant consequence
frameshift
missense
splicing
stop gained
Effect size
P−value
>0.05
<0.05
<1e−3
<1e−5
<5e−8
<1e−11
Thresholds
Significance Threshold
Total P−value
Lowest P−value

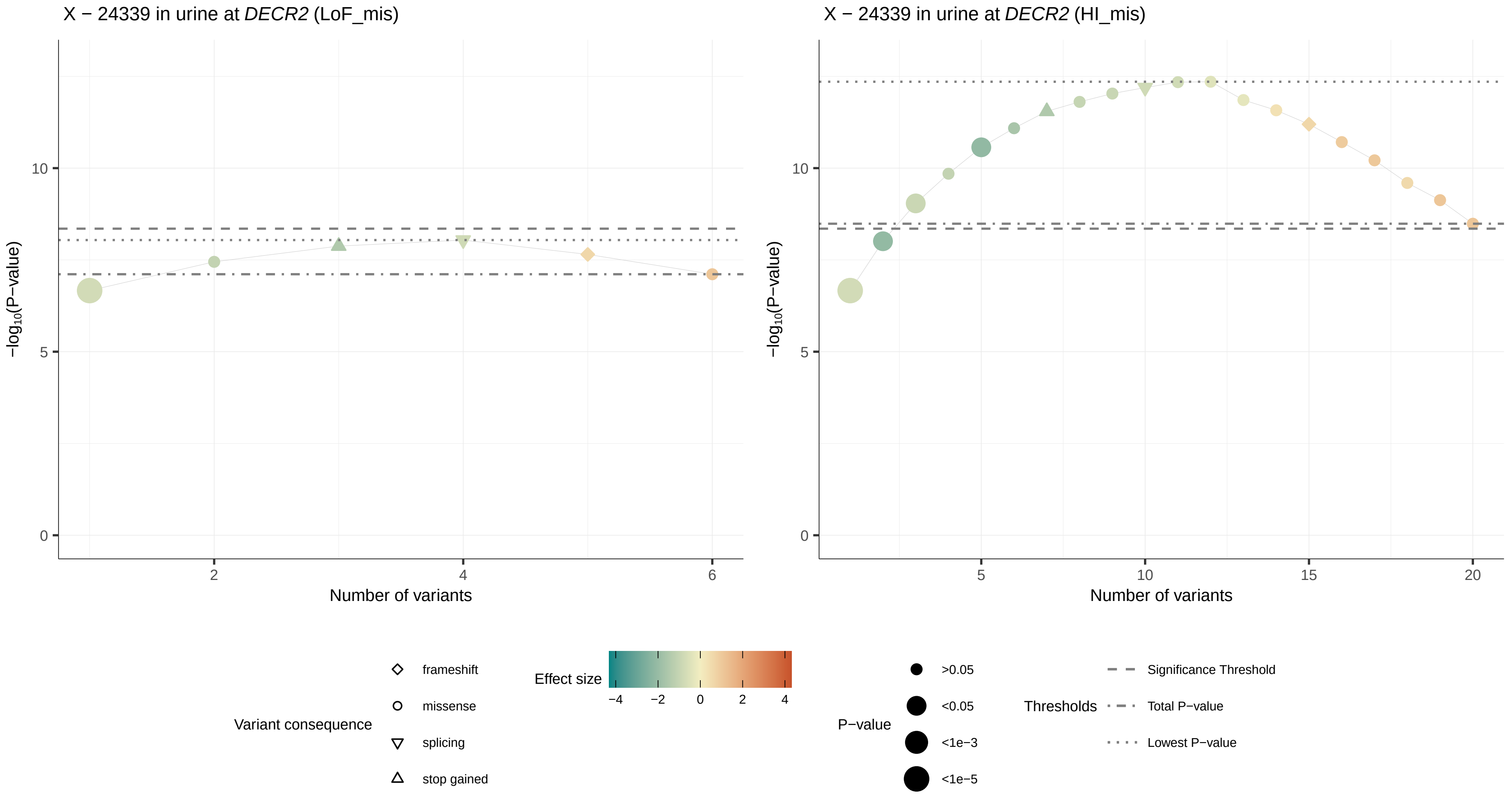
X − 24339 in urine at DECR2 (LoF_mis)
X − 24339 in urine at DECR2 (HI_mis)
−log10(P−value)
Number of variants
0
5
10
2
4
6
15
20
Variant consequence
frameshift
missense
splicing
stop gained
Effect size
−4
−2
0
2
4
P−value
>0.05
<0.05
<1e−3
<1e−5
Thresholds
Significance Threshold
Total P−value
Lowest P−value

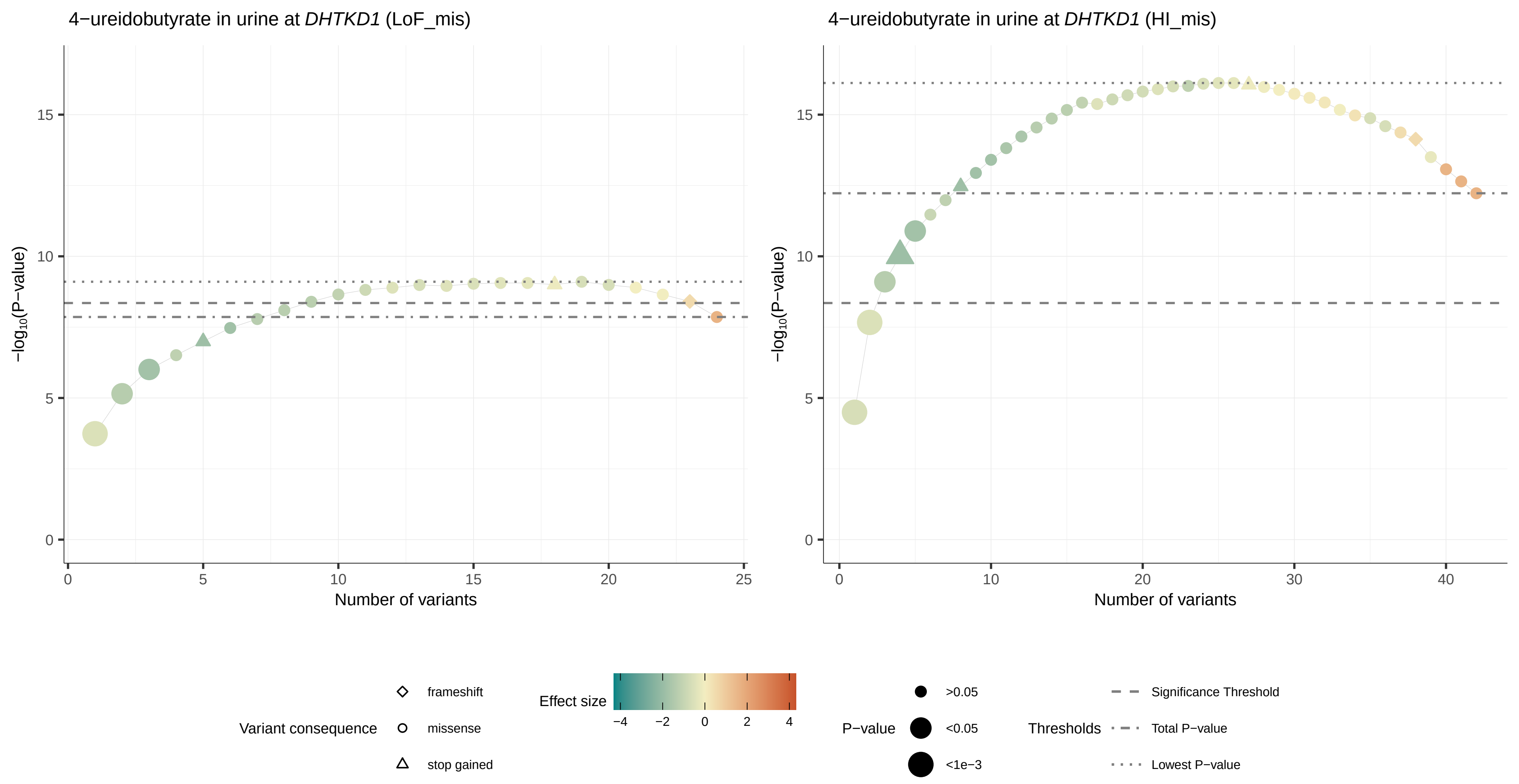
4−ureidobutyrate in urine at DHTKD1 (LoF_mis)
4−ureidobutyrate in urine at DHTKD1 (HI_mis)
−log10(P−value)
Number of variants
Variant consequence
frameshift
missense
stop gained
Effect size
−4
−2
0
2
4
P−value
>0.05
<0.05
<1e−3
Thresholds
Significance Threshold
Total P−value
Lowest P−value

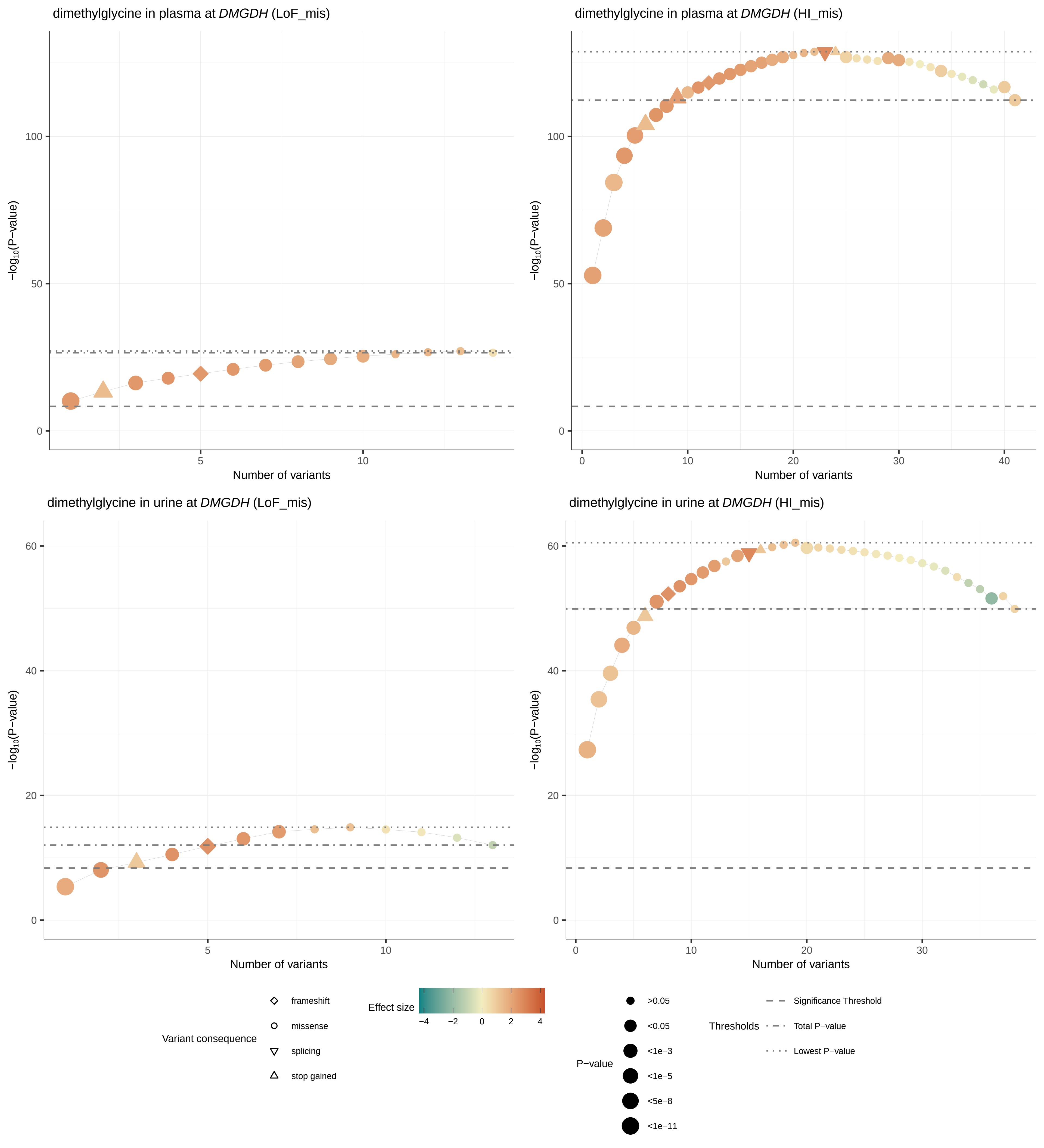

dimethylglycine in plasma at DMGDH (LoF_mis)
dimethylglycine in plasma at DMGDH (HI_mis)
dimethylglycine in urine at DMGDH (LoF_mis)
dimethylglycine in urine at DMGDH (HI_mis)
−log10(P−value)
Number of variants
Variant consequence
frameshift
missense
splicing
stop gained
Effect size
P−value
>0.05
<0.05
<1e−3
<1e−5
<5e−8
<1e−11
Thresholds
Significance Threshold
Total P−value
Lowest P−value

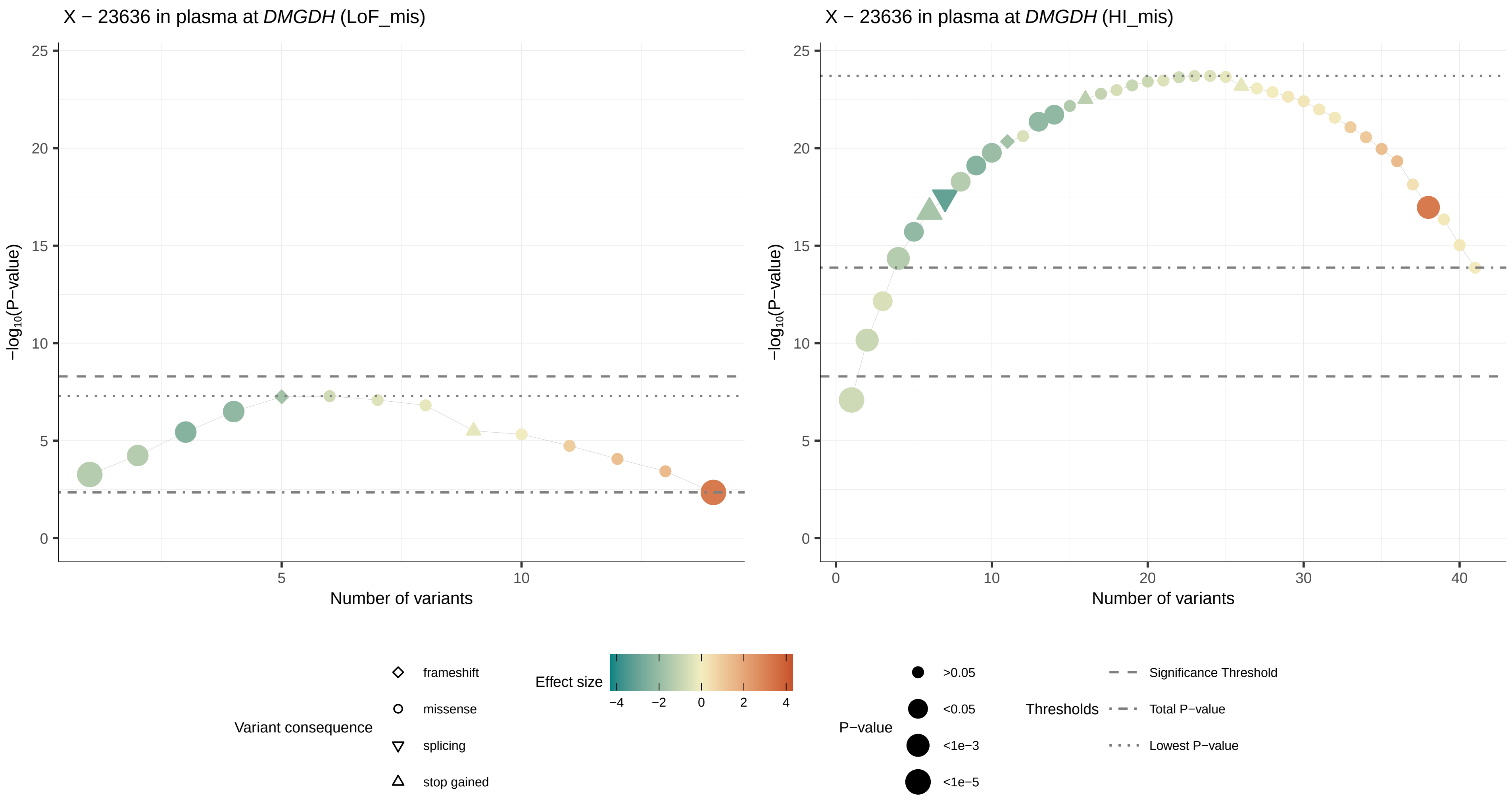
X − 23636 in plasma at *DMGDH* (LoF_mis)
X − 23636 in plasma at *DMGDH* (HI_mis)
−log10(P−value)
Number of variants
Variant consequence
frameshift
missense
splicing
stop gained
Effect size
−4
−2
0
2
4
P−value
>0.05
<0.05
<1e−3
<1e−5
Thresholds
Significance Threshold
Total P−value
Lowest P−value

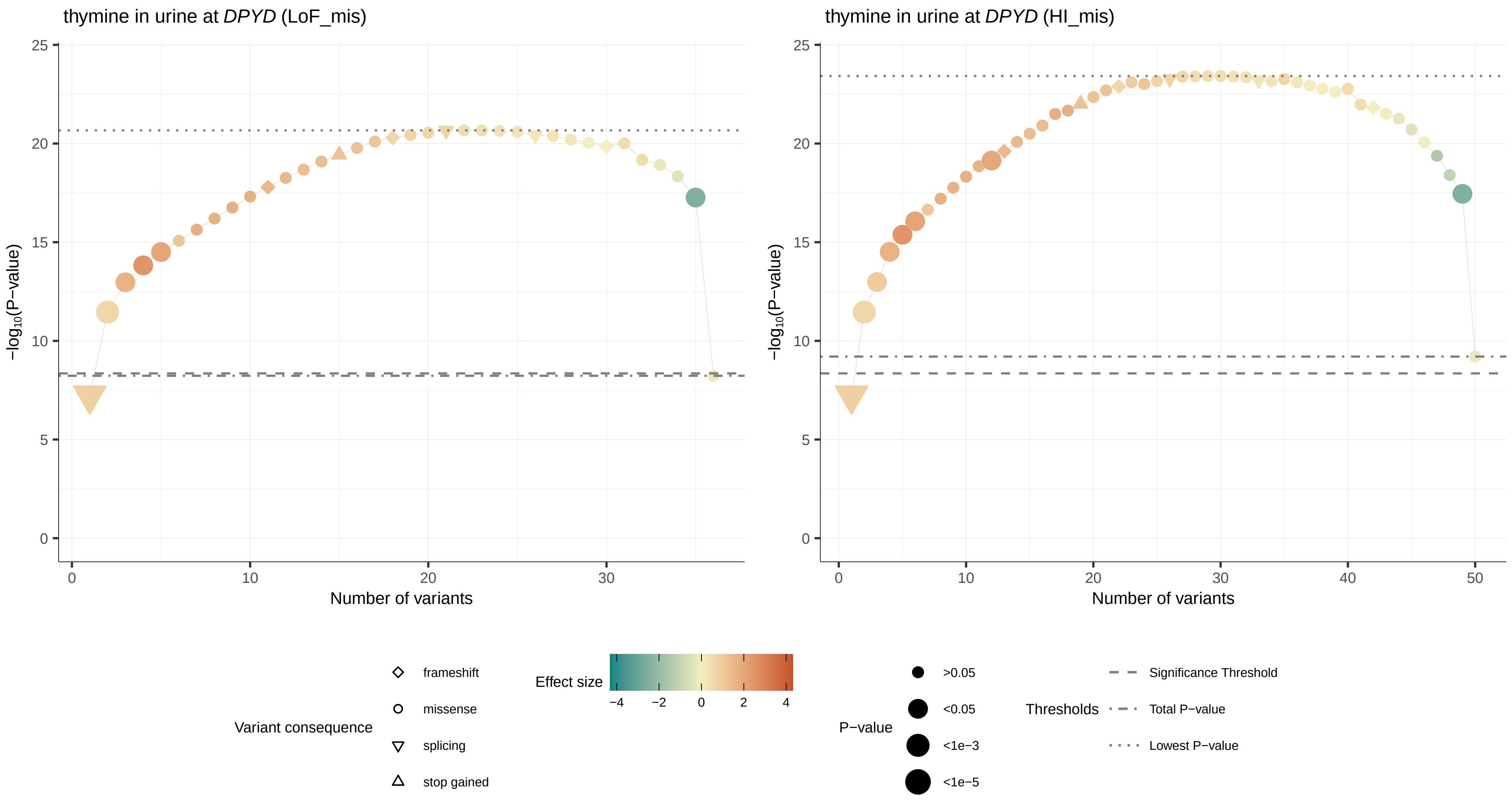

thymine in urine at DPYD (LoF_mis)
thymine in urine at DPYD (HI_mis)
−log10(P−value)
Number of variants
Variant consequence
frameshift
missense
splicing
stop gained
Effect size
P−value
>0.05
<0.05
<1e−3
<1e−5
Thresholds
Significance Threshold
Total P−value
Lowest P−value

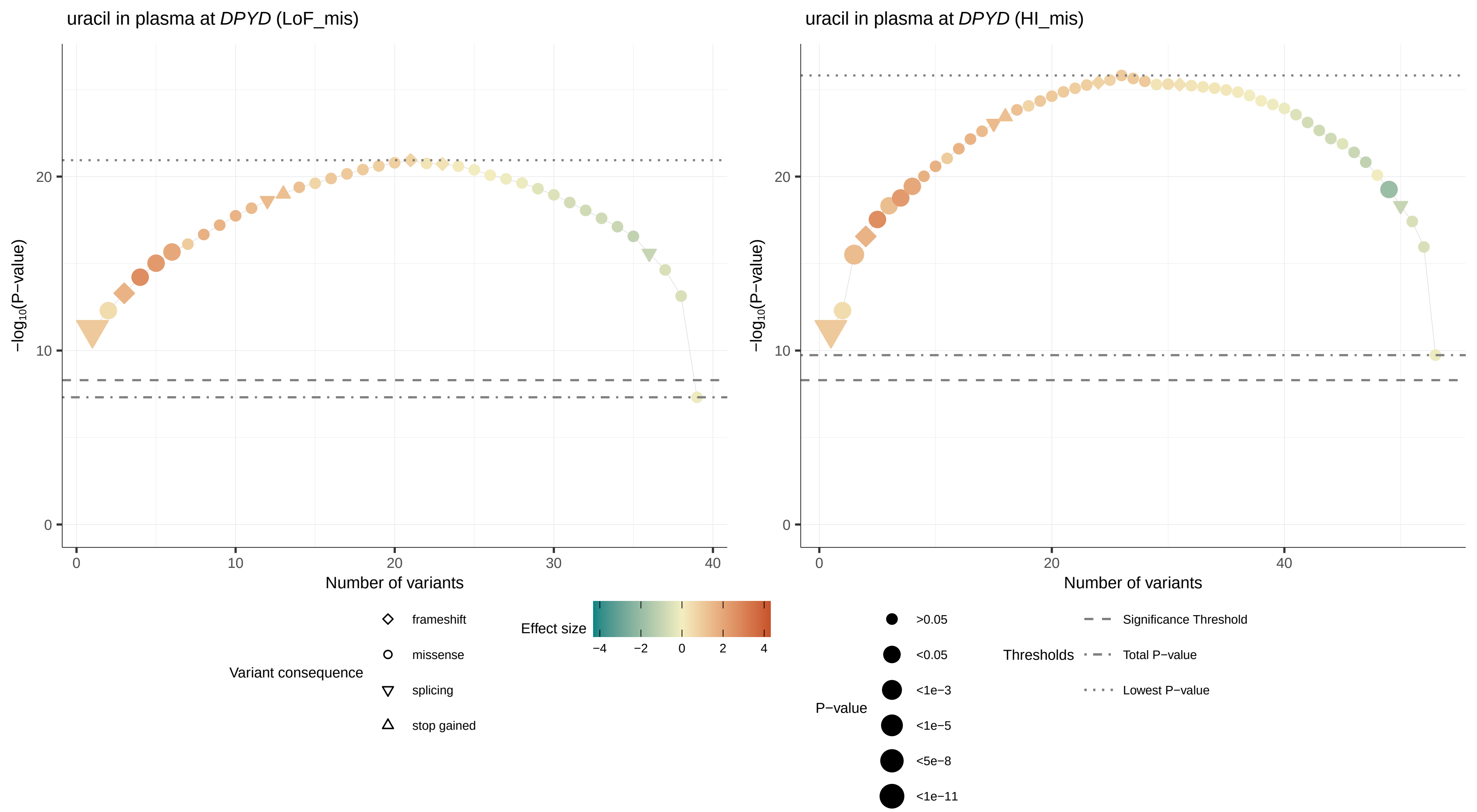

uracil in plasma at DPYD (LoF_mis)
uracil in plasma at DPYD (HI_mis)
−log10(P−value)
Number of variants
Variant consequence
frameshift
missense
splicing
stop gained
Effect size
P−value
>0.05
<0.05
<1e−3
<1e−5
<5e−8
<1e−11
Thresholds
Significance Threshold
Total P−value
Lowest P−value

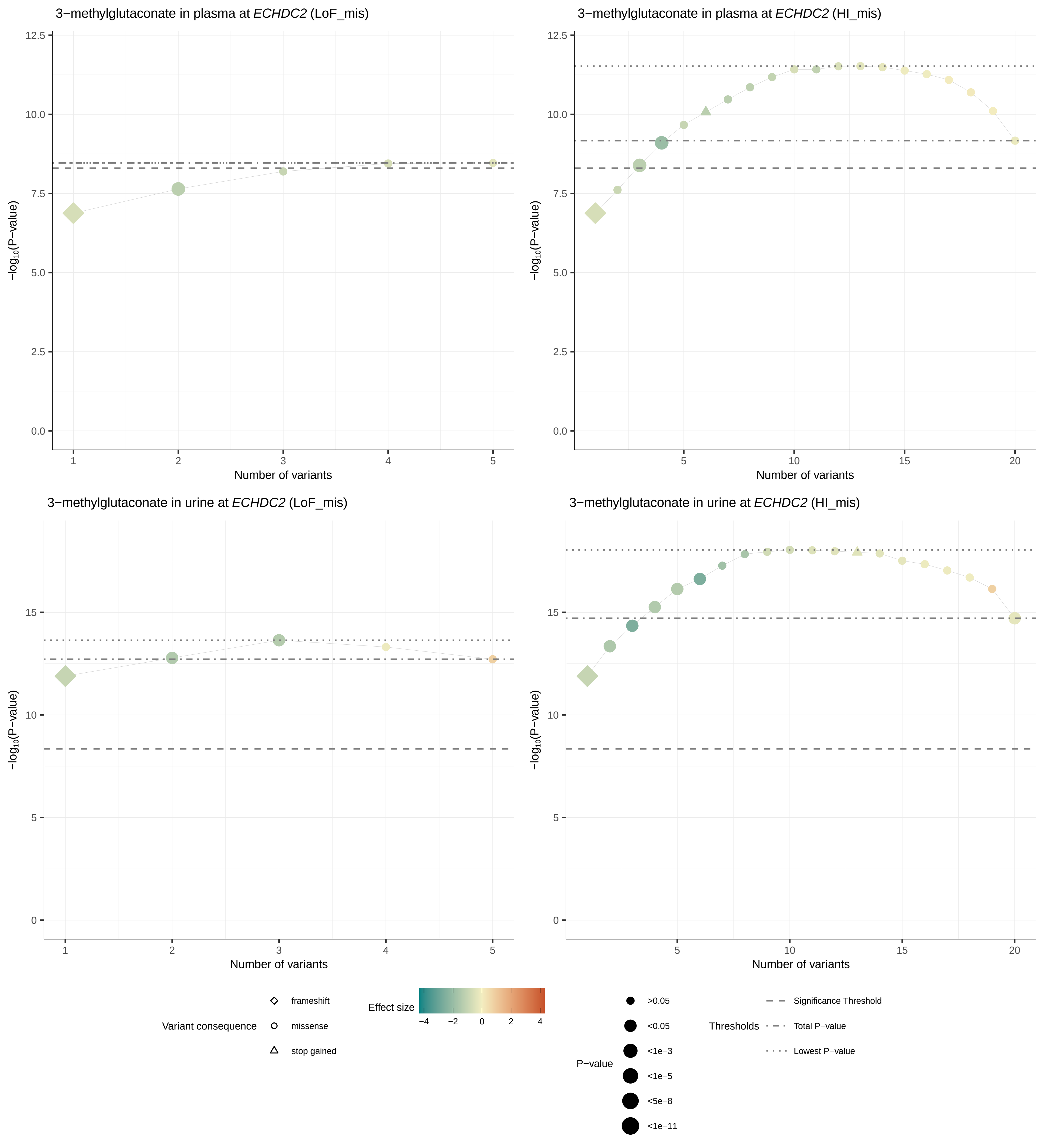
3−methylglutaconate in plasma at ECHDC2 (LoF_mis)
3−methylglutaconate in plasma at ECHDC2 (HI_mis)
3−methylglutaconate in urine at ECHDC2 (LoF_mis)
3−methylglutaconate in urine at ECHDC2 (HI_mis)
−log10(P−value)
Number of variants
Variant consequence
frameshift
missense
stop gained
Effect size
P−value
>0.05
<0.05
<1e−3
<1e−5
<5e−8
<1e−11
Thresholds
Significance Threshold
Total P−value
Lowest P−value

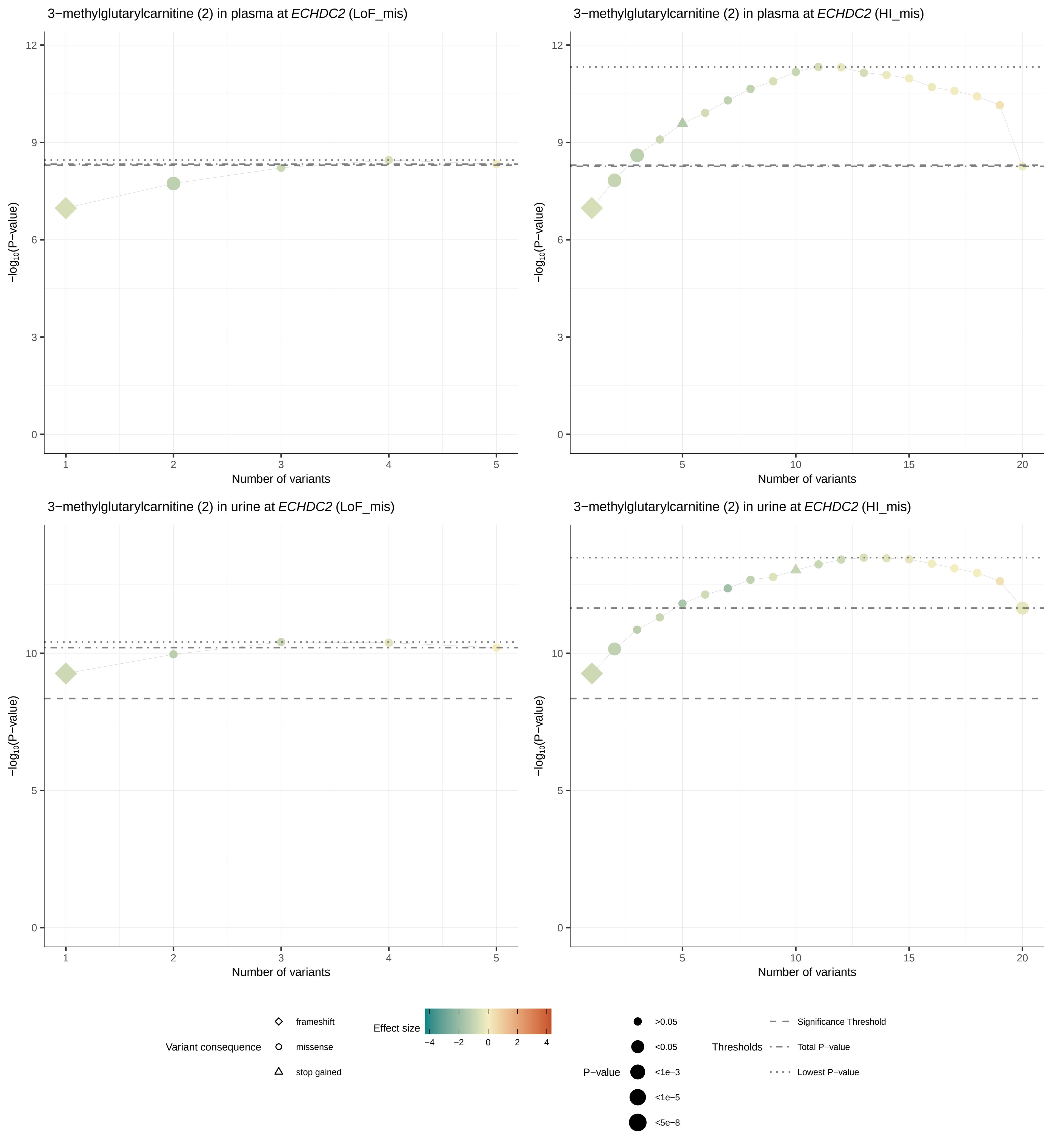
3−methylglutarylcarnitine (2) in plasma at ECHDC2 (LoF_mis)
3−methylglutarylcarnitine (2) in plasma at ECHDC2 (HI_mis)
3−methylglutarylcarnitine (2) in urine at ECHDC2 (LoF_mis)
3−methylglutarylcarnitine (2) in urine at ECHDC2 (HI_mis)
−log10(P−value)
Number of variants
Variant consequence
frameshift
missense
stop gained
Effect size
P−value
>0.05
<0.05
<1e−3
<1e−5
<5e−8
Thresholds
Significance Threshold
Total P−value
Lowest P−value

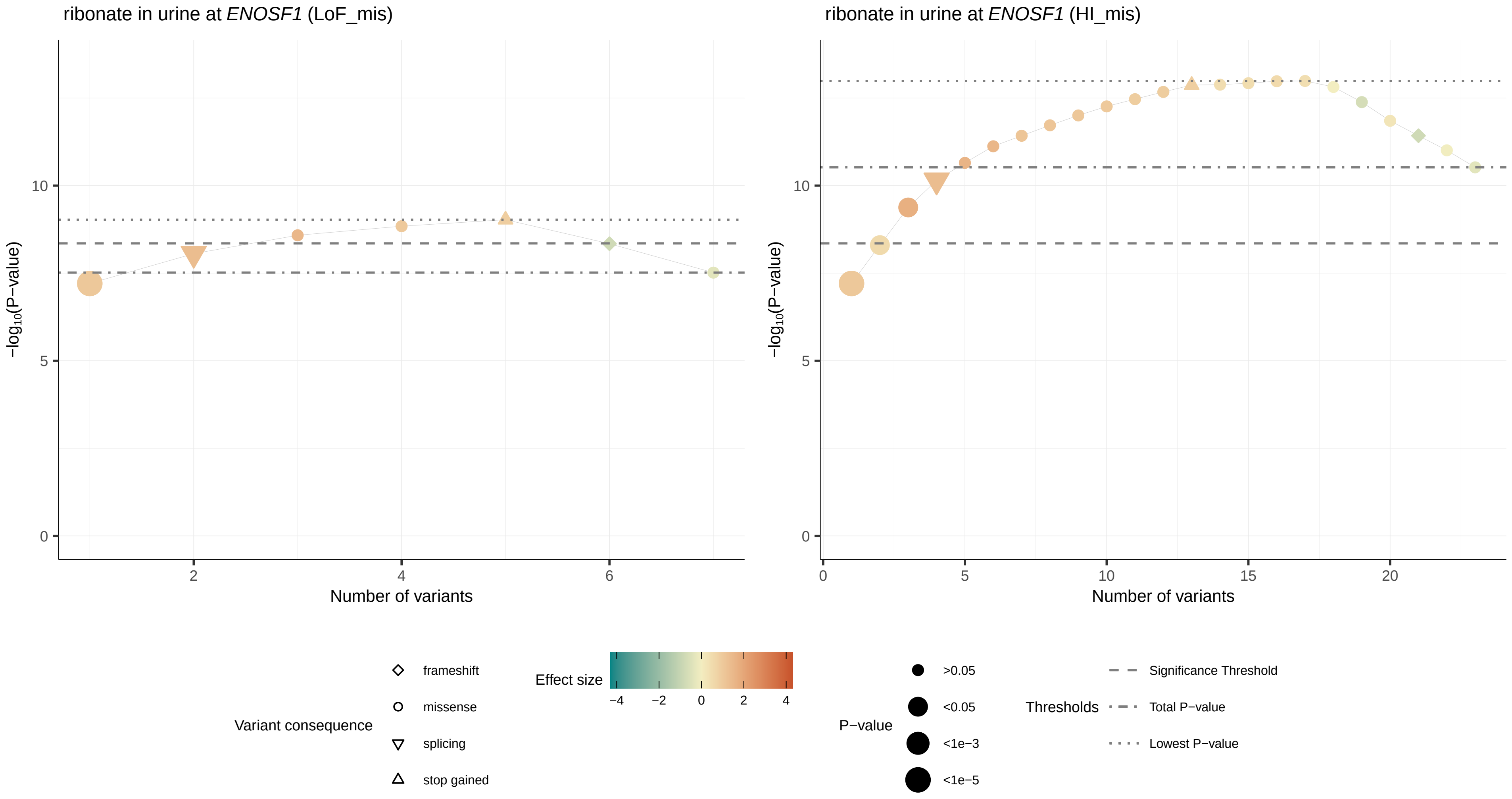

ribonate in urine at ENOSF1 (LoF_mis)
ribonate in urine at ENOSF1 (HI_mis)
−log10(P−value)
Number of variants
0
5
10
2
4
6
15
20
Variant consequence
frameshift
missense
splicing
stop gained
Effect size
−4
−2
0
2
4
P−value
>0.05
<0.05
<1e−3
<1e−5
Thresholds
Significance Threshold
Total P−value
Lowest P−value

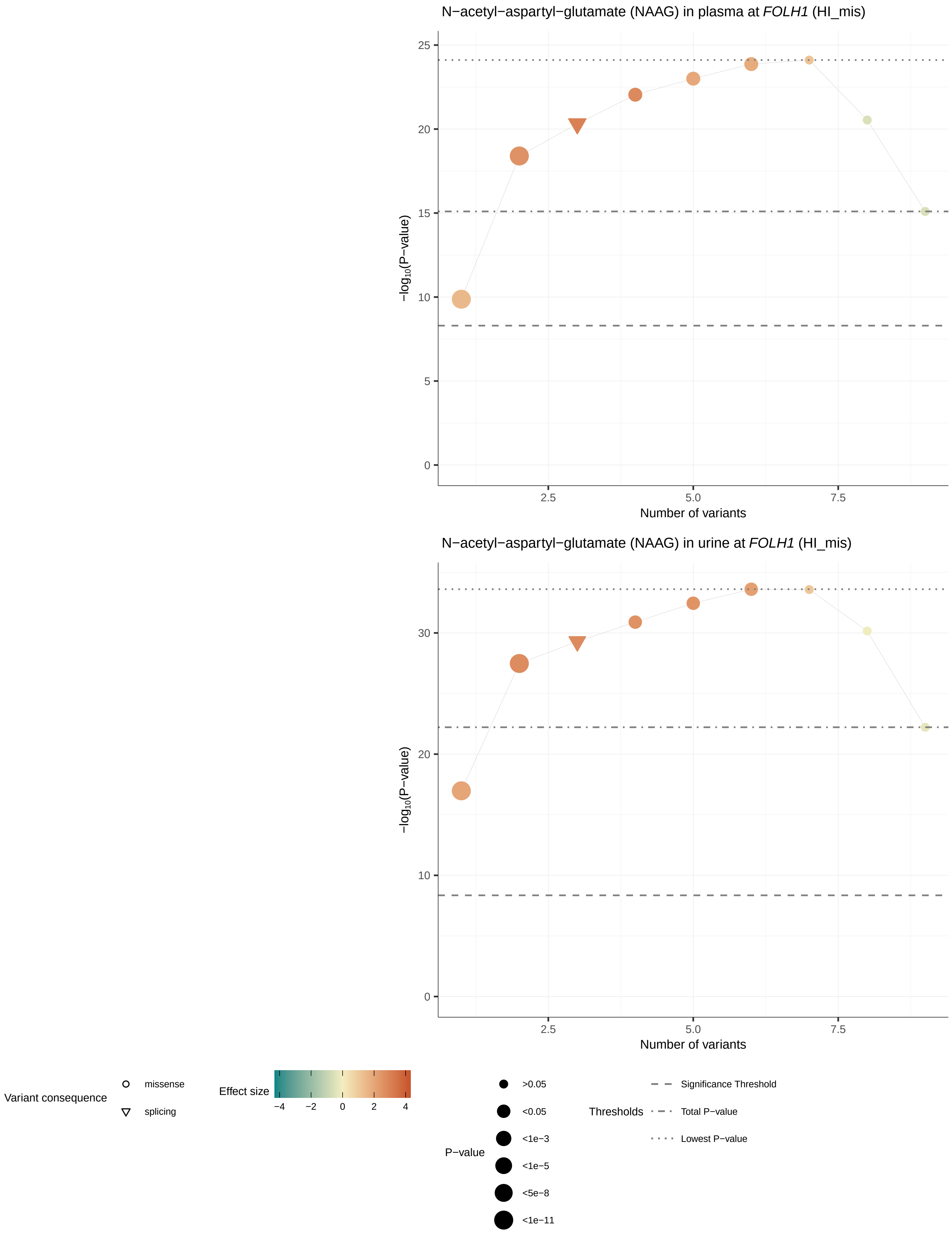

N−acetyl−aspartyl−glutamate (NAAG) in plasma at FOLH1 (HI_mis)
−log10(P−value)
Number of variants
N−acetyl−aspartyl−glutamate (NAAG) in urine at FOLH1 (HI_mis)
−log10(P−value)
Number of variants
Variant consequence
missense
splicing
Effect size
P−value
>0.05
<0.05
<1e−3
<1e−5
<5e−8
<1e−11
Thresholds
Significance Threshold
Total P−value
Lowest P−value

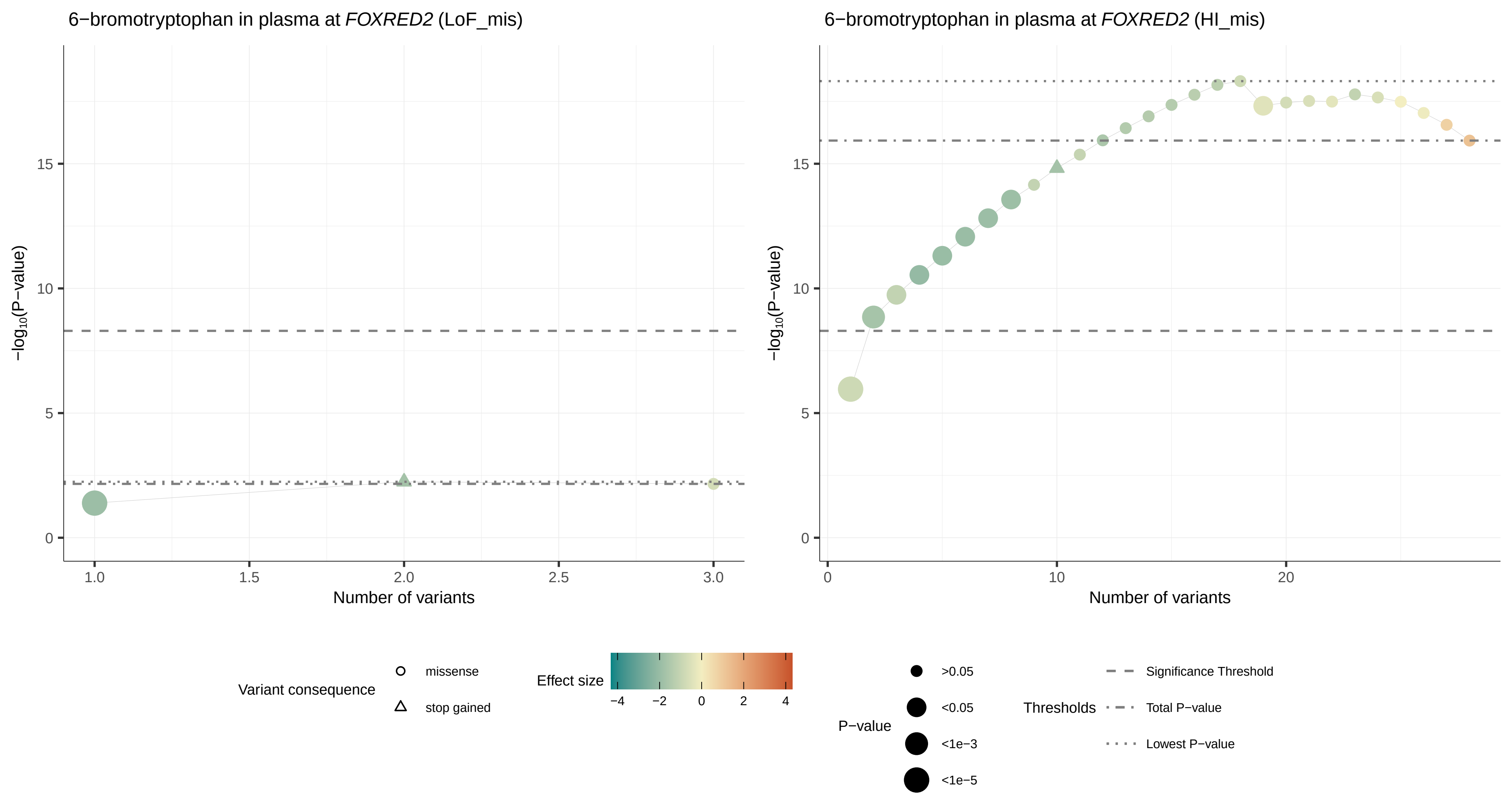
6−bromotryptophan in plasma at FOXRED2 (LoF_mis)
6−bromotryptophan in plasma at FOXRED2 (HI_mis)
−log10(P−value)
Number of variants
Variant consequence
missense
stop gained
Effect size
−4
−2
0
2
4
P−value
>0.05
<0.05
<1e−3
<1e−5
Thresholds
Significance Threshold
Total P−value
Lowest P−value

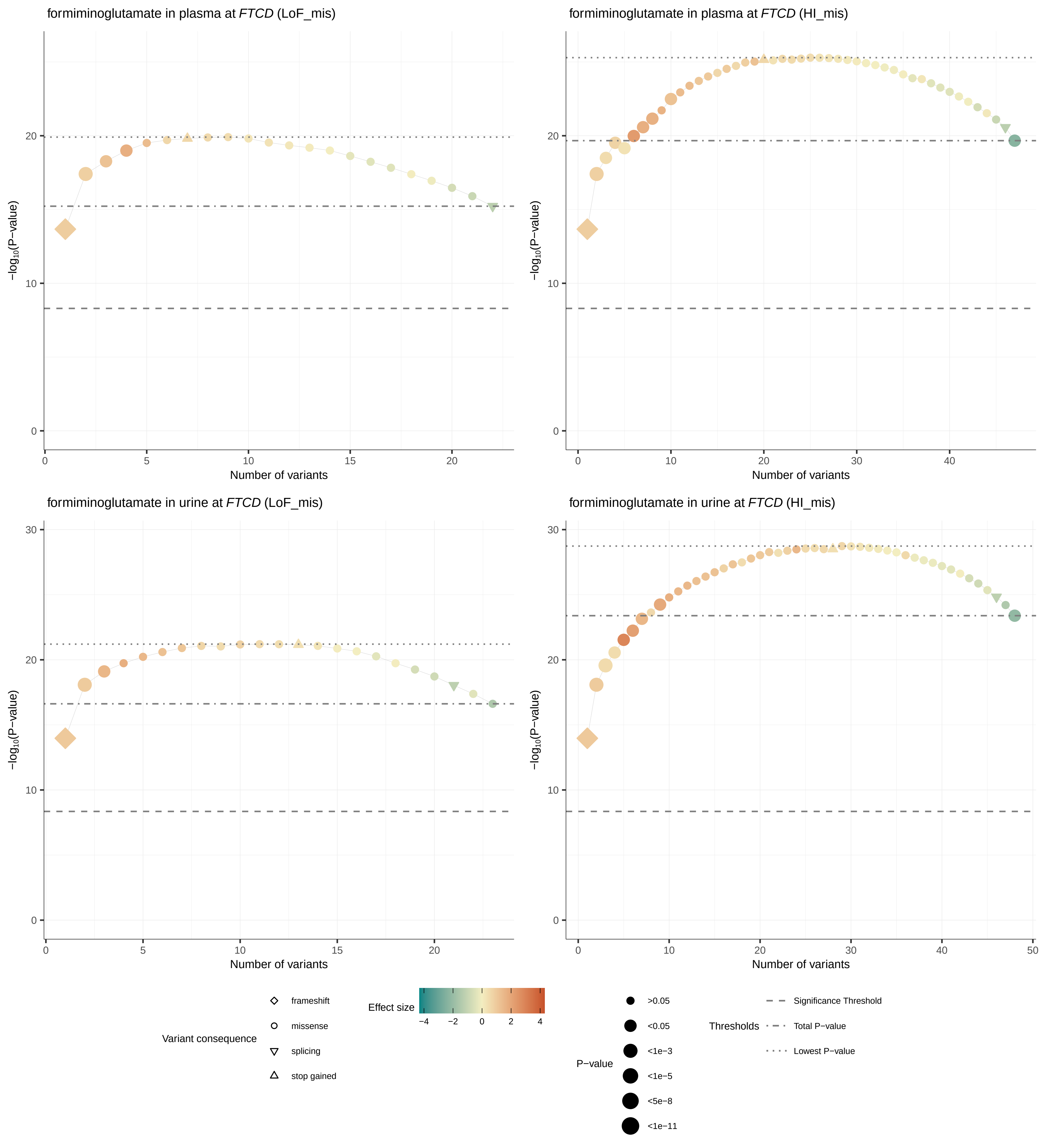
formiminoglutamate in plasma at FTCD (LoF_mis)
formiminoglutamate in plasma at FTCD (HI_mis)
formiminoglutamate in urine at FTCD (LoF_mis)
formiminoglutamate in urine at FTCD (HI_mis)
−log10(P−value)
Number of variants
Variant consequence
frameshift
missense
splicing
stop gained
Effect size
−4 −2 0 2 4
P−value
>0.05
<0.05
<1e−3
<1e−5
<5e−8
<1e−11
Thresholds
Significance Threshold
Total P−value
Lowest P−value

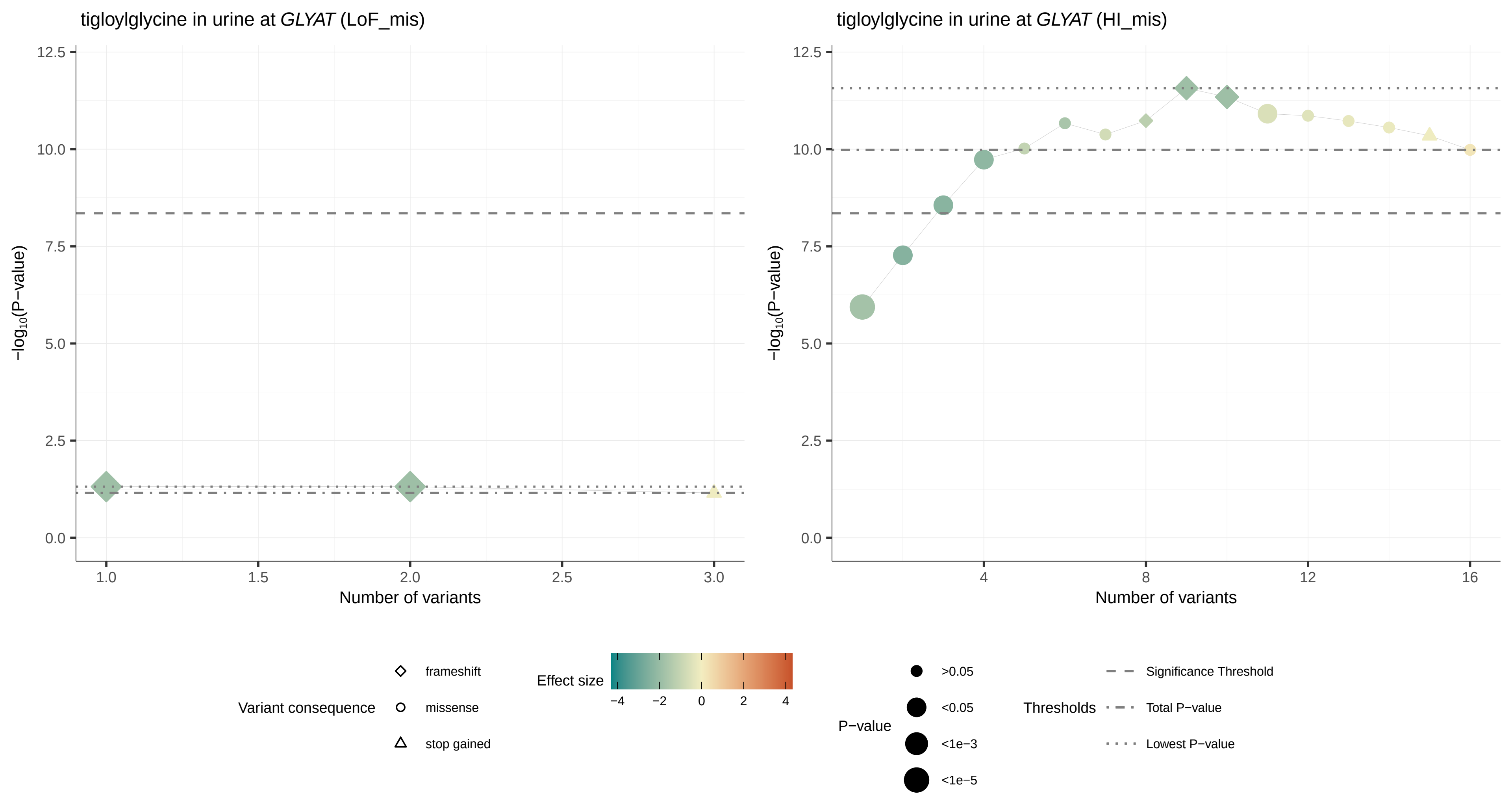
tigloylglycine in urine at GLYAT (LoF_mis)
tigloylglycine in urine at GLYAT (HI_mis)
−log10(P−value)
12.5
10.0
7.5
5.0
2.5
0.0
1.0
1.5
2.0
2.5
3.0
4
8
12
16
Number of variants
Variant consequence
frameshift
missense
stop gained
Effect size
−4
−2
0
2
4
P−value
>0.05
<0.05
<1e−3
<1e−5
Thresholds
Significance Threshold
Total P−value
Lowest P−value

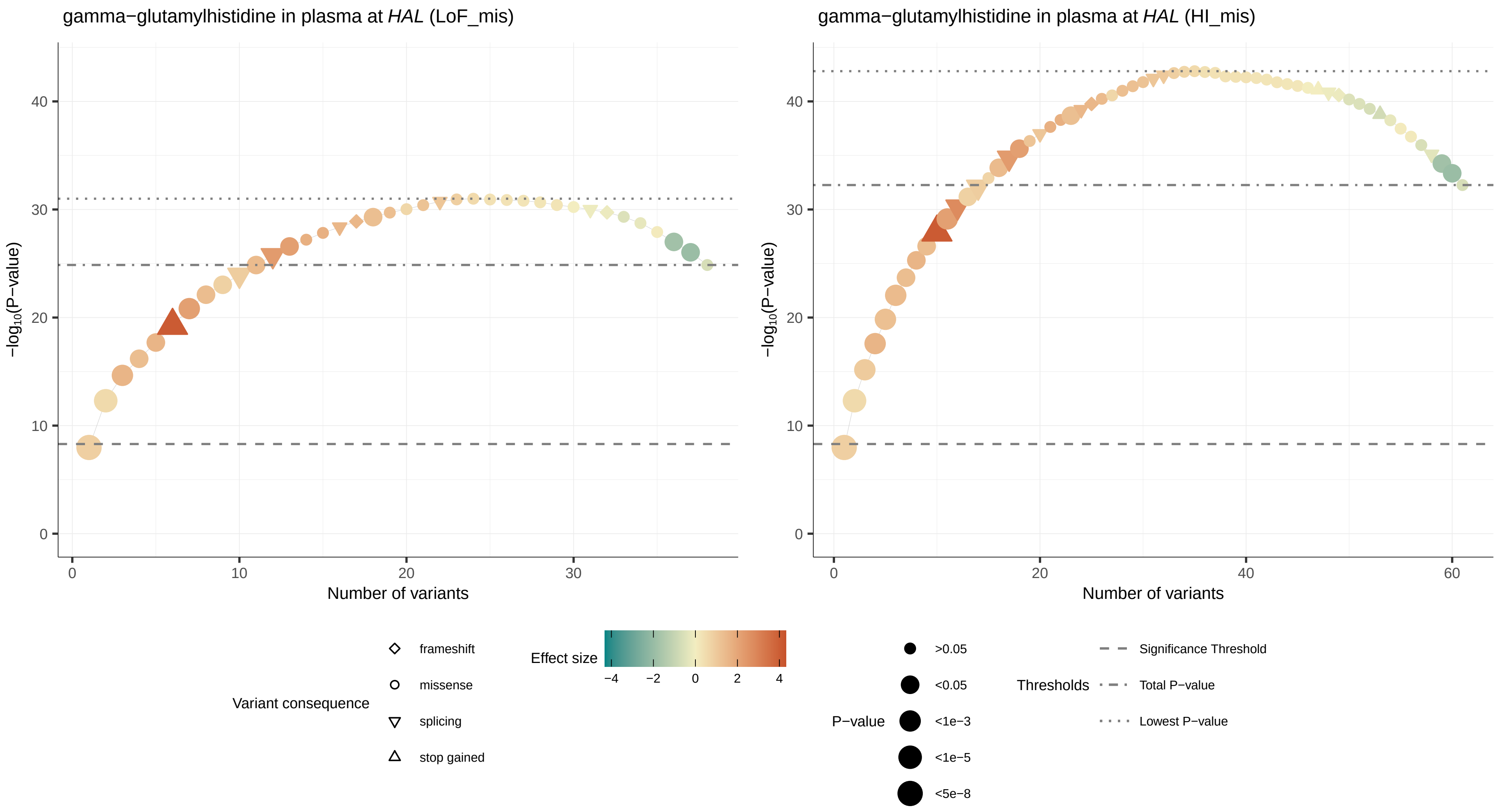

gamma−glutamylhistidine in plasma at *HAL* (LoF_mis)
gamma−glutamylhistidine in plasma at *HAL* (HI_mis)
−log10(P−value)
Number of variants
0
10
20
30
40
60
Variant consequence
frameshift
missense
splicing
stop gained
Effect size
−4
−2
0
2
4
P−value
>0.05
<0.05
<1e−3
<1e−5
<5e−8
Thresholds
Significance Threshold
Total P−value
Lowest P−value

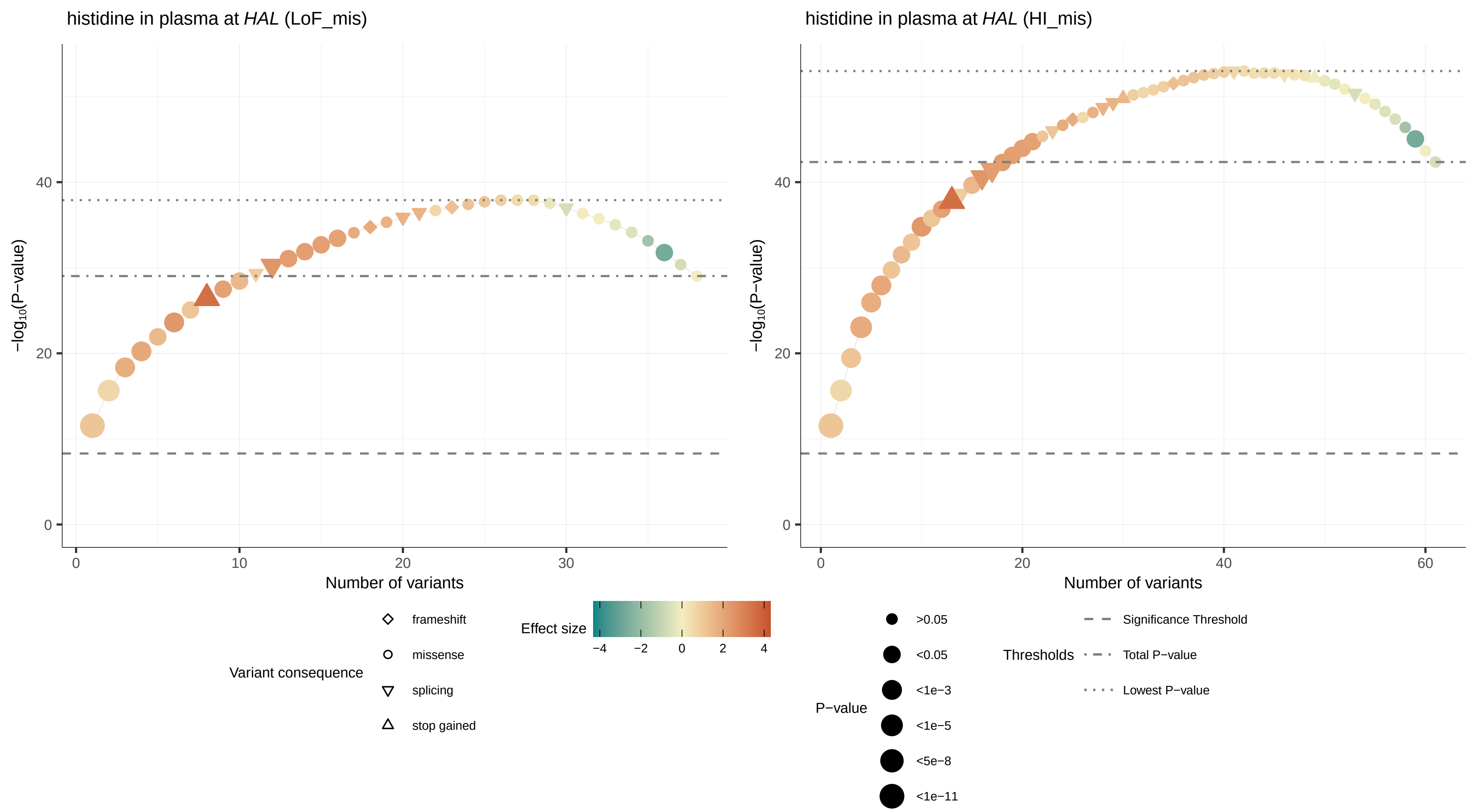
histidine in plasma at HAL (LoF_mis)
histidine in plasma at HAL (HI_mis)
−log10(P−value)
Number of variants
0
20
40
0
10
20
30
60
Variant consequence
frameshift
missense
splicing
stop gained
Effect size
−4
−2
0
2
4
P−value
>0.05
<0.05
<1e−3
<1e−5
<5e−8
<1e−11
Thresholds
Significance Threshold
Total P−value
Lowest P−value

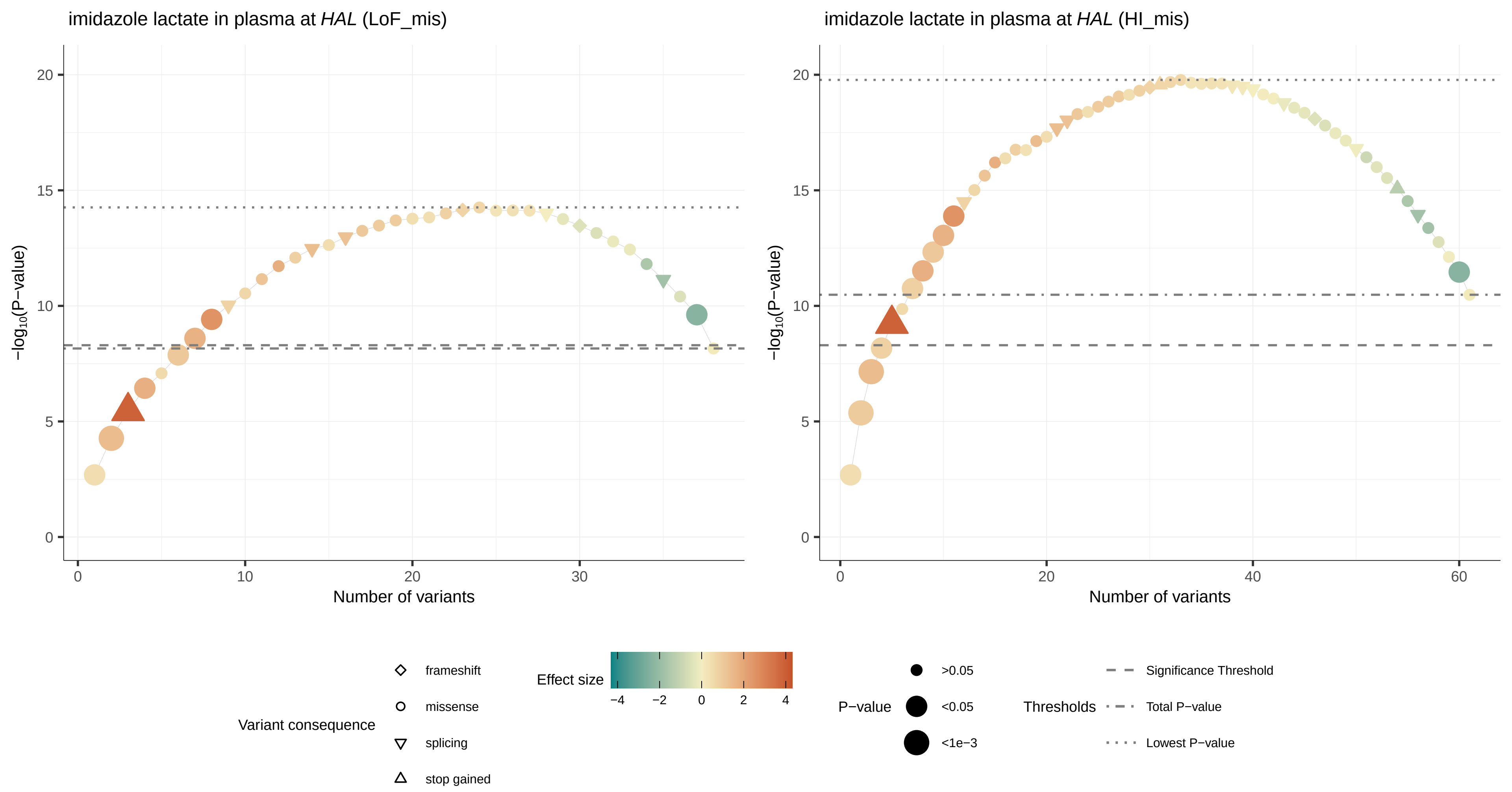

imidazole lactate in plasma at HAL (LoF_mis)
imidazole lactate in plasma at HAL (HI_mis)
−log10(P−value)
Number of variants
0
5
10
15
20
0
10
20
30
40
60
Variant consequence
frameshift
missense
splicing
stop gained
Effect size
−4
−2
0
2
4
P−value
>0.05
<0.05
<1e−3
Thresholds
Significance Threshold
Total P−value
Lowest P−value

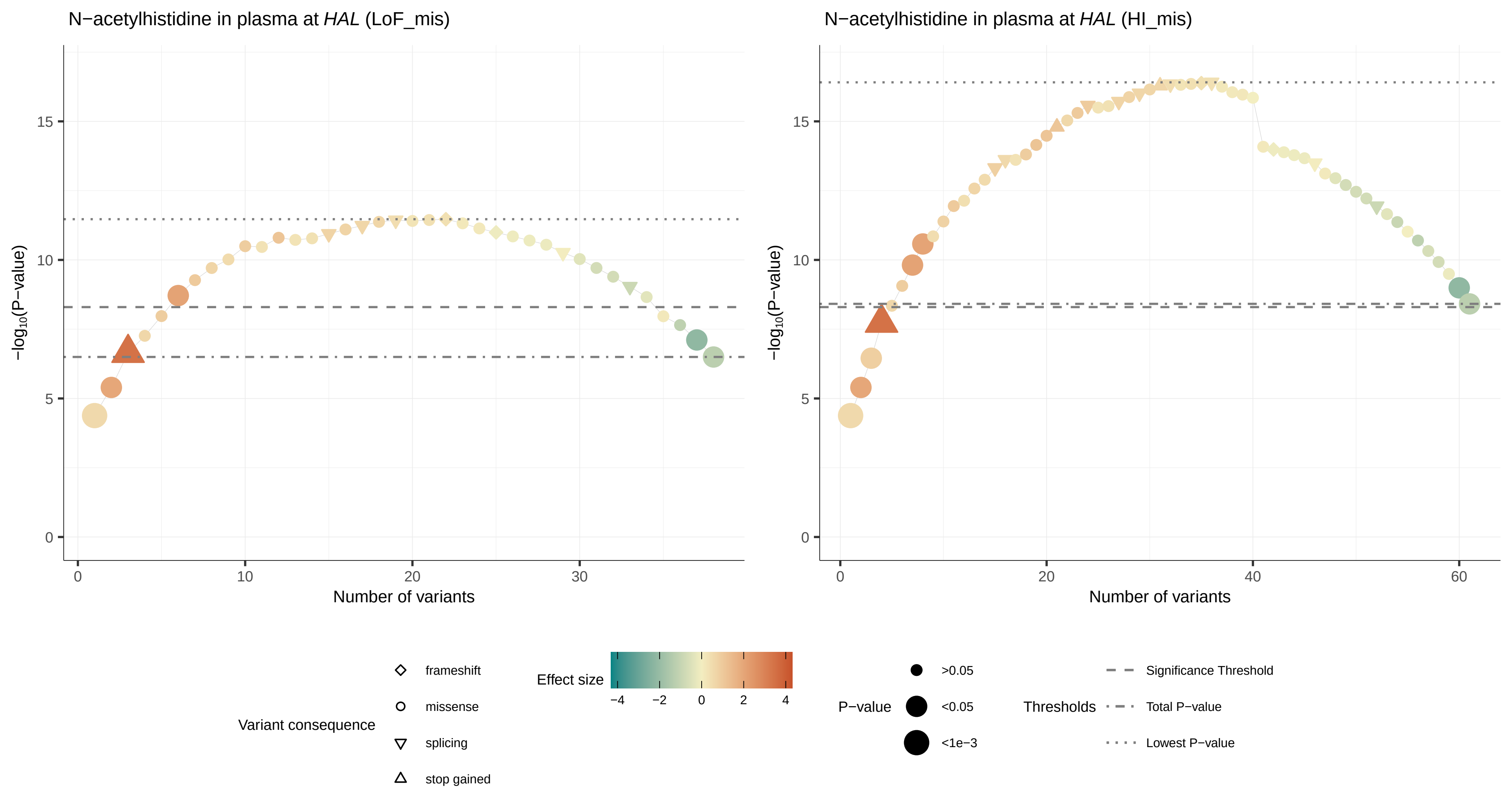

N−acetylhistidine in plasma at HAL (LoF_mis)
N−acetylhistidine in plasma at HAL (HI_mis)
−log10(P−value)
Number of variants
Variant consequence
frameshift
missense
splicing
stop gained
Effect size
P−value
>0.05
<0.05
<1e−3
Thresholds
Significance Threshold
Total P−value
Lowest P−value

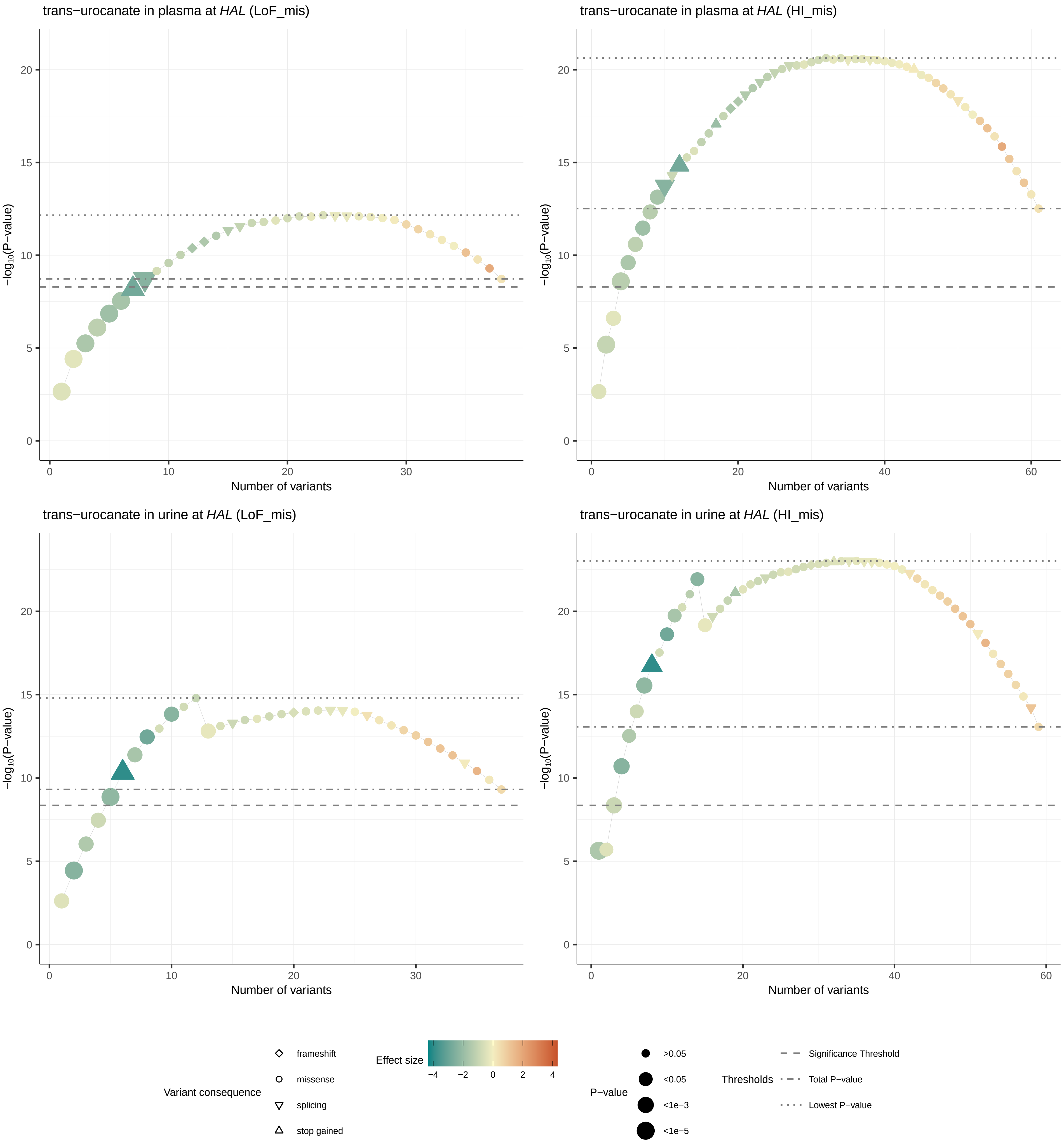

trans−urocanate in plasma at HAL (LoF_mis)
trans−urocanate in plasma at HAL (HI_mis)
trans−urocanate in urine at HAL (LoF_mis)
trans−urocanate in urine at HAL (HI_mis)
−log10(P−value)
Number of variants
Variant consequence
frameshift
missense
splicing
stop gained
Effect size
−4
−2
0
2
4
P−value
>0.05
<0.05
<1e−3
<1e−5
Thresholds
Significance Threshold
Total P−value
Lowest P−value

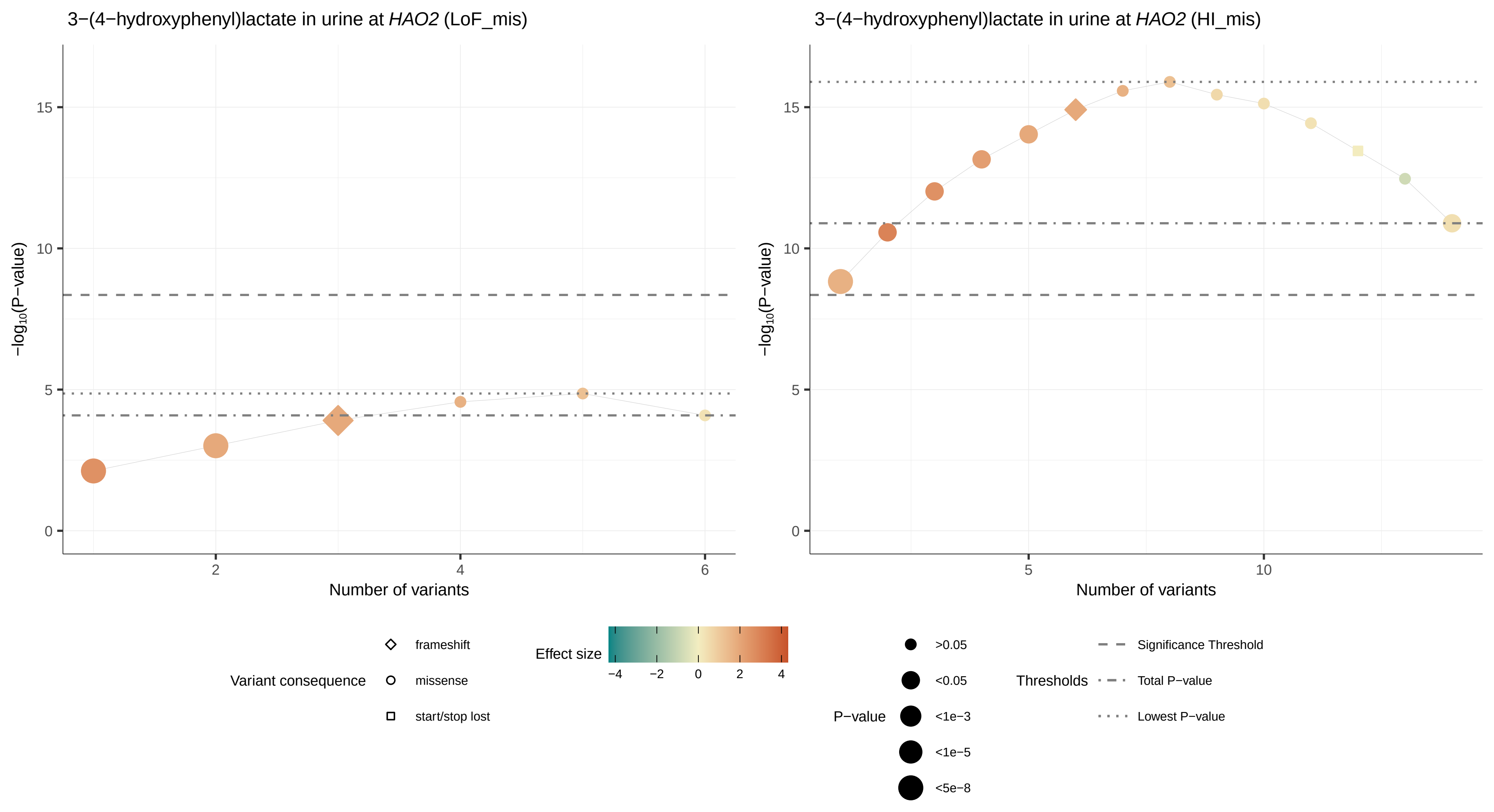

3−(4−hydroxyphenyl)lactate in urine at HAO2 (LoF_mis)
3−(4−hydroxyphenyl)lactate in urine at HAO2 (HI_mis)
−log10(P−value)
Number of variants
Variant consequence
frameshift
missense
start/stop lost
Effect size
−4
−2
0
2
4
P−value
>0.05
<0.05
<1e−3
<1e−5
<5e−8
Thresholds
Significance Threshold
Total P−value
Lowest P−value

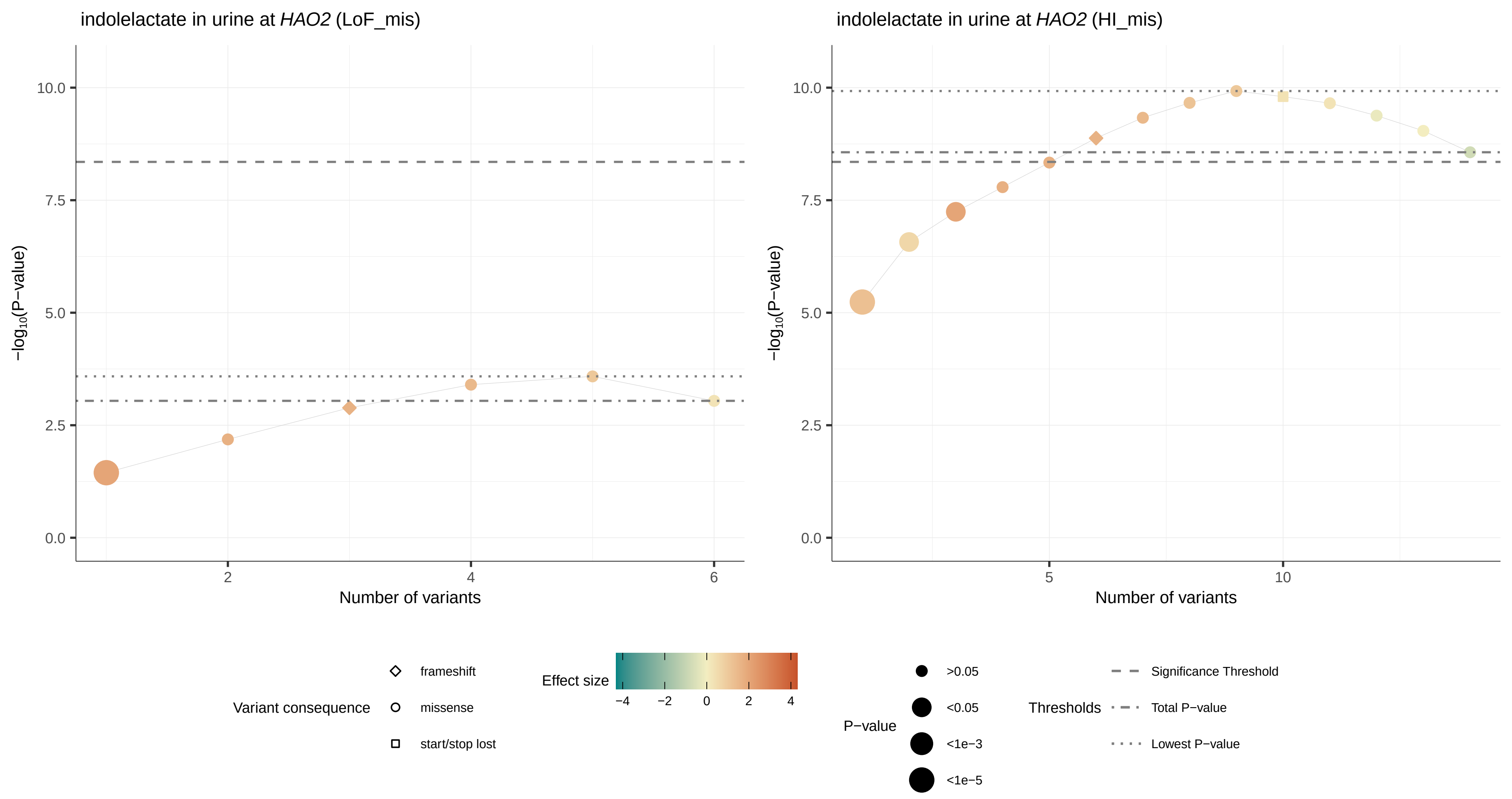

indolelactate in urine at HAO2 (LoF_mis)
indolelactate in urine at HAO2 (HI_mis)
−log10(P−value)
10.0
7.5
5.0
2.5
0.0
2
4
6
5
10
Number of variants
Variant consequence
frameshift
missense
start/stop lost
Effect size
−4
−2
0
2
4
P−value
>0.05
<0.05
<1e−3
<1e−5
Thresholds
Significance Threshold
Total P−value
Lowest P−value

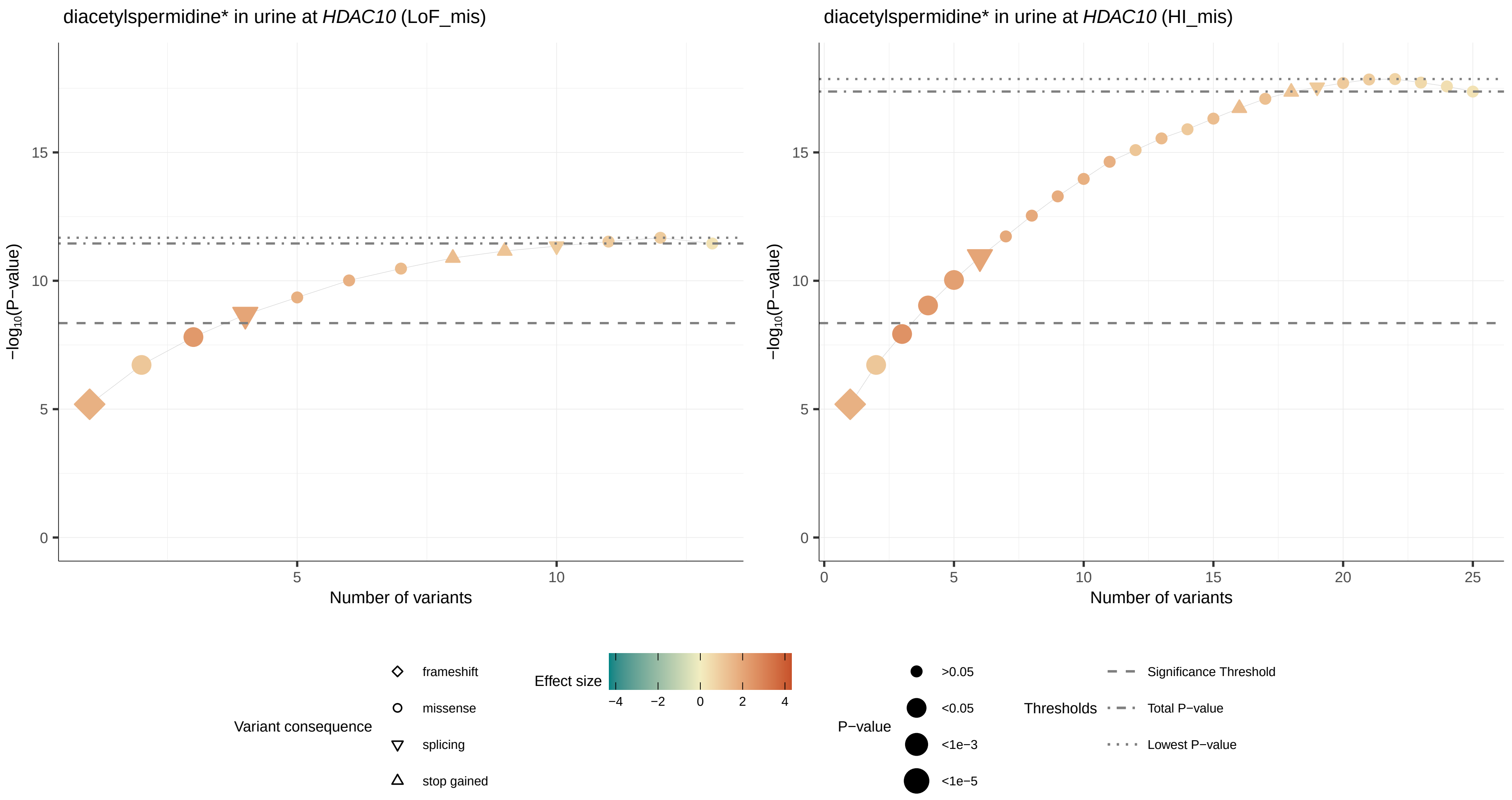
diacetylspermidine* in urine at HDAC10 (LoF_mis)
diacetylspermidine* in urine at HDAC10 (HI_mis)
−log10(P−value)
Number of variants
Variant consequence
frameshift
missense
splicing
stop gained
Effect size
P−value
>0.05
<0.05
<1e−3
<1e−5
Thresholds
Significance Threshold
Total P−value
Lowest P−value

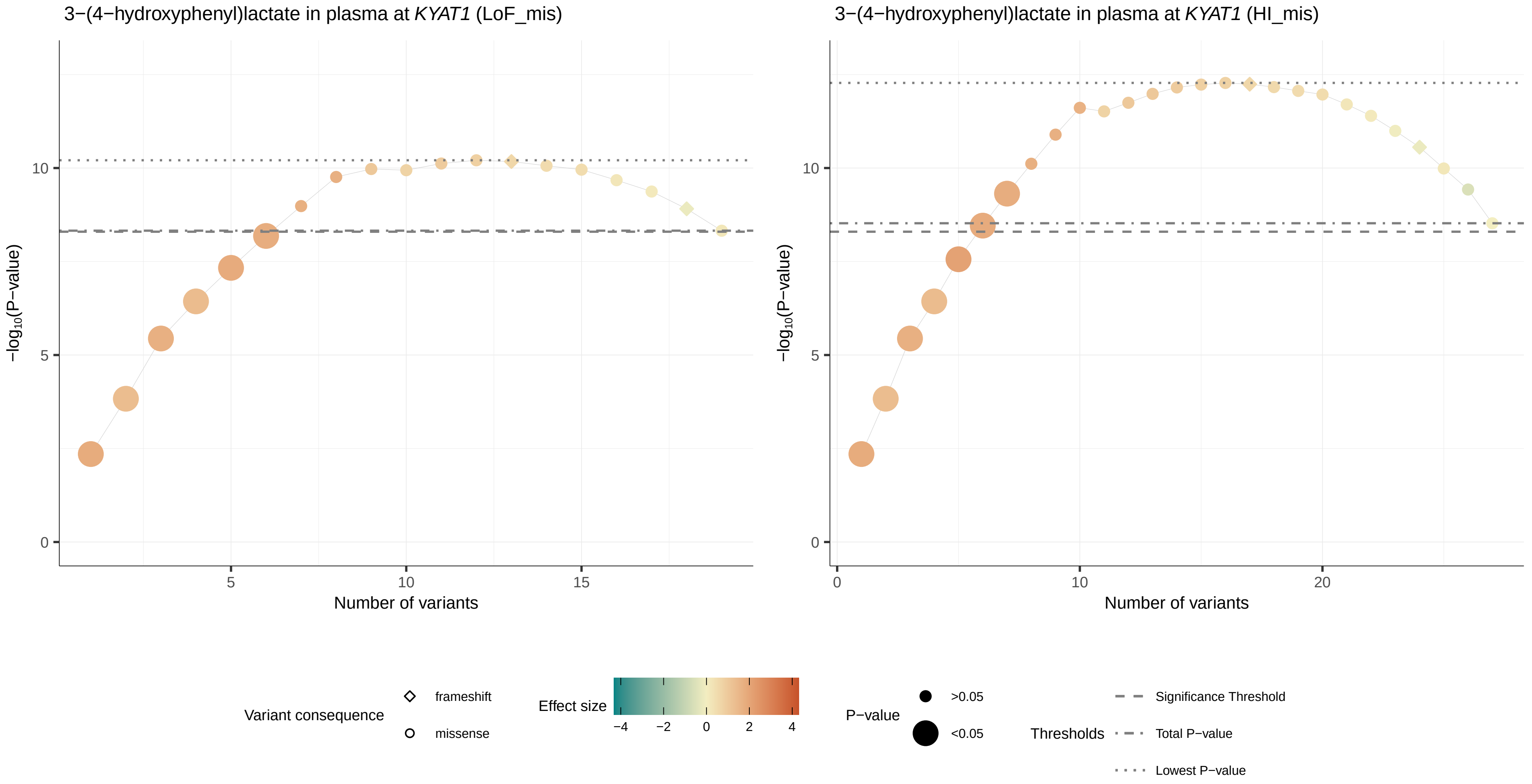

3−(4−hydroxyphenyl)lactate in plasma at KYAT1 (LoF_mis)
3−(4−hydroxyphenyl)lactate in plasma at KYAT1 (HI_mis)
−log10(P−value)
Number of variants
0
5
10
15
20
Variant consequence
frameshift
missense
Effect size
−4
−2
0
2
4
P−value
>0.05
<0.05
Thresholds
Significance Threshold
Total P−value
Lowest P−value

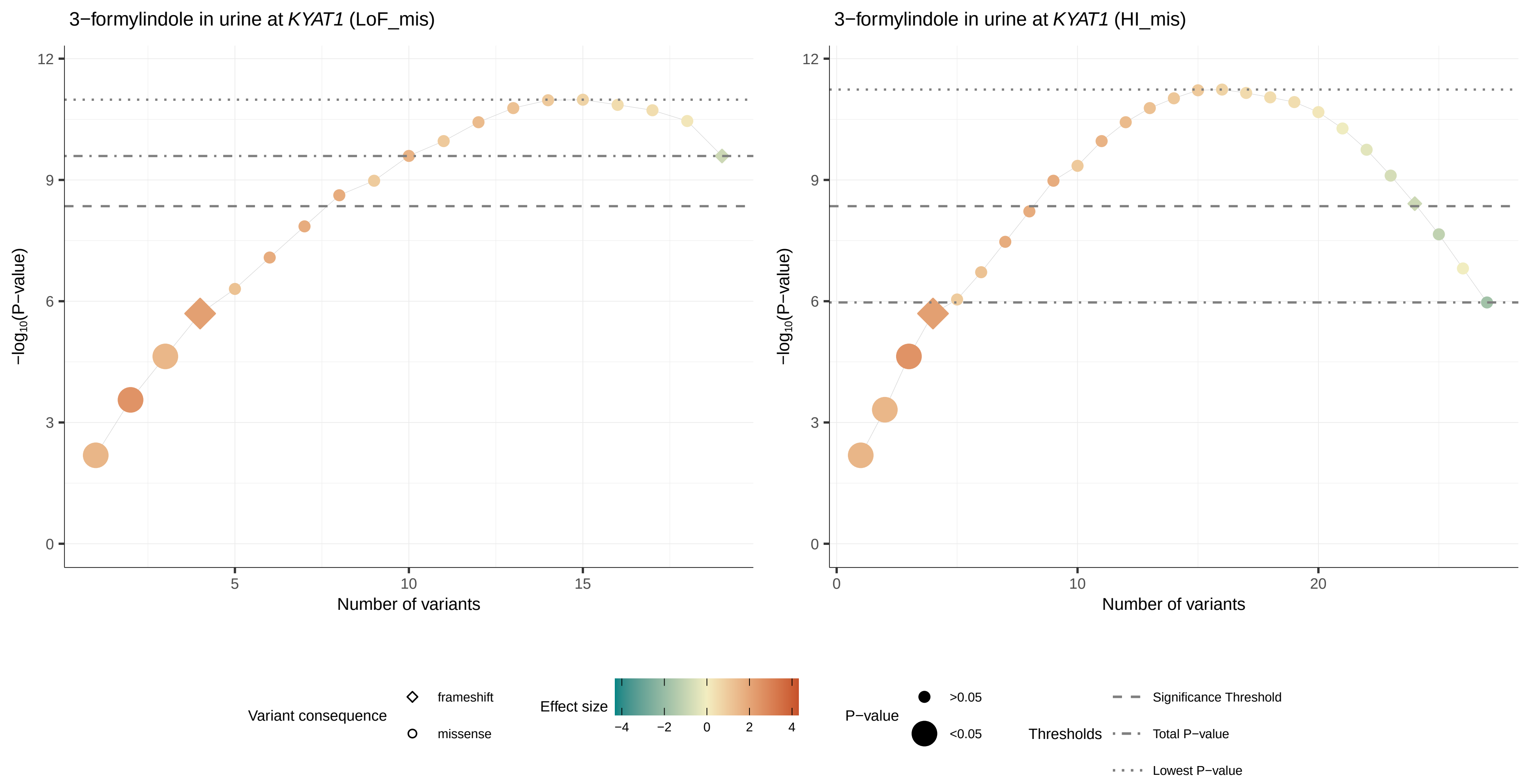
3−formylindole in urine at KYAT1 (LoF_mis)
3−formylindole in urine at KYAT1 (HI_mis)
−log10(P−value)
Number of variants
Variant consequence
frameshift
missense
Effect size
P−value
>0.05
<0.05
Thresholds
Significance Threshold
Total P−value
Lowest P−value

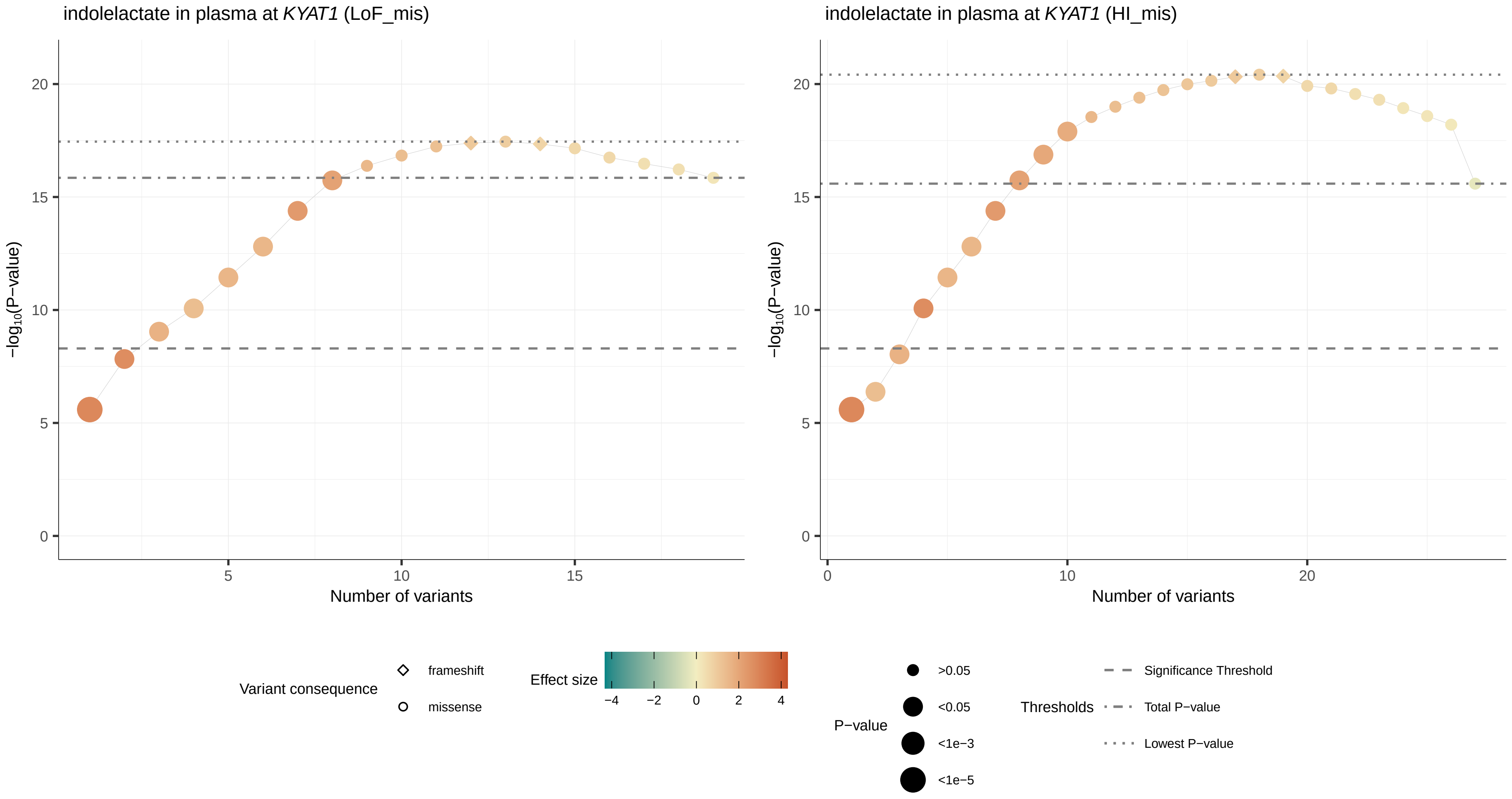

indolelactate in plasma at KYAT1 (LoF_mis)
indolelactate in plasma at KYAT1 (HI_mis)
−log10(P−value)
Number of variants
Variant consequence
frameshift
missense
Effect size
−4
−2
0
2
4
P−value
>0.05
<0.05
<1e−3
<1e−5
Thresholds
Significance Threshold
Total P−value
Lowest P−value

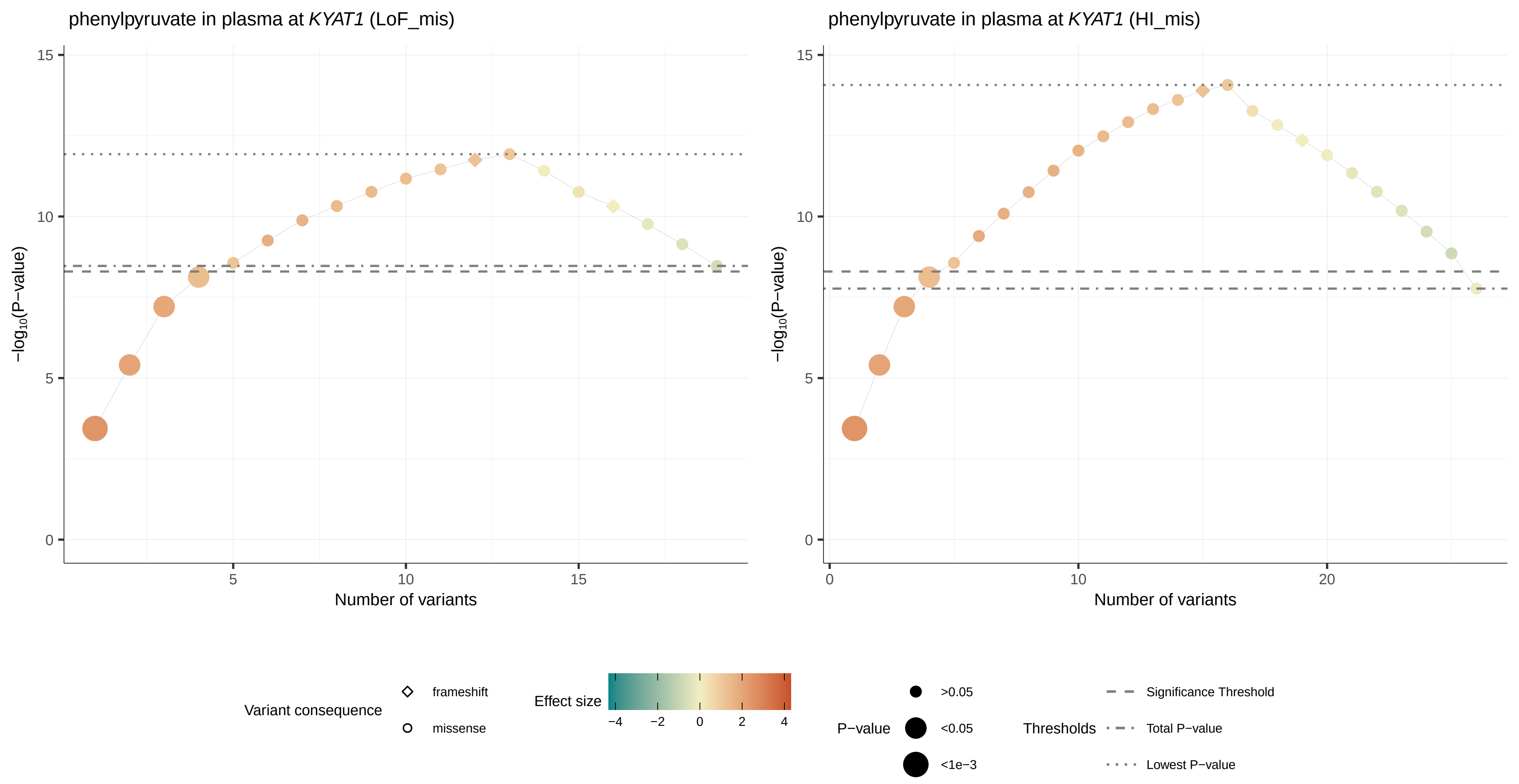

phenylpyruvate in plasma at *KYAT1* (LoF_mis)
phenylpyruvate in plasma at *KYAT1* (HI_mis)
−log10(P−value)
Number of variants
0
5
10
15
20
Variant consequence
frameshift
missense
Effect size
−4
−2
2
4
P−value
>0.05
<0.05
<1e−3
Thresholds
Significance Threshold
Total P−value
Lowest P−value

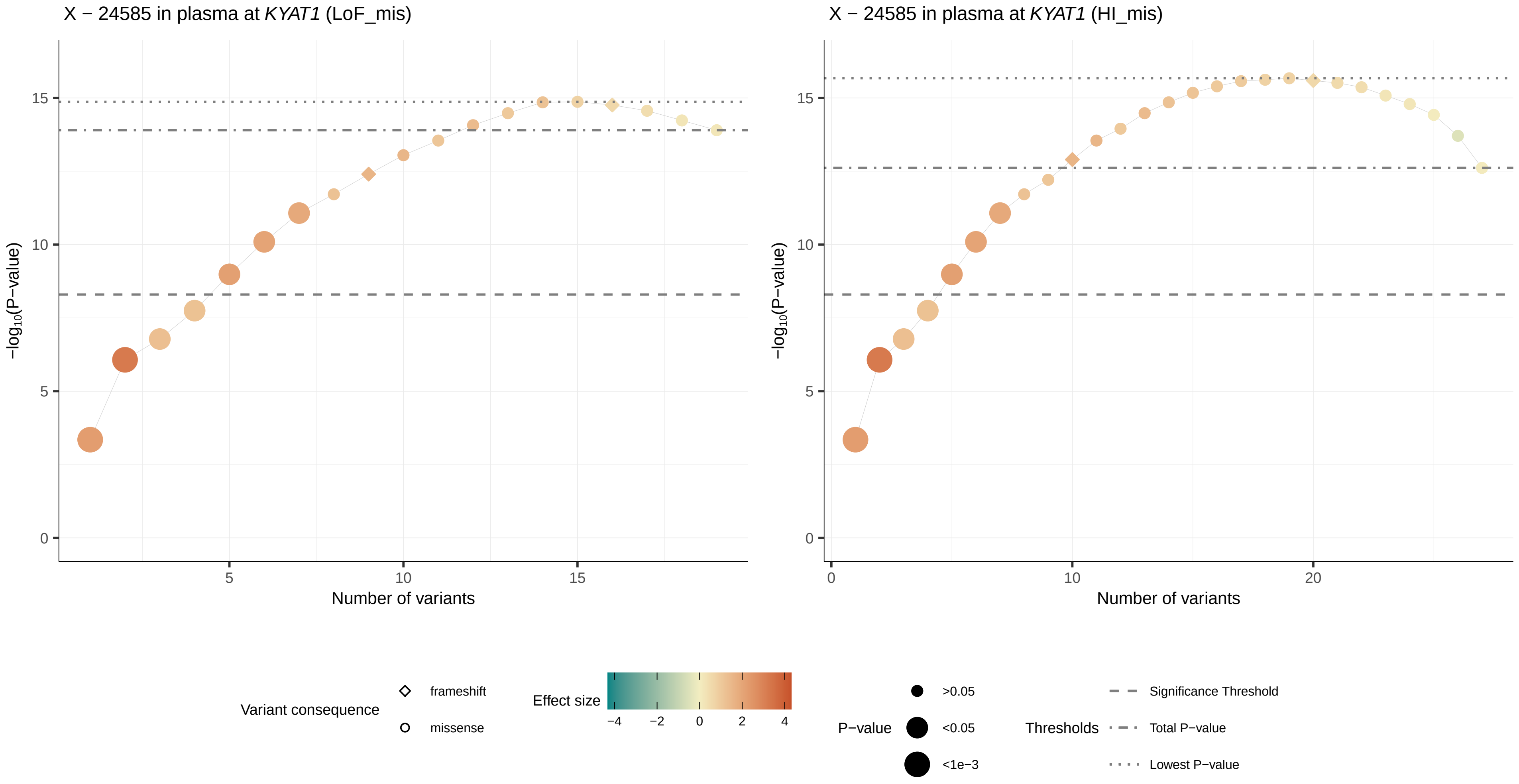

X − 24585 in plasma at KYAT1 (LoF_mis)
X − 24585 in plasma at KYAT1 (HI_mis)
−log10(P−value)
Number of variants
Variant consequence
frameshift
missense
Effect size
P−value
>0.05
<0.05
<1e−3
Thresholds
Significance Threshold
Total P−value
Lowest P−value

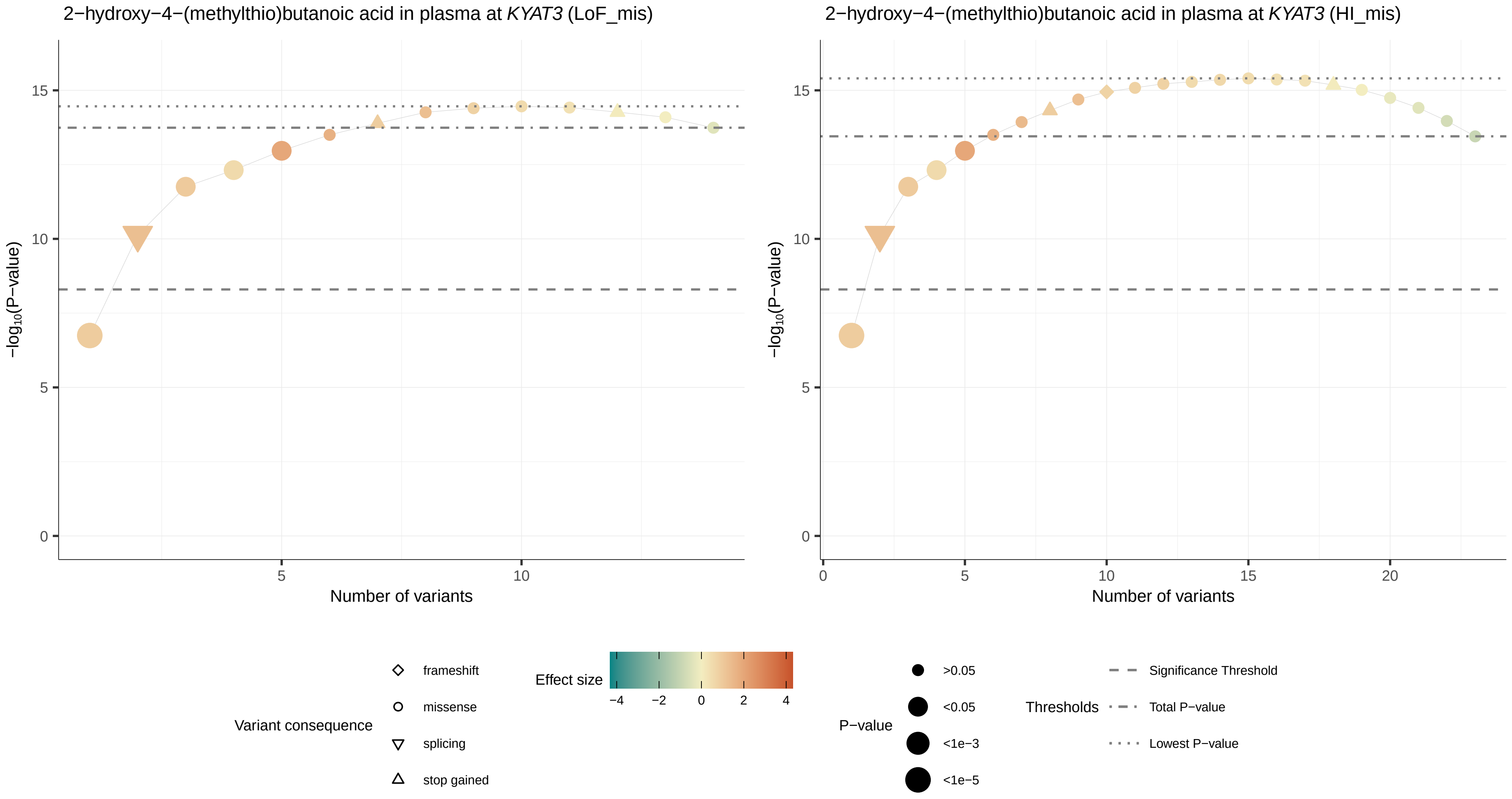

2−hydroxy−4−(methylthio)butanoic acid in plasma at KYAT3 (LoF_mis)
2−hydroxy−4−(methylthio)butanoic acid in plasma at KYAT3 (HI_mis)
−log10(P−value)
Number of variants
Variant consequence
frameshift
missense
splicing
stop gained
Effect size
P−value
>0.05
<0.05
<1e−3
<1e−5
Thresholds
Significance Threshold
Total P−value
Lowest P−value

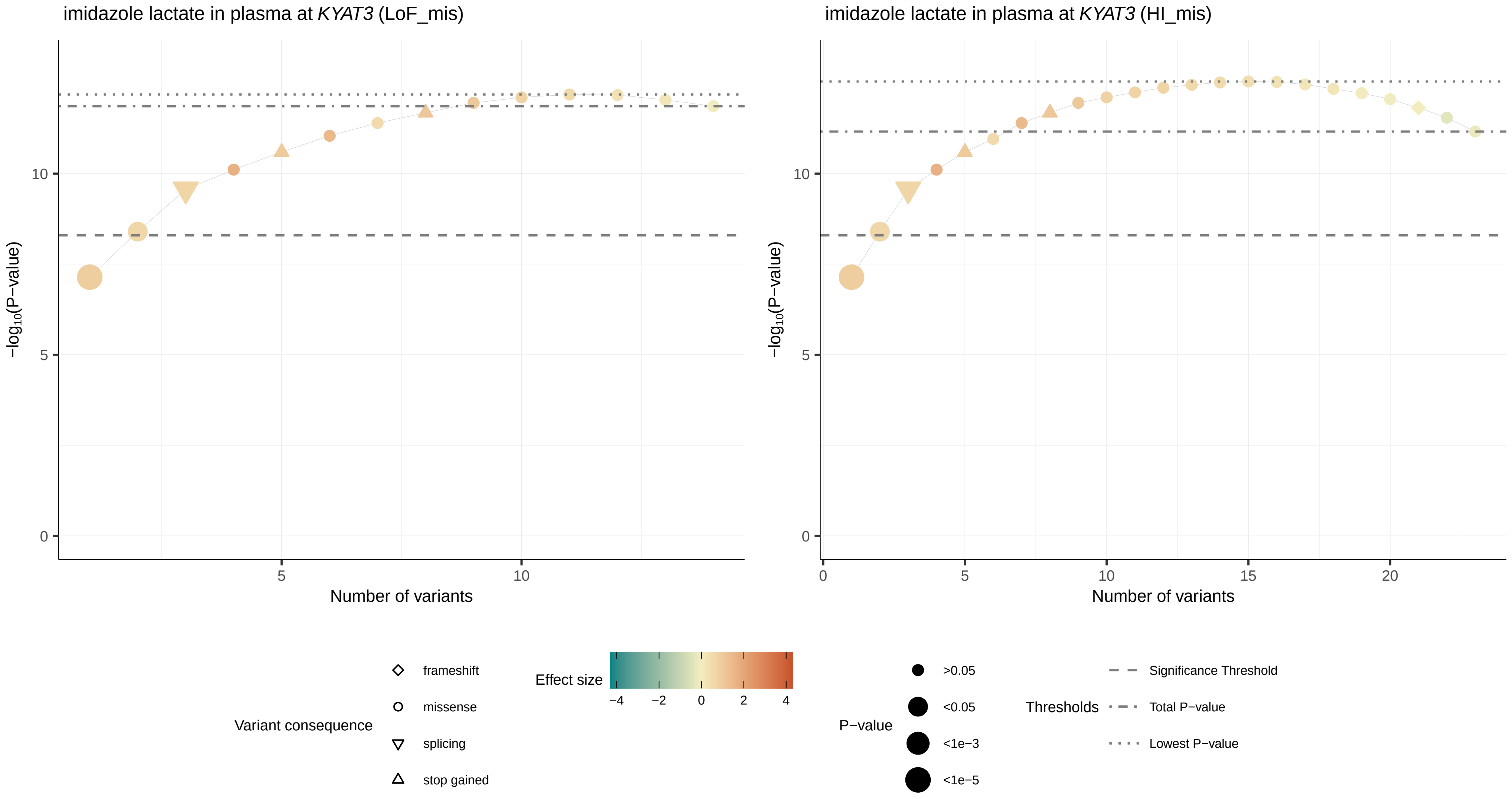
imidazole lactate in plasma at KYAT3 (LoF_mis)
imidazole lactate in plasma at KYAT3 (HI_mis)
−log10(P−value)
Number of variants
Variant consequence
frameshift
missense
splicing
stop gained
Effect size
−4
−2
0
2
4
P−value
>0.05
<0.05
<1e−3
<1e−5
Thresholds
Significance Threshold
Total P−value
Lowest P−value

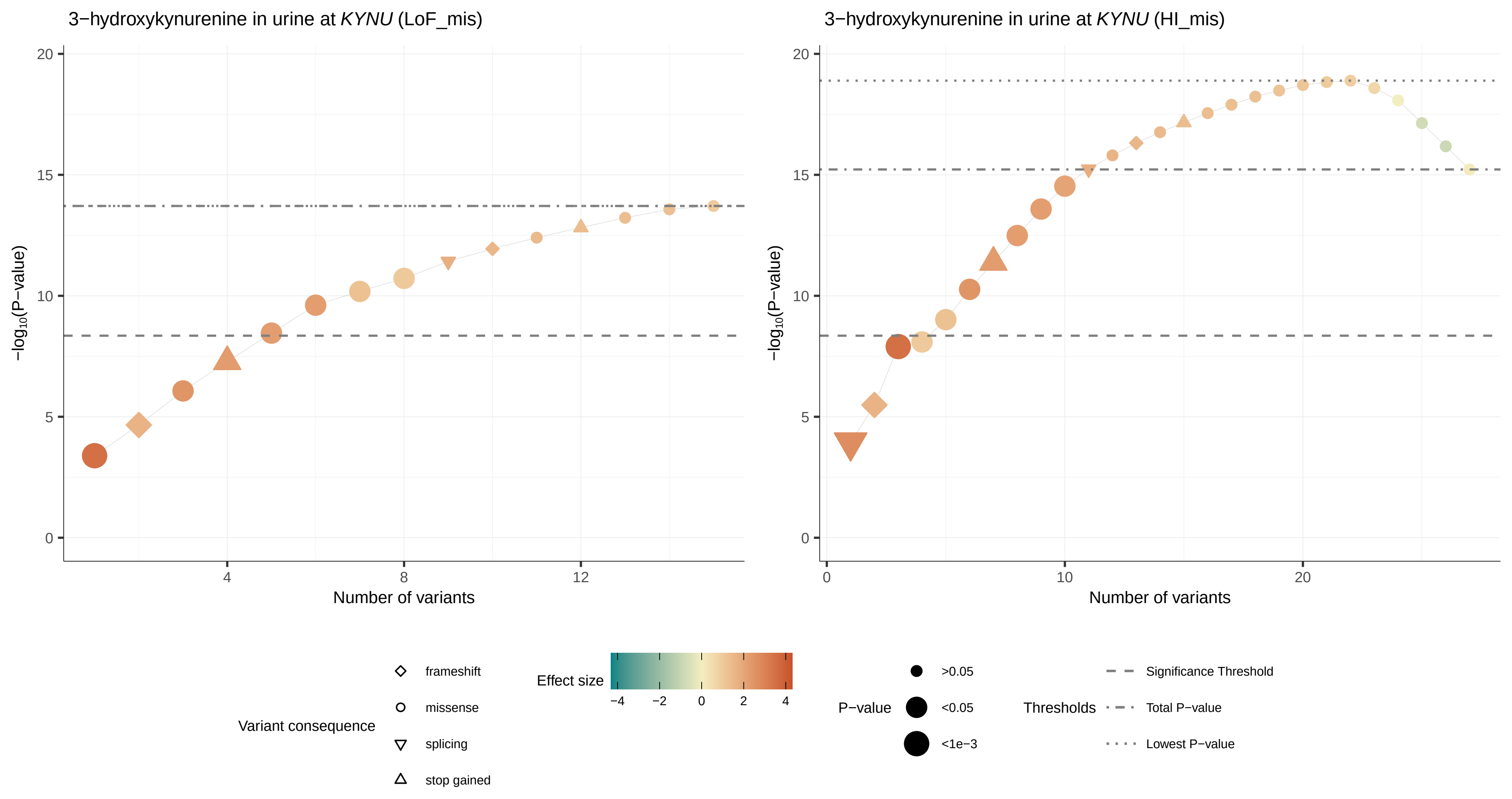
3−hydroxykynurenine in urine at KYNU (LoF_mis)
3−hydroxykynurenine in urine at KYNU (HI_mis)
−log10(P−value)
Number of variants
Variant consequence
frameshift
missense
splicing
stop gained
Effect size
−4
−2
0
2
4
P−value
>0.05
<0.05
<1e−3
Thresholds
Significance Threshold
Total P−value
Lowest P−value

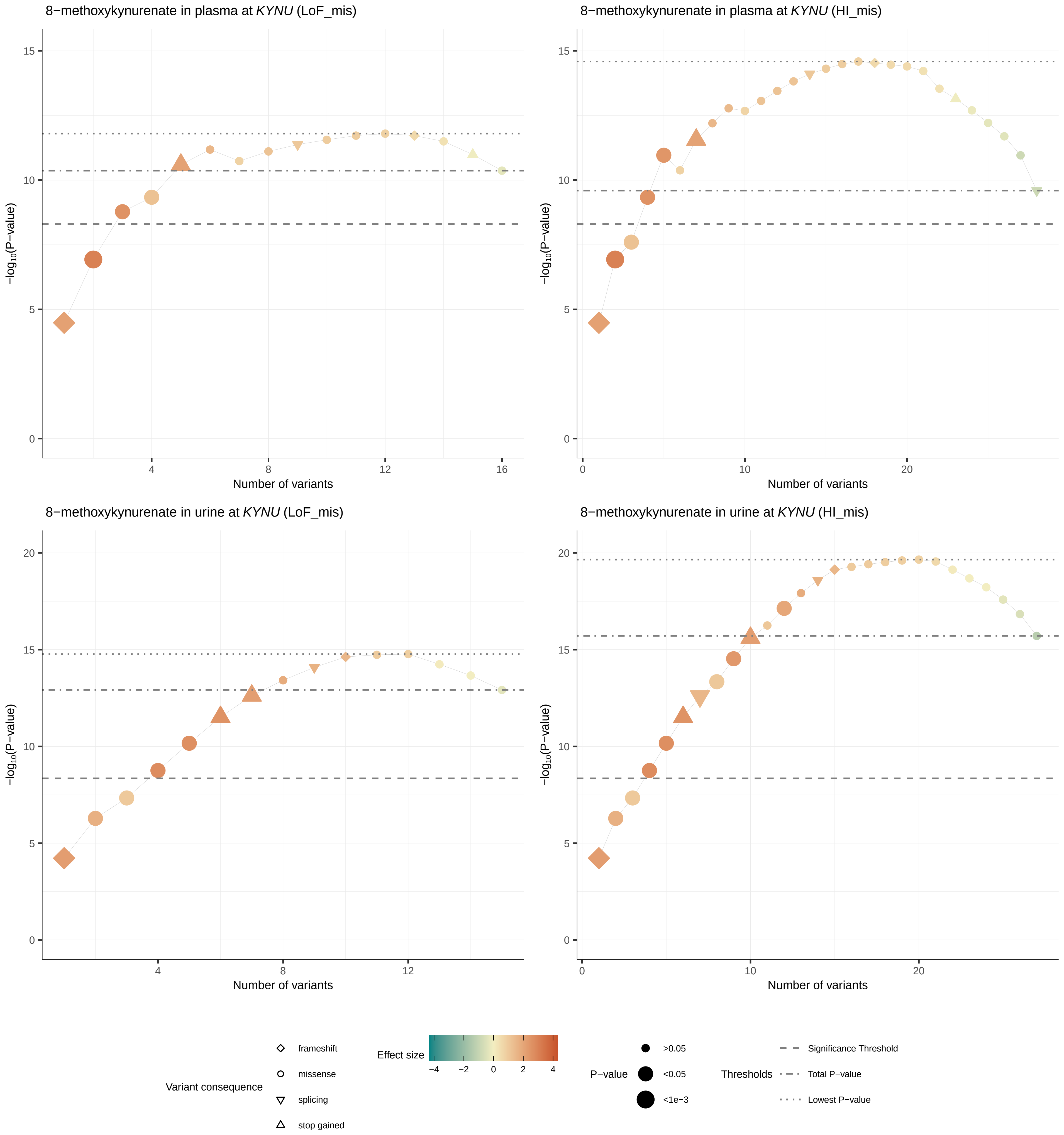

8−methoxykynurenate in plasma at KYNU (LoF_mis)
8−methoxykynurenate in plasma at KYNU (HI_mis)
8−methoxykynurenate in urine at KYNU (LoF_mis)
8−methoxykynurenate in urine at KYNU (HI_mis)
−log10(P−value)
Number of variants
Variant consequence
frameshift
missense
splicing
stop gained
Effect size
−4 −2 0 2 4
P−value
>0.05
<0.05
<1e−3
Thresholds
Significance Threshold
Total P−value
Lowest P−value

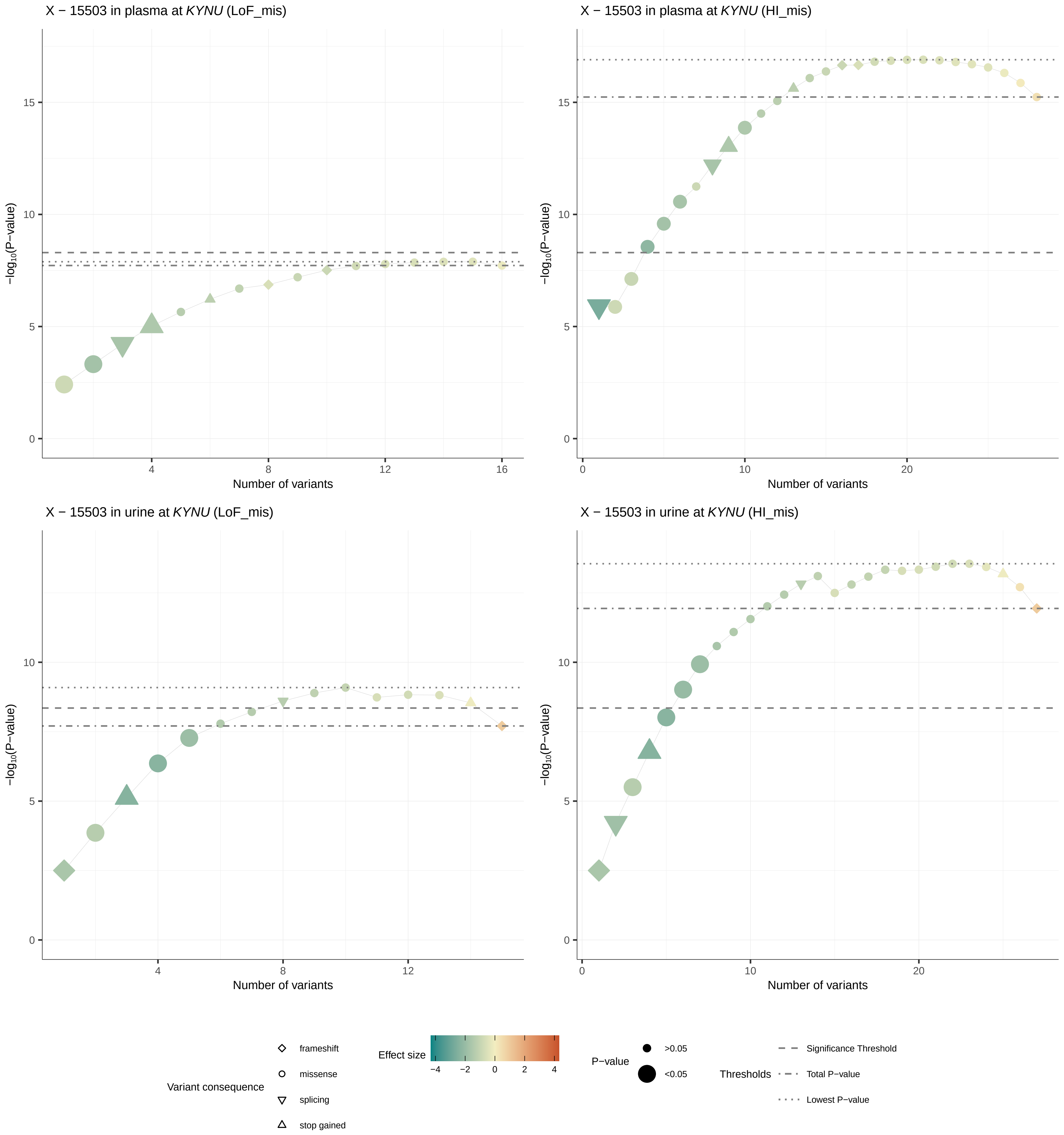

X − 15503 in plasma at KYNU (LoF_mis)
X − 15503 in plasma at KYNU (HI_mis)
X − 15503 in urine at KYNU (LoF_mis)
X − 15503 in urine at KYNU (HI_mis)
−log10(P−value)
Number of variants
Variant consequence
frameshift
missense
splicing
stop gained
Effect size
−4
−2
0
2
4
P−value
>0.05
<0.05
Thresholds
Significance Threshold
Total P−value
Lowest P−value

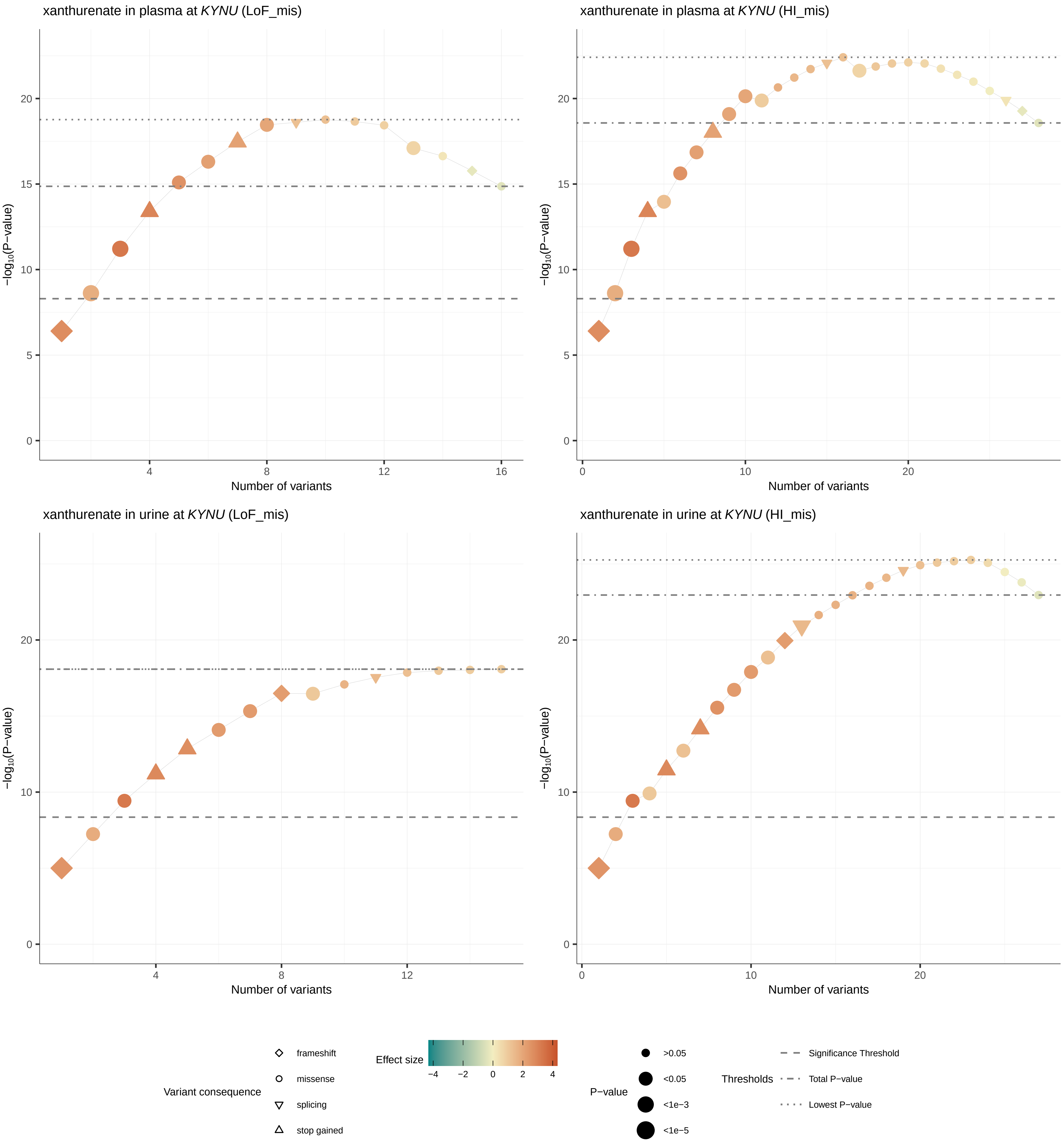
xanthurenate in plasma at KYNU (LoF_mis)
xanthurenate in plasma at KYNU (HI_mis)
xanthurenate in urine at KYNU (LoF_mis)
xanthurenate in urine at KYNU (HI_mis)
−log10(P−value)
Number of variants
Variant consequence
frameshift
missense
splicing
stop gained
Effect size
P−value
>0.05
<0.05
<1e−3
<1e−5
Thresholds
Significance Threshold
Total P−value
Lowest P−value

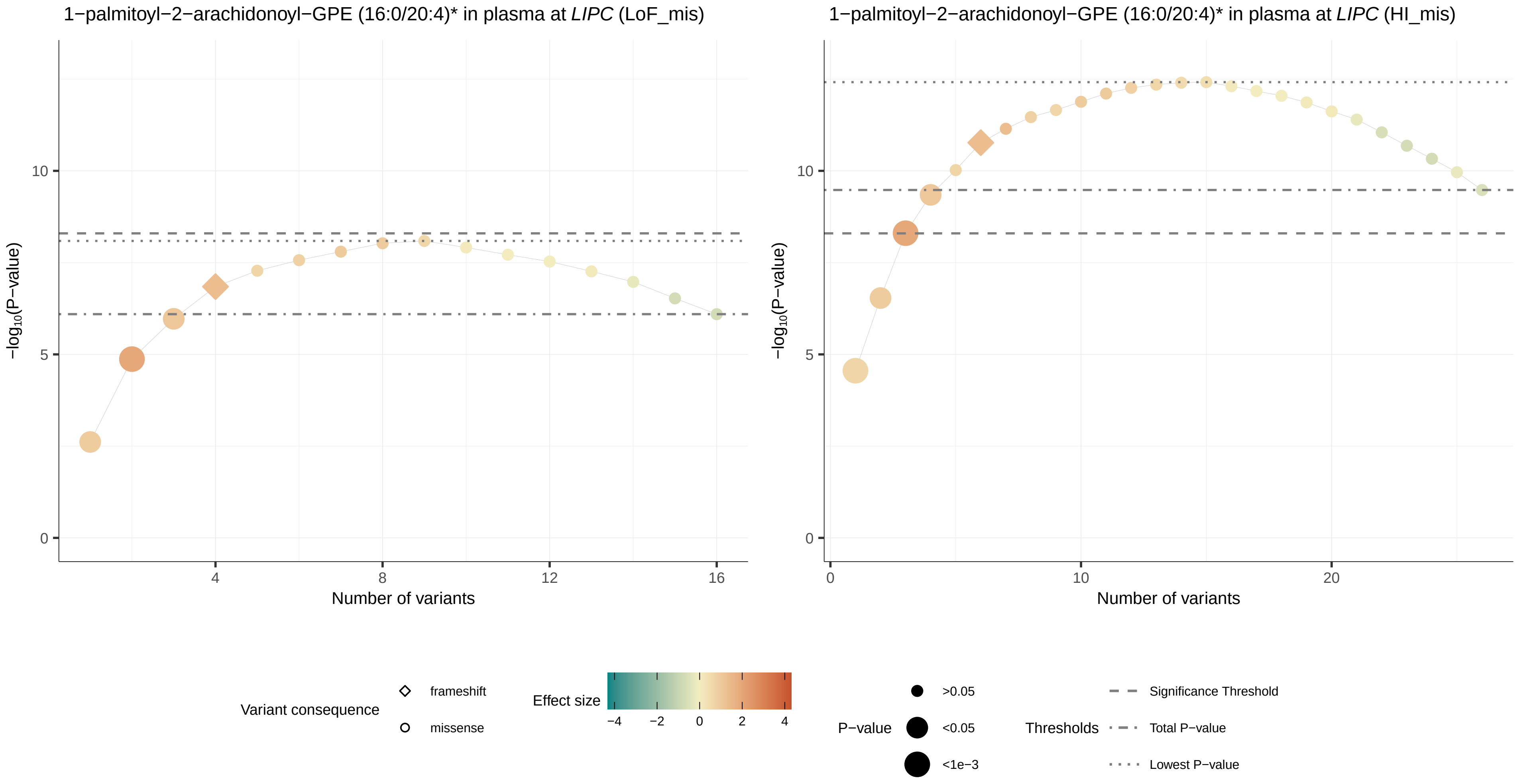
1−palmitoyl−2−arachidonoyl−GPE (16:0/20:4)* in plasma at LIPC (LoF_mis)
1−palmitoyl−2−arachidonoyl−GPE (16:0/20:4)* in plasma at LIPC (HI_mis)
−log10(P−value)
Number of variants
Variant consequence
frameshift
missense
Effect size
−4
−2
0
2
4
P−value
>0.05
<0.05
<1e−3
Thresholds
Significance Threshold
Total P−value
Lowest P−value

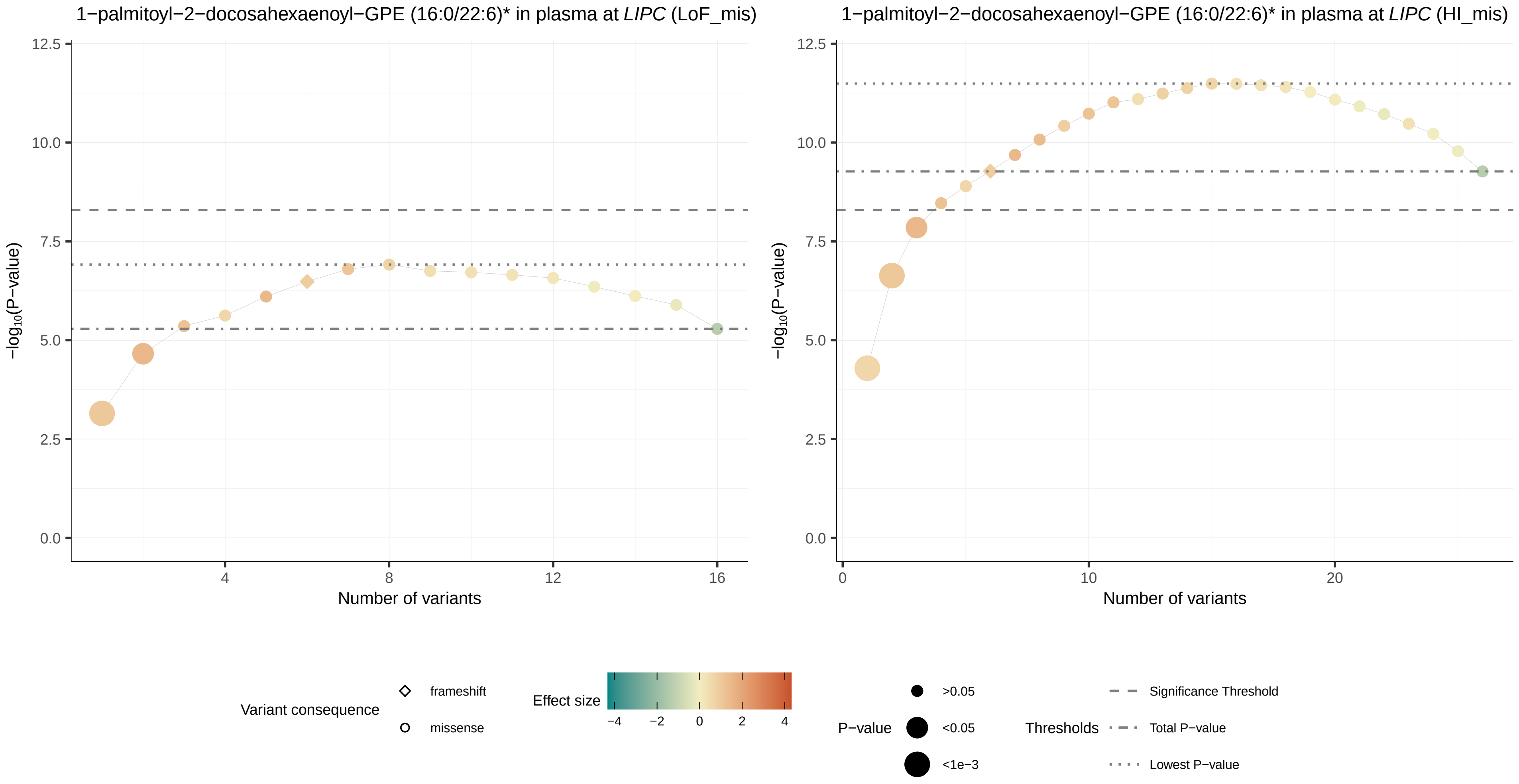

1−palmitoyl−2−docosahexaenoyl−GPE (16:0/22:6)* in plasma at LIPC (LoF_mis)
1−palmitoyl−2−docosahexaenoyl−GPE (16:0/22:6)* in plasma at LIPC (HI_mis)
−log10(P−value)
12.5
10.0
7.5
5.0
2.5
0.0
4
8
12
16
0
10
20
Number of variants
Variant consequence
frameshift
missense
Effect size
−4
−2
0
2
4
P−value
>0.05
<0.05
<1e−3
Thresholds
Significance Threshold
Total P−value
Lowest P−value

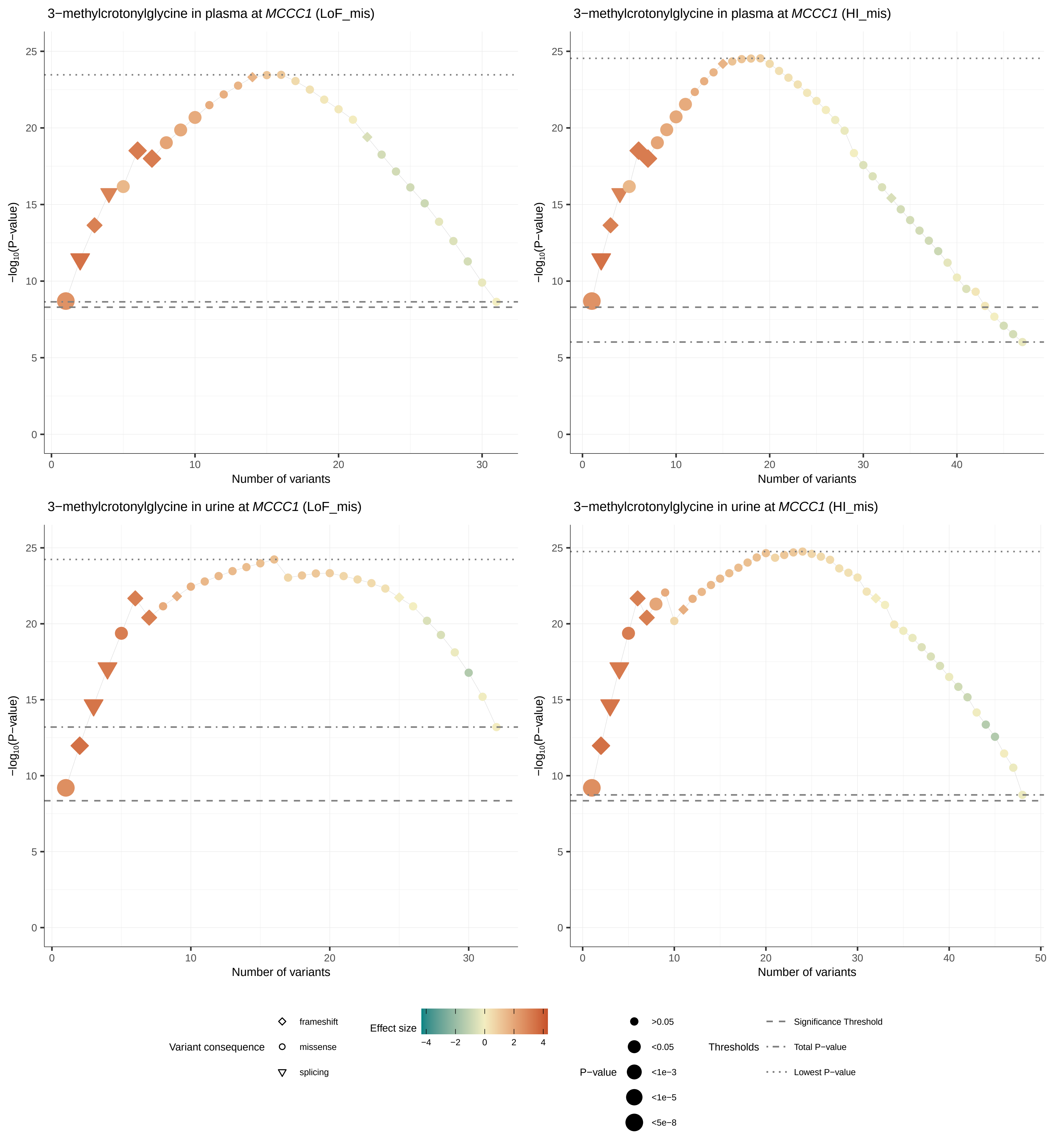

3−methylcrotonylglycine in plasma at MCCC1 (LoF_mis)
3−methylcrotonylglycine in plasma at MCCC1 (HI_mis)
3−methylcrotonylglycine in urine at MCCC1 (LoF_mis)
3−methylcrotonylglycine in urine at MCCC1 (HI_mis)
−log10(P−value)
Number of variants
Variant consequence
frameshift
missense
splicing
Effect size
P−value
>0.05
<0.05
<1e−3
<1e−5
<5e−8
Thresholds
Significance Threshold
Total P−value
Lowest P−value

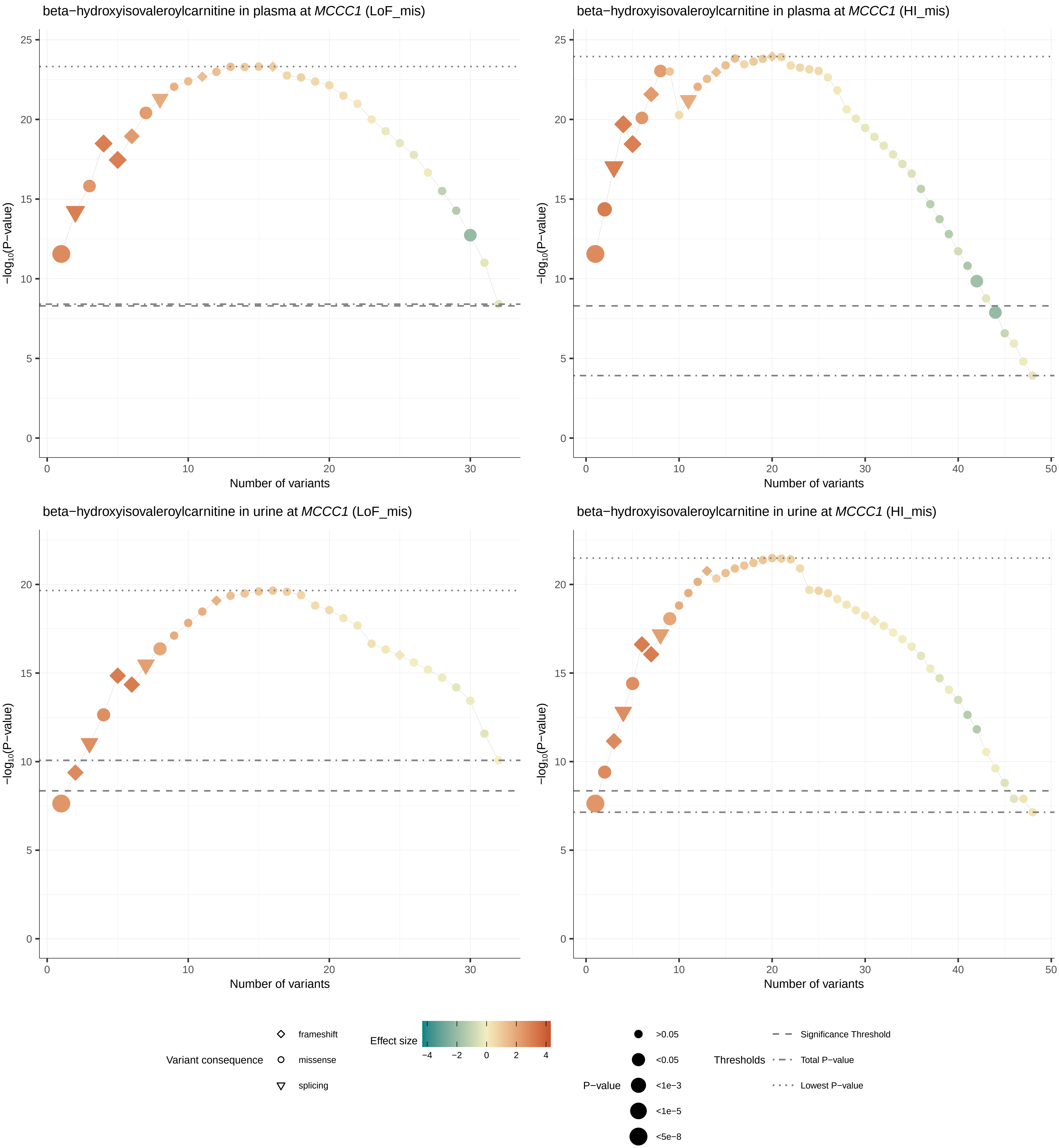

beta−hydroxyisovaleroylcarnitine in plasma at MCCC1 (LoF_mis)
beta−hydroxyisovaleroylcarnitine in plasma at MCCC1 (HI_mis)
beta−hydroxyisovaleroylcarnitine in urine at MCCC1 (LoF_mis)
beta−hydroxyisovaleroylcarnitine in urine at MCCC1 (HI_mis)
−log10(P−value)
Number of variants
Variant consequence
frameshift
missense
splicing
Effect size
−4
−2
0
2
4
P−value
>0.05
<0.05
<1e−3
<1e−5
<5e−8
Thresholds
Significance Threshold
Total P−value
Lowest P−value

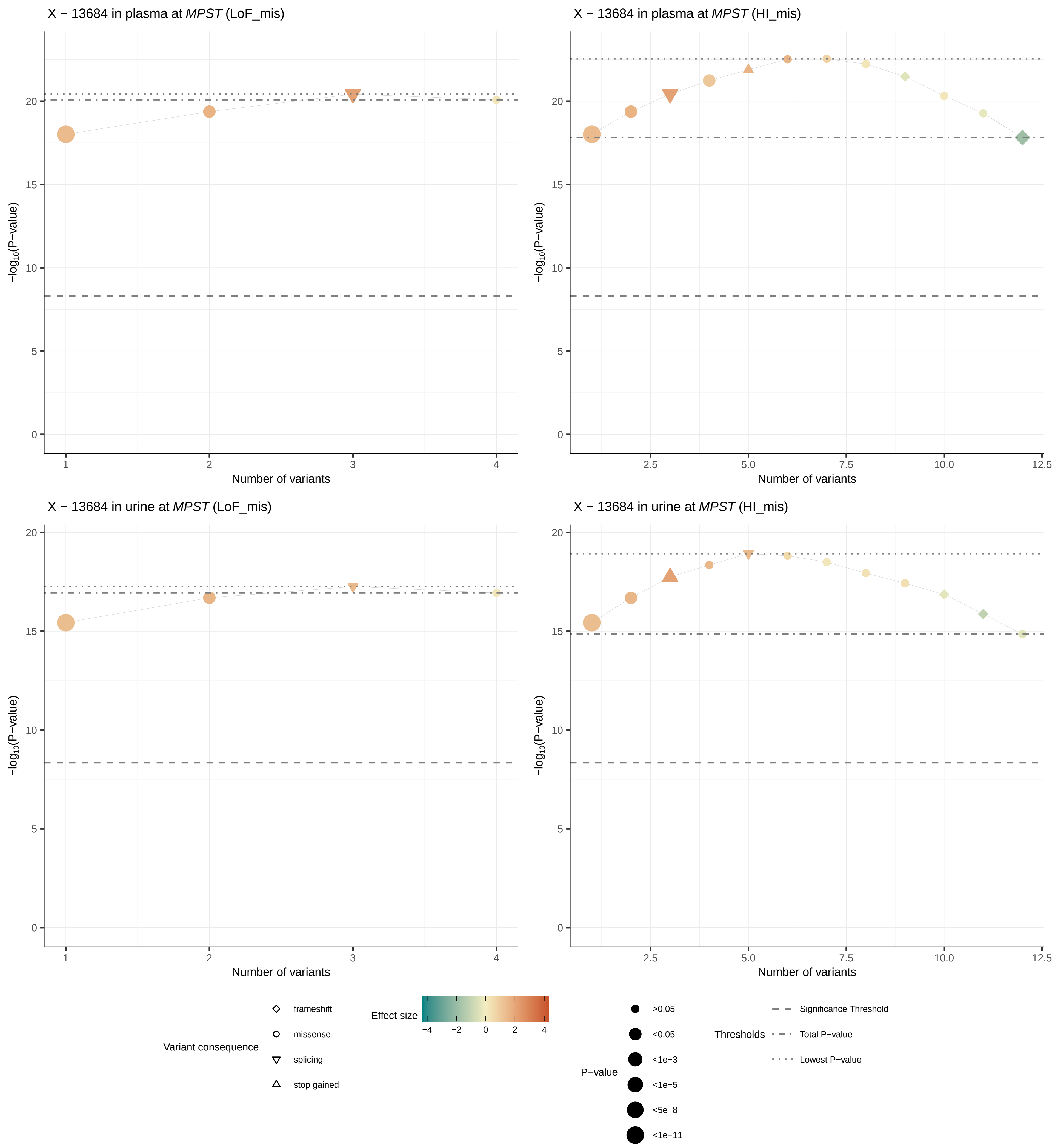
X − 13684 in plasma at MPST (LoF_mis)
X − 13684 in plasma at MPST (HI_mis)
X − 13684 in urine at MPST (LoF_mis)
X − 13684 in urine at MPST (HI_mis)
−log10(P−value)
Number of variants
Variant consequence
frameshift
missense
splicing
stop gained
Effect size
P−value
>0.05
<0.05
<1e−3
<1e−5
<5e−8
<1e−11
Thresholds
Significance Threshold
Total P−value
Lowest P−value

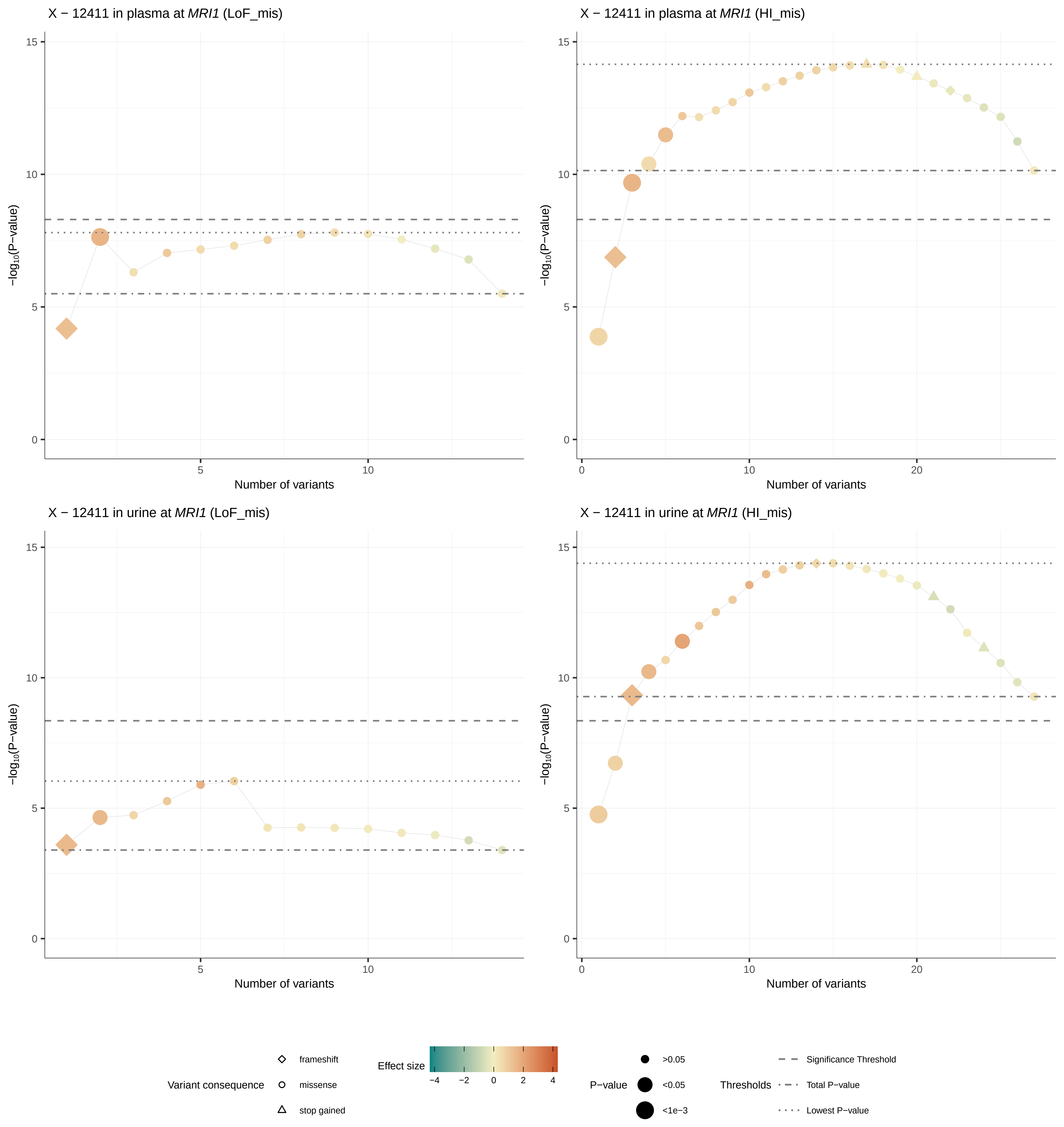

X − 12411 in plasma at MRI1 (LoF_mis)
X − 12411 in plasma at MRI1 (HI_mis)
X − 12411 in urine at MRI1 (LoF_mis)
X − 12411 in urine at MRI1 (HI_mis)
−log10(P−value)
Number of variants
Variant consequence
frameshift
missense
stop gained
Effect size
P−value
>0.05
<0.05
<1e−3
Thresholds
Significance Threshold
Total P−value
Lowest P−value

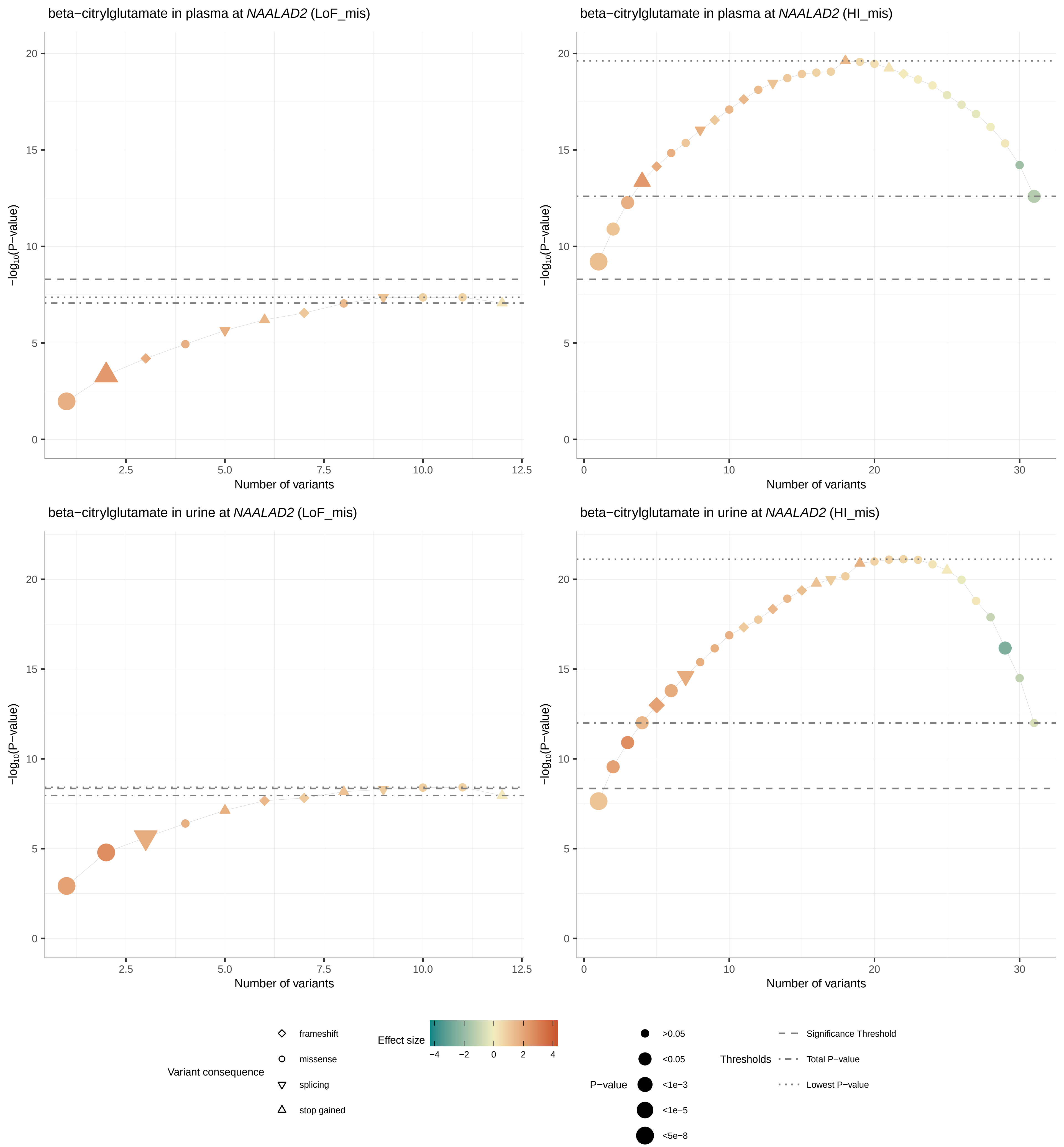
beta−citrylglutamate in plasma at *NAALAD2* (LoF_mis)
beta−citrylglutamate in plasma at *NAALAD2* (HI_mis)
beta−citrylglutamate in urine at *NAALAD2* (LoF_mis)
beta−citrylglutamate in urine at *NAALAD2* (HI_mis)
$-\log_{10}$(P−value)
Number of variants
Variant consequence
frameshift
missense
splicing
stop gained
Effect size
−4
−2
0
2
4
P−value
>0.05
<0.05
<1e−3
<1e−5
<5e−8
Thresholds
Significance Threshold
Total P−value
Lowest P−value

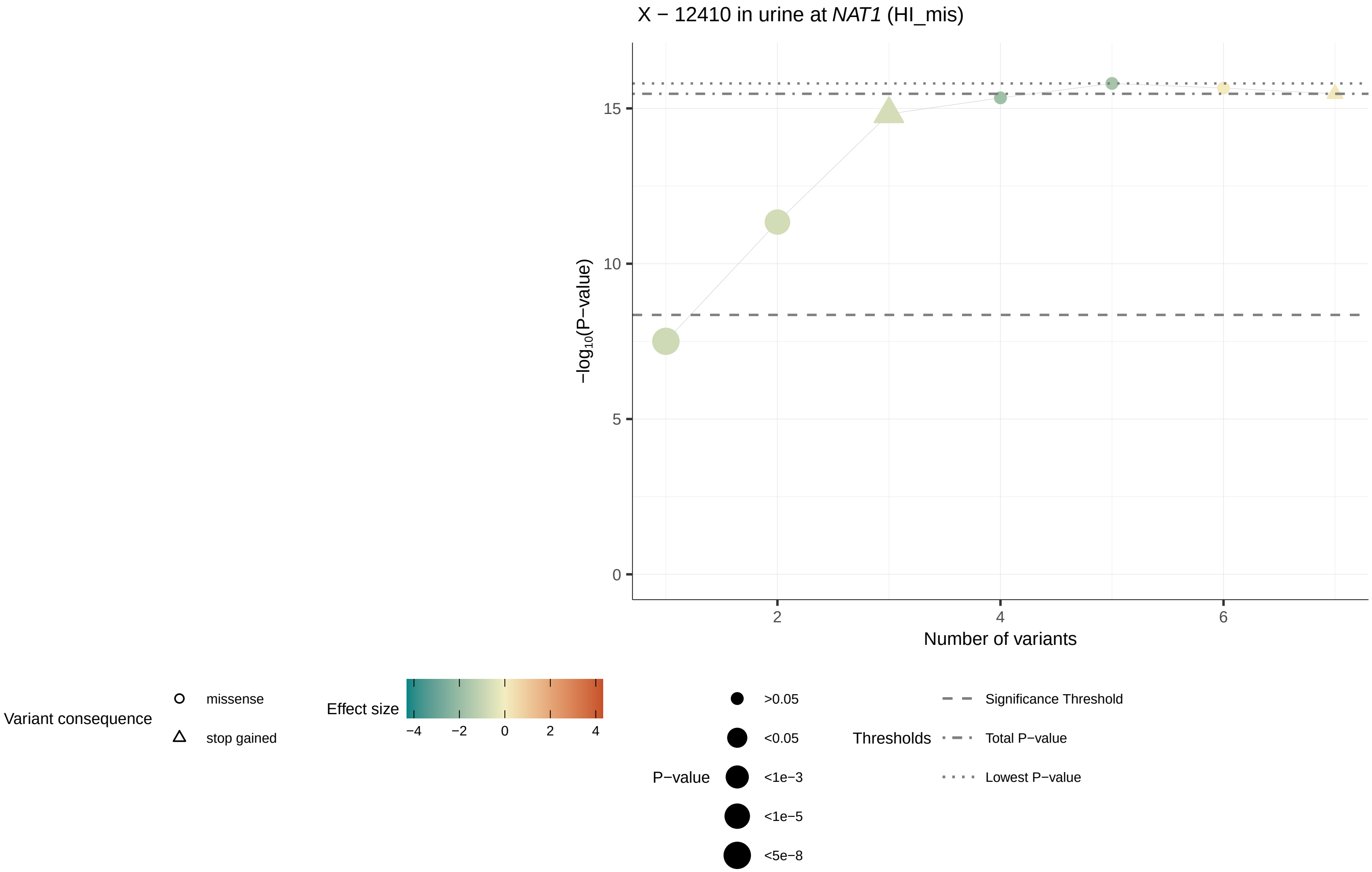
X − 12410 in urine at *NAT1* (HI_mis)
−log10(P−value)
0
5
10
15
2
4
6
Number of variants
Variant consequence
missense
stop gained
Effect size
−4
−2
0
2
4
P−value
>0.05
<0.05
<1e−3
<1e−5
<5e−8
Thresholds
Significance Threshold
Total P−value
Lowest P−value

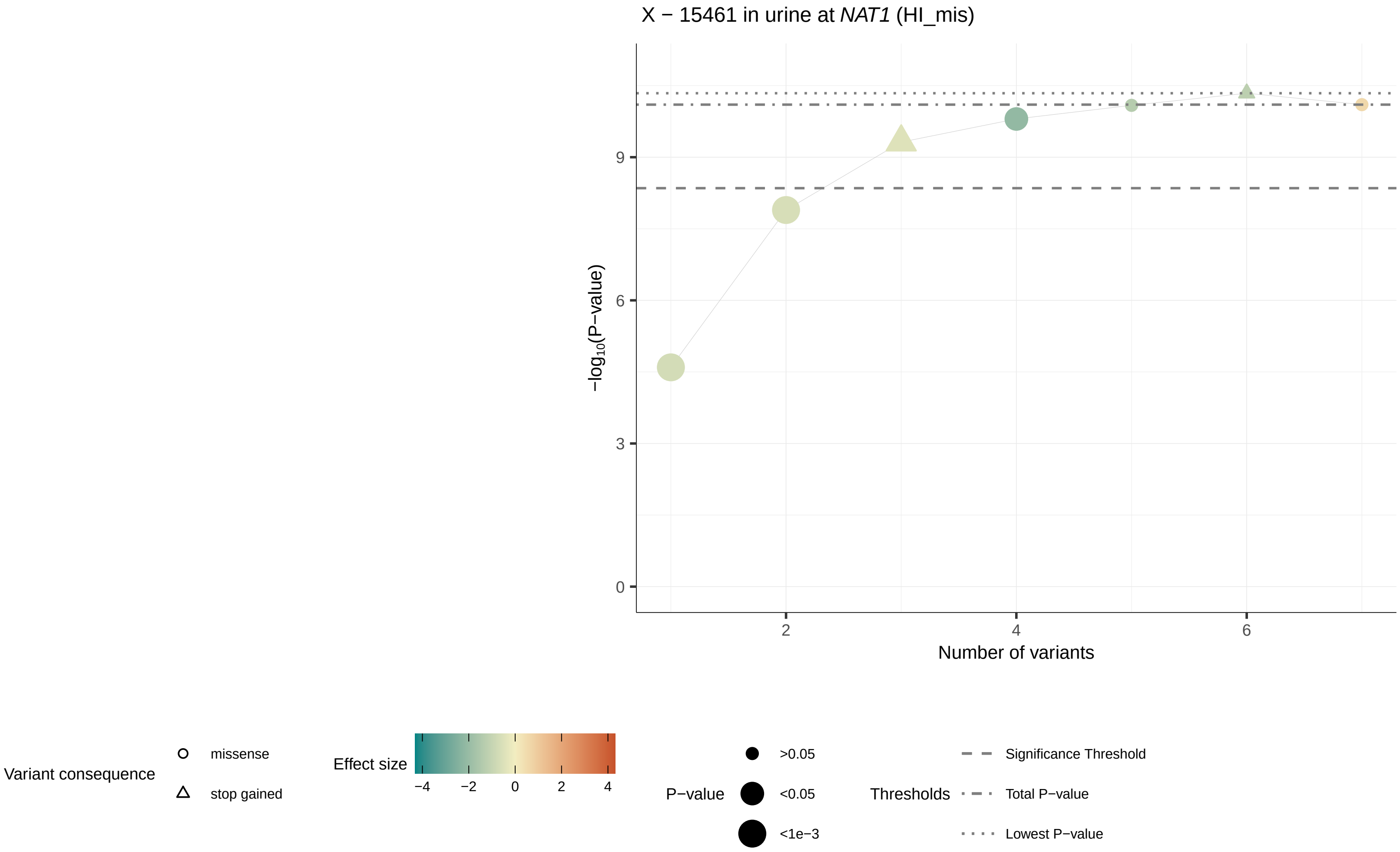

X − 15461 in urine at NAT1 (HI_mis)
−log10(P−value)
0
3
6
9
2
4
6
Number of variants
Variant consequence
missense
stop gained
Effect size
−4
−2
0
2
4
P−value
>0.05
<0.05
<1e−3
Thresholds
Significance Threshold
Total P−value
Lowest P−value

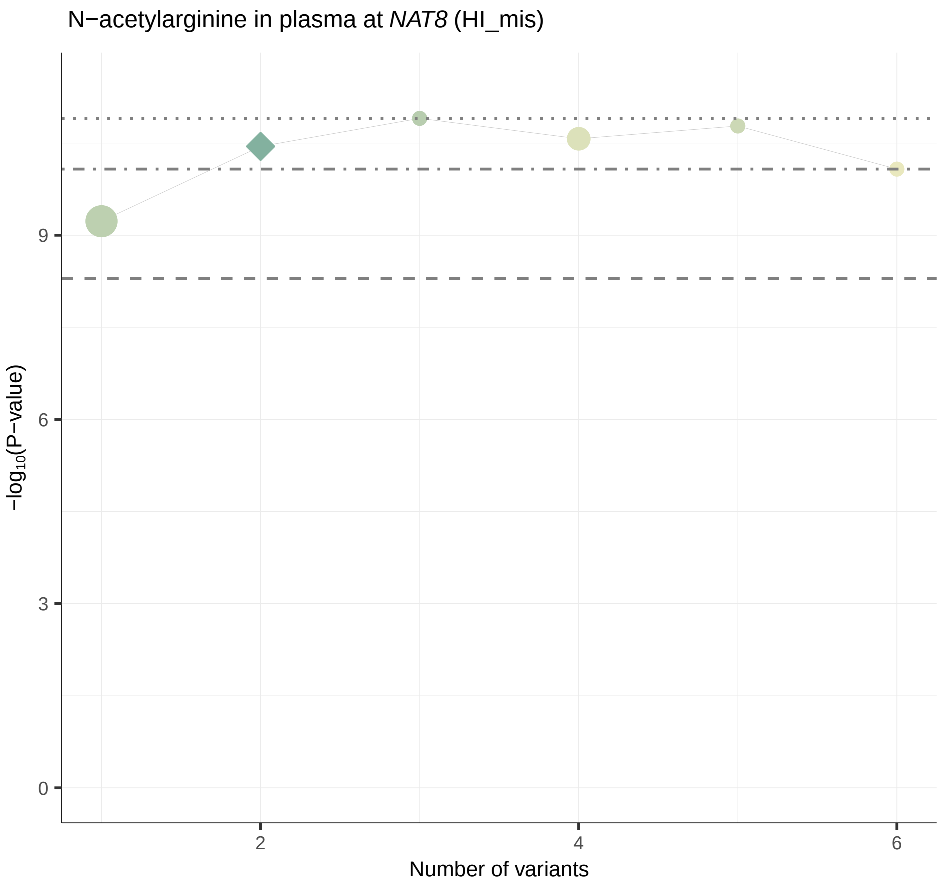
N−acetylarginine in plasma at NAT8 (HI_mis)
−log10(P−value)
0
3
6
9
2
4
6
Number of variants

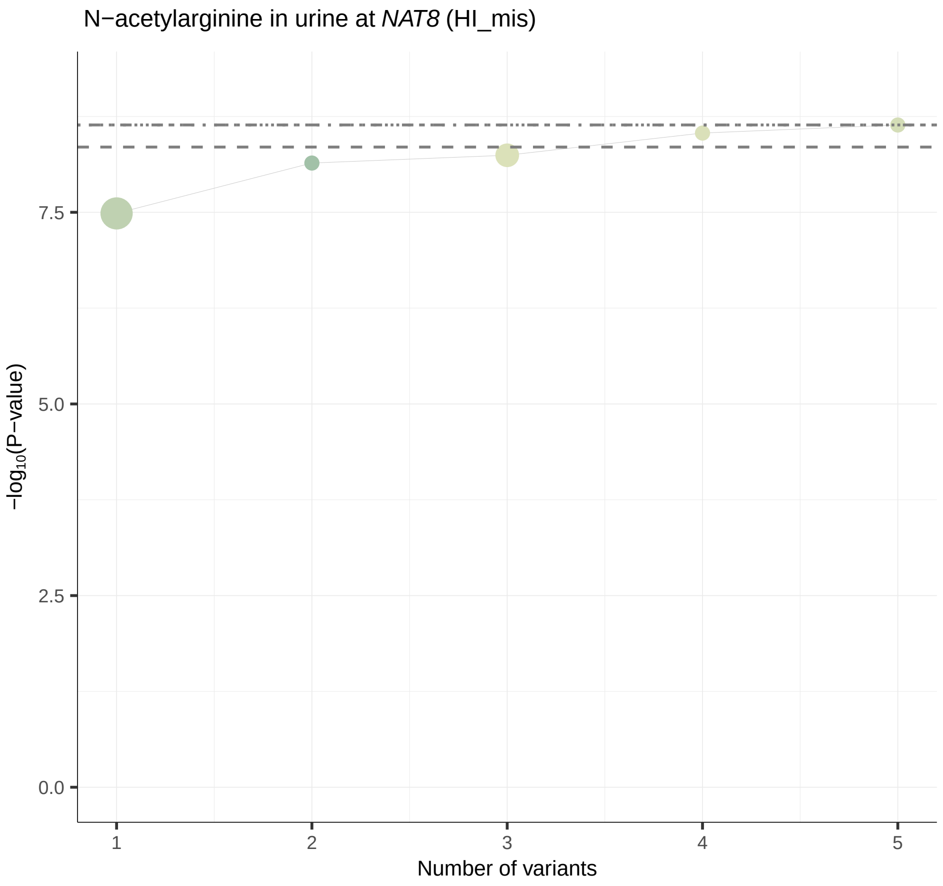
N−acetylarginine in urine at NAT8 (HI_mis)
−log10(P−value)
0.0
2.5
5.0
7.5
1
2
3
4
5
Number of variants

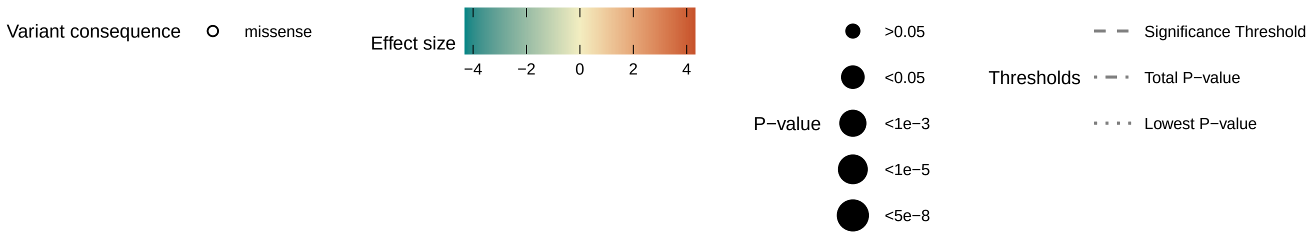
Variant consequence
missense
Effect size
−4
−2
0
2
4
P−value
>0.05
<0.05
<1e−3
<1e−5
<5e−8
Thresholds
Significance Threshold
Total P−value
Lowest P−value

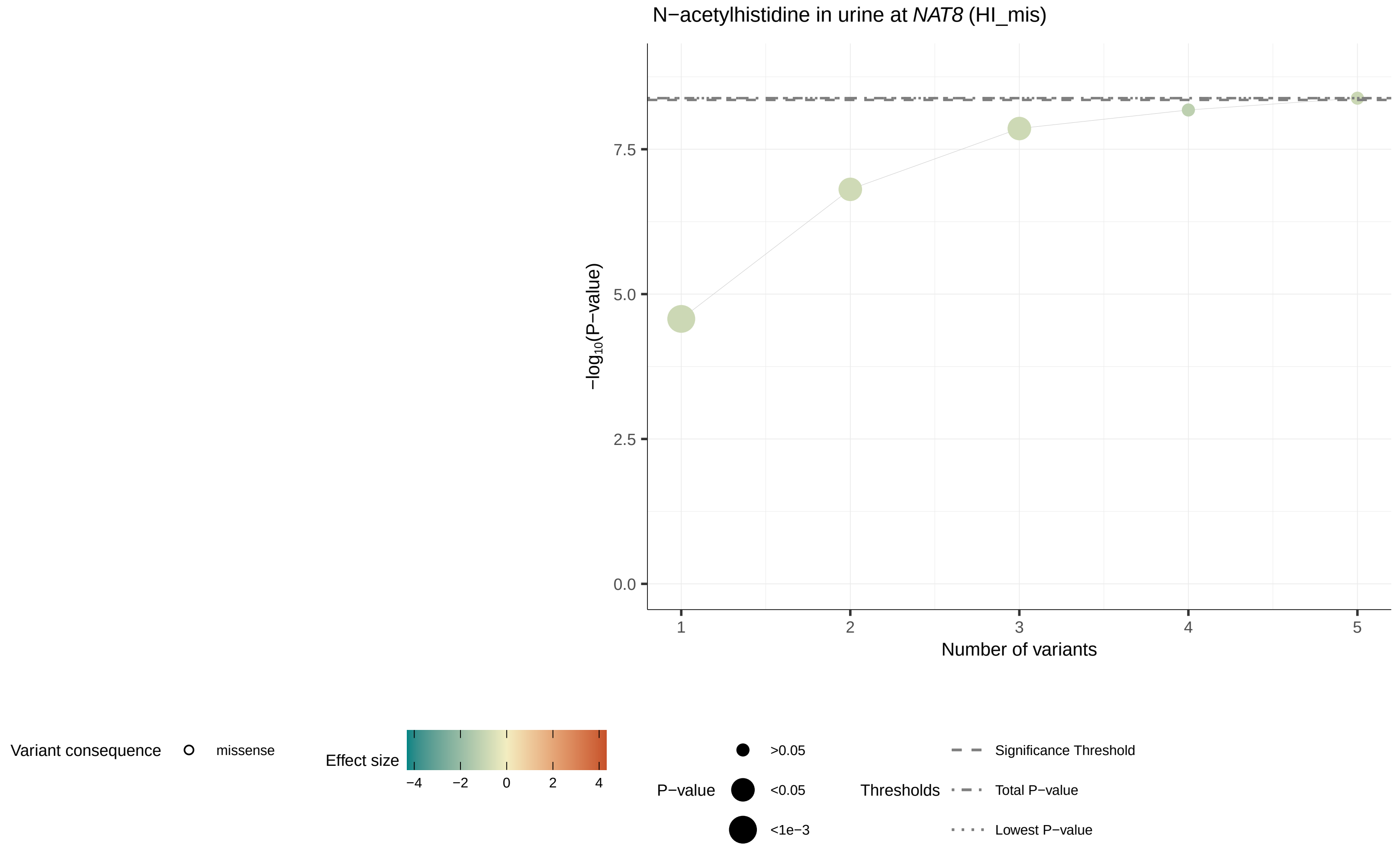

N−acetylhistidine in urine at NAT8 (HI_mis)
−log10(P−value)
7.5
5.0
2.5
0.0
1
2
3
4
5
Number of variants
Variant consequence
missense
Effect size
−4
−2
0
2
4
P−value
>0.05
<0.05
<1e−3
Thresholds
Significance Threshold
Total P−value
Lowest P−value

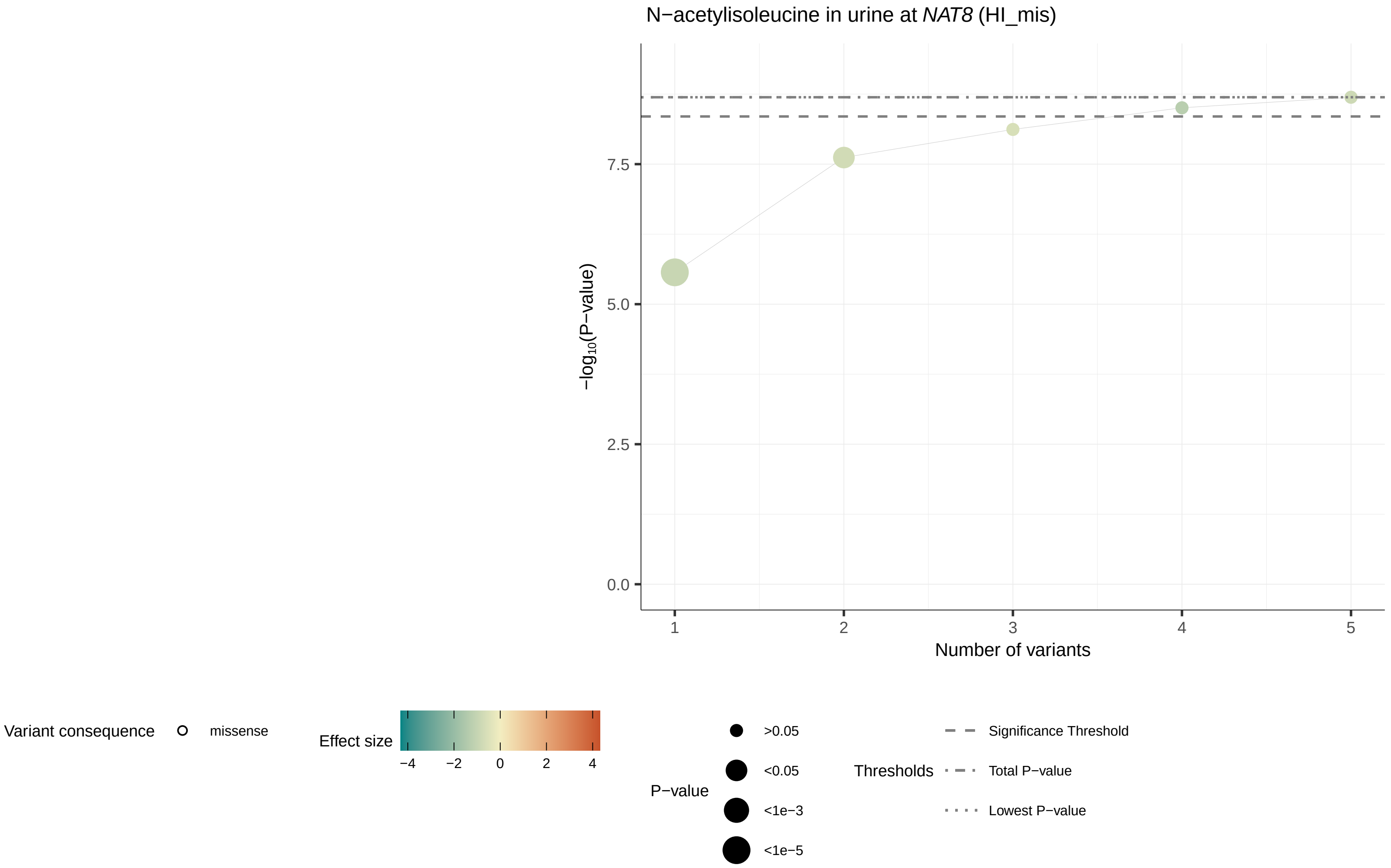
N−acetylisoleucine in urine at NAT8 (HI_mis)
−log10(P−value)
7.5
5.0
2.5
0.0
1
2
3
4
5
Number of variants
Variant consequence
missense
Effect size
−4
−2
0
2
4
P−value
>0.05
<0.05
<1e−3
<1e−5
Thresholds
Significance Threshold
Total P−value
Lowest P−value

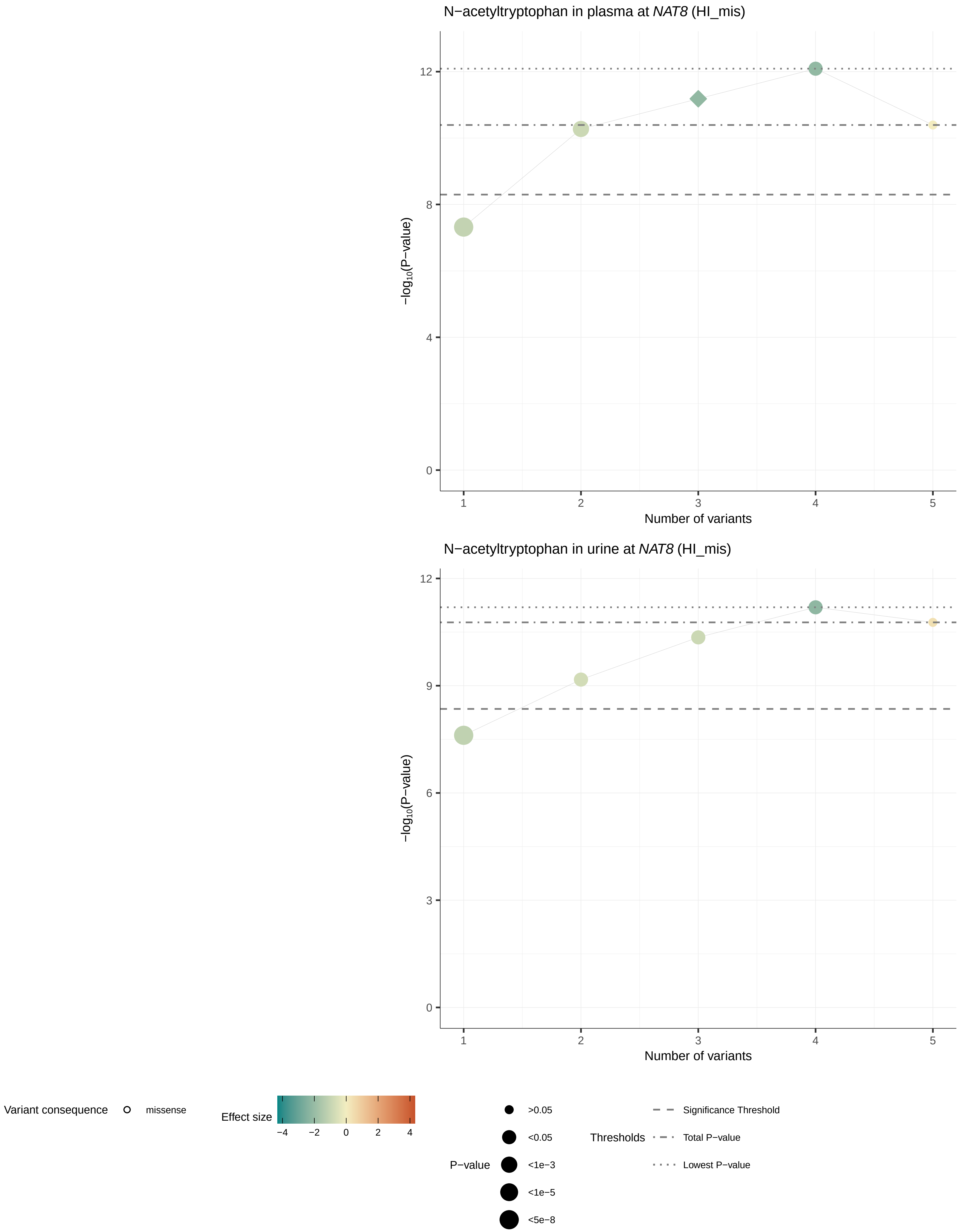
N−acetyltryptophan in plasma at NAT8 (HI_mis)
−log10(P−value)
Number of variants
N−acetyltryptophan in urine at NAT8 (HI_mis)
−log10(P−value)
Number of variants
Variant consequence
missense
Effect size
P−value
>0.05
<0.05
<1e−3
<1e−5
<5e−8
Thresholds
Significance Threshold
Total P−value
Lowest P−value

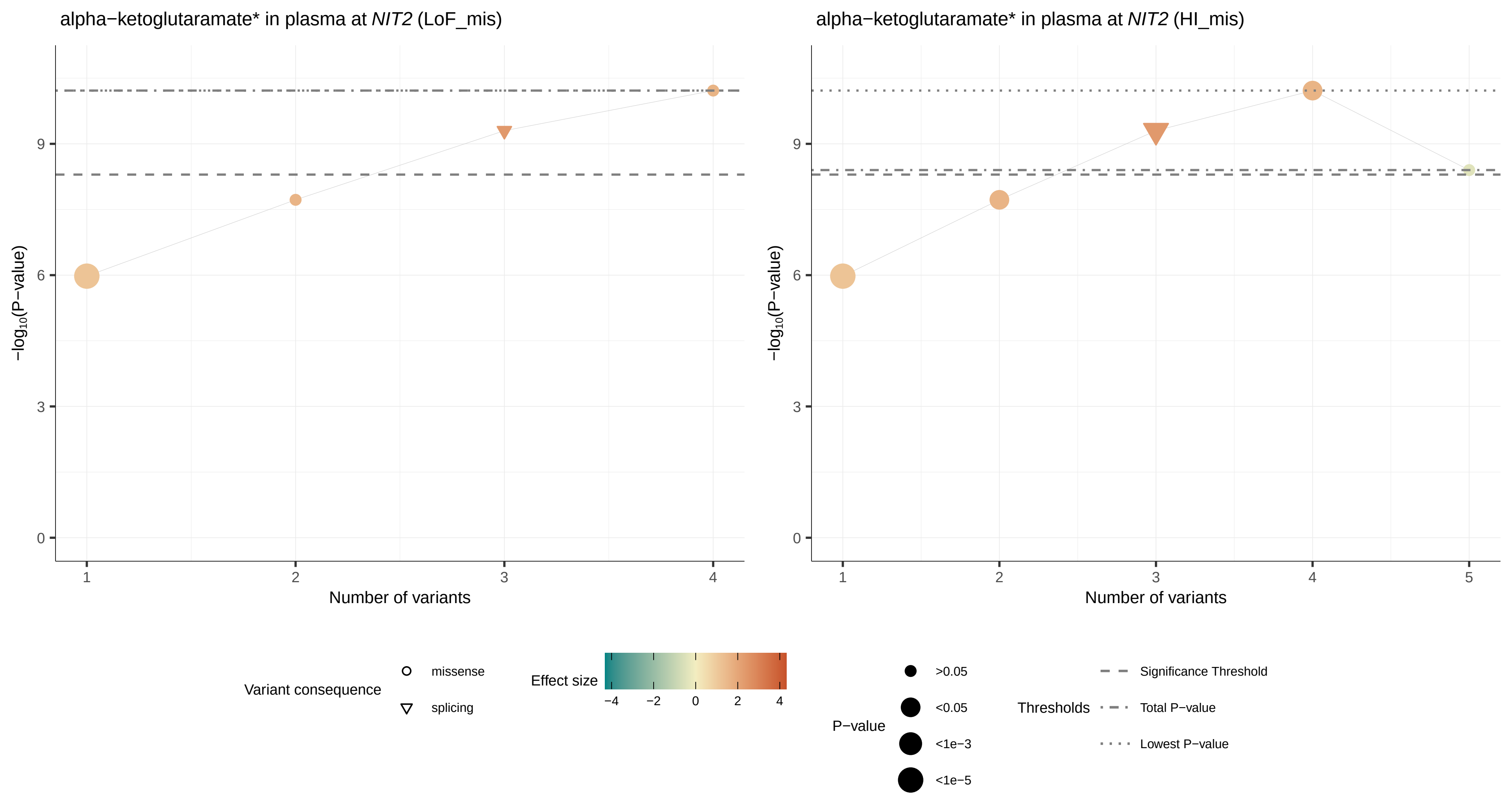

alpha−ketoglutaramate* in plasma at NIT2 (LoF_mis)
alpha−ketoglutaramate* in plasma at NIT2 (HI_mis)
−log10(P−value)
0
3
6
9
1
2
3
4
5
Number of variants
Variant consequence
missense
splicing
Effect size
−4
−2
0
2
4
P−value
>0.05
<0.05
<1e−3
<1e−5
Thresholds
Significance Threshold
Total P−value
Lowest P−value

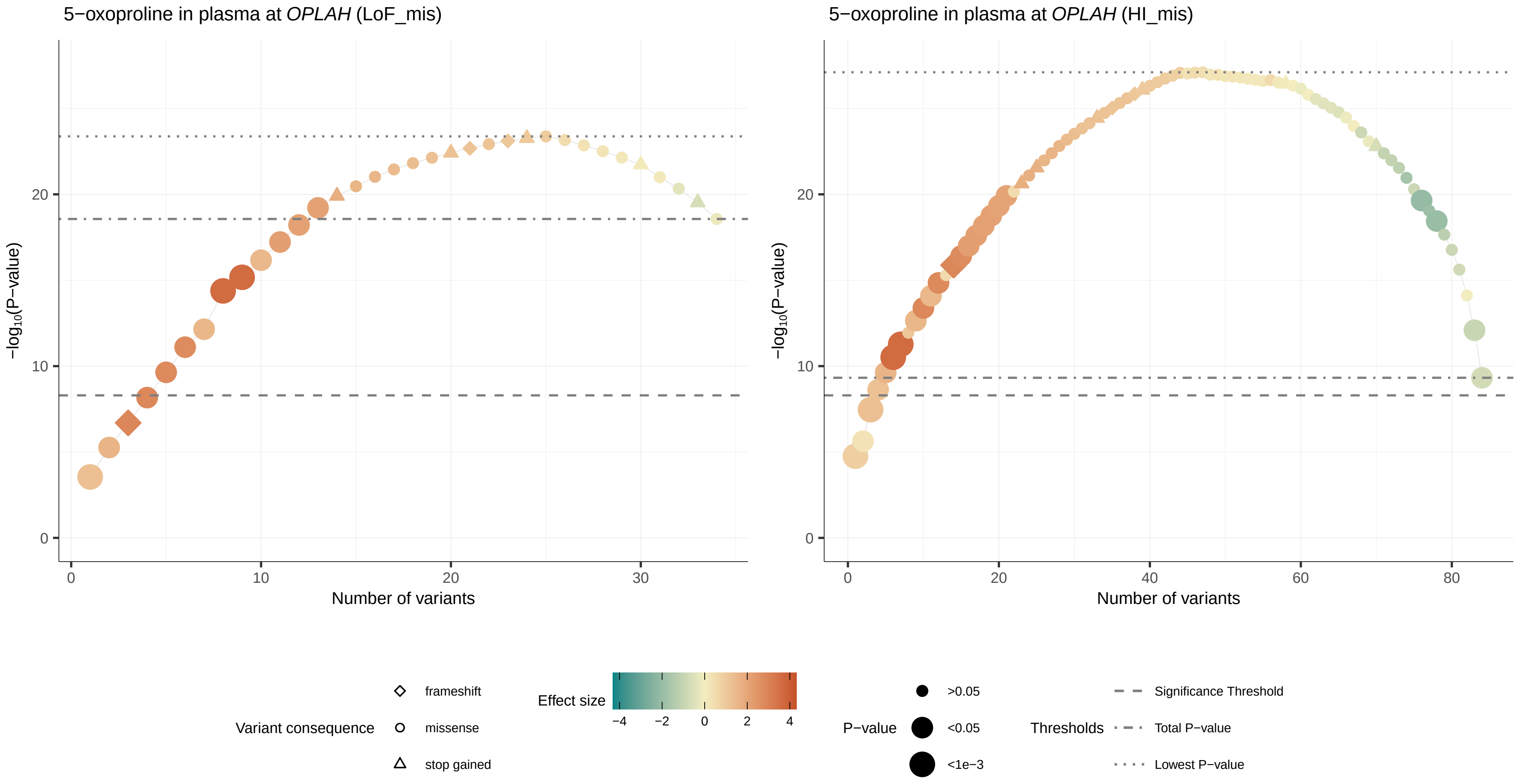

5−oxoproline in plasma at OPLAH (LoF_mis)
5−oxoproline in plasma at OPLAH (HI_mis)
−log10(P−value)
Number of variants
0
10
20
30
40
60
80
Variant consequence
frameshift
missense
stop gained
Effect size
−4
−2
0
2
4
P−value
>0.05
<0.05
<1e−3
Thresholds
Significance Threshold
Total P−value
Lowest P−value

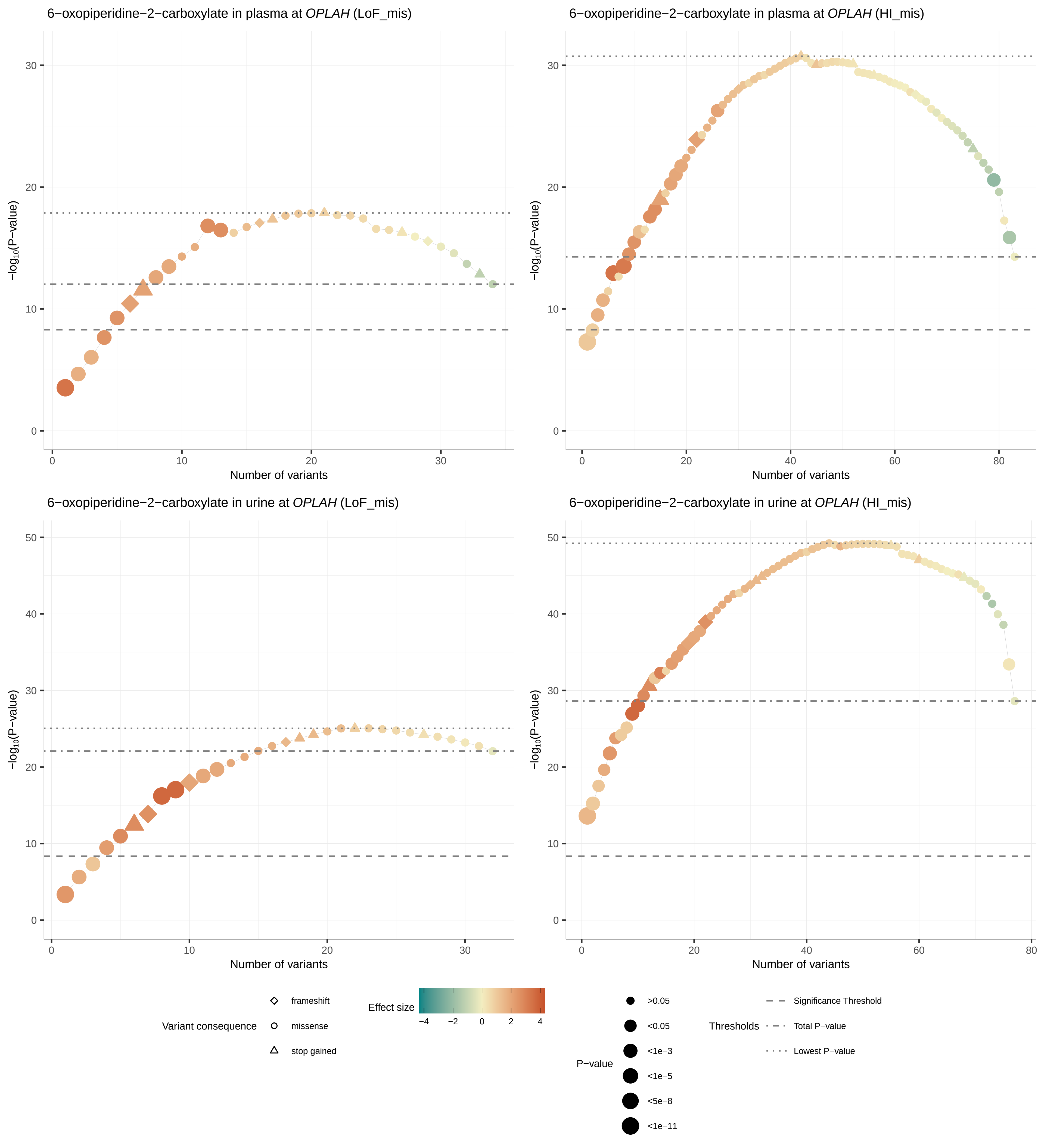
6−oxopiperidine−2−carboxylate in plasma at OPLAH (LoF_mis)
6−oxopiperidine−2−carboxylate in plasma at OPLAH (HI_mis)
6−oxopiperidine−2−carboxylate in urine at OPLAH (LoF_mis)
6−oxopiperidine−2−carboxylate in urine at OPLAH (HI_mis)
−log10(P−value)
Number of variants
Variant consequence
frameshift
missense
stop gained
Effect size
−4 −2 0 2 4
P−value
>0.05
<0.05
<1e−3
<1e−5
<5e−8
<1e−11
Thresholds
Significance Threshold
Total P−value
Lowest P−value

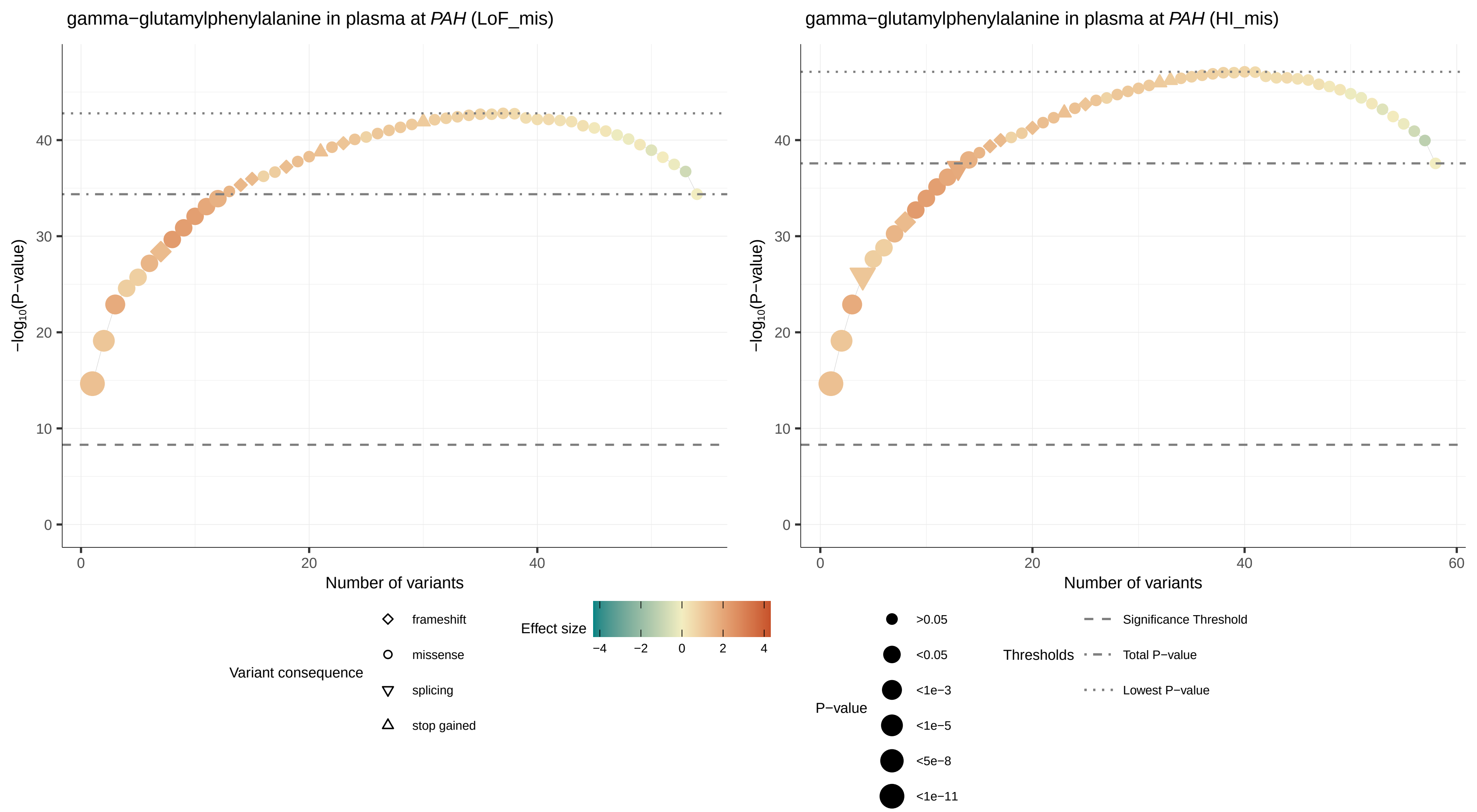

gamma−glutamylphenylalanine in plasma at PAH (LoF_mis)
gamma−glutamylphenylalanine in plasma at PAH (HI_mis)
−log10(P−value)
Number of variants
Variant consequence
frameshift
missense
splicing
stop gained
Effect size
−4
−2
0
2
4
P−value
>0.05
<0.05
<1e−3
<1e−5
<5e−8
<1e−11
Thresholds
Significance Threshold
Total P−value
Lowest P−value

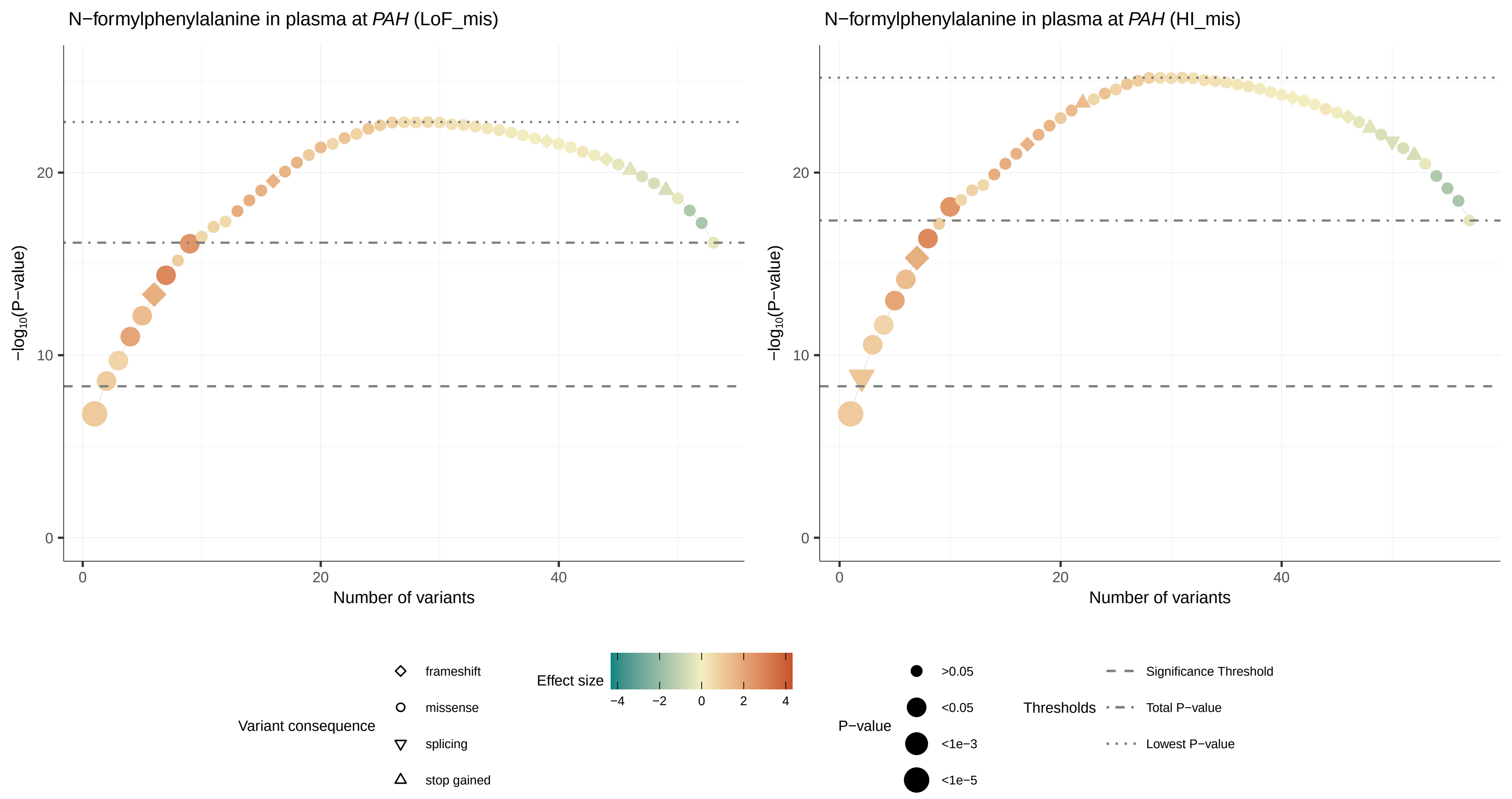

N−formylphenylalanine in plasma at PAH (LoF_mis)
N−formylphenylalanine in plasma at PAH (HI_mis)
−log10(P−value)
Number of variants
0
10
20
40
Variant consequence
frameshift
missense
splicing
stop gained
Effect size
−4
−2
2
4
P−value
>0.05
<0.05
<1e−3
<1e−5
Thresholds
Significance Threshold
Total P−value
Lowest P−value

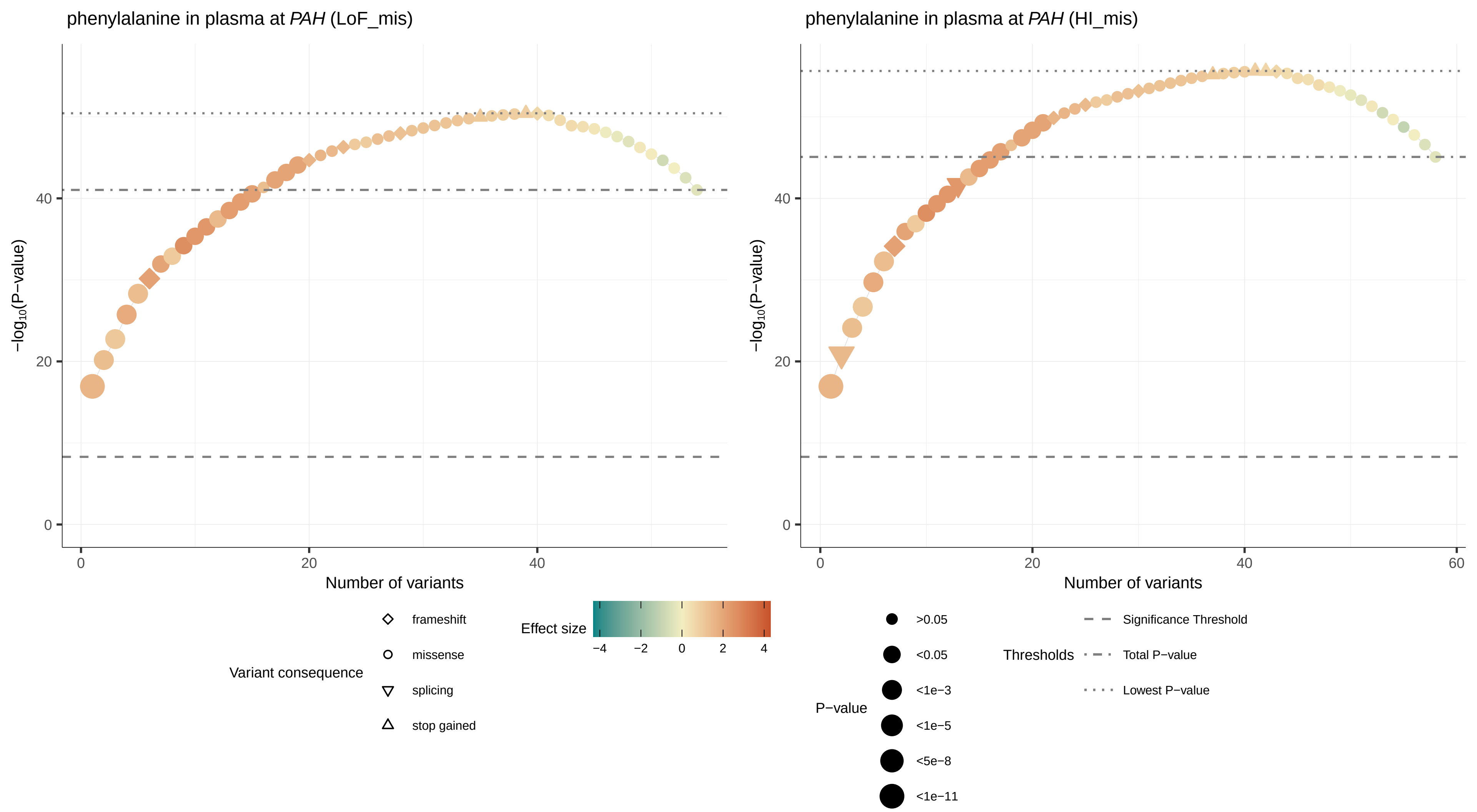

phenylalanine in plasma at PAH (LoF_mis)
phenylalanine in plasma at PAH (HI_mis)
−log10(P−value)
Number of variants
0
20
40
60
Variant consequence
frameshift
missense
splicing
stop gained
Effect size
−4
−2
0
2
4
P−value
>0.05
<0.05
<1e−3
<1e−5
<5e−8
<1e−11
Thresholds
Significance Threshold
Total P−value
Lowest P−value

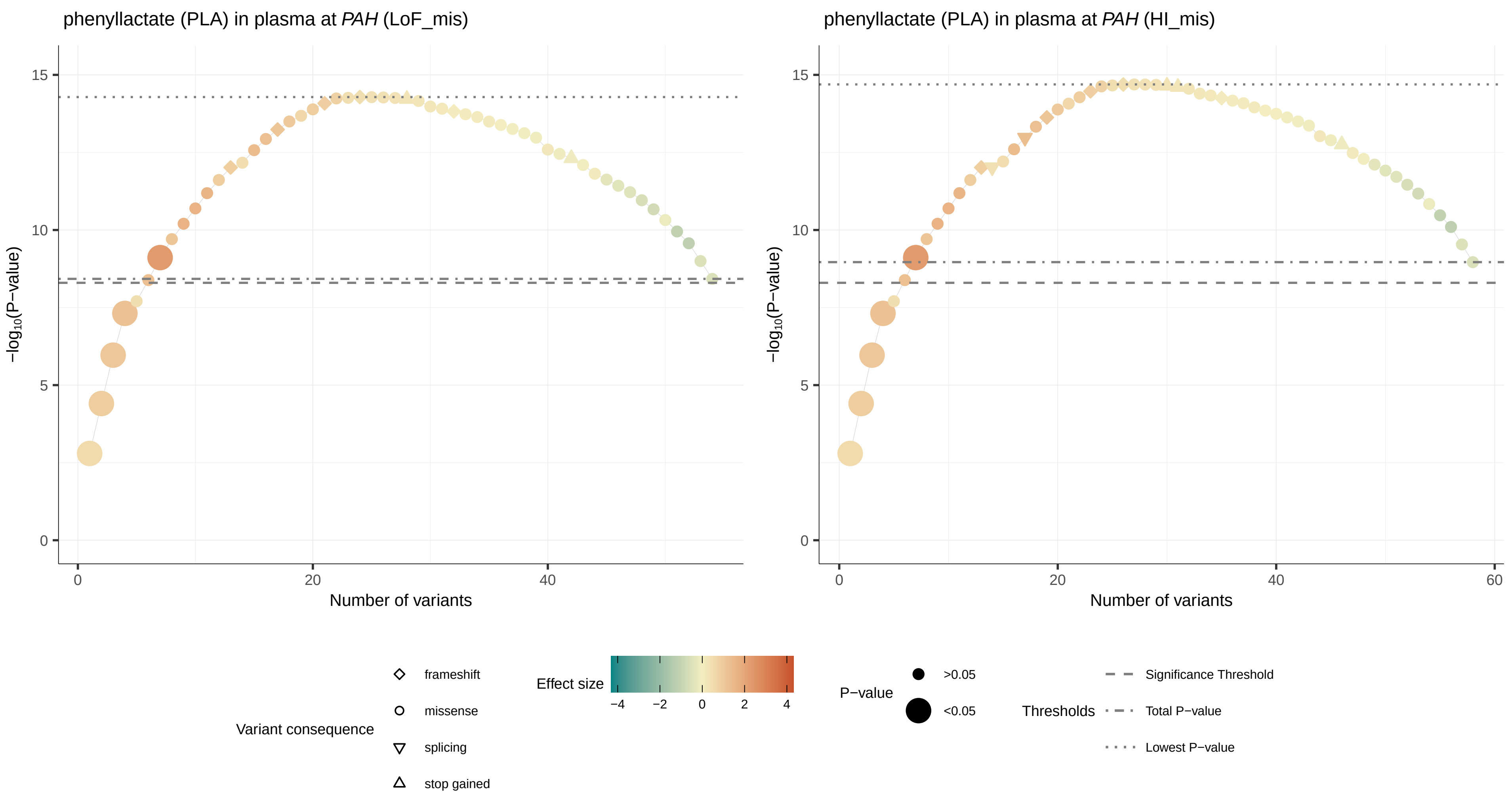

phenyllactate (PLA) in plasma at PAH (LoF_mis)
phenyllactate (PLA) in plasma at PAH (HI_mis)
−log10(P−value)
Number of variants
0
5
10
15
0
20
40
60
Variant consequence
frameshift
missense
splicing
stop gained
Effect size
−4
−2
0
2
4
P−value
>0.05
<0.05
Thresholds
Significance Threshold
Total P−value
Lowest P−value

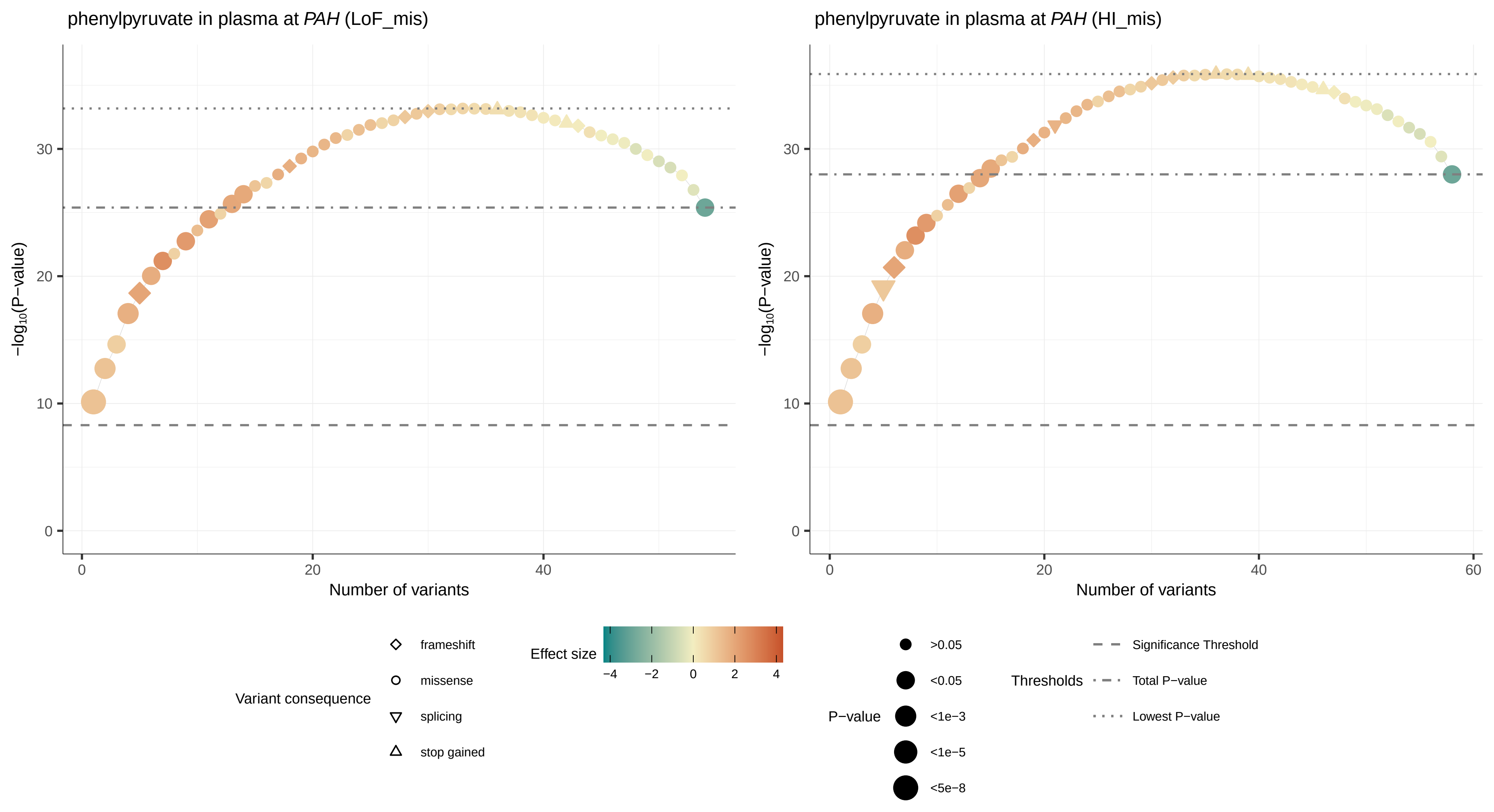

phenylpyruvate in plasma at PAH (LoF_mis)
phenylpyruvate in plasma at PAH (HI_mis)
−log10(P−value)
Number of variants
0
10
20
30
0
20
40
60
Variant consequence
frameshift
missense
splicing
stop gained
Effect size
−4
−2
0
2
4
P−value
>0.05
<0.05
<1e−3
<1e−5
<5e−8
Thresholds
Significance Threshold
Total P−value
Lowest P−value

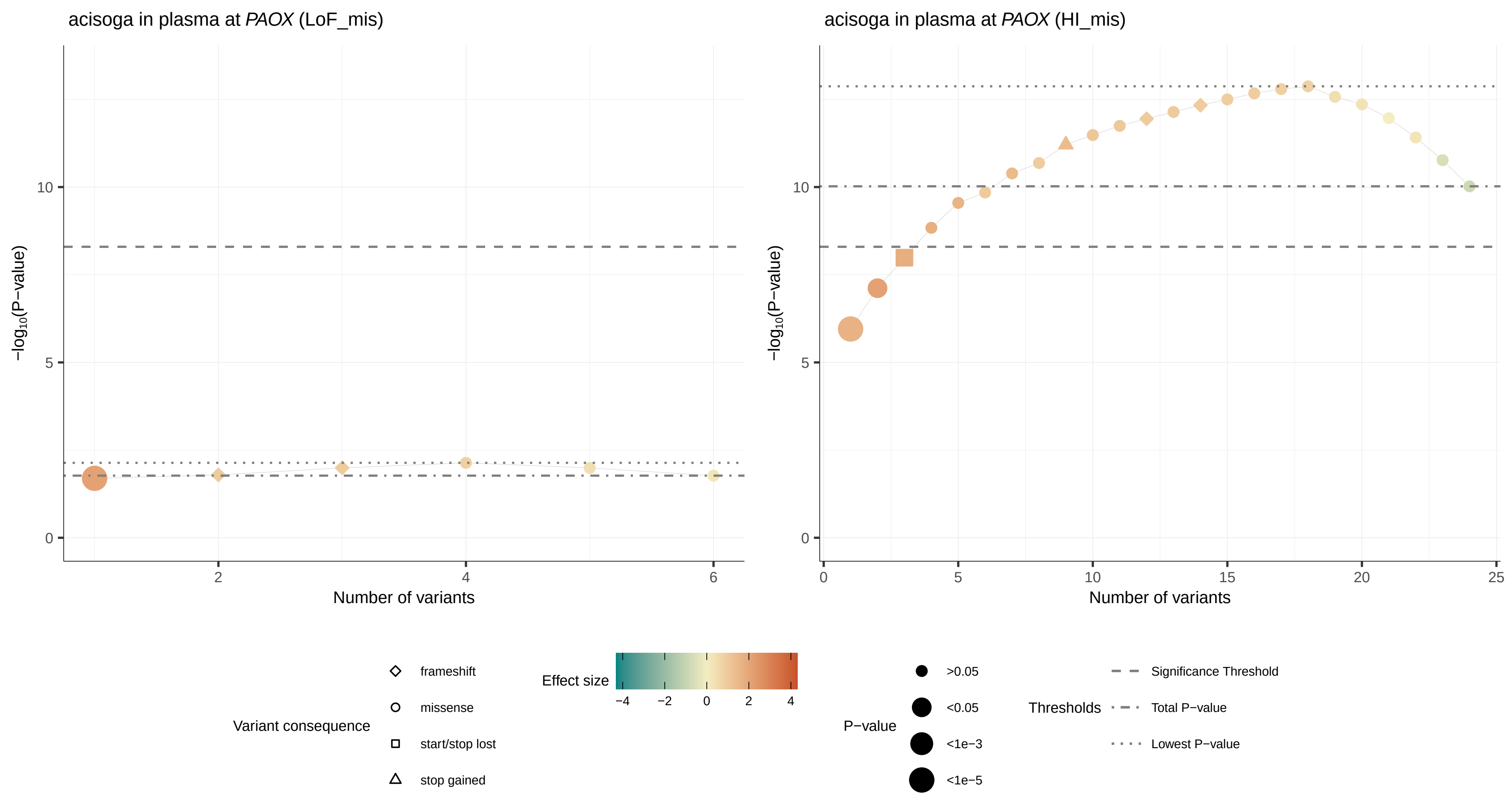

acisoga in plasma at PAOX (LoF_mis)
acisoga in plasma at PAOX (HI_mis)
−log10(P−value)
Number of variants
Variant consequence
frameshift
missense
start/stop lost
stop gained
Effect size
P−value
>0.05
<0.05
<1e−3
<1e−5
Thresholds
Significance Threshold
Total P−value
Lowest P−value

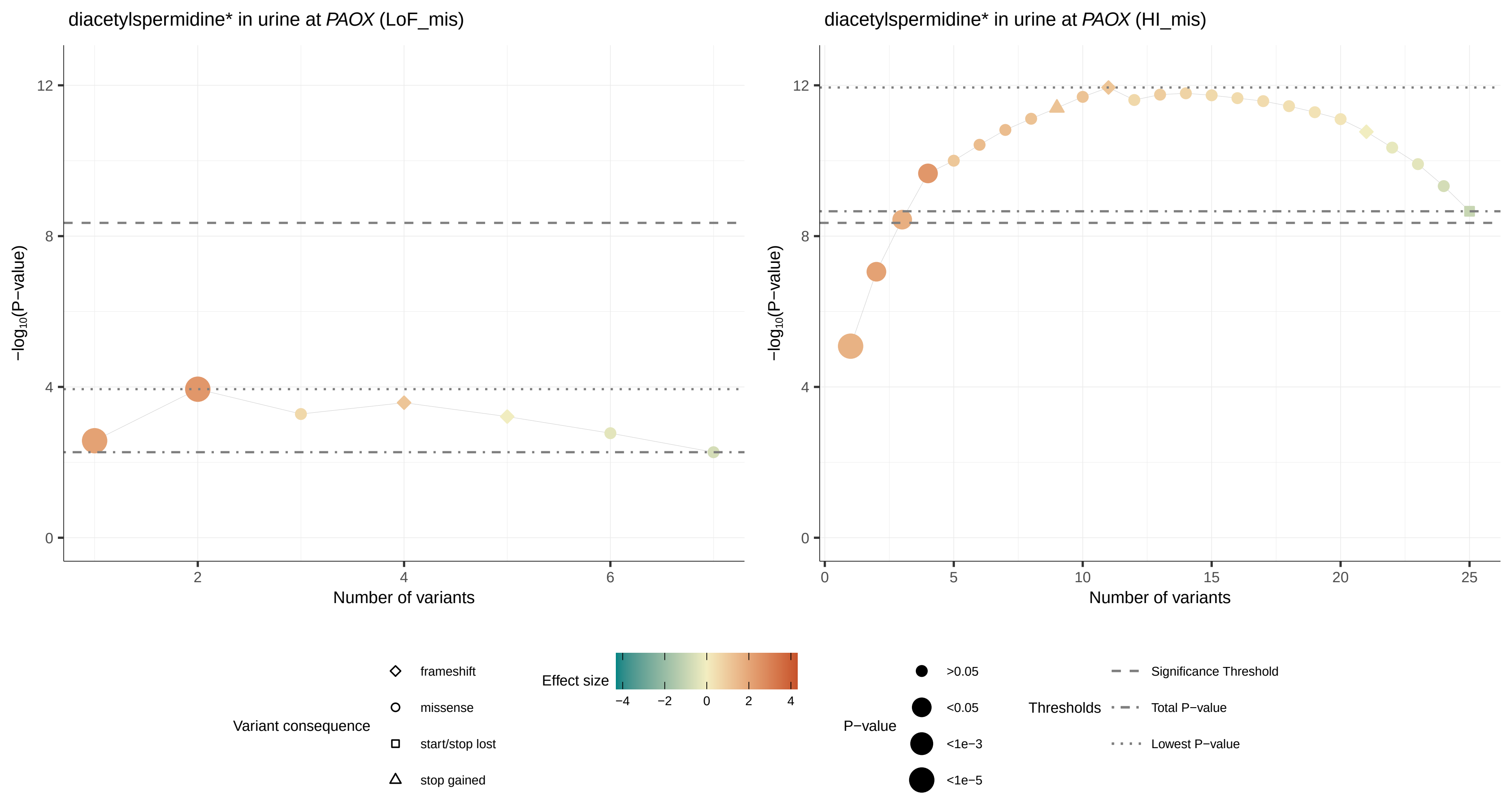

diacetylspermidine* in urine at *PAOX* (LoF_mis)
diacetylspermidine* in urine at *PAOX* (HI_mis)
−log10(P−value)
Number of variants
Variant consequence
frameshift
missense
start/stop lost
stop gained
Effect size
P−value
>0.05
<0.05
<1e−3
<1e−5
Thresholds
Significance Threshold
Total P−value
Lowest P−value

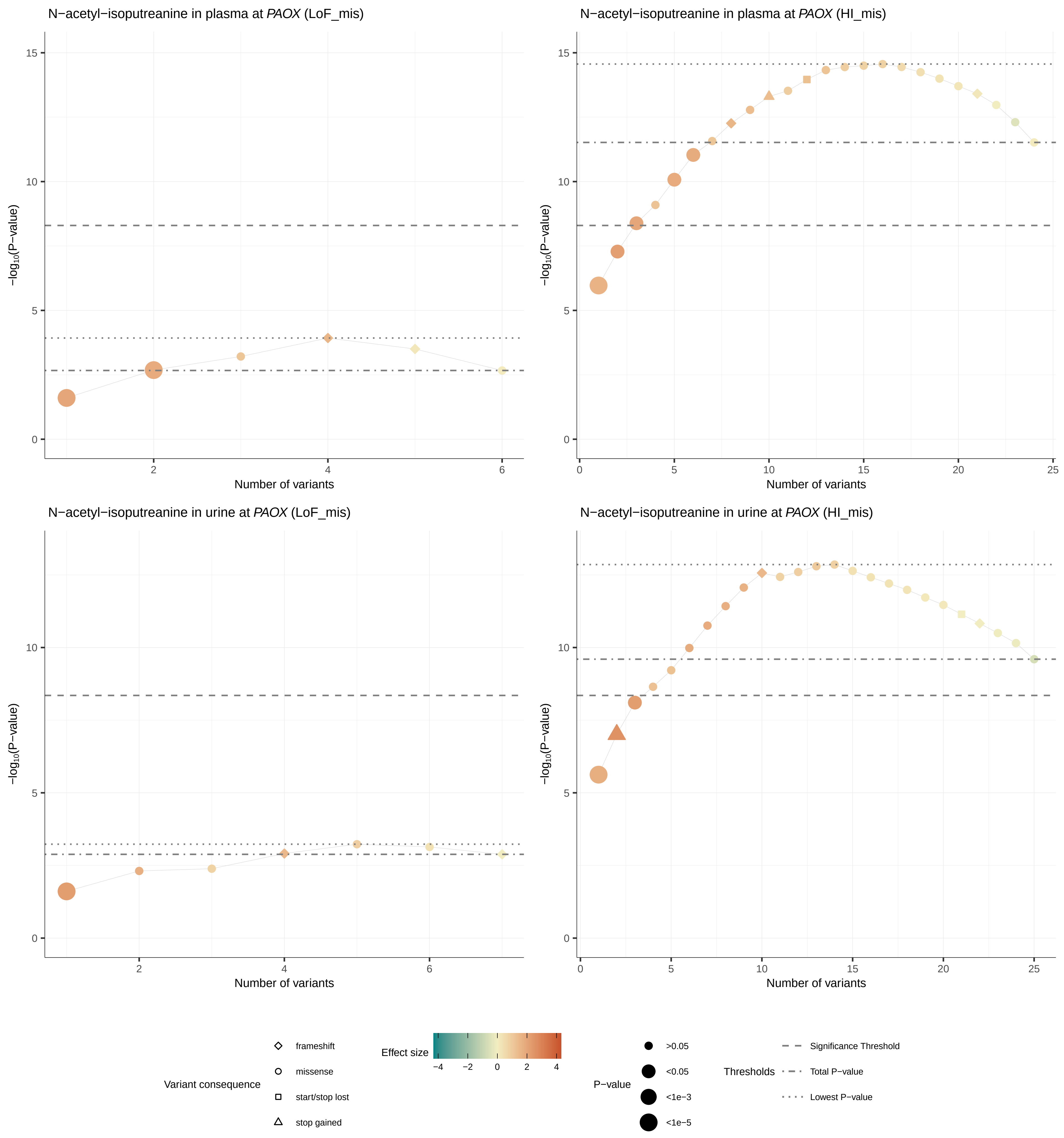

N−acetyl−isoputreanine in plasma at *PAOX* (LoF_mis)
N−acetyl−isoputreanine in plasma at *PAOX* (HI_mis)
N−acetyl−isoputreanine in urine at *PAOX* (LoF_mis)
N−acetyl−isoputreanine in urine at *PAOX* (HI_mis)
−log10(P−value)
Number of variants
Variant consequence
frameshift
missense
start/stop lost
stop gained
Effect size
−4
−2
0
2
4
P−value
>0.05
<0.05
<1e−3
<1e−5
Thresholds
Significance Threshold
Total P−value
Lowest P−value

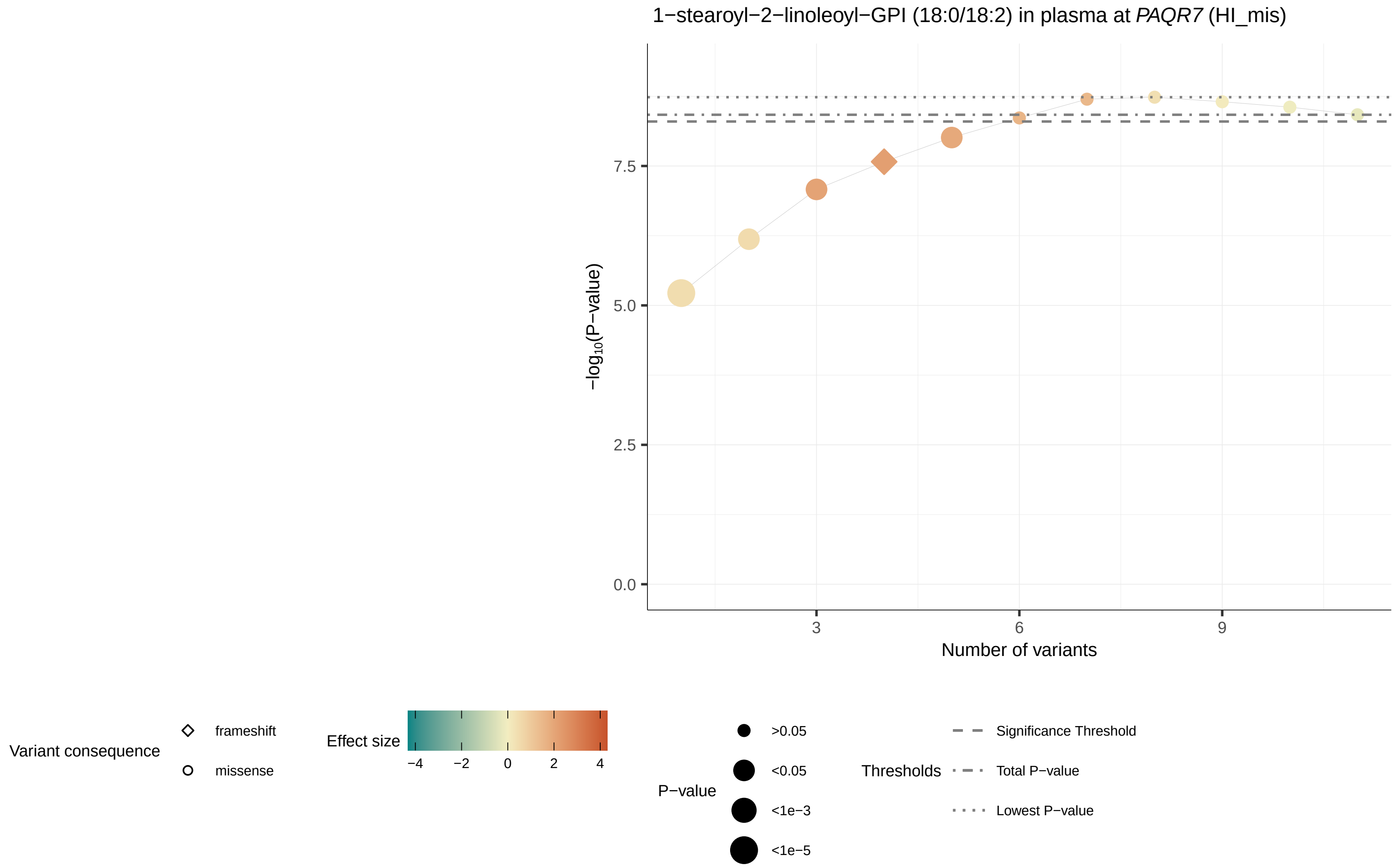
1−stearoyl−2−linoleoyl−GPI (18:0/18:2) in plasma at PAQR7 (HI_mis)
−log10(P−value)
0.0
2.5
5.0
7.5
3
6
9
Number of variants
Variant consequence
frameshift
missense
Effect size
−4
−2
0
2
4
P−value
>0.05
<0.05
<1e−3
<1e−5
Thresholds
Significance Threshold
Total P−value
Lowest P−value

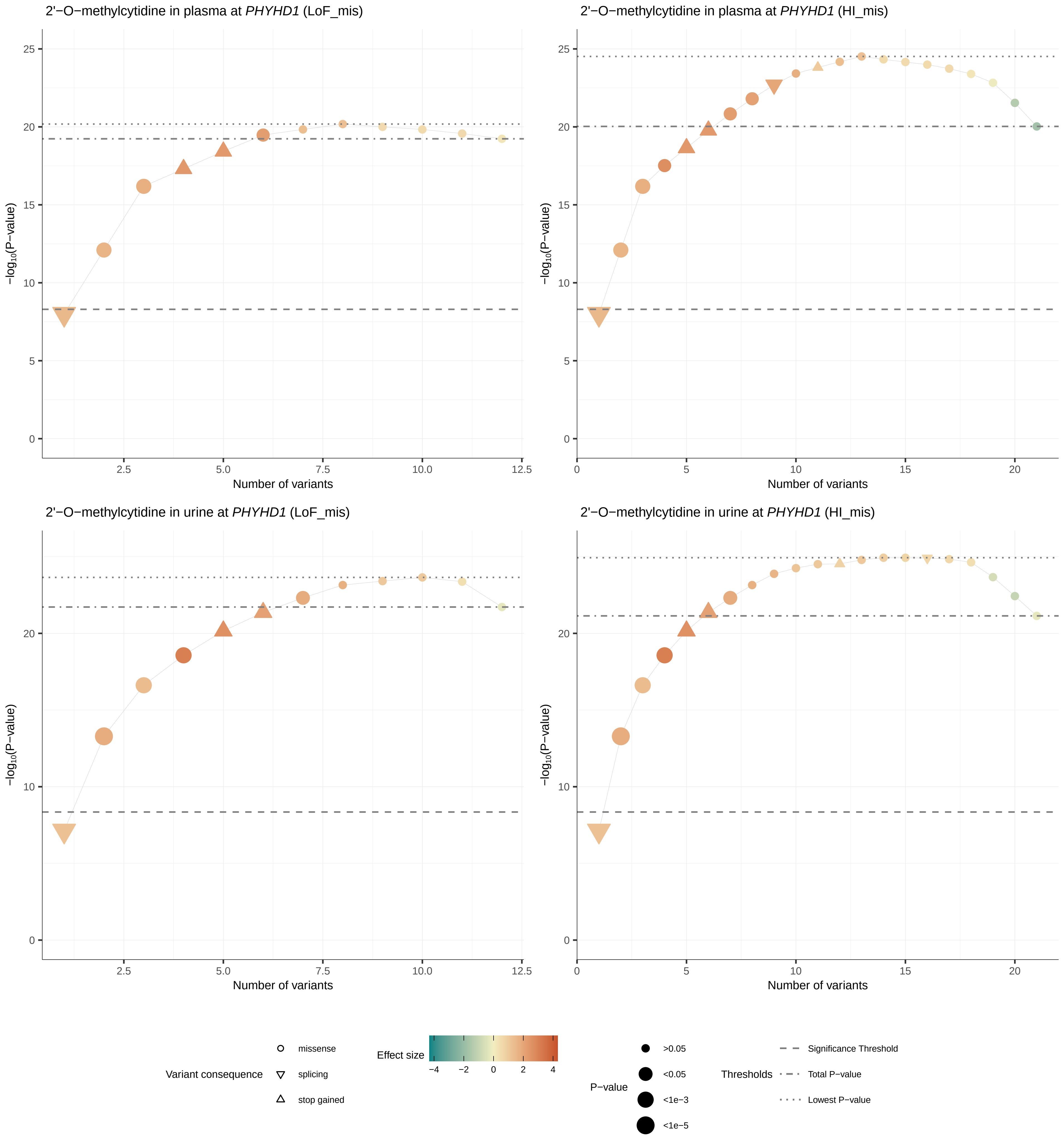
2'−O−methylcytidine in plasma at PHYHD1 (LoF_mis)
2'−O−methylcytidine in plasma at PHYHD1 (HI_mis)
2'−O−methylcytidine in urine at PHYHD1 (LoF_mis)
2'−O−methylcytidine in urine at PHYHD1 (HI_mis)
−log10(P−value)
Number of variants
Variant consequence
missense
splicing
stop gained
Effect size
P−value
>0.05
<0.05
<1e−3
<1e−5
Thresholds
Significance Threshold
Total P−value
Lowest P−value

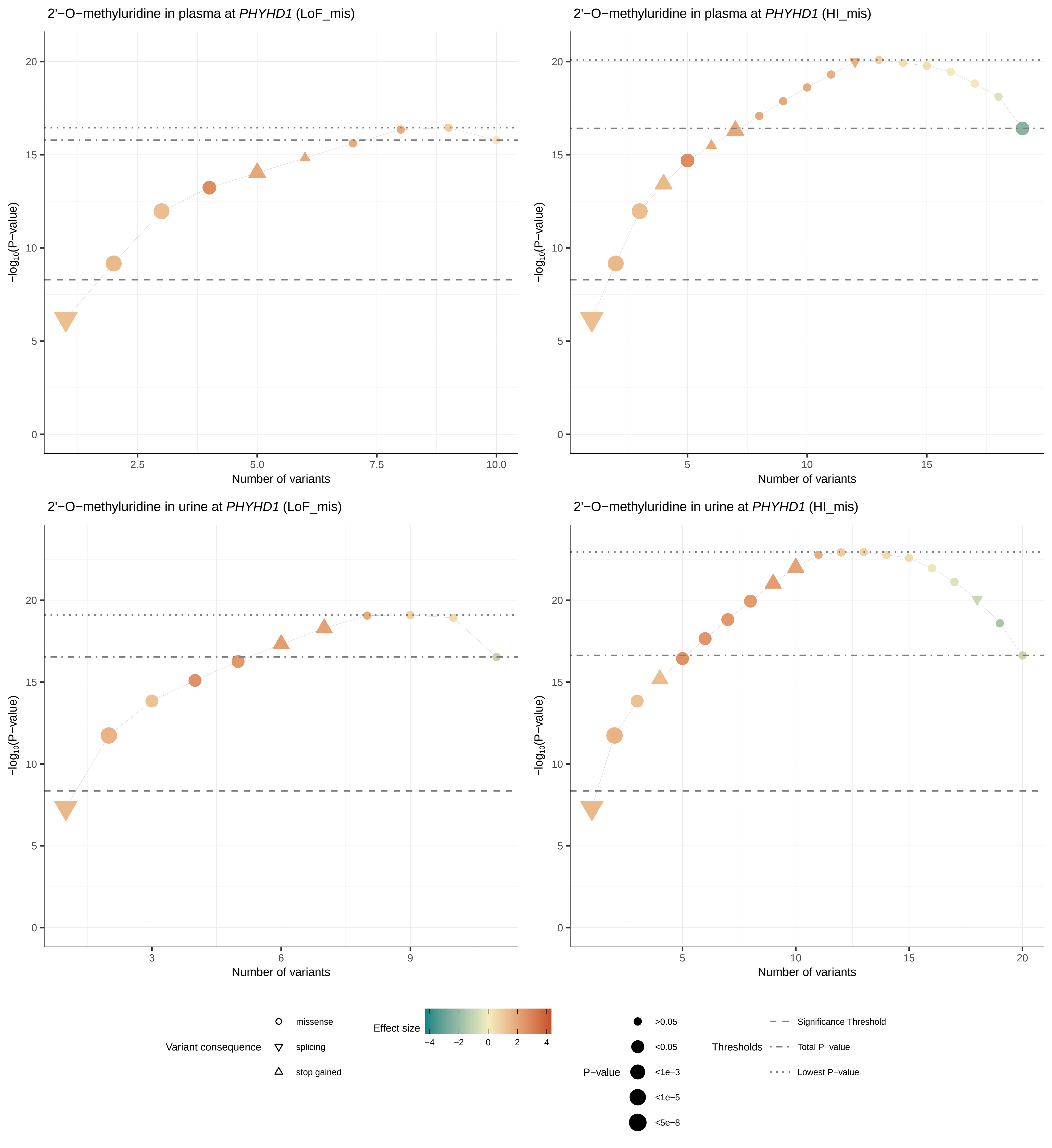

2'−O−methyluridine in plasma at PHYHD1 (LoF_mis)
2'−O−methyluridine in plasma at PHYHD1 (HI_mis)
2'−O−methyluridine in urine at PHYHD1 (LoF_mis)
2'−O−methyluridine in urine at PHYHD1 (HI_mis)
−log10(P−value)
Number of variants
Variant consequence
missense
splicing
stop gained
Effect size
P−value
>0.05
<0.05
<1e−3
<1e−5
<5e−8
Thresholds
Significance Threshold
Total P−value
Lowest P−value

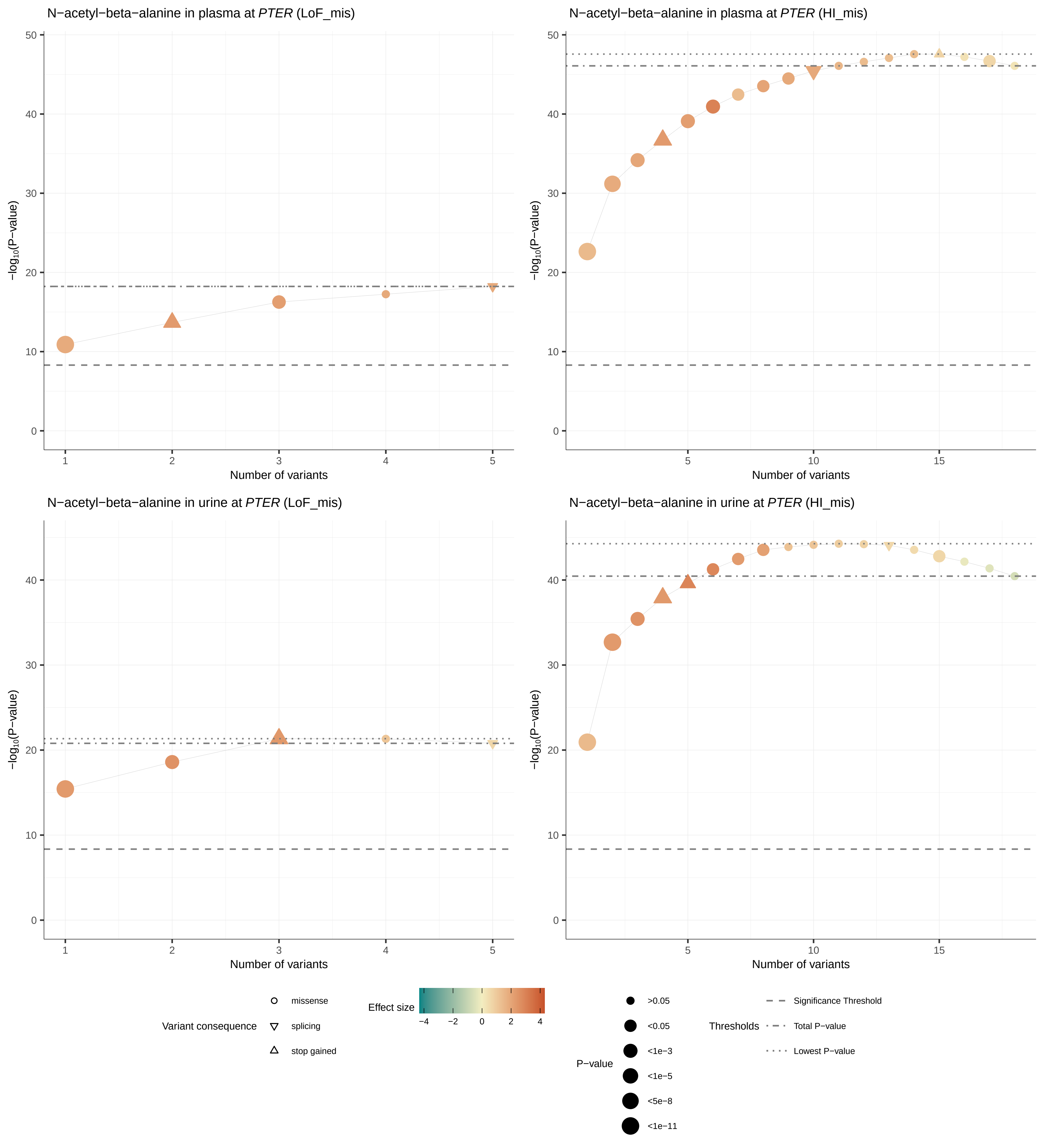

N−acetyl−beta−alanine in plasma at PTER (LoF_mis)
N−acetyl−beta−alanine in plasma at PTER (HI_mis)
N−acetyl−beta−alanine in urine at PTER (LoF_mis)
N−acetyl−beta−alanine in urine at PTER (HI_mis)
−log10(P−value)
Number of variants
Variant consequence
missense
splicing
stop gained
Effect size
−4 −2 0 2 4
P−value
>0.05
<0.05
<1e−3
<1e−5
<5e−8
<1e−11
Thresholds
Significance Threshold
Total P−value
Lowest P−value

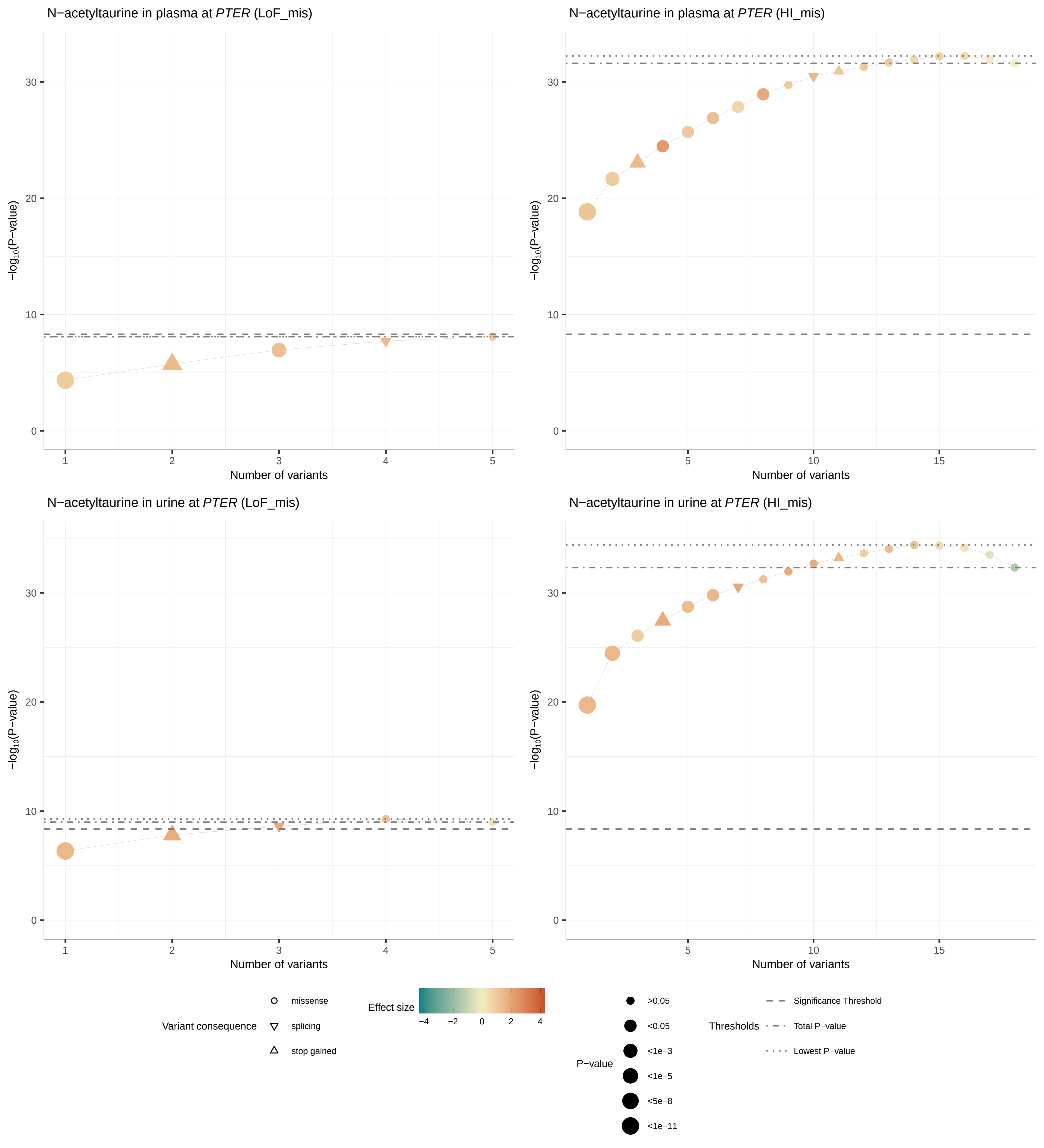

N−acetyltaurine in plasma at PTER (LoF_mis)
N−acetyltaurine in plasma at PTER (HI_mis)
N−acetyltaurine in urine at PTER (LoF_mis)
N−acetyltaurine in urine at PTER (HI_mis)
−log10(P−value)
Number of variants
Variant consequence
missense
splicing
stop gained
Effect size
−4 −2 0 2 4
P−value
>0.05
<0.05
<1e−3
<1e−5
<5e−8
<1e−11
Thresholds
Significance Threshold
Total P−value
Lowest P−value

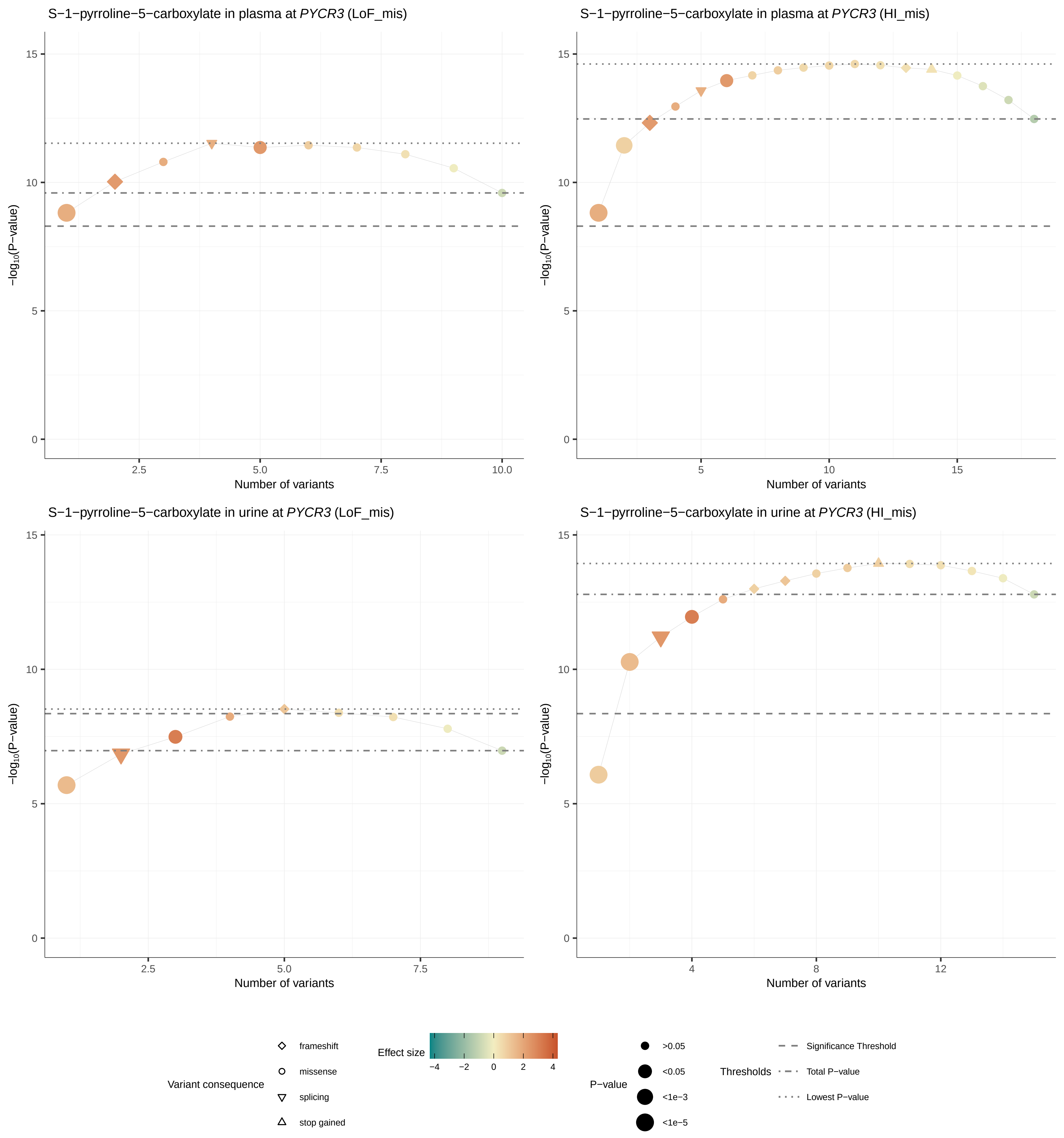

S−1−pyrroline−5−carboxylate in plasma at PYCR3 (LoF_mis)
S−1−pyrroline−5−carboxylate in plasma at PYCR3 (HI_mis)
S−1−pyrroline−5−carboxylate in urine at PYCR3 (LoF_mis)
S−1−pyrroline−5−carboxylate in urine at PYCR3 (HI_mis)
−log10(P−value)
Number of variants
Variant consequence
frameshift
missense
splicing
stop gained
Effect size
P−value
>0.05
<0.05
<1e−3
<1e−5
Thresholds
Significance Threshold
Total P−value
Lowest P−value

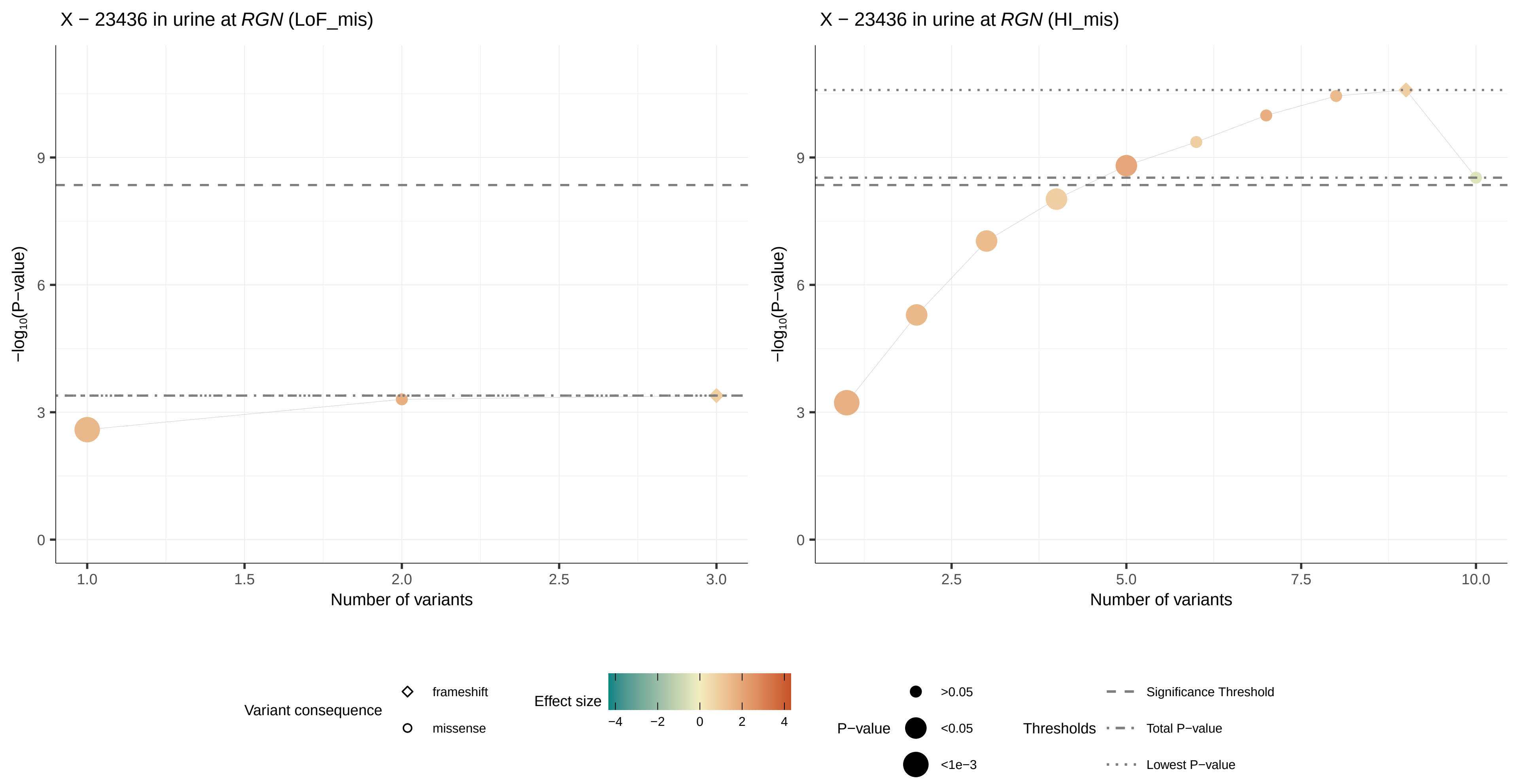

X − 23436 in urine at *RGN* (LoF_mis)
X − 23436 in urine at *RGN* (HI_mis)
−log10(P−value)
Number of variants
Variant consequence
frameshift
missense
Effect size
−4
−2
0
2
4
P−value
>0.05
<0.05
<1e−3
Thresholds
Significance Threshold
Total P−value
Lowest P−value

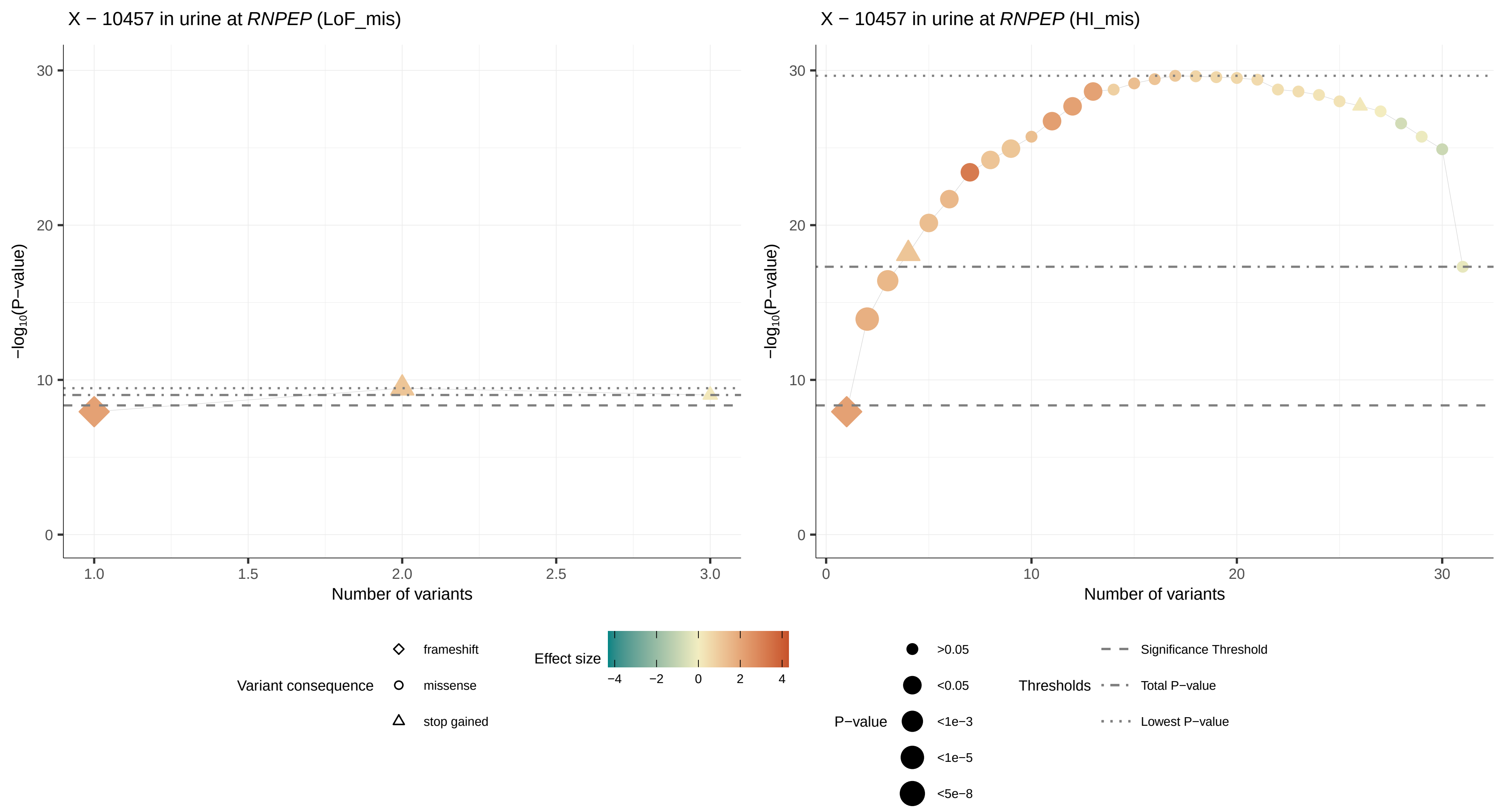
X − 10457 in urine at RNPEP (LoF_mis)
X − 10457 in urine at RNPEP (HI_mis)
−log10(P−value)
Number of variants
Variant consequence
frameshift
missense
stop gained
Effect size
P−value
>0.05
<0.05
<1e−3
<1e−5
<5e−8
Thresholds
Significance Threshold
Total P−value
Lowest P−value

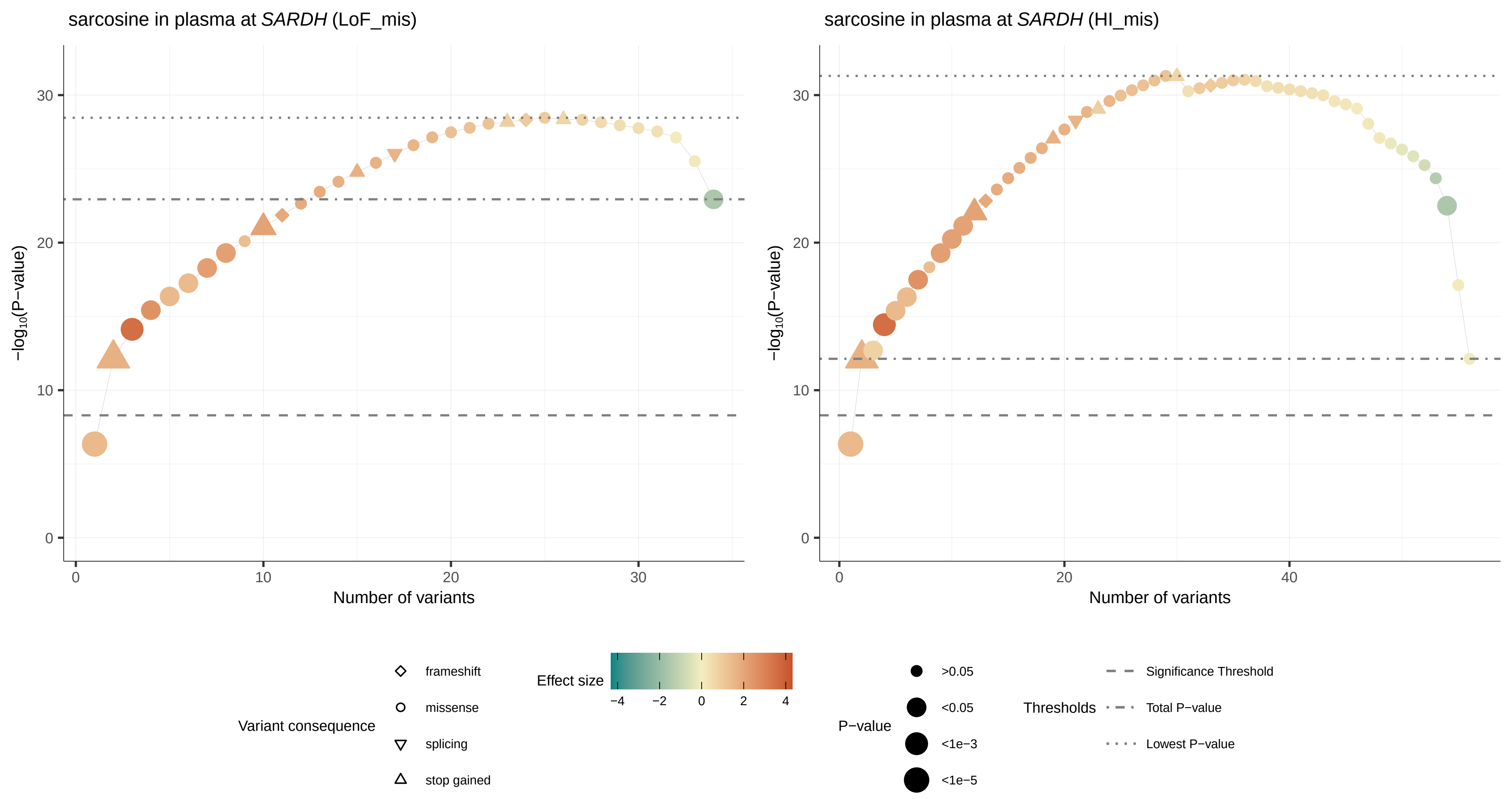
sarcosine in plasma at *SARDH* (LoF_mis)
sarcosine in plasma at *SARDH* (HI_mis)
−log10(P−value)
Number of variants
0
10
20
30
40
frameshift
missense
splicing
stop gained
Variant consequence
Effect size
−4
−2
0
2
4
P−value
>0.05
<0.05
<1e−3
<1e−5
Thresholds
Significance Threshold
Total P−value
Lowest P−value

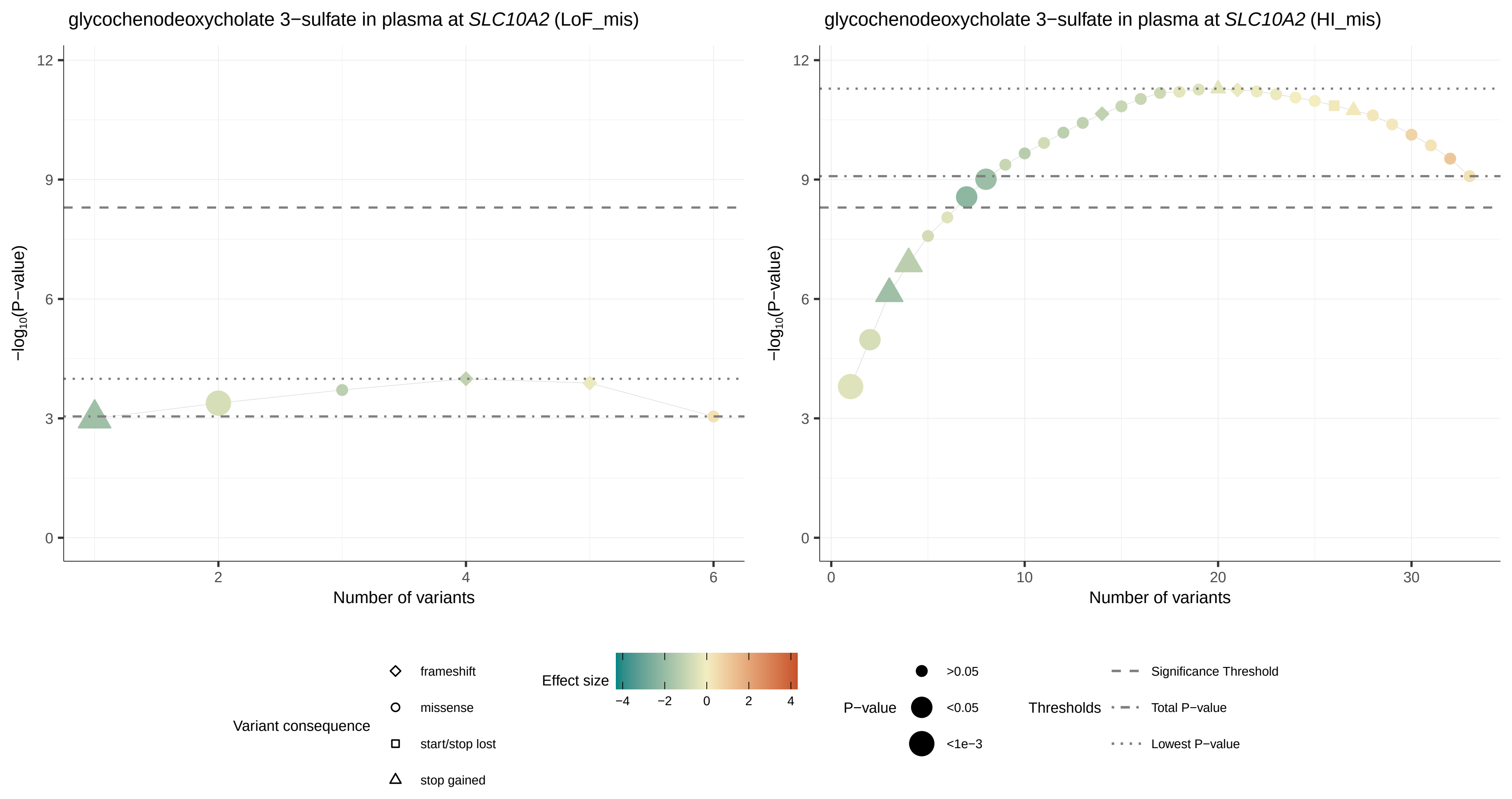

glycochenodeoxycholate 3−sulfate in plasma at SLC10A2 (LoF_mis)
glycochenodeoxycholate 3−sulfate in plasma at SLC10A2 (HI_mis)
−log10(P−value)
Number of variants
Variant consequence
frameshift
missense
start/stop lost
stop gained
Effect size
P−value
>0.05
<0.05
<1e−3
Thresholds
Significance Threshold
Total P−value
Lowest P−value

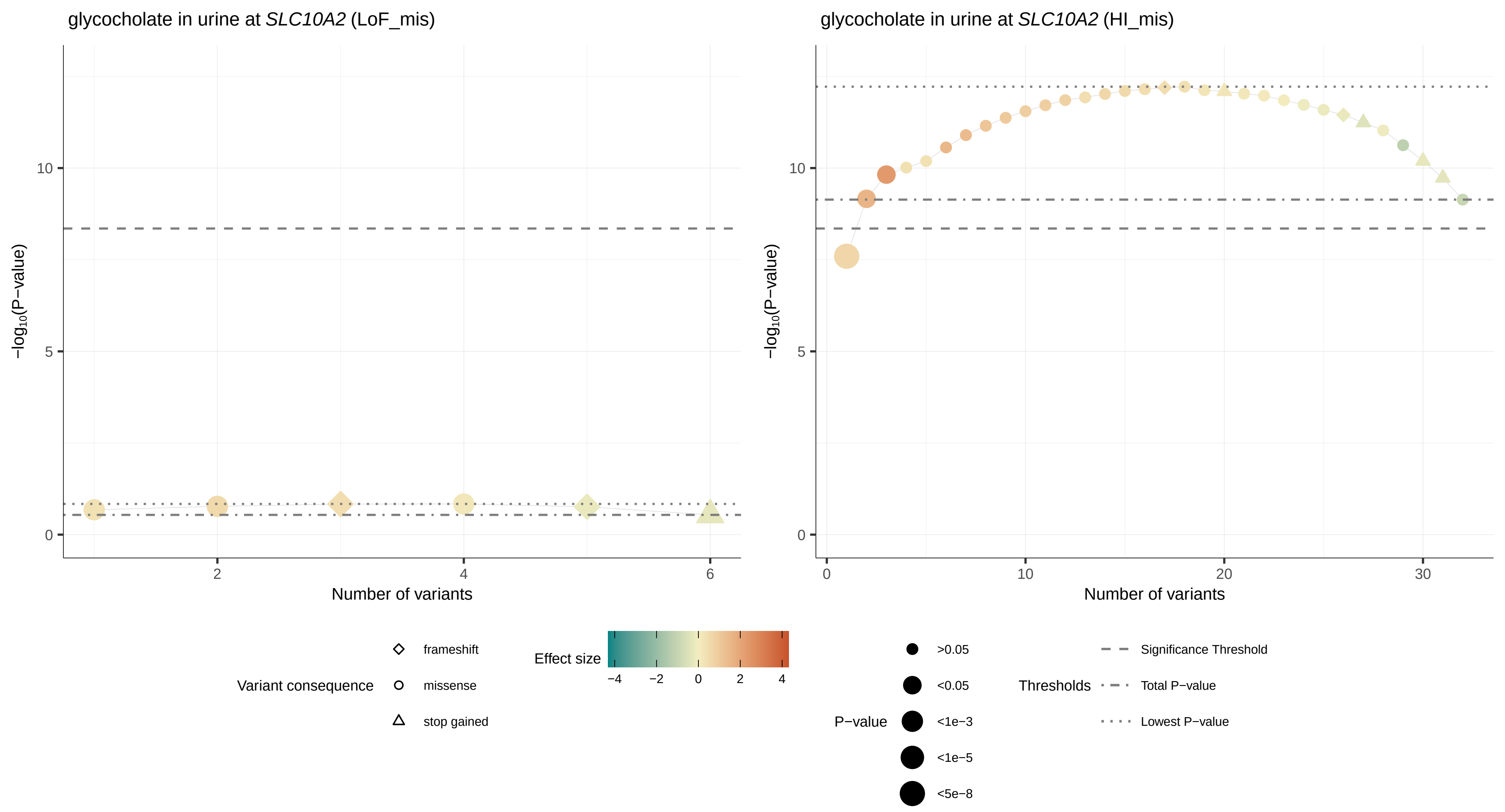

glycocholate in urine at SLC10A2 (LoF_mis)
glycocholate in urine at SLC10A2 (HI_mis)
−log10(P−value)
Number of variants
0
5
10
2
4
6
20
30
Variant consequence
frameshift
missense
stop gained
Effect size
−4
−2
0
2
4
P−value
>0.05
<0.05
<1e−3
<1e−5
<5e−8
Thresholds
Significance Threshold
Total P−value
Lowest P−value

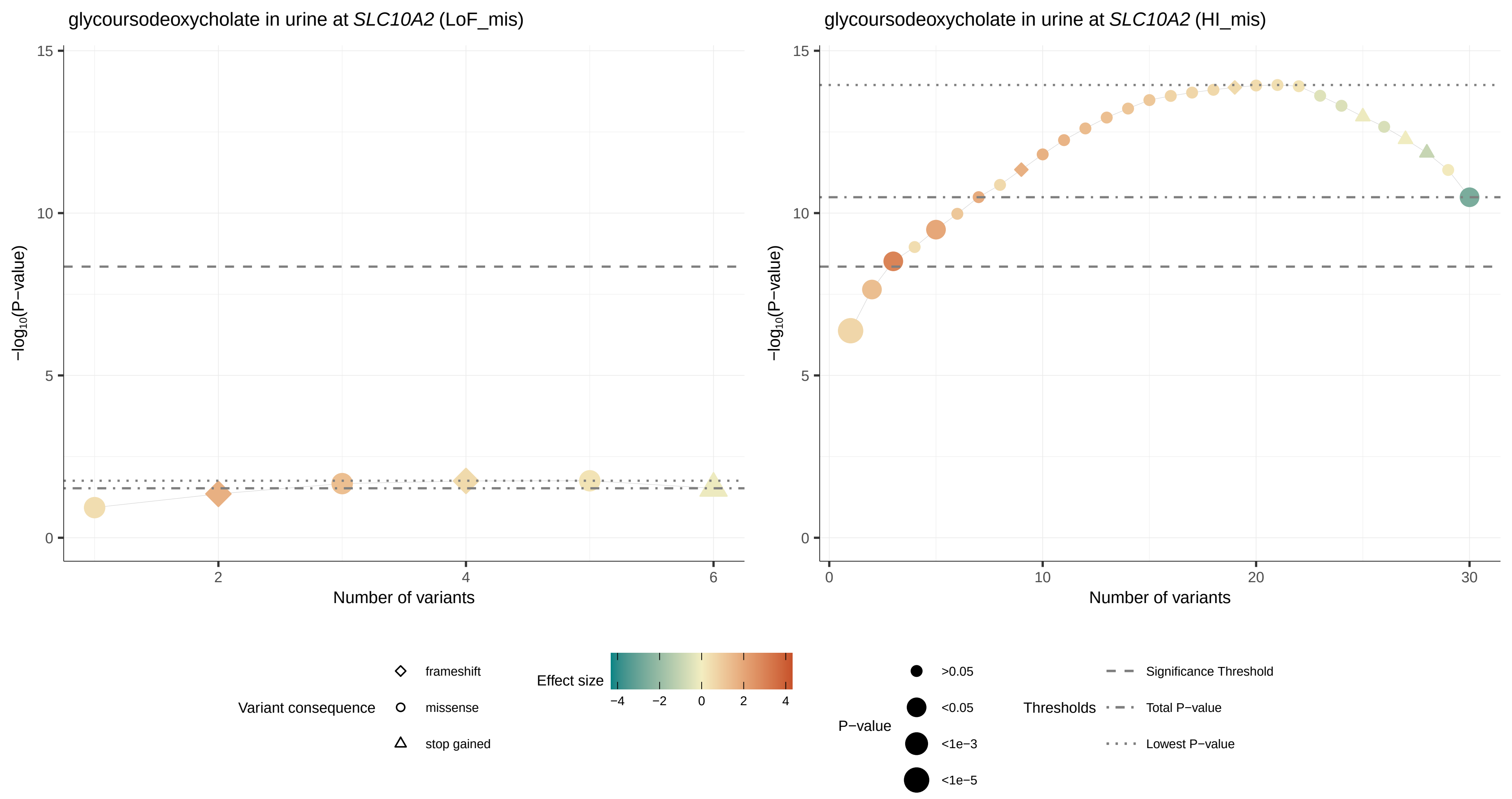
glycoursodeoxycholate in urine at *SLC10A2* (LoF_mis)
glycoursodeoxycholate in urine at *SLC10A2* (HI_mis)
−log10(P−value)
Number of variants
Variant consequence
frameshift
missense
stop gained
Effect size
−4
−2
0
2
4
P−value
>0.05
<0.05
<1e−3
<1e−5
Thresholds
Significance Threshold
Total P−value
Lowest P−value

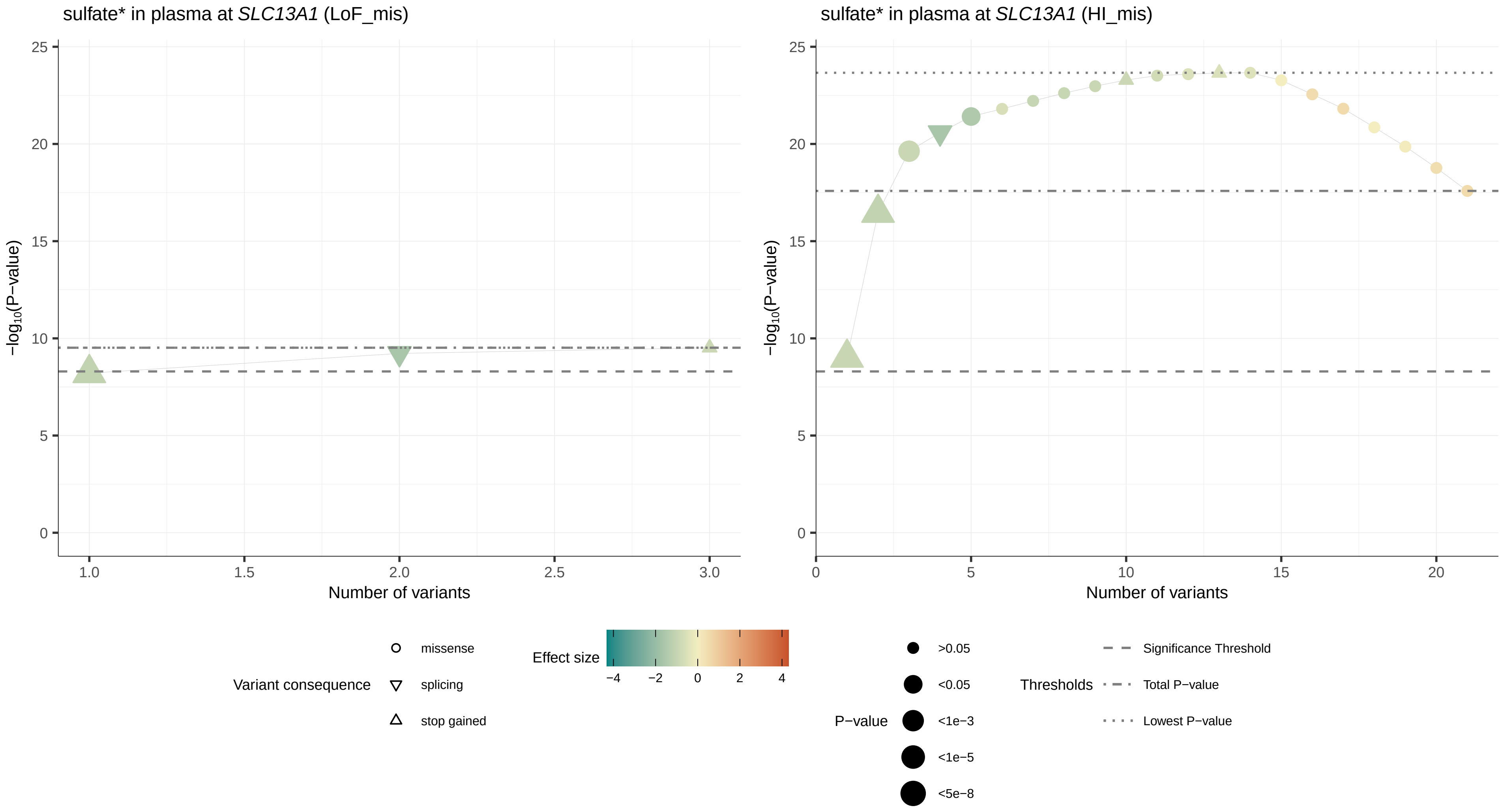

sulfate* in plasma at SLC13A1 (LoF_mis)
sulfate* in plasma at SLC13A1 (HI_mis)
−log10(P−value)
Number of variants
Variant consequence
missense
splicing
stop gained
Effect size
−4
−2
0
2
4
P−value
>0.05
<0.05
<1e−3
<1e−5
<5e−8
Thresholds
Significance Threshold
Total P−value
Lowest P−value

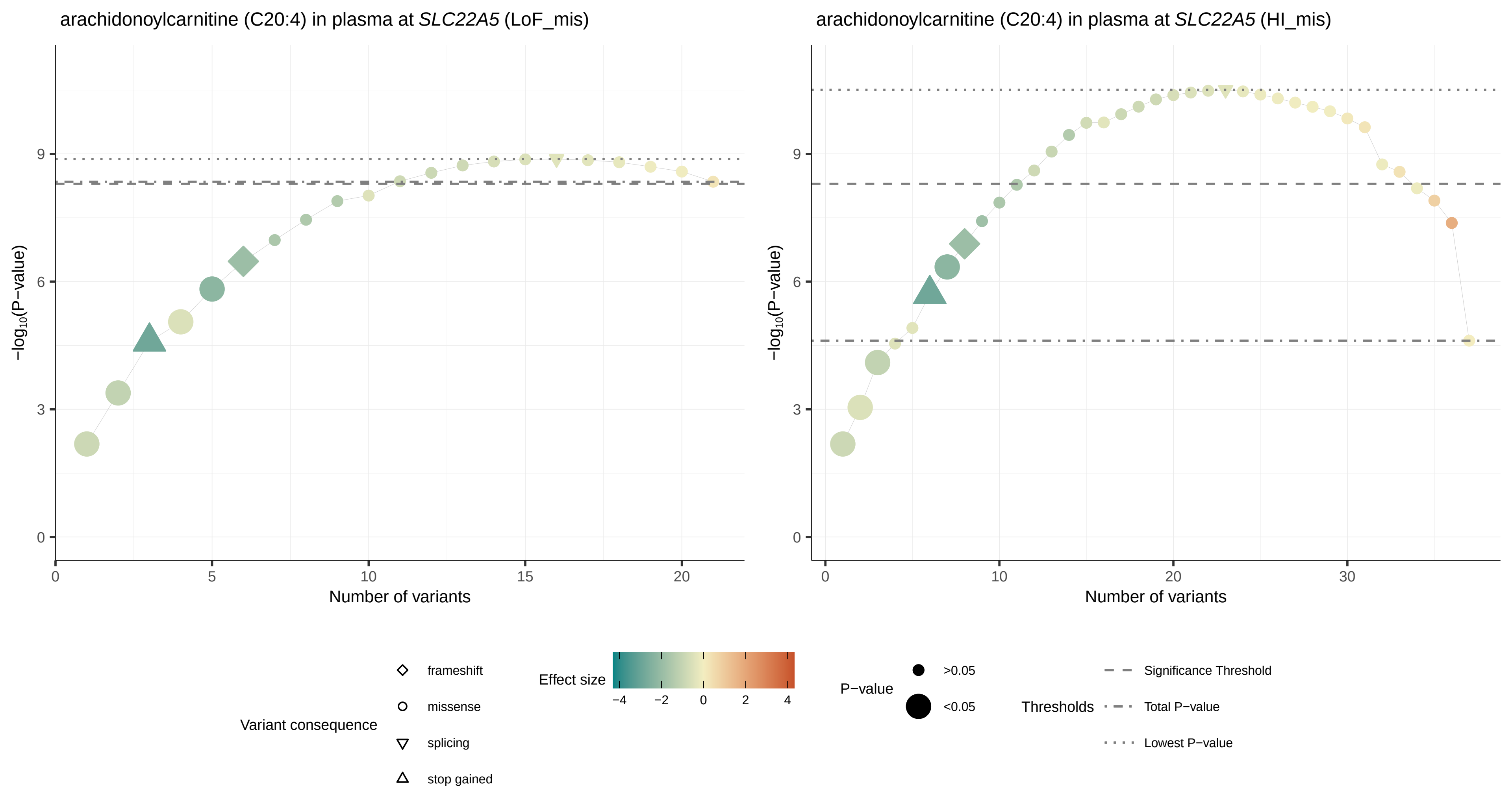

arachidonoylcarnitine (C20:4) in plasma at SLC22A5 (LoF_mis)
arachidonoylcarnitine (C20:4) in plasma at SLC22A5 (HI_mis)
−log10(P−value)
Number of variants
Variant consequence
frameshift
missense
splicing
stop gained
Effect size
−4
−2
0
2
4
P−value
>0.05
<0.05
Thresholds
Significance Threshold
Total P−value
Lowest P−value

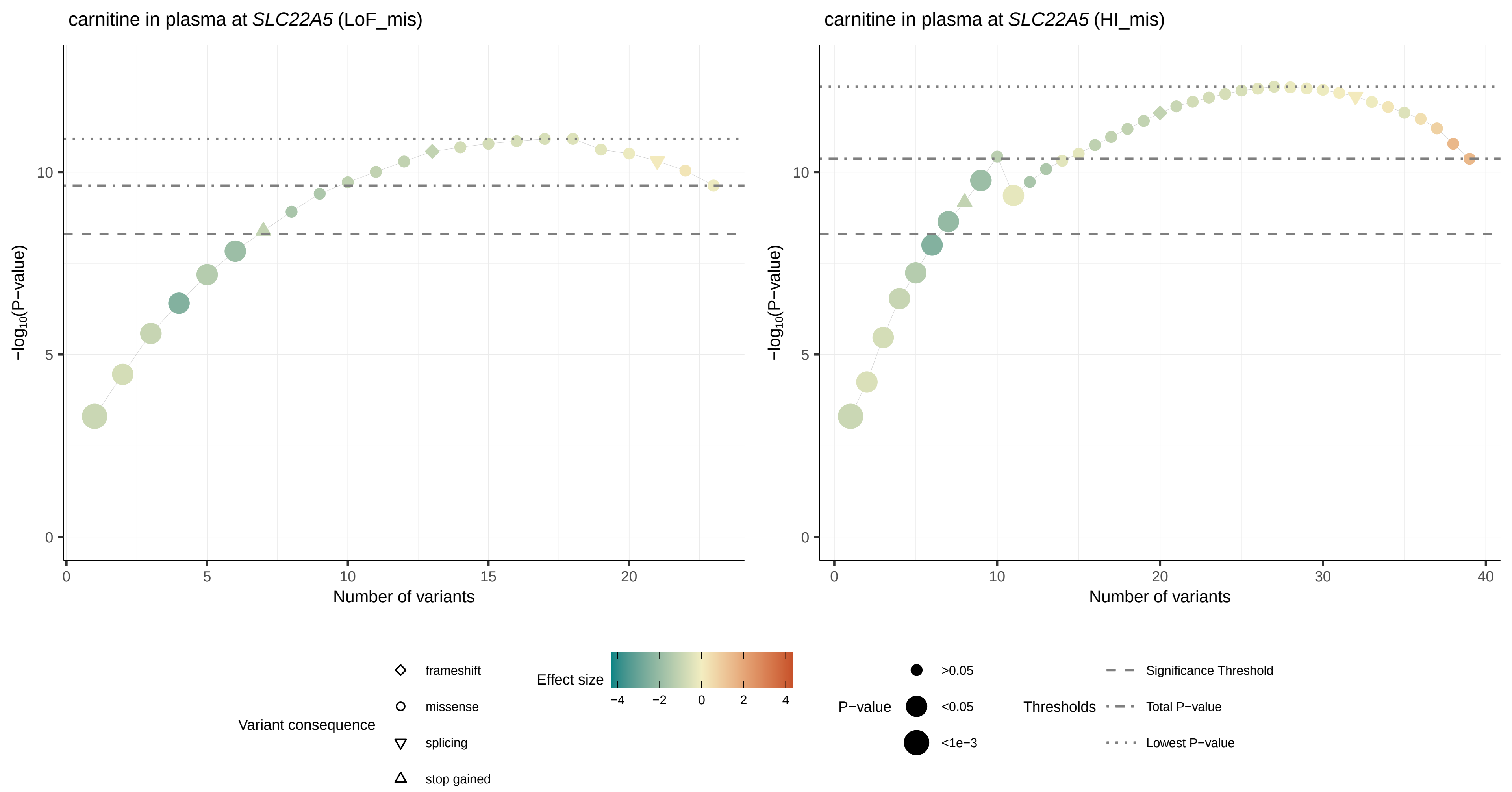

carnitine in plasma at SLC22A5 (LoF_mis)
carnitine in plasma at SLC22A5 (HI_mis)
−log10(P−value)
Number of variants
0
5
10
15
20
30
40
Variant consequence
frameshift
missense
splicing
stop gained
Effect size
−4
−2
0
2
4
P−value
>0.05
<0.05
<1e−3
Thresholds
Significance Threshold
Total P−value
Lowest P−value

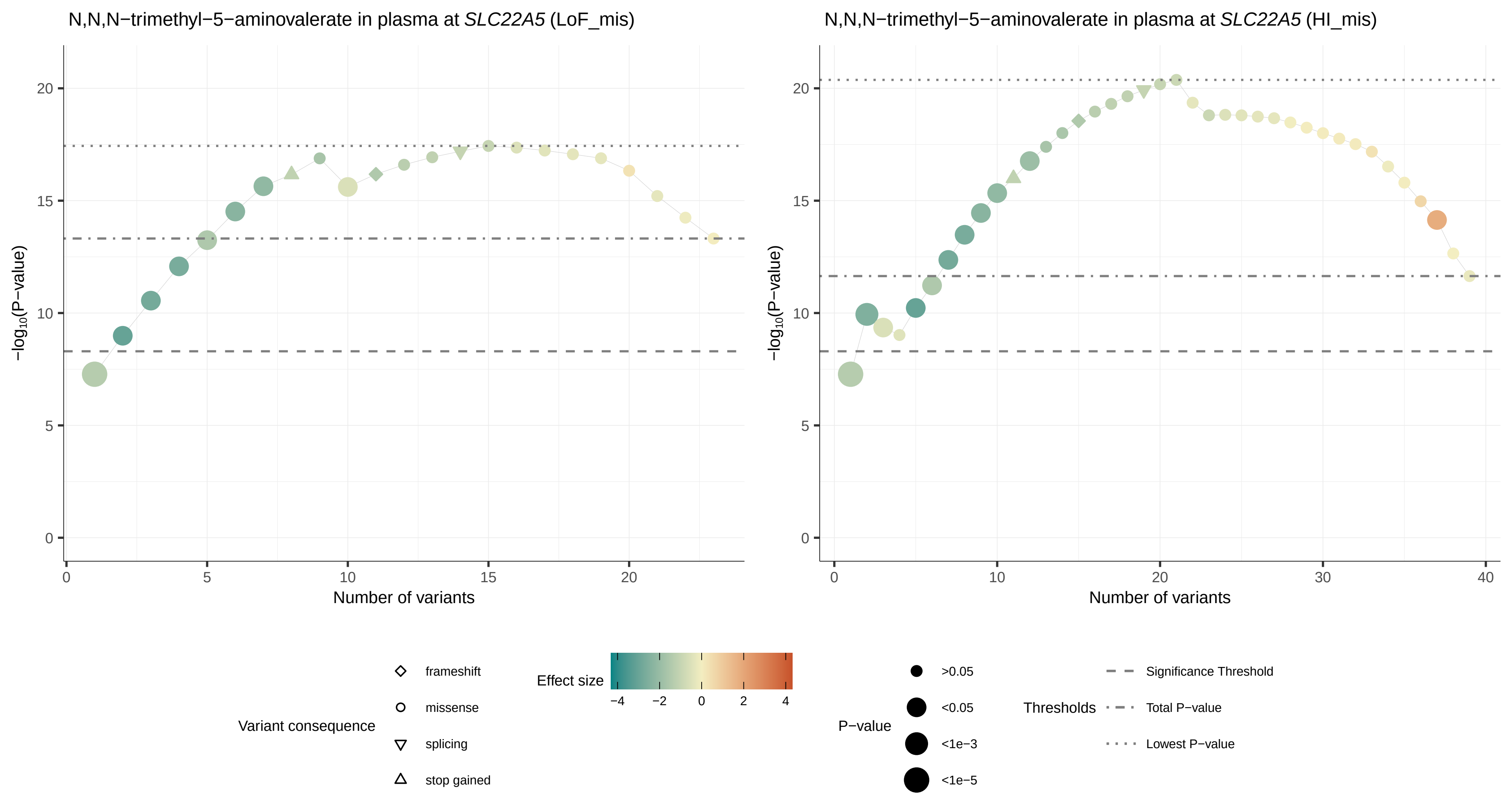
N,N,N−trimethyl−5−aminovalerate in plasma at SLC22A5 (LoF_mis)
N,N,N−trimethyl−5−aminovalerate in plasma at SLC22A5 (HI_mis)
−log10(P−value)
Number of variants
Variant consequence
frameshift
missense
splicing
stop gained
Effect size
−4
−2
0
2
4
P−value
>0.05
<0.05
<1e−3
<1e−5
Thresholds
Significance Threshold
Total P−value
Lowest P−value

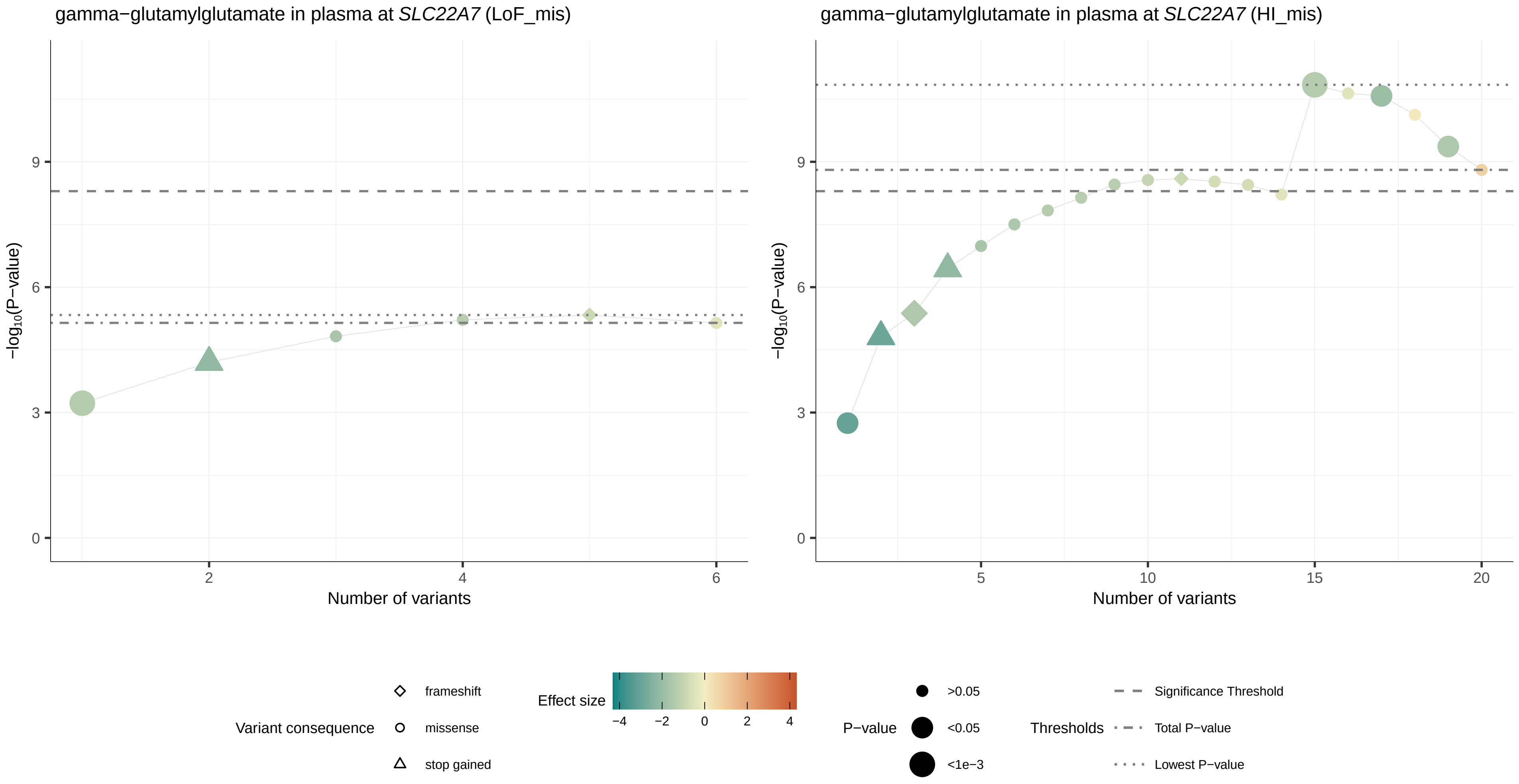

gamma−glutamylglutamate in plasma at SLC22A7 (LoF_mis)
gamma−glutamylglutamate in plasma at SLC22A7 (HI_mis)
−log10(P−value)
Number of variants
0
3
6
9
2
4
6
5
10
15
20
Variant consequence
frameshift
missense
stop gained
Effect size
−4
−2
0
2
4
P−value
>0.05
<0.05
<1e−3
Thresholds
Significance Threshold
Total P−value
Lowest P−value

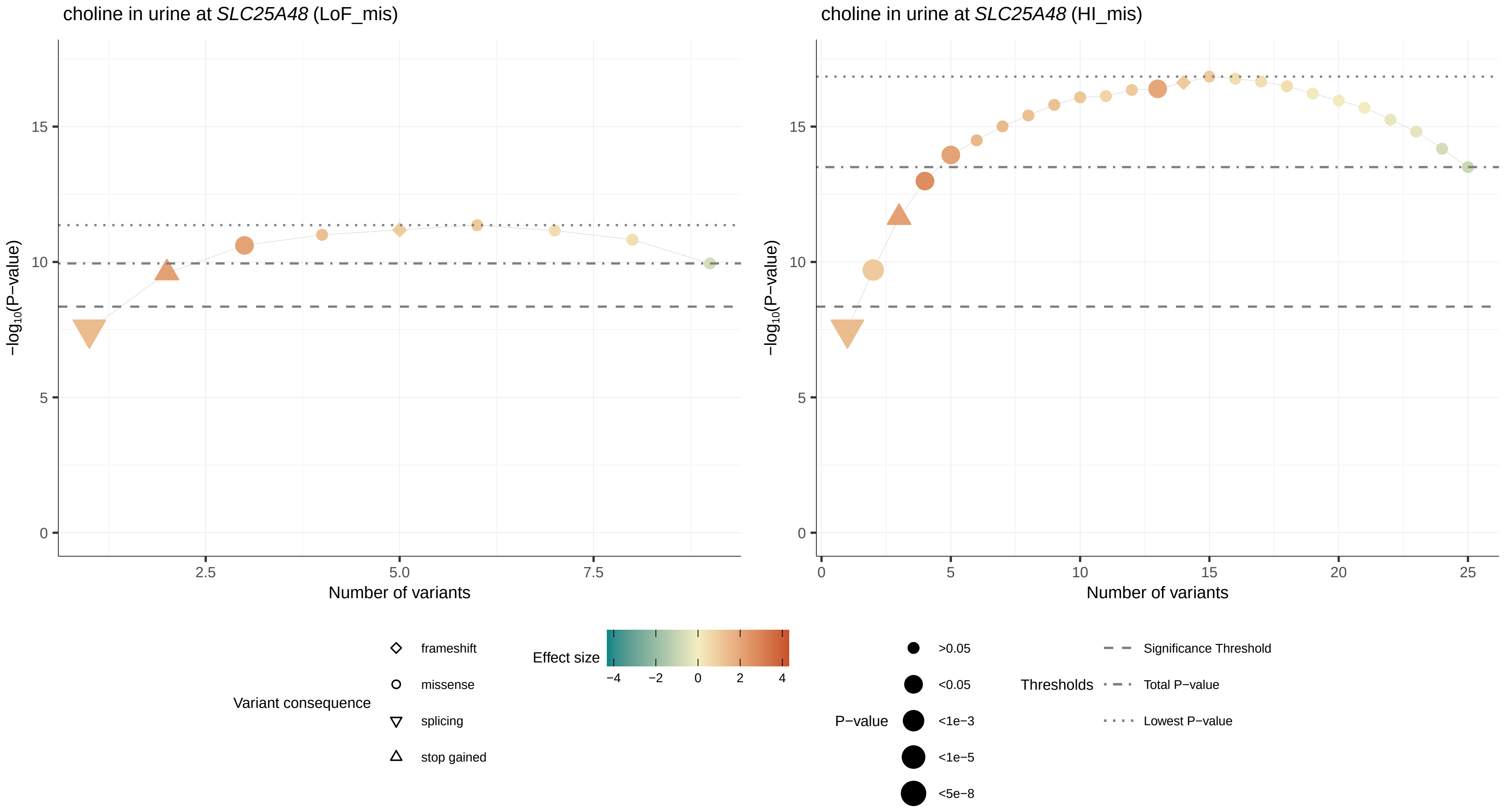

choline in urine at SLC25A48 (LoF_mis)
choline in urine at SLC25A48 (HI_mis)
−log10(P−value)
Number of variants
0
5
10
15
2.5
5.0
7.5
20
25
Variant consequence
frameshift
missense
splicing
stop gained
Effect size
−4
−2
0
2
4
P−value
>0.05
<0.05
<1e−3
<1e−5
<5e−8
Thresholds
Significance Threshold
Total P−value
Lowest P−value

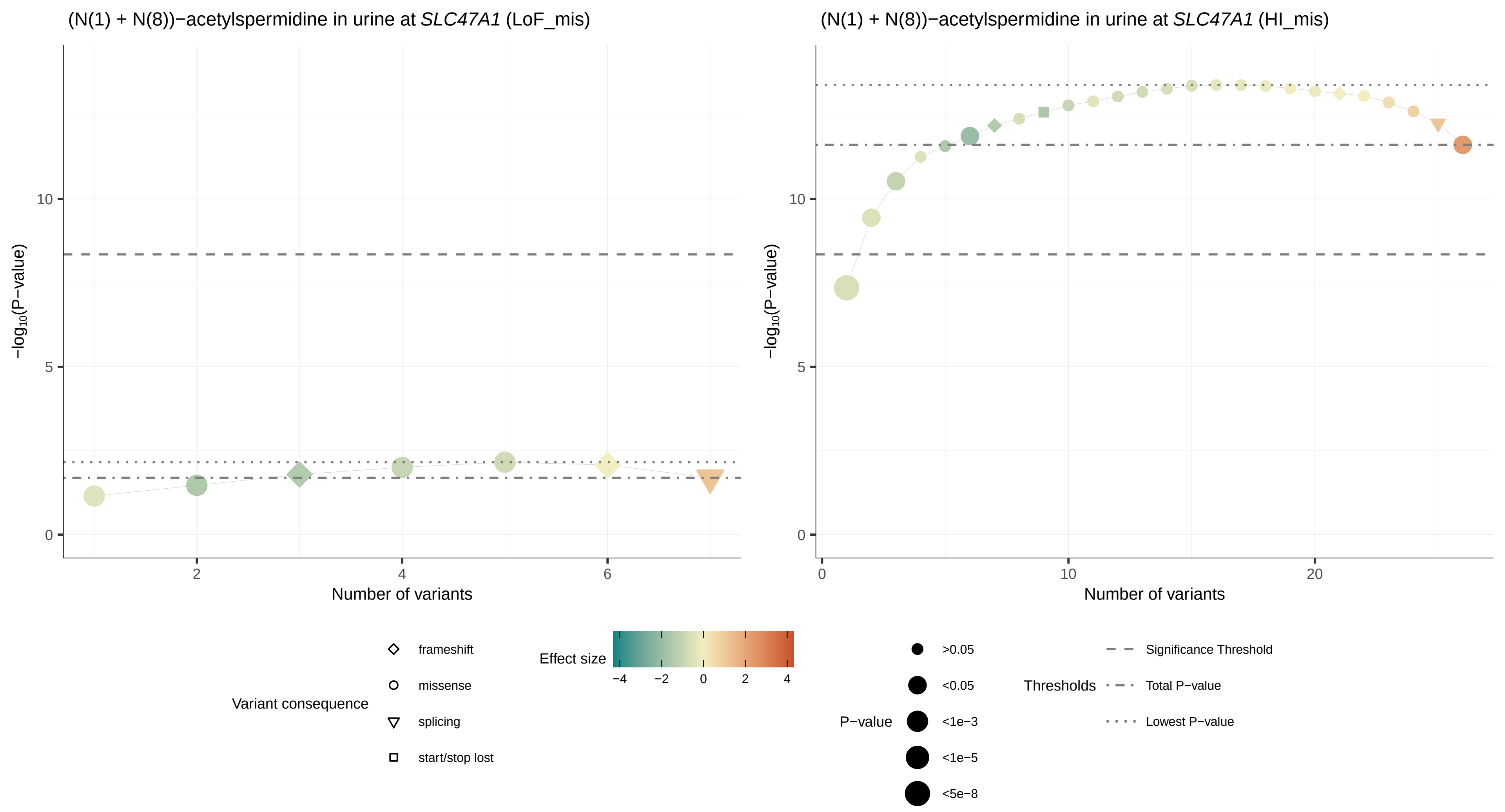

(N(1) + N(8))−acetylspermidine in urine at *SLC47A1* (LoF_mis)
(N(1) + N(8))−acetylspermidine in urine at *SLC47A1* (HI_mis)
−log10(P−value)
Number of variants
0
5
10
2
4
6
20
Variant consequence
frameshift
missense
splicing
start/stop lost
Effect size
−4
−2
0
2
4
P−value
>0.05
<0.05
<1e−3
<1e−5
<5e−8
Thresholds
Significance Threshold
Total P−value
Lowest P−value

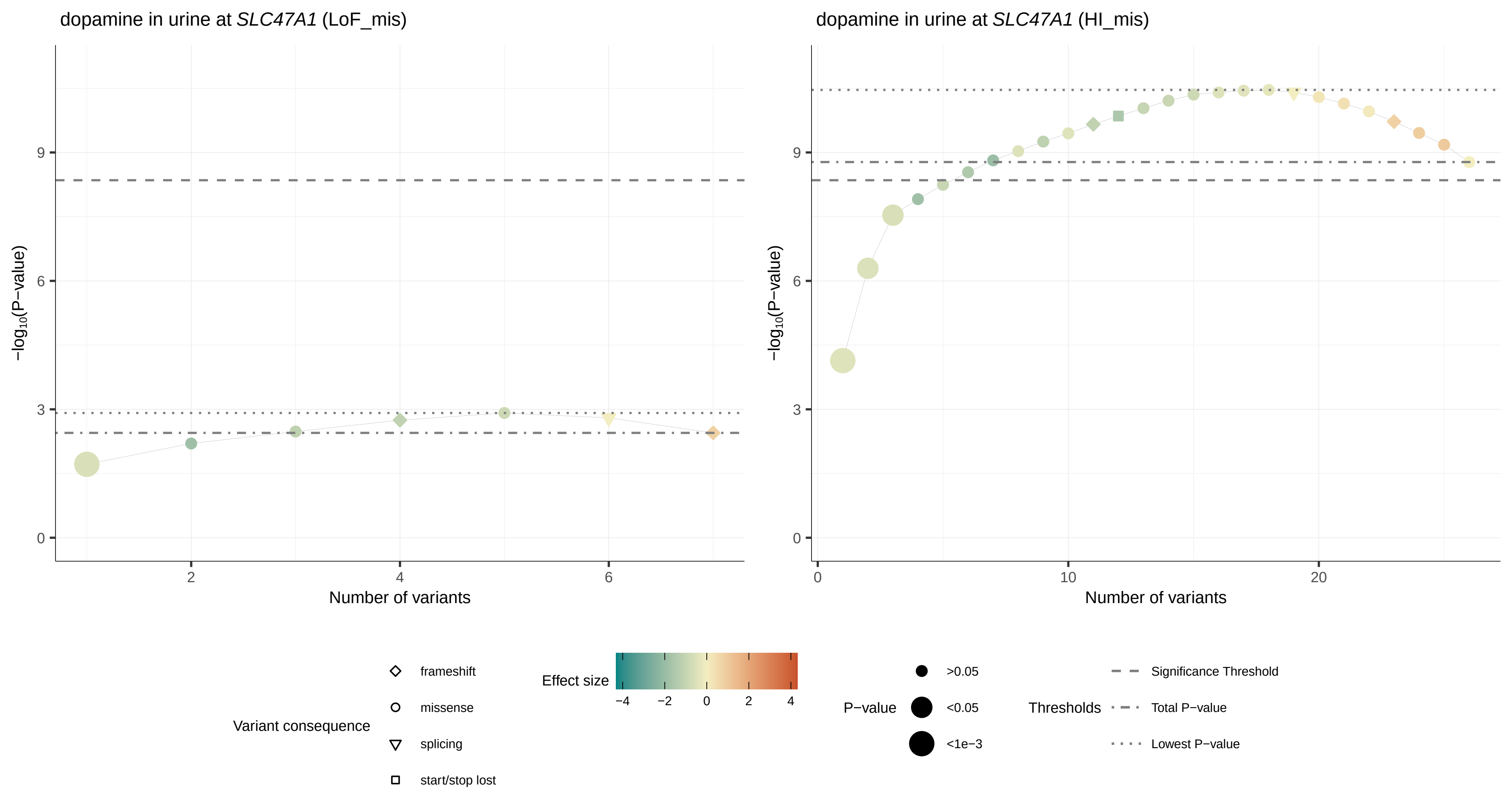

dopamine in urine at SLC47A1 (LoF_mis)
dopamine in urine at SLC47A1 (HI_mis)
−log10(P−value)
Number of variants
Variant consequence
frameshift
missense
splicing
start/stop lost
Effect size
−4
−2
0
2
4
P−value
>0.05
<0.05
<1e−3
Thresholds
Significance Threshold
Total P−value
Lowest P−value

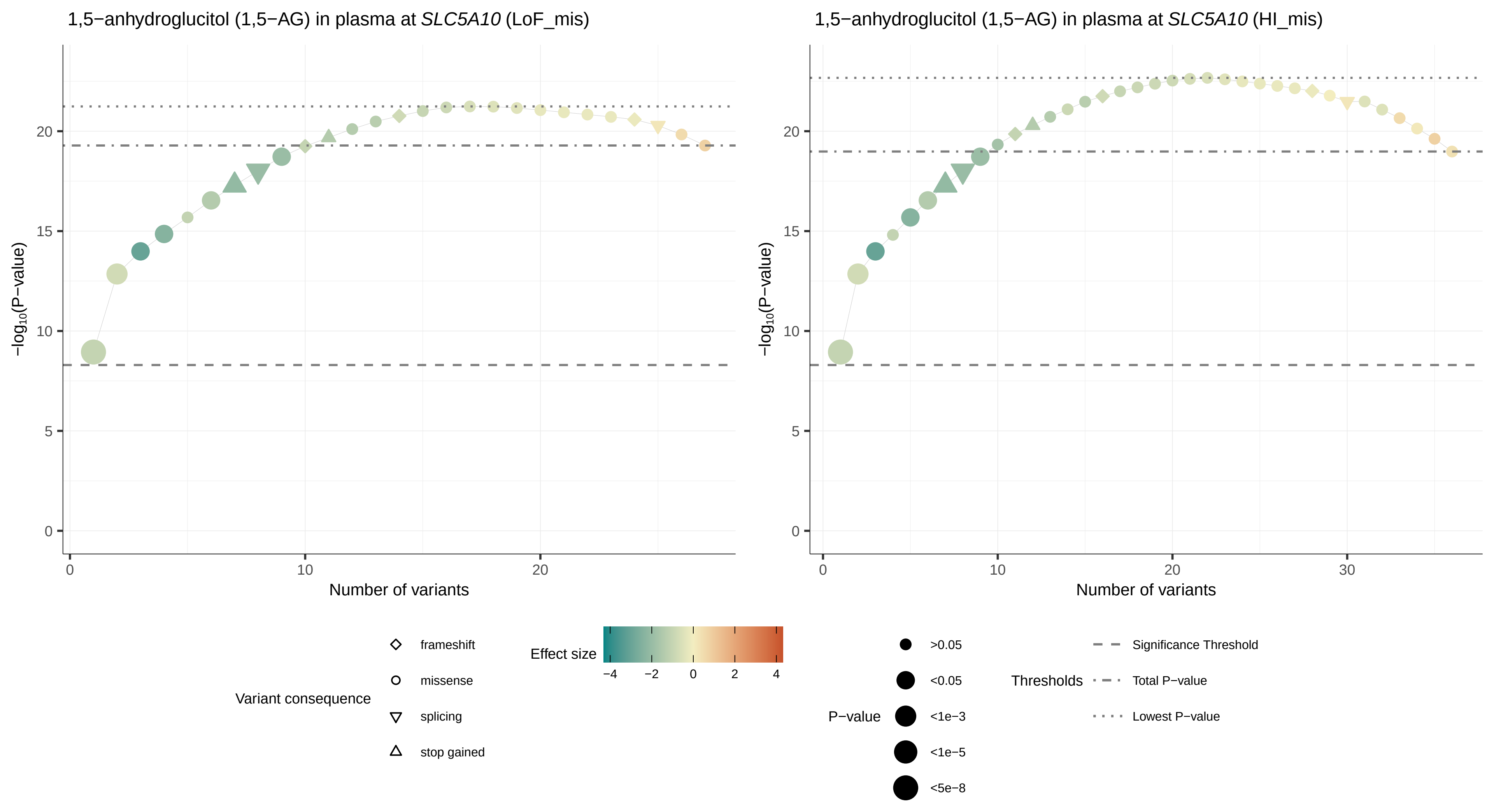
1,5−anhydroglucitol (1,5−AG) in plasma at SLC5A10 (LoF_mis)
1,5−anhydroglucitol (1,5−AG) in plasma at SLC5A10 (HI_mis)
−log10(P−value)
Number of variants
Variant consequence
frameshift
missense
splicing
stop gained
Effect size
P−value
>0.05
<0.05
<1e−3
<1e−5
<5e−8
Thresholds
Significance Threshold
Total P−value
Lowest P−value

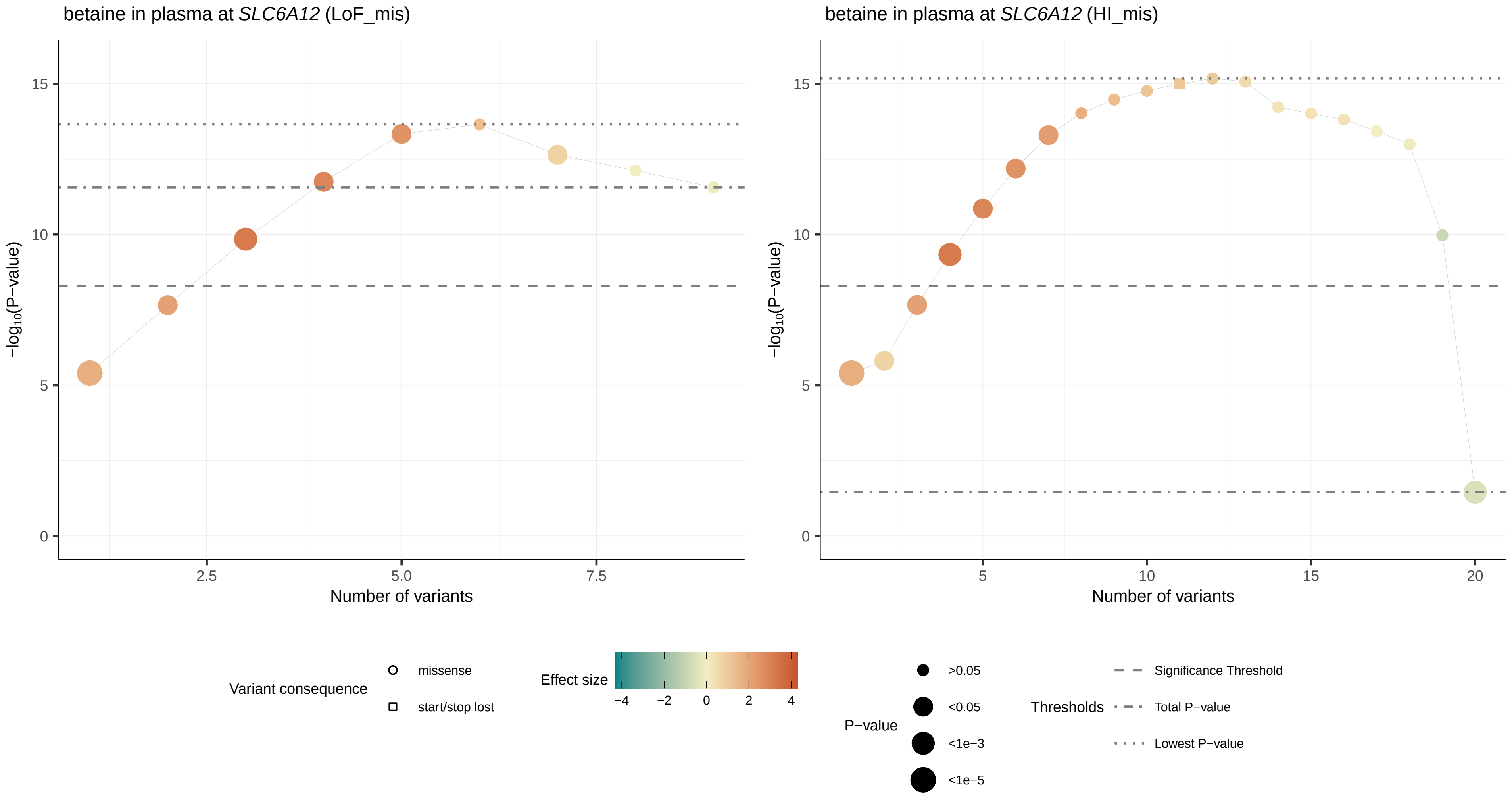

betaine in plasma at SLC6A12 (LoF_mis)
betaine in plasma at SLC6A12 (HI_mis)
−log10(P−value)
Number of variants
Variant consequence
missense
start/stop lost
Effect size
P−value
>0.05
<0.05
<1e−3
<1e−5
Thresholds
Significance Threshold
Total P−value
Lowest P−value

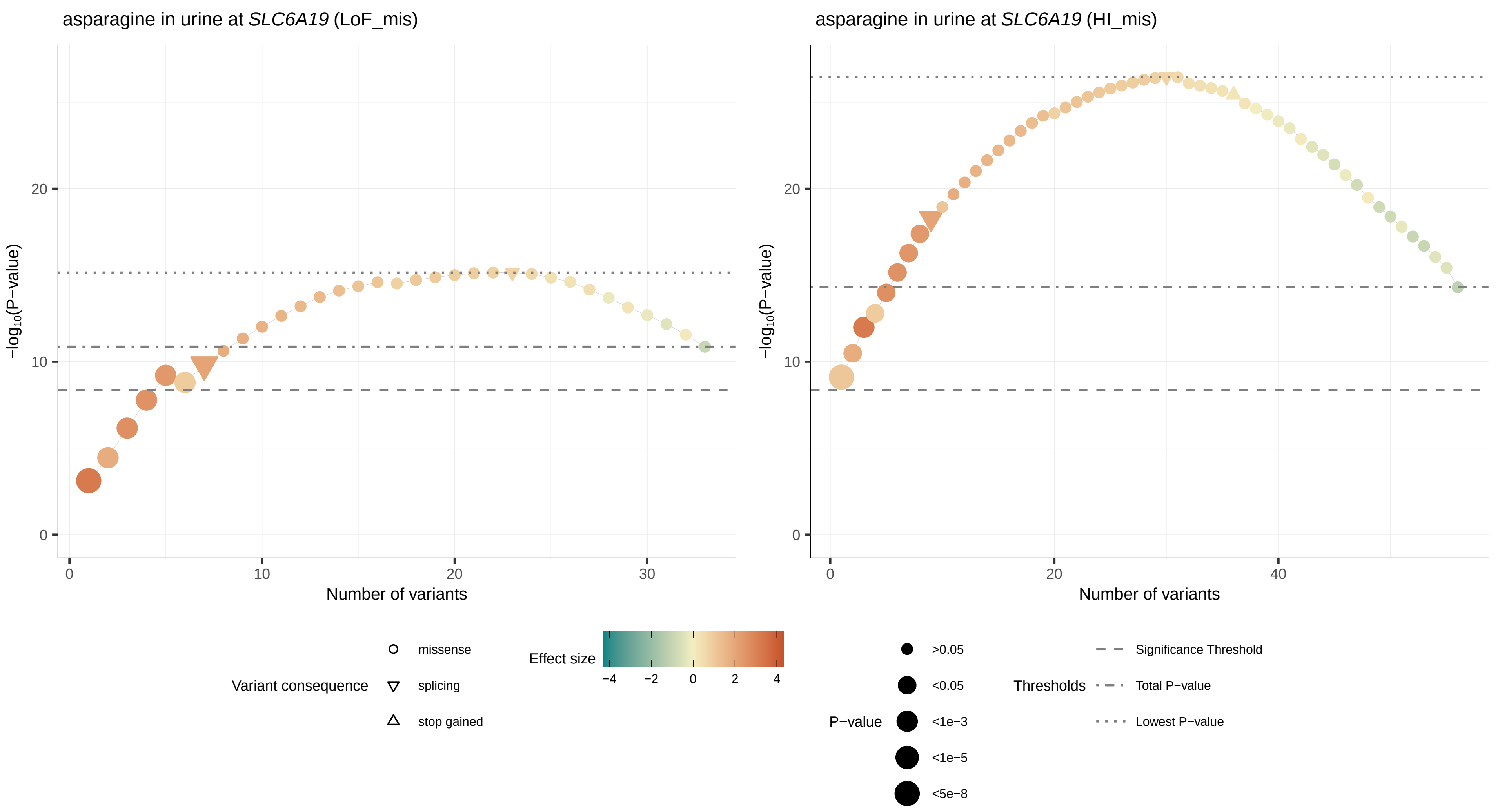

asparagine in urine at SLC6A19 (LoF_mis)
asparagine in urine at SLC6A19 (HI_mis)
−log10(P−value)
Number of variants
0
10
20
30
40
Variant consequence
missense
splicing
stop gained
Effect size
−4
−2
0
2
4
P−value
>0.05
<0.05
<1e−3
<1e−5
<5e−8
Thresholds
Significance Threshold
Total P−value
Lowest P−value

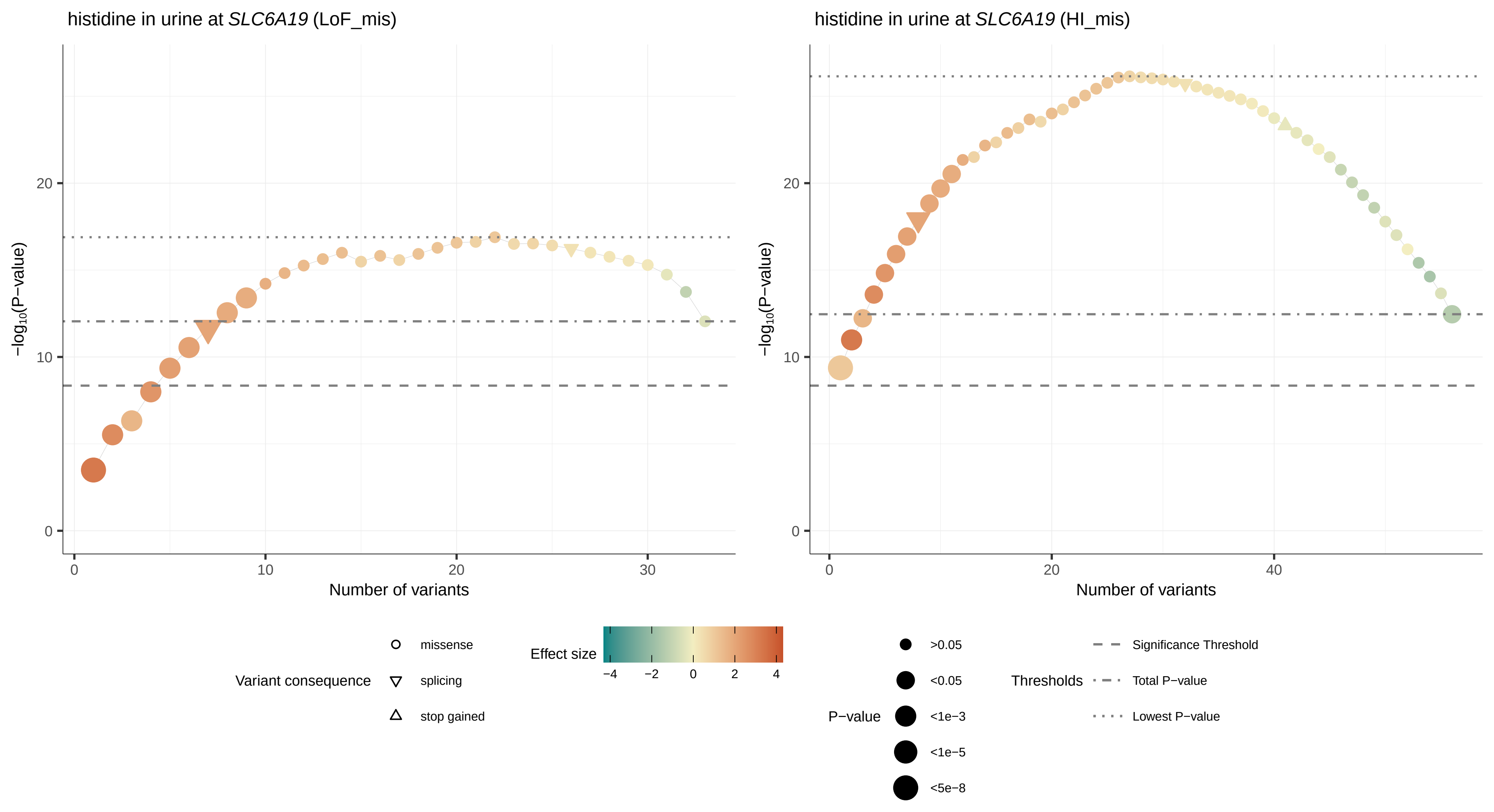

histidine in urine at SLC6A19 (LoF_mis)
histidine in urine at SLC6A19 (HI_mis)
−log10(P−value)
Number of variants
0
10
20
30
40
Variant consequence
missense
splicing
stop gained
Effect size
−4
−2
0
2
4
P−value
>0.05
<0.05
<1e−3
<1e−5
<5e−8
Thresholds
Significance Threshold
Total P−value
Lowest P−value

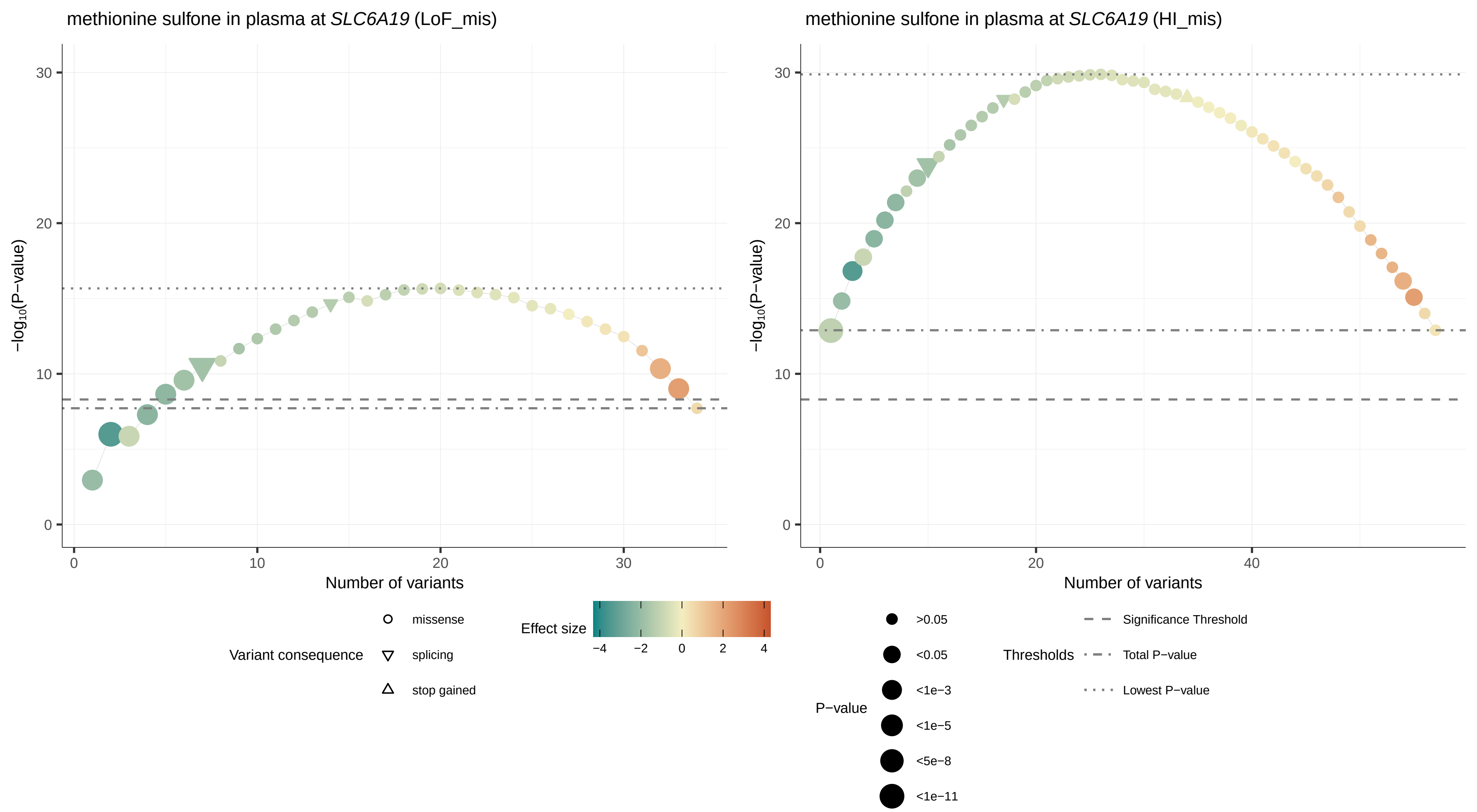

methionine sulfone in plasma at SLC6A19 (LoF_mis)
methionine sulfone in plasma at SLC6A19 (HI_mis)
−log10(P−value)
Number of variants
Variant consequence
missense
splicing
stop gained
Effect size
−4
−2
0
2
4
P−value
>0.05
<0.05
<1e−3
<1e−5
<5e−8
<1e−11
Thresholds
Significance Threshold
Total P−value
Lowest P−value

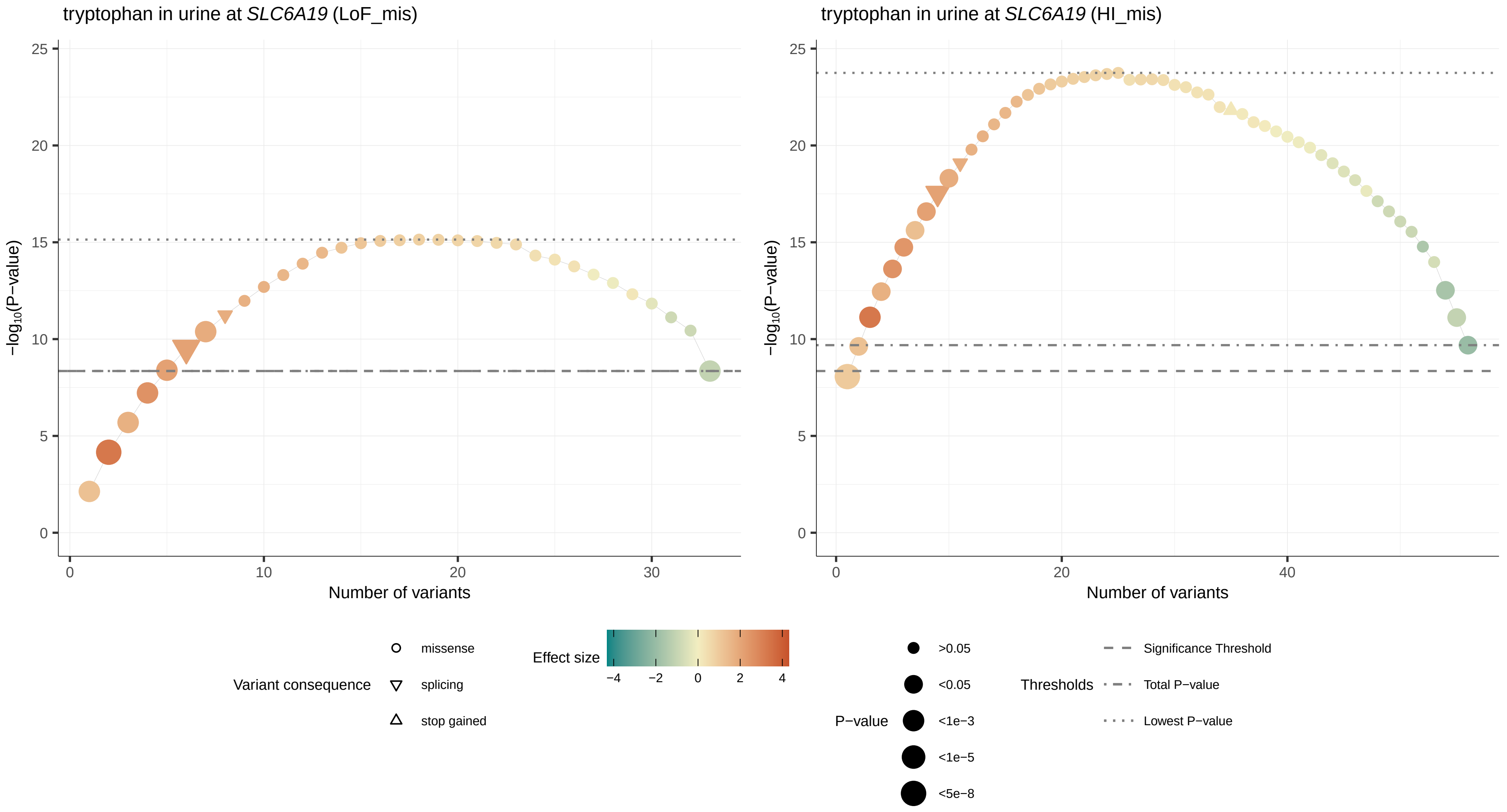

tryptophan in urine at SLC6A19 (LoF_mis)
tryptophan in urine at SLC6A19 (HI_mis)
−log10(P−value)
Number of variants
Variant consequence
missense
splicing
stop gained
Effect size
−4
−2
0
2
4
P−value
>0.05
<0.05
<1e−3
<1e−5
<5e−8
Thresholds
Significance Threshold
Total P−value
Lowest P−value

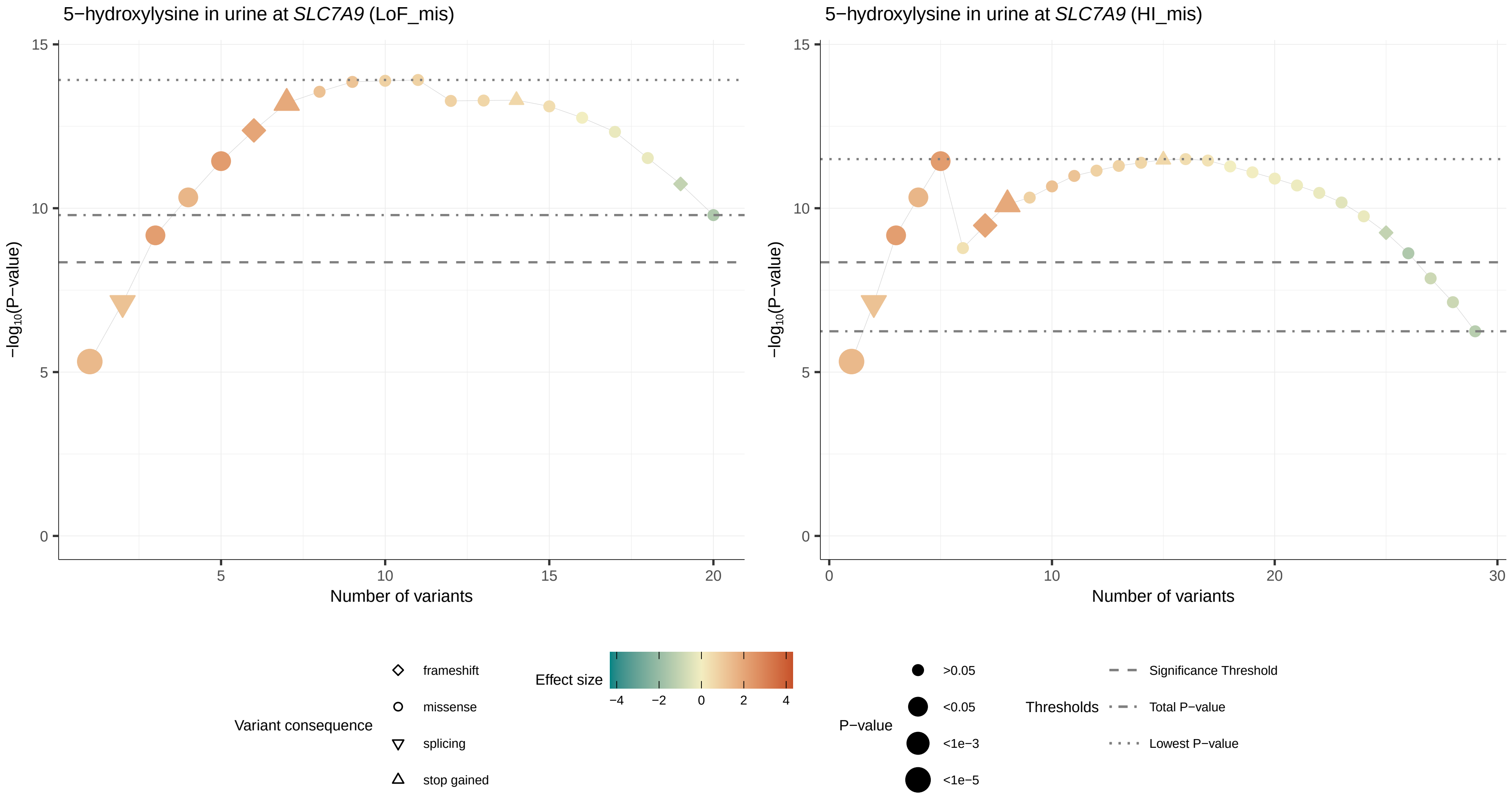

5−hydroxylysine in urine at *SLC7A9* (LoF_mis)
5−hydroxylysine in urine at *SLC7A9* (HI_mis)
$-\log_{10}$(P−value)
Number of variants
Variant consequence
frameshift
missense
splicing
stop gained
Effect size
−4
−2
0
2
4
P−value
>0.05
<0.05
<1e−3
<1e−5
Thresholds
Significance Threshold
Total P−value
Lowest P−value

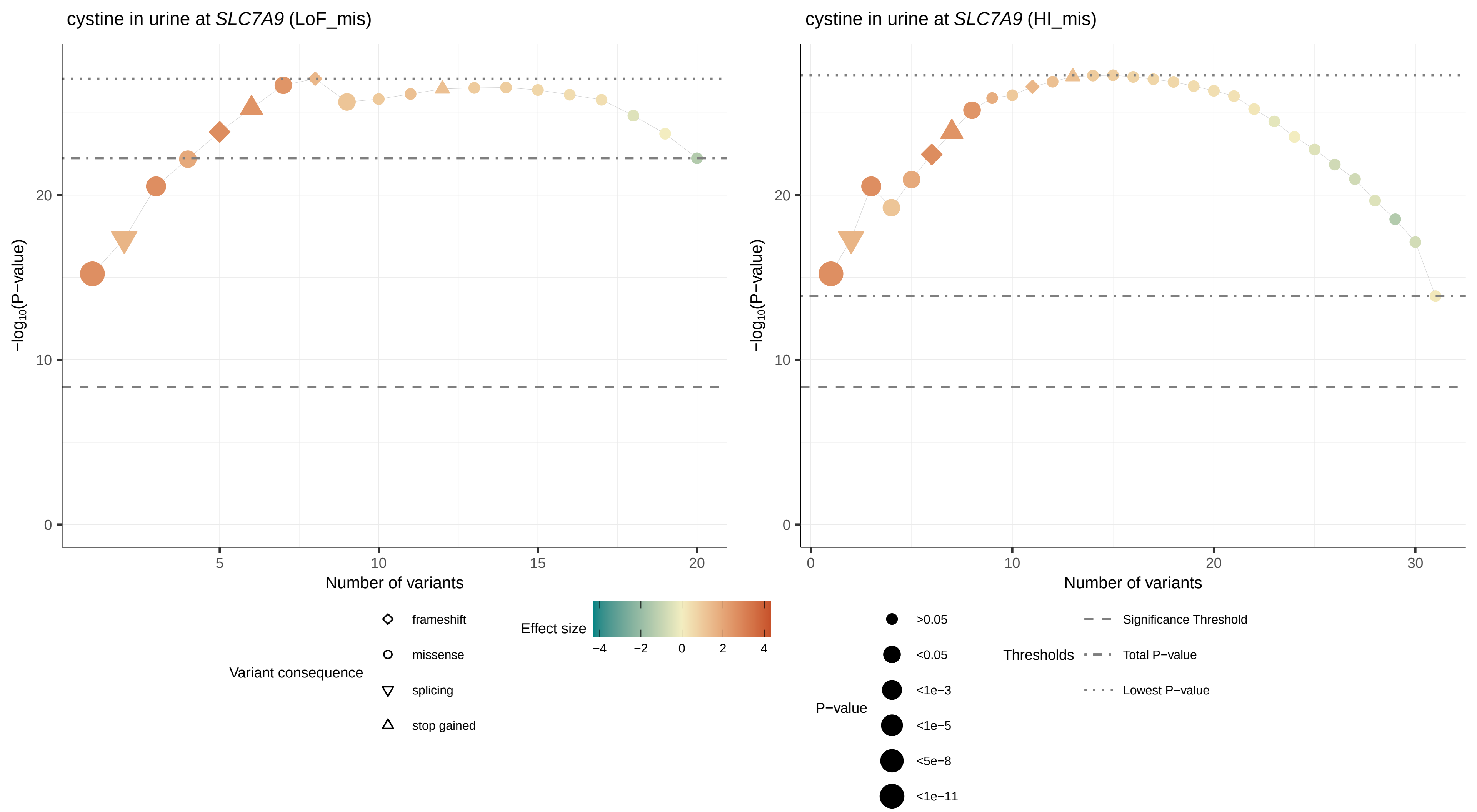

cystine in urine at SLC7A9 (LoF_mis)
cystine in urine at SLC7A9 (HI_mis)
−log10(P−value)
Number of variants
0
10
20
5
15
30
Variant consequence
frameshift
missense
splicing
stop gained
Effect size
−4
−2
2
4
P−value
>0.05
<0.05
<1e−3
<1e−5
<5e−8
<1e−11
Thresholds
Significance Threshold
Total P−value
Lowest P−value

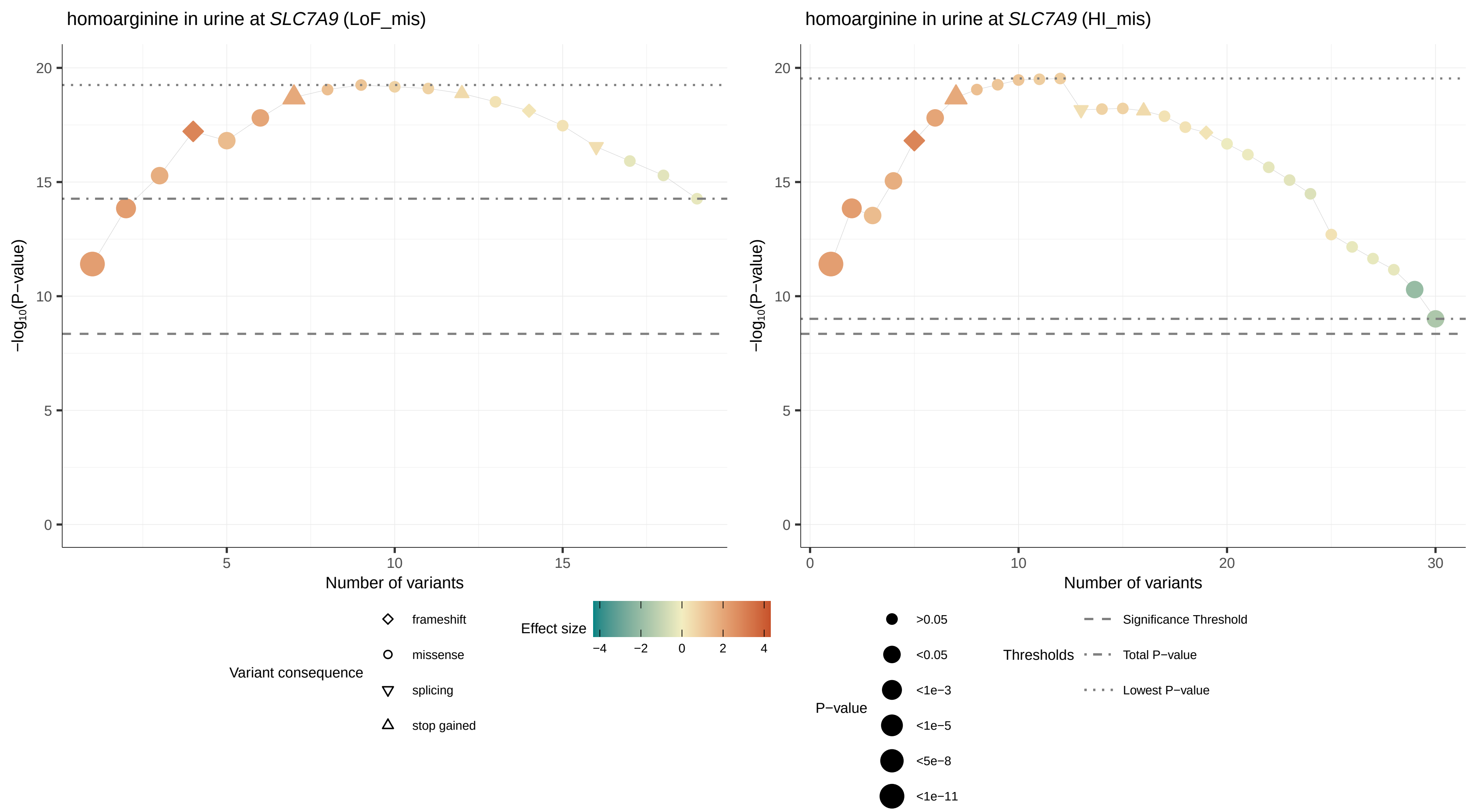
homoarginine in urine at *SLC7A9* (LoF_mis)
homoarginine in urine at *SLC7A9* (HI_mis)
−log10(P−value)
Number of variants
0
5
10
15
20
30
Variant consequence
frameshift
missense
splicing
stop gained
Effect size
−4
−2
2
4
P−value
>0.05
<0.05
<1e−3
<1e−5
<5e−8
<1e−11
Thresholds
Significance Threshold
Total P−value
Lowest P−value

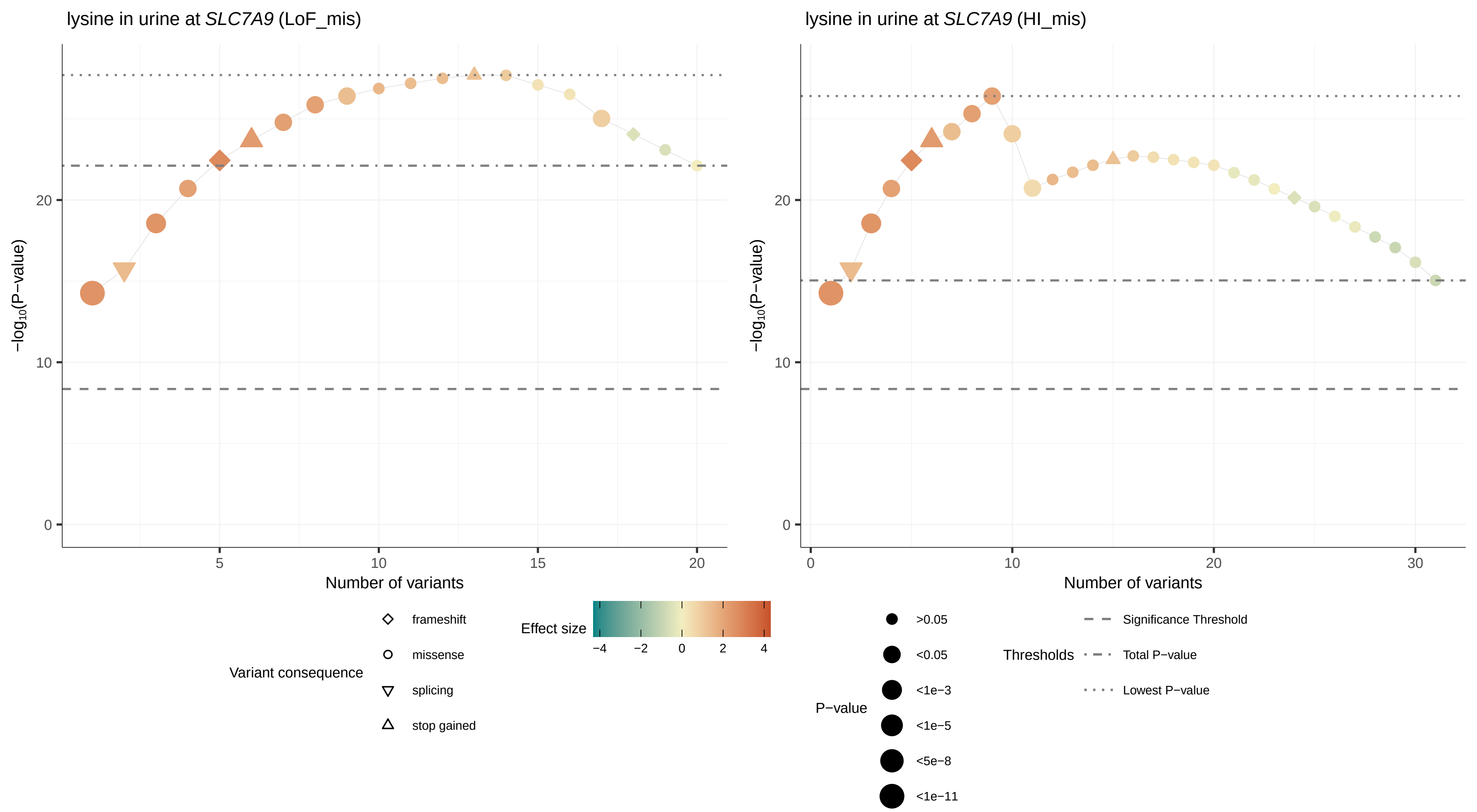

lysine in urine at SLC7A9 (LoF_mis)
lysine in urine at SLC7A9 (HI_mis)
−log10(P−value)
Number of variants
Variant consequence
frameshift
missense
splicing
stop gained
Effect size
−4 −2 0 2 4
P−value
>0.05
<0.05
<1e−3
<1e−5
<5e−8
<1e−11
Thresholds
Significance Threshold
Total P−value
Lowest P−value

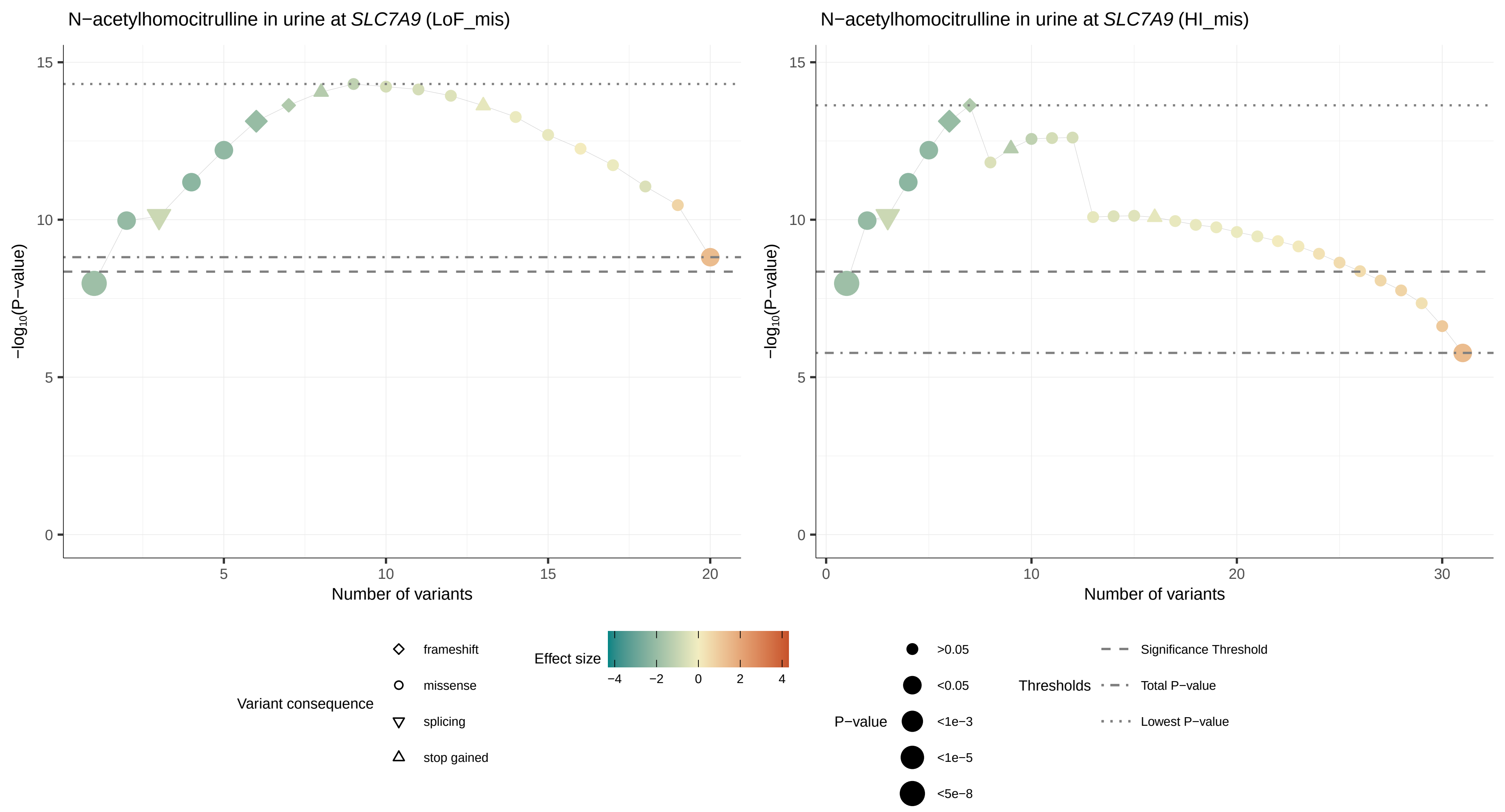
N−acetylhomocitrulline in urine at *SLC7A9* (LoF_mis)
N−acetylhomocitrulline in urine at *SLC7A9* (HI_mis)
−log10(P−value)
Number of variants
Variant consequence
frameshift
missense
splicing
stop gained
Effect size
P−value
>0.05
<0.05
<1e−3
<1e−5
<5e−8
Thresholds
Significance Threshold
Total P−value
Lowest P−value

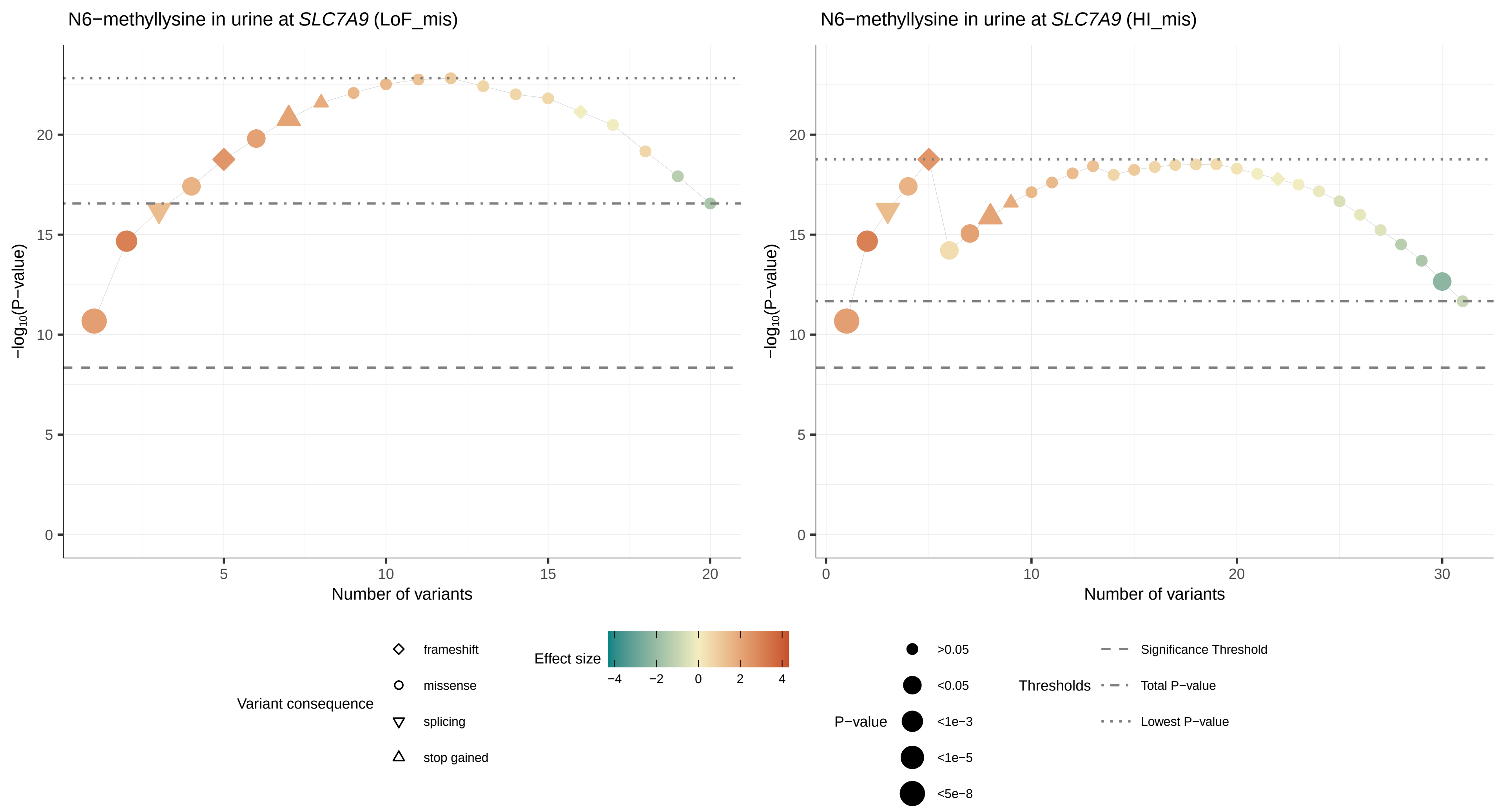

N6−methyllysine in urine at SLC7A9 (LoF_mis)
N6−methyllysine in urine at SLC7A9 (HI_mis)
−log10(P−value)
Number of variants
0
5
10
15
20
Variant consequence
frameshift
missense
splicing
stop gained
Effect size
−4
−2
0
2
4
P−value
>0.05
<0.05
<1e−3
<1e−5
<5e−8
Thresholds
Significance Threshold
Total P−value
Lowest P−value

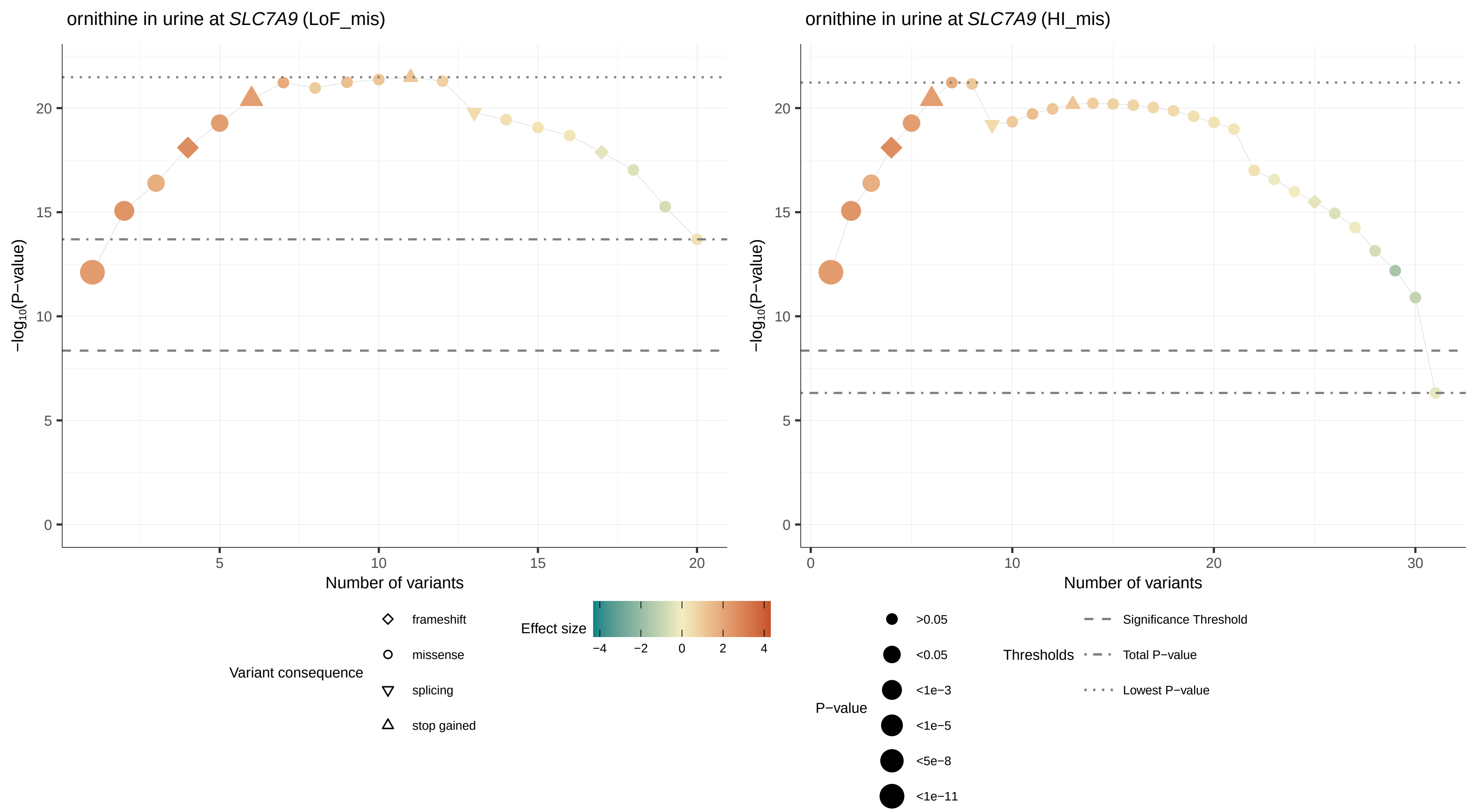
ornithine in urine at SLC7A9 (LoF_mis)
ornithine in urine at SLC7A9 (HI_mis)
−log10(P−value)
Number of variants
0
5
10
15
20
30
Variant consequence
frameshift
missense
splicing
stop gained
Effect size
−4
−2
0
2
4
P−value
>0.05
<0.05
<1e−3
<1e−5
<5e−8
<1e−11
Thresholds
Significance Threshold
Total P−value
Lowest P−value

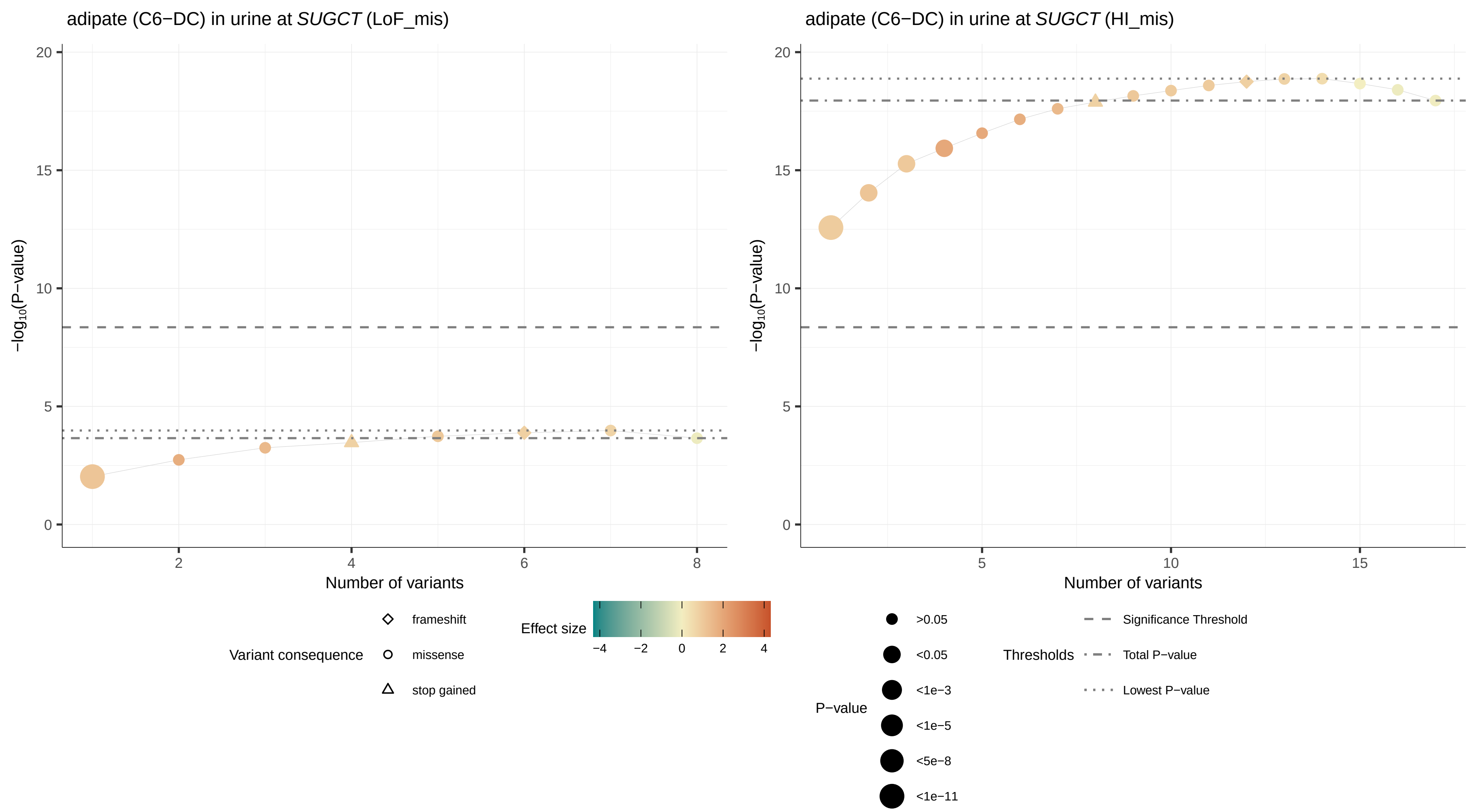
adipate (C6−DC) in urine at SUGCT (LoF_mis)
adipate (C6−DC) in urine at SUGCT (HI_mis)
−log10(P−value)
Number of variants
Variant consequence
frameshift
missense
stop gained
Effect size
P−value
>0.05
<0.05
<1e−3
<1e−5
<5e−8
<1e−11
Thresholds
Significance Threshold
Total P−value
Lowest P−value

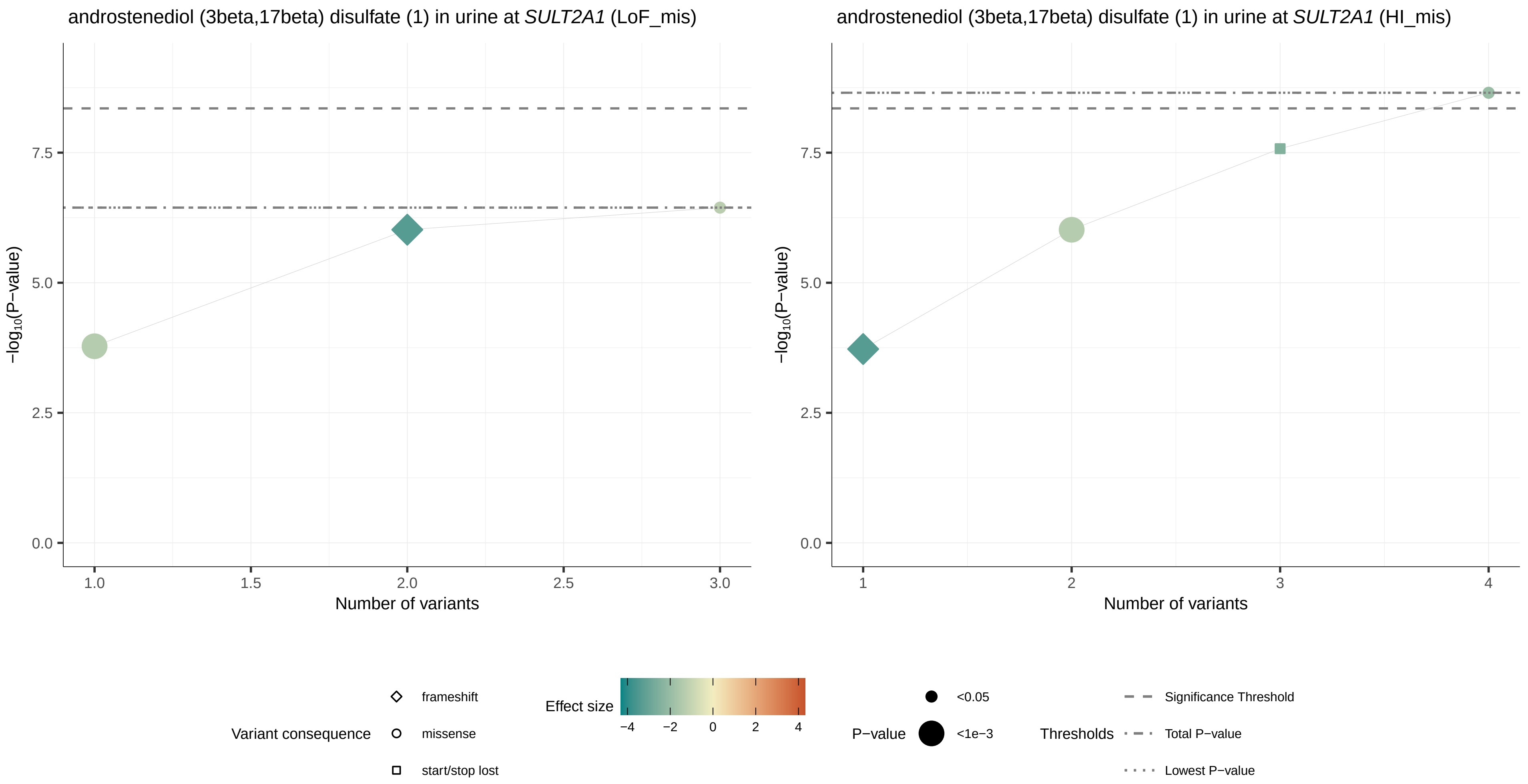

androstenediol (3beta,17beta) disulfate (1) in urine at SULT2A1 (LoF_mis)
androstenediol (3beta,17beta) disulfate (1) in urine at SULT2A1 (HI_mis)
−log10(P−value)
0.0
2.5
5.0
7.5
1.0
1.5
2.0
2.5
3.0
1
2
3
4
Number of variants
Variant consequence
frameshift
missense
start/stop lost
Effect size
−4
−2
0
2
4
P−value
<0.05
<1e−3
Thresholds
Significance Threshold
Total P−value
Lowest P−value

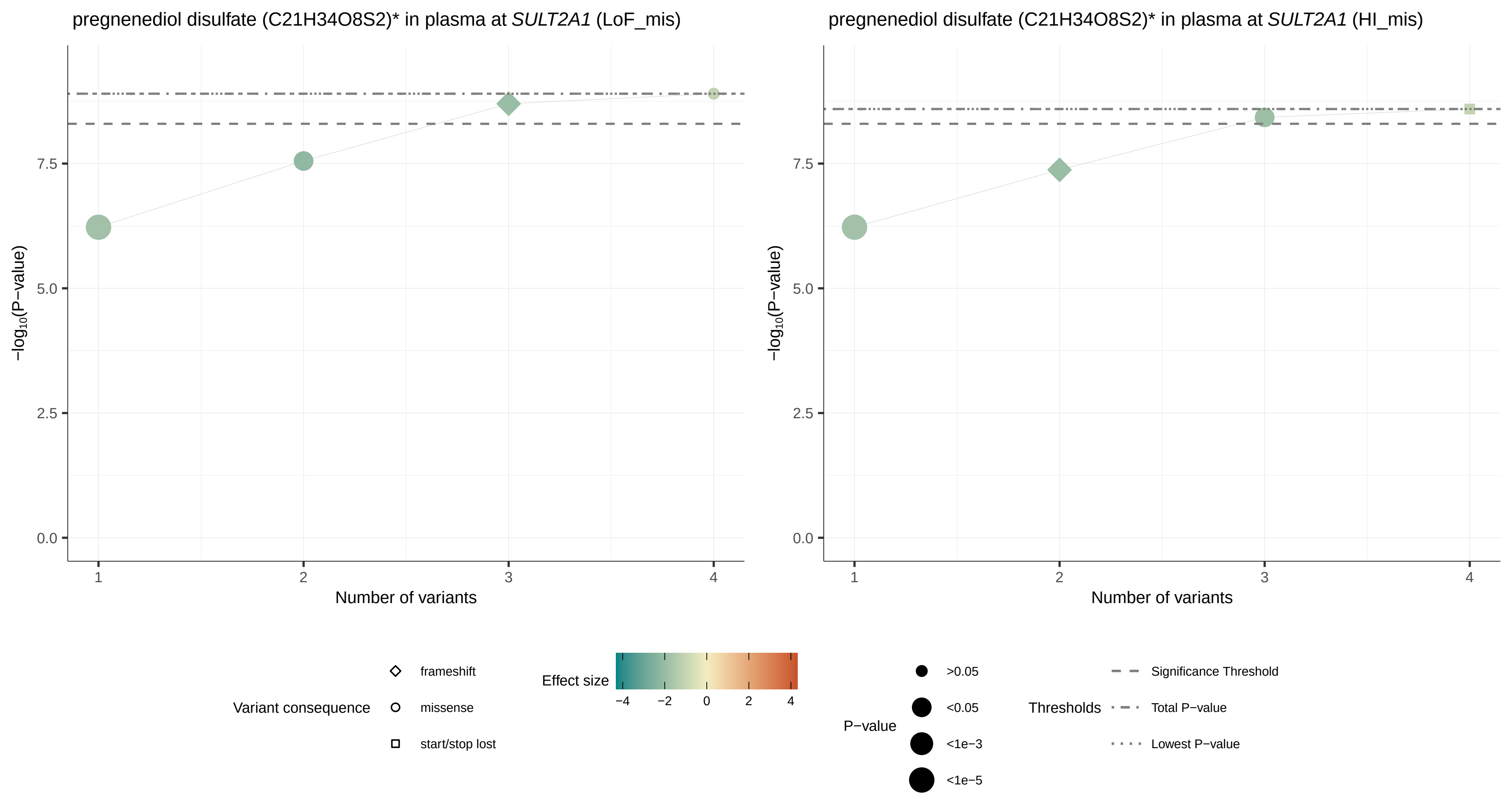

pregnenediol disulfate (C21H34O8S2)* in plasma at SULT2A1 (LoF_mis)
pregnenediol disulfate (C21H34O8S2)* in plasma at SULT2A1 (HI_mis)
−log10(P−value)
0.0
2.5
5.0
7.5
1
2
3
4
Number of variants
Variant consequence
frameshift
missense
start/stop lost
Effect size
−4
−2
0
2
4
P−value
>0.05
<0.05
<1e−3
<1e−5
Thresholds
Significance Threshold
Total P−value
Lowest P−value

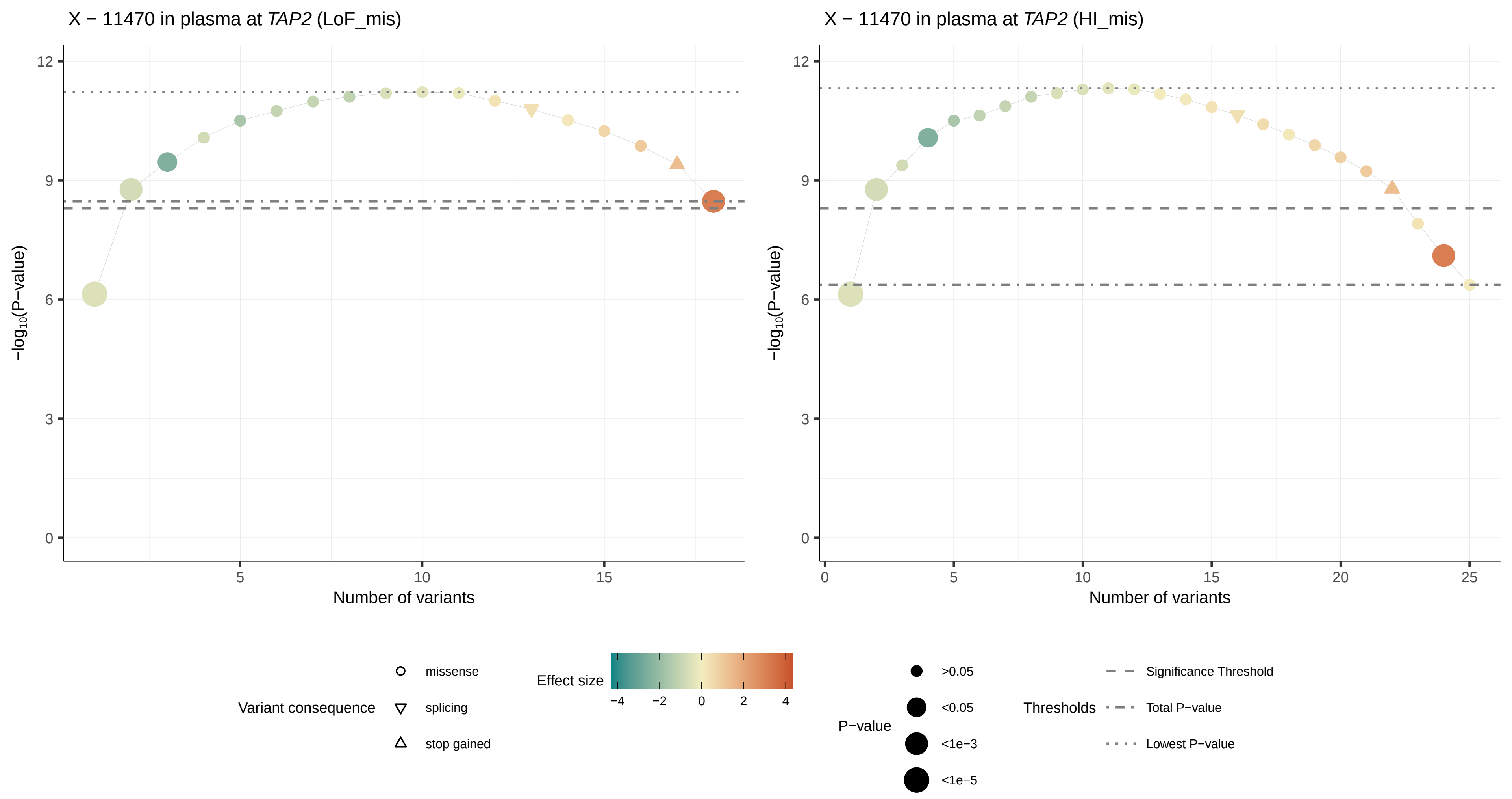
X − 11470 in plasma at *TAP2* (LoF_mis)
X − 11470 in plasma at *TAP2* (HI_mis)
−log10(P−value)
Number of variants
0
3
6
9
12
5
10
15
20
25
Variant consequence
missense
splicing
stop gained
Effect size
−4
−2
0
2
4
P−value
>0.05
<0.05
<1e−3
<1e−5
Thresholds
Significance Threshold
Total P−value
Lowest P−value

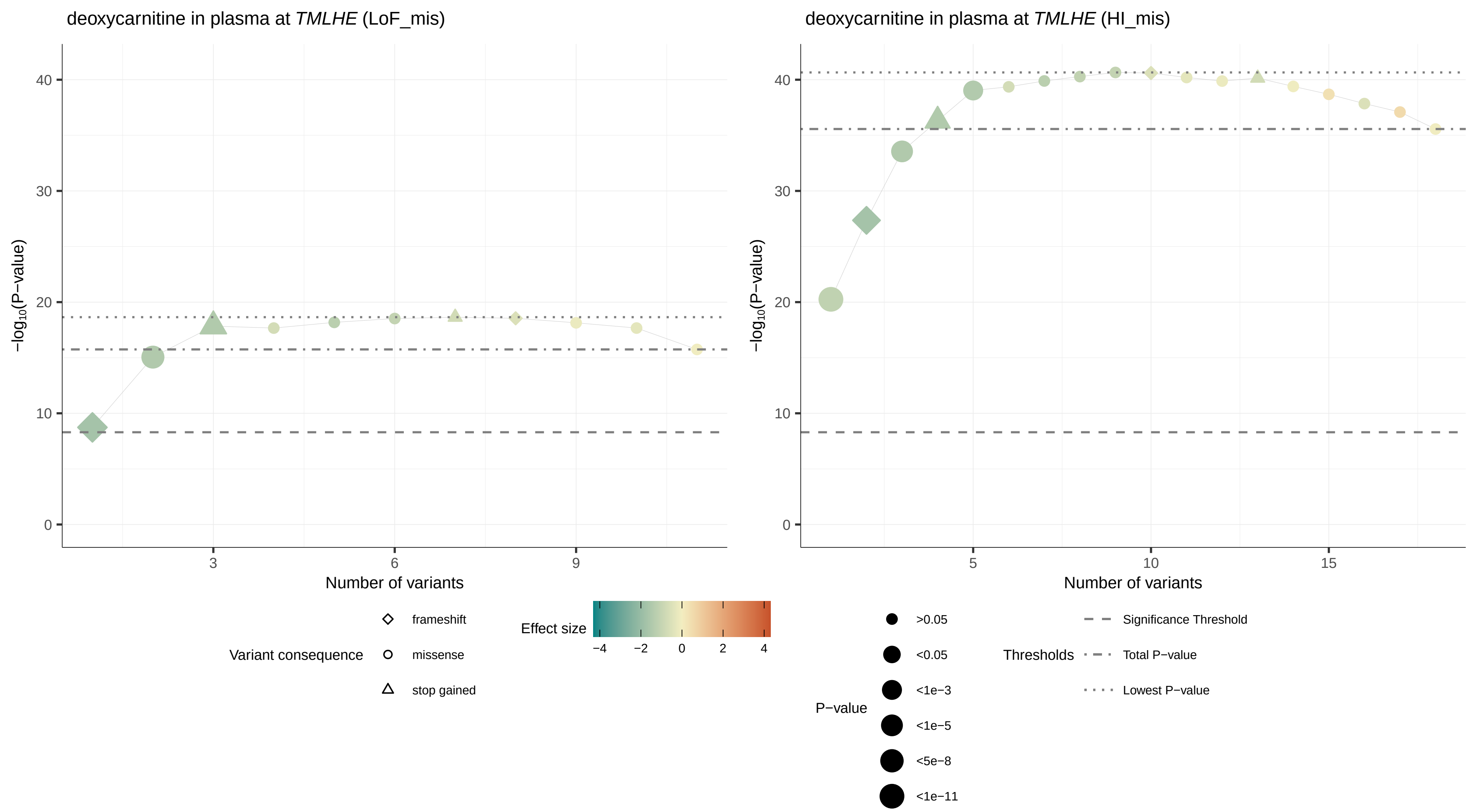

deoxycarnitine in plasma at *TMLHE* (LoF_mis)
deoxycarnitine in plasma at *TMLHE* (HI_mis)
−log10(P−value)
Number of variants
Variant consequence
frameshift
missense
stop gained
Effect size
−4
−2
0
2
4
P−value
>0.05
<0.05
<1e−3
<1e−5
<5e−8
<1e−11
Thresholds
Significance Threshold
Total P−value
Lowest P−value

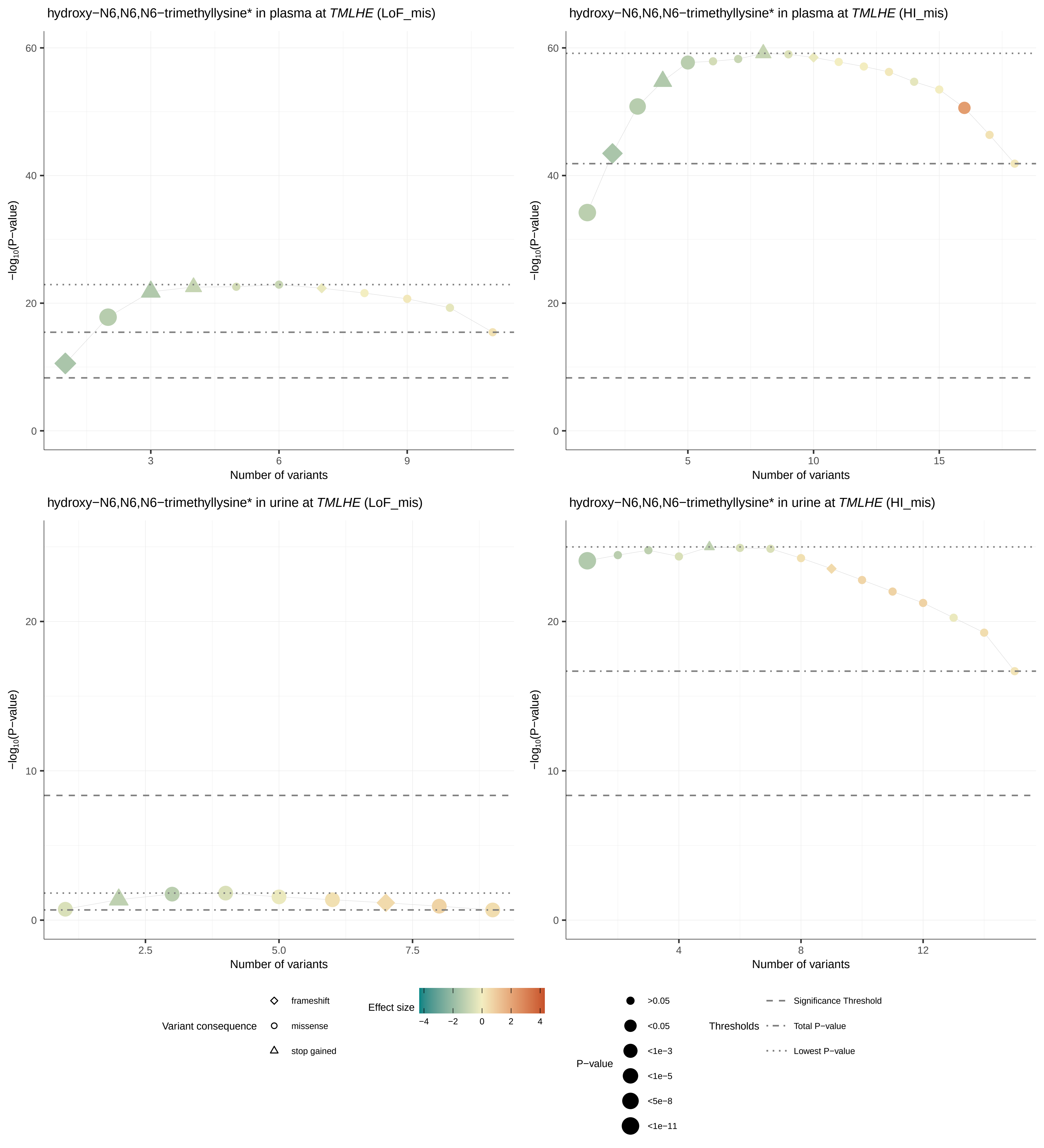

hydroxy−N6,N6,N6−trimethyllysine* in plasma at TMLHE (LoF_mis)
hydroxy−N6,N6,N6−trimethyllysine* in plasma at TMLHE (HI_mis)
hydroxy−N6,N6,N6−trimethyllysine* in urine at TMLHE (LoF_mis)
hydroxy−N6,N6,N6−trimethyllysine* in urine at TMLHE (HI_mis)
−log10(P−value)
Number of variants
Variant consequence
frameshift
missense
stop gained
Effect size
−4 −2 0 2 4
P−value
>0.05
<0.05
<1e−3
<1e−5
<5e−8
<1e−11
Thresholds
Significance Threshold
Total P−value
Lowest P−value

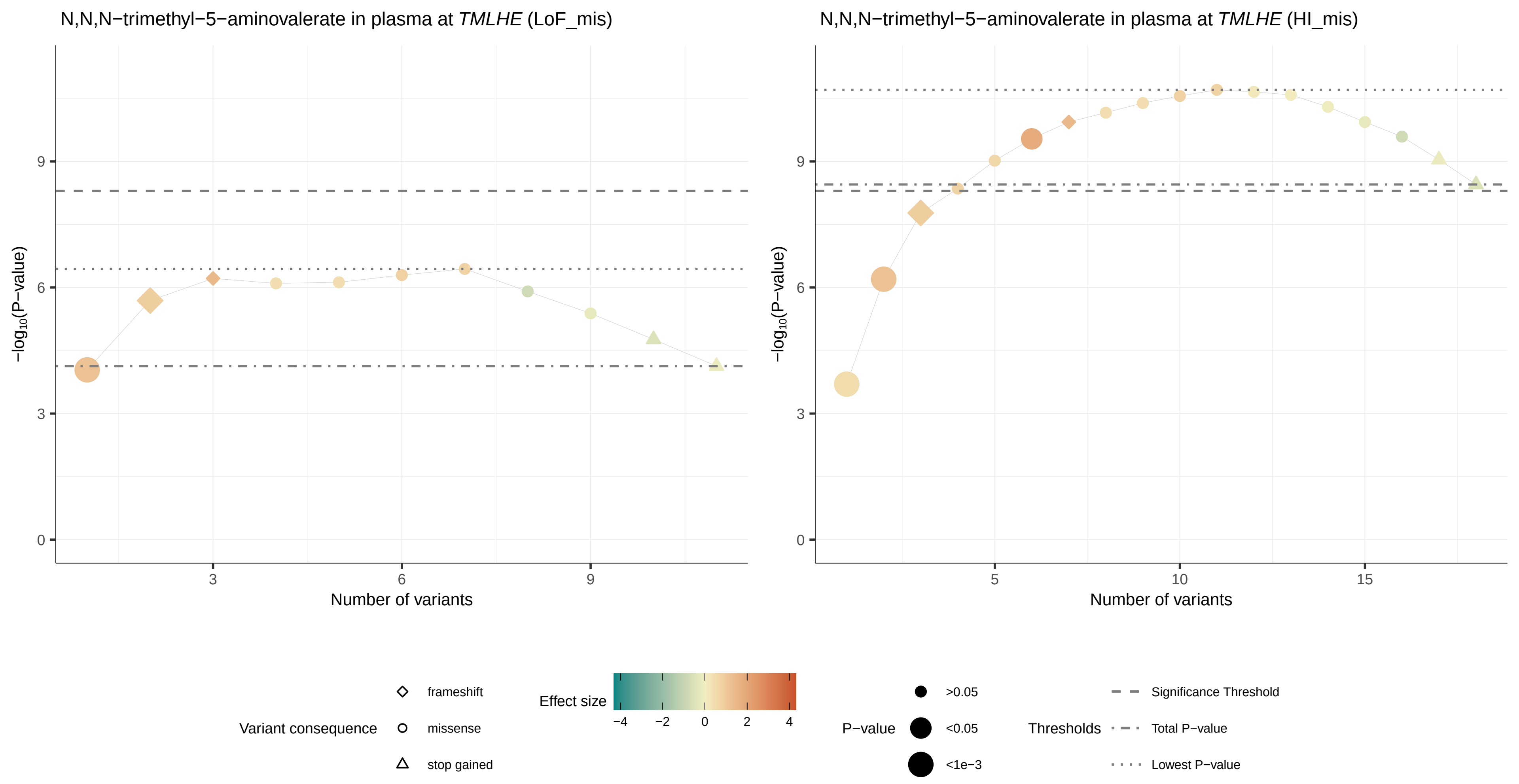
N,N,N−trimethyl−5−aminovalerate in plasma at *TMLHE* (LoF_mis)
N,N,N−trimethyl−5−aminovalerate in plasma at *TMLHE* (HI_mis)
−log10(P−value)
0
3
6
9
Number of variants
3
6
9
5
10
15
Variant consequence
frameshift
missense
stop gained
Effect size
−4
−2
0
2
4
P−value
>0.05
<0.05
<1e−3
Thresholds
Significance Threshold
Total P−value
Lowest P−value

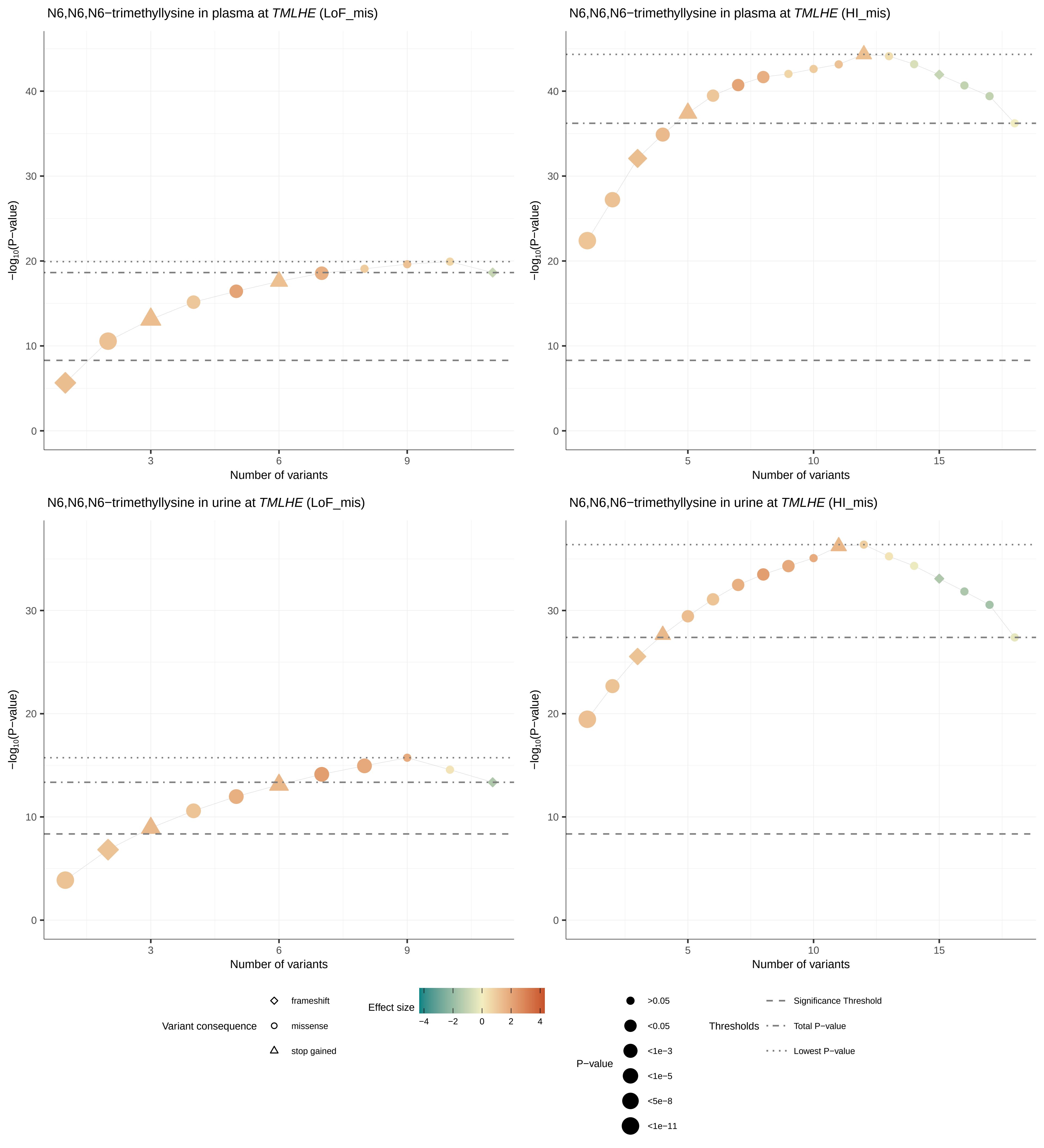

N6,N6,N6−trimethyllysine in plasma at TMLHE (LoF_mis)
N6,N6,N6−trimethyllysine in plasma at TMLHE (HI_mis)
N6,N6,N6−trimethyllysine in urine at TMLHE (LoF_mis)
N6,N6,N6−trimethyllysine in urine at TMLHE (HI_mis)
−log10(P−value)
Number of variants
Variant consequence
frameshift
missense
stop gained
Effect size
P−value
>0.05
<0.05
<1e−3
<1e−5
<5e−8
<1e−11
Thresholds
Significance Threshold
Total P−value
Lowest P−value

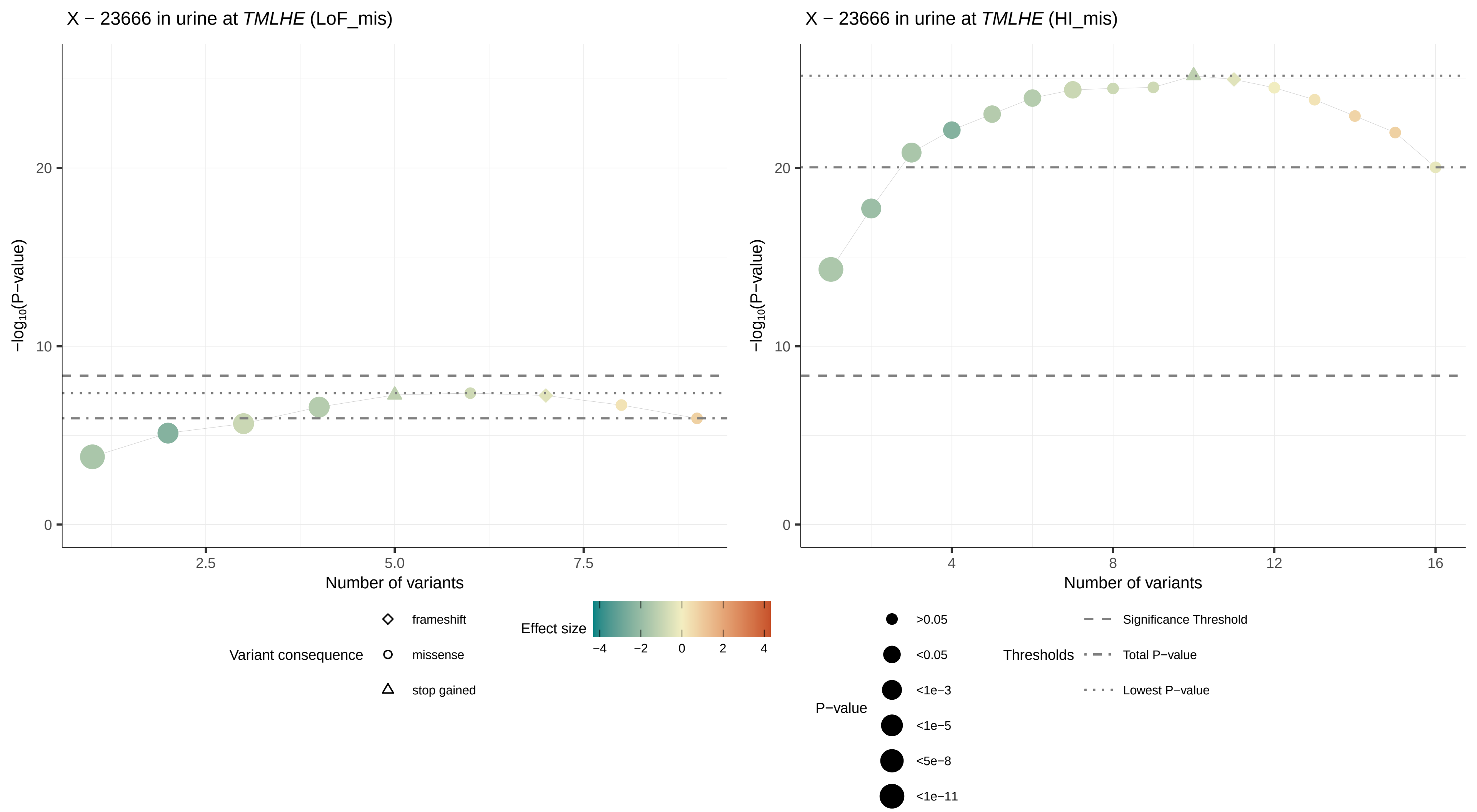

X − 23666 in urine at TMLHE (LoF_mis)
X − 23666 in urine at TMLHE (HI_mis)
−log10(P−value)
Number of variants
0
10
20
2.5
5.0
7.5
4
8
12
16
Variant consequence
frameshift
missense
stop gained
Effect size
−4
−2
0
2
4
P−value
>0.05
<0.05
<1e−3
<1e−5
<5e−8
<1e−11
Thresholds
Significance Threshold
Total P−value
Lowest P−value

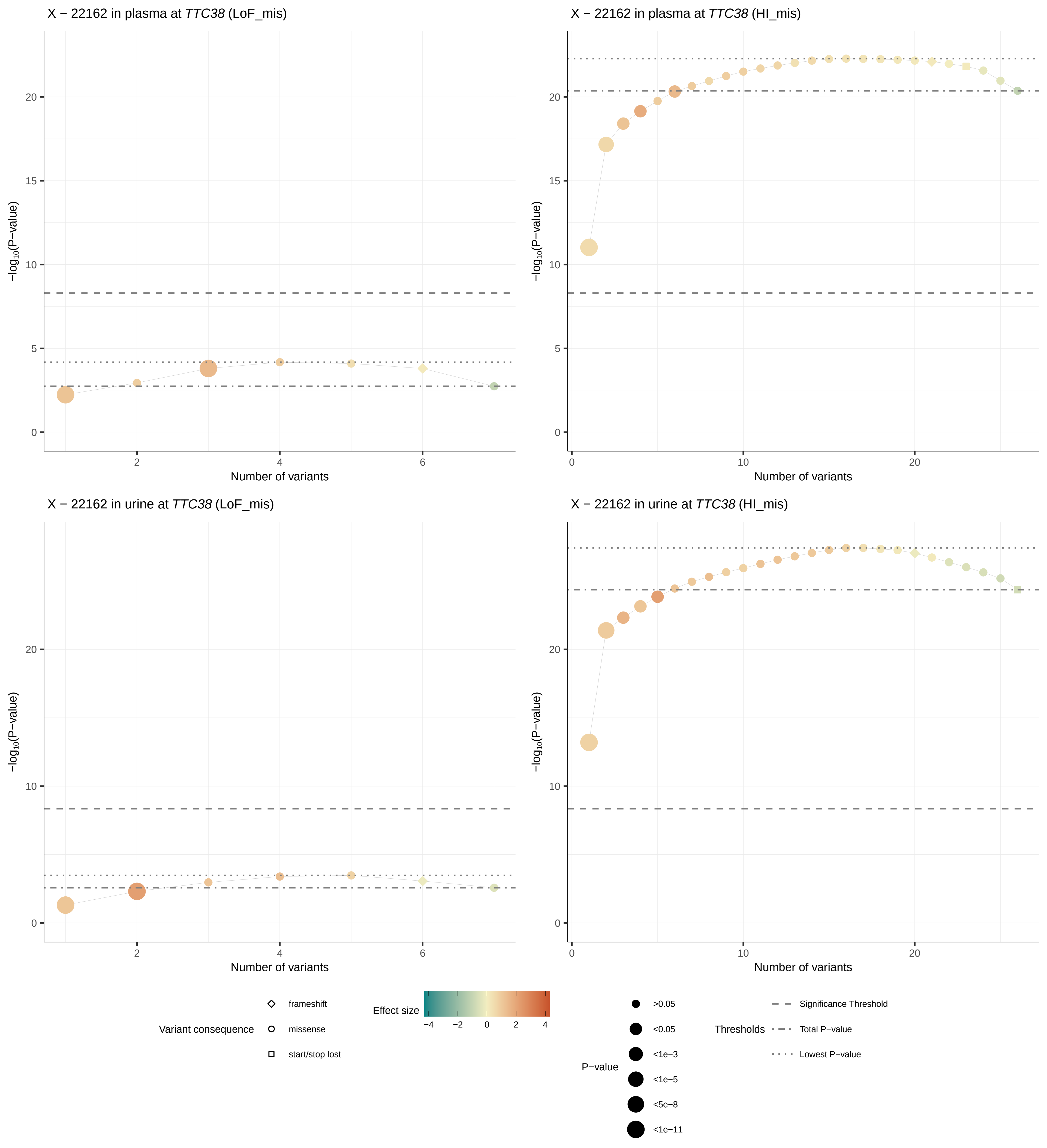
X − 22162 in plasma at TTC38 (LoF_mis)
X − 22162 in plasma at TTC38 (HI_mis)
X − 22162 in urine at TTC38 (LoF_mis)
X − 22162 in urine at TTC38 (HI_mis)
−log10(P−value)
Number of variants
Variant consequence
frameshift
missense
start/stop lost
Effect size
P−value
>0.05
<0.05
<1e−3
<1e−5
<5e−8
<1e−11
Thresholds
Significance Threshold
Total P−value
Lowest P−value

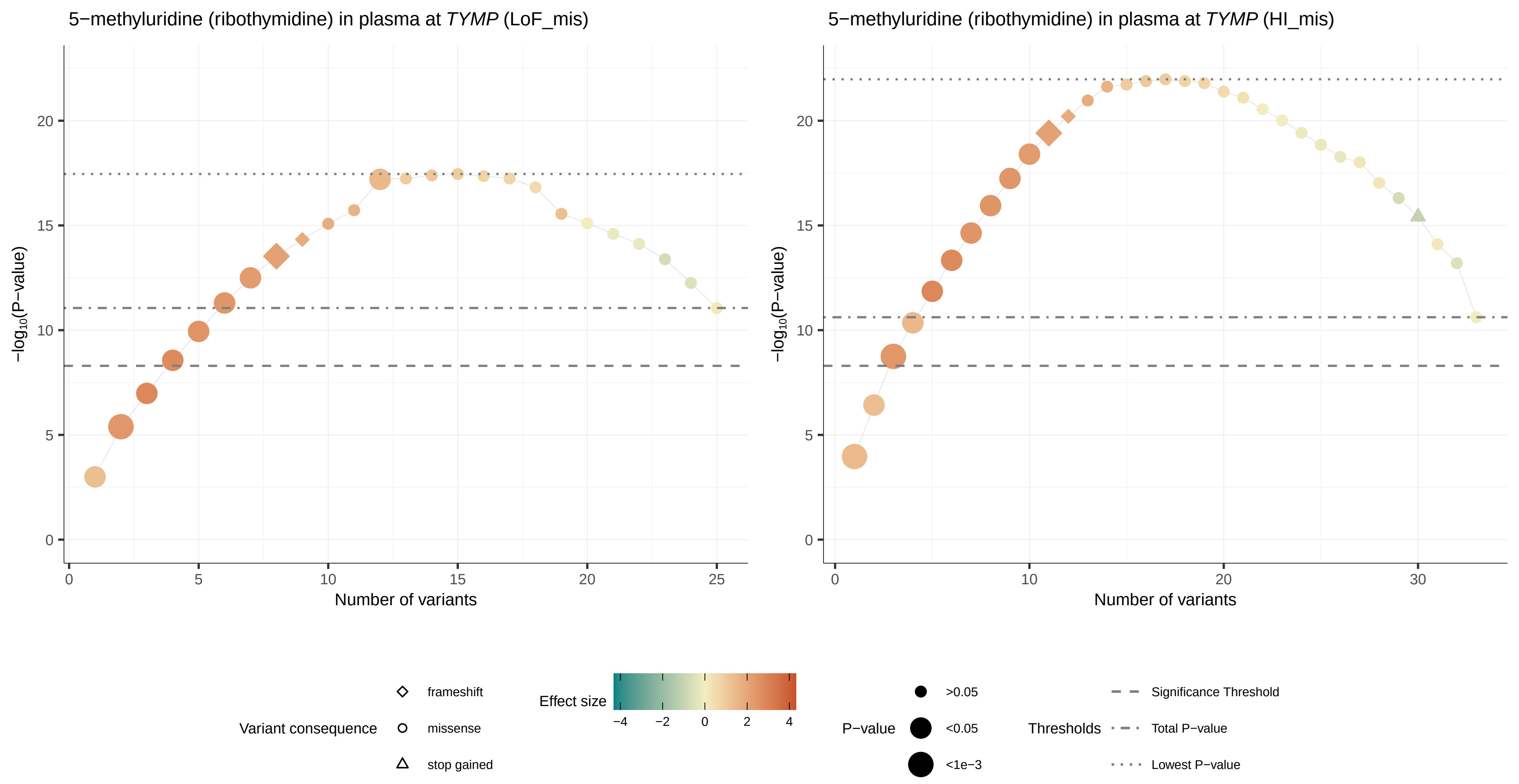

5−methyluridine (ribothymidine) in plasma at TYMP (LoF_mis)
5−methyluridine (ribothymidine) in plasma at TYMP (HI_mis)
−log10(P−value)
Number of variants
0
5
10
15
20
25
30
Variant consequence
frameshift
missense
stop gained
Effect size
−4
−2
0
2
4
P−value
>0.05
<0.05
<1e−3
Thresholds
Significance Threshold
Total P−value
Lowest P−value

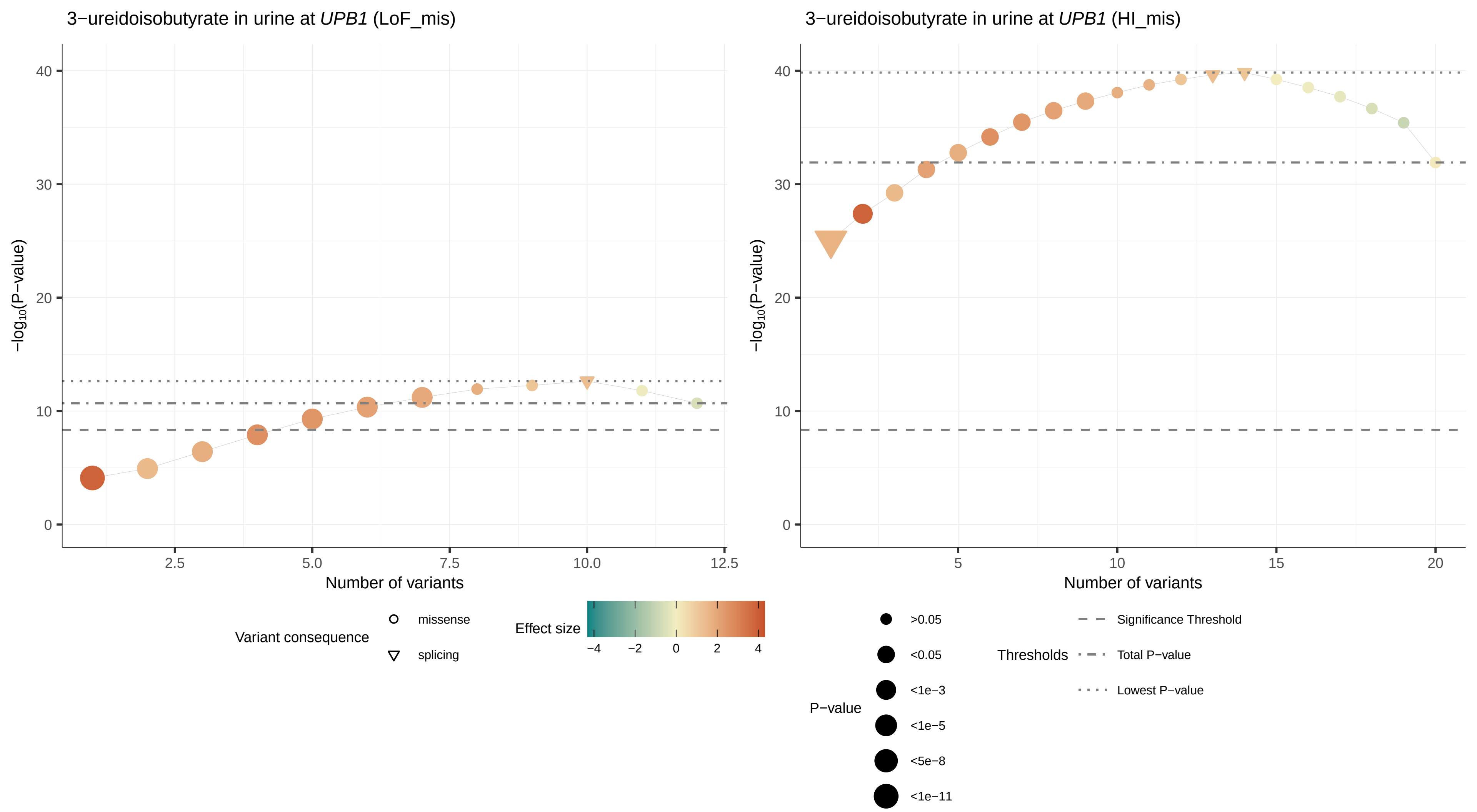
3−ureidoisobutyrate in urine at UPB1 (LoF_mis)
3−ureidoisobutyrate in urine at UPB1 (HI_mis)
−log10(P−value)
Number of variants
Variant consequence
missense
splicing
Effect size
−4
−2
0
2
4
P−value
>0.05
<0.05
<1e−3
<1e−5
<5e−8
<1e−11
Thresholds
Significance Threshold
Total P−value
Lowest P−value

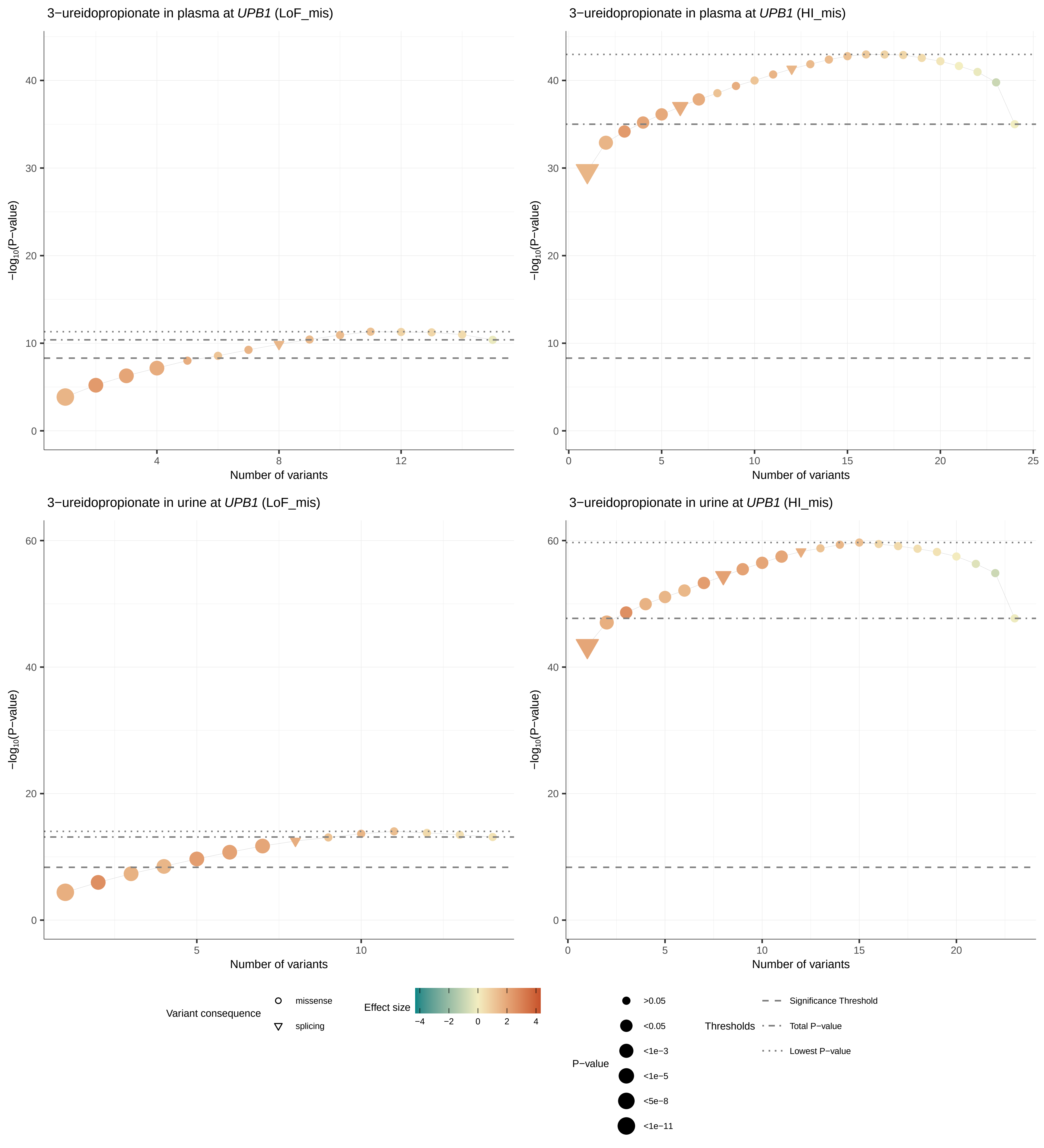
3−ureidopropionate in plasma at UPB1 (LoF_mis)
3−ureidopropionate in plasma at UPB1 (HI_mis)
3−ureidopropionate in urine at UPB1 (LoF_mis)
3−ureidopropionate in urine at UPB1 (HI_mis)
−log10(P−value)
Number of variants
Variant consequence
missense
splicing
Effect size
−4 −2 0 2 4
P−value
>0.05
<0.05
<1e−3
<1e−5
<5e−8
<1e−11
Thresholds
Significance Threshold
Total P−value
Lowest P−value

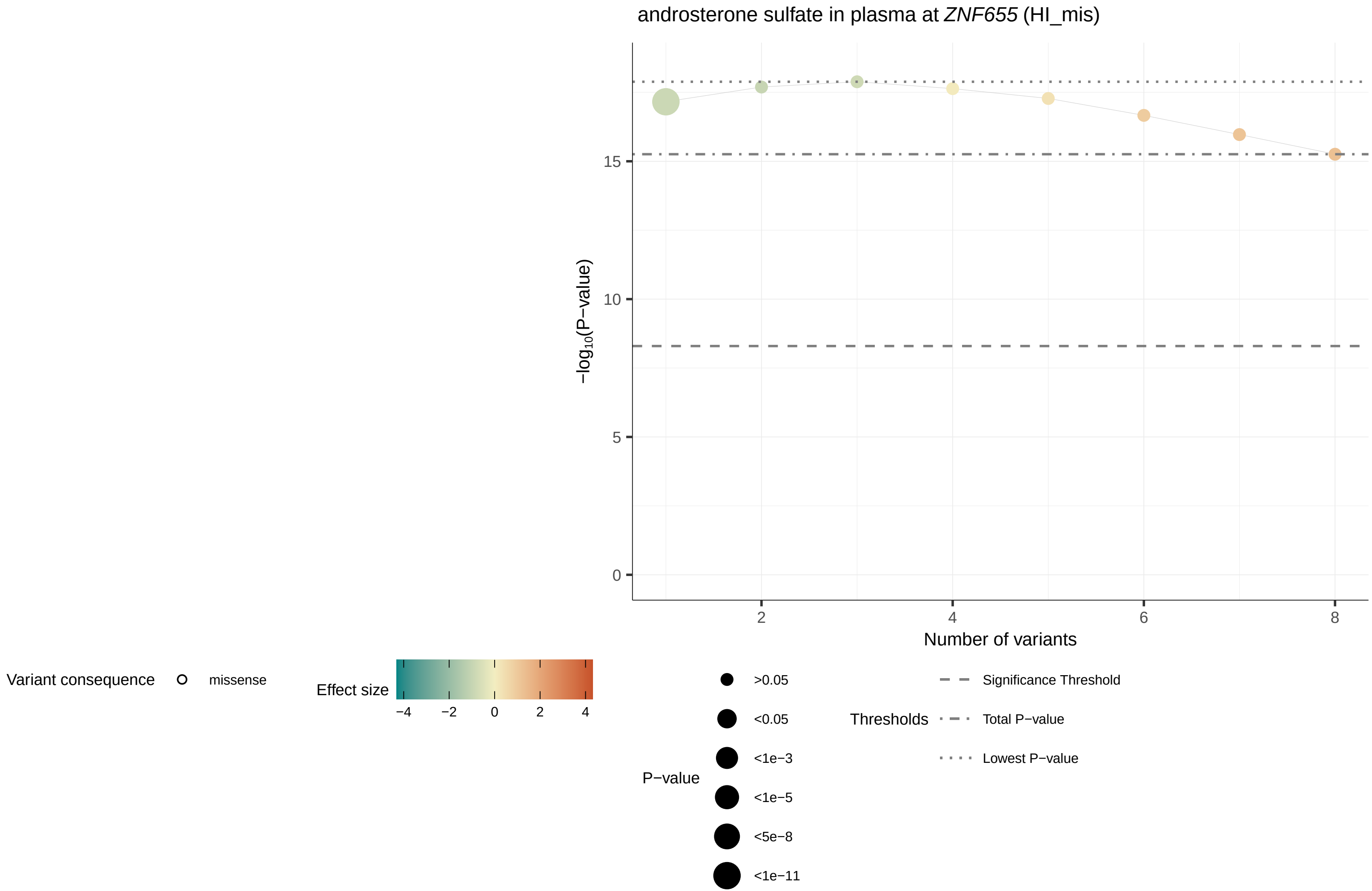

androsterone sulfate in plasma at ZNF655 (HI_mis)
−log10(P−value)
0
5
10
15
2
4
6
8
Number of variants
Variant consequence
missense
Effect size
−4
−2
0
2
4
P−value
>0.05
<0.05
<1e−3
<1e−5
<5e−8
<1e−11
Thresholds
Significance Threshold
Total P−value
Lowest P−value

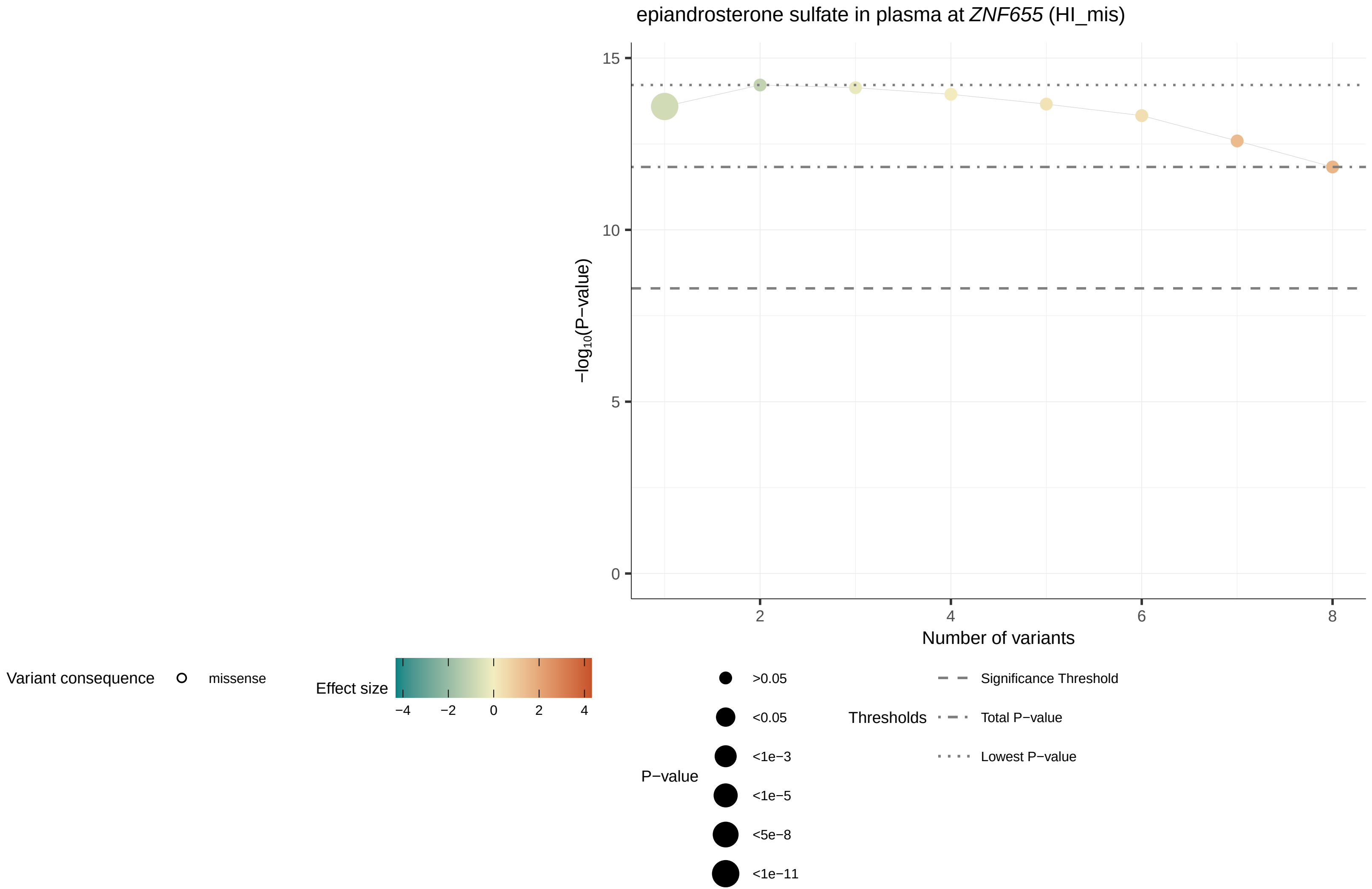
epiandrosterone sulfate in plasma at ZNF655 (HI_mis)
−log10(P−value)
15
10
5
0
2
4
6
8
Number of variants
Variant consequence
missense
Effect size
−4
−2
0
2
4
P−value
>0.05
<0.05
<1e−3
<1e−5
<5e−8
<1e−11
Thresholds
Significance Threshold
Total P−value
Lowest P−value
